# High density information storage through isotope ratio encoding†

**DOI:** 10.1039/d4sc03519d

**Published:** 2024-08-22

**Authors:** Petra Sőregi, Márton Zwillinger, Lajos Vágó, Márton Csékei, Andras Kotschy

**Affiliations:** a Servier Research Institute of Medicinal Chemistry Záhony utca 7 1031 Budapest Hungary andras.kotschy@servier.com; b Hevesy György PhD School of Chemistry, Eötvös Loránd University Pázmány Péter sétány 1/A 1117 Budapest Hungary; c Kastély u. 49/A 2045 Törökbálint Hungary

## Abstract

The need for reliable information storage is on a steep rise. Sequence-defined polymers, particularly oligonucleotides, are already in use in several areas, while compound mixtures also offer a simple way for storing information. We investigated the use of a set of isotopologues in information storage by mixing, where the information is stored in the form of a mass spectrometric (MS) fingerprint of the mixture. A small molecule with 24 non-labile and replaceable hydrogen atoms was selected as a model, and a set of components covering the D_0_–D_24_ deuteration range were synthesized. Theoretical analysis predicted that by mixing up to 10 out of the prepared components, one can encode over 130 million different combinations and distinguish their MS fingerprints. As a proof of principle, several mixtures predicted to have similar fingerprints were prepared and their MS fingerprints were recorded. From each measured MS fingerprint, we were able to unambiguously identify the actual composition of the mixture. It was also demonstrated that one can make the MS fingerprints of a given mixture unique, thereby making counterfeiting of the stored information very difficult. Finally, the utility of isotope ratio encoding in covalent tagging was also demonstrated.

## Abstract 2

Chemical information storage by mixing allowed to encode over 100 Mb of information using isotopologues of a simple organic molecule.

## Graphical abstract

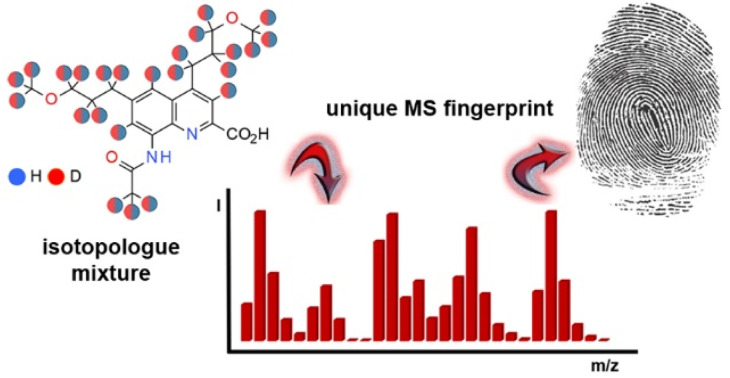

## Introduction

The exponentially increasing need for data storage in our society, as well as the importance of safeguarding sensitive information from tampering, has initiated the exploration of alternative information storage options beyond electronics. The necessity of reliable writing, the expected stability of the stored information, and its accurate reading are just a few of the key requirements that directed the attention to chemical data storage. Nature has evolved its covalently linked, sequence defined macromolecules, nucleotides in particular, with a biochemical toolbox for high density information storage and reliable reading. Thus, it is not surprising that synthetic DNA strands^1–4^ and oligopeptides^5^ were both shown to be useful instruments for information storage. Besides using nature's own building blocks, several reports appeared recently on information encoding in sequence defined polymers using bi- or multifunctional small organic molecules.^6,7^ Here information is converted into a specific sequence of the building block repertoire, which is then written through the synthesis of the given oligomer.^8^ Reading of the information proceeds through the sequencing of the oligomer typically using sophisticated mass spectrometric (MS) analysis including fragmentation,^9,10^ or biotechnological tools.^11^ The quality of information storage in sequence defined oligomers is largely dependent on the reproducibility of the oligomer synthesis and the resolution of the reading method. Sequence defined oligonucleotides have widespread use as covalent tags^12,13^ due to the advanced state of the technology behind their use, while other oligomers, oligopeptides^14^ in particular, are also catching up.

Another emerging approach uses compound mixtures for chemical information storage.^15^ The proper selection of the components, usually all having distinct molecular characteristics (*e.g.* MW^16^ or NMR chemical shift^17^) allows for the simple and reliable writing and reading of the code. Although simpler to execute, the information storage capacity of this approach is below sequence defined polymers'. A specific kind of information storage through mixing uses isotopologues of the same molecule as components.^18^ Varying the isotope composition without changing the molecular formula or structure alters the MS signal of the component but does not impact its other characteristics, which makes it appealing for biological applications. It was also reported recently that isotope ratio encoding can be combined with sequence defined coding.^19,20^ The unique identifiers in this latter method were the MS fingerprints of covalently linked D_0_–D_4_ isotopologue mixtures that could be recorded with standard high-resolution spectrometers. Another, recently published approach involves the use of ^13^C/^12^C isotope ratios to encode information that could be read by NMR spectroscopy.^21^

The sensitivity and reproducibility of the MS measurements suggest that the information storage capacity of isotope ratio encoding might go well beyond that of the other mixing techniques. Here we show that mixing a set of up to ten components resulting from the multi-deuteration (from 0 to 24 deuterium atoms) of the same molecule, allows for the generation of over 130 million different isotopologue combinations whose MS fingerprints are unambiguously distinguishable. We also highlight how varying the extent of deuteration of the individual components gives rise to unique MS fingerprints, making the counterfeiting of the stored information exceedingly difficult.

## Results and discussion

### General concept

**Fig. 1:**
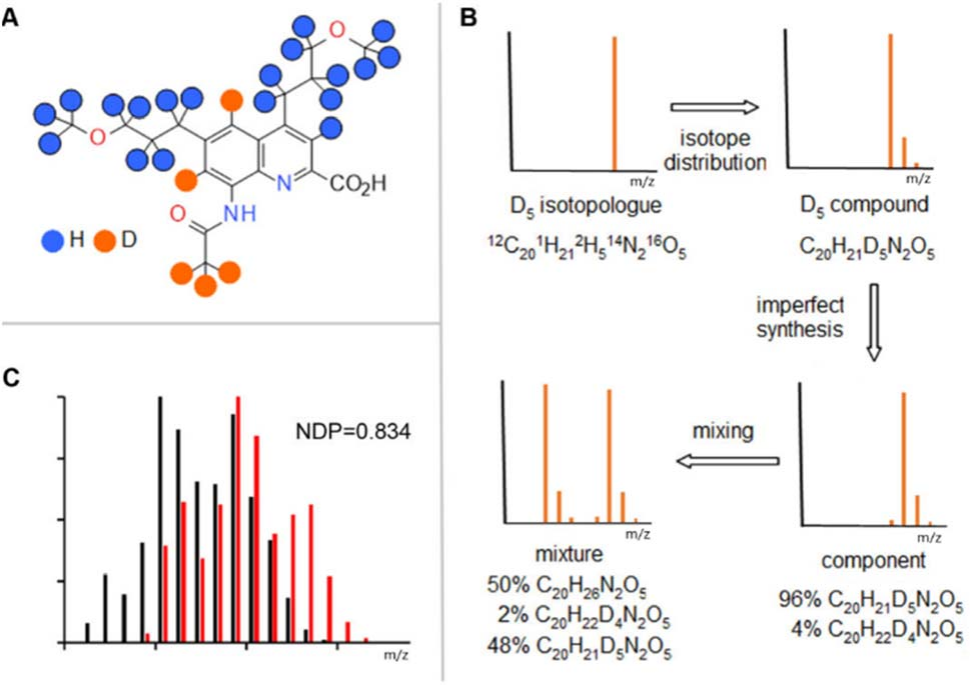
The inherent complexity of isotope ratio encoding. (A) The molecule selected for this study with an arbitrary D_5_ deuteration level. Circles denote positions where D labelling is feasible. (B) The origin of the complexity of MS fingerprints. (C) Quantification of the similarity of two arbitrary MS fingerprints using NDP.

Organic molecules, like our choice for this proof-of-concept study of 8-acetamido-4,6-di(3′-methoxypropyl)-quinoline 2-carboxylic acid (Fig. 1A), typically contain several positionally different hydrogen atoms. Some of these hydrogen atoms, when replaced with deuterium, furnish a chemically stable isotope label (represented by blue and orange dots in Fig. 1A), while others will be replaced by hydrogen in protic media (denoted as H in Fig. 1A). As one can see, 24 of the 26 hydrogen atoms in 1 could be used for deuterium labelling. Replacing some of these hydrogens by deuterium (*e.g.* the 5 represented by orange dots in Fig. 1A) gives another isotopologue. If each atom were a well-defined isotope, the MS trace of this molecule (D_5_ isotopologue) would be a single peak (Fig. 1B, top left). In practice, since all elements that make up our compound have their unique natural isotope distribution, our D_5_ compound's MS spectra is actually more complex as shown in Fig. 1B (top right). This representation still assumes that in the 5 highlighted positions one achieved a 100% deuterium incorporation, which is practically impossible. Even with a very efficient 99% positional deuteration efficiency the proportion of the D_5_ compound will only be around 95% while the rest will be mostly D_4_ compounds. To avoid confusion, in this article we will call the synthesized, and therefore not fully deuterated compounds “components”. Fig. 1B (bottom right corner) shows the MS trace of such a component.

In practical applications of isotope ratio encoding, one stores information in mixtures of components. The MS trace of the mixture, which we will call an MS fingerprint from now on, is the combination of the MS traces of the individual components where their respective intensity is proportional to their fraction in the mixture. Fig. 1B (bottom left) shows how the MS fingerprint of a 1 : 1 mixture of a D_0_ and a D_5_ component evolves from the MS traces of the ingredients. The essence of using isotope ratio encoding is the accurate and reproducible recording of MS fingerprints and the quantitative assessment of their similarity. To this end, we chose the “Normalized Dot Product” function (NDP) that is widely used in proteomics search algorithms,^22–24^ as well as for the comparison of the mass spectra of small molecules.^25,26^ An NDP score can take up a value between 0 and 1, and the closer the NDP value is to 1, the higher the similarity of the MS fingerprints is. To assess the limits of information storage by isotope ratio encoding we selected a molecule (Fig. 1A) that enabled the expansion of the deuteration window to 0–24 deuterium atoms per molecule. Our action plan was relatively straightforward: (1) synthesis of the D_0_–D_24_ components, ideally with a high deuterium incorporation efficiency, (2) exact quantification of the isotopologue composition of components by HRMS, (3) in parallel, theoretical investigation of the information storage capacity by isotope ratio encoding with our isotopologue compound collection, and assessment of how the quality of the prepared components impacts this capacity, (4) selection and application of the mixing rules to prepare mixtures and subsequent analysis of the mixtures by HRMS, (5) proof of principle experiments testing MS pattern recognition and code reading from the prepared mixtures, and (6) looking into the limitations that might arise if high density isotope ratio encoding is used for tagging.

### Assessing the theoretical potential of D_0_–D_24_ isotopologue compound ratio encoding

Our selected model system, 8-acetamido-4,6-bis(3′-methoxypropyl)-quinoline 2-carboxylic acid (1) possesses 24 non-labile hydrogen atoms that can be replaced by deuterium, offering a broad, D_0_–D_24_ deuteration window (Fig. 1A). Its structure also allows for the use of easily accessible building blocks, which can be combined in multiple ways offering a variety of chemical routes to reach the desired deuteration level.

First, we wanted to understand the information storage potential of the theoretical D_0_–D_24_ isotopologue compound collection of 1. To this end, we defined the mixing rules, then created a virtual set of mixtures. We calculated the theoretical isotope fingerprint collection for the given virtual set and calculated the similarity of each pair of isotope fingerprints within the same virtual set. The theoretical isotope fingerprints were generated as a linear combination of the isotope traces of the compounds in the mixture. The isotope trace for any atomic composition was obtained from EnviPat service.^27^ To quantify the similarity of any pair of fingerprints, the NDP function was used, as described above. The smaller the highest NDP value within a virtual set is, the less alike the fingerprints are in a code. Our earlier studies have proven that the laboratory MS equipment can reliably distinguish two fingerprints with a similarity below NDP = 0.9990, therefore this value was set as a benchmark in the modelling. It is trivial that the number of mixtures we can prepare from a given set of isotopologue compounds depends on two factors: the maximum number of components we use within a certain mixture and the proportion size we use for mixing (*i.e.*, 10% or 25% increments). In our system, having 25 isotopologue compounds (D_0_–D_24_), when using up to two components for a given mixture with a ratio of any component being 25% or its multiple (*i.e.*, no component at 0%), we arrive at 900 possible mixtures and the most alike MS fingerprints have an NDP of 0.9573 (Table 1). Decreasing the minimal ratio of a component to 10%, the number of possible mixtures increases to 2700 and the NDP of the most similar fingerprints changes to 0.9945. If we increase the allowed number of isotopologues in a mixture to three, then using the same increment sizes as before, we arrive at 6900 and 82 800 mixtures, respectively (Table 1). It is interesting to note that the larger number of components also results in a decreased similarity with the NDP values of 0.8994 for the 25% and 0.9912 for the 10% mixing. When we use four components from the D_0_–D_24_ compounds in a mixture with a 25% increment, the number of mixtures increases to 12 650 and the similarity of the most alike pair drops to an NDP of 0.8538. When using the same set with a 10% increment, the number of mixtures exceeds 1 million and the NDP is 0.9887. To exploit the maximal potential of the 10% mixing increment we can allow up to 10 different components in any mixture. In this case, the number of possible mixtures increases significantly to 131 128 140 without compromising the quality of the coding. It is not surprising that the most alike mixtures in this code are the binary ones already identified above (Table 1, entry 4) and the NDP value is therefore the same 0.9945. Of course, setting the increment at 10% and allowing only multiple-fold increments are arbitrary restrictions but the vast number of distinguishable mixtures we can generate this way demonstrates the power of this information encoding principle well.

**Table 1:** The theoretical potential of isotope ratio encoding using a collection of D_0_–D_24_ isotopologues of 1 and practical potential using the synthesized isotopologues 1a–y

No. of components	Composition unit	No. of mixtures	Highest NDP using D_0_–D_24_ MS fingerprints	Highest NDP using 1a–y MS fingerprints
2	25%	900	0.9573	0.9857
3	25%	6900	0.8994	0.9813
4	25%	12 650	0.8538	0.9739
2	10%	2700	0.9945	0.9984
3	10%	82 800	0.9912	0.9981
4	10%	1 062 600	0.9887	0.9976
1–10	10%	131 128 140	0.9945	0.9984
1–10^a^	10%	131 128 140	0.9992	nd

a Calculated for C_182_H_224_N_20_O_32_.

In applications where the isotope ratio encoding is used as a tagging method the inherent isotope distribution of the tagged molecules can interfere with the coding. To simulate such a situation, we increased the formula of our D_0_–D_24_ isotopologues to a decamer's (C_182_H_224_N_20_O_32_) from the original monomer's (C_20_H_26_N_2_O_5_). Indeed, when we create mixtures of up to 10 components with an increment of 10% using these decamers with increased atomic numbers, the most similar mixtures will have an NDP of 0.9992 that might be difficult to distinguish reliably. On the other hand, when increasing the increment size in the mixing to 20%, which still enables us to code 118 755 molecules, the NDP fell to 0.9966, which is well within our applicability range. Of course, this theoretical scenario implies that we want to distinguish over a 100 thousand molecules of the same atomic composition, which is more stringent than real applications. As soon as the molecules we want to distinguish by tagging cover a more diverse set of atomic compositions, we can proportionally increase the number of the entities we can code by tagging.

### Synthesis of D_0_–D_24_ components

**Scheme 1:**
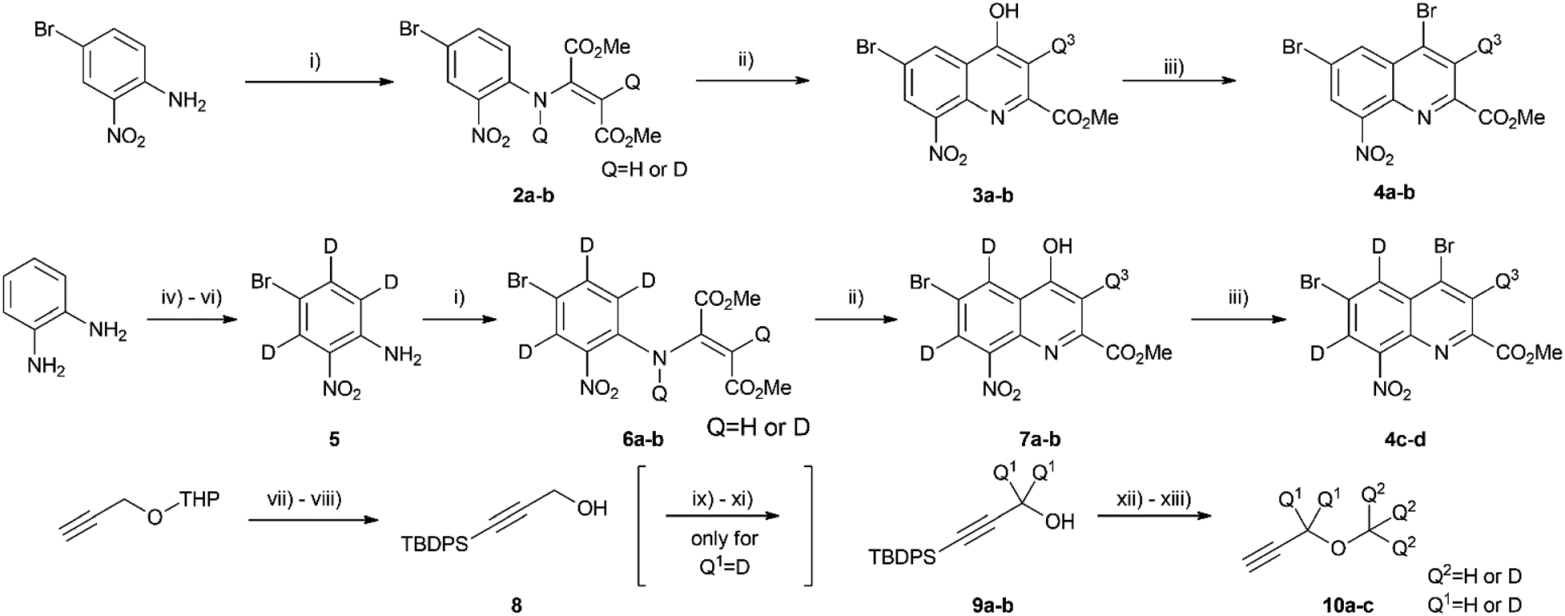
Synthesis of the deuterated building blocks. (i) DMAD, MeOH/MeOD, 60 °C, 48 h – 8 d; (ii) P_2_O_5_, H_2_O/D_2_O, 150 °C, 5 h; (iii) POBr_3_, DCE, 80 °C, 50–120 min; (iv) HCl·EtOH, r.t., 15 min, then D_2_O, MW, 150 °C, 3 × 30 min; (v) *m*CPBA, MeCN, 0 °C, 15 min; (vi) NBS, AcOH, 50 °C, 90 min; (vii) *n*-BuLi, THF, −78 °C – r.t., then TBDPS-Cl, −30 °C – r.t., 18 h; (viii) *p*TsOH·H_2_O, MeOH, r.t., 1 h; (ix) Jones' reagent, acetone, 0 °C, 1 h; (x) TMS-CHN_2_, DCM, 0 °C, 1 h; (xi) LiAlD_4_, THF, −78 °C, 10 min; (xii) NaH, CD_3_I, THF, r.t., 1 h; (xiii) TBAF, 2-Me-THF, r.t., 18 h.

**Scheme 2:**
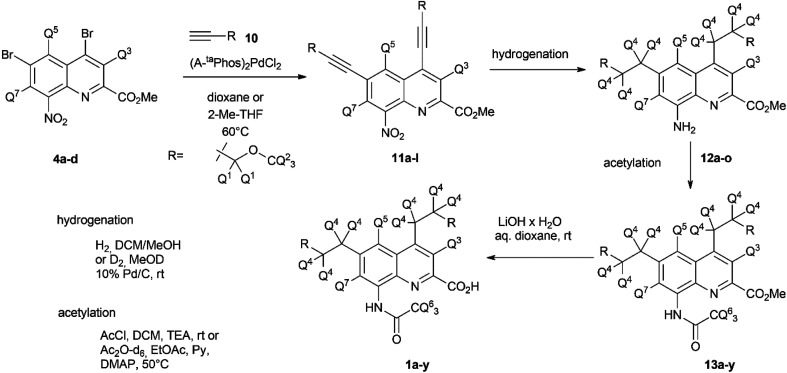
Construction of the different isotopologues (1a–y) used in this study.

Once the theoretical information storage potential of the D_0_–D_24_ compound collection had been established, we moved on to its practical implementation. The initial step involved synthesizing these compounds with high deuterium incorporation. Compound 1 can be deconstructed into three principal building blocks, each capable of carrying a defined number of deuterium atoms. The first building block, methyl 4,6-dibromo-8-nitroquinoline-2-carboxylate (4), served as the core molecule with the capacity to accommodate 0 to 3 deuterium atoms. The second building block, methyl propargyl ether (10) can be introduced into positions 4 and 6 *via* the bromine atoms of 4 and functions as the side chain allowing the introduction of up to 9 additional deuterium atoms per position. Lastly, the acetylation of the amine group enabled the introduction of either 0 or 3 deuterium atoms, depending on the acylating agent used (Scheme 1). By combining these building blocks appropriately, a pathway was established for synthesizing all deuterated compounds required for this study. Modifying a previously described synthesis strategy,^20^ compounds 4a–d were successfully prepared, achieving an outstanding 96–97% positional deuterium incorporation. Although methyl propargyl ether is commercially available, the synthesis of deuterated analogues was necessary. This involved introducing three deuterium atoms to form a CD_3_ moiety using CD_3_I on the suitable protected propargyl alcohol (9), demonstrating excellent, 99+% incorporation efficiency. Additionally, implementing an oxidation–reduction sequence of the methylene moiety resulted in the CD_2_ linker with an impressive 98% positional deuterium incorporation (Table 2). After obtaining the necessary building blocks, all 25 components were synthesized. The desired deuteration level of each component (1a–y) was achieved by combining appropriately deuterated and non-deuterated reagents, as outlined in the ESI (Table S1).† The synthesis process began with the double Sonogashira coupling between compounds 4 and 10, yielding compound 11 (Scheme 2). Simultaneous saturation of the triple bond and reduction of the nitro group using a Pd/C catalyst and hydrogen or deuterium gas produced compound 12. This saturation step provided an opportunity to incorporate 8 deuterium atoms into the side chains, resulting in 94% positional deuterium incorporation, cumulatively accounting for 61% across the 8 positions. During deuteroacetylation, a small decrease in the deuterium content of the reagent's acetyl group was observed, likely occurring in its activated form. This deuterium content might be improved from 94% to 97% by replacing the labile hydrogens in the reagents with deuterium prior to the addition of the acetylating agent (see ESI† for details). Finally, hydrolysis yielded the desired components 1a–y.

**Table 2:** The typical per atom and cumulative (global) deuterium incorporation efficiency in the different positions of the building blocks used to prepare the isotopologues 1a–y

	Q^1^	Q^2^	Q^3^	Q^4^	Q^5^	Q^6^	Q^7^
Deuteration efficiency	98%	99+%	96%	94%	97%	94% (97%^a^)	97%
Cumulated deuteration	96%	97%	96%	61%	97%	83% (91%^a^)	97%

a With improved method.

Since the combined effect of natural isotope abundance and enrichment of specific isotopologues leads to complex MS patterns, a problem identified and treated in metabolomic studies,^28,29^ the exact isotopologue distribution of 1a–y was deconvoluted from their mass spectra (for details see ESI and Table S2†). As expected, achieving 100% incorporation efficiency in every reaction and position was not feasible, and this influenced the composition of the products, demonstrating the necessity of rerunning the calculation on the theoretical studies once the components are synthesized and qualified. Initially, our goal was to understand how the actual isotopologue composition affects the effectiveness of coding. Subsequently, we aimed to assess the performance of the encoding method by preparing specific component mixtures from 1a–y, recording their experimental MS spectra, and determining if we could accurately determine the mixtures' composition by comparing the recorded MS fingerprints with the collection of calculated fingerprints.

### Coding and reading information in isotopologue mixtures

To establish how the real isotopologue composition of components 1a–y impacts the efficacy of the coding method the MS fingerprints of all the different monomeric mixtures that were present in Table 1 were calculated and the NDP of the most similar mixtures were identified within all sets. The results, shown in Table 1 (right column), clearly demonstrate what one might also expect intuitively. Since the individual components 1a–y contain more than one member of the D_0_–D_24_ set, their encoding power slightly decreases, leading to increased similarity. Although the increase of the NDP values is apparent for all codes (*i.e.*, 0.9945 to 0.9984 for binary, 0.9912 to 0.9981 for ternary, and 0.9887 to 0.9976 for quaternary mixtures using 10% mixing increments) it was promising that the threshold of the MS fingerprint reproducibility (0.9990) has not been reached. After calculating the 25 single components', 2700 binary mixtures', 82 800 ternary mixtures' and 1 062 600 quaternary mixtures' MS fingerprints (1 148 125 in total), arising from mixing 1a–y, 22 examples were found, where the similarity to the closest analogue was greater than 0.9980, while 5174 mixtures gave an NDP between 0.9980 and 0.9970. Interestingly, while for most mixtures the highest similarity relationship was reciprocal (*i.e.*, mixture Mx was the most similar to mixture My, and *vice versa*), this was not a general rule. We selected and prepared all 22 mixtures where the predicted similarity with the most alike mixture was above 0.9980 (binary and ternary mixtures) and several other ternary and quaternary mixtures with a predicted similarity above 0.9970 (34 in total, see Table S3† for details). For simplicity, here we present 4 of the binary mixtures (M1–M4) and 5 of the ternary mixtures (M5–M9), where the predicted similarity was amongst the highest and 5 of the quaternary mixtures (M10–M14). The composition of the mixtures is shown in Table 3. This table also lists the composition and similarity of each mixture to its closest analogue based on their calculated MS spectra.

**Table 3:** The composition of the mixtures M1–M34 selected for the proof-of-concept studies, the calculated similarity and composition of the most difficult to distinguish mixtures based on their calculated and measured MS fingerprint, as well as the similarity of the calculated and measured MS fingerprints for the given mixture

Mixture	Composition	Closest analogue and NDP based on calculated MS fingerprints	NDP of calculated and measured MS fingerprints	Closest analogue and NDP based on measured MS fingerprints
M1	1i (0.9) – 1n (0.1)	M2 0.9982	M1 1.0000	M2 0.9983
M2	1i (0.9) – 1m (0.1)	M1 0.9982	M2 1.0000	M1 0.9982
M3	1a (0.9) – 1n (0.1)	M4 0.9983	M3 0.9997	M4 0.9984
M4	1a (0.9) – 1m (0.1)	M3 0.9983	M4 0.9998	M3 0.9985
M5	1g (0.8) –1h (0.1)– 1n (0.1)	M6 0.9981	M5 0.9998	M6 0.9983
M6	1g (0.8) –1h (0.1)– 1m (0.1)	M5 0.9981	M6 0.9995	M5 0.9979
M7	1a (0.8) –1b (0.1)– 1n (0.1)	M8 0.9981	M7 0.9987	M8 0.9980
M8	1a (0.8) –1b (0.1)– 1m (0.1)	M7 0.9981	M8 0.9991	M7 0.9976
M9	1d (0.1) –1l (0.5)– 1m (0.4)	M10 0.9975	M9 0.9998	M10 0.9975
M10	1d (0.1) –1l (0.5)– 1m (0.3)– 1n (0.1)	M9 0.9975	M10 0.9996	M9 0.9968
M11	1a (0.1)– 1g (0.7)– 1l (0.1)– 1n (0.1)	M12 0.9974	M11 0.9998	M12 0.9971
M12	1a (0.1)– 1g (0.7)– 1l (0.1)– 1m (0.1)	M11 0.9974	M12 0.9999	M11 0.9976
M13	1a (0.6) – 1b (0.2) – 1n (0.1) – 1u (0.1)	M14 0.9971	M13 0.9986	M14 0.9961
M14	1a (0.6) – 1b (0.2) – 1m (0.1) – 1u (0.1)	M13 0.9971	M14 0.9978	1a (0.7) – 1b (0.1) – 1m (0.1) – 1u (0.1) 0.9958
M35	1a (0.1)– 1b (0.1)– 1e (0.1)– 1i (0.1)– 1l (0.1)– 1n (0.1)– 1r (0.1)– 1s (0.1)– 1v (0.1)– 1y (0.1)	Nd	M35 0.9923	

To establish the optimal conditions for code reading by MS, we prepared a dilution series of 1a in acetonitrile, determined the linearity range of the detector, and adjusted the injected quantities to fit within that range. Mixtures were prepared by the volumetric dispensation of stock solutions in propionitrile. The MS fingerprints were recorded following dilution to the desired concentration range.

First, the measured and theoretical MS fingerprints of M1–M34 were compared (Table 3) to assess the impact of the experimental error from mixing on the coding. It was reassuring to see that most of the NDPs exceeded 0.9990, and the lowest one (M14) was still 0.9978. Next, the measured MS fingerprints of M1–M34 were compared to the database of 1 148 125 calculated MS fingerprints searching for the most similar ones for each of them. For reliable information encoding the measured fingerprint must unambiguously match its calculated equivalent. Our results showed that for all the prepared and experimentally characterized mixtures (M1–M34) the composition could be identified with high certainty because the most similar calculated fingerprints always matched the experimental compositions. This result proves that isotope ratio encoding is an efficient tool to write and read information. An interesting indicator of the reliability of information reading from an isotope mixture is the separation between the most similar and the second most similar calculated MS fingerprints when compared to the measured one. In the M1–M34 set, the NDP values for the second most similar fingerprints were in the range of 0.9951–0.9985, all showing a considerable separation from the first hit (Table 3). It is interesting to note that 11 out of the 14 predicted closest analogues (mixtures with the second most similar calculated MS fingerprint) were identified both by using the calculated or the measured MS fingerprint of the primary mixture. For the four mixtures, M14, M31, M33 and M34 the closest analogues identified by using their measured or the calculated MS fingerprints were very similar, deferring in only a single component present in 10% (for details see Table S3†). The high similarity between the calculated and measured MS fingerprints for M1–M34 suggests that reliable information storage with these mixtures is feasible. On the other hand, these mixtures contained only 4 components, which is well below the potential of the method. To evaluate the robustness of mixing and the potential impact of variations in the volumetric dispensing, the 10-component mixture M35 was also prepared. Its MS fingerprint was recorded and compared with the calculated fingerprint based on its composition (see ESI† for details). The obtained similarity was 0.9923, which is somewhat lower than for the mixtures with fewer components. On the other hand, it was already shown (Table 1) that the more components the mixture has the less similar its closest analogue will be. Comparison of the measured MS fingerprint of M35 with the calculated MS fingerprints of potential close analogues enabled the unambiguous identification of its composition, reaffirming the power of this information encoding method.

### Specificity of the code

**Fig. 2:**
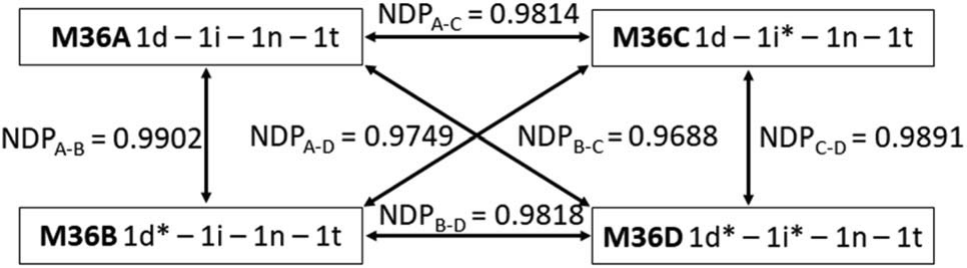
Similarity of the 1d(0.25)-1i(0.25)-1n(0.25)-1t(0.25) mixture (M36A) and its 1d* and 1i* containing analogues (M36B–D).

The necessity of reproducible information writing and reading makes the information stored in molecules or their mixtures inherently easy to copy once read since the deconvolution of the mixture composition from the readout is usually straightforward. In case of isotope ratio encoding the correct deconvolution of the MS fingerprint (*i.e.*, its conversion into a mixing ratio) would either require a collection of isotopologues with a 100% deuteration or the knowledge of the exact composition and therefore MS fingerprint of all the components used in the process. The safety and non-reproducibility of the stored information can further be enhanced by the introduction of an element of uncertainty into the chemical code, which should be exact and difficult to reproduce at the same time. The isotopologue components and their mixtures offer this possibility because we can induce difficult-to-reproduce variations in the positional deuteration level of our components that will translate into a specific change of the fingerprint. To demonstrate that, components 1d and 1i were resynthesized with a different composition to yield 1d* and 1i* (Table S1†). By modifying the deuteroacetylation conditions the 94% proportion of the major D_3_ isotopologue in 1d was changed to 65% in 1d*. In 1i the major D_8_ isotopologue (Q^2^, Q^5^, Q^7^ = D, Q^4^ = H) was present in 94%, while the isotopomeric 1i* contained only 62% D_8_ (Q^2^, Q^5^, Q^7^ = H, Q^4^ = D). In mixtures containing 1d and 1i either one or both can be replaced by 1d* and 1i*, respectively. For example, changing 1d to 1d* in a 1d(0.25)-1i(0.25)-1n(0.25)-1t(0.25) mixture (M36A) resulted M36B, and the similarity between M36A and M36B was 0.9902, which, while quite high, but can be distinguished reliably. Replacing 1i with 1i* gave M36C that gave an NDP value of 0.9814 when compared to M36A, again quite similar but distinguishable. Finally, when both 1d and 1i were replaced by 1d* and 1i*, respectively (M36D), the similarity score dropped to 0.9688, as expected. These results prove that fluctuations in the deuteration efficiency, particularly if encouraged by design, translate into detectable differences in the MS fingerprint of a mixture, which is highly reproducible when one has a stock of the components, but otherwise might be difficult to forge (Fig. 2).

### Chemical tagging

**Scheme 3:**
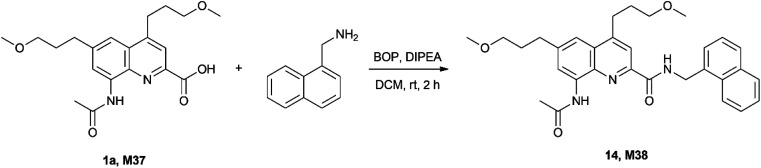
Test reaction used to assess the utility of the information storage as a covalent tag (deuteriums not shown).

Finally, we were interested to know whether isotope ratio encoding is compatible with chemical tagging. The principal question is whether the integrity of the isotope content is maintained in the process. To this end the quaternary mixture of 1a (0.28)–1g (0.24)–1k (0.24)–1s (0.24) (M37) was prepared and its MS fingerprint was recorded. Then it was coupled with 1-aminomethyl-naphthalene as a simple example using standard conditions (Scheme 3).

This transformation was selected because amide coupling is by far the most frequently used chemical route for covalent tagging due to its robustness. Based on the MS fingerprint of the isotopologue mixture M37 and of the amine, the theoretical MS fingerprint was calculated for the product M38 and compared with the measured MS fingerprint (see ESI† for details). We were delighted to find that the measured and the calculated MS fingerprints of the tagged molecule showed a remarkably high similarity (NDP = 0.9993), well within the range we observed previously (*cf.*Table 3), therefore proving that the amide coupling did not impair the isotope ratio code.

## Conclusions

Isotope ratio encoding coupled with mass spectrometric detection is a powerful way of writing and reading information using simple molecules. We selected the aminoquinoline carboxylic acid derivative 1, which has 24 non-labile hydrogen atoms that could be replaced by deuterium, and studied the information encoding capacity that can be achieved by mixing its isotopologues. Theory predicted that using up to 10 out of the D_0_–D_24_ isotopologues, one can encode and distinguish more than 130 million different combinations. To put theory into practice we have elaborated the synthesis of isotope labelled analogues of all the key building blocks of 1 with high deuteration efficiency, and with the appropriate combination of the deuterated and non-deuterated building blocks we prepared the 25 components (1a–y) and established their actual isotope composition. Having adapted the mixture similarity predictions to the actual components 1a–y, we selected and prepared several binary, ternary, and quaternary mixtures with a high predicted similarity (NDP > 0.9970), registered their MS fingerprints and compared them with the predictions. In every case, we were able to identify the actual composition of the mixture unambiguously, which proves the power of this encoding method. We also demonstrated the potential utility of isotope ratio encoding in covalent tagging. These results open the way for the application of isotope ratio encoding in diverse areas where high density information encoding by chemical means could be beneficial.

## Author contributions

Petra Sőregi: conceptualization, data curation, formal analysis, funding acquisition, investigation, methodology, writing – original draft. Márton Zwillinger: conceptualization, formal analysis, methodology, writing – review & editing. Lajos Vágó: software. Márton Csékei: conceptualization, project administration, supervision, writing – review & editing. András Kotschy: conceptualization, project administration, supervision, writing- original draft, review & editing.

## Conflicts of interest

There are no conflicts to declare.

## Supplementary Material

SC-015-D4SC03519D-s001

## Data Availability

The data supporting this article have been included as part of the ESI.† The code for calculations related to MS fingerprints can be found at https://github.com/VagoLali/isotopeRatioEncoding.
