# Supplementary material for: High density information storage through isotope ratio encoding
Source: Chem Sci. 2024 Aug 22;15(36):14938–45. doi: 10.1039/d4sc03519d (PMC11376023; doi:10.1039/d4sc03519d)

Supplementary Information (SI) for Chemical Science.
This journal is © The Royal Society of Chemistry 2024

High density information storage through isotope ratio encoding

Petra Sőregi,[a,b] Márton Zwillinger,[a] Lajos Vágó,[c] Márton Csékei,*[a] and Andras Kotschy*[a]

[a]Servier Research Institute of Medicinal Chemistry Záhony utca 7, 1031 Budapest, Hungary
[b]Hevesy György PhD School of Chemistry, Eötvös Loránd University, Pázmány Péter sétány 1/A, 1117 Budapest, Hungary
[c]Kastély u. 49/A, 2045 Törökbálint, Hungary

Table of Contents

1. Tables and Figures S2
2 Calculations S5
3 General information S6
4 Experimental data S7
4.1 General procedures S7
4.1.1 General procedure 1: Hydroxy-bromo exchange S7
4.1.2 General procedure 2A: Sonogashira coupling with methyl propargyl ether S7
4.1.3 General procedure 2B: Sonogashira coupling with silyl protected alkynes S7
4.1.4 General procedure 3: NO_2 to NH_2 group reduction S8
4.1.5 General procedure 4A: Hydrogenation reaction S8
4.1.6 General procedure 4B: Deuteration reaction S8
4.1.7 General procedure 5A: Acylation reaction S8
4.1.8 General procedure 5B: Deuteroacylation reaction S8
4.1.9 General procedure 6: Methyl ester hydrolysis S9
4.2 Synthetic procedure and analytical data of the newly synthesized compounds in this work S9
4.3 NMR spectra of newly synthesized compounds S50
4.4 HR-MS measurements and evaluation of the 1a-1y series S136
4.4.1 Conditions for HRMS measurements S136
4.4.2 HR-MS measurements of 1a-1y S137
4.5 HR-MS measurements of prepared mixtures S144
4.6 Amide coupling: procedure, HRMS measurements S153

1. Tables

Table S1. Selected combination of deuterated building blocks to achieve desired deuteration levels.

	Q^1	Q^2	Q^3	Q^4	Q^5	Q^6	Q^7
**1b**			1				
**1c**					1		1
**1d**			1		1		1
**1d***						3	
**1e**			1			3	
**1f**					1	3	1
**1g**		6					
**1h**		6	1				
**1i**		6			1		1
**1i***				8			
**1j**		6				3	
**1k**		6	1			3	
**1l**		6			1	3	1

	Q^1	Q^2	Q^3	Q^4	Q^5	Q^6	Q^7
**1m**			1	8		3	
**1n**				8	1	3	1
**1o**		6		8			
**1p**		6	1	8			
**1q**		6		8	1		1
**1r**		6		8		3	
**1s**		6	1	8		3	
**1t**		6		8	1	3	1
**1u**		6	1	8	1	3	1
**1v**	4	6		8		3	
**1w**	4	6	1	8		3	
**1x**	4	6		8	1	3	1
**1y**	4	6	1	8	1	3	1

Table S2. The isotopologue composition of 1a-y calculated from Table S4 and Table S5.

	D0	D1	D2	D3	D4	D5	D6	D7	D8	D9	D10	D11	D12	D13	D14	D15	D16	D17	D18	D19	D20	D21	D22	D23	D24
**1a**	100.0																								
**1b**	3.9	96.1																							
**1c**	0.5	4.7	90.8	2.3	1.6																				
**1d**	0.4	1.3	15.5	82.8																					
**1d***	0.4	4.7	29.8	65.1																					
**1e**	0.2	0.4	1.9	18.5	79.0																				
**1f**	0.1	0	0.2	1.7	16.1	80.2	1.6	0.2																	
**1g**	0	0	0	0	0.1	0.8	99.1																		
**1h**	0.1	0.5	0.1	0.1	0.1	0.1	4.0	94.9																	
**1i**	0	0	0.2	0	0	0.1	0.2	3.0	94.6	1.8															
**1i***	0	0	0	0.2	0.2	0.7	7.0	20.4	62.5	8.0	0.9	0.2													
**1j**	0.1	0.1	0.0	0.1	0.1	0.1	0.3	2.4	20.2	76.6															
**1k**	0	0	0	0	0	0.1	0	0.5	4.2	25.4	69.9														
**1l**	0	0	0	0	0	0	0.1	0.1	0.8	5.2	28.0	64.6	1.0	0.2	0.1										
**1m**	0	0	0	0	0	0.1	0.1	0.2	0.6	2.6	10.9	32.7	48.2	3.6	0.8	0.2	0.1								
**1n**	0.1	0.1	0.1	0.2	0.1	0.1	0	0.1	0.3	0.4	2.4	11.1	33.3	48.6	2.5	0.6	0.1								
**1o**	0	0	0	0	0.2	0	0	0	0.2	0.3	0.6	3.2	6.1	15.2	67.9	5.0	1.1	0.2	0.1						
**1p**	0	0	0	0	0	0	0	0	0.1	0.1	0.1	0.3	1.0	5.4	20.1	61.8	8.1	2.3	0.5	0.2	0.1				
**1q**	0	0	0	0	0	0	0	0	0	0	0	0.2	0.2	0.3	2.1	15.6	73.9	5.8	1.6	0.2	0.1				
**1r**	0	0	0	0	0	0	0	0.1	0	0.1	0.1	0.2	0.2	0.5	1.4	7.2	27.2	58.5	3.5	0.8	0.2	0.1			
**1s**	0	0	0	0	0	0	0	0	0	0	0	0.1	0	0.1	0.4	1.7	7.9	25.4	54.3	7.4	1.9	0.4	0.2	0.1	
**1t**	0	0	0	0	0	0	0	0.1	0.1	0.1	0	0.1	0	0.1	0.1	0.2	0.8	4.5	22.0	67.8	3.0	0.8	0.1	0.1	
**1u**	0	0	0	0	0	0	0	0.1	0.1	0.1	0	0.1	0	0	0.1	0.3	0.5	1.4	6.9	28.2	60.0	1.5	0.5	0.2	0.1
**1v**	0	0	0	0	0	0	0	0	0	0	0	0	0.1	0	0.1	0.2	0.6	1.1	2.1	9.5	32.1	53.5	0.3	0.3	0.1
**1w**	0	0	0	0	0	0	0	0	0	0	0	0	0.1	0	0	0	0	0.1	0.2	1.1	6.3	28.0	64.2		
**1x**	0	0	0	0	0	0	0	0	0	0	0	0	0	0	0	0	0.1	0.1	0.1	0.3	1.7	9.0	33.2	55.5	
**1y**	0	0	0	0	0	0	0	0	0	0	0	0	0	0	0	0	0	0	0	0.1	0.3	1.5	7.8	30.6	59.8

Table S3. M1-M35 prepared mixtures and their calculated NDPs.

mixture	Composition	Closest analogue and NDP based on calculated MS fingerprints	NDP of calculated and measured MS fingerprint	Closest analogue and NDP based on measured MS fingerprint
**M1**	**1i** (0.9) – **1n** (0.1)	**M2** 0.9982	**M1** 1.0000	**M2** 0.9983
**M2**	**1i** (0.9) – **1m** (0.1)	**M1** 0.9982	**M2** 1.0000	**M1** 0.9982
**M3**	**1a** (0.9) – **1n** (0.1)	**M4** 0.9983	**M3** 0.9997	**M4** 0.9984
**M4**	**1a** (0.9) – **1m** (0.1)	**M3** 0.9983	**M4** 0.9998	**M3** 0.9985
**M5**	**1g** (0.8) -**1h** (0.1)- **1n** (0.1)	**M6** 0.9981	**M5** 0.9998	**M6** 0.9983
**M6**	**1g** (0.8) -**1h** (0.1)- **1m** (0.1)	**M5** 0.9981	**M6** 0.9995	**M5** 0.9979
**M7**	**1a** (0.8) -**1b** (0.1)- **1n** (0.1)	**M8** 0.9981	**M7** 0.9987	**M8** 0.9980
**M8**	**1a** (0.8) -**1b** (0.1)- **1m** (0.1)	**M7** 0.9981	**M8** 0.9991	**M7** 0.9976
**M9**	**1d** (0.1) -**1l** (0.5)- **1m** (0.4)	**M10** 0.9975	**M9** 0.9998	**M10** 0.9975
**M10**	**1d** (0.1) -**1l** (0.5)- **1m** (0.3)- **1n** (0.1)	**M9** 0.9975	**M10** 0.9996	**M9** 0.9968
**M11**	**1a** (0.1)- **1g** (0.7)- **1l** (0.1)- **1n** (0.1)	**M12** 0.9974	**M11** 0.9998	**M12** 0.9971
**M12**	**1a** (0.1)- **1g** (0.7)- **1l** (0.1)- **1m** (0.1)	**M11** 0.9974	**M12** 0.9999	**M11** 0.9976
**M13**	**1a** (0.6) – **1b** (0.2) – **1n** (0.1) – **1u** (0.1)	**M14** 0.9971	**M13** 0.9986	**M14** 0.9961
**M14**	**1a** (0.6) – **1b** (0.2) – **1m** (0.1) – **1u** (0.1)	**M13** 0.9971	**M14** 0.9978	**1a** (0.7) – **1b** (0.1) – **1m** (0.1) – **1u** (0.1) 0.9958
**M15**	**1l** (0.9)- **1n** (0.1)	**M16** 0.9981	**M15** 0.9999	**M16** 0.9984
**M16**	**1l** (0.9)- **1m** (0.1)	**M15** 0.9981	**M16** 1.0000	**M15** 0.9979
**M17**	**1g** (0.9)- **1n** (0.1)	**M18** 0.9983	**M17** 0.9996	**M18** 0.9984
**M18**	**1g** (0.9)- **1m** (0.1)	**M17** 0.9983	**M18** 0.9998	**M17** 0.9982
**M19**	**1g** (0.9)- **1l** (0.1)	**M18** 0.9981	**M19** 0.9996	**M18** 0.9982
**M20**	**1g** (0.8) -**1f** (0.1)- **1n** (0.1)	**M21** 0.9980	**M20** 0.9998	**M21** 0.9981
**M21**	**1g** (0.8) -**1f** (0.1)- **1m** (0.1)	**M20** 0.9980	**M21** 0.9997	**M20** 0.9980
**M22**	**1a** (0.9) – **1l** (0.1)	**M4** 0.9981	**M22** 0.9997	**M4** 0.9983
**M23**	**1b** (0.9) – **1n** (0.1)	**M24** 0.9982	**M23** 0.9997	**M24** 0.9981
**M24**	**1b** (0.9) – **1m** (0.1)	**M23** 0.9982	**M24** 0.9998	**M23** 0.9978
**M25**	**1c** (0.9) – **1n** (0.1)	**M26** 0.9981	**M25** 0.9998	**M26** 0.9982
**M26**	**1c** (0.9) – **1m** (0.1)	**M25** 0.9981	**M26** 0.9998	**M25** 0.9982
**M27**	**1h** (0.9) – **1n** (0.1)	**M28** 0.9982	**M27** 0.9995	**M28** 0.9980
**M28**	**1h** (0.9) – **1m** (0.1)	**M27** 0.9982	**M28** 0.9997	**M27** 0.9980
**M29**	**1h** (0.7)- **1n** (0.1)- **1u** (0.1)- **1x** (0.1)	**M30** 0.9972	**M29** 0.9999	**M30** 0.9969
**M30**	**1h** (0.7)- **1m** (0.1)- **1u** (0.1)- **1x** (0.1)	**M29** 0.9972	**M30** 0.9998	**M29** 0.9970
**M31**	**1a** (0.1) – **1i** (0.7) – **1k** (0.1) – **1m** (0.1)	**M32** 0.9973	**M31** 0.9986	**1a** (0.1) – **1i** (0.7) – **1k** (0.1) – **1l** (0.1) 0.9960
**M32**	**1a** (0.1) – **1i** (0.7) – **1k** (0.1) – **1n** (0.1)	**M31** 0.9973	**M32** 0.9988	**M31** 0.9965
**M33**	**1b** (0.7) – **1m** (0.1) – **1v** (0.1) – **1y** (0.1)	**M34** 0.9972	**M33** 0.9980	**1b** (0.6) – **1m** (0.1) – **1v** (0.1) – **1y** (0.2) 0.9951
**M34**	**1b** (0.7) – **1n** (0.1) – **1v** (0.1) – **1y** (0.1)	**M33** 0.9972	**M34** 0.9983	**1b** (0.6) – **1n** (0.1) – **1v** (0.2) – **1y** (0.2) 0.9953
**M35**	**1a** (0.1)- **1b** (0.1)- **1e** (0.1)- **1i** (0.1)- **1l** (0.1)- **1n** (0.1)- **1r** (0.1)- **1s** (0.1)- **1v** (0.1)- **1y** (0.1)	nd	**M35** 0.9923	**1a** (0.1)- **1b** (0.1)- **1e** (0.1)- **1i** (0.1)- **1l** (0.1)- **1m** (0.1)- **1r** (0.1)- **1s** (0.1)- **1v** (0.1)- **1y** (0.1) 0.9792

2 Calculations

The program and data input files referenced below are available at https://github.com/VagoLali/isotopeRatioEncoding

Isotopologue distribution of components 1a-y
The composition of **1a-y** were deconvoluted from their measured mass spectra manually by sequential subtraction of the underlying patterns using the theoretical spectra of D_0-D_{24} compounds as elements of the combination. The theoretical spectra of the D_0-D_{24} compounds were calculated using enviPat from Eawag3 available at the https://envipat.eawag.ch/website.

Theoretical spectra of mixtures
The theoretical spectra of each mixture in a study were calculated and stored by the attached python code ("isotopic_decomposition.py", called "software" from now on). A theoretical spectrum of a given mixture was calculated as a linear combination of the theoretical spectra corresponding to its elements (D_0-D_{24}), weighed by the respective ratios of elements present in the mixture.
*For example the mixture **1a**(0.2) – **1b**(0.8) contains C1=0.2×1.00+0.8×0.039=0.2312 part D_0 compound and C2=0.2×0.00+0.8×0.961=0.7688 part D_1 compound, whose theoretical spectra should be combined with the above obtained coefficients (C1, C2).*

Similarity of mass spectra
Normalized dot product (also known as spectral contrast angle or cosine similarity) is a commonly used function to measure the similarity between two mass spectra. Normalized dot product (NDP) for mass spectra ***A*** and ***B***, consisting of ***k*** peaks is given as following:

$$NDP_{A,B} = \frac{\sum_{i=1}^{k} I_{A,i} \cdot I_{B,i}}{\sqrt{\sum_{i=1}^{k} I_{A,i}^2 \cdot \sum_{i=1}^{k} I_{B,i}^2}}$$

Where:
$I_{A,i}$ describes the relative intensity of the ith peak in spectrum of ***A***
$I_{B,i}$ describes the relative intensity of the ith peak in spectrum of ***B***

NDP values range from 0-1, where the higher score corresponds to higher similarity.

Distribution of similarities within a mixing system
The software performs pairwise comparison of theoretical mass spectra *via* NDP function across all the mixtures within a mixing system. The number of pairs having NDP values in certain ranges (bins) is recorded to create an NDP distribution, which is a useful descriptor of coding quality. The software also reports on the pairs with NDP values over a user defined threshold level as an output to allow further analysis. Pair(s) with the highest NDP value within a mixing system is/are also reported along with the corresponding NDPs.

Spectrum searching
The software allows for spectrum similarity searching within the library of theoretical spectra in a mixing system, based on pairwise comparison *via* NDP function. The best 5 hits with the respective NDP values are reported as an output.

3 General information

All reagents obtained from commercial sources were used without further purification. Anhydrous solvents were obtained from commercial sources and used without further drying. Nitrogen gas dried on a column of Drierite® was used as inert atmosphere. In hydrogenation reactions H_2/D_2 pressure was provided with a balloon. D_2 gas was obtained directly from a D_2 cylinder (D_2 content: 99.96%). The reactions were monitored using LC-MS and GC-MS instruments. Analytical LC-MS: Agilent HP1200 LC with Agilent 6140 quadrupole MS, operating in positive or negative ion electrospray ionisation mode. Molecular weight scan range was 100 to 1350 m/z. Parallel UV detection was done at 210 nm and 254 nm. Samples were supplied as a 1 mM solution in MeCN with 2 µL loop injection, unless stated otherwise. LC-MS analyses were performed on two instruments, one of which was operated with basic, and the other with acidic eluents. Basic LC-MS: Gemini-NX, 3 µm, C18, 50 mm × 3.00 mm i.d. column at 23°C, at a flow rate of 1 mL min^{-1} using 5 mM aq. NH_4HCO_3 solution and MeCN as eluents. Acidic LC-MS: ZORBAX Eclipse XDB-C18, 1.8 µm, 50 mm × 4.6 mm i.d. column at 40°C, at a flow rate of 1 mL min^{-1} using water and MeCN as eluents, both containing 0.07 V/V% TFA. Combination gas chromatography and low-resolution mass spectrometry were performed on Agilent 6850 gas chromatograph and Agilent 5975C mass spectrometer using 15 m × 0.25 mm column with 0.25 µm HP-5MS coating and helium as carrier gas. Ion source: EI^+, 70 eV, 230°C, quadrupole: 150°C, interface: 300°C. Flash chromatography was performed on ISCO CombiFlash Rf 200i or ISCO CombiFlash Torrent® with pre-packed silica-gel cartridges (Redi*Sep*®*R*$_f$ Gold High Performance). Preparative HPLC purifications were performed on an ISCO CombiFlash EZ Prep system with a Gemini-NX® 10 µm C18, 250 mm × 50 mm column running at a flow rate of 118 mL min^{-1} with UV diode array detection. ^1H NMR, and proton-decoupled ^{13}C NMR measurements were performed on Bruker Avance III 500 MHz spectrometer and Bruker Avance III 400 MHz spectrometer, using DMSO-d_6 as solvent. ^1H and ^{13}C NMR data are in the form of delta values, given in part per million (ppm), using the residual peak of the solvent as internal standard (DMSO-d_6: 2.50 ppm (^1H) / 39.5 ppm (^{13}C)). Splitting patterns are designated as: s (singlet), d (doublet), t (triplet), m (multiplet), br s (broad singlet), dd (doublet of doublets), dt (doublet of triplets). Solvent peaks are marked with a " ▼ " sign on the spectra, type of solvent is indicated by "★" if other than water or the deuterated solvent on the spectra. For all intermediates, the ^{13}C NMR spectrum is only given for the nondeuterated molecules as no significant change in the chemical shift can be observed when deuterated analogues are measured, the presence of deuterium atoms causes only the disappearance of corresponding peaks. For all final compounds (**1a-y**), both ^1H and ^{13}C NMR spectra are given. In some cases, due to tautomers or amide rotamers two sets of signals appear in the spectra. LC-HRMS were determined on an Agilent 1290 Infinity II - Agilent 6545 LC-QTOF, ion source temperature 200°C, ESI +/-, ionization voltage: +/-4.5 kV. InfinityLab Poroshell 120 SB-C18, 2.1 mm, 1.9 µm column. Mass resolution: min. 10000. GC-HRMS were determined on Agilent 7890B gas chromatograph and AccuTOF GCX mass spectrometer using 15 m × 0.25 mm column with 0.25 µm HP-5MS coating and helium as carrier gas. Ion source: FI, 37V, interface: 320°C. Approximatively 0.01 mg mL^{-1} stock solutions of **1a-y** were prepared in propionitrile with an accuracy of 1% in 100 mL measuring flasks. Mixtures were prepared by measuring the individual components into a vial using Sartorius Tacta mechanical 1-channel pipettes and the volume was adjusted to 1 mL using acetonitrile. Chemical names were generated by BioviaDraw 2021.

4 Experimental data

4.1 General procedures

4.1.1 General procedure 1: Hydroxy-bromo exchange

A pear-shaped flask equipped with a magnetic stirring bar was filled with **4-hydroxy-quinoline derivative** (1.0 equiv.) and was dissolved in 1,2-dichloroethane (5.00 mL/mmol), then phosphoryl bromide (2.00 equiv.) was added in one portion. The reaction mixture was stirred at 80°C until complete conversion was observed (usually 50 – 120 minutes). The reaction mixture was diluted with DCM, water was added, and the pH was set to neutral with 25% aq. NaOH solution, then the mixture was filtered through a pad of celite. The organic phase was separated from the filtrate and washed with water, dried over Na_2SO_4, filtered and the filtrate was concentrated *in vacuo*. Then the crude product was purified via flash chromatography using heptane and DCM as eluents.

4.1.2 General procedure 2A: Sonogashira coupling with methyl propargyl ether

A pear-shaped flask equipped with a magnetic stirring bar was filled with **4,6-dibromo-quinoline derivative** (1.0 equiv.) and copper(I) iodide (0.2 equiv.). The flask was evacuated then back-filled with N_2 (repeated 3 times), then dry, degassed 1,4-dioxane (7.5 mL/mmol), 3-methoxyprop-1-yne (3.0 equiv.) and *N*-isopropylpropan-2-amine (15.0 equiv.) were added at room temperature while stirring. The reaction mixture was heated to 60°C and bis(di-*tert*-butyl(4-dimethylaminophenyl) phosphine) dichloropalladium(II) (0.05 equiv.) was added at this temperature. Then it was stirred at 60°C until complete conversion was observed (usually 0.5 – 3 hours). Then the mixture was diluted with DCM, washed twice with 5% aq. citric acid solution and once with sat. aq. $NaHCO_3$ solution. The combined aqueous phase was back-extracted twice with DCM. The combined organic phase was dried over Na_2SO_4, filtered and the filtrate was concentrated *in vacuo*. Then the crude product was purified via flash chromatography using heptane, DCM and MeOH as eluents.

4.1.3 General procedure 2B: Sonogashira coupling with silyl protected alkynes

A pear-shaped flask equipped with a magnetic stirring bar was filled with the **acetylene derivative** (6.0 equiv.), 2-methyltetrahydrofuran (7.5 mL/mmol) and tetrabutylammonium fluoride trihydrate (6.0 equiv.) and the reaction mixture was stirred at room temperature until complete conversion was observed (usually 18 hours). Then sat. aq. NH_4Cl solution was added, and the phases were separated. The organic phase was washed twice with sat. aq. NH_4Cl solution, then the combined aqueous phase was back-extracted twice with 2-methyltetrahydrofuran. The combined organic phase was dried over Na_2SO_4 and filtered. The filtrate was transferred into a pear-shaped flask, then **4,6-dibromo-quinoline derivative** (1.0 equiv.), copper(I) iodide (0.2 equiv.), bis(di-*tert*-butyl(4-dimethylaminophenyl) phosphine) dichloropalladium(II) (0.05 equiv.), and *N*-isopropylpropan-2-amine (15.0 equiv.) were added. The flask was evacuated then backfilled with N_2 (repeated 3 times), then the reaction mixture was heated to 60°C and stirred at this temperature until complete conversion was observed (usually 1 hour). Then EtOAc was added, and the resulting solution was washed twice with 5% aq. citric acid solution and once with sat. aq. $NaHCO_3$ solution. The combined aqueous phase was back-extracted twice with EtOAc. The combined organic phase was dried over Na_2SO_4, filtered and the filtrate was concentrated *in vacuo*. Then the crude product was purified via flash chromatography using heptane, DCM and MeOH as eluents.

4.1.4 General procedure 3: NO_2 to NH_2 group reduction

A pear-shaped flask equipped with a magnetic stirring bar was filled with **8-nitro-quinoline** derivative (1.0 equiv.), iron (10.0 equiv.), acetic acid (10.0 equiv.) and ethanol (40 mL/mmol, 70% aq. solution). The reaction mixture was stirred at 50°C until complete conversion was observed (usually 2 hours). Then it was cooled to room temperature and the pH was set to 8 with 25 m/m% aq. ammonia solution. The insoluble material was removed by filtration through a pad of celite, then the filtrate was extracted twice with DCM. The organic phase was dried over Na_2SO_4, filtered and the filtrate was concentrated *in vacuo*. Then the crude product was purified via flash chromatography using heptane, DCM and MeOH as eluents.

4.1.5 General procedure 4A: Hydrogenation reaction

A pear-shaped flask equipped with a stirring bar was filled with **alkynyl quinoline** derivative (1.0 equiv.) and 10 m/m% Pd/C (0.1 g/g quinoline derivative). The flask was evacuated then back-filled with N_2 (repeated 3 times), then DCM (50 mL/g), MeOH (50 mL/g) and *N*,*N*-diethylethanamine (10 mL/g) were added. The headspace of the vial was filled with N_2 and evacuated (repeated twice), then filled with hydrogen gas (1 atm) and the reaction mixture was stirred at room temperature until complete conversion was observed (usually 3 – 18 hours). Then it was filtered through a pad of celite and the filtrate was concentrated *in vacuo*. Then the crude product was purified via flash chromatography using heptane, DCM and MeOH as eluents.

4.1.6 General procedure 4B: Deuteration reaction

A pear-shaped flask equipped with a magnetic stirring bar was filled with **alkynyl quinoline** derivative (1.0 equiv.) and 10 m/m% Pd/C (0.1 g/g quinoline derivative). Then the flask was evacuated then backfilled with N_2 (repeated 3 times), then MeOD (25 mL/g) was added. The mixture was sonicated in an ultrasonic bath for 15 minutes, then it was concentrated *in vacuo*. The MeOD addition, sonication and concentration process was repeated 5 times. Then MeOD (50 mL/g quinoline derivative) was added. The headspace of the flask was filled with N_2 and evacuated (repeated twice), then filled with deuterium gas (1 atm) and the reaction mixture was stirred at room temperature until complete conversion was observed (usually 2 – 18 hours). Then it was filtered through a pad of celite and the filtrate was concentrated *in vacuo*. Then the crude product was purified via flash chromatography using heptane, DCM and MeOH as eluents.

4.1.7 General procedure 5A: Acylation reaction

A pear-shaped flask equipped with a magnetic stirring bar was filled with **8-amino-quinoline** derivative (1.0 equiv.), DCM (30 mL/mmol) and *N*,*N*-diethylethanamine (5 equiv.). The mixture was cooled to 0°C and acetyl chloride (3 equiv.) was added, then the mixture was allowed to warm to room temperature and stirred until complete conversion was observed (usually 1 hour). Then it was washed with water, 1 M aq. HCl solution, sat. aq. $NaHCO_3$ solution, then dried over Na_2SO_4, filtered and the filtrate was concentrated *in vacuo*. Then the crude product was purified via flash chromatography using heptane, DCM and MeOH as eluents.

4.1.8 General procedure 5B: Deuteroacylation reaction

A pear-shaped flask equipped with a magnetic stirring bar was filled with **8-amino-quinoline** derivative (1.0 equiv.), *N*,*N*-dimethylpyridin-4-amine (0.2 equiv.), pyridine (10 equiv.), and EtOAc (20 mL/mmol). Then (2,2,2-trideuterioacetyl) 2,2,2-trideuterioacetate (3 equiv.) was added and the mixture was stirred at 50°C until complete conversion was observed

(usually 18 hours). Then it was washed with water, 1 M aq. citric acid solution, sat. aq. $NaHCO_3$ solution, dried over Na_2SO_4, filtered and the filtrate was concentrated *in vacuo*. Then the crude product was purified via flash chromatography using DCM and MeOH as eluents.

4.1.9 General procedure 6: Methyl ester hydrolysis

A pear-shaped flask equipped with a magnetic stirring bar was filled with **methyl quinoline-2-carboxylate** derivative (1.0 equiv.) and was dissolved in 1,4-dioxane (100 mL/g). A solution of lithium hydroxide monohydrate (10 equiv.) in water (50 mL/g) was added and the reaction mixture was stirred at room temperature until complete conversion was ob served (usually 1 hour). Then the excess lithium hydroxide was quenched by 1 M aq. HCl solution. Then the reaction mixture was diluted with DCM. The pH of the aqueous phase was set to ~5 with 1 M aq. citric acid solution, then the organic phase was separated and washed with 1 M aq. citric acid solution and brine. Then it was dried over Na_2SO_4, filtered and the filtrate was concentrated *in vacuo*. Then the crude product was purified via flash chromatography using heptane, DCM and MeOH as eluents. Then the product was further purified by RP-HPLC using 1% aq. HCOOH solution and MeCN as eluents.

4.2 Synthetic procedure and analytical data of the newly synthesized compounds in this work

***tert*-butyl-diphenyl-(3-tetrahydropyran-2-yloxyprop-1-ynyl)silane (15)**

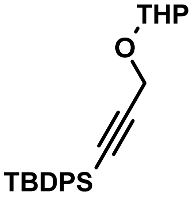

In a 1000 mL pear-shaped flask equipped with a magnetic stirring bar and a thermometer, 25.00 mL **2-prop-2-ynoxytetrahydropyran** (1.0 equiv., 177.8 mmol.) was dissolved in 178 mL dry, degassed tetrahydrofuran (1 mL/mmol 2-prop-2-ynoxytetrahydropyran) and the mixture was cooled to –78°C under N_2 atmosphere. A solution of 75 mL *n*-butyllithium (1.05 equiv., 186.7 mmol, 2.5 mol/L in hexanes) was added to the mixture dropwise, and the mixture was warmed to room temperature. Then it was stirred for 90 minutes and was cooled to –30°C again. Then 47.74 mL ***tert*-butyl-chloro-diphenyl-silane** (1.05 equiv., 186.7 mmol) was added to the mixture dropwise and was warmed to room temperature and stirred overnight. Then the mixture was quenched by 50 mL 1 M aq. HCl solution, extracted with 150 mL EtOAc three times. The combined organic phase was washed with 50 mL brine, dried over Na_2SO_4, filtered and the filtrate was concentrated *in vacuo*. Then the crude product was purified via flash chromatography using heptane and DCM as eluents to afford 51.28 g ***tert*-butyl-diphenyl-(3-tetrahydropyran-2-yloxyprop-1-ynyl)silane (8)** (135.5 mmol, 76%) as a colourless oil.

^{1}H NMR (400 MHz, [D$_6$]DMSO): δ 7.77-7.70 (m, 4H, Ar-H), 7.49-7.40 (m, 6H, Ar-H), 4.88 (t, *J* = 3.0 Hz, 1H, CH), 4.44 (dt, *J* = 16.6 Hz, *J* = 10.2 Hz, 2H, CH_2), 3.82-3.74 (m, 1H, CH_2), 3.50-3.41 (m, 2H, CH_2), 1.79-1.62 (m, 2H, CH_2), 1.57-1.40 (m, 4H, CH_2), 1.02 (s, 9H, CH_3). ^{13}C NMR (100 MHz, [D$_6$]DMSO): δ 135.1, 132.3, 129.9, 128.1, 107.2, 96.4, 85.1, 61.5, 54.4, 29.9, 26.8, 24.9, 18.9, 18.0. HRMS (GC-FI): *m/z* [M$^+$] calcd for $C_{24}H_{30}O_2Si$: 378.2015; found: 378.2011.

3-[*tert*-butyl(diphenyl)silyl]prop-2-yn-1-ol (8)

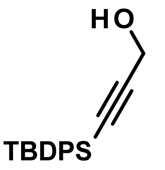

To a 1000 mL pear-shaped flask equipped with a magnetic stirring bar 50.00 g ***tert*-butyl-diphenyl-(3-tetrahydropyran-2-yloxyprop-1-ynyl)silane** (1.0 equiv., 132.1 mmol), 264 mL methanol (2 mL/mmol *tert*-butyl-diphenyl-(3-tetrahydropyran-2-yloxyprop-1-ynyl)silane) and 227 mg 4-methylbenzenesulfonic acid (0.01 equiv., 1.321 mmol) were added and the mixture was stirred at room temperature for 1 hour. Then the reaction mixture was diluted with 200 mL EtOAc, washed with brine, dried over Na_2SO_4, filtered and the filtrate was concentrated *in vacuo*. Then the crude product was purified via flash chromatography using heptane and EtOAc as eluents to afford 33.72 g **3-[*tert*-butyl(diphenyl)silyl]prop-2-yn-1-ol** (91% purity, 104.2 mmol, 79%) as a white solid.

^{1}H NMR (400 MHz, [D$_6$]DMSO): δ 7.80-7.71 (m, 4H, Ar-H), 7.49-7.38 (m, 6H, Ar-H), 5.44 (t, *J* = 6.0 Hz, 1H, OH), 4.27 (d, *J* = 6.0 Hz, 2H, CH$_2$), 1.02 (s, 9H, CH$_3$). ^{13}C NMR (100 MHz, [D$_6$]DMSO): δ 135.2, 132.6, 129.8, 128.0, 111.2, 82.4, 49.7, 26.8, 18.0. HRMS (GC-FI): *m/z* [M+H]$^+$ calcd for $C_{19}H_{22}OSi$: 294.1440; found: 294.1444.

***tert*-butyl-(3-methoxyprop-1-ynyl)-diphenyl-silane (16)**

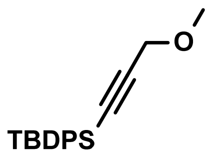

A 100 mL pear-shaped flask equipped with a magnetic stirring bar was filled with 3.846 g **3-[*tert*-butyl(diphenyl)silyl]prop-2-yn-1-ol** (91% purity, 1.0 equiv., 6.180 mmol), dissolved in 15.5 mL dry, degassed tetrahydrofuran (2.5 mL/mmol 3-[*tert*-butyl(diphenyl)silyl]prop-2-yn-1-ol) and 0.462 mL iodomethane (1.2 equiv., 7.716 mmol), then 309 mg sodium hydride (60% dispersion in mineral oil,1.25 equiv., 7.73 mmol) was added in portions. The reaction mixture was stirred at room temperature for 1 hour. Then the excess sodium hydride was quenched with 5 mL 1 M HCl, then 50 mL EtOAc was added and the phases were separated. Then the organic phase was washed with 25 mL brine solution, dried over Na_2SO_4, filtered and the filtrate was concentrated *in vacuo*. Then the crude product was purified via flash chromatography using DCM and MeOH as eluents to afford 1.756 g ***tert*-butyl-(3-methoxyprop-1-ynyl)-diphenyl-silane** (5.692 mmol, 92%) as a colourless oil.

^{1}H NMR (400 MHz, [D$_6$]DMSO): δ 7.78-7.73 (m, 4H, Ar-H), 7.49-7.39 (m, 6H, Ar-H), 4.31 (s, 2H, CH$_2$), 3.37 (s, 3H, OCH$_3$), 1.03 (s, 9H, CH$_3$). ^{13}C NMR (100 MHz, [D$_6$]DMSO): δ 135.1, 132.3, 129.9, 128.0, 107.0, 85.5, 59.7, 57.0, 26.8, 18.0. HRMS (ESI): *m/z* [M+NH$_4$]$^+$ calcd for $C_{20}H_{24}OSi$: 326.1935; found: 326.1927.

***tert*-butyl-diphenyl-[3-(trideuteriomethoxy)prop-1-ynyl]silane (17)**

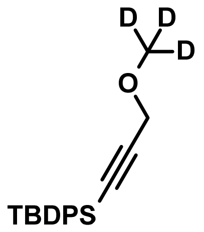

A 500 mL pear-shaped flask equipped with a magnetic stirring bar was filled with 21.98 g **3-[*tert*-butyl(diphenyl)silyl]prop-2-yn-1-ol** (91% purity, 67.91 mmol, 1 equiv.), dissolved in 170 mL dry, degassed tetrahydrofuran (2.5 mL/mmol 3-[*tert*-butyl(diphenyl)silyl]prop-2-yn-1-ol) and 5.072 mL trideuterio(iodo)methane (81.49 mmol, 1.2 equiv.), then 3.395 g sodium hydride (60% dispersion in mineral oil, 84.89 mmol, 1.25 equiv.) was added in portions. The reaction mixture was stirred at room temperature for 1 hour. Then the excess sodium hydride was quenched with 50 mL 1 M HCl, then 500 ml EtOAc was added. Then the phases were separated, and the organic phase was washed with 150 mL brine, dried over Na_2SO_4, filtered and the filtrate was concentrated *in vacuo*. Then the crude product was purified via flash chromatography using heptane and EtOAc as eluents to afford 19.24 g ***tert*-butyl-diphenyl-[3-(trideuteriomethoxy)prop-1-ynyl]silane** (61.75 mmol, 91%) as a colourless oil.

^{1}H NMR (400 MHz, [D$_6$]DMSO): δ 7.79-7.72 (m, 4H, Ar-H), 7.49-7.39 (m, 6H, Ar-H), 4.31 (s, 2H, CH$_2$), 1.03 (s, 9H, CH$_3$). ^{13}C NMR (100 MHz, [D$_6$]DMSO): δ 135.1, 132.3, 129.9, 128.0, 107.1, 85.4, 59.6, 26.8, 18.0. HRMS (ESI): *m/z* [M+NH$_4$]$^+$ calcd for $C_{20}D_3H_{21}OSi$: 329.2123; found: 329.2116.

3-[*tert*-butyl(diphenyl)silyl]prop-2-ynoic acid (18)

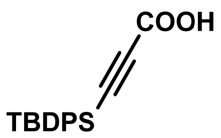

In a 1000 mL pear-shaped flask 20.00 g **3-[*tert*-butyl(diphenyl)silyl]prop-2-yn-1-ol** (90% purity, 1.0 equiv., 61.12 mmol) was dissolved in 611 mL acetone (10 mL/mmol **3-[*tert*-butyl(diphenyl)silyl]prop-2-yn-1-ol**). Then the reaction mixture was cooled to 0°C and 73.2 mL trioxochromium, sulfuric acid (1:1) (1.67 M, 2 equiv., 122.2 mmol, freshly prepared: 12.5 g CrO_3 was dissolved in a mixture of 13 mL of cc. H_2SO_4 and 62 mL water) was added dropwise to the solution. A green precipitate was formed. The reaction mixture was allowed to warm to room temperature and stirred at this temperature for 1 hour. Then the reaction mixture was concentrated *in vacuo*, the residue was extracted with 3 × 100 mL EtOAc, the combined organic phase was washed with water and brine, dried over Na_2SO_4, filtered and the filtrate was concentrated *in vacuo*. Then the crude product was purified via flash chromatography using heptane and EtOAc as eluents to afford 13.77 g **3-[*tert*-butyl(diphenyl)silyl]prop-2-ynoic acid** (44.64 mmol, 73%) as an off-white solid.

^{1}H NMR (500 MHz, [D$_6$]DMSO): δ 14.20 (br s, 1H, COOH), 7.77-7.69 (m, 4H, Ar-H), 7.53-7.44 (m, 6H, Ar-H), 1.05 (s, 9H, CH_3). ^{13}C NMR (125 MHz, [D$_6$]DMSO): δ 153.4, 135.1, 130.8, 130.4, 128.3, 100.2, 86.2, 59.8, 26.6, 20.8, 18.1. HRMS (ESI): *m/z* [M-H]$^-$ calcd for $C_{19}H_{19}O_2Si$: 307.1160; found: 307.1159.

methyl 3-[*tert*-butyl(diphenyl)silyl]prop-2-ynoate (19)

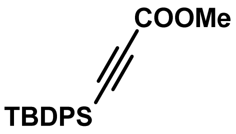

A 1000 mL pear-shaped flask equipped with magnetic stirring bar was filled with 12.00 g **3-[*tert*-butyl(diphenyl)silyl]prop-2-ynoic acid** (1.0 equiv., 38.91 mmol) dissolved in 389 mL dichloromethane (10 mL/mmol 3-[*tert*-butyl(diphenyl)silyl]prop-2-ynoic acid) and methanol (10 mL/mmol 3-[*tert*-butyl(diphenyl)silyl]prop-2-ynoic acid). Without closing the flask, 39 mL **diazomethyl(trimethyl)silane** (2.0 M in hexanes, 2.0 equiv., 77.8 mmol) was added slowly, while the flask was cooled in an ice bath. Then the reaction mixture was stirred at this temperature for 1 hour. Then the excess diazomethyl(trimethyl)silane was quenched by adding glacial acetic acid until the gas evolution stopped. Then the reaction mixture was concentrated *in vacuo*. Then the crude product was purified via flash chromatography using heptane, DCM and MeOH as eluents to afford 11.79 g **methyl 3-[*tert*-butyl(diphenyl)silyl]prop-2-ynoate** (36.55 mmol, 94%) as a white, crystalline solid.

^{1}H NMR (500 MHz, [D$_6$]DMSO): δ 7.74-7.69 (m, 4H, Ar-H), 7.54-7.44 (m, 6H, Ar-H), 3.79 (s, 3H, C(O)OCH$_3$), 1.05 (s, 9H, CH_3). ^{13}C NMR (125 MHz, [D$_6$]DMSO): δ 152.4, 135.1, 130.5, 130.4, 128.4, 98.4, 88.4, 53.3, 26.6, 18.1. HRMS (GC-FI): *m/z* [M$^+$] calcd for $C_{20}H_{22}O_2Si$: 322.1389; found: 322.1387.

3-[*tert*-butyl(diphenyl)silyl]-1,1-dideuterio-prop-2-yn-1-ol (9)

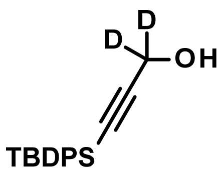

To a 1000 mL pear-shaped flask equipped with a magnetic stirring bar 10.00 g **methyl 3-[*tert*-butyl(diphenyl)silyl]prop-2-ynoate** (1.0 equiv., 31.00 mmol) and 620 mL dry, degassed THF (20 ml/mmol methyl 3-[*tert*-butyl(diphenyl)silyl]prop-2-ynoate) were measured, then the mixture was cooled to –78°C. At this temperature 13.40 g lithium aluminum deuteride (1.2 equiv., 37.20 mmol) was added to the mixture in portions during a 10-minute period, then the reaction mixture was stirred at –78°C for 10 minutes (Low temperature and proper dilution is necessary to avoid by-product formation). Then the reaction mixture was warmed to –30°C and 14 mL water was added slowly, then 14 mL 15% aq. NaOH solution and 42 mL water were added. The reaction mixture was warmed to room temperature, $MgSO_4$ was added and stirred for 30 minutes, then filtered and the filtrate was concentrated *in vacuo*. Then the crude product was purified via flash chromatography using heptane and EtOAc as eluents to afford 6.872 g **3-[*tert*-butyl(diphenyl)silyl]-1,1-dideuterio-**

prop-2-yn-1-ol (23.17 mmol, 75%) as a colourless oil.
^{1}H NMR (500 MHz, [D$_6$]DMSO): δ 7.77-7.73 (m, 4H, Ar-H), 7.46-7.41 (m, 6H, Ar-H), 5.39 (s, 1H, OH), 1.02 (s, 9H, CH_3). ^{13}C NMR (125 MHz, [D$_6$]DMSO): δ 135.1, 132.6, 129.8, 127.9, 111.2, 82.4, 26.8, 18.0. HRMS (GC-FI): *m/z* [M+] calcd for $C_{19}D_2H_{20}OSi$: 296.1565; found: 296.1565.

***tert*-butyl-[3,3-dideuterio-3-(trideuteriomethoxy)prop-1-ynyl]-diphenyl-silane (20)**

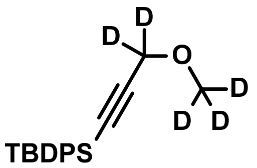

A 250 mL pear-shaped flask equipped with a magnetic stirring bar was filled with 6.000 g **3-[*tert*-butyl(diphenyl)silyl]-1,1-dideuterio-prop-2-yn-1-ol** (1.0 equiv., 20.24 mmol), dissolved in 51 mL dry, degassed tetrahydrofuran (2.5 mL/mmol 3-[*tert*-butyl(diphenyl)silyl]-1,1-dideuterio-prop-2-yn-1-ol) and 1.51 mL **trideuterio(iodo)methane** (1.2 equiv., 24.29 mmol), then 1.012 g sodium hydride (60% dispersion in mineral oil, 1.25 equiv., 25.30 mmol) was added in portions. The reaction mixture was stirred at room temperature for 1 hour until. Then the excess sodium hydride was quenched with 10 mL 1 M HCl, then 100 mL EtOAc was added and the phases were separated. Then the organic phase was washed with 25 mL brine, dried over Na_2SO_4, filtered and the filtrate was concentrated *in vacuo*. Then the crude product was purified via flash chromatography using heptane and EtOAc as eluents to afford 4.650 g ***tert*-butyl-[3,3-dideuterio-3-(trideuteriomethoxy)prop-1-ynyl]-diphenyl-silane** (14.54 mmol, 72%) as a colourless oil.
^{1}H NMR (400 MHz, [D$_6$]DMSO): δ 7.78-7.73 (m, 4H, Ar-H), 7.49-7.42 (m, 6H, Ar-H), 1.03 (s, 9H, CH_3). ^{13}C NMR (100 MHz, [D$_6$]DMSO): δ 135.1, 132.3, 129.9, 128.0, 85.4, 26.8, 18.0. HRMS (ESI): *m/z* [M+NH$_4$]$^+$ calcd for $C_{20}D_5H_{19}OSi$: 331.2254; found: 331.2250.

methyl 4,6-dibromo-8-nitro-quinoline-2-carboxylate (4a)

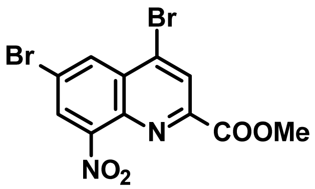

Using **General Procedure 1** and 20.75 g **methyl 6-bromo-4-hydroxy-8-nitro-quinoline-2-carboxylate** (83% purity,1.0 equiv., 63.44 mmol prepared as described by Zwillinger *et al.*[1]), 16.42 g **methyl 4,6-dibromo-8-nitro-quinoline-2-carboxylate** was obtained as a pale yellow solid (42.10 mmol, 66%).
^{1}H NMR (500 MHz, [D$_6$]DMSO): δ 8.84 (d, *J*= 1.9 Hz, 1H, Ar-H), 8.58 (d, *J*= 1.9 Hz, 1H, Ar-H), 8.56 (s, 1H, Ar-H), 3.96 (s, 3H, CH_3). ^{13}C NMR (125 MHz, [D$_6$]DMSO): δ 163.3, 149.3, 148.9, 137.2, 134.0, 131.8, 129.8, 128.1, 127.3, 122.3, 53.3. HRMS (ESI): *m/z* [M+H]$^+$ calcd for $C_{11}H_6Br_2N_2O_4$: 388.8767; found: 388.8760.

1 M. Zwillinger, P. S. Reddy, B. Wicher, P. K. Mandal, M. Csékei, L. Fisher, A. Kotschy, I. Huc, *Chem. Eur. J.* 2020, **26**, *72*, 17366-17370.

dimethyl (*Z*)-2-(4-bromo-2-nitro-anilino)-3-deuterio-but-2-enedioate (2b)

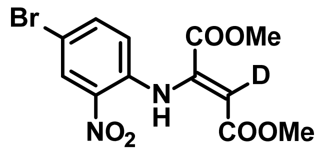

A 500 mL, pear-shaped flask equipped with a magnetic stirring bar was filled with 25.00 g **4-bromo-2-nitro-aniline** (1.0 equiv., 115.2 mmol),115 mL dry, degassed DCM and 25 mL D_2O. The mixture was stirred vigorously for 10 minutes. The organic layer was separated and stirred for 10 minutes with a fresh portion of 25 mL D_2O. The organic phase was separated, dried over Na_2SO_4, filtered and the filtrate was concentrated *in vacuo.* The content of the flask was transferred into a 250 mL three-necked round-bottom flask and 46.8 mL dry, degassed MeOD was added. The flask was fitted with a reflux condenser and a dropping funnel containing 21.2 mL **dimethyl but-2-ynedioate** (1.5 equiv., 172 mmol). The mixture was heated to 60°C and the dimethyl but-2-ynedioate was added slowly. After 48 hours of stirring 70% conversion was reached (no further conversion could be reached by longer reaction times). Then the mixture was cooled to 0°C while stirring vigorously. The formed crystals were filtered under dry N_2 atmosphere on a G3 frit and washed twice with 20 mL ice-cold MeOD, then dried *in vacuo* to yield 25.83 g **dimethyl (*Z*)-2-(4-bromo-2-nitro-anilino)-3-deuterio-but-2-enedioate** (71.95 mmol, 62%) as yellow crystals.

^{1}H NMR (500 MHz, [D$_6$]DMSO): δ 10.81 (s, 1H, NH), 8.30 (d, *J*= 2.4 Hz, 1H, Ar-H), 7.82 (dd, *J* = 8.8 Hz, *J*= 2.3 Hz, 1H, Ar-H), 6.89 (d, *J* = 8.8 Hz, 1H, Ar-H), 3.74 (s, 3H, C(O)OCH$_3$), 3.73 (s, 1H, C(O)OCH$_3$). ^{13}C NMR (125 MHz, [D$_6$]DMSO): δ 167.6, 163.2, 143.1, 138.3, 137.7, 135.2, 128.1, 123.3, 113.6, 102.3, 53.5, 51.9. HRMS (ESI): *m/z* [M+H]$^+$ calcd for $C_{12}DH_{10}BrN_2O_6$: 359.9936, found: 359.9921.

methyl 6-bromo-3-deuterio-4-hydroxy-8-nitro-quinoline-2-carboxylate (3b)

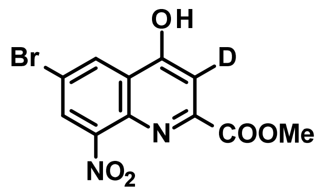

A 500 mL pear-shaped flask equipped with a magnetic stirring bar was filled with dry N_2, 143.9 g **di-phosphorus pentaoxide** (12.2 equiv., 1014 mmol), closed with a rubber septum and 18.34 mL **deuterated water** (12.2 equiv., 1014 mmol) was slowly added while the flask was cooled in an ice bath. After the excessive heat evolution ceased, the mixture was heated to 250°C for 2 hours (until it became homogeneous), then cooled to 150°C. Then 30.00 g **dimethyl (*Z*)-2-(4-bromo-2-nitro-anilino)-3-deuterio-but-2-enedioate** (1.0 equiv., 83.07 mmol) was added while stirring vigorously. The reaction mixture was stirred at 150°C for 5 h. Then the reaction mixture was cooled to room temperature and leached by sonication with 500 mL water. The pH was set to 7–8 by adding 25% aq. NaOH solution while stirring. Ice was added to keep the temperature below 30°C. The crude product was filtered and washed with 50 mL cold water then dried on air to afford 17.01 g **methyl 6-bromo-3-deuterio-4-hydroxy-8-nitro-quinoline-2-carboxylate** (72% purity, 37.33 mmol, 45%) as brown crystals.

^{1}H NMR (500 MHz, [D$_6$]DMSO): δ 8.61 (br s, 1H, OH), 8.56/7.79 (d, *J* = 1.6 Hz, 1H, Ar-H), 7.71/7.47 (d/dd, *J* = 8.7 Hz, *J* = 1.9 Hz, 1H, Ar-H), 3.94/3.93 (s/s, 3H, C(O)OCH$_3$) (Presence of tautomers!). HRMS (ESI): *m/z* [M+H]$^+$ calcd for $C_{11}DH_6BrN_2O_5$: 327.9674; found: 326.9674.

methyl 4,6-dibromo-3-deuterio-8-nitro-quinoline-2-carboxylate (4b)

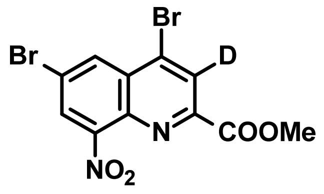

Using **General Procedure 1** and 17.00 g **methyl 6-bromo-3-deuterio-4-hydroxy-8-nitro-quinoline-2-carboxylate** (72% purity, 1.0 equiv., 37.31 mmol), 7.689 g **methyl 4,6-dibromo-3-deuterio-8-nitro-quinoline-2-carboxylate** was obtained as a pale yellow solid (19.67 mmol, 53%).

^{1}H NMR (500 MHz, [D$_6$]DMSO): δ 8.84 (d, *J*= 2.0 Hz, 1H, Ar-H), 8.58 (d, *J* = 2.0 Hz, 1H, Ar-H), 3.96 (s, 3H, C(O)OCH$_3$). HRMS (ESI): *m/z* [M+H]$^+$ calcd for $C_{11}DH_5Br_2N_2O_4$: 389.8830; found: 389.8829.

4-bromo-2,3,5-trideuterio-6-nitro-aniline (5)

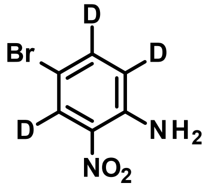

A 1000 mL pear-shaped flask equipped with a magnetic stirring bar was filled with 30.50 g **2,3,4,5-tetradeuterio-6-nitro-aniline** (1.0 equiv., 214.6 mmol, prepared as described by Zwillinger *et al.*[2]) and 215 mL acetic acid (1 mL/mmol **2,3,4,5-tetradeuterio-6-nitro-aniline**), heated to 50°C, then 38.19 g *N*-bromo succinimide (1.00 equiv., 214.6 mmol) was added during a 45 minute period. Then the reaction mixture was heated for further 45 minutes. Then it was poured onto 1000 mL ice-cold water, filtered, and washed with 3×100 mL ice-cold water. The filtered crystals were dried *in vacuo* to give 47.21 g **4-bromo-2,3,5-trideuterio-6-nitro-aniline** (210.4 mmol, 98%) as an orange solid.

^{1}H NMR (500 MHz, [D$_6$]DMSO): δ 7.58 (s, 2H, NH_2). ^{13}C NMR (125 MHz, [D$_6$]DMSO): δ 145.3, 130.5, 104.8. HRMS (EI): *m/z* [M+] calcd for $C_6D_3H_2BrN_2O_2$: 218.9723; found: 218.9718.

dimethyl (*Z*)-2-(4-bromo-2,3,5-trideuterio-6-nitro-anilino)but-2-enedioate (6a)

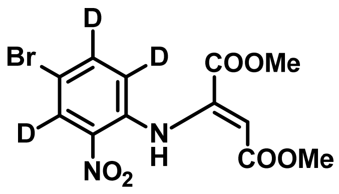

A 500 mL, 3-necked round-bottom flask equipped with a magnetic stirring bar was filled with 35.00 g **4-bromo-2-nitro-aniline** (1.0 equiv., 159.1 mmol) and 79.5 mL dry, degassed MeOH. The flask was fitted with a reflux condenser and a dropping funnel containing 24.4 mL **dimethyl but-2-ynedioate** (1.25 equiv., 199 mmol). The mixture was heated to 60°C and the dimethyl but-2-ynedioate was added slowly. After 8 days of stirring 76% conversion was reached (no further conversion could be reached by longer reaction times). Then the mixture was cooled to 0°C while stirring vigorously. The formed crystals were filtered on a G3 frit and washed twice with 30 mL ice-cold MeOH, then dried *in vacuo* to yield 39.46 g **dimethyl (*Z*)-2-(4-bromo-2,3,5-trideuterio-6-nitro-anilino)but-2-enedioate** (106.8 mmol, 67%) as yellow crystals.

^{1}H NMR (500 MHz, [D$_6$]DMSO): δ 10.82 (s, 1H, NH), 5.84 (s, 1H, CH), 3,74 (s, 3H, C(O)OCH_3), 3.73 (s, 3H, C(O)OCH_3). ^{13}C NMR (125 MHz, [D$_6$]DMSO): δ 167.6, 163.2, 143.2, 138.2, 135.1, 113.4, 102.3, 53.5, 51.9. HRMS (ESI): *m/z* $[M+H]^+$ calcd for $C_{12}D_3H_8BrN_2O_6$: 362.0062, found: 362.0064.

methyl 6-bromo-5,7-dideuterio-4-hydroxy-8-nitro-quinoline-2-carboxylate (7a)

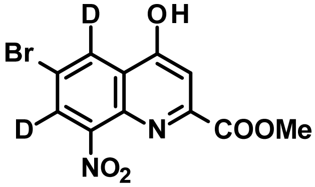

A 500 mL pear-shaped flask equipped with a magnetic stirring bar was filled with dry N_2, 95.63 g di-phosphorus pentaoxide (12.2 equiv., 673.8 mmol), closed with a rubber septum and 12.14 mL water (12.2 equiv., 673.8 mmol) was slowly added while the flask was cooled in an ice bath. After the excessive heat evolution ceased, the mixture was heated to 250°C for 2 hours (until it became homogeneous), then cooled to 150°C. Then 20.00 g **dimethyl (*Z*)-2-(4-bromo-2,3,5-trideuterio-6-nitro-anilino)but-2-enedioate** (1.0 equiv., 55.23 mmol) was added while stirring vigorously. The reaction mixture was stirred at 150°C for 5 hours. Then the reaction mixture was cooled to room temperature and leached by sonication with 500 mL water. The pH was set to 7–8 by adding 25% aq. NaOH solution while stirring. Ice was added to keep the temperature below 30°C. The crude product was filtered and washed with 50 mL cold water then dried on air to afford 13.31 g **methyl 6-bromo-5,7-dideuterio-4-hydroxy-8-nitro-quinoline-2-carboxylate** (81% purity, 32.87 mmol, 59%) as brown crystals.

^{1}H NMR (400 MHz, [D$_6$]DMSO): δ 7.06 (br s, 1H, Ar-H), 3.94 (s, 3H, C(O)OCH_3). HRMS (ESI): *m/z* $[M+H]^+$ calcd for

[2] M. Zwillinger, L. Fischer, G. Sályi, S. Szabó, M. Csékei, I. Huc, A. Kotschy, *J Am Chem Soc* **2022**, *144*, 19078–19088.

$C_{11}D_2H_5BrN_2O_5$: 328.9737; found: 328.9734.

methyl 4,6-dibromo-5,7-dideuterio-8-nitro-quinoline-2-carboxylate (4c)

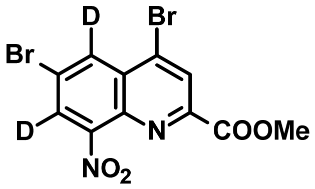

Using **General Procedure 1** and 6.500 g **methyl 6-bromo-5,7-dideuterio-4-hydroxy-8-nitro-quinoline-2-carboxylate** (81% purity, 12.46 mmol, 1.0 equiv.), 2.474 g **methyl 4,6-dibromo-5,7-dideuterio-8-nitro-quinoline-2-carboxylate** was obtained as a pale yellow solid (6.310 mmol, 49%).

^{1}H NMR (500 MHz, [D$_6$]DMSO): δ 8.56 (s, 1H, Ar-H), 3.95 (s, 3H, C(O)OCH$_3$). HRMS (ESI): *m/z* [M+H]$^+$ calcd for $C_{11}D_2H_4Br_2N_2O_4$: 390.8893; found: 390.8894.

dimethyl (*Z*)-2-(4-bromo-2,3,5-trideuterio-6-nitro-anilino)-3-deuterio-but-2-enedioate (6b)

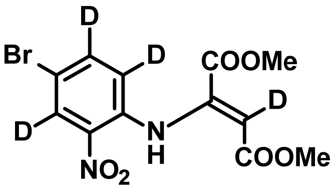

A 500 mL, pear-shaped flask equipped with a magnetic stirring bar was filled with 20.00 g **4-bromo-2,3,5-trideuterio-6-nitro-aniline** (1.0 equiv., 91.34 mmol), 90 mL dry, degassed DCM and 25 mL D_2O. The mixture was stirred vigorously for 10 minutes. The organic layer was separated and stirred for 10 minutes with a fresh portion of 33 mL D_2O. The organic phase was separated, dried over Na_2SO_4, filtered and the filtrate was concentrated *in vacuo*. The content of the flask was transferred into a 250 mL three-necked round-bottom flask and 36.95 mL dry, degassed MeOD was added. The flask was fitted with a reflux condenser and a dropping funnel containing 36.95 mL **dimethyl but-2-ynedioate** (1.5 equiv., 135.9 mmol). The mixture was heated to 60°C and the dimethyl but-2-ynedioate was added slowly. After 48 hours of stirring 80% conversion was reached (no further conversion could be reached by longer reaction times). Then the mixture was cooled to 0°C while stirring vigorously. The formed crystals were filtered under dry N_2 atmosphere on a G3 frit and washed twice with 20 mL ice-cold MeOD, then dried *in vacuo* to yield 24.69 g **dimethyl (*Z*)-2-(4-bromo-2,3,5-trideuterio-6-nitro-anilino)-3-deuterio-but-2-enedioate** (68.19 mmol, 75%) as yellow crystals.

^{1}H NMR (400 MHz, [D$_6$]DMSO): δ 10.82 (s, 1H, NH/ND), 3,74 (s, 3H, CH$_3$), 3.73 (s, 3H, CH$_3$). ^{13}C NMR (100 MHz, [D$_6$]DMSO): δ 167.5, 163.2, 142.9, 138.1, 135.0, 113.4, 53.5, 51.9. HRMS (ESI): *m/z* [M+H]$^+$ calcd for $C_{12}D_4H_7BrN_2O_6$: 363.0124, found: 363.0127.

methyl 6-bromo-3,5,7-trideuterio-4-hydroxy-8-nitro-quinoline-2-carboxylate (7b)

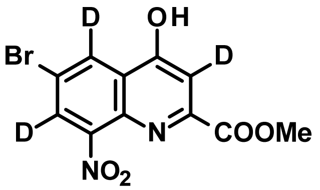

A 250 mL pear-shaped flask equipped with a magnetic stirring bar was filled with dry N_2, 4.026 g **di-phosphorus pentaoxide** (12.2 equiv., 201.0 mmol), closed with a rubber septum and 12.4 mL **deuterated water** (12.2 equiv., 201.0 mmol) was slowly added while the flask was cooled in an ice bath. After the excessive heat evolution ceased, the mixture was heated to 250°C for 2 hours (until it became homogeneous), then cooled to 150°C and 6.000 g **dimethyl (*Z*)-2-(4-bromo-2,3,5-trideuterio-6-nitro-anilino)-3-deuterio-but-2-enedioate** (1.0 equiv, 16.48 mmol) was added while stirring vigorously. The reaction mixture was stirred at 150°C for 5 h. Then the reaction mixture was cooled to room temperature and leached by sonication with 500 mL water. The pH was set to 7-8 by adding 25% aq. NaOH solution while stirring. Ice was added to keep the temperature below 30°C. The crude product was filtered and washed with 50 mL cold water then dried on air to afford 4.722 g **methyl 6-bromo-3,5,7-trideuterio-4-hydroxy-8-nitro-quinoline-2-carboxylate** (79% purity, 11.30 mmol, 69%) as brown crystals.

^{1}H NMR (400 MHz, [D$_6$]DMSO): δ 3.95 (s, 3H, C(O)OCH$_3$). HRMS (ESI): *m/z* [M+H]$^+$ calcd for $C_{11}D_3H_4BrN_2O_5$: 329.9799;

found: 329.9796.

methyl 4,6-dibromo-3,5,7-trideuterio-8-nitro-quinoline-2-carboxylate (4d)

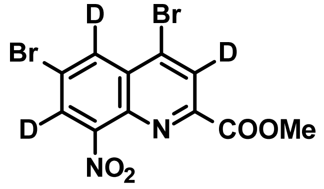

Using **General Procedure 1** and 4.700 g **methyl 6-bromo-3,5,7-trideuterio-4-hydroxy-8-nitro-quinoline-2-carboxylate** (79% purity, 11.28 mmol, 1.0 equiv.), 3.121 g **methyl 4,6-dibromo-3,5,7-trideuterio-8-nitro-quinoline-2-carboxylate** was obtained as pale yellow crystals (7.040 mmol, 70%).

^{1}H NMR (400 MHz, [D$_6$]DMSO): δ 3.96 (s, 3H, C(O)OCH$_3$). HRMS (ESI): *m/z* [M+H]$^+$ calcd for $C_{11}D_3H_3Br_2N_2O_4$: 391.8955; found: 391.8956.

methyl 4,6-bis(3-methoxyprop-1-ynyl)-8-nitro-quinoline-2-carboxylate (11a)

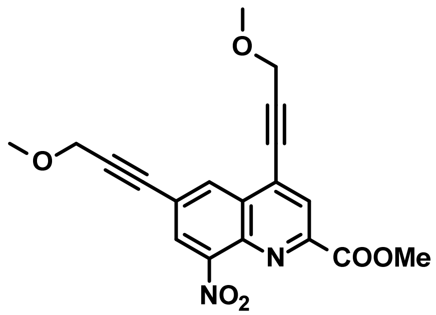

Using **General Procedure 2A** and 2.000 g **methyl 4,6-dibromo-8-nitro-quinoline-2-carboxylate** (5.128 mmol, 1.0 equiv.), 1.571 g **methyl 4,6-bis(3-methoxyprop-1-ynyl)-8-nitro-quinoline-2-carboxylate** was obtained as pale brown crystals (4.266 mmol, 83%).

^{1}H NMR (500 MHz, [D$_6$]DMSO): δ 8.58 (d, *J* = 1.8 Hz, 1H, Ar-H), 8.46 (d, *J* = 1.8 Hz, 1H, Ar-H), 8.29 (s, 1H, Ar-H), 4.59 (s, 2H, CH$_2$), 4.45 (s, 2H, CH$_2$), 3.95 (s, 3H, C(O)OCH$_3$), 3.44 (s, 3H, OCH$_3$), 3.39 (s, 3H, C(O)OCH$_3$). ^{13}C NMR (125 MHz, [D$_6$]DMSO): δ 163.8, 149.6, 148.9, 137.3, 131.3, 130.2, 128.5, 126.7, 125.6, 122.4, 99.6, 91.2, 83.5, 79.9, 59.7, 59.5, 57.5, 57.3, 53.2. HRMS (ESI): *m/z* [M+H]$^+$ calcd for $C_{19}H_{16}N_2O_6$: 369.1081; found: 369.1084.

methyl 3-deuterio-4,6-bis(3-methoxyprop-1-ynyl)-8-nitro-quinoline-2-carboxylate (11b)

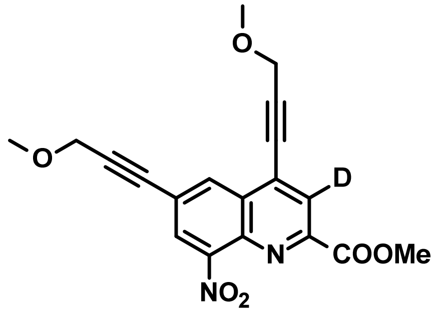

Using **General Procedure 2A** and 4,500 g **methyl 4,6-dibromo-3-deuterio-8-nitro-quinoline-2-carboxylate** (11.51 mmol, 1.0 equiv.), 2.106 g **methyl 3-deuterio-4,6-bis(3-methoxyprop-1-ynyl)-8-nitro-quinoline-2-carboxylate** was obtained as pale brown crystals (5.702 mmol, 50%).

^{1}H NMR (400 MHz, [D$_6$]DMSO): δ 8.60 (d, *J* = 1.8 Hz, 1H, Ar-H), 8.47 (d, *J* = 1.8 Hz, 1H, Ar-H), 4.60 (s, 2H, CH$_2$), 4.45 (s, 2H, CH$_2$), 3.95 (s, 3H, C(O)OCH$_3$), 3.44 (s, 3H, OCH$_3$), 3.39 (s, 3H, OCH$_3$). HRMS (ESI): *m/z* [M+H]$^+$ calcd for $C_{19}DH_{15}N_2O_6$: 370.1144; found: 370.1145

methyl 5,7-dideuterio-4,6-bis(3-methoxyprop-1-ynyl)-8-nitro-quinoline-2-carboxylate (11c)

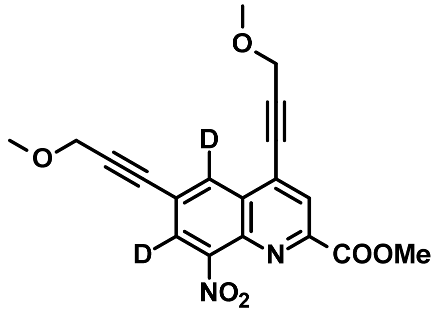

Using **General Procedure 2A** and 1.600 g **methyl 4,6-dibromo-5,7-dideuterio-8-nitro-quinoline-2-carboxylate** (4.082 mmol, 1.0 equiv.), 1.127 g **methyl 5,7-dideuterio-4,6-bis(3-methoxyprop-1-ynyl)-8-nitro-quinoline-2-carboxylate** was obtained as pale brown crystals (3.043 mmol, 74%).

^{1}H NMR (400 MHz, [D$_6$]DMSO): δ 8.29 (s, 1H, Ar-H), 4.59 (s, 2H, CH$_2$), 4.45 (s, 2H, CH$_2$), 3.95 (s, 3H, C(O)OCH$_3$), 3.44 (s, 3H, OCH$_3$), 3.39 (s, 3H, OCH$_3$). HRMS (ESI): *m/z* [M+H]$^+$ calcd for $C_{19}D_2H_{14}N_2O_6$: 371.1207; found: 371.1210.

methyl 3,5,7-trideuterio-4,6-bis(3-methoxyprop-1-ynyl)-8-nitro-quinoline-2-carboxylate (11d)

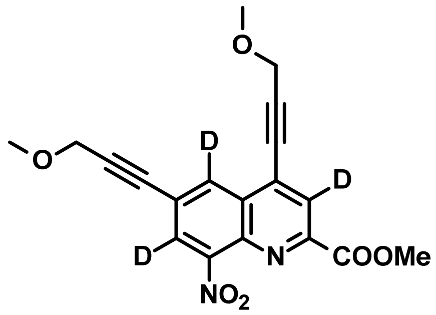

Using **General Procedure 2A** and 900 mg **methyl 4,6-dibromo-3,5,7-trideuterio-8-nitro-quinoline-2-carboxylate** (2.29 mmol, 1.0 equiv.), 402 mg **methyl 3,5,7-trideuterio-4,6-bis(3-methoxyprop-1-ynyl)-8-nitro-quinoline-2-carboxylate** was obtained as pale brown crystals (91% purity, 0.985 mmol, 43%).

^{1}H NMR (500 MHz, [D$_6$]DMSO): δ 4.60 (s, 2H, CH_2), 4.45 (s, 2H, CH_2), 3.96 (s, 3H, C(O)OCH_3), 3.44 (s, 3H, OCH_3), 3.39 (s, 3H, OCH_3). HRMS (ESI): *m/z* $[M+H]^+$ calcd for $C_{19}D_3H_{13}N_2O_6$: 372.1269; found: 372.1273.

methyl 8-nitro-4,6-bis[3-(trideuteriomethoxy)prop-1-ynyl]quinoline-2-carboxylate (11e)

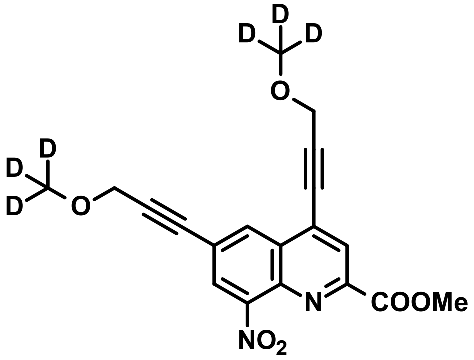

Using **General Procedure 2B** and 4.000 g **methyl 4,6-dibromo-8-nitro-quinoline-2-carboxylate** (10.26 mmol, 1.0 equiv.), and 18.99 g ***tert*-butyl-diphenyl-[3-(trideuteriomethoxy)prop-1-ynyl]silane** (61.54 mmol, 6.0 equiv.), 2.367 g **methyl 8-nitro-4,6-bis[3-(trideuteriomethoxy)prop-1-ynyl]quinoline-2-carboxylate** was obtained as pale brown crystals (92% purity, 5.816 mmol, 57%).

^{1}H NMR (500 MHz, [D$_6$]DMSO): δ 8.58 (d, *J* = 1.8 Hz, 1H, Ar-H), 8.44 (d, *J* = 1.8 Hz, 1H, Ar-H), 8.27 (s, 1H, Ar-H), 4.59 (s, 2H, CH_2), 4.44 (s, 2H, CH_2), 3.95 (s, 3H, C(O)OCH_3). HRMS (ESI): *m/z* $[M+H]^+$ calcd for $C_{19}D_6H_{10}N_2O_6$: 375.1458; found: 375.1460.

methyl 3-deuterio-8-nitro-4,6-bis[3-(trideuteriomethoxy)prop-1-ynyl]quinoline-2-carboxylate (11f)

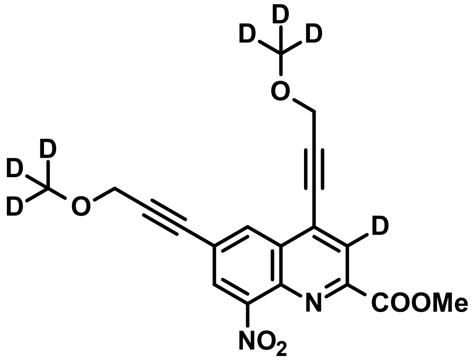

Using **General Procedure 2B** and 2.700 g **methyl 4,6-dibromo-3-deuterio-8-nitro-quinoline-2-carboxylate** (6.906 mmol, 1.0 equiv.) and 12.91 g ***tert*-butyl-diphenyl-[3-(trideuteriomethoxy)prop-1-ynyl]silane** (41.43 mmol, 6.0 equiv.), 2.866 g **methyl 3-deuterio-8-nitro-4,6-bis[3-(trideuteriomethoxy)prop-1-ynyl]quinoline-2-carboxylate** was obtained as pale brown crystals (81% purity, 6.190 mmol, 90%).

^{1}H NMR (400 MHz, [D$_6$]DMSO): δ 8.57 (d, *J* = 1.8 Hz, 1H, Ar-H), 8.44 (d, *J* = 1.8 Hz, 1H, Ar-H), 4.59 (s, 2H, CH_2), 4.44 (s, 2H, CH_2), 3.95 (s, 3H, C(O)OCH_3). HRMS (ESI): *m/z* $[M+H]^+$ calcd for $C_{19}D_7H_9N_2O_6$: 376.1520; found: 376.1521.

methyl 5,7-dideuterio-8-nitro-4,6-bis[3-(trideuteriomethoxy)prop-1-ynyl]quinoline-2-carboxylate (11g)

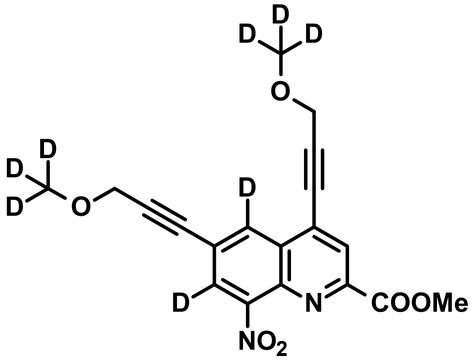

Using **General Procedure 2B** and 2.000 g **methyl 4,6-dibromo-5,7-dideuterio-8-nitro-quinoline-2-carboxylate** (5.102 mmol, 1.0 equiv.) and 9.536 g ***tert*-butyl-diphenyl-[3-(trideuteriomethoxy)prop-1-ynyl]silane** (30.61 mmol, 6.0 equiv.), 1.628 g **methyl 5,7-dideuterio-8-nitro-4,6-bis[3-(trideuteriomethoxy)prop-1-ynyl]quinoline-2-carboxylate** was obtained as pale brown crystals (89% purity, 3.852 mmol, 75%).

^{1}H NMR (400 MHz, [D$_6$]DMSO): δ 8.30 (s, 1H, Ar-H), 4.59 (s, 2H, CH$_2$), 4.45 (s, 2H, CH$_2$), 3.95 (s, 3H, C(O)OCH$_3$). HRMS (ESI): *m/z* [M+H]$^+$ calcd for $C_{19}D_8H_8N_2O_6$: 377.1583; found: 377.1583.

methyl 3,5,7-trideuterio-8-nitro-4,6-bis[3-(trideuteriomethoxy)prop-1-ynyl]quinoline-2-carboxylate (11h)

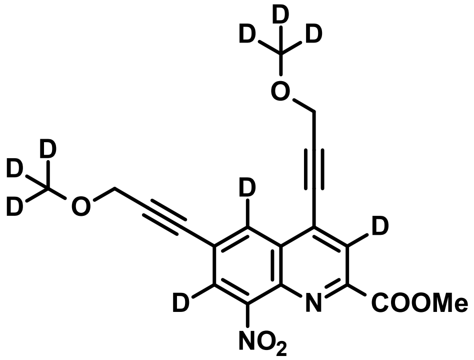

Using **General Procedure 2B** and 3.000 g **methyl 4,6-dibromo-3,5,7-trideuterio-8-nitro-quinoline-2-carboxylate** (7.634 mmol, 1.0 equiv.) and 14.27 g ***tert*-butyl-diphenyl-[3-(trideuteriomethoxy)prop-1-ynyl]silane** (45.80 mmol, 6.0 equiv.), 2.459 g **methyl 3,5,7-trideuterio-8-nitro-4,6-bis[3-(trideuteriomethoxy)prop-1-ynyl]quinoline-2-carboxylate** was obtained as pale brown crystals (94% purity, 5.228 mmol, 80%).

^{1}H NMR (400 MHz, [D$_6$]DMSO): δ 4.59 (s, 2H, CH$_2$), 4.45 (s, 2H, CH$_2$), 3.95 (s, 3H, C(O)OCH$_3$). HRMS (ESI): *m/z* [M+H]$^+$ calcd for $C_{19}D_9H_7N_2O_6$: 378.1646; found: 378.1648.

methyl 4,6-bis[3,3-dideuterio-3-(trideuteriomethoxy)prop-1-ynyl]-8-nitro-quinoline-2-carboxylate (11i)

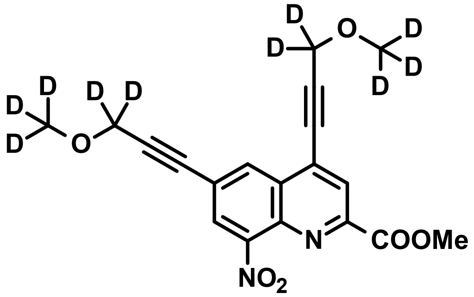

Using **General Procedure 2B** and 250 mg **methyl 4,6-dibromo-8-nitro-quinoline-2-carboxylate** (0.641 mmol, 1.0 equiv.), and 1.206 g ***tert*-butyl-[3,3-dideuterio-3-(trideuteriomethoxy)prop-1-ynyl]-diphenyl-silane** (3.846 mmol, 6.0 equiv.), 228 mg **methyl 4,6-bis[3,3-dideuterio-3-(trideuteriomethoxy)prop-1-ynyl]-8-nitro-quinoline-2-carboxylate** was obtained as pale brown crystals (96% purity, 0.577 mmol, 90%).

^{1}H NMR (500 MHz, [D$_6$]DMSO): δ 8.60 (d, *J* = 1.8 Hz, 1H, Ar-H), 8.47 (d, *J* = 1.8 Hz, 1H, Ar-H), 8.30 (s, 1H, Ar-H), 3.95 (s, 3H, C(O)OCH$_3$). HRMS (ESI): *m/z* [M+H]$^+$ calcd for $C_{19}D_{10}H_6N_2O_6$: 379.1709; found: 379.1711.

methyl 3-deuterio-4,6-bis[3,3-dideuterio-3-(trideuteriomethoxy)prop-1-ynyl]-8-nitro-quinoline-2-carboxylate (11j)

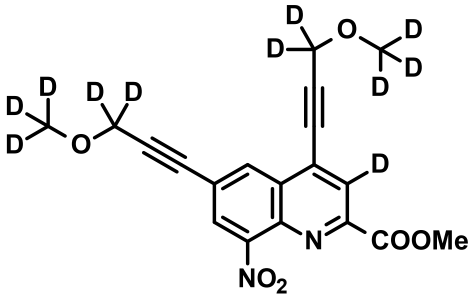

Using **General Procedure 2B** and 250 mg **methyl 4,6-dibromo-3-deuterio-8-nitro-quinoline-2-carboxylate** (0.639 mmol, 1.0 equiv.), and 1.203 g ***tert*-butyl-[3,3-dideuterio-3-(trideuteriomethoxy)prop-1-ynyl]-diphenyl-silane** (3.836 mmol, 6.0 equiv.), 225 mg **methyl 3-deuterio-4,6-bis[3,3-dideuterio-3-(trideuteriomethoxy)prop-1-ynyl]-8-nitro-quinoline-2-carboxylate** was obtained as pale brown crystals (91% purity, 0.540 mmol, 84%).

^{1}H NMR (500 MHz, [D$_6$]DMSO): δ 8.59 (d, *J* = 1.8 Hz, 1H, Ar-H), 8.48 (d, *J* = 1.8 Hz, 1H, Ar-H), 3.96 (s, 3H, C(O)OCH$_3$). HRMS (ESI): *m/z* [M+H]$^+$ calcd for $C_{19}D_{11}H_5N_2O_6$: 380.1772; found: 380.1775.

methyl 5,7-dideuterio-4,6-bis[3,3-dideuterio-3-(trideuteriomethoxy)prop-1-ynyl]-8-nitro-quinoline-2-carboxylate (11k)

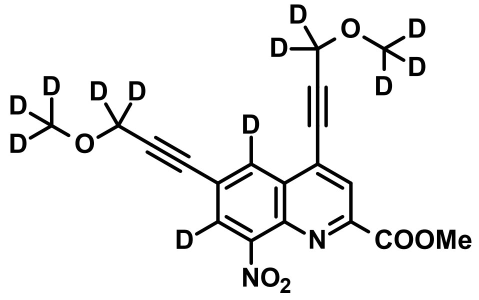

Using **General Procedure 2B** and 250 mg **methyl 4,6-dibromo-5,7-dideuterio-8-nitro-quinoline-2-carboxylate** (0.638 mmol, 1.0 equiv.), and 1.200 g ***tert*-butyl-[3,3-dideuterio-3-(trideuteriomethoxy)prop-1-ynyl]-diphenyl-silane** (3.827 mmol, 6.0 equiv.), 236 mg **methyl 5,7-dideuterio-4,6-bis[3,3-dideuterio-3-(trideuteriomethoxy)prop-1-ynyl]-8-nitro-quinoline-2-carboxylate** was obtained as pale brown crystals (91% purity, 0.5335 mmol, 84%).

^{1}H NMR (500 MHz, [D$_6$]DMSO): 8.30 (s, 1H), 3.96 (s, 3H, C(O)OCH$_3$). HRMS (ESI): *m/z* [M+H]$^+$ calcd for $C_{19}D_{12}H_4N_2O_6$: 381.1834; found: 381.1835.

methyl 3,5,7-trideuterio-4,6-bis[3,3-dideuterio-3-(trideuteriomethoxy)prop-1-ynyl]-8-nitro-quinoline-2-carboxylate (11l)

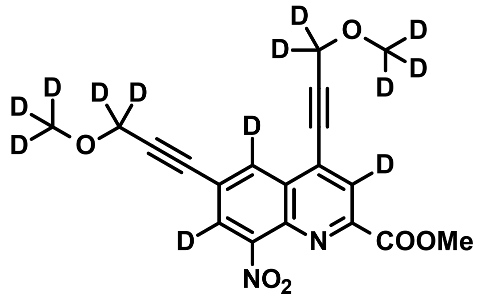

Using **General Procedure 2B** and 250 mg **methyl 4,6-dibromo-3,5,7-trideuterio-8-nitro-quinoline-2-carboxylate** (0.636 mmol, 1.0 equiv.), and 1.197 g ***tert*-butyl-[3,3-dideuterio-3-(trideuteriomethoxy)prop-1-ynyl]-diphenyl-silane** (3.817 mmol, 6.0 equiv.), 187 mg **methyl 3,5,7-trideuterio-4,6-bis[3,3-dideuterio-3-(trideuteriomethoxy)prop-1-ynyl]-8-nitro-quinoline-2-carboxylate** was obtained as a pale brown crystals (85% purity, 0.417 mmol, 66%).

^{1}H NMR (500 MHz, [D$_6$]DMSO): δ 3.96 (s, 3H, C(O)OCH$_3$). HRMS (ESI): *m/z* [M+H]$^+$ calcd for $C_{19}D_{13}H_{13}N_2O_6$: 382.1897; found: 382.1900.

methyl 8-amino-4,6-bis(3-methoxyprop-1-ynyl)quinoline-2-carboxylate (21)

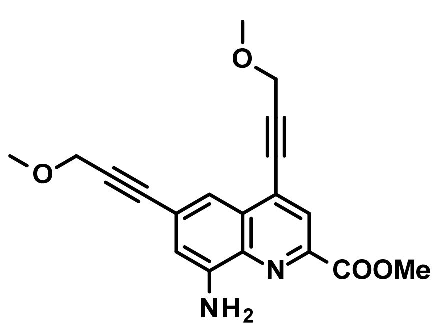

Using **General Procedure 3** and 500 mg **methyl 4,6-bis(3-methoxyprop-1-ynyl)-8-nitro-quinoline-2-carboxylate** (1.36 mmol, 1.0 equiv.), 491 mg **methyl 8-amino-4,6-bis(3-methoxyprop-1-ynyl)quinoline-2-carboxylate** was obtained as a yellow oil (90% purity, 1.31 mmol, 96%).

^{1}H NMR (500 MHz, [D$_6$]DMSO): δ 8.07 (s, 1H, Ar-H), 7.34 (d, *J* = 1.7 Hz, 1H, Ar-H), 6.96 (d, *J* = 1.7 Hz, 1H, Ar-H), 6.39 (s, 2H, NH$_2$), 4.54 (s, 2H, CH$_2$), 4.38 (s, 2H, CH$_2$), 3.94 (s, 3H, C(O)OCH$_3$), 3.41 (s, 3H, OCH$_3$), 3.36 (s, 3H, OCH$_3$). ^{13}C NMR (125 MHz, [D$_6$]DMSO): δ 164.5, 147.2, 143.9, 136.0, 128.7, 128.5, 124.4, 124.2, 113.4, 111.3, 96.9, 87.6, 86.1, 81.1, 59.6, 59.5, 57.3, 57.1, 52.8. HRMS (ESI): *m/z* [M+H]$^+$ calcd for $C_{19}H_{18}N_2O_4$: 339.1339; found: 339.1340.

methyl 8-amino-3-deuterio-4,6-bis(3-methoxyprop-1-ynyl)quinoline-2-carboxylate (22)

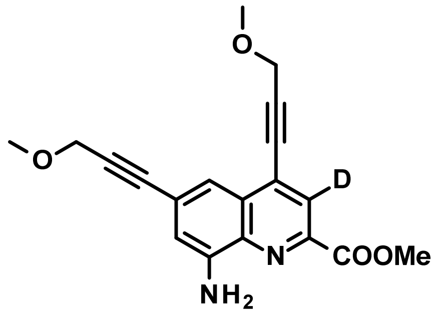

Using **General Procedure 3** and 762 mg **methyl 3-deuterio-4,6-bis(3-methoxyprop-1-ynyl)-8-nitro-quinoline-2-carboxylate** (2.06 mmol, 1.0 equiv.), 652 mg **methyl 8-amino-3-deuterio-4,6-bis(3-methoxyprop-1-ynyl)quinoline-2-carboxylate** was obtained as a yellow oil (91% purity, 1.75 mmol, 85%).

^{1}H NMR (400 MHz, [D$_6$]DMSO): δ 7.34 (d, *J* = 1.7 Hz, 1H, Ar-H), 6.96 (d, *J* = 1.7 Hz, 1H, Ar-H), 6.38 (s, 2H, NH$_2$), 4.54 (s, 2H, CH$_2$), 4.38 (s, 2H, CH$_2$), 3.94 (s, 3H, C(O)OCH$_3$), 3.42 (s, 3H, OCH$_3$), 3.36 (s, 3H, OCH$_3$). HRMS (ESI): *m/z* [M+H]$^+$ calcd for $C_{19}DH_{17}N_2O_4$: 340.1402; found: 340.1402.

methyl 8-amino-3,5,7-trideuterio-4,6-bis(3-methoxyprop-1-ynyl)quinoline-2-carboxylate (23)

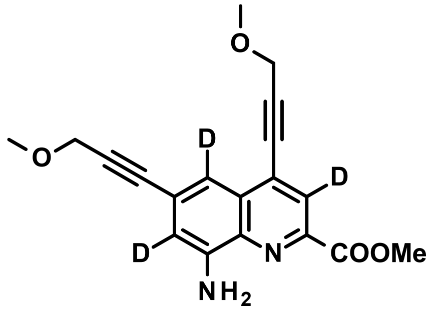

Using **General Procedure 3** and 390 mg **methyl 3,5,7-trideuterio-4,6-bis(3-methoxyprop-1-ynyl)-8-nitro-quinoline-2-carboxylate** (91% purity, 0.9556 mmol, 1.0 equiv.), 466 mg **methyl 8-amino-3,5,7-trideuterio-4,6-bis(3-methoxyprop-1-ynyl)quinoline-2-carboxylate** was obtained as a yellow oil (49% purity, 0.6688 mmol, 70%).

^{1}H NMR (500 MHz, [D$_6$]DMSO): δ 6.37 (s, 2H, NH$_2$), 4.54 (s, 2H, CH$_2$), 4.38 (s, 2H, CH$_2$), 3.95 (s, 3H, C(O)OCH$_3$), 3.42 (s, 3H, OCH$_3$), 3.36 (s, 3H, OCH$_3$). HRMS (ESI): *m/z* [M+H]$^+$ calcd for $C_{19}D_3H_{15}N_2O_4$: 342.1528; found: 342.1526.

methyl 8-amino-4,6-bis[3-(trideuteriomethoxy)prop-1-ynyl]quinoline-2-carboxylate (24)

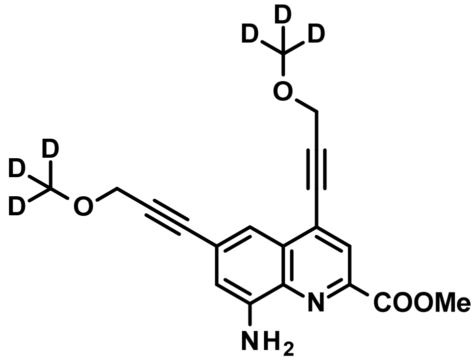

Using **General Procedure 3** and 2.300 g **methyl 8-nitro-4,6-bis[3-(trideuteriomethoxy)prop-1-ynyl]quinoline-2-carboxylate** (92% purity, 5.652 mmol, 1.0 equiv.), 1.562 g **methyl 8-amino-4,6-bis[3-(trideuteriomethoxy)prop-1-ynyl]quinoline-2-carboxylate** was obtained as a yellow oil (93% purity, 4.218 mmol, 75%).

^{1}H NMR (400 MHz, [D$_6$]DMSO): δ 8.07 (s, 1H, Ar-H), 7.34 (d, *J* = 1.7 Hz, 1H, Ar-H), 6.96 (d, *J* = 1.7 Hz, 1H, Ar-H), 6.39 (s, 2H, NH$_2$), 4.54 (s, 2H, CH$_2$), 4.38 (s, 2H, CH$_2$), 3.94 (s, 3H, C(O)OCH$_3$). HRMS (ESI): *m/z* [M+H]$^+$ calcd for $C_{19}D_6H_{12}N_2O_4$: 345.1716; found: 345.1719.

methyl 8-amino-3-deuterio-4,6-bis[3-(trideuteriomethoxy)prop-1-ynyl]quinoline-2-carboxylate (25)

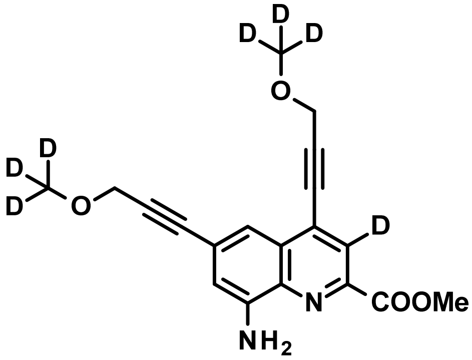

Using **General Procedure 3** and 1.500 g **methyl 3-deuterio-8-nitro-4,6-bis[3-(trideuteriomethoxy)prop-1-ynyl]quinoline-2-carboxylate** (81% purity, 3.237 mmol, 1.0 equiv.), 1.016 g **methyl 8-amino-3-deuterio-4,6-bis[3-(trideuteriomethoxy)prop-1-ynyl]quinoline-2-carboxylate** was obtained as a yellow oil (91% purity, 2.677 mmol, 83%).

^{1}H NMR (400 MHz, [D$_6$]DMSO): δ 7.34 (d, *J* = 1.7 Hz, 1H, Ar-H), 6.96 (d, *J* = 1.7 Hz, 1H, Ar-H), 6.38 (s, 2H, NH$_2$), 4.54 (s, 2H, CH$_2$), 4.38 (s, 2H, CH$_2$), 3.94 (s, 3H, C(O)OCH$_3$). HRMS (ESI): *m/z* [M+H]$^+$ calcd for $C_{19}D_7H_{11}N_2O_4$: 346.1779; found: 346.1784.

methyl 8-amino-5,7-dideuterio-4,6-bis[3-(trideuteriomethoxy)prop-1-ynyl]quinoline-2-carboxylate (26)

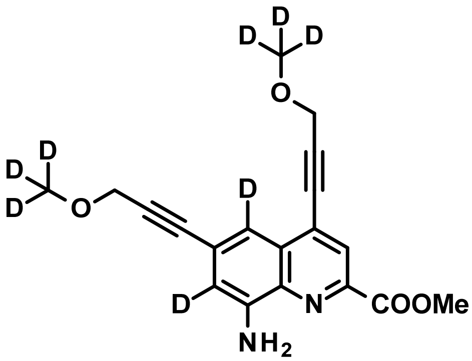

Using **General Procedure 3** and 1.500 g **methyl 5,7-dideuterio-8-nitro-4,6-bis[3-(trideuteriomethoxy)prop-1-ynyl]quinoline-2-carboxylate** (89% purity, 3.547 mmol, 1.0 equiv.), 1.102 g **methyl 8-amino-5,7-dideuterio-4,6-bis[3-(trideuteriomethoxy)prop-1-ynyl]quinoline-2-carboxylate** was obtained as a yellow oil (90% purity, 2.863 mmol, 81%).

^{1}H NMR (400 MHz, [D$_6$]DMSO): δ 8.08 (s, 1H, Ar-H), 6.38 (s, 2H, NH$_2$), 4.54 (s, 2H, CH$_2$), 4.38 (s, 2H, CH$_2$), 3.94 (s, 3H, C(O)OCH$_3$). HRMS (ESI): *m/z* [M+H]$^+$ calcd for $C_{19}D_8H_{10}N_2O_4$: 347.1841; found: 347.1842.

methyl 8-amino-4,6-bis(3-methoxypropyl)quinoline-2-carboxylate (12a)

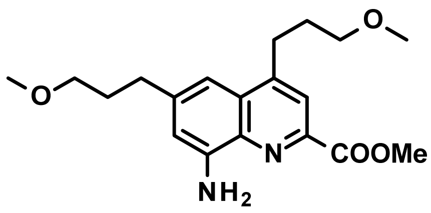

Using **General Procedure 4A** and 609 mg **methyl 4,6-bis(3-methoxyprop-1-ynyl)-8-nitro-quinoline-2-carboxylate** (1.65 mmol, 1.0 equiv.), 522 mg **methyl 8-amino-4,6-bis(3-methoxypropyl)quinoline-2-carboxylate** was obtained as a yellow oil (1.51 mmol, 91%).

^{1}H NMR (500 MHz, [D$_6$]DMSO): δ 7.86 (s, 1H, Ar-H), 7.04 (d, *J* = 1.6 Hz, 1H, Ar-H), 6.80 (d, *J* = 1.6 Hz, 1H, Ar-H), 6.03 (s, 2H, NH$_2$), 3.92 (s, 3H, C(O)OCH$_3$), 3.38 (t, *J* = 6.3 Hz, 2H, CH$_2$O), 3.35 (t, *J* = 6.3 Hz, 2H, CH$_2$O), 3.26 (s, 3H, OCH$_3$), 3.25 (s, 3H, OCH$_3$), 3.05 (t, *J* = 7.6 Hz, 2H, CH$_2$), 2.69 (t, *J* = 7.6 Hz, 2H, CH$_2$) 1.93-1.81 (m, 4H, CH$_2$). ^{13}C NMR (125 MHz, [D$_6$]DMSO): δ 165.5, 148.2,

146.5, 144.0, 142.6, 135.6, 128.8, 120.4, 109.9, 108.3, 71.2, 71.1, 57.87, 57.86, 52.4, 32.7, 30.7, 29.2, 28.3. HRMS (ESI): *m/z* $[M+H]^+$ calcd for $C_{19}H_{26}N_2O_4$: 347.1965; found: 347.1967.

methyl 8-amino-3-deuterio-4,6-bis(3-methoxypropyl)quinoline-2-carboxylate (12b)

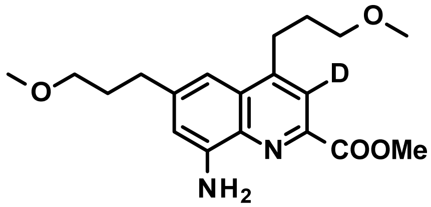

Using **General Procedure 4A** and 1.000 g **methyl 3-deuterio-4,6-bis(3-methoxyprop-1-ynyl)-8-nitro-quinoline-2-carboxylate** (3.472 mmol, 1.0 equiv.), 696 mg **methyl 8-amino-3-deuterio-4,6-bis(3-methoxypropyl)quinoline-2-carboxylate** was obtained as a yellow oil (87% purity, 1.74 mmol, 64%).

^{1}H NMR (400 MHz, [D$_6$]DMSO): δ 7.05 (d, *J* = 1.5 Hz, 1H, Ar-H), 6.80 (d, *J* = 1.5 Hz, 1H, Ar-H), 6.03 (s, 2H, NH_2), 3.92 (s, 3H, $C(O)OCH_3$), 3.39 (t, *J* = 6.3 Hz, 2H, CH_2O), 3.35 (t, *J* = 6.3 Hz, 2H, CH_2O), 3.26 (s, 3H, OCH_3), 3.25 (s, 3H, OCH_3), 3.09-3.02 (m, 2H, CH_2), 2.69 (t, *J* = 7.9 Hz, 2H, CH_2) 1.95-1.81 (m, 4H, CH_2). HRMS (ESI): *m/z* $[M+H]^+$ calcd for $C_{19}DH_{25}N_2O_4$: 348.2028; found: 348.2030.

methyl 8-amino-5,7-dideuterio-4,6-bis(3-methoxypropyl)quinoline-2-carboxylate (12c)

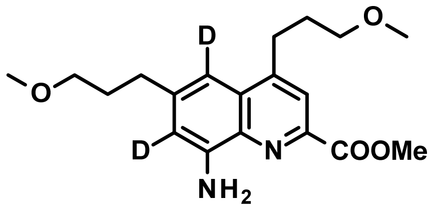

Using **General Procedure 4A** and 500 mg **methyl 5,7-dideuterio-4,6-bis(3-methoxyprop-1-ynyl)-8-nitro-quinoline-2-carboxylate** (1.35 mmol, 1.0 equiv.), 370 mg **methyl 8-amino-5,7-dideuterio-4,6-bis(3-methoxypropyl)quinoline-2-carboxylate** was obtained as a yellow oil (1.06 mmol, 79%).

^{1}H NMR (400 MHz, [D$_6$]DMSO): δ 7.85 (s, 1H, Ar-H), 6.03 (s, 2H, NH_2), 3.92 (s, 3H, $C(O)OCH_3$), 3.38 (t, *J* = 6.5 Hz, 2H, CH_2O), 3.35 (t, *J* = 6.5 Hz, 2H, CH_2O), 3.26 (s, 3H, OCH_3), 3.25 (s, 3H, OCH_3), 3.05 (t, *J* = 7.4 Hz, 2H, CH_2), 2.69 (t, *J* = 7.4 Hz, 2H, CH_2) 1.93-1.81 (m, 4H, CH_2). HRMS (ESI): *m/z* $[M+H]^+$ calcd for $C_{19}D_2H_{24}N_2O_4$: 349.2091; found: 349.2095.

methyl 8-amino-4,6-bis[3-(trideuteriomethoxy)propyl]quinoline-2-carboxylate (12d)

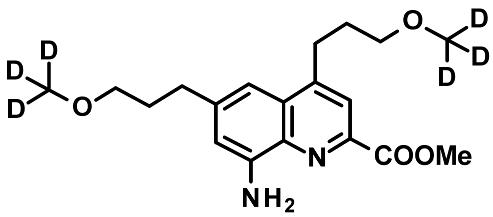

Using **General Procedure 4A** and 700 mg **methyl 8-amino-4,6-bis[3-(trideuteriomethoxy)prop-1-ynyl]quinoline-2-carboxylate** (93% purity, 1.89 mmol, 1.0 equiv.), 654 mg **methyl 8-amino-4,6-bis[3-(trideuteriomethoxy)propyl]quinoline-2-carboxylate** was obtained as a yellow oil (93% purity, 1.73 mmol, 91%).

^{1}H NMR (400 MHz, [D$_6$]DMSO): δ 7.86 (s, 1H, Ar-H), 7.04 (d, *J* = 1.6 Hz, 1H, Ar-H), 6.80 (d, *J* = 1.6 Hz, 1H, Ar-H), 6.04 (s, 2H, NH_2), 3.92 (s, 3H, $C(O)OCH_3$), 3.38 (t, *J* = 6.3 Hz, 2H, CH_2O), 3.35 (t, *J* = 6.3 Hz, 2H, CH_2O), 3. 05 (t, *J* = 7.6 Hz, 2H, CH_2), 2.69 (t, *J* = 7.6 Hz, 2H, CH_2) 1.94-1.81 (m, 4H, CH_2). HRMS (ESI): *m/z* $[M+H]^+$ calcd for $C_{19}D_6H_{20}N_2O_4$: 353.2342; found: 353.2346.

methyl 8-amino-3-deuterio-4,6-bis[3-(trideuteriomethoxy)propyl]quinoline-2-carboxylate (12e)

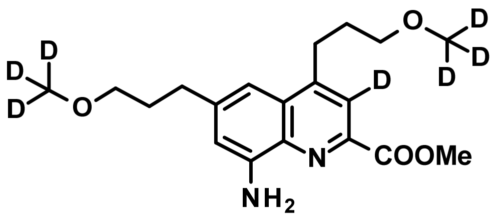

Using **General Procedure 4A** and 600 mg **methyl 8-amino-3-deuterio-4,6-bis[3-(trideuteriomethoxy)prop-1-ynyl]quinoline-2-carboxylate** (91% purity, 1.58 mmol, 1.0 equiv.), 599 mg **methyl 8-amino-3-deuterio-4,6-bis[3-(trideuteriomethoxy)propyl]quinoline-2-carboxylate** was obtained as a yellow oil (87% purity, 1.47 mmol, 93%).

^{1}H NMR (400 MHz, [D$_6$]DMSO): δ 7.05 (d, *J* = 1.6 Hz, 1H, Ar-H), 6.80 (d, *J* = 1.6 Hz, 1H, Ar-H), 6.03 (s, 2H, NH_2), 3.92 (s, 3H, C(O)OCH_3), 3.38 (t, *J* = 6.3 Hz, 2H, CH_2O), 3.35 (t, *J* = 6.3 Hz, 2H, CH_2O), 3. 05 (t, *J* = 7.6 Hz, 2H, CH_2), 2.69 (t, *J* = 7.6 Hz, 2H, CH_2) 1.95-1.81 (m, 4H, CH_2). HRMS (ESI): *m/z* $[M+H]^+$ calcd for $C_{19}D_7H_{19}N_2O_4$: 354.2405; found: 354.2406.

methyl 8-amino-5,7-dideuterio-4,6-bis[3-(trideuteriomethoxy)propyl]quinoline-2-carboxylate (12f)

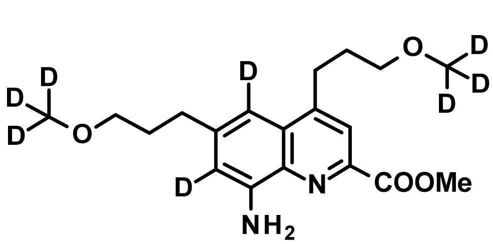

Using **General Procedure 4A** and 650 mg **methyl 8-amino-5,7-dideuterio-4,6-bis[3-(trideuteriomethoxy)prop-1-ynyl]quinoline-2-carboxylate** (90% purity, 1.689 mmol, 1.0 equiv.), 636 mg **methyl 8-amino-5,7-dideuterio-4,6-bis[3-(trideuteriomethoxy)propyl]quinoline-2-carboxylate** was obtained as a yellow oil (84% purity, 1.51 mmol, 89%).

^{1}H NMR (400 MHz, [D$_6$]DMSO): δ 7.85 (s, 1H, Ar-H), 6.02 (s, 2H, NH_2), 3.92 (s, 3H, C(O)OCH_3), 3.38 (t, *J* = 6.3 Hz, 2H, CH_2O), 3.35 (t, *J* = 6.3 Hz, 2H, CH_2O), 3. 05 (t, *J* = 7.5 Hz, 2H, CH_2), 2.69 (t, *J* = 7.5 Hz, 2H, CH_2) 1.94-1.81 (m, 4H, CH_2). HRMS (ESI): *m/z* $[M+H]^+$ calcd for $C_{19}D_8H_{18}N_2O_4$: 355.2467; found: 355.2470.

methyl 8-amino-4,6-bis(1,1,2,2-tetradeuterio-3-methoxy-propyl)quinoline-2-carboxylate (27)

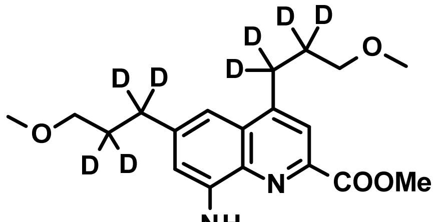

Using **General Procedure 4B** and 600 mg **methyl 8-amino-4,6-bis(3-methoxyprop-1-ynyl)quinoline-2-carboxylate** (88% purity, 1.56 mmol, 1.0 equiv.), 323 mg **methyl 8-amino-4,6-bis(1,1,2,2-tetradeuterio-3-methoxy-propyl)quinoline-2-carboxylate** was obtained as a yellow oil (87% purity, 0.793 mmol, 51%).

^{1}H NMR (400 MHz, [D$_6$]DMSO): δ 7.86 (s, 1H, Ar-H), 7.04 (s, 1H, Ar-H), 6.80 (s, 1H, Ar-H), 6.02 (s, 2H, NH_2), 3.92 (s, 3H, C(O)OCH_3), 3.37 (s, 2H, CH_2O), 3.33 (s, 2H, CH_2O), 3.25 (s, 3H, OCH_3), 3.24 (s, 3H, OCH_3). HRMS (ESI): *m/z* $[M+H]^+$ calcd for $C_{19}D_8H_{18}N_2O_4$: 355.2467; found: 355.2469.

methyl 8-amino-3-deuterio-4,6-bis(1,1,2,2-tetradeuterio-3-methoxy-propyl)quinoline-2-carboxylate (28)

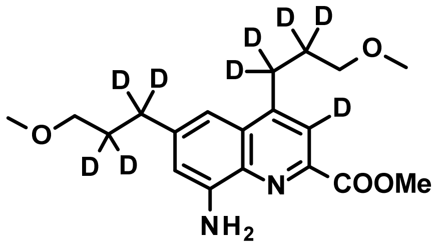

Using **General Procedure 4B** and 490 mg **methyl 8-amino-3-deuterio-4,6-bis(3-methoxyprop-1-ynyl)quinoline-2-carboxylate** (91% purity, 1.31 mmol, 1.0 equiv.), 450 mg **methyl 8-amino-3-deuterio-4,6-bis(1,1,2,2-tetradeuterio-3-methoxy-propyl)quinoline-2-carboxylate** was obtained as a yellow oil (91% purity, 1.15 mmol, 88%).

^{1}H NMR (400 MHz, [D$_6$]DMSO): 7.05 (d, *J* = 1.6 Hz, 1H, Ar-H), 6.80 (d, *J* = 1.6 Hz, 1H, Ar-H), 6.01 (s, 2H, NH_2), 3.92 (s, 3H, C(O)OCH_3), 3.37 (s, 2H, CH_2O), 3.34 (s, 2H, CH_2O), 3.25 (s, 3H, OCH_3), 3.24 (s, 3H, OCH_3). HRMS (ESI): *m/z* $[M+H]^+$ calcd for $C_{19}D_9H_{17}N_2O_4$: 356.2530; found:356.2530.

methyl 8-amino-5,7-dideuterio-4,6-bis(1,1,2,2-tetradeuterio-3-methoxy-propyl)quinoline-2-carboxylate (12g)

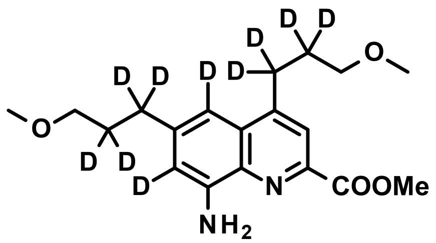

Using **General Procedure 4B** and 400 mg **methyl 5,7-dideuterio-4,6-bis(3-methoxyprop-1-ynyl)-8-nitro-quinoline-2-carboxylate** (1.08 mmol, 1.0 equiv.), 321 mg **methyl 8-amino-5,7-dideuterio-4,6-bis(1,1,2,2-tetradeuterio-3-methoxy-propyl)quinoline-2-carboxylate** was obtained as a yellow oil (94% purity, 0.821 mmol, 76%).

^{1}H NMR (400 MHz, [D$_6$]DMSO): δ 7.86 (s, 1H, Ar-H), 6.02 (s, 2H, NH$_2$), 3.92 (s, 3H, C(O)OCH$_3$), 3.37 (s, 2H, CH$_2$O), 3.34 (s, 2H, CH$_2$O), 3.25 (s, 3H, OCH$_3$), 3.24 (s, 3H, OCH$_3$). HRMS (ESI): *m/z* [M+H]$^+$ calcd for $C_{19}D_{10}H_{16}N_2O_4$: 357.2593; found: 357.2599.

methyl 8-amino-4,6-bis[1,1,2,2-tetradeuterio-3-(trideuteriomethoxy)propyl]quinoline-2-carboxylate (12h)

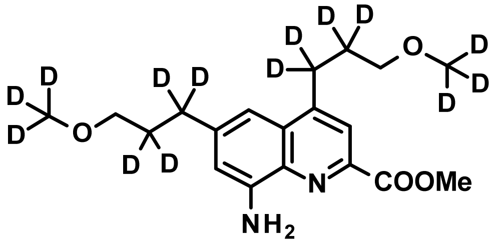

Using **General Procedure 4B** and 406 mg **methyl 8-amino-4,6-bis[3-(trideuteriomethoxy)prop-1-ynyl]quinoline-2-carboxylate** (93% purity, 1.10 mmol, 1.0 equiv.), 401 mg **methyl 8-amino-4,6-bis[1,1,2,2-tetradeuterio-3-(trideuteriomethoxy)propyl]quinoline-2-carboxylate** was obtained as a yellow oil (88% purity, 0.979 mmol, 89%).

^{1}H NMR (500 MHz, [D$_6$]DMSO): δ 7.86 (s, 1H, Ar-H), 7.04 (d, *J* = 1.5 Hz, 1H, Ar-H), 6.80 (d, *J* = 1.5 Hz, 1H, Ar-H), 6.03 (s, 2H, NH$_2$), 3.92 (s, 3H, C(O)OCH$_3$), 3.37 (s, 2H, CH$_2$O), 3.33 (s, 2H, CH$_2$O). HRMS (ESI): *m/z* [M+H]$^+$ calcd for $C_{19}D_{14}H_{12}N_2O_4$: 361.2844; found: 361.2845.

methyl 8-amino-3-deuterio-4,6-bis[1,1,2,2-tetradeuterio-3-(trideuteriomethoxy)propyl]quinoline-2-carboxylate (12i)

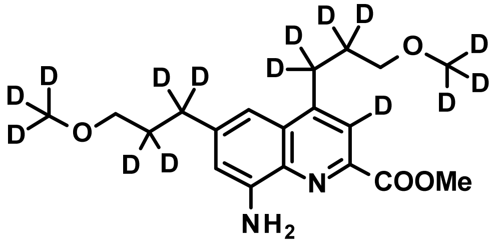

Using **General Procedure 4B** and 490 mg **methyl 8-amino-3-deuterio-4,6-bis[3-(trideuteriomethoxy)prop-1-ynyl]quinoline-2-carboxylate** (81% purity, 1.14 mmol, 1.0 equiv.), 472 mg **methyl 8-amino-3-deuterio-4,6-bis[1,1,2,2-tetradeuterio-3-(trideuteriomethoxy)propyl]quinoline-2-carboxylate** was obtained as a yellow oil (80% purity, 1.05 mmol, 92%).

^{1}H NMR (400 MHz, [D$_6$]DMSO): δ 7.04 (d, *J* = 1.4 Hz, 1H, Ar-H), 6.80 (d, *J* = 1.4 Hz, 1H, Ar-H), 6.03 (s, 2H, NH$_2$), 3.92 (s, 3H, C(O)OCH$_3$), 3.37 (s, 2H, CH$_2$O), 3.34 (s, 2H, CH$_2$O). HRMS (ESI): *m/z* [M+H]$^+$ calcd for $C_{19}D_{15}H_{11}N_2O_4$: 362.2907; found: 362.2911.

methyl 8-amino-5,7-dideuterio-4,6-bis[1,1,2,2-tetradeuterio-3-(trideuteriomethoxy)propyl]quinoline-2-carboxylate (12j)

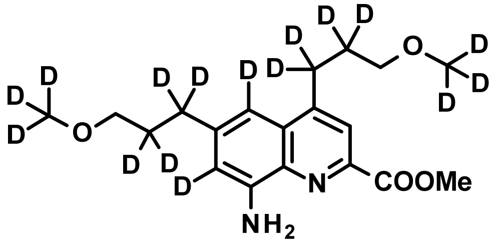

Using **General Procedure 4B** and 160 mg **methyl 8-amino-5,7-dideuterio-4,6-bis[3-(trideuteriomethoxy)prop-1-ynyl]quinoline-2-carboxylate** (90% purity, 0.416 mmol, 1.0 equiv.), 158 mg **methyl 8-amino-5,7-dideuterio-4,6-bis[1,1,2,2-tetradeuterio-3-(trideuteriomethoxy)propyl]quinoline-2-carboxylate** was obtained as a yellow oil (88% purity, 0.384 mmol, 92%).

^{1}H NMR (500 MHz, [D$_6$]DMSO): δ 7.86 (s, 1H, Ar-H), 6.02 (s, 2H, NH$_2$), 3.92 (s, 3H, C(O)OCH$_3$), 3.37 (s, 2H, CH$_2$O), 3.34 (s, 2H, CH$_2$O). HRMS (ESI): *m/z* [M+H]$^+$ calcd for $C_{19}D_{16}H_{10}N_2O_4$: 363.2970; found: 363.2969.

methyl 8-amino-3,5,7-trideuterio-4,6-bis[1,1,2,2-tetradeuterio-3-(trideuteriomethoxy)propyl]quinoline-2-carboxylate (12k)

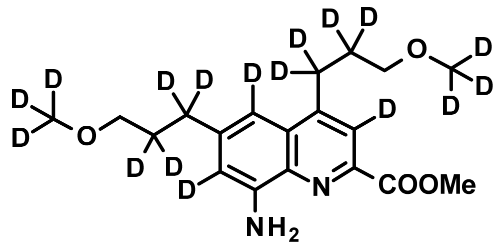

Using **General Procedure 4B** and 532 mg **methyl 3,5,7-trideuterio-8-nitro-4,6-bis[3-(trideuteriomethoxy)prop-1-ynyl]quinoline-2-carboxylate** (94% purity, 1.33 mmol, 1.0 equiv.), 421 mg **methyl 8-amino-3,5,7-trideuterio-4,6-bis[1,1,2,2-tetradeuterio-3-(trideuteriomethoxy)propyl]quinoline-2-carboxylate** was obtained as a yellow oil (93% purity, 1.08 mmol, 81%).

^{1}H NMR (400 MHz, [D$_6$]DMSO): δ 6.02 (s, 2H, NH_2), 3.92 (s, 3H, C(O)OCH_3), 3.37 (s, 2H, CH_2O), 3.34 (s, 2H, CH_2O). HRMS (ESI): *m/z* [M+H]$^+$ calcd for $C_{19}D_{17}H_9N_2O_4$: 364.3032; found: 364.3035.

methyl 8-amino-4,6-bis[1,1,2,2,3,3-hexadeuterio-3-(trideuteriomethoxy)propyl]quinoline-2-carboxylate (12l)

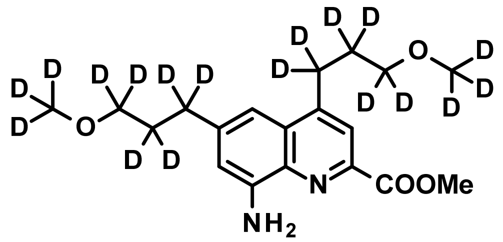

Using **General Procedure 4B** and 210 mg **methyl 4,6-bis[3,3-dideuterio-3-(trideuteriomethoxy)prop-1-ynyl]-8-nitro-quinoline-2-carboxylate** (96% purity, 0.534 mmol, 1.0 equiv.), 321 mg **methyl 8-amino-4,6-bis[1,1,2,2,3,3-hexadeuterio-3-(trideuteriomethoxy)propyl]quinoline-2-carboxylate** was obtained as a yellow oil (55% purity, 0.484 mmol, 91%). ^{1}H NMR (500 MHz, [D$_6$]DMSO): δ 7.86 (s, 1H, Ar-H), 7.04 (d, *J* = 1.7 Hz, 1H, Ar-H), 6.80 (d, *J* = 1.7 Hz, 1H, Ar-H), 6.03 (s, 2H, NH_2), 3.92 (s, 3H, C(O)OCH_3). HRMS (ESI): *m/z* [M+H]$^+$ calcd for $C_{19}D_{18}H_8N_2O_4$: 365.3095; found: 365.3096.

methyl 8-amino-3-deuterio-4,6-bis[1,1,2,2,3,3-hexadeuterio-3-(trideuteriomethoxy)propyl]quinoline-2-carboxylate (12m)

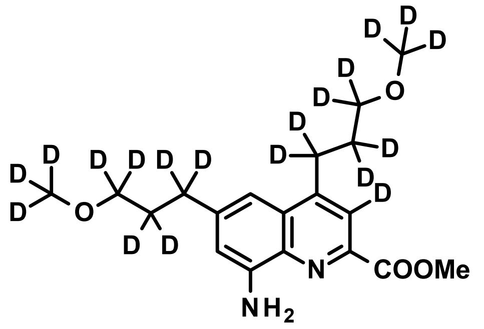

Using **General Procedure 4B** and 225 mg **methyl 3-deuterio-4,6-bis[3,3-dideuterio-3-(trideuteriomethoxy)prop-1-ynyl]-8-nitro-quinoline-2-carboxylate** (91% purity, 0.540 mmol, 1.0 equiv.), 301 mg **methyl 8-amino-3-deuterio-4,6-bis[1,1,2,2,3,3-hexadeuterio-3-(trideuteriomethoxy)propyl]quinoline-2-carboxylate** was obtained as a yellow oil (52% purity, 0.428 mmol, 79%).

^{1}H NMR (500 MHz, [D$_6$]DMSO): δ 7.04 (d, *J* = 1.7 Hz, 1H, Ar-H), 6.80 (d, *J* = 1.7 Hz, 1H, Ar-H), 6.03 (s, 2H, NH_2), 3.92 (s, 3H, C(O)OCH_3). HRMS (ESI): *m/z* [M+H]$^+$ calcd for $C_{19}D_{19}H_7N_2O_4$: 366.3158; found: 366.3158.

methyl 8-amino-5,7-dideuterio-4,6-bis[1,1,2,2,3,3-hexadeuterio-3-(trideuteriomethoxy)propyl]quinoline-2-carboxylate (12n)

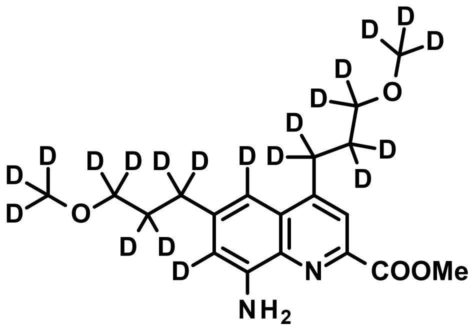

Using **General Procedure 4B** and 212 mg **methyl 5,7-dideuterio-4,6-bis[3,3-dideuterio-3-(trideuteriomethoxy)prop-1-ynyl]-8-nitro-quinoline-2-carboxylate** (91% purity, 0.509 mmol, 1.0 equiv.), 300 mg **methyl 8-amino-5,7-dideuterio-4,6-bis[1,1,2,2,3,3-hexadeuterio-3-(trideuteriomethoxy)propyl]quinoline-2-carboxylate** was obtained as a yellow oil (70% purity, 0.438 mmol, 86%).

^{1}H NMR (500 MHz, [D$_6$]DMSO): δ 7.86 (s, 1H, Ar-H), 6.02 (s, 2H, NH$_2$), 3.92 (s, 3H, C(O)OCH$_3$). HRMS (ESI): *m/z* [M+H]$^+$ calcd for $C_{19}D_{20}H_6N_2O_4$: 367.3221; found: 367.3222.

methyl 8-amino-3,5,7-trideuterio-4,6-bis[1,1,2,2,3,3-hexadeuterio-3-(trideuteriomethoxy)propyl]quinoline-2-carboxylate (12o)

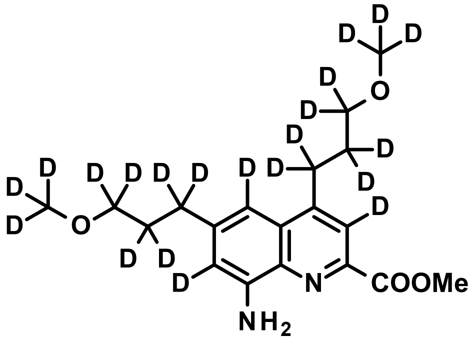

Using **General Procedure 4B** and 180 mg **methyl 3,5,7-trideuterio-4,6-bis[3,3-dideuterio-3-(trideuteriomethoxy)prop-1-ynyl]-8-nitro-quinoline-2-carboxylate** (85% purity, 0.401 mmol, 1.0 equiv.), 230 mg **methyl 8-amino-3,5,7-trideuterio-4,6-bis[1,1,2,2,3,3-hexadeuterio-3-(trideuteriomethoxy)propyl]quinoline-2-carboxylate** was obtained as a yellow oil (61% purity, 0.382 mmol, 95%).

^{1}H NMR (500 MHz, [D$_6$]DMSO): δ 6.02 (s, 2H, NH$_2$), 3.92 (s, 3H, C(O)OCH$_3$). HRMS (ESI): *m/z* [M+H]$^+$ calcd for $C_{19}D_{21}H_5N_2O_4$: 368.3283; found: 368.3284.

methyl 8-acetamido-4,6-bis(3-methoxypropyl)quinoline-2-carboxylate (13a)

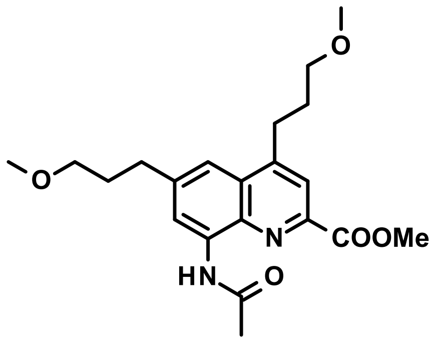

Using **General Procedure 5A** and 696 mg **methyl 8-amino-4,6-bis(3-methoxypropyl)quinoline-2-carboxylate** (2.01 mmol, 1.0 equiv.), 758 mg **methyl 8-acetamido-4,6-bis(3-methoxypropyl)quinoline-2-carboxylate** was obtained as a yellow oil (1.91 mmol, 95%).

^{1}H NMR (500 MHz, [D$_6$]DMSO): δ 9.85 (s, 1H, NH), 8.55 (d, *J* = 1.5 Hz, 1H, Ar-H), 7.98 (s, 1H, Ar-H), 7.64 (d, *J* = 1.5 Hz, 1H, Ar-H), 3.97 (s, 3H, C(O)OCH$_3$), 3.39 (t, *J* = 6.2 Hz, 2H, CH$_2$), 3.37 (t, *J* = 6.2 Hz, 2H, CH$_2$), 3.26 (s, 3H, OCH$_3$), 3.25 (s, 3H, OCH$_3$), 3.15 (t, *J* = 7.7 Hz, 2H, CH$_2$), 2.83 (t, *J* = 7.7 Hz, 2H, CH$_2$), 2.28 (s, 3H, C(O)CH$_3$), 1.97-1.85 (m, 4H, CH$_2$). ^{13}C NMR (125 MHz, [D$_6$]DMSO): δ 168.6, 165.1, 149.7, 144.5, 143.3, 136.2, 135.5, 128.0, 121.0, 118.0, 116.0, 70.97, 70.95, 57.89, 57.88, 52.7, 32.8, 30.7, 29.4, 28.1, 24.8. HRMS (ESI): *m/z* [M+H]$^+$ calcd for $C_{21}H_{28}N_2O_5$: 389.2071; found: 389.2070.

methyl 8-acetamido-3-deuterio-4,6-bis(3-methoxypropyl)quinoline-2-carboxylate (13b)

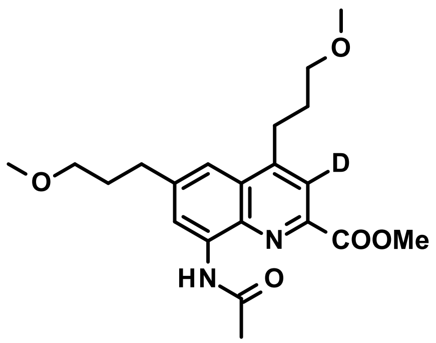

Using **General Procedure 5A** and 690 mg **methyl 8-amino-3-deuterio-4,6-bis(3-methoxypropyl)quinoline-2-carboxylate** (87% purity, 1.73 mmol, 1.0 equiv.), 567 mg **methyl 8-acetamido-3-deuterio-4,6-bis(3-methoxypropyl)quinoline-2-carboxylate** was obtained as a yellow oil (1.46 mmol, 84%).

^{1}H NMR (400 MHz, [D$_6$]DMSO): δ 9.87 (s, 1H, NH), 8.56 (d, *J* = 1.4 Hz, 1H, Ar-H), 7.64 (d, *J* = 1.4 Hz, 1H, Ar-H), 3.97 (s, 3H, C(O)OCH$_3$), 3.39 (t, *J* = 6.3 Hz, 2H, CH$_2$), 3.37 (t, *J* = 6.3 Hz, 2H, CH$_2$), 3.26 (s, 3H, OCH$_3$), 3.25 (s, 3H, OCH$_3$), 3.16 (t, *J* = 7.4 Hz, 2H, CH$_2$), 2.83 (t, *J* = 7.4 Hz, 2H, CH$_2$), 2.28 (s, 3H, C(O)CH$_3$), 1.97-1.85 (m, 4H, CH$_2$). HRMS (ESI): *m/z* [M+H]$^+$ calcd for $C_{21}DH_{27}N_2O_5$: 390.2134; found: 390.2135.

methyl 8-acetamido-5,7-dideuterio-4,6-bis(3-methoxypropyl)quinoline-2-carboxylate (13c)

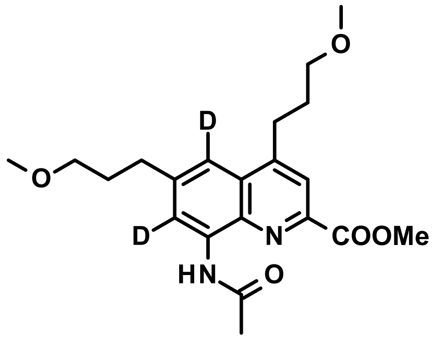

Using **General Procedure 5A** and 180 mg **methyl 8-amino-5,7-dideuterio-4,6-bis(3-methoxypropyl)quinoline-2-carboxylate** (0.517 mmol, 1.0 equiv.), 176 mg **methyl 8-acetamido-5,7-dideuterio-4,6-bis(3-methoxypropyl)quinoline-2-carboxylate** was obtained as a yellow oil (0.451 mmol, 87%).

^{1}H NMR (400 MHz, [D$_6$]DMSO): δ 9.85 (s, 1H, NH), 7.98 (s, 1H, Ar-H), 3.97 (s, 3H, C(O)OCH$_3$), 3.39 (t, *J* = 6.1 Hz, 2H, CH$_2$), 3.37 (t, *J* = 6.1 Hz, 2H, CH$_2$), 3.26 (s, 3H, OCH$_3$), 3.25 (s, 3H, OCH$_3$), 3.15 (t, *J* = 7.7 Hz, 2H, CH$_2$), 2.83 (t, *J* = 7.7 Hz, 2H, CH$_2$), 2.28 (s, 3H, C(O)CH$_3$), 1.97-1.85 (m, 4H, CH$_2$). HRMS (ESI): *m/z* [M+H]$^+$ calcd for $C_{21}D_2H_{26}N_2O_5$: 391.2197; found: 3891.2200.

methyl 8-acetamido-3,5,7-trideuterio-4,6-bis(3-methoxyprop-1-ynyl)quinoline-2-carboxylate (29)

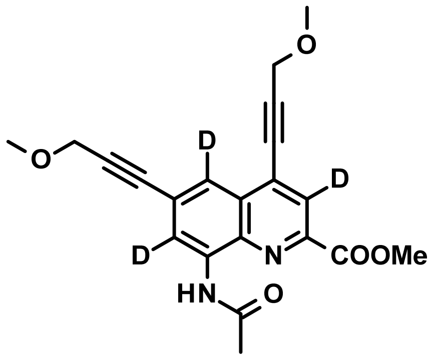

Using **General Procedure 5A** and 380 mg **methyl 8-amino-3,5,7-trideuterio-4,6-bis(3-methoxyprop-1-ynyl)quinoline-2-carboxylate** (49% purity, 0.545 mmol, 1.0 equiv.), 415 mg **methyl 8-acetamido-3,5,7-trideuterio-4,6-bis(3-methoxyprop-1-ynyl)quinoline-2-carboxylate** was obtained as a yellow oil (41% purity, 0.444 mmol, 81%).

^{1}H NMR (500 MHz, [D$_6$]DMSO): δ 9.91 (s, 1H, NH), 4.58 (s, 2H, CH$_2$), 4.43 (s, 2H, CH$_2$), 3.99 (s, 3H, C(O)OCH$_3$), 3.43 (s, 3H, OCH$_3$), 3.38 (s, 3H, OCH$_3$), 2.31 (s, 3H, C(O)CH$_3$). ^{13}C NMR (125 MHz, [D$_6$]DMSO): δ 169.2, 164.2, 146.1, 137.0, 136.0, 129.6, 127.9, 123.4, 98.4, 89.2, 85.3, 80.4, 59.6, 59.5, 57.4, 57.2, 53.1, 24.7. HRMS (ESI): *m/z* [M+H]$^+$ calcd for $C_{21}D_3H_{17}N_2O_5$: 384.1633; found: 384.1637.

methyl 4,6-bis(3-methoxyprop-1-ynyl)-8-[(2,2,2-trideuterioacetyl)amino]quinoline-2-carboxylate (30)

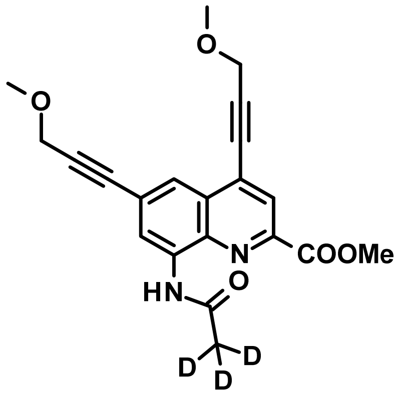

Using **General Procedure 5B** and 280 mg **methyl 8-amino-4,6-bis(3-methoxyprop-1-ynyl)quinoline-2-carboxylate** (94% purity, 0.778 mmol, 1.0 equiv.), 270 mg **methyl 4,6-bis(3-methoxyprop-1-ynyl)-8-[(2,2,2-trideuterioacetyl)amino]quinoline-2-carboxylate** (86% purity, 0.606 mmol, 78%) as a yellow solid.

^{1}H NMR (500 MHz, [D$_6$]DMSO): δ 9.87 (s, 1H, NH), 8.66 (d, *J* = 1.7 Hz, 1H, Ar-H), 8.18 (s, 1H, Ar-H), 7.89 (d, J = 1.7 Hz, 1H, Ar-H), 4.57 (s, 2H, CH_2), 4.43 (s, 2H, CH_2), 3.99 (s, 3H, C(O)OCH_3), 3.43 (s, 3H, OCH_3), 3.38 (s, 3H, OCH_3). ^{13}C NMR (125 MHz, [D$_6$]DMSO): δ 169.3, 164.2, 146.1, 136.9, 136.1, 129.7, 127.9, 124.6, 123.6, 121.6, 119.5, 98.4, 89.2, 85.4, 80.5, 59.6, 59.5, 57.4, 57.2, 53.1. HRMS (ESI): *m/z* $[M+H]^+$ calcd for $C_{21}D_3H_{17}N_2O_5$: 384.1633; found: 384.1634.

methyl 4,6-bis(3-methoxypropyl)-8-[(2,2,2-trideuterioacetyl)amino]quinoline-2-carboxylate (31)

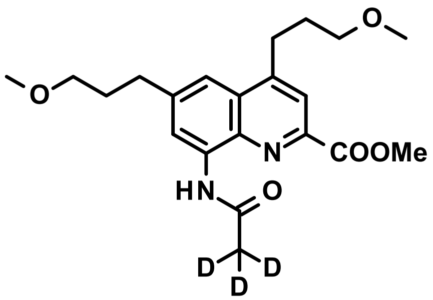

Using **General Procedure 4A** and 250 mg **methyl 4,6-bis(3-methoxyprop-1-ynyl)-8-[(2,2,2-trideuterioacetyl)amino]quinoline-2-carboxylate** (86% purity, 0.561 mmol, 1.0 equiv.), 195 mg **methyl 4,6-bis(3-methoxypropyl)-8-[(2,2,2-trideuterioacetyl)amino]quinoline-2-carboxylate** was obtained as a yellow oil (85% purity, 0.423 mmol, 76%).

^{1}H NMR (500 MHz, [D$_6$]DMSO): δ 9.86 (s, 1H, NH), 8.56 (d, *J* = 1.3 Hz, 1H, Ar-H), 7.99 (s, 1H, Ar-H), 7.65 (d, *J* = 1.3 Hz, 1H, Ar-H), 3.97 (s, 3H, C(O)OCH_3), 3.40 (t, *J* = 6.1 Hz, 2H, CH_2), 3.37 (t, *J* = 6.1 Hz, 2H, CH_2), 3.26 (s, 3H, OCH_3), 3.25 (s, 3H, OCH3), 3.16 (t, *J* = 7.6 Hz, 2H, CH_2), 2.84 (t, *J* = 7.6 Hz, 2H, CH_2), 1.96-1.85 (m, 4H, CH_2). HRMS (ESI): *m/z* $[M+H]^+$ calcd for $C_{21}D_3H_{25}N_2O_5$: 392.2259; found: 392.2260.

[*Note: The efficiency of the deuteroacylation reaction can be enhanced by following a modified procedure of 5B*:

A 25 mL pear-shaped flask equipped with a magnetic stirring bar was filled with 50 mg **methyl 8-amino-4,6-bis(3-methoxyprop-1-ynyl)quinoline-2-carboxylate** (1.0 equiv., 0.144 mmol). The flask was evacuated then backfilled with N_2 (repeated 3x), then 3 mL MeOD (513 equiv., 73.8 mmol) was added. The mixture was stirred at rt for 1 h, then was concentrated *in vacuo*. This was repeated 5 times. Then 3.53 mg *N*,*N*-dimethylpyridin-4-amine (0.2 equiv., 0.0289 mmol), 0.117 mL pyridine (10 equiv., 1.44 mmol), and 2.89 mL EtOAc (20 mL/mmol) were added. Then 0.418 mL (2,2,2-trideuterioacetyl) 2,2,2-trideuterioacetate (3 equiv., 0.433 mmol) was added and the mixture was stirred at 50°C for 18 hours. Then it was washed with water, 1 M aq. citric acid solution, sat. aq. $NaHCO_3$ solution, dried over Na_2SO_4, filtered and the filtrate was concentrated *in vacuo*. Then the crude product was purified *via* flash chromatography using DCM and MeOH as eluents to afford 57 mg **methyl 4,6-bis(3-methoxypropyl)-8-[(2,2,2-trideuterioacetyl)amino]quinoline-2-carboxylate** (67% purity, 0.0976 mmol, 68%) as a yellow oil.

Figure S1. HRMS of the product obtained in the two procedures: 93%D incorporation/position→ 96%.

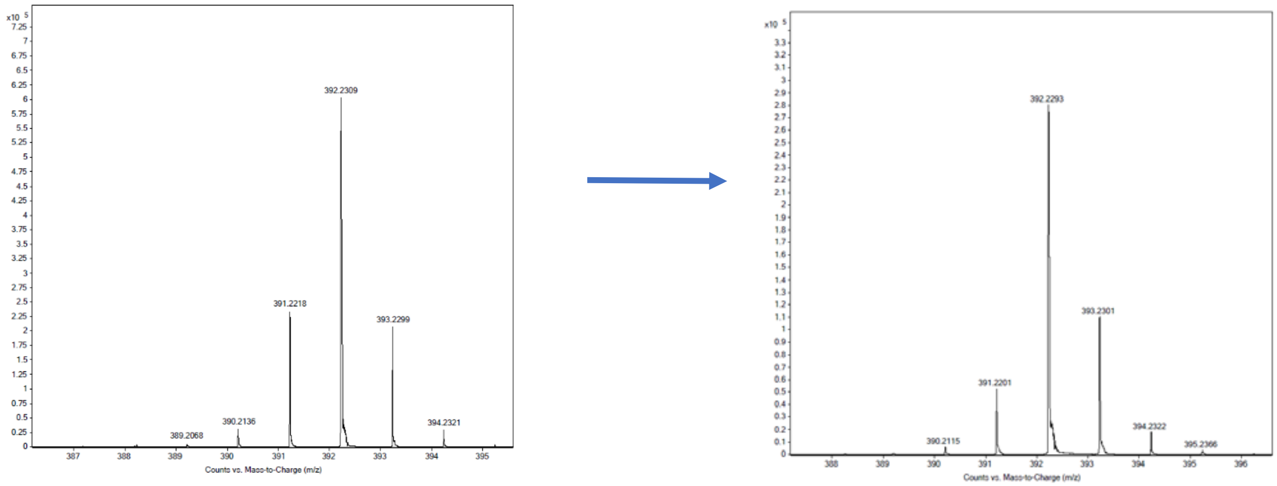

methyl 8-acetamido-3,5,7-trideuterio-4,6-bis(3-methoxypropyl)quinoline-2-carboxylate (13d)

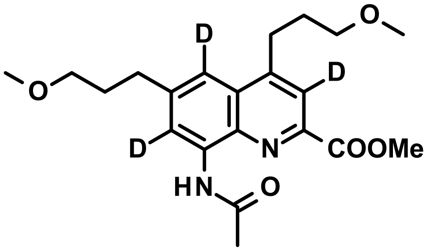

Using **General Procedure 4A** and 400 mg **methyl 8-acetamido-3,5,7-trideuterio-4,6-bis(3-methoxyprop-1-ynyl)quinoline-2-carboxylate** (41% purity, 0.428 mmol, 1.0 equiv.), 180 mg **methyl 8-acetamido-3,5,7-trideuterio-4,6-bis(3-methoxypropyl)quinoline-2-carboxylate** was obtained as a yellow oil (63% purity, 0.290 mmol, 68%).

^{1}H NMR (500 MHz, [D$_6$]DMSO): δ 9.86 (s, 1H, NH), 3.97 (s, 3H, C(O)OCH$_3$), 3.40 (t, *J* = 6.2 Hz, 2H, CH$_2$), 3.37 (t, *J* = 6.2 Hz, 2H, CH$_2$), 3.26 (s, 3H, OCH$_3$), 3.25 (s, 3H, OCH$_3$), 3.17 (t, *J* = 7.7 Hz, 2H, CH$_2$), 2.83 (t, *J* = 7.7 Hz, 2H, CH$_2$), 2.28 (s, 3H, C(O)CH$_3$), 1.97-1.85 (m, 4H, CH$_2$). HRMS (ESI): *m/z* [M+H]$^+$ calcd for $C_{21}D_3H_{25}N_2O_5$: 392.2259; found: 392.2261.

methyl 3-deuterio-4,6-bis(3-methoxypropyl)-8-[(2,2,2-trideuterioacetyl)amino]quinoline-2-carboxylate (13e)

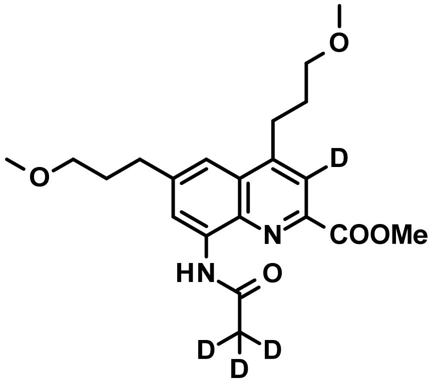

Using **General Procedure 5B** and 380 mg **methyl 8-amino-3-deuterio-4,6-bis(3-methoxypropyl)quinoline-2-carboxylate** (51% purity, 0.558 mmol, 1.0 equiv.), 301 mg **methyl 3-deuterio-4,6-bis(3-methoxypropyl)-8-[(2,2,2-trideuterioacetyl)amino]quinoline-2-carboxylate** was obtained as a yellow oil (65% purity, 0.499 mmol, 89%).

^{1}H NMR (500 MHz, [D$_6$]DMSO): δ 9.86 (s, 1H, NH), 8.56 (d, *J* = 1.2 Hz, 1H, Ar-H), 7.65 (d, *J* = 1.2 Hz, 1H, Ar-H), 3.97 (s, 3H, C(O)OCH$_3$), 3.40 (t, *J* = 6.2 Hz, 2H, CH$_2$), 3.37 (t, *J* = 6.2 Hz, 2H, CH$_2$), 3.26 (s, 3H, OCH$_3$), 3.25 (s, 3H, OCH$_3$), 3.16 (t, *J* = 7.7 Hz, 2H, CH$_2$), 2.84 (t, *J* = 7.7 Hz, 2H, CH$_2$), 1.97-1.85 (m, 4H, CH$_2$). HRMS (ESI): *m/z* [M+H]$^+$ calcd for $C_{21}D_4H_{24}N_2O_5$: 393.2322; found: 393.2324.

methyl 5,7-dideuterio-4,6-bis(3-methoxypropyl)-8-[(2,2,2-trideuterioacetyl)amino]quinoline-2-carboxylate (13f)

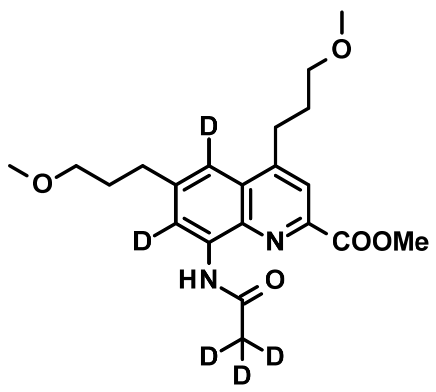

Using **General Procedure 5B** and 228 mg **methyl 8-amino-5,7-dideuterio-4,6-bis(3-methoxypropyl)quinoline-2-carboxylate** (0.581 mmol, 1.0 equiv.), 240 mg **methyl 5,7-dideuterio-4,6-bis(3-methoxypropyl)-8-[(2,2,2-trideuterioacetyl)amino]quinoline-2-carboxylate** was obtained as a yellow oil (87% purity, 0.531 mmol, 91%).

^{1}H NMR (500 MHz, [D$_6$]DMSO): δ 9.86 (s, 1H, NH), 8.00 (s, 1H, Ar-H), 3.97 (s, 3H, C(O)OCH$_3$), 3.40 (t, *J* = 6.2 Hz, 2H, CH$_2$), 3.37 (t, *J* = 6.2 Hz, 2H, CH$_2$), 3.26 (s, 3H, OCH$_3$), 3.25 (s, 3H, OCH$_3$), 3.17 (t, *J* = 7.6 Hz, 2H, CH$_2$), 2.84 (t, *J* = 7.6 Hz, 2H, CH$_2$), 1.97-1.85 (m, 4H, CH$_2$). HRMS (ESI): *m/z* [M+H]$^+$ calcd for $C_{21}D_5H_{23}N_2O_5$: 394.2385; found: 394.2386.

methyl 8-acetamido-4,6-bis[3-(trideuteriomethoxy)prop-1-ynyl]quinoline-2-carboxylate (32)

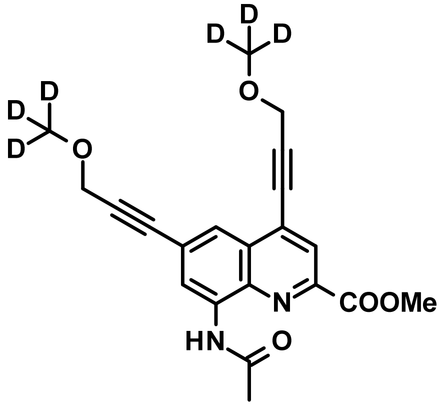

Using **General Procedure 5A** and 300 mg **methyl 8-amino-4,6-bis[3-(trideuteriomethoxy)prop-1-ynyl]quinoline-2-carboxylate (**84% purity, 0.732 mmol, 1.0 equiv), 344 mg **methyl 8-acetamido-4,6-bis[3-(trideuteriomethoxy)prop-1-ynyl]quinoline-2-carboxylate** was obtained as a yellow solid (67% purity, 0.597 mmol, 82%).

^{1}H NMR (400 MHz, [D$_6$]DMSO): δ 9.92 (s, 1H, NH), 8.69 (d, *J* = 1.5 Hz, 1H, Ar-H), 8.22 (s, 1H, Ar-H), 7.94 (d, *J* = 1.5 Hz, 1H, Ar-H), 4.57 (s, 2H, CH$_2$), 4.43 (s, 2H, CH$_2$), 3.99 (s, 3H, C(O)OCH$_3$), 2.31 (s, 3H, C(O)CH$_3$). ^{13}C NMR (100 MHz, [D$_6$]DMSO): δ 169.2, 164.3, 146.2, 137.0, 136.2, 129.7, 128.0, 124.6, 123.6, 121.6, 119.6, 98.4, 89.2, 85.3, 80.5, 59.5, 59.4, 53.1, 24.7. HRMS (ESI): *m/z* [M+H]$^+$ calcd for $C_{21}D_6H_{14}N_2O_5$: 387.1822; found: 387.1826.

methyl 8-acetamido-4,6-bis[3-(trideuteriomethoxy)propyl]quinoline-2-carboxylate (13g)

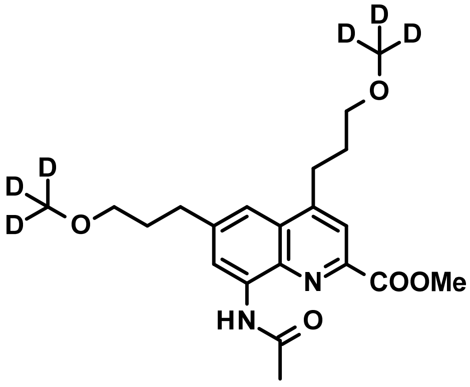

Using **General Procedure 5A** and 300 mg **methyl 8-amino-4,6-bis[3-(trideuteriomethoxy)propyl]quinoline-2-carboxylate** (93% purity, 0.792 mmol, 1.0 equiv.), 301 mg **methyl 8-acetamido-4,6-bis[3-(trideuteriomethoxy)propyl]quinoline-2-carboxylate** was obtained as a yellow oil (92% purity, 0.720 mmol, 89%).

^{1}H NMR (500 MHz, [D$_6$]DMSO): δ 9.85 (s, 1H, NH), 8.55 (d, *J* = 1.5 Hz, 1H, Ar-H), 7.99 (s, 1H, Ar-H), 7.65 (d, *J* = 1.5 Hz, 1H, Ar-H), 3.97 (s, 3H, C(O)OCH$_3$), 3.39 (t, *J* = 6.2 Hz, 2H, CH$_2$), 3.37 (t, *J* = 6.2 Hz, 2H, CH$_2$), 3.16 (t, *J* = 7.7 Hz, 2H, CH$_2$), 2.83 (t, *J* = 7.7 Hz, 2H, CH$_2$), 2.28 (s, 3H, C(O)CH$_3$), 1.97-1.85 (m, 4H, CH$_2$). HRMS (ESI): *m/z* [M+H]$^+$ calcd for $C_{21}D_6H_{22}N_2O_5$: 395.2448; found: 395.2449.

methyl 8-acetamido-3-deuterio-4,6-bis[3-(trideuteriomethoxy)propyl]quinoline-2-carboxylate (13h)

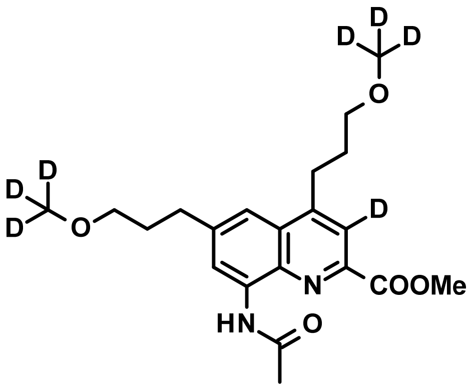

Using **General Procedure 5A** and 300 mg **methyl 8-amino-3-deuterio-4,6-bis[3-(trideuteriomethoxy)propyl]quinoline-2-carboxylate** (87% purity, 0.7383 mmol, 1.0 equiv.), 302 mg **methyl 8-acetamido-3-deuterio-4,6-bis[3-(trideuteriomethoxy)propyl]quinoline-2-carboxylate** was obtained as a yellow oil (90% purity, 0.687 mmol, 93%).

^{1}H NMR (400 MHz, [D$_6$]DMSO): δ 9.86 (s, 1H, NH), 8.55 (d, J = 1.2 Hz, 1H, Ar-H), 7.65 (d, J = 1.2 Hz, 1H, Ar-H), 3.97 (s, 3H, C(O)OCH$_3$), 3.39 (t, J = 6.2 Hz, 2H, CH$_2$), 3.37 (t, J = 6.2 Hz, 2H, CH$_2$), 3.16 (t, J = 7.7 Hz, 2H, CH$_2$), 2.83 (t, J = 7.7 Hz, 2H, CH$_2$), 2.28 (s, 3H, C(O)CH$_3$), 1.97-1.85 (m, 4H, CH$_2$). HRMS (ESI): m/z [M+H]$^+$ calcd for $C_{21}D_7H_{21}N_2O_5$: 396.2510; found: 396.2509.

methyl 8-acetamido-3-deuterio-4,6-bis[3-(trideuteriomethoxy)prop-1-ynyl]quinoline-2-carboxylate (33)

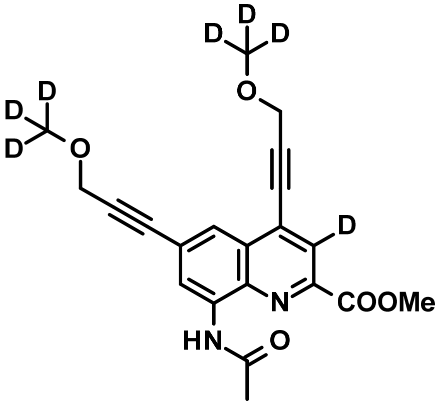

Using **General Procedure 5A** and 330 mg **methyl 8-amino-3-deuterio-4,6-bis[3-(trideuteriomethoxy)prop-1-ynyl]quinoline-2-carboxylate** (82% purity, 0.721 mmol, 1.0 equiv.), 433 mg **methyl 8-acetamido-3-deuterio-4,6-bis[3-(trideuteriomethoxy)prop-1-ynyl]quinoline-2-carboxylate (57% purity, 0.637 mmol, 88%)** as yellow solid.

^{1}H NMR (400 MHz, [D$_6$]DMSO): δ 9.87 (s, 1H, NH), 8.65 (d, J = 1.7 Hz, 1H, Ar-H), 7.87 (d, J = 1.7 Hz, 1H, Ar-H), 4.57 (s, 2H, CH$_2$), 4.42 (s, 2H, CH$_2$), 3.99 (s, 3H, C(O)OCH$_3$), 2.31 (s, 3H, C(O)CH$_3$). ^{13}C NMR (100 MHz, [D$_6$]DMSO): δ 169.2, 164.2, 146.0, 136.9, 136.1, 129.6, 127.9, 123.6, 121.6, 119.4, 98.4, 89.2, 85.3, 80.4, 59.5, 59.4, 53.1, 24.7. HRMS (ESI): m/z [M+H]$^+$ calcd for $C_{21}D_7H_{13}N_2O_5$: 388.1884; found: 388.1884.

methyl 8-acetamido-5,7-dideuterio-4,6-bis[3-(trideuteriomethoxy)prop-1-ynyl]quinoline-2-carboxylate (34)

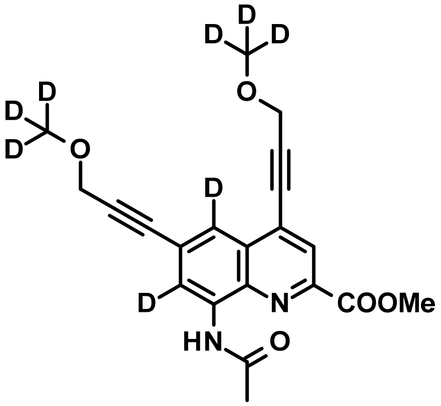

Using **General Procedure 5A** and 300 mg **methyl 8-amino-5,7-dideuterio-4,6-bis[3-(trideuteriomethoxy)prop-1-ynyl]quinoline-2-carboxylate** (72% purity, 0.624 mmol, 1.0 equiv.), 300 mg **methyl 8-acetamido-5,7-dideuterio-4,6-bis[3-(trideuteriomethoxy)prop-1-ynyl]quinoline-2-carboxylate** (69% purity, 0.533 mmol, 85%) as yellow solid.

^{1}H NMR (400 MHz, [D$_6$]DMSO): δ 9.87 (s, 1H, NH), 8.17 (s, 1H, Ar-H), 4.57 (s, 2H, CH$_2$), 4.42 (s, 2H, CH$_2$), 3.99 (s, 3H, C(O)OCH$_3$), 2.31 (s, 3H, C(O)CH$_3$). ^{13}C NMR (100 MHz, [D$_6$]DMSO): δ 169.2, 164.2, 146.1, 136.9, 136.0, 129.7, 127.8, 124.6, 123.4, 98.4, 89.3, 85.3, 80.5, 59.5, 59.4, 53.1, 24.7. HRMS (ESI): m/z [M+H]$^+$ calcd for $C_{21}D_8H_{12}N_2O_5$: 389.1947; found: 389.1948.

methyl 8-acetamido-5,7-dideuterio-4,6-bis[3-(trideuteriomethoxy)propyl]quinoline-2-carboxylate (13i)

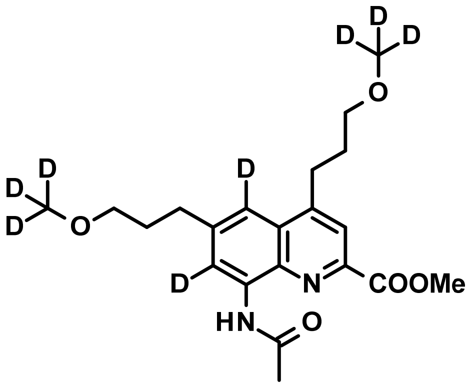

Using **General Procedure 5A** and 270 mg **methyl 8-amino-5,7-dideuterio-4,6-bis[3-(trideuteriomethoxy)propyl]quinoline-2-carboxylate** (84% purity, 0.640 mmol, 1.0 equiv.), 256 mg **methyl 8-acetamido-5,7-dideuterio-4,6-bis[3-(trideuteriomethoxy)propyl]quinoline-2-carboxylate** was obtained as a yellow oil (87% purity, 0.562 mmol, 88%).

^{1}H NMR (400 MHz, [D$_6$]DMSO): δ 9.86 (s, 1H, NH), 8.00 (s, 1H, Ar-H), 3.97 (s, 3H, C(O)OCH$_3$), 3.39 (t, *J* = 6.4 Hz, 2H, CH$_2$), 3.37 (t, *J* = 6.4 Hz, 2H, CH$_2$), 3.16 (t, *J* = 7.7 Hz, 2H, CH$_2$), 2.83 (t, *J* = 7.7 Hz, 2H, CH$_2$), 2.28 (s, 3H, C(O)CH$_3$), 1.97-1.85 (m, 4H, CH$_2$). HRMS (ESI): *m/z* [M+H]$^+$ calcd for $C_{21}D_8H_{20}N_2O_5$: 397.2573; found: 397.2573.

methyl 8-acetamido-4,6-bis(1,1,2,2-tetradeuterio-3-methoxy-propyl)quinoline-2-carboxylate (35)

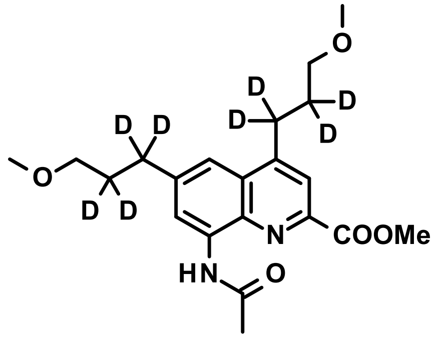

Using **General Procedure 5A** and 284 mg **methyl 8-amino-4,6-bis(1,1,2,2-tetradeuterio-3-methoxy-propyl)quinoline-2-carboxylate** (88% purity, 0.705 mmol, 1.0 equiv.), 271 mg **methyl 8-acetamido-4,6-bis(1,1,2,2-tetradeuterio-3-methoxy-propyl)quinoline-2-carboxylate** was obtained as a yellow oil (94% purity, 0.643 mmol, 91%).

^{1}H NMR (500 MHz, [D$_6$]DMSO): δ 9.86 (s, 1H, NH), 8.55 (d, *J* = 1.5 Hz, 1H, Ar-H), 7.99 (s, 1H, Ar-H), 7.64 (d, *J* = 1.5 Hz, 1H, Ar-H), 3.97 (s, 3H, C(O)OCH$_3$), 3.38 (s, 2H, CH$_2$), 3.36 (s, 2H, CH$_2$), 3.26 (s, 3H, OCH$_3$), 3.25 (s, 3H, OCH$_3$), 2.28 (s, 3H, C(O)CH$_3$). HRMS (ESI): *m/z* [M+H]$^+$ calcd for $C_{21}D_8H_{20}N_2O_5$: 397.2573; found: 397.2577.

methyl 8-[(2,2,2-trideuterioacetyl)amino]-4,6-bis[3-(trideuteriomethoxy)propyl]quinoline-2-carboxylate (13j)

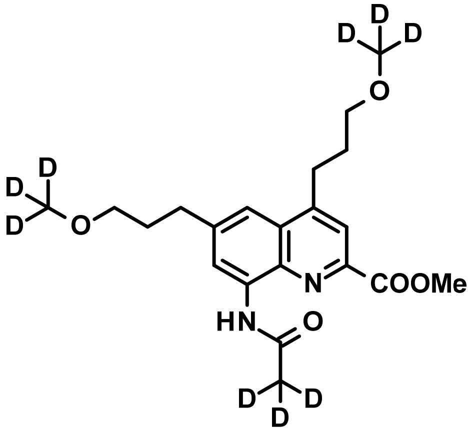

Using **General Procedure 5B** and 300 mg **methyl 8-amino-4,6-bis[3-(trideuteriomethoxy)propyl]quinoline-2-carboxylate** (93% purity, 0.792 mmol, 1.0 equiv.), 303 mg **methyl 8-[(2,2,2-trideuterioacetyl)amino]-4,6-bis[3-(trideuteriomethoxy)propyl]quinoline-2-carboxylate** was obtained as a yellow oil (92% purity, 0.701 mmol, 89%).

^{1}H NMR (500 MHz, [D$_6$]DMSO): δ 9.86 (s, 1H, NH), 8.56 (d, *J* = 1.2 Hz, 1H, Ar-H), 8.00 (s, 1H, Ar-H), 7.66 (d, *J* = 1.2 Hz, 1H, Ar-H), 3.97 (s, 3H, C(O)OCH$_3$), 3.39 (t, *J* = 6.1 Hz, 2H, CH$_2$), 3.37 (t, *J* = 6.1 Hz, 2H, CH$_2$), 3.1 (t, *J* = 7.3 Hz, 2H, CH$_2$), 2.83 (t, *J* = 7.3 Hz, 2H, CH$_2$), 1.97-1.85 (m, 4H, CH$_2$). HRMS (ESI): *m/z* [M+H]$^+$ calcd for $C_{21}D_9H_{19}N_2O_5$: 398.2636; found: 398.2637.

methyl 8-acetamido-3-deuterio-4,6-bis(1,1,2,2-tetradeuterio-3-methoxy-propyl)quinoline-2-carboxylate (36)

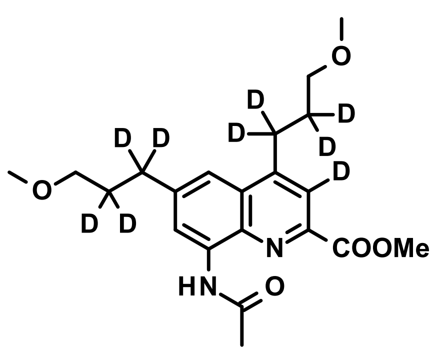

Using **General Procedure 5A** and 350 mg **methyl 8-amino-3-deuterio-4,6-bis(1,1,2,2-tetradeuterio-3-methoxy-propyl)quinoline-2-carboxylate** (91% purity, 0.896 mmol, 1.0 equiv.), 372 mg **methyl 8-acetamido-3-deuterio-4,6-bis(1,1,2,2-tetradeuterio-3-methoxy-propyl)quinoline-2-carboxylate** was obtained as a yellow oil (92% purity, 0.861 mmol, 96%).

^{1}H NMR (400 MHz, [D$_6$]DMSO): δ 9.86 (s, 1H, NH), 8.56 (d, *J* = 1.4 Hz, 1H, Ar-H), 7.65 (d, *J* = 1.4 Hz, 1H, Ar-H), 3.97 (s, 3H, C(O)OCH$_3$), 3.38 (s, 2H, CH$_2$), 3.36 (s, 2H, CH$_2$), 3.26 (s, 3H, OCH$_3$), 3.25 (s, 3H, OCH$_3$), 2.28 (s, 3H, C(O)CH$_3$). HRMS (ESI): *m/z* [M+H]$^+$ calcd for $C_{21}D_9H_{19}N_2O_5$: 398.2636; found: 398.2637.

methyl 3-deuterio-8-[(2,2,2-trideuterioacetyl)amino]-4,6-bis[3-(trideuteriomethoxy)prop-1-ynyl]quinoline-2-carboxylate (37)

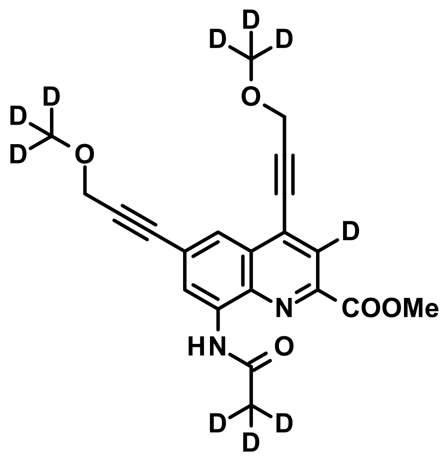

Using **General Procedure 5B** and 300 mg **methyl 8-amino-4,6-bis[3-(trideuteriomethoxy)prop-1-ynyl]quinoline-2-carboxylate** (87% purity, 0.756 mmol, 1.0 equiv.), 198 mg **methyl 3-deuterio-8-[(2,2,2-trideuterioacetyl)amino]-4,6-bis[3-(trideuteriomethoxy)prop-1-ynyl]quinoline-2-carboxylate** was obtained as a yellow solid (79% purity, 0.401 mmol, 53%).

^{1}H NMR (500 MHz, [D$_6$]DMSO): δ 9.87 (s, 1H, NH), 8.66 (d, *J* = 1.7 Hz, 1H, Ar-H), 7.88 (d, J = 1.7 Hz, 1H, Ar-H), 4.57 (s, 2H, CH$_2$), 4.42 (s, 2H, CH$_2$), 3.99 (s, 3H, C(O)OCH$_3$). ^{13}C NMR (125 MHz, [D$_6$]DMSO): δ 169.3, 164.2, 146.0, 136.9, 136.1, 129.6, 127.9, 123.6, 121.6, 119.4, 98.4, 89.2, 85.3, 80.4, 59.5, 59.4, 53.1. HRMS (ESI): *m/z* [M+H]$^+$ calcd for $C_{21}D_{10}H_{10}N_2O_5$: 391.2073; found: 391.2073.

methyl 3-deuterio-8-[(2,2,2-trideuterioacetyl)amino]-4,6-bis[3-(trideuteriomethoxy)propyl]quinoline-2-carboxylate (13k)

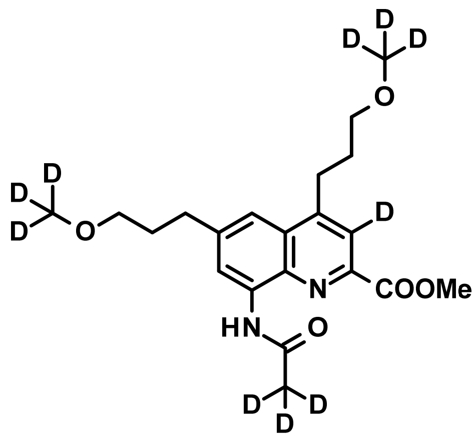

Using **General Procedure 5B** and 210 mg **methyl 8-amino-3-deuterio-4,6-bis[3-(trideuteriomethoxy)propyl]quinoline-2-carboxylate** (87% purity, 0.517 mmol, 1.0 equiv.), 214 mg **methyl 3-deuterio-8-[(2,2,2-trideuterioacetyl)amino]-4,6-bis[3-(trideuteriomethoxy)propyl]quinoline-2-carboxylate** was obtained as a yellow oil (82% purity, 0.440 mmol, 85%).

^{1}H NMR (400 MHz, [D$_6$]DMSO): δ 9.86 (s, 1H, NH), 8.56 (d, *J* = 1.2 Hz, 1H, Ar-H), 7.65 (d, *J* = 1.2 Hz, 1H, Ar-H), 3.97 (s, 3H, C(O)OCH$_3$), 3.39 (t, *J* = 6.3 Hz, 2H, CH$_2$), 3.37 (t, *J* = 6.3 Hz, 2H, CH$_2$), 3.10 (t, *J* = 7.8 Hz, 2H, CH$_2$), 2.83 (t, *J* = 7.8 Hz, 2H, CH$_2$), 1.97-1.85 (m, 4H, CH$_2$). HRMS (ESI): *m/z* [M+H]$^+$ calcd for $C_{21}D_{10}H_{18}N_2O_5$: 399.2699; found: 399.2701.

methyl 5,7-dideuterio-8-[(2,2,2-trideuterioacetyl)amino]-4,6-bis[3-(trideuteriomethoxy)propyl]quinoline-2-carboxylate (13l)

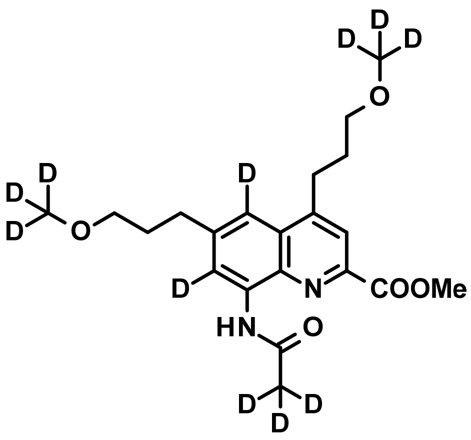

Using **General Procedure 5B** and 161 mg **methyl 8-amino-5,7-dideuterio-4,6-bis[3-(trideuteriomethoxy)propyl]quinoline-2-carboxylate** (84% purity, 0.382 mmol, 1.0 equiv.), 172 mg **methyl 5,7-dideuterio-8-[(2,2,2-trideuterioacetyl)amino]-4,6-bis[3-(trideuteriomethoxy)propyl]quinoline-2-carboxylate** was obtained as a yellow oil (77% purity, 0.332 mmol, 87%).

^{1}H NMR (400 MHz, [D$_6$]DMSO): δ 9.86 (s, 1H, NH), 8.00 (s, 1H, Ar-H), 3.97 (s, 3H, C(O)OCH$_3$), 3.39 (t, *J* = 6.2 Hz, 2H, CH$_2$), 3.37 (t, *J* = 6.2 Hz, 2H, CH$_2$), 3.15 (t, *J* = 7.7 Hz, 2H, CH$_2$), 2.83 (t, *J* = 7.7 Hz, 2H, CH$_2$), 1.97-1.85 (m, 4H, CH$_2$). HRMS (ESI): *m/z* [M+H]$^+$ calcd for $C_{21}D_{11}H_{17}N_2O_5$: 400.2761; found: 400.2761.

methyl 3-deuterio-4,6-bis(1,1,2,2-tetradeuterio-3-methoxy-propyl)-8-[(2,2,2-trideuterioacetyl)amino]quinoline-2-carboxylate (13m)

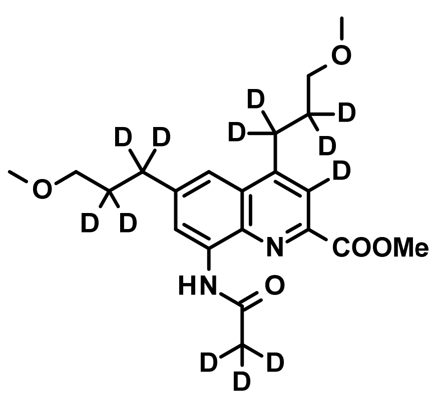

Using **General Procedure 5B** and 165 mg **methyl 8-amino-3-deuterio-4,6-bis(1,1,2,2-tetradeuterio-3-methoxy-propyl)quinoline-2-carboxylate** (82% purity, 0.381 mmol, 1.0 equiv.), 167 mg **methyl 3-deuterio-4,6-bis(1,1,2,2-tetradeuterio-3-methoxy-propyl)-8-[(2,2,2-trideuterioacetyl)amino]quinoline-2-carboxylate** was obtained as a yellow oil (81% purity, 0.338 mmol, 89%).

^{1}H NMR (400 MHz, [D$_6$]DMSO): δ 9.86 (s, 1H, NH), 8.56 (d, *J* = 1.6 Hz, 1H, Ar-H), 7.65 (d, *J* = 1.6 Hz, 1H, Ar-H), 3.97 (s, 3H, C(O)OCH$_3$), 3.38 (s, 2H, CH$_2$), 3.36 (s, 2H, CH$_2$), 3.26 (s, 3H, OCH$_3$), 3.25 (s, 3H, OCH$_3$). HRMS (ESI): *m/z* [M+H]$^+$ calcd for $C_{21}D_{12}H_{16}N_2O_5$: 401.2824; found: 401.2822.

methyl 5,7-dideuterio-4,6-bis(1,1,2,2-tetradeuterio-3-methoxy-propyl)-8-[(2,2,2-trideuterioacetyl)amino]quinoline-2-carboxylate (13n)

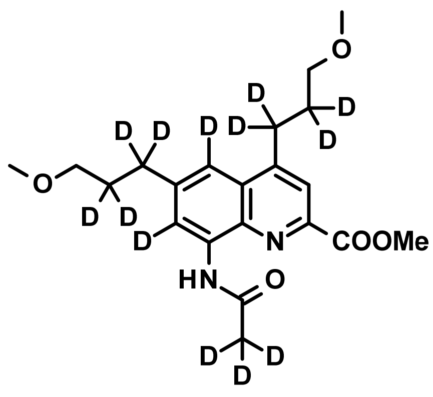

Using **General Procedure 5B** and 175 mg **methyl 8-amino-5,7-dideuterio-4,6-bis(1,1,2,2-tetradeuterio-3-methoxy-propyl)quinoline-2-carboxylate** (79% purity, 0.388 mmol, 1.0 equiv.), 188 mg **methyl 5,7-dideuterio-4,6-bis(1,1,2,2-tetradeuterio-3-methoxy-propyl)-8-[(2,2,2-trideuterioacetyl)amino]quinoline-2-carboxylate** was obtained as a yellow oil (70% purity, 0.328 mmol, 85%).

^{1}H NMR (500 MHz, [D$_6$]DMSO): δ 9.86 (s, 1H, NH), 8.00 (s, 1H, Ar-H), 3.97 (s, 3H, C(O)OCH$_3$), 3.38 (s, 2H, CH$_2$), 3.36 (s, 2H, CH$_2$), 3.26 (s, 3H, OCH$_3$), 3.25 (s, 3H, OCH$_3$). HRMS (ESI): *m/z* [M+H]$^+$ calcd for $C_{21}D_{13}H_{15}N_2O_5$: 402.2887; found: 402.2886.

methyl 8-acetamido-4,6-bis[1,1,2,2-tetradeuterio-3-(trideuteriomethoxy)propyl]quinoline-2-carboxylate (13o)

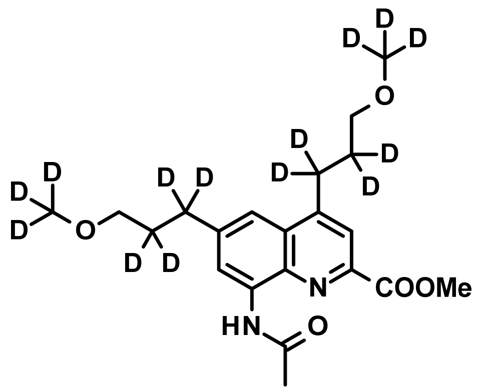

Using **General Procedure 4B** and 320 mg **methyl 8-acetamido-4,6-bis[3-(trideuteriomethoxy)prop-1-ynyl]quinoline-2-carboxylate** (67% purity, 0.555 mmol, 1.0 equiv.), 203 mg **methyl 8-acetamido-4,6-bis[1,1,2,2-tetradeuterio-3-(trideuteriomethoxy)propyl]quinoline-2-carboxylate** was obtained as a yellow oil (87% purity, 0.439 mmol, 79%).

^{1}H NMR (500 MHz, [D$_6$]DMSO): δ 9.86 (s, 1H, NH), 8.55 (d, *J* = 1.6 Hz, 1H, Ar-H), 8.00 (s, 1H, Ar-H), 7.66 (d, *J* = 1.6 Hz, 1H, Ar-H), 3.97 (s, 3H, C(O)OCH$_3$), 3.38 (s, 2H, CH$_2$), 3.36 (s, 2H, CH$_2$), 2.28 (s, 3H, C(O)CH$_3$). HRMS (ESI): *m/z* [M+H]$^+$ calcd for $C_{21}D_{14}H_{14}N_2O_5$: 403.2950; found: 403.2951.

methyl 8-acetamido-3-deuterio-4,6-bis[1,1,2,2-tetradeuterio-3-(trideuteriomethoxy)propyl]quinoline-2-carboxylate (13p)

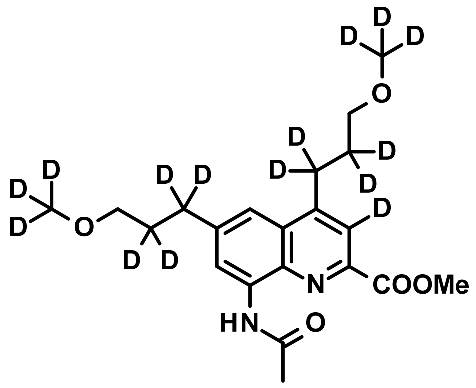

Using **General Procedure 4B** and 300 mg **methyl 8-acetamido-3-deuterio-4,6-bis[3-(trideuteriomethoxy)prop-1-ynyl]quinoline-2-carboxylate** (57% purity, 0.441 mmol, 1.0 equiv.), 202 mg **methyl 8-acetamido-3-deuterio-4,6-bis[1,1,2,2-tetradeuterio-3-(trideuteriomethoxy)propyl]quinoline-2-carboxylate** was obtained as a yellow oil (65% purity, 0.325 mmol, 74%).

^{1}H NMR (400 MHz, [D$_6$]DMSO): δ 9.86 (s, 1H, NH), 8.55 (d, *J* = 1.7 Hz, 1H, Ar-H), 7.65 (d, *J* = 1.7 Hz, 1H, Ar-H), 3.97 (s, 3H, C(O)OCH$_3$), 3.38 (s, 2H, CH$_2$), 3.36 (s, 2H, CH$_2$), 2.28 (s, 3H, C(O)CH$_3$). HRMS (ESI): *m/z* [M+H]$^+$ calcd for $C_{21}D_{15}H_{13}N_2O_5$: 404.3012; found: 404.3014.

methyl 8-acetamido-5,7-dideuterio-4,6-bis[1,1,2,2-tetradeuterio-3-(trideuteriomethoxy)propyl]quinoline-2-carboxylate (13q)

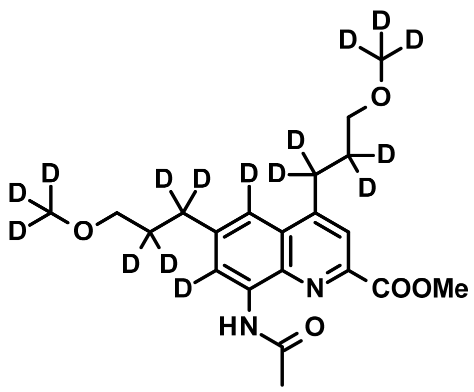

Using **General Procedure 4B** and 280 mg **methyl 8-acetamido-5,7-dideuterio-4,6-bis[3-(trideuteriomethoxy)prop-1-ynyl]quinoline-2-carboxylate** (69% purity, 0.497 mmol, 1.0 equiv.), 186 mg **methyl 8-acetamido-5,7-dideuterio-4,6-bis[1,1,2,2-tetradeuterio-3-(trideuteriomethoxy)propyl]quinoline-2-carboxylate** was obtained as a yellow oil (74% purity, 0.340 mmol, 68%).

^{1}H NMR (400 MHz, [D$_6$]DMSO): δ 9.86 (s, 1H, NH), 7.99 (s, 1H, Ar-H), 3.97 (s, 3H, C(O)OCH$_3$), 3.38 (s, 2H, CH$_2$), 3.36 (s, 2H, CH$_2$), 2.28 (s, 3H, C(O)CH$_3$). HRMS (ESI): *m/z* [M+H]$^+$ calcd for $C_{21}D_{16}H_{12}N_2O_5$: 405.3075; found: 405.3080.

methyl 4,6-bis[1,1,2,2-tetradeuterio-3-(trideuteriomethoxy)propyl]-8-[(2,2,2-trideuterioacetyl)amino]quinoline-2-carboxylate (13r)

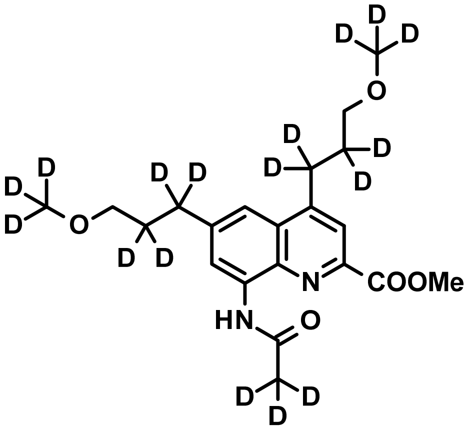

Using **General Procedure 5B** and 114 mg **methyl 8-amino-4,6-bis[1,1,2,2-tetradeuterio-3-(trideuteriomethoxy)propyl]quinoline-2-carboxylate** (88% purity, 0.227 mmol, 1.0 equiv.), 94 mg **methyl 4,6-bis[1,1,2,2-tetradeuterio-3-(trideuteriomethoxy)propyl]-8-[(2,2,2-trideuterioacetyl)amino]quinoline-2-carboxylate** was obtained as a yellow oil (89% purity, 0.206 mmol, 74%).

^{1}H NMR (500 MHz, [D$_6$]DMSO): δ 9.86 (s, 1H, NH), 8.56 (d, *J* = 1.5 Hz, 1H, Ar-H), 8.00 (s, 1H, Ar-H), 7.66 (d, *J* = 1.5 Hz, 1H, Ar-H), 3.97 (s, 3H, C(O)OCH$_3$), 3.38 (s, 2H, CH$_2$), 3.36 (s, 2H, CH$_2$). HRMS (ESI): *m/z* [M+H]$^+$ calcd for $C_{21}D_{17}H_{11}N_2O_5$: 406.3138; found: 406.3138.

methyl 3-deuterio-4,6-bis[1,1,2,2-tetradeuterio-3-(trideuteriomethoxy)propyl]-8-[(2,2,2-trideuterioacetyl)amino]quinoline-2-carboxylate (13s)

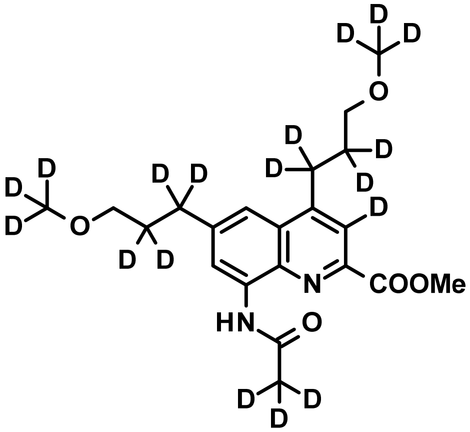

Using **General Procedure 4B** and 100 mg **methyl 3-deuterio-8-[(2,2,2-trideuterioacetyl)amino]-4,6-bis[3-(trideuteriomethoxy)prop-1-ynyl]quinoline-2-carboxylate** (79% purity, 0.497 mmol, 1.0 equiv.), 98 mg **methyl 3-deuterio-4,6-bis[1,1,2,2-tetradeuterio-3-(trideuteriomethoxy)propyl]-8-[(2,2,2-trideuterioacetyl)amino]quinoline-2-carboxylate** was obtained as a yellow oil (81% purity, 0.340 mmol, 97%).

^{1}H NMR (500 MHz, [D$_6$]DMSO): δ 9.86 (s, 1H, NH), 8.55 (d, *J* = 1.7 Hz, 1H, Ar-H), 7.64 (d, *J* = 1.7 Hz, 1H, Ar-H), 3.97 (s, 3H, C(O)OCH$_3$), 3.38 (s, 2H, CH$_2$), 3.35 (s, 2H, CH$_2$). HRMS (ESI): *m/z* [M+H]$^+$ calcd for $C_{21}D_{18}H_{10}N_2O_5$: 407.3201; found: 407.3202.

methyl 5,7-dideuterio-4,6-bis[1,1,2,2-tetradeuterio-3-(trideuteriomethoxy)propyl]-8-[(2,2,2-trideuterioacetyl)amino]quinoline-2-carboxylate (13t)

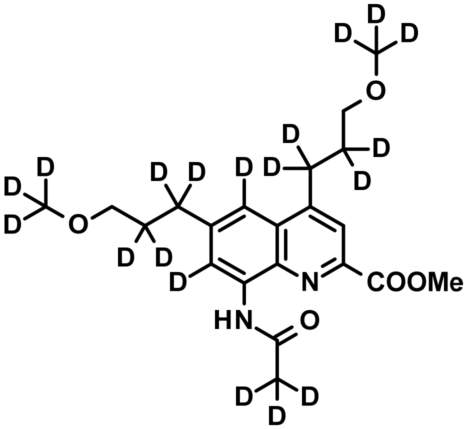

Using **General Procedure 5B** and 150 mg **methyl 8-amino-5,7-dideuterio-4,6-bis[1,1,2,2-tetradeuterio-3-(trideuteriomethoxy)propyl]quinoline-2-carboxylate** (88% purity, 0.364 mmol, 1.0 equiv.), 190 mg **methyl 5,7-dideuterio-4,6-bis[1,1,2,2-tetradeuterio-3-(trideuteriomethoxy)propyl]-8-[(2,2,2-trideuterioacetyl)amino]quinoline-2-carboxylate** was obtained as a yellow oil (70% purity, 0.326 mmol, 90%).

^{1}H NMR (400 MHz, [D$_6$]DMSO): δ 9.87 (s, 1H, NH), 8.00 (s, 1H, Ar-H), 3.97 (s, 3H, C(O)OCH$_3$), 3.38 (s, 2H, CH$_2$), 3.36 (s, 2H, CH$_2$). HRMS (ESI): *m/z* [M+H]$^+$ calcd for $C_{21}D_{19}H_9N_2O_5$: 408.3264; found: 408.3262.

methyl 3,5,7-trideuterio-4,6-bis[1,1,2,2-tetradeuterio-3-(trideuteriomethoxy)propyl]-8-[(2,2,2-trideuterioacetyl)amino]quinoline-2-carboxylate (13u)

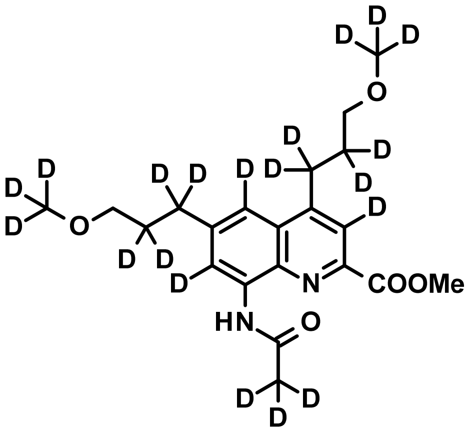

Using **General Procedure 5B** and 175 mg **methyl 8-amino-3,5,7-trideuterio-4,6-bis[1,1,2,2-tetradeuterio-3-(trideuteriomethoxy)propyl]quinoline-2-carboxylate** (80% purity, 0.385 mmol, 1.0 equiv.), 152 mg **methyl 3,5,7-trideuterio-4,6-bis[1,1,2,2-tetradeuterio-3-(trideuteriomethoxy)propyl]-8-[(2,2,2-trideuterioacetyl)amino]quinoline-2-carboxylate** was obtained as a yellow oil (92% purity, 0.342 mmol, 89%).

^{1}H NMR (400 MHz, [D$_6$]DMSO): δ 9.87 (s, 1H, NH), 3.97 (s, 3H, C(O)OCH$_3$), 3.38 (s, 2H, CH$_2$), 3.36 (s, 2H, CH$_2$). HRMS (ESI): *m/z* [M+H]$^+$ calcd for $C_{21}D_{20}H_8N_2O_5$: 409.3326; found: 409.3327.

methyl 4,6-bis[1,1,2,2,3,3-hexadeuterio-3-(trideuteriomethoxy)propyl]-8-[(2,2,2-trideuterioacetyl)amino]quinoline-2-carboxylate (13v)

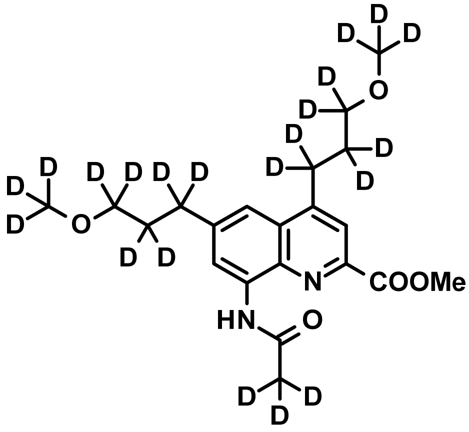

Using **General Procedure 5B** and 320 mg **methyl 8-amino-4,6-bis[1,1,2,2,3,3-hexadeuterio-3-(trideuteriomethoxy)propyl]quinoline-2-carboxylate** (55% purity, 0.483 mmol, 1.0 equiv.), 299 mg **methyl 4,6-bis[1,1,2,2,3,3-hexadeuterio-3-(trideuteriomethoxy)propyl]-8-[(2,2,2-trideuterioacetyl)amino]quinoline-2-carboxylate** was obtained as a yellow oil (63% purity, 0.460 mmol, 95%).

^{1}H NMR (500 MHz, [D$_6$]DMSO): δ 9.87 (s, 1H, NH), 8.56 (d, *J* = 1.7 Hz, 1H, Ar-H), 8.00 (s, 1H, Ar-H), 7.66 (d, *J* = 1.7 Hz, 1H, Ar-H), 3.97 (s, 3H, C(O)OCH$_3$). HRMS (ESI): *m/z* [M+H]$^+$ calcd for $C_{21}D_{21}H_7N_2O_5$: 410.3389; found: 410.3391.

methyl 3-deuterio-4,6-bis[1,1,2,2,3,3-hexadeuterio-3-(trideuteriomethoxy)propyl]-8-[(2,2,2-trideuterioacetyl)amino]quinoline-2-carboxylate (13w)

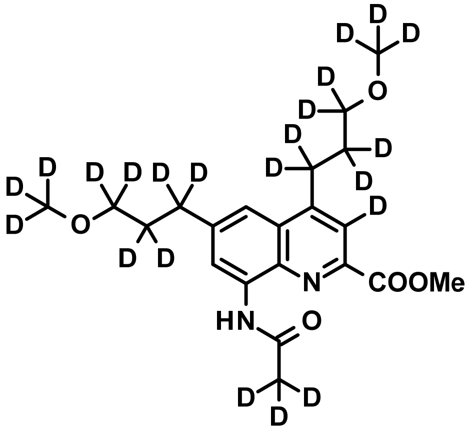

Using **General Procedure 5B** and 290 mg **methyl 8-amino-3-deuterio-4,6-bis[1,1,2,2,3,3-hexadeuterio-3-(trideuteriomethoxy)propyl]quinoline-2-carboxylate** (52% purity, 0.413 mmol, 1.0 equiv.), 274 mg **methyl 3-deuterio-4,6-bis[1,1,2,2,3,3-hexadeuterio-3-(trideuteriomethoxy)propyl]-8-[(2,2,2-trideuterioacetyl)amino]quinoline-2-carboxylate** was obtained as a yellow oil (51% purity, 0.340 mmol, 82%).

^{1}H NMR (500 MHz, [D$_6$]DMSO): δ 9.87 (s, 1H, NH), 8.56 (d, *J* = 1.6 Hz, 1H, Ar-H), 7.66 (d, *J* = 1.6 Hz, 1H, Ar-H), 3.97 (s, 3H, C(O)OCH$_3$). HRMS (ESI): *m/z* [M+H]$^+$ calcd for $C_{21}D_{22}H_6N_2O_5$: 411.3452; found: 411.3450.

methyl 5,7-dideuterio-4,6-bis[1,1,2,2,3,3-hexadeuterio-3-(trideuteriomethoxy)propyl]-8-[(2,2,2-trideuterioacetyl)amino]quinoline-2-carboxylate (13x)

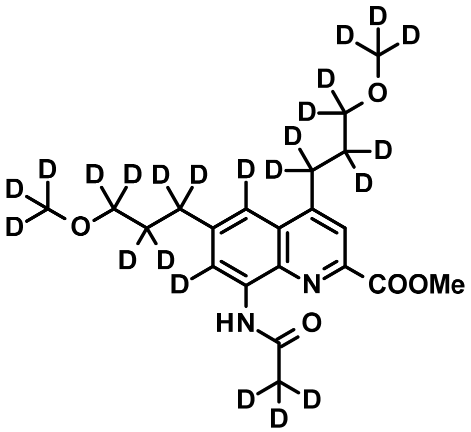

Using **General Procedure 5B** and 230 mg **methyl 8-amino-5,7-dideuterio-4,6-bis[1,1,2,2,3,3-hexadeuterio-3-(trideuteriomethoxy)propyl]quinoline-2-carboxylate** (70% purity, 0.439 mmol, 1.0 equiv.), 222 mg **methyl 5,7-dideuterio-4,6-bis[1,1,2,2,3,3-hexadeuterio-3-(trideuteriomethoxy)propyl]-8-[(2,2,2-trideuterioacetyl)amino]quinoline-2-carboxylate** was obtained as a yellow oil (70% purity, 0.378 mmol, 86%).

^{1}H NMR (500 MHz, [D$_6$]DMSO): 9.87 (s, 1H, NH), 8.00 (s, 1H, Ar-H), 3.97 (s, 3H, C(O)OCH$_3$). HRMS (ESI): *m/z* [M+H]$^+$ calcd for $C_{21}D_{23}H_5N_2O_5$: 412.3515; found: 412.3516.

methyl 3,5,7-trideuterio-4,6-bis[1,1,2,2,3,3-hexadeuterio-3-(trideuteriomethoxy)propyl]-8-[(2,2,2-trideuterioacetyl)amino]quinoline-2-carboxylate (13y)

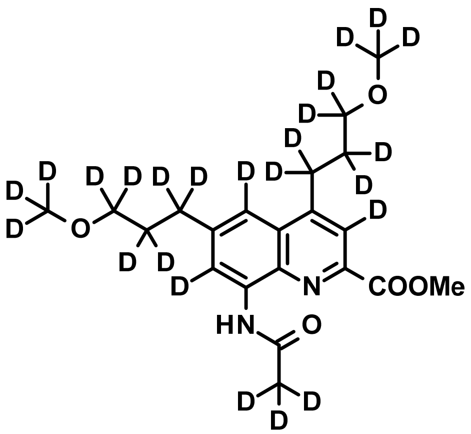

Using **General Procedure 5B** and 220 mg **methyl 8-amino-3,5,7-trideuterio-4,6-bis[1,1,2,2,3,3-hexadeuterio-3-(trideuteriomethoxy)propyl]quinoline-2-carboxylate** (61% purity, 0.365 mmol, 1.0 equiv.), 197 mg **methyl 3,5,7-trideuterio-4,6-bis[1,1,2,2,3,3-hexadeuterio-3-(trideuteriomethoxy)propyl]-8-[(2,2,2-trideuterioacetyl)amino]quinoline-2-carboxylate** was obtained as a yellow oil (62% purity, 0.296 mmol, 81%).

^{1}H NMR (500 MHz, [D$_6$]DMSO): 9.86 (s, 1H, NH), 3.97 (s, 3H, C(O)OCH$_3$). HRMS (ESI): *m/z* [M+H]$^+$ calcd for $C_{21}D_{24}H_4N_2O_5$: 413.3577; found: 413.3578.

8-acetamido-4,6-bis(3-methoxypropyl)quinoline-2-carboxylic acid (1a)

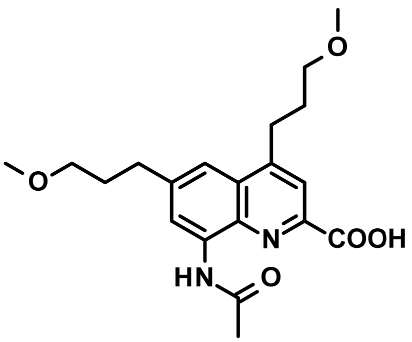

Using **General Procedure 6** and 1.200 g **methyl 8-amino-4,6-bis(3-methoxypropyl)quinoline-2-carboxylate** (3.089 mmol, 1.0 equiv.), 1.130 g **8-acetamido-4,6-bis(3-methoxypropyl)quinoline-2-carboxylic acid** was obtained as a yellow oil (2.976 mmol, 96%).

HPLC-UV purity: 98.6%.

^{1}H NMR (500 MHz, [D$_6$]DMSO): δ 13.24 (br s, 1H, COOH), 10.46 (s, 1H, NH), 8.70 (d, J = 1.4 Hz, 1H, Ar-H), 8.02 (s, 1H, Ar-H), 7.64 (d, J = 1.4 Hz, 1H, Ar-H), 3.39 (t, J = 6.3 Hz, 2H, CH$_2$), 3.36 (t, J = 6.3 Hz, 2H, CH$_2$), 3.26 (s, 3H, OCH$_3$), 3.25 (s, 3H, OCH$_3$), 3.15 (t, J = 7.6 Hz, 2H, CH$_2$), 2.83 (t, J = 7.6 Hz, 2H, CH$_2$), 2.32 (s, 3H, C(O)CH$_3$), 1.97-1.84 (m, 4H, CH$_2$). ^{13}C NMR (125 MHz, [D$_6$]DMSO): δ 169.3, 165.5, 150.1, 144.1, 143.4, 136.0, 135.6, 128.3, 120.1, 118.3, 116.0, 70.98, 70.97, 57.91, 57.90, 32.8, 30.7, 29.4, 28.2, 24.8. HRMS (ESI): *m/z* [M+H]$^+$ calcd for $C_{20}H_{26}N_2O_5$: 375.1914; found: 375.1916.

8-acetamido-3-deuterio-4,6-bis(3-methoxypropyl)quinoline-2-carboxylic acid (1b)

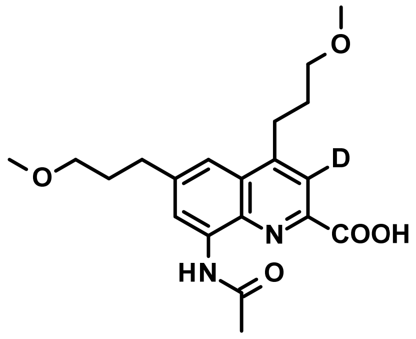

Using **General Procedure 6** and 550 mg **methyl 8-acetamido-3-deuterio-4,6-bis(3-methoxypropyl)quinoline-2-carboxylate** (1.412 mmol, 1.0 equiv.), 294 mg **8-acetamido-3-deuterio-4,6-bis(3-methoxypropyl)quinoline-2-carboxylic acid** was obtained as a yellow oil (0.763 mmol, 54%).

HPLC-UV purity: 97.4%.

^{1}H NMR (500 MHz, [D$_6$]DMSO): δ 10.42 (s, 1H, NH), 8.56 (d, *J* = 1.4 Hz, 1H, Ar-H), 7.54 (d, *J* = 1.4 Hz, 1H, Ar-H), 3.38 (t, *J* = 6.3 Hz, 2H, CH$_2$), 3.36 (t, *J* = 6.3 Hz, 2H, CH$_2$), 3.25 (s, 6H, OCH$_3$), 3.07 (t, *J* = 7.6 Hz, 2H, CH$_2$), 2.80 (t, *J* = 7.6 Hz, 2H, CH$_2$), 2.28 (s, 3H, C(O)CH$_3$), 1.97-1.84 (m, 4H, CH$_2$). ^{13}C NMR (125 MHz, [D$_6$]DMSO): δ 168.9, 167.8, 151.3, 147.9, 141.3, 135.7, 127.0, 116.9, 115.7, 71.06, 71.03, 57.90, 57.89, 32.8, 30.8, 29.4, 28.0, 24.8. HRMS (ESI): *m/z* [M+H]$^+$ calcd for $C_{20}DH_{25}N_2O_5$: 376.1977; found: 376.1977.

8-acetamido-5,7-dideuterio-4,6-bis(3-methoxypropyl)quinoline-2-carboxylic acid (1c)

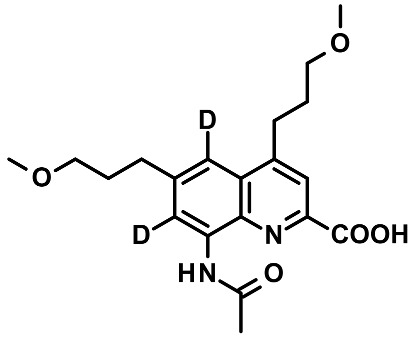

Using **General Procedure 6** and 170 mg **methyl 8-acetamido-5,7-dideuterio-4,6-bis(3-methoxypropyl)quinoline-2-carboxylate** (0.436 mmol, 1.0 equiv.), 130 mg **8-acetamido-5,7-dideuterio-4,6-bis(3-methoxypropyl)quinoline-2-carboxylic acid** was obtained as a yellow oil (0.339 mmol, 78%).

HPLC-UV purity: 98.2%.

^{1}H NMR (500 MHz, [D$_6$]DMSO): δ 13.28 (br s, 1H, COOH), 10.47 (s, 1H, NH), 8.04 (s, 1H, Ar-H), 3.39 (t, *J* = 6.3 Hz, 2H, CH$_2$), 3.37 (t, *J* = 6.3 Hz, 2H, CH$_2$), 3.26 (s, 3H, OCH$_3$), 3.25 (s, 3H, OCH$_3$), 3.17 (t, *J* = 7.6 Hz, 2H, CH$_2$), 2.83 (t, *J* = 7.6 Hz, 2H, CH$_2$), 2.32 (s, 3H, C(O)CH$_3$), 1.97-1.84 (m, 4H, CH$_2$). ^{13}C NMR (125 MHz, [D$_6$]DMSO): δ 169.2, 165.5, 150.1, 144.1, 143.2, 135.9, 135.6, 128.2, 120.1, 70.98, 70.96, 57.92, 57.90, 32.7, 30.7, 29.4, 28.2, 24.8. HRMS (ESI): *m/z* [M+H]$^+$ calcd for $C_{20}D_2H_{24}N_2O_5$: 377.2040; found: 377.2042.

8-acetamido-3,5,7-trideuterio-4,6-bis(3-methoxypropyl)quinoline-2-carboxylic acid (1d)

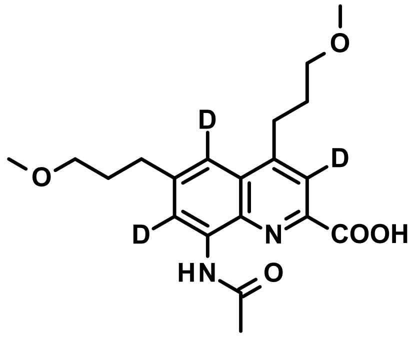

Using **General Procedure 6** and 165 mg **methyl 8-acetamido-3,5,7-trideuterio-4,6-bis(3-methoxypropyl)quinoline-2-carboxylate** (63% purity, 0.266 mmol, 1.0 equiv.), 31 mg **8-acetamido-3,5,7-trideuterio-4,6-bis(3-methoxypropyl)quinoline-2-carboxylic acid** was obtained as a yellow oil (0.082 mmol, 31%).

HPLC-UV purity: 99.6%.

^{1}H NMR (500 MHz, [D$_6$]DMSO): δ 13.27 (br s, 1H, COOH), 10.47 (s, 1H, NH), 3.39 (t, *J* = 6.3 Hz, 2H, CH$_2$), 3.37 (t, *J* = 6.3 Hz, 2H, CH$_2$), 3.26 (s, 3H, OCH$_3$), 3.25 (s, 3H, OCH$_3$), 3.16 (t, *J* = 7.6 Hz, 2H, CH$_2$), 2.83 (t, *J* = 7.6 Hz, 2H, CH$_2$), 2.32 (s, 3H, C(O)CH$_3$), 1.97-1.84 (m, 4H, CH$_2$). ^{13}C NMR (125 MHz, [D$_6$]DMSO): δ 169.2, 165.6, 150.0, 144.2, 143.2, 135.9, 135.6, 128.2, 70.98, 70.97, 57.92, 57.90, 32.7, 30.7, 29.3, 28.2, 24.8. HRMS (ESI): *m/z* [M+H]$^+$ calcd for $C_{20}D_3H_{23}N_2O_5$: 378.2103; found: 378.2106.

4,6-bis(3-methoxypropyl)-8-[(2,2,2-trideuterioacetyl)amino]quinoline-2-carboxylic acid (1d*)

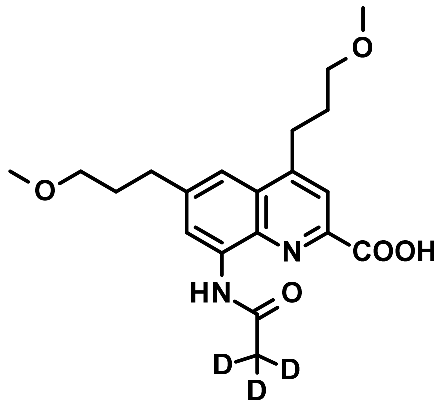

Using **General Procedure 6** and 338 mg **methyl 4,6-bis(3-methoxypropyl)-8-[(2,2,2-trideuterioacetyl)amino]quinoline-2-carboxylate** (74% purity, 0.639 mmol, 1.0 equiv.), 242 mg **4,6-bis(3-methoxypropyl)-8-[(2,2,2-trideuterioacetyl)amino]quinoline-2-carboxylic acid** was obtained as a yellow oil (0.616 mmol, 96%).

HPLC-UV purity: 96.4%.

^{1}H NMR (500 MHz, [D$_6$]DMSO): δ 10.50 (s, 1H, NH), 8.64 (s, 1H, Ar-H), 7.96 (s, 1H, Ar-H), 7.59 (d, J = 1.2 Hz, 1H, Ar-H), 3.38 (t, J = 6.1 Hz, 2H, CH$_2$), 3.37 (t, J = 6.1 Hz, 2H, CH$_2$), 3.26 (s, 3H, OCH$_3$), 3.25 (s, 3H, OCH$_3$), 3.11 (t, J = 7.8 Hz, 2H, CH$_2$), 2.83 (t, J = 7.6 Hz, 2H, CH$_2$), 1.94-1.85 (m, 4H, CH$_2$). ^{13}C NMR (125 MHz, [D$_6$]DMSO): δ 169.1, 166.8, 149.0, 142.4, 135.9, 135.6, 127.6, 120.6, 117.6, 115.8, 71.0, 57.88, 57.86, 32.8, 30.8, 29.4, 28.1, 24.8. HRMS (ESI): m/z [M+H]$^+$ calcd for $C_{20}D_3H_{23}N_2O_5$: 378.2103; found: 378.2111.

3-deuterio-4,6-bis(3-methoxypropyl)-8-[(2,2,2-trideuterioacetyl)amino]quinoline-2-carboxylic acid (1e)

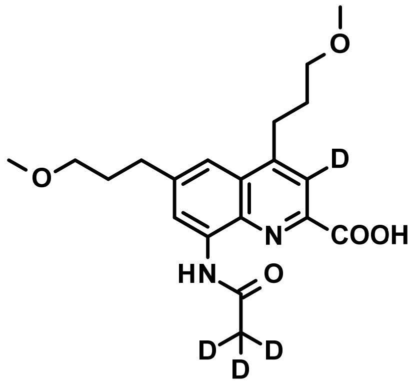

Using **General Procedure 6** and 246 mg (65% purity, 0.408 mmol, 1.0 equiv.) **methyl 3-deuterio-4,6-bis(3-methoxypropyl)-8-[(2,2,2-trideuterioacetyl)amino]quinoline-2-carboxylate**, 49 mg **3-deuterio-4,6-bis(3-methoxypropyl)-8-[(2,2,2-trideuterioacetyl)amino]quinoline-2-carboxylic acid** was obtained as a yellow oil (0.130 mmol, 32%).

HPLC-UV purity: 99.4%.

^{1}H NMR (500 MHz, [D$_6$]DMSO): δ 10.51 (s, 1H, NH), 8.67 (d, J = 1.4 Hz, 1H, Ar-H), 7.62 (d, J = 1.4 Hz, 1H, Ar-H), 3.39 (t, J = 6.3 Hz, 2H, CH$_2$), 3.37 (t, J = 6.3 Hz, 2H, CH$_2$), 3.25 (s, 6H, OCH$_3$), 3.07 (t, J = 7.6 Hz, 2H, CH$_2$), 2.83 (t, J = 7.6 Hz, 2H, CH$_2$), 1.97-1.84 (m, 4H, CH$_2$). ^{13}C NMR (125 MHz, [D$_6$]DMSO): δ 169.3, 166.0, 149.6, 145.4, 143.0, 136.0, 135.6, 128.0, 118.0, 115.9, 70.99, 70.98, 57.91, 57.90, 32.8, 30.8, 29.3, 28.1. HRMS (ESI): m/z [M+H]$^+$ calcd for $C_{20}D_4H_{22}N_2O_5$: 379.2166; found: 379.2166.

5,7-dideuterio-4,6-bis(3-methoxypropyl)-8-[(2,2,2-trideuterioacetyl)amino]quinoline-2-carboxylic acid (1f)

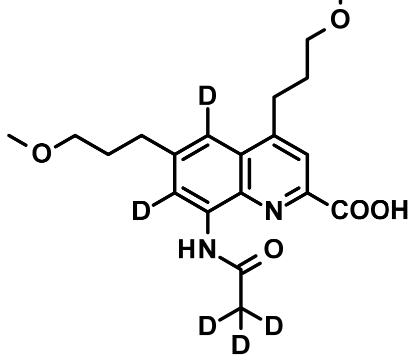

Using **General Procedure 6** and 232 mg **methyl 5,7-dideuterio-4,6-bis(3-methoxypropyl)-8-[(2,2,2-trideuterioacetyl)amino]quinoline-2-carboxylate** (87% purity, 0.512 mmol, 1.0 equiv.), 89 mg **5,7-dideuterio-4,6-bis(3-methoxypropyl)-8-[(2,2,2-trideuterioacetyl)amino]quinoline-2-carboxylic acid** was obtained as a yellow oil (99.9% purity, 0.235 mmol, 46%).

HPLC-UV purity: 99.9%.

^{1}H NMR (500 MHz, [D$_6$]DMSO): δ 10.50 (s, 1H, NH), 7.98 (s, 1H, Ar-H), 3.39 (t, J = 6.3 Hz, 2H, CH$_2$), 3.37 (t, J = 6.3 Hz, 2H, CH$_2$), 3.25 (br s, 6H, OCH$_3$), 3.13 (t, J = 7.6 Hz, 2H, CH$_2$), 2.82 (t, J = 7.6 Hz, 2H, CH$_2$), 1.97-1.84 (m, 4H, CH$_2$). ^{13}C NMR (125 MHz, [D$_6$]DMSO): δ 169.3, 166.2, 149.5, 145.9, 142.7, 135.9, 135.6, 127.9, 120.3, 71.00, 70.99, 57.92, 57.90, 32.7, 30.7, 29.4, 28.2. HRMS (ESI): m/z [M+H]$^+$ calcd for $C_{20}D_5H_{21}N_2O_5$: 380.2228; found: 380.2228.

8-acetamido-4,6-bis[3-(trideuteriomethoxy)propyl]quinoline-2-carboxylic acid (1g)

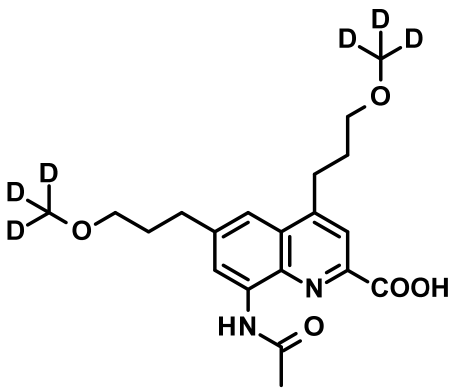

Using **General Procedure 6** and 296 mg **methyl 8-acetamido-4,6-bis[3-(trideuteriomethoxy)propyl]quinoline-2-carboxylate** (92% purity, 0.691 mmol, 1.0 equiv.), 93 mg **8-acetamido-4,6-bis[3-(trideuteriomethoxy)propyl]quinoline-2-carboxylic acid** was obtained as a yellow oil (98.2% purity, 0.244 mmol, 35%).

HPLC-UV purity: 98.2%.

^{1}H NMR (500 MHz, [D$_6$]DMSO): δ 10.47 (s, 1H, NH), 8.63 (s, 1H, Ar-H), 7.95 (s, 1H, Ar-H), 7.59 (s, 1H, Ar-H), 3.38 (t, *J* = 6.3 Hz, 2H, CH$_2$), 3.36 (t, *J* = 6.3 Hz, 2H, CH$_2$), 3.11 (t, *J* = 7.2 Hz, 2H, CH$_2$), 2.81 (t, *J* = 7.2 Hz, 2H, CH$_2$), 2.30 (s, 3H, C(O)CH$_3$), 1.97-1.84 (m, 4H, CH$_2$). ^{13}C NMR (125 MHz, [D$_6$]DMSO): δ 169.1, 166.8, 149.0, 142.3, 135.9, 135.7, 127.6, 120.6, 117.6, 115.8, 70.90, 70.89, 32.8, 30.8, 29.4, 28.2, 24.8. HRMS (ESI): *m/z* [M+H]$^+$ calcd for $C_{20}D_6H_{20}N_2O_5$: 381.2291; found: 381.2292.

8-acetamido-3-deuterio-4,6-bis[3-(trideuteriomethoxy)propyl]quinoline-2-carboxylic acid (1h)

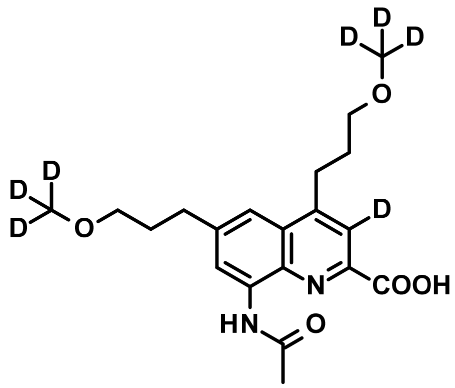

Using **General Procedure 6** and 260 mg **methyl 8-acetamido-3-deuterio-4,6-bis[3-(trideuteriomethoxy)propyl]quinoline-2-carboxylate** (90% purity, 0.661 mmol, 1.0 equiv.), 65 mg **8-acetamido-3-deuterio-4,6-bis[3-(trideuteriomethoxy)propyl]quinoline-2-carboxylic acid** was obtained as a yellow oil (99.3% purity, 0.170 mmol, 26%).

HPLC-UV purity: 99.3%.

^{1}H NMR (500 MHz, [D$_6$]DMSO): δ 10.47 (s, 1H, NH), 8.73 (d, *J* = 1.3 Hz, 1H, Ar-H), 7.62 (d, *J* = 1.3 Hz, 1H, Ar-H), 3.38 (t, *J* = 6.3 Hz, 2H, CH$_2$), 3.37 (t, *J* = 6.3 Hz, 2H, CH$_2$), 3.14 (t, *J* = 7.2 Hz, 2H, CH$_2$), 2.82 (t, *J* = 7.2 Hz, 2H, CH$_2$), 2.31 (s, 3H, C(O)CH$_3$), 1.97-1.84 (m, 4H, CH$_2$). ^{13}C NMR (125 MHz, [D$_6$]DMSO): δ 169.2, 166.3, 149.3, 142.7, 136.0, 135.6, 127.9, 117.8, 115.9, 70.89, 70.87, 32.8, 30.8, 29.4, 28.1, 24.8. HRMS (ESI): *m/z* [M+H]$^+$ calcd for $C_{20}D_7H_{19}N_2O_5$: 382.2354; found: 382.2355.

8-acetamido-5,7-dideuterio-4,6-bis[3-(trideuteriomethoxy)propyl]quinoline-2-carboxylic acid (1i)

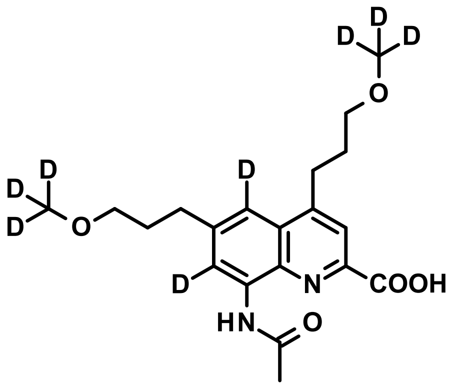

Using **General Procedure 6** and 250 mg **methyl 8-acetamido-5,7-dideuterio-4,6-bis[3-(trideuteriomethoxy)propyl]quinoline-2-carboxylate** (87% purity, 0.548 mmol, 1.0 equiv.), 60 mg **8-acetamido-5,7-dideuterio-4,6-bis[3-(trideuteriomethoxy)propyl]quinoline-2-carboxylic acid** was obtained as a yellow oil (98.2% purity, 0.154 mmol, 28%).

HPLC-UV purity: 98.2%.

^{1}H NMR (500 MHz, [D_6]DMSO): δ 10.49 (s, 1H, NH), 8.00 (s, 1H, Ar-H), 3.39 (t, *J* = 6.3 Hz, 2H, CH_2), 3.36 (t, *J* = 6.3 Hz, 2H, CH_2), 3.14 (t, *J* = 7.2 Hz, 2H, CH_2), 2.82 (t, *J* = 7.2 Hz, 2H, CH_2), 2.31 (s, 3H, C(O)CH_3), 1.97-1.84 (m, 4H, CH_2). ^{13}C NMR (125 MHz, [D_6]DMSO): δ 169.2, 166.0, 149.6, 142.8, 135.9, 135.6, 128.0, 120.3, 70.88, 70.87, 32.7, 30.7, 29.4, 28.2, 24.8. HRMS (ESI): *m/z* $[M+H]^+$ calcd for $C_{20}D_8H_{18}N_2O_5$: 383.2417; found: 383.2418.

8-acetamido-4,6-bis(1,1,2,2-tetradeuterio-3-methoxy-propyl)quinoline-2-carboxylic acid (1i*)

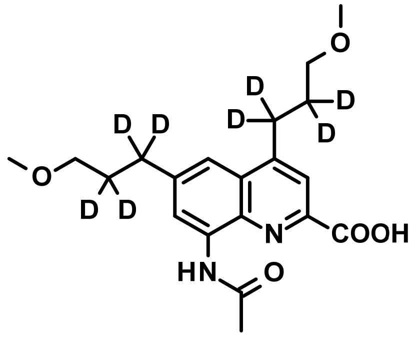

Using **General Procedure 6** and 268 mg **methyl 8-acetamido-4,6-bis(1,1,2,2-tetradeuterio-3-methoxy-propyl)quinoline-2-carboxylate** (94% purity, 0.712 mmol, 1.0 equiv.), 53 mg **8-acetamido-4,6-bis(1,1,2,2-tetradeuterio-3-methoxy-propyl)quinoline-2-carboxylic acid** was obtained as a yellow oil (0.139 mmol, 19%).

HPLC-UV purity: 99.6%.

^{1}H NMR (500 MHz, [D_6]DMSO): δ 10.47 (s, 1H, NH), 8.59 (d, *J* = 1.4 Hz, 1H, Ar-H), 7.90 (s, 1H, Ar-H), 7.56 (d, *J* = 1.4 Hz, 1H, Ar-H), 3.36 (s, 2H, CH_2), 3.35 (s, 2H, CH_2), 3.252 (s, 3H, OCH_3), 3.251 (s, 3H, OCH_3), 2.29 (s, 3H, C(O)CH_3). ^{13}C NMR (125 MHz, [D_6]DMSO): δ 169.0, 167.3, 148.4, 141.7, 135.8, 135.7, 127.3, 120.9, 117.2, 115.7, 70.90, 70.89, 57.92, 57.90, 24.8. HRMS (ESI): *m/z* $[M+H]^+$ calcd for $C_{20}D_8H_{18}N_2O_5$: 383.2417; found: 383.2414.

8-[(2,2,2-trideuterioacetyl)amino]-4,6-bis[3-(trideuteriomethoxy)propyl]quinoline-2-carboxylic acid (1j)

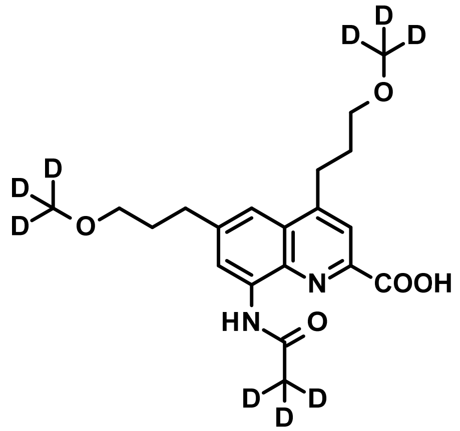

Using **General Procedure 6** and 185 mg **methyl 8-[(2,2,2-trideuterioacetyl)amino]-4,6-bis[3-(trideuteriomethoxy)propyl]quinoline-2-carboxylate** (92% purity, 0.423 mmol, 1.0 equiv.), 41 mg **8-[(2,2,2-trideuterioacetyl)amino]-4,6-bis[3-(trideuteriomethoxy)propyl]quinoline-2-carboxylic acid** was obtained as a yellow oil (94.9% purity, 0.102 mmol, 24%).

HPLC-UV purity: 94.9%.

^{1}H NMR (500 MHz, [D_6]DMSO): δ 10.49 (s, 1H, NH), 8.68 (d, *J* = 1.1 Hz, 1H, Ar-H), 8.00 (s, 1H, Ar-H), 7.63 (d, *J* = 1.1 Hz, 1H, Ar-H), 3.39 (t, *J* = 6.3 Hz, 2H, CH_2), 3.36 (t, *J* = 6.3 Hz, 2H, CH_2), 3.14 (t, *J* = 7.2 Hz, 2H, CH_2), 2.83 (t, *J* = 7.2 Hz, 2H, CH_2), 1.97-1.84 (m, 4H, CH_2). ^{13}C NMR (125 MHz, [D_6]DMSO): δ 169.3, 166.0, 149.7, 143.0, 136.0, 135.6, 128.0, 120.3, 118.0, 115.9, 70.89, 70.87, 32.8, 30.8, 29.4, 28.2. HRMS (ESI): *m/z* $[M+H]^+$ calcd for $C_{20}D_9H_{17}N_2O_5$: 384.2479; found: 384.2483.

3-deuterio-8-[(2,2,2-trideuterioacetyl)amino]-4,6-bis[3-(trideuteriomethoxy)propyl]quinoline-2-carboxylic acid (1k)

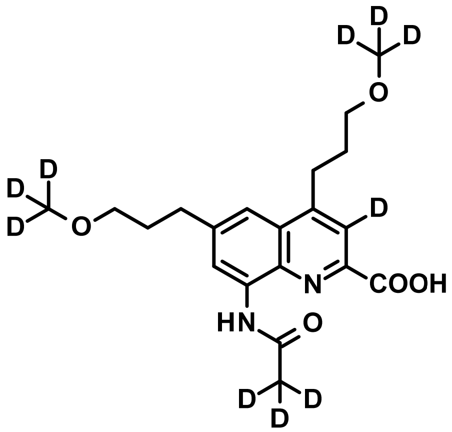

Using **General Procedure 6** and 200 mg **methyl 3-deuterio-8-[(2,2,2-trideuterioacetyl)amino]-4,6-bis[3-(trideuteriomethoxy)propyl]quinoline-2-carboxylate** (82% purity, 0.412 mmol, 1.0 equiv.), 119 mg **3-deuterio-8-[(2,2,2-trideuterioacetyl)amino]-4,6-bis[3-(trideuteriomethoxy)propyl]quinoline-2-carboxylic acid** was obtained as a yellow oil (97.2% purity, 0.300 mmol, 73%).

HPLC-UV purity: 97.2%.

^{1}H NMR (500 MHz, [D$_6$]DMSO): δ 10.48 (s, 1H, NH), 8.63 (d, *J* = 1.3 Hz, 1H, Ar-H), 7.59 (d, *J* = 1.3 Hz, 1H, Ar-H), 3.38 (t, *J* = 6.3 Hz, 2H, CH$_2$), 3.36 (t, *J* = 6.3 Hz, 2H, CH$_2$), 3.11 (t, *J* = 7.2 Hz, 2H, CH$_2$), 2.82 (t, *J* = 7.2 Hz, 2H, CH$_2$), 1.97-1.84 (m, 4H, CH$_2$). ^{13}C NMR (125 MHz, [D$_6$]DMSO): δ 169.2, 166.8, 148.9, 147.7, 142.3, 135.9, 135.7, 127,6, 117.5, 115.8, 70.90, 70.89, 32.8, 30.8, 29.4, 28.1. HRMS (ESI): *m/z* [M+H]$^+$ calcd for $C_{20}D_{10}H_{16}N_2O_5$: 385.2542; found: 384.2483.

5,7-dideuterio-8-[(2,2,2-trideuterioacetyl)amino]-4,6-bis[3-(trideuteriomethoxy)propyl]quinoline-2-carboxylic acid (1l)

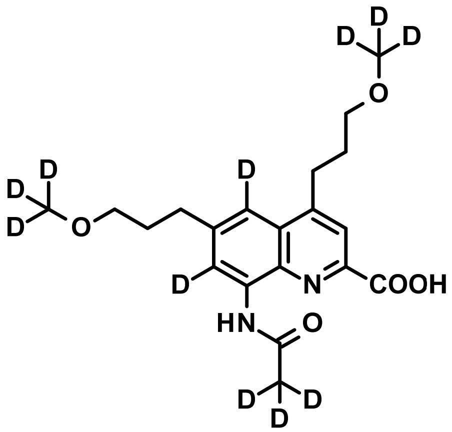

Using **General Procedure 6** and 170 mg **methyl 5,7-dideuterio-8-[(2,2,2-trideuterioacetyl)amino]-4,6-bis[3-(trideuteriomethoxy)propyl]quinoline-2-carboxylate** (77% purity, 0.328 mmol, 1.0 equiv.), 45 mg **5,7-dideuterio-8-[(2,2,2-trideuterioacetyl)amino]-4,6-bis[3-(trideuteriomethoxy)propyl]quinoline-2-carboxylic acid** was obtained as a yellow oil (93.3% purity, 0.109 mmol, 33%).

HPLC-UV purity: 93.3%.

^{1}H NMR (500 MHz, [D$_6$]DMSO): δ 10.47 (s, 1H, NH), 7.97 (s, 1H, Ar-H), 3.39 (t, *J* = 6.3 Hz, 2H, CH$_2$), 3.36 (t, *J* = 6.3 Hz, 2H, CH$_2$), 3.13 (t, *J* = 7.2 Hz, 2H, CH$_2$), 2.82 (t, *J* = 7.2 Hz, 2H, CH$_2$), 1.97-1.84 (m, 4H, CH$_2$). ^{13}C NMR (125 MHz, [D$_6$]DMSO): δ 169.2, 166.6, 149.1, 147.1, 142.4, 135.9, 135.7, 127.7, 120.5, 70.90, 70.89, 32.7, 30.8, 29.4, 28.2. HRMS (ESI): *m/z* [M+H]$^+$ calcd for $C_{20}D_{11}H_{15}N_2O_5$: 386.2605; found: 386.2606.

3-deuterio-4,6-bis(1,1,2,2-tetradeuterio-3-methoxy-propyl)-8-[(2,2,2-trideuterioacetyl)amino]quinoline-2-carboxylic acid (1m)

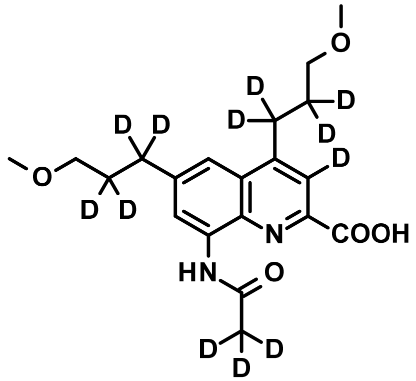

Using **General Procedure 6** and 160 mg **methyl 3-deuterio-4,6-bis(1,1,2,2-tetradeuterio-3-methoxy-propyl)-8-[(2,2,2-trideuterioacetyl)amino]quinoline-2-carboxylate** (81% purity, 0.324 mmol, 1.0 equiv.), 57 mg **3-deuterio-4,6-bis(1,1,2,2-tetradeuterio-3-methoxy-propyl)-8-[(2,2,2-trideuterioacetyl)amino]quinoline-2-carboxylic acid** was obtained as a yellow oil (96.2% purity, 0.142 mmol, 33%).

HPLC-UV purity: 96.2%.

^{1}H NMR (500 MHz, [D$_6$]DMSO): δ 10.46 (s, 1H, NH), 8.64 (d, *J* = 1.7 Hz, 1H, Ar-H), 7.59 (d, *J* = 1.7 Hz, 1H, Ar-H), 3.37 (s, 2H, CH$_2$), 3.35 (s, 2H, CH$_2$), 3.25 (br s, 6H, OCH$_3$). ^{13}C NMR (125 MHz, [D$_6$]DMSO): δ 169.2, 166.5, 149.1, 142.5, 135.9, 135.6, 127.8, 117.7, 115.8, 70.87, 70.86, 57.92, 57.91. HRMS (ESI): *m/z* [M+H]$^+$ calcd for $C_{20}D_{12}H_{14}N_2O_5$: 387.2668; found: 387.2666.

5,7-dideuterio-4,6-bis(1,1,2,2-tetradeuterio-3-methoxy-propyl)-8-[(2,2,2-trideuterioacetyl)amino]quinoline-2-carboxylic acid (1n)

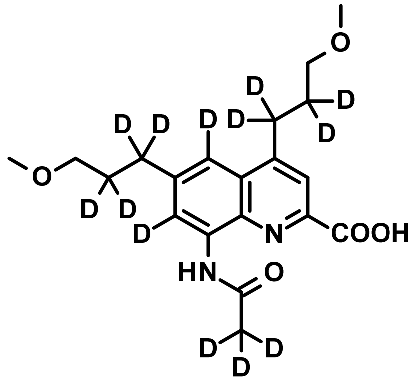

Using **General Procedure 6** and 170 mg **methyl 5,7-dideuterio-4,6-bis(1,1,2,2-tetradeuterio-3-methoxy-propyl)-8-[(2,2,2-trideuterioacetyl)amino]quinoline-2-carboxylate** (70% purity, 0.296 mmol, 1.0 equiv.), 53 mg **5,7-dideuterio-4,6-bis(1,1,2,2-tetradeuterio-3-methoxy-propyl)-8-[(2,2,2-trideuterioacetyl)amino]quinoline-2-carboxylic acid** was obtained as a yellow oil (93.9% purity, 0.129 mmol, 43%).

HPLC-UV purity: 93.9%.

^{1}H NMR (500 MHz, [D$_6$]DMSO): δ 10.50 (s, 1H, NH), 7.97 (s, 1H, Ar-H), 3.37 (s, 2H, CH$_2$), 3.35 (s, 2H, CH$_2$), 3.25 (br s, 6H, OCH$_3$). ^{13}C NMR (125 MHz, [D$_6$]DMSO): δ 169.2, 166.3, 149.3, 146.3, 142.5, 135.9, 135.6, 127.8, 120.4, 70.87, 70.86, 57.93, 57.91. HRMS (ESI): *m/z* [M+H]$^+$ calcd for $C_{20}D_{13}H_{13}N_2O_5$: 388.2730; found: 388.2731.

8-acetamido-4,6-bis[1,1,2,2-tetradeuterio-3-(trideuteriomethoxy)propyl]quinoline-2-carboxylic acid (1o)

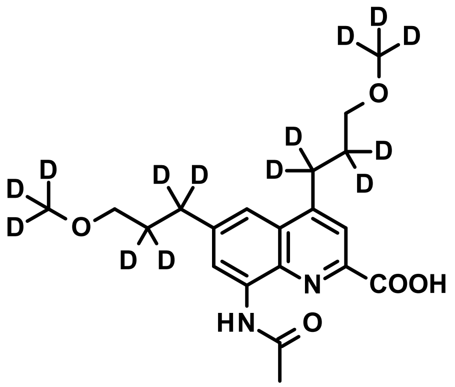

Using **General Procedure 6** and 200 mg **methyl 8-acetamido-4,6-bis[1,1,2,2-tetradeuterio-3-(trideuteriomethoxy)propyl]quinoline-2-carboxylate** (87% purity, 0.432 mmol, 1.0 equiv.), 51 mg **8-acetamido-4,6-bis[1,1,2,2-tetradeuterio-3-(trideuteriomethoxy)propyl]quinoline-2-carboxylic acid** was obtained as a yellow oil (99.7% purity, 0.131 mmol, 47%).

HPLC-UV purity: 99.7%.

^{1}H NMR (500 MHz, [D$_6$]DMSO): δ 13.36 (br s, 1H, COOH), 10.48 (s, 1H, NH), 8.70 (d, *J* = 1.7 Hz, 1H, Ar-H), 8.02 (s, 1H, Ar-H), 7.64 (d, *J* = 1.7 Hz, 1H, Ar-H), 3.37 (s, 2H, CH$_2$), 3.35 (s, 2H, CH$_2$), 2.32 (s, 3H, C(O)CH$_3$). ^{13}C NMR (125 MHz, [D$_6$]DMSO): δ 169.3, 165.6, 150.0, 144.4, 143.2, 136.0, 135.6, 128.3, 120.2, 118.2, 116.0, 70.73, 70.72, 24.8. HRMS (ESI): *m/z* [M+H]$^+$ calcd for $C_{20}D_{14}H_{12}N_2O_5$: 389.2793; found: 389.2972.

8-acetamido-3-deuterio-4,6-bis[1,1,2,2-tetradeuterio-3-(trideuteriomethoxy)propyl]quinoline-2-carboxylic acid (1p)

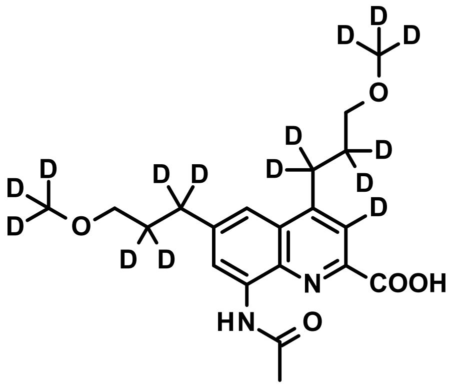

Using **General Procedure 6** and 195 mg **methyl 8-acetamido-3-deuterio-4,6-bis[1,1,2,2-tetradeuterio-3-(trideuteriomethoxy)propyl]quinoline-2-carboxylate** (65% purity, 0.314 mmol, 1.0 equiv.), 66 mg **8-acetamido-3-deuterio-4,6-bis[1,1,2,2-tetradeuterio-3-(trideuteriomethoxy)propyl]quinoline-2-carboxylic acid** was obtained as a yellow oil (99.5% purity, 0.169 mmol, 54%).

HPLC-UV purity: 99.5%.

^{1}H NMR (500 MHz, [D$_6$]DMSO): δ 13.32 (br s, 1H, COOH), 10.47 (s, 1H, NH), 8.70 (d, J = 1.6 Hz, 1H, Ar-H), 7.64 (d, J = 1.6 Hz, 1H, Ar-H), 3.38 (s, 2H, CH$_2$), 3.35 (s, 2H, CH$_2$), 2.32 (s, 3H, C(O)CH$_3$). ^{13}C NMR (125 MHz, [D$_6$]DMSO): δ 169.3, 165.6, 150.0, 144.2, 143.3, 136.0, 135.6, 128.3, 118.2, 116.0, 70.73, 70.72, 24.8. HRMS (ESI): *m/z* [M+H]$^+$ calcd for $C_{20}D_{15}H_{11}N_2O_5$: 390.2856; found: 390.2856.

8-acetamido-5,7-dideuterio-4,6-bis[1,1,2,2-tetradeuterio-3-(trideuteriomethoxy)propyl]quinoline-2-carboxylic acid (1q)

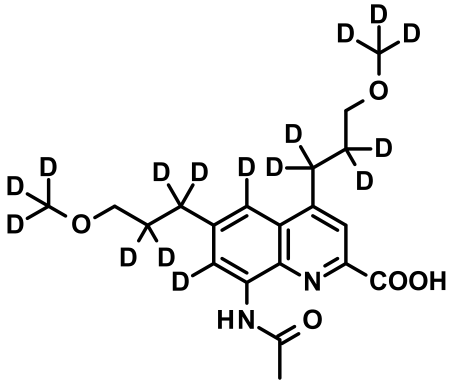

Using **General Procedure 6** and 180 mg **methyl 8-acetamido-5,7-dideuterio-4,6-bis[1,1,2,2-tetradeuterio-3-(trideuteriomethoxy)propyl]quinoline-2-carboxylate** (74% purity, 0.329 mmol, 1.0 equiv.), 74 mg **8-acetamido-5,7-dideuterio-4,6-bis[1,1,2,2-tetradeuterio-3-(trideuteriomethoxy)propyl]quinoline-2-carboxylic acid** was obtained as a yellow oil (98.7% purity, 0.190 mmol, 58%).

HPLC-UV purity: 98.7%.

^{1}H NMR (500 MHz, [D$_6$]DMSO): δ 13.33 (br s, 1H, COOH), 10.47 (s, 1H, NH), 8.03 (s, 1H, Ar-H), 3.38 (s, 2H, CH$_2$), 3.35 (s, 2H, CH$_2$), 2.32 (s, 3H, C(O)CH$_3$). ^{13}C NMR (125 MHz, [D$_6$]DMSO): δ 169.2, 165.6, 150.1, 144.2, 143.1, 135.9, 135.6, 128.2, 120.2, 70.73, 70.72, 24.8. HRMS (ESI): *m/z* [M+H]$^+$ calcd for $C_{20}D_{16}H_{10}N_2O_5$: 391.2919; found: 391.2919.

4,6-bis[1,1,2,2-tetradeuterio-3-(trideuteriomethoxy)propyl]-8-[(2,2,2-trideuterioacetyl)amino]quinoline-2-carboxylic acid (1r)

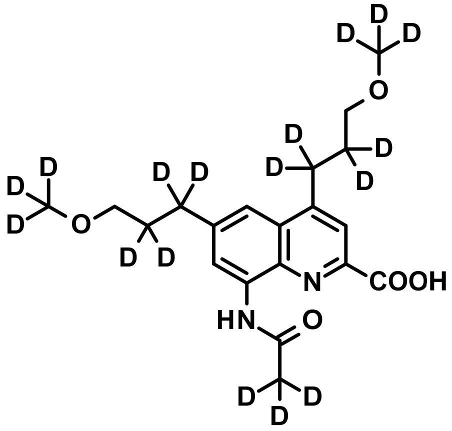

Using **General Procedure 6** and 90 mg **methyl 4,6-bis[1,1,2,2-tetradeuterio-3-(trideuteriomethoxy)propyl]-8-[(2,2,2-trideuterioacetyl)amino]quinoline-2-carboxylate** (89% purity, 0.198 mmol, 1.0 equiv.), 40 mg **4,6-bis[1,1,2,2-tetradeuterio-3-(trideuteriomethoxy)propyl]-8-[(2,2,2-trideuterioacetyl)amino]quinoline-2-carboxylic acid** was obtained as a yellow oil (99.8% purity, 0.102 mmol, 41%).

HPLC-UV purity: 99.8%.

^{1}H NMR (500 MHz, [D$_6$]DMSO): δ 10.51 (s, 1H, NH), 8.64 (d, J = 1.5 Hz, 1H, Ar-H), 7.95 (s, 1H, Ar-H), 7.58 (d, J = 1.5 Hz, 1H, Ar-H), 3.37 (s, 2H, CH$_2$),

3.35 (s, 2H, CH_2). ^{13}C NMR (125 MHz, $[D_6]DMSO$): δ 169.2, 166.7, 149.0, 147.4, 142.4, 135.9, 135.7, 127.7, 120.6, 117.6, 115.8, 70.76, 70.75. HRMS (ESI): *m/z* $[M+H]^+$ calcd for $C_{20}D_{17}H_9N_2O_5$: 392.2982; found: 392.2981.

3-deuterio-4,6-bis[1,1,2,2-tetradeuterio-3-(trideuteriomethoxy)propyl]-8-[(2,2,2-trideuterioacetyl)amino]quinoline-2-carboxylic acid (1s)

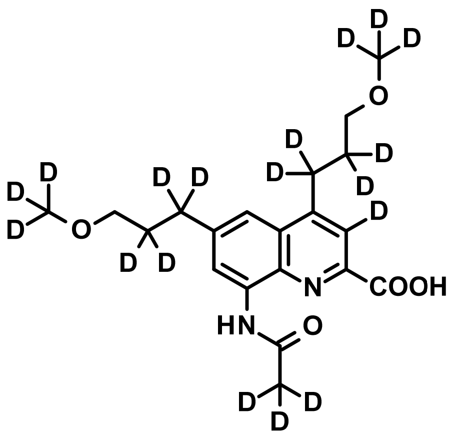

Using **General Procedure 6** and 90 mg **methyl 3-deuterio-4,6-bis[1,1,2,2-tetradeuterio-3-(trideuteriomethoxy)propyl]-8-[(2,2,2-trideuterioacetyl)amino]quinoline-2-carboxylate** (81% purity, 0.179 mmol, 1.0 equiv.), 34 mg **3-deuterio-4,6-bis[1,1,2,2-tetradeuterio-3-(trideuteriomethoxy)propyl]-8-[(2,2,2-trideuterioacetyl)amino]quinoline-2-carboxylic acid** was obtained as a yellow oil (98.9% purity, 0.087 mmol, 48%).

HPLC-UV purity: 98.9%.

^1H NMR (500 MHz, $[D_6]DMSO$): δ 13.35 (br s, 1H, COOH), 10.47 (s, 1H, NH), 8.70 (d, J = 1.6 Hz, 1H, Ar-H), 7.63 (d, J = 1.6 Hz, 1H, Ar-H), 3.37 (s, 2H, CH_2), 3.35 (s, 2H, CH_2). ^{13}C NMR (125 MHz, $[D_6]DMSO$): δ 169.3, 165.6, 149.9, 144.2, 143.3, 136.0, 135.6, 128.3, 118.2, 116.0, 70.73, 70.72. HRMS (ESI): *m/z* $[M+H]^+$ calcd for $C_{20}D_{18}H_8N_2O_5$: 393.3044; found: 393.3044.

5,7-dideuterio-4,6-bis[1,1,2,2-tetradeuterio-3-(trideuteriomethoxy)propyl]-8-[(2,2,2-trideuterioacetyl)amino]quinoline-2-carboxylic acid (1t)

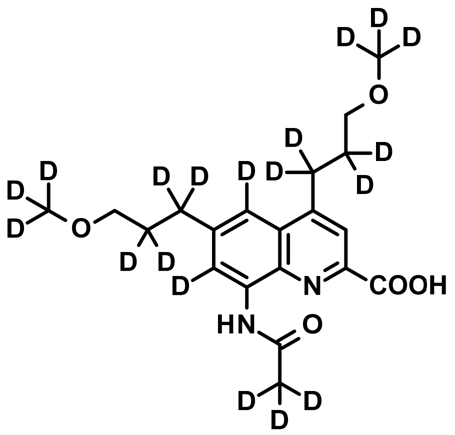

Using **General Procedure 6** and 170 mg **methyl 5,7-dideuterio-4,6-bis[1,1,2,2-tetradeuterio-3-(trideuteriomethoxy)propyl]-8-[(2,2,2-trideuterioacetyl)amino]quinoline-2-carboxylate** (70% purity, 0.292 mmol, 1.0 equiv.), 31 mg **5,7-dideuterio-4,6-bis[1,1,2,2-tetradeuterio-3-(trideuteriomethoxy)propyl]-8-[(2,2,2-trideuterioacetyl)amino]quinoline-2-carboxylic acid** was obtained as a yellow oil (99.2% purity, 0.079 mmol, 27%).

HPLC-UV purity: 99.2%.

^1H NMR (500 MHz, $[D_6]DMSO$): δ 10.47 (s, 1H, NH), 8.04 (s, 1H, Ar-H), 3.38 (s, 2H, CH_2), 3.35 (s, 2H, CH_2). ^{13}C NMR (125 MHz, $[D_6]DMSO$): δ 169.3, 166.2, 149.5, 145.9, 142.6, 135.9, 135.6, 127.9, 120.4, 70.75, 70.74. HRMS (ESI): *m/z* $[M+H]^+$ calcd for $C_{20}D_{19}H_7N_2O_5$: 394.3107; found: 394.3107.

3,5,7-trideuterio-4,6-bis[1,1,2,2-tetradeuterio-3-(trideuteriomethoxy)propyl]-8-[(2,2,2-trideuterioacetyl)amino]quinoline-2-carboxylic acid (1u)

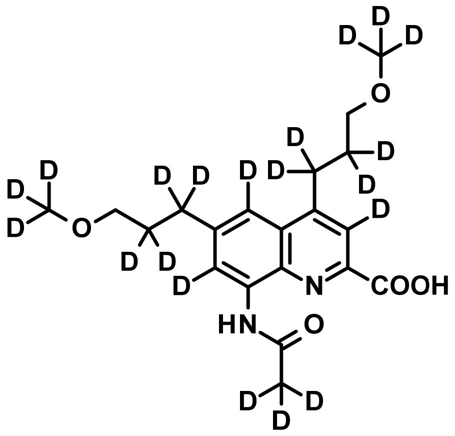

Using **General Procedure 6** and 90 mg **methyl 3,5,7-trideuterio-4,6-bis[1,1,2,2-tetradeuterio-3-(trideuteriomethoxy)propyl]-8-[(2,2,2-trideuterioacetyl)amino]quinoline-2-carboxylate** (92% purity, 0.203 mmol, 1.0 equiv.), 17 mg **3,5,7-trideuterio-4,6-bis[1,1,2,2-tetradeuterio-3-(trideuteriomethoxy)propyl]-8-[(2,2,2-trideuterioacetyl)amino]quinoline-2-carboxylic acid** was obtained as a yellow oil (95.9% purity, 0.041 mmol, 19%).

HPLC-UV purity: 95.9%.

^{1}H NMR (500 MHz, [D$_6$]DMSO): δ 10.49 (s, 1H, NH), 3.38 (s, 2H, CH_2), 3.35 (s, 2H, CH_2). ^{13}C NMR (125 MHz, [D$_6$]DMSO): δ 169.3, 165.8, 149.8, 144.7, 143.0, 135.9, 135.6, 128.1, 70.74, 70.72. HRMS (ESI): *m/z* $[M+H]^+$ calcd for $C_{20}D_{20}H_6N_2O_5$: 395.3170; found: 395.3171.

4,6-bis[1,1,2,2,3,3-hexadeuterio-3-(trideuteriomethoxy)propyl]-8-[(2,2,2-trideuterioacetyl)amino]quinoline-2-carboxylic acid (1v)

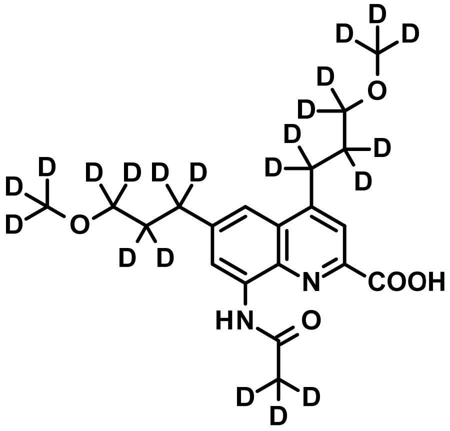

Using **General Procedure 6** and 190 mg **methyl 4,6-bis[1,1,2,2,3,3-hexadeuterio-3-(trideuteriomethoxy)propyl]-8-[(2,2,2-trideuterioacetyl)amino]quinoline-2-carboxylate** (63% purity, 0.292 mmol, 1.0 equiv.), 30 mg **4,6-bis[1,1,2,2,3,3-hexadeuterio-3-(trideuteriomethoxy)propyl]-8-[(2,2,2-trideuterioacetyl)amino]quinoline-2-carboxylic acid** was obtained as a yellow oil (98.9% purity, 0.075 mmol, 25%).

HPLC-UV purity: 98.9%.

^{1}H NMR (500 MHz, [D$_6$]DMSO): δ 10.52 (s, 1H, NH), 8.66 (d, *J* = 1.5 Hz, 1H, Ar-H), 7.98 (s, 1H, Ar-H), 7.61 (d, *J* = 1.5 Hz, 1H, Ar-H). ^{13}C NMR (125 MHz, [D$_6$]DMSO): δ 169.3, 166.2, 149.5, 145.9, 142.8, 136.0, 135.6, 128.0, 120.4, 117.9, 115.9. HRMS (ESI): *m/z* $[M+H]^+$ calcd for $C_{20}D_{21}H_5N_2O_5$: 396.3230; found: 396.3233.

3-deuterio-4,6-bis[1,1,2,2,3,3-hexadeuterio-3-(trideuteriomethoxy)propyl]-8-[(2,2,2-trideuterioacetyl)amino]quinoline-2-carboxylic acid (1w)

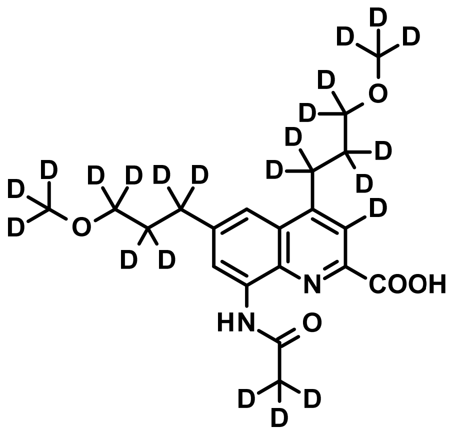

Using **General Procedure 6** and 250 mg **methyl 3-deuterio-4,6-bis[1,1,2,2,3,3-hexadeuterio-3-(trideuteriomethoxy)propyl]-8-[(2,2,2-trideuterioacetyl)amino]quinoline-2-carboxylate** (51% purity, 0.311 mmol, 1.0 equiv.), 30 mg **3-deuterio-4,6-bis[1,1,2,2,3,3-hexadeuterio-3-(trideuteriomethoxy)propyl]-8-[(2,2,2-trideuterioacetyl)amino]quinoline-2-carboxylic acid** was obtained as a yellow oil (95.2% purity, 0.072 mmol, 21%).

HPLC-UV purity: 95.2%.

^{1}H NMR (500 MHz, [D$_6$]DMSO): δ 10.53 (s, 1H, NH), 8.66 (d, *J* = 1.6 Hz, 1H, Ar-H), 7.60 (d, *J* = 1.6 Hz, 1H, Ar-H). ^{13}C NMR (125 MHz, [D$_6$]DMSO): δ 169.2, 166.3, 149.3, 146.2, 142.7, 136.0, 135.6, 127.9, 117.8, 115.9. HRMS (ESI): *m/z* [M+H]$^+$ calcd for $C_{20}D_{22}H_4N_2O_5$: 397.3295; found: 397.3297.

5,7-dideuterio-4,6-bis[1,1,2,2,3,3-hexadeuterio-3-(trideuteriomethoxy)propyl]-8-[(2,2,2-trideuterioacetyl)amino]quinoline-2-carboxylic acid (1x)

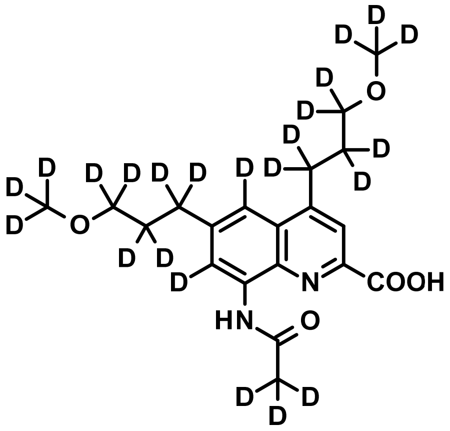

Using **General Procedure 6** and 200 mg **methyl 5,7-dideuterio-4,6-bis[1,1,2,2,3,3-hexadeuterio-3-(trideuteriomethoxy)propyl]-8-[(2,2,2-trideuterioacetyl)amino]quinoline-2-carboxylate** (70% purity, 0.340 mmol, 1.0 equiv.), 41 mg **5,7-dideuterio-4,6-bis[1,1,2,2,3,3-hexadeuterio-3-(trideuteriomethoxy)propyl]-8-[(2,2,2-trideuterioacetyl)amino]quinoline-2-carboxylic acid** was obtained as a yellow oil (99.5% purity, 0.103 mmol, 30%).

HPLC-UV purity: 99.5%.

^{1}H NMR (500 MHz, [D$_6$]DMSO): δ 10.51 (s, 1H, NH), 7.97 (s, 1H, Ar-H). ^{13}C NMR (125 MHz, [D$_6$]DMSO): δ 169.2, 166.3, 149.4, 146.3, 142.5, 135.9, 135.6, 127.8, 120.4. HRMS (ESI): *m/z* [M+H]$^+$ calcd for $C_{20}D_{23}H_3N_2O_5$: 398.3358; found: 398.3359.

3,5,7-trideuterio-4,6-bis[1,1,2,2,3,3-hexadeuterio-3-(trideuteriomethoxy)propyl]-8-[(2,2,2-trideuterioacetyl)amino]quinoline-2-carboxylic acid (1y)

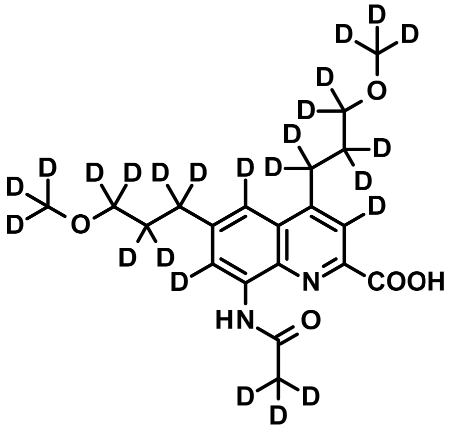

Using **General Procedure 6** and 170 mg **methyl 3,5,7-trideuterio-4,6-bis[1,1,2,2,3,3-hexadeuterio-3-(trideuteriomethoxy)propyl]-8-[(2,2,2-trideuterioacetyl)amino]quinoline-2-carboxylate** (62% purity, 0.256 mmol, 1.0 equiv.), 40 mg **3,5,7-trideuterio-4,6-bis[1,1,2,2,3,3-hexadeuterio-3-(trideuteriomethoxy)propyl]-8-[(2,2,2-trideuterioacetyl)amino]quinoline-2-carboxylic acid** was obtained as a yellow oil (99.2% purity, 0.099 mmol, 39%).

HPLC-UV purity: 99.2%.

^{1}H NMR (500 MHz, [D$_6$]DMSO): δ 10.54 (s, 1H, NH). ^{13}C NMR (125 MHz, [D$_6$]DMSO): δ 169.2, 166.3, 149.3, 146.2, 142.5, 135.9, 135.6, 127.8. HRMS (ESI): *m/z* [M+H]$^+$ calcd for $C_{20}D_{24}H_2N_2O_5$: 399.3421; found: 399.3419.

8-acetamido-N-benzyl-4,6-bis(3-methoxypropyl)quinoline-2-carboxamide (14)

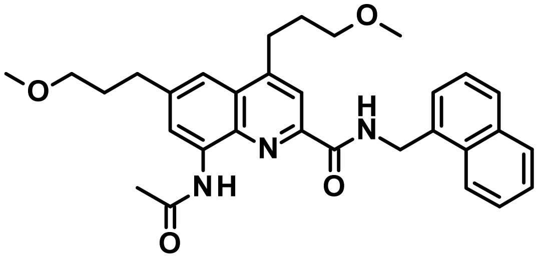

A 20 mL, screw-cap vial equipped with a magnetic stirring bar was filled with 40.00 mg **8-acetamido-4,6-bis(3-methoxypropyl)quinoline-2-carboxylic acid** (1.0 equiv., 0.1068 mmol), 61.4 mg benzotriazol-1-yloxy-tris(dimethylamino)phosphonium, hexafluorophosphate (1:1) (1.3 equiv., 0.1389 mmol), and 37 µL DIPEA (2.0 equiv., 0.2137 mmol) and 3.21 mL dry, degassed DCM. The mixture was stirred at RT for 30 min, then 20.99 mg **1-naphthylmethanamine** (2.0 equiv., 0.2137 mmol) was added and the reaction mixture was stirred for further 30 min, until full conversion was observed. The mixture was concentrated *in vacuo*. Then the crude product was purified *via* RP-HPLC using 1% aq. HCOOH solution and MeCN as eluents to yield 42 mg **8-acetamido-N-benzyl-4,6-bis(3-methoxypropyl)quinoline-2-carboxamide** (0.0818 mmol, 77%) as white crystals**.**

^{1}H NMR (500 MHz, [D6]DMSO): δ 10.32 (s, 1H, NH), 10.07 (t, *J* = 6.2 Hz, 1H, NH), 8.63 (d, *J* = 1.5 Hz, 1H, Ar-H), 8.32 (d, *J* = 8.3 Hz, 1H, Ar-H), 8.09 (s, 1H, Ar-H), 7.97 (dd, *J* = 1.1 Hz, 8.0 Hz, 1H, Ar-H), 7.86 (d, *J* = 8.0 Hz, 1H, Ar-H), 7.65 (d, *J* = 1.5 Hz, 1H, Ar-H), 7.63-7.47 (m, 4H, Ar-H), 5.10 (d, *J* = 6.3 Hz, 2H, CH2), 3.41 (t, *J* = 6.2 Hz, 2H, CH2), 3.37 (t, *J* = 6.2 Hz, 2H, CH2), 3.27 (s, 3H, CH3), 3.25 (s, 3H, CH3), 3.17 (t, *J* = 7.9 Hz, 2H, CH2), 2.83 (t, *J* = 7.9 Hz, 2H, CH2), 2.29 (s, 3H, CH3), 1.98-1.86 (m, 4H, CH2). ^{13}C NMR (125 MHz, [D6]DMSO): δ 169.1, 164.2, 149.7, 147.1, 142.3, 135.7, 135.5, 134.5, 133.3, 130.8, 128.6, 128.0, 127.5, 126.3, 125.8, 125.5, 125.1, 123.5, 119.0, 118.5, 116.1, 71.03, 71.00, 57.9, 40.5, 32.7, 30.7, 29.4, 28.3, 24.7. HRMS (ESI): *m/z* [M+H]$^+$ calcd for $C_{31}H_{35}N_3O_4$: 514.2700; found: 514.2705.

4.3 NMR spectra of newly synthesized compounds

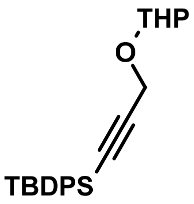

15

^{1}H, ^{13}C NMR

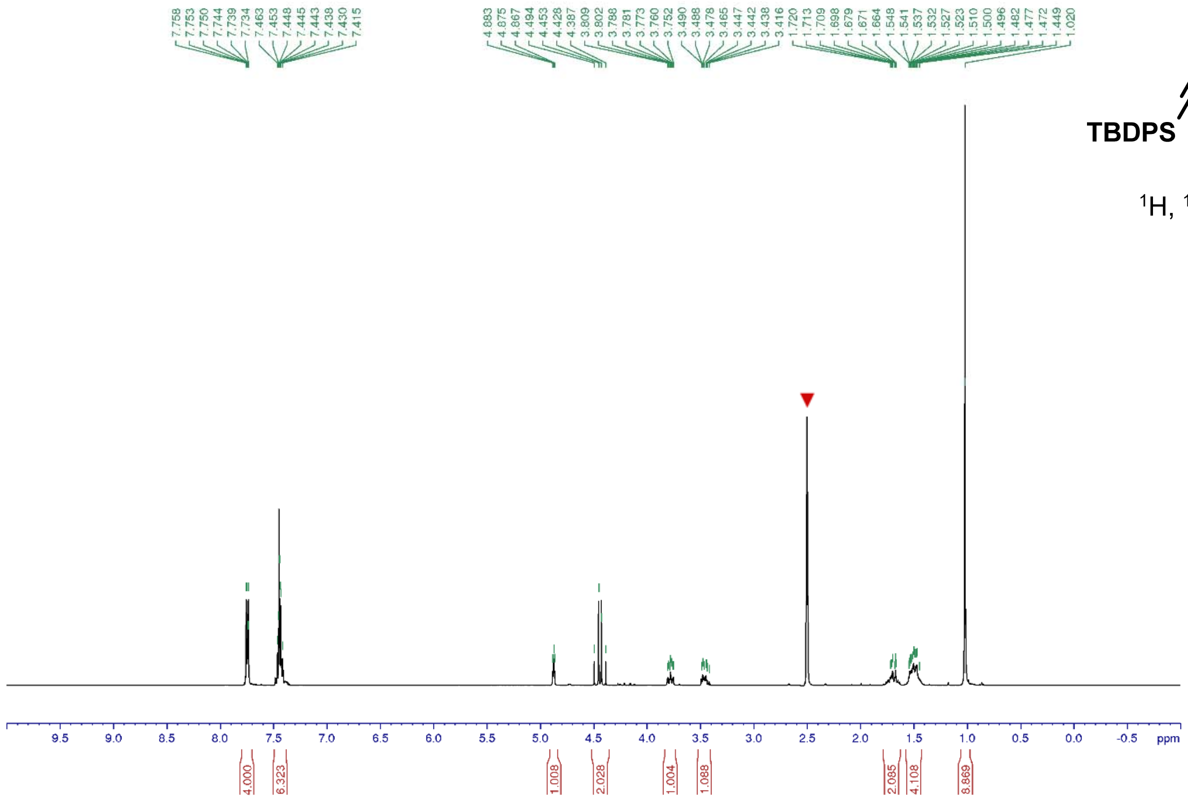

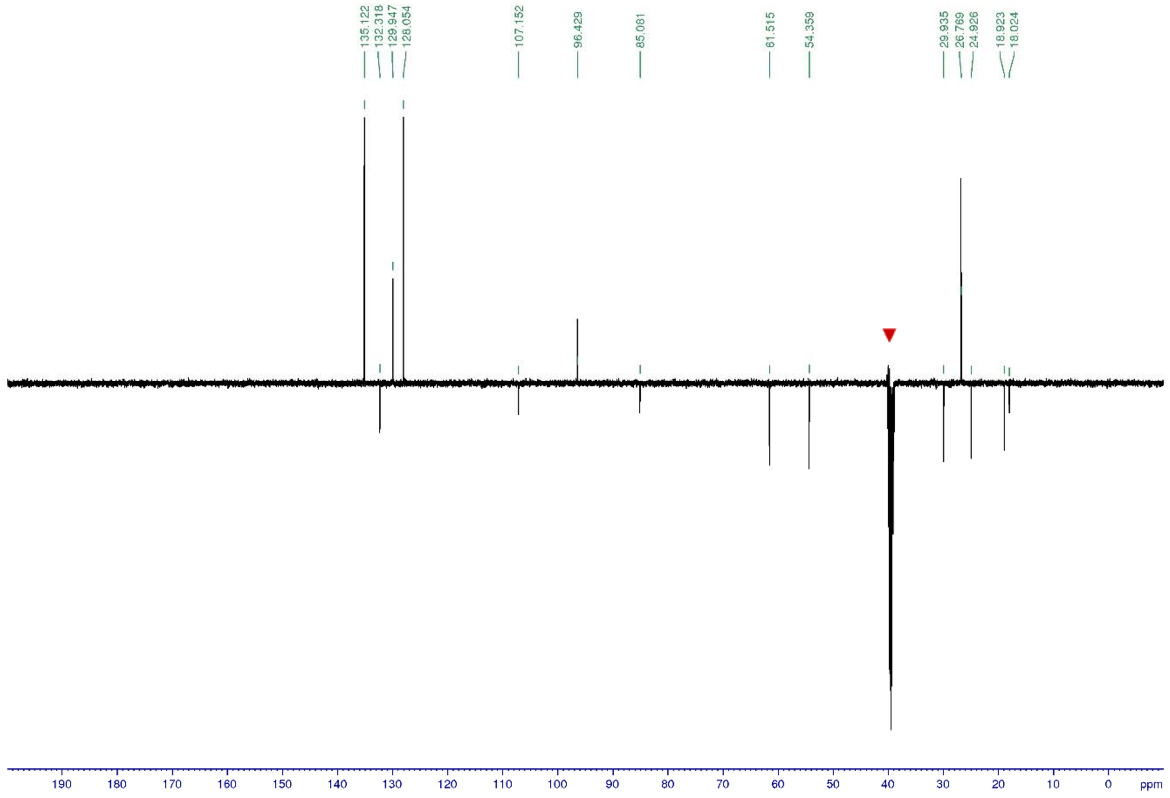

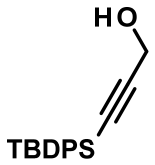
HO
TBDPS

8

^{1}H, ^{13}C NMR

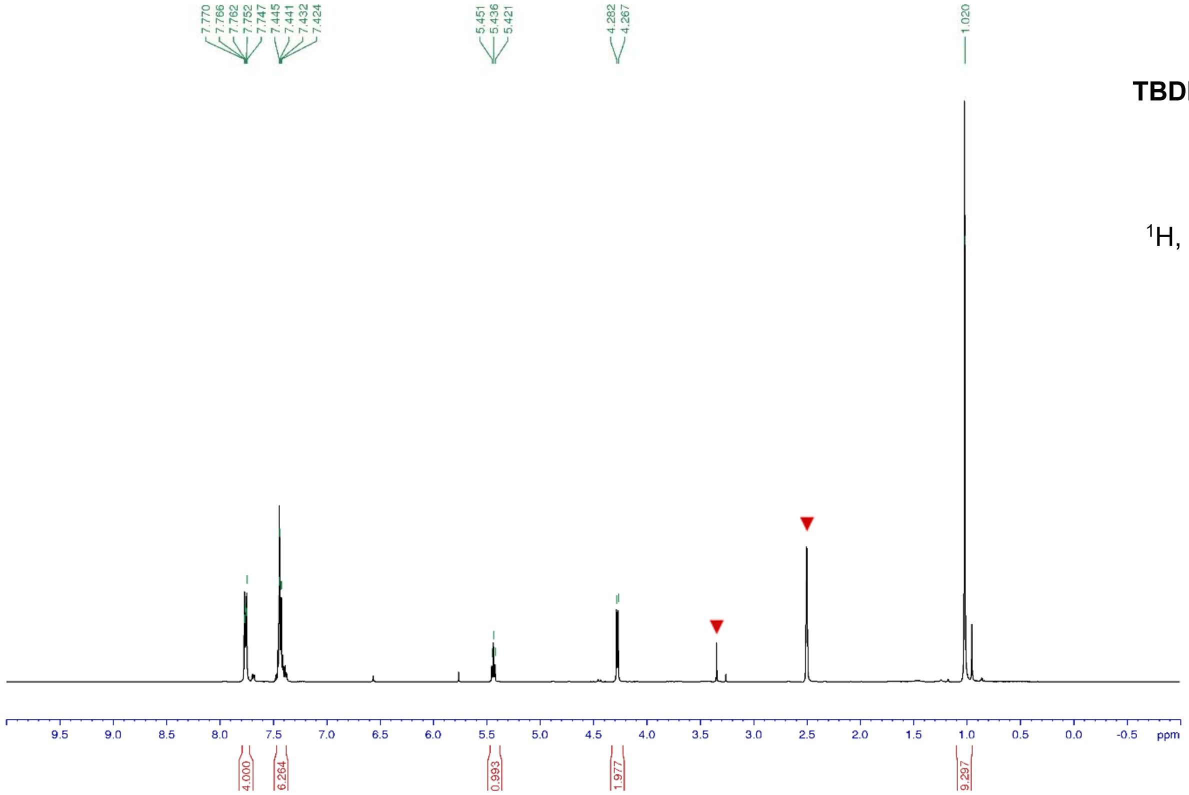
7.770
7.766
7.762
7.752
7.747
7.445
7.441
7.432
7.424
5.451
5.436
5.421
4.282
4.267
1.020
9.5 9.0 8.5 8.0 7.5 7.0 6.5 6.0 5.5 5.0 4.5 4.0 3.5 3.0 2.5 2.0 1.5 1.0 0.5 0.0 -0.5 ppm
4.000
6.264
0.993
1.977
9.297

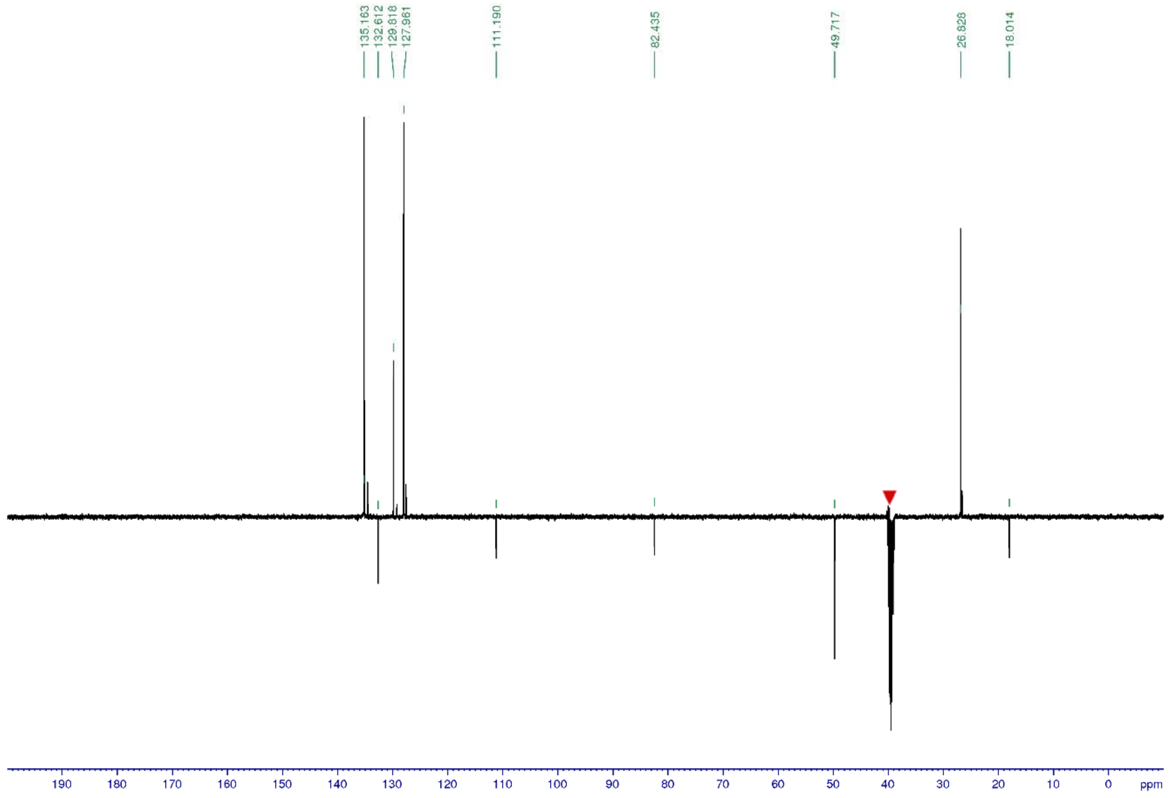
135.163
132.612
129.818
127.961
111.190
82.435
49.717
26.828
18.014
190 180 170 160 150 140 130 120 110 100 90 80 70 60 50 40 30 20 10 0 ppm

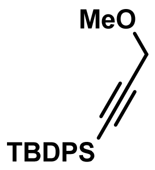

16
^{1}H, ^{13}C NMR

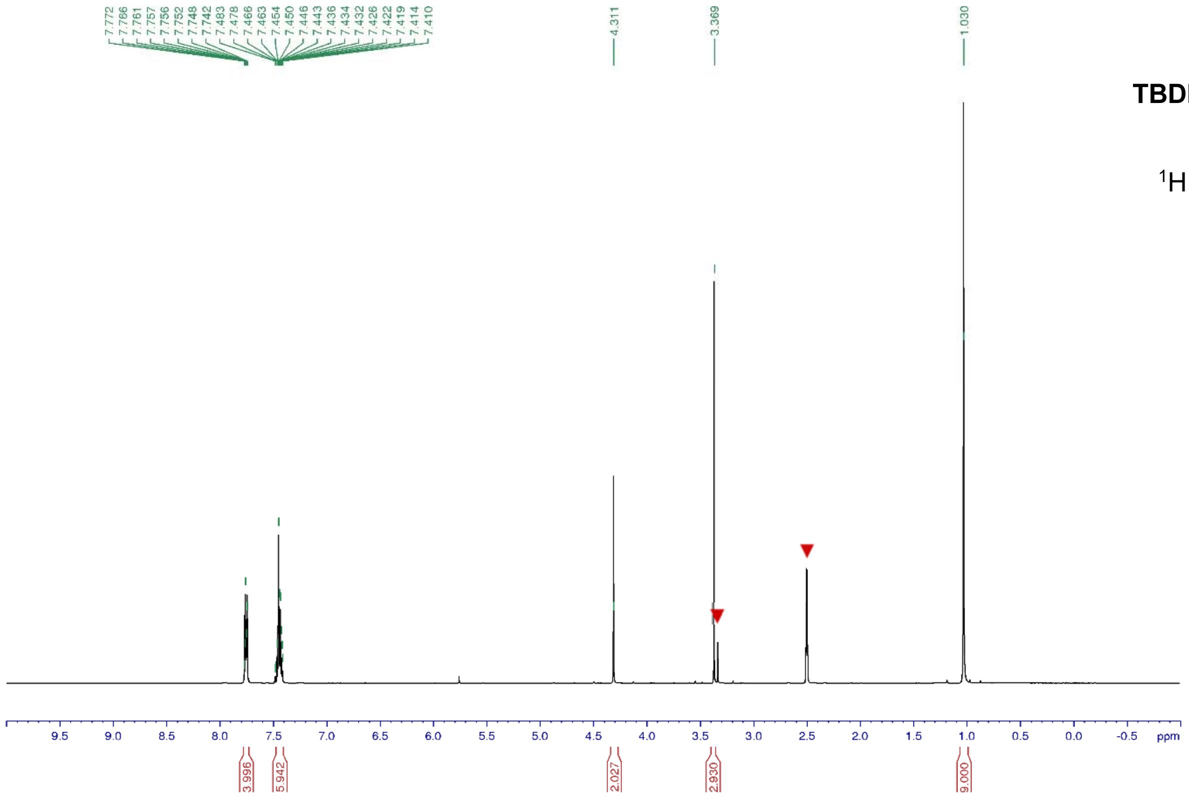

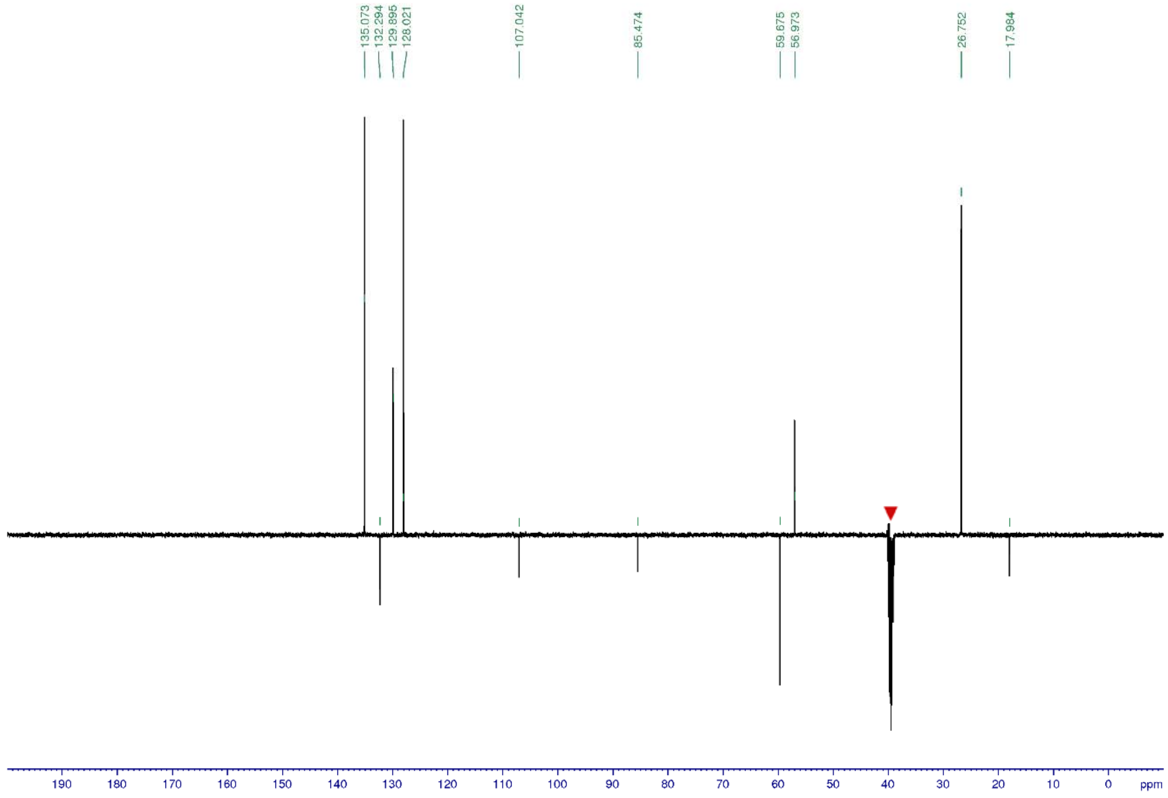

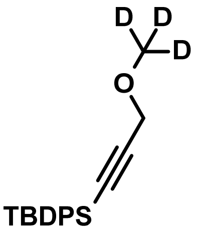

17
^1H, ^{13}C NMR

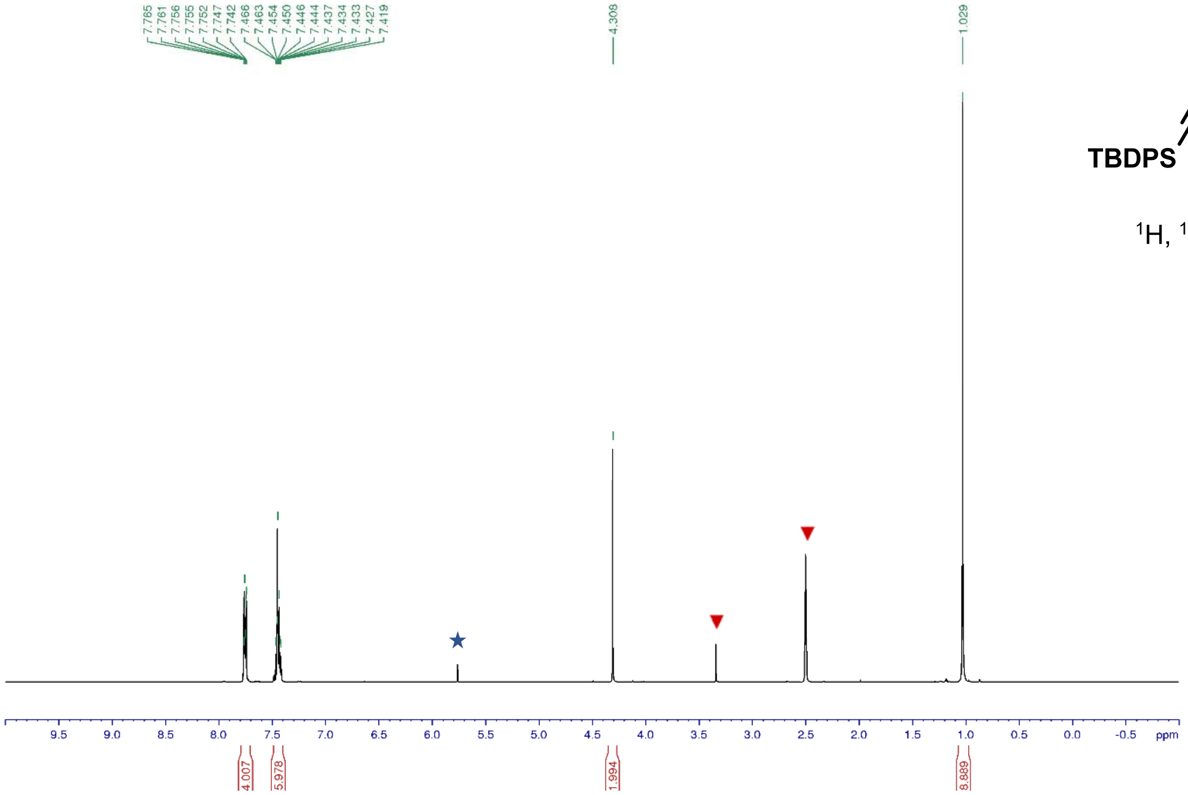

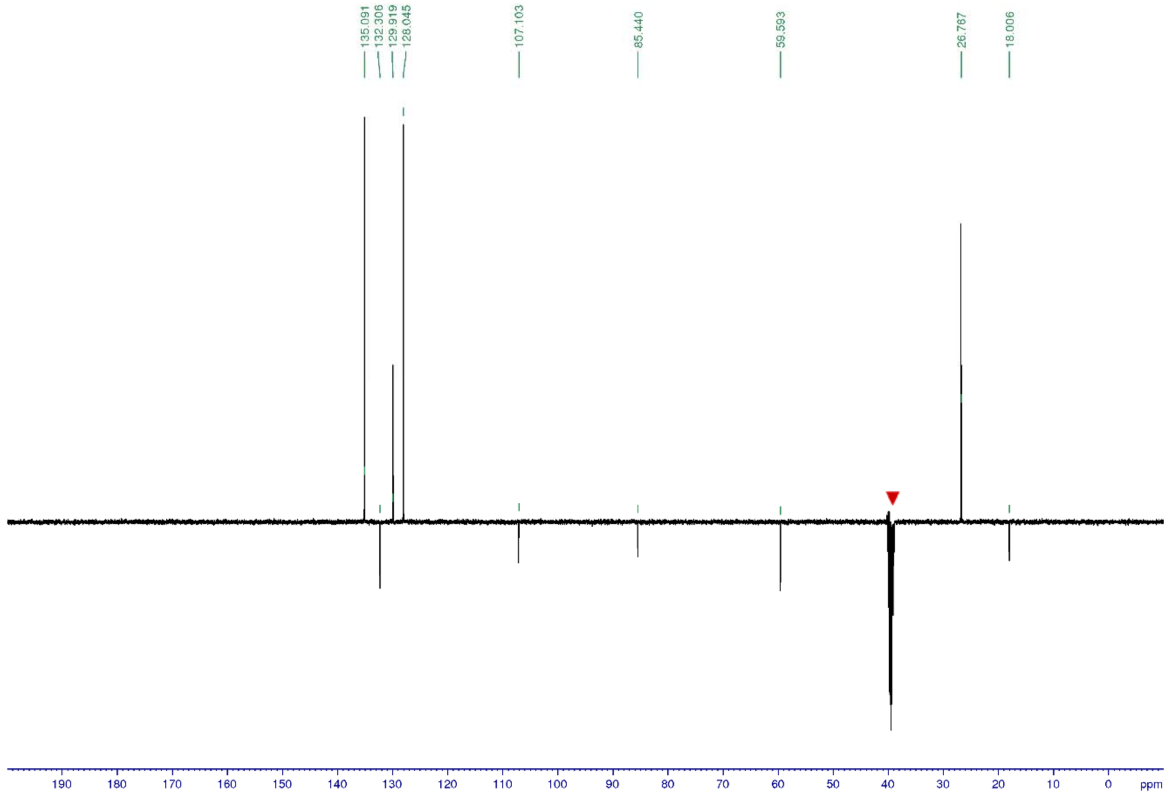

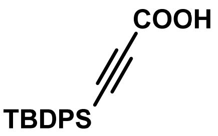
COOH
TBDPS

18

^1H, ^{13}C NMR

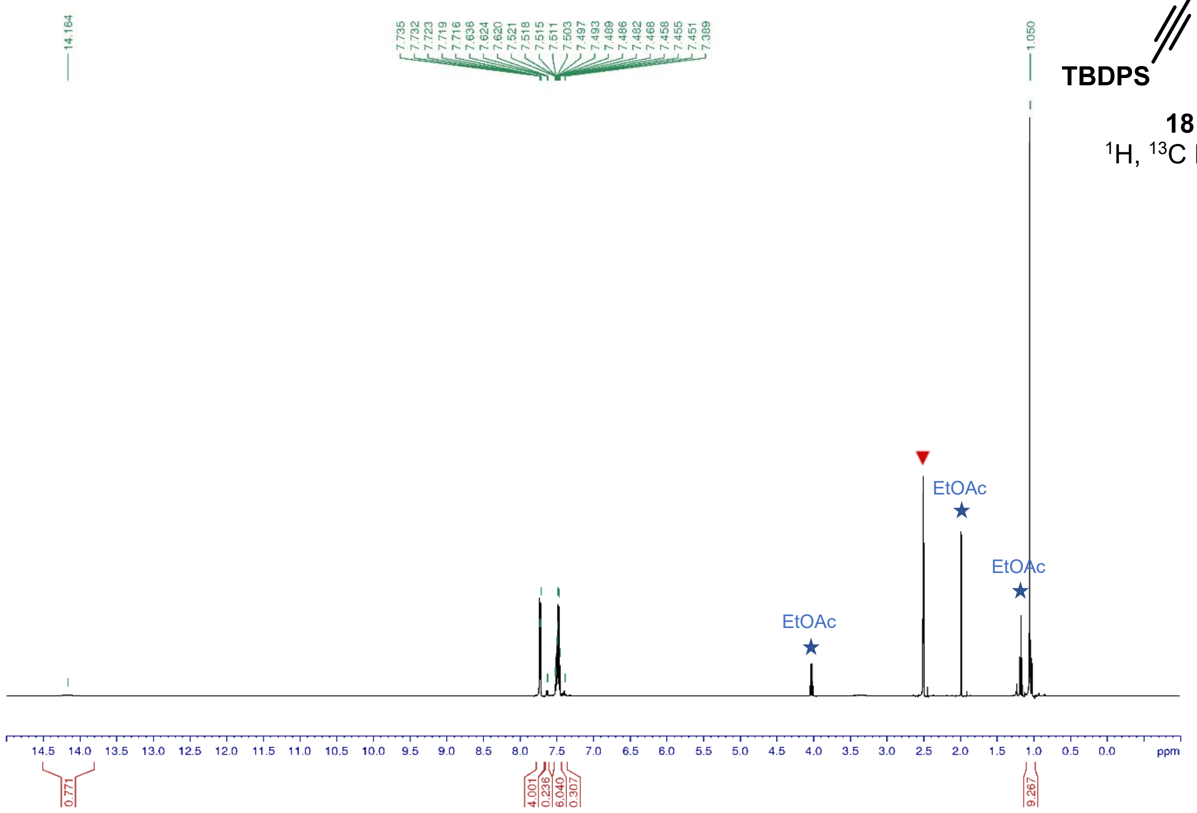
14.164
7.735 7.732 7.723 7.719 7.716 7.636 7.624 7.620 7.521 7.518 7.515 7.511 7.503 7.497 7.493 7.489 7.486 7.482 7.468 7.458 7.455 7.451 7.389
1.050
EtOAc
EtOAc
EtOAc
0.771
4.001
0.236
6.040
0.307
9.267
ppm

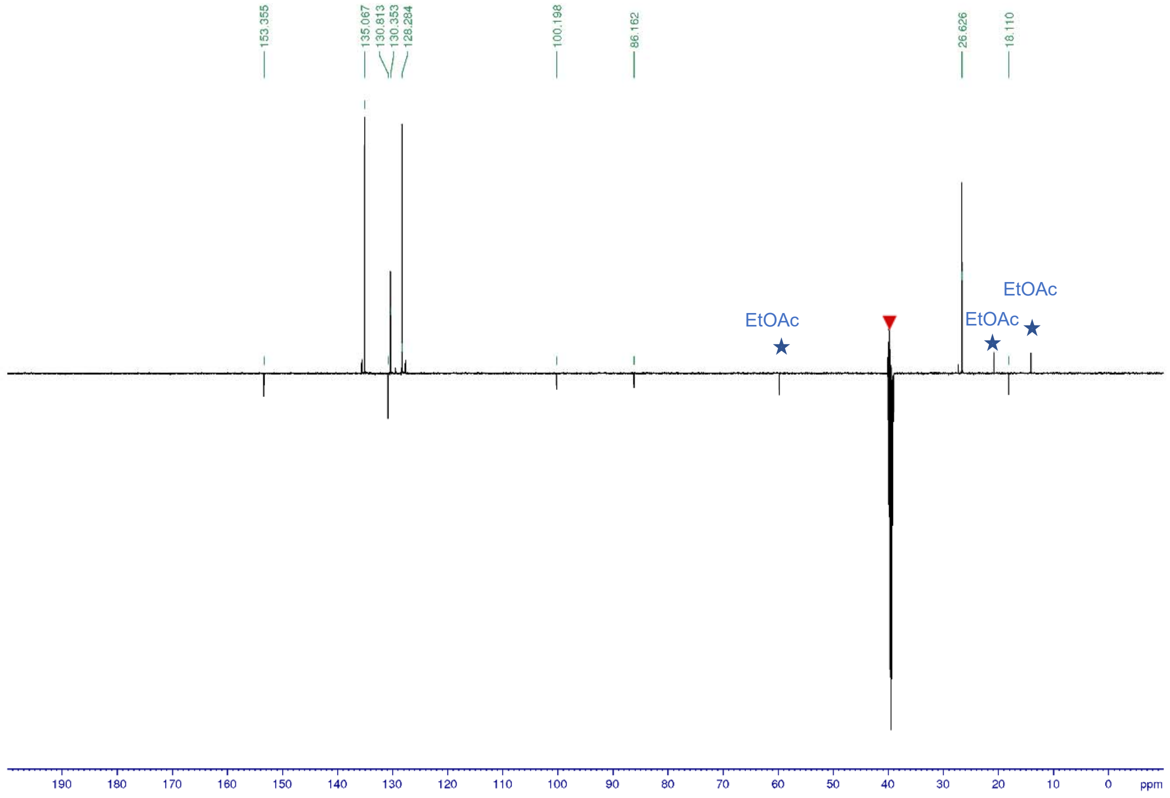
153.355
135.067
130.813
130.353
128.284
100.198
86.162
26.626
18.110
EtOAc
EtOAc
EtOAc
ppm

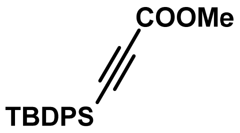

19

^1H, ^{13}C NMR

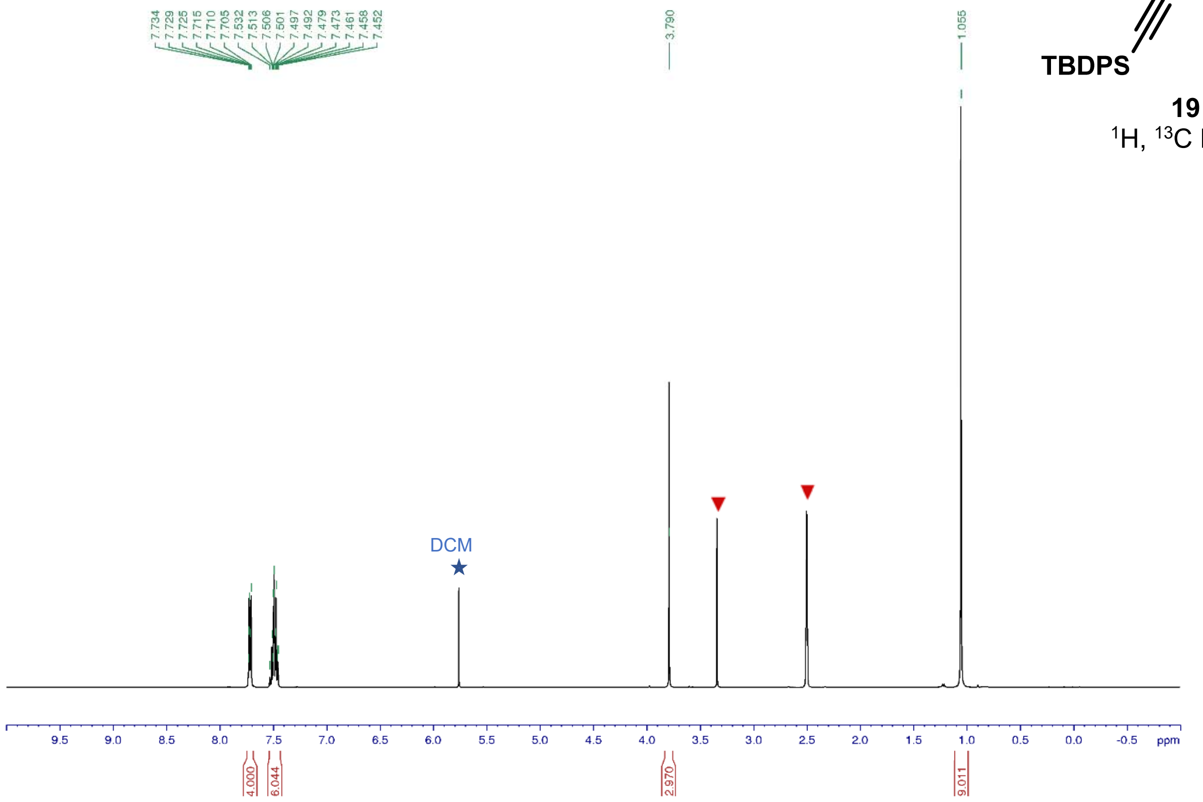
7.734
7.729
7.725
7.715
7.710
7.705
7.532
7.513
7.506
7.501
7.497
7.492
7.479
7.473
7.461
7.458
7.452
3.790
1.055
DCM
4.000
6.044
2.970
9.011
ppm

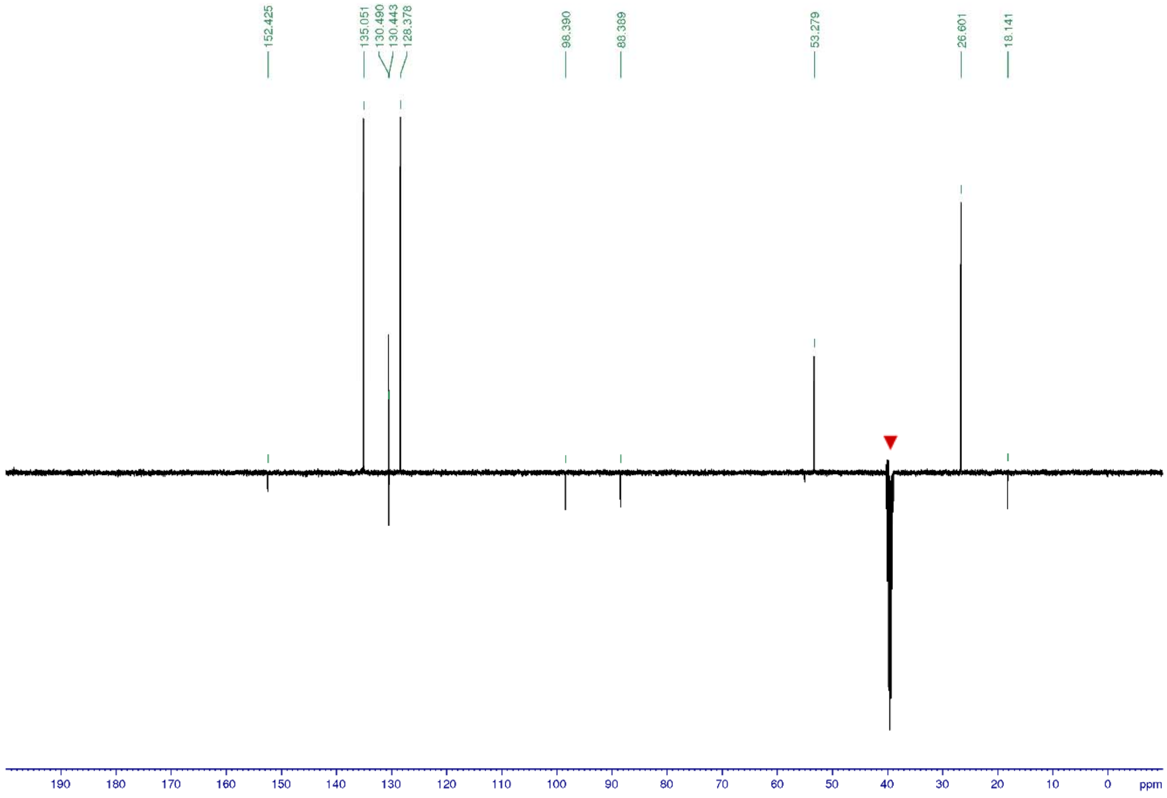
152.425
135.051
130.490
130.443
128.378
98.390
88.389
53.279
26.601
18.141
ppm

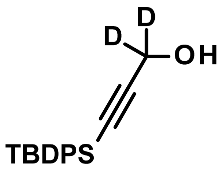
D
D
OH
TBDPS

9
^{1}H, ^{13}C NMR

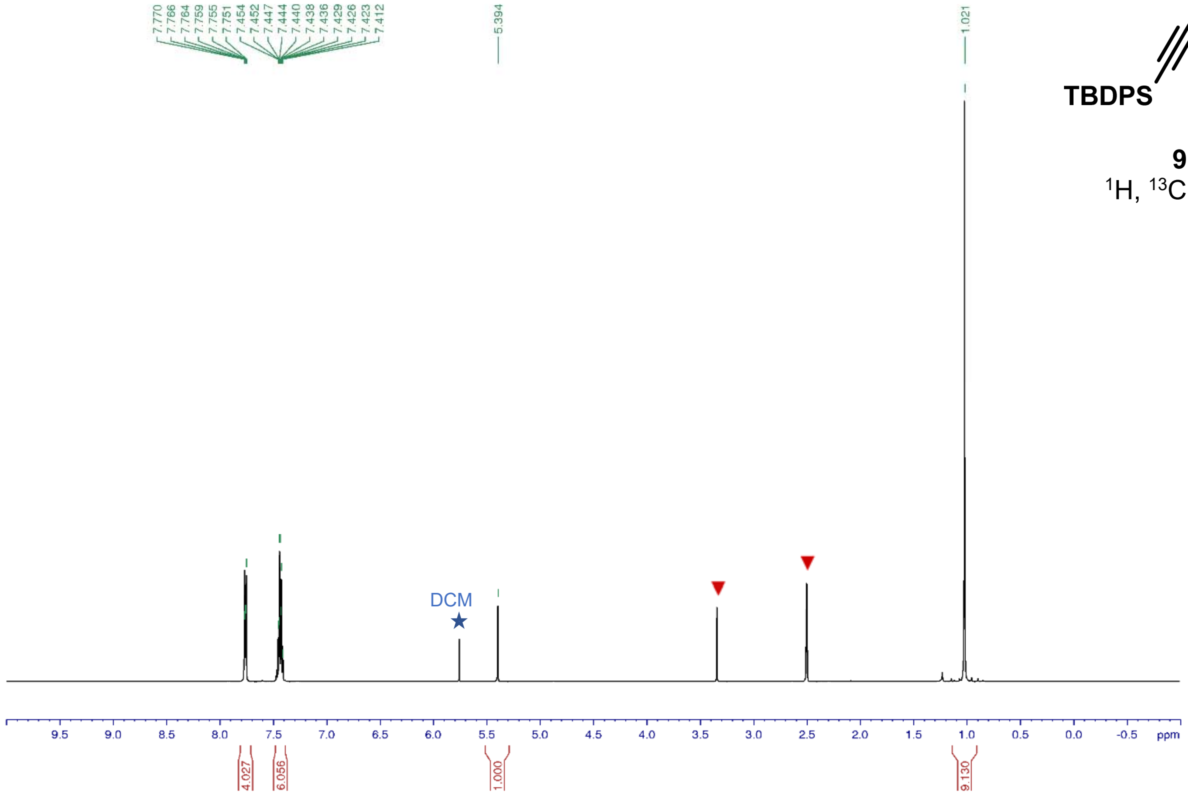
7.770
7.766
7.764
7.759
7.755
7.751
7.454
7.452
7.447
7.444
7.440
7.438
7.436
7.429
7.426
7.423
7.412
5.394
1.021
DCM
4.027
6.056
1.000
9.130
ppm

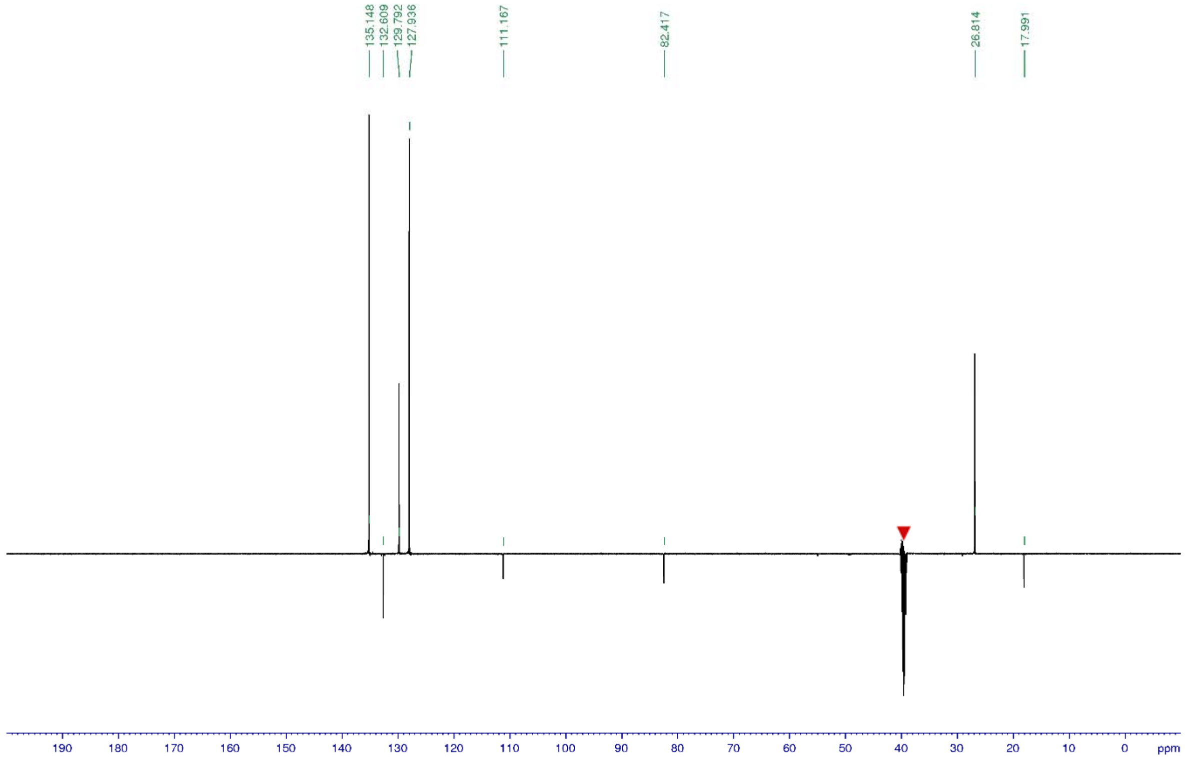
135.148
132.609
129.792
127.936
111.167
82.417
26.814
17.991
ppm

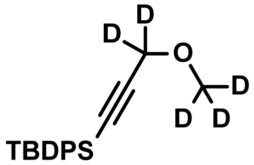
D
D
O
D
D
D
TBDPS

20
^{1}H, ^{13}C NMR

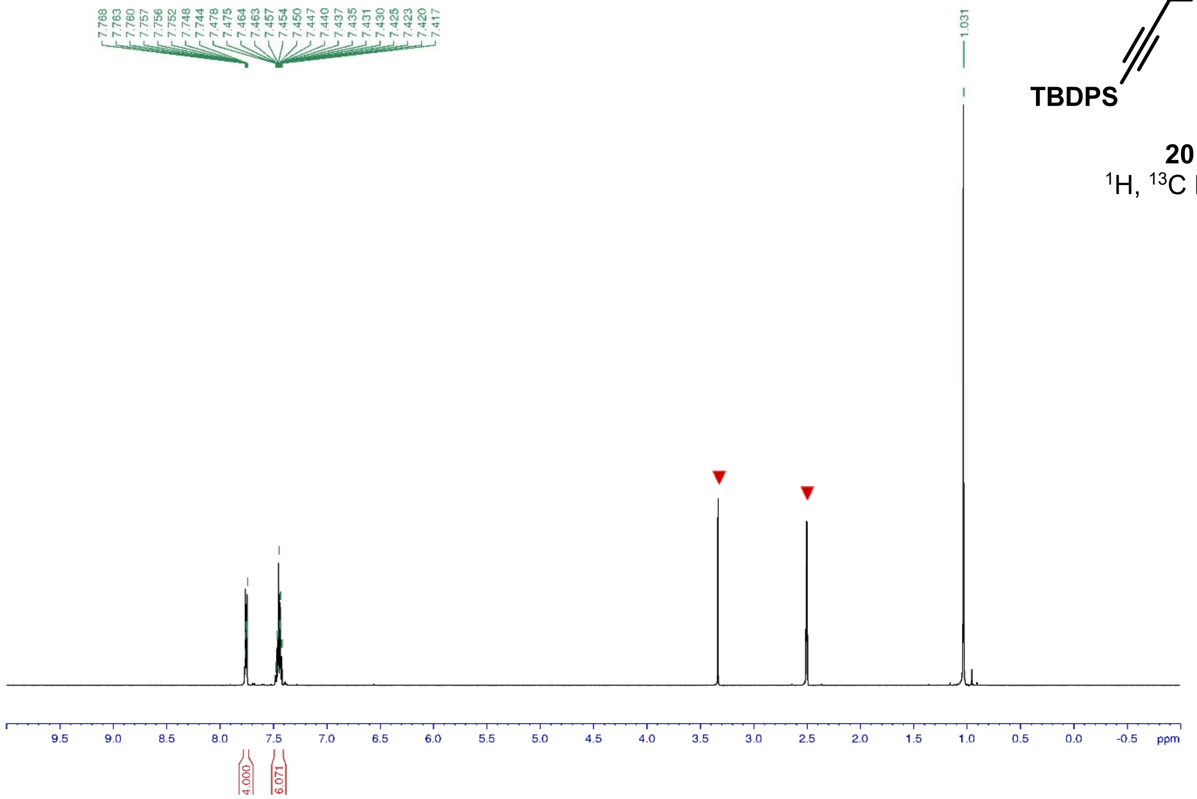
7.768
7.763
7.760
7.757
7.756
7.752
7.748
7.744
7.478
7.475
7.464
7.463
7.457
7.454
7.450
7.447
7.440
7.437
7.435
7.431
7.430
7.425
7.423
7.420
7.417
1.031
4.000
6.071
ppm

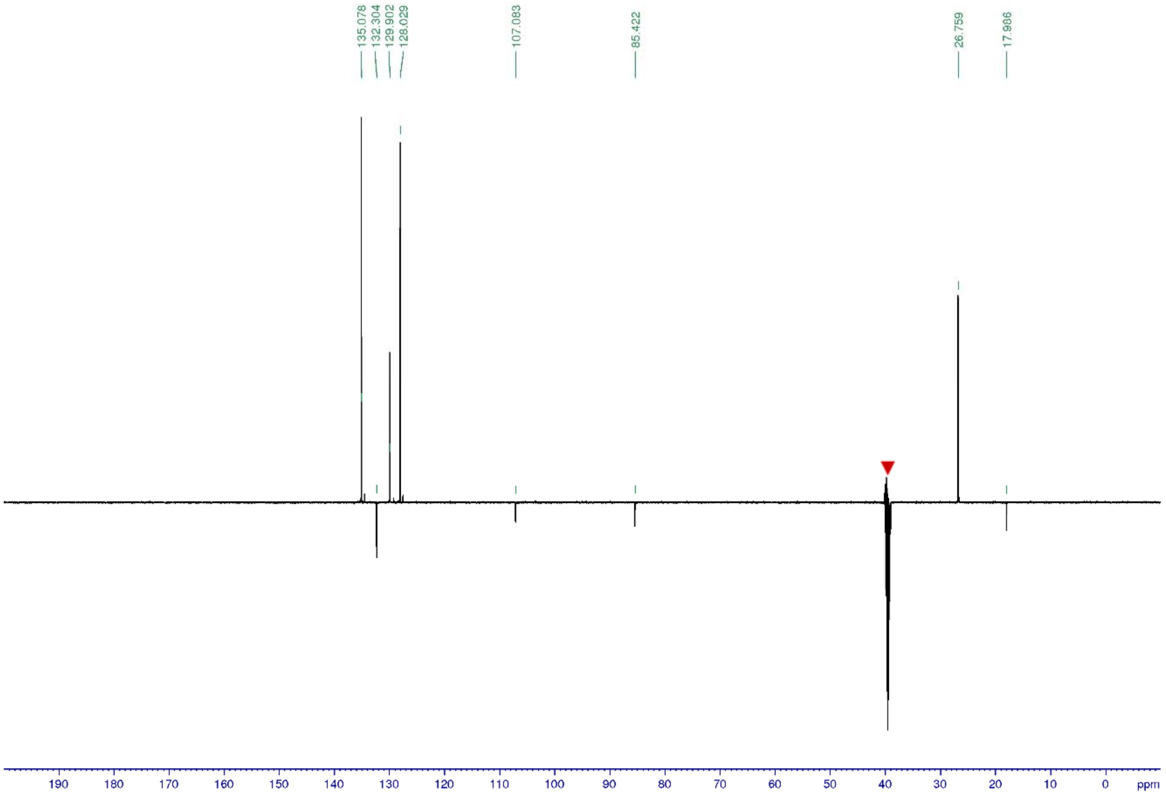
135.078
132.304
129.902
128.029
107.083
85.422
26.759
17.986
ppm

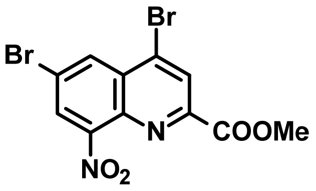
Br
Br
N
COOMe
NO_2

4a

^{1}H, ^{13}C NMR

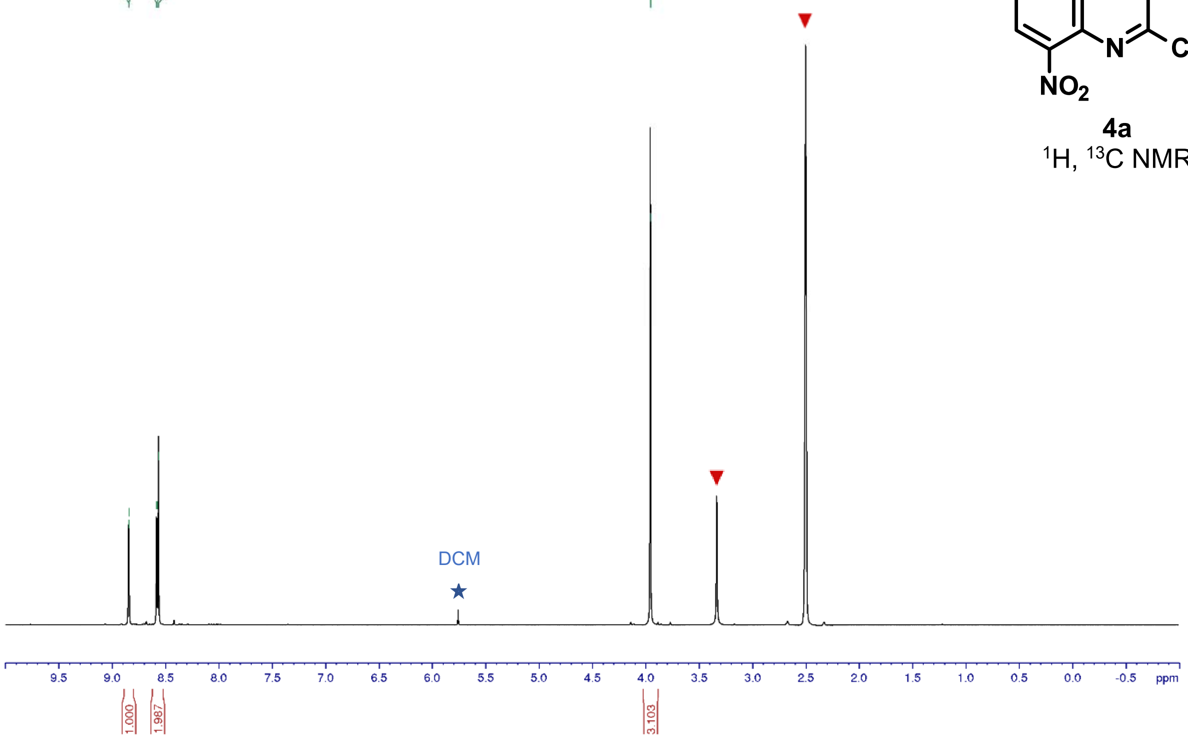
8.845
8.841
8.584
8.579
8.564
3.955
DCM
1.000
1.987
3.103
ppm

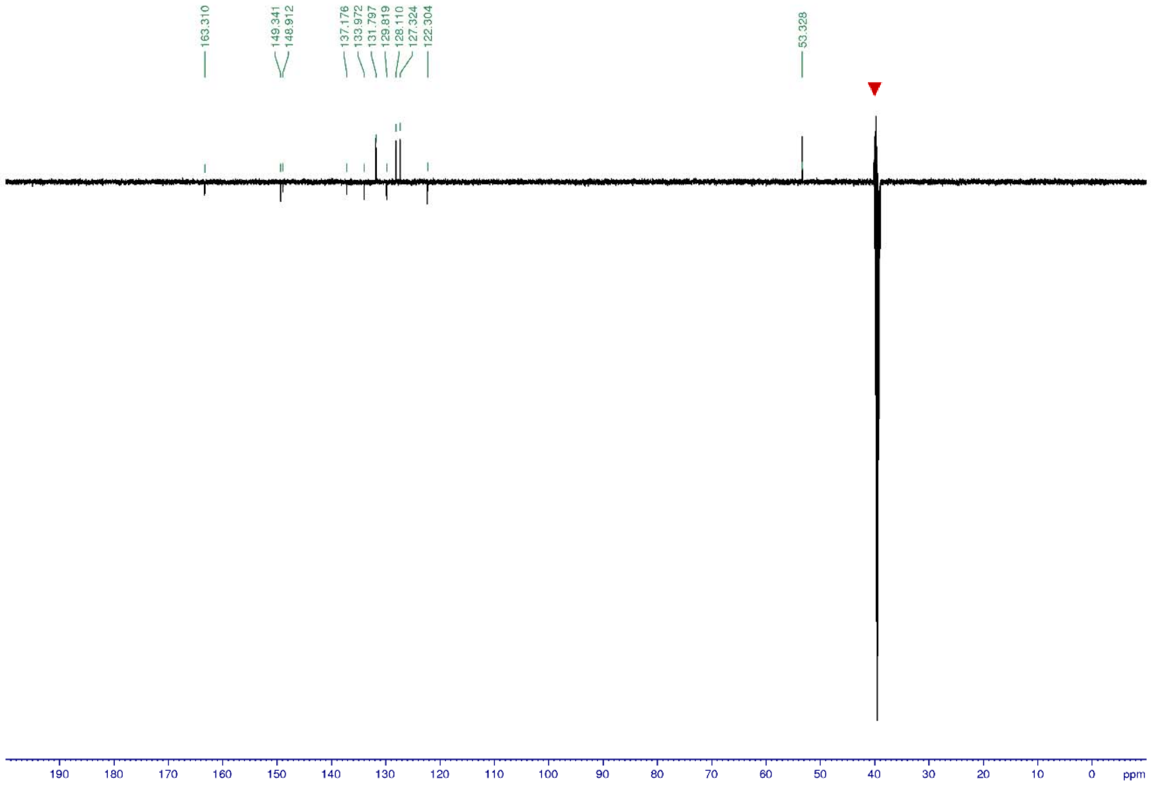
163.310
149.341
148.912
137.176
133.972
131.797
129.819
128.110
127.324
122.304
53.328
ppm

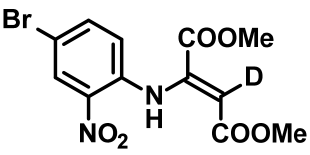
Br
COOMe
D
N
H
NO2
COOMe

2b

^{1}H, ^{13}C NMR

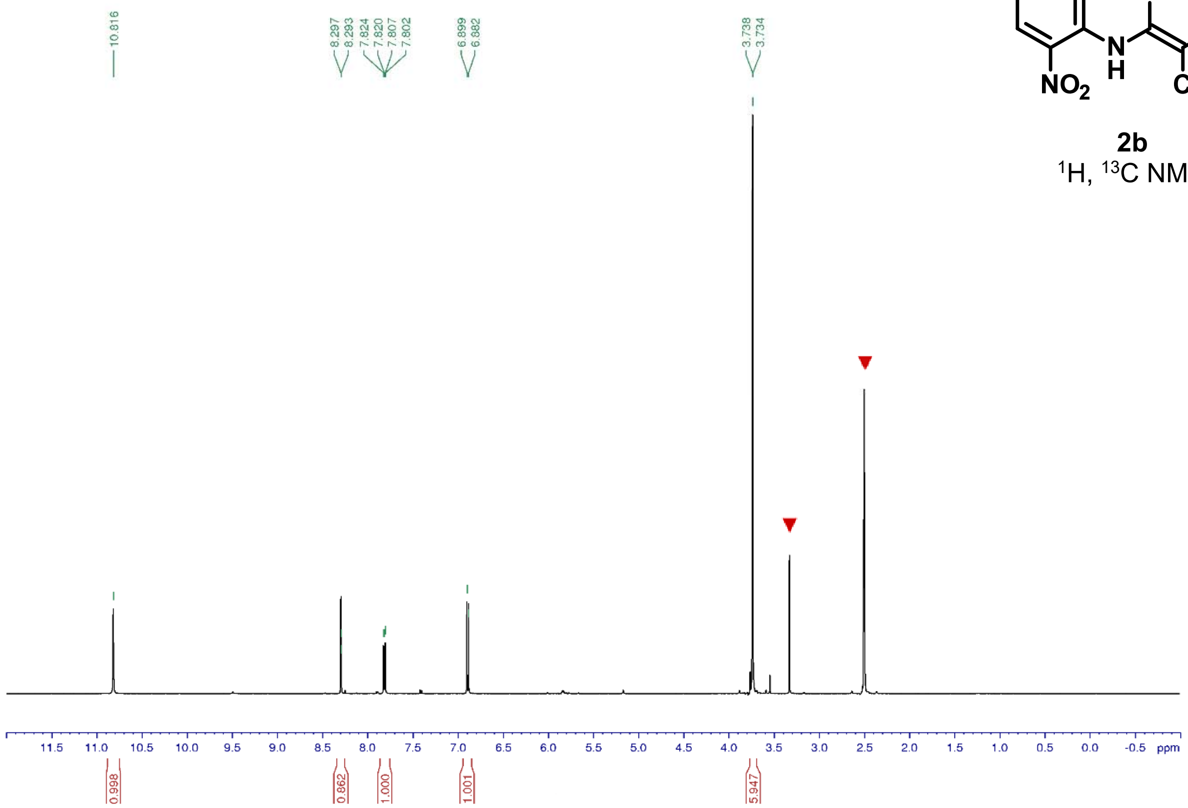
10.816
8.297
8.293
7.824
7.820
7.807
7.802
6.899
6.882
3.738
3.734
11.5 11.0 10.5 10.0 9.5 9.0 8.5 8.0 7.5 7.0 6.5 6.0 5.5 5.0 4.5 4.0 3.5 3.0 2.5 2.0 1.5 1.0 0.5 0.0 -0.5 ppm
0.998
0.862
1.000
1.001
5.947

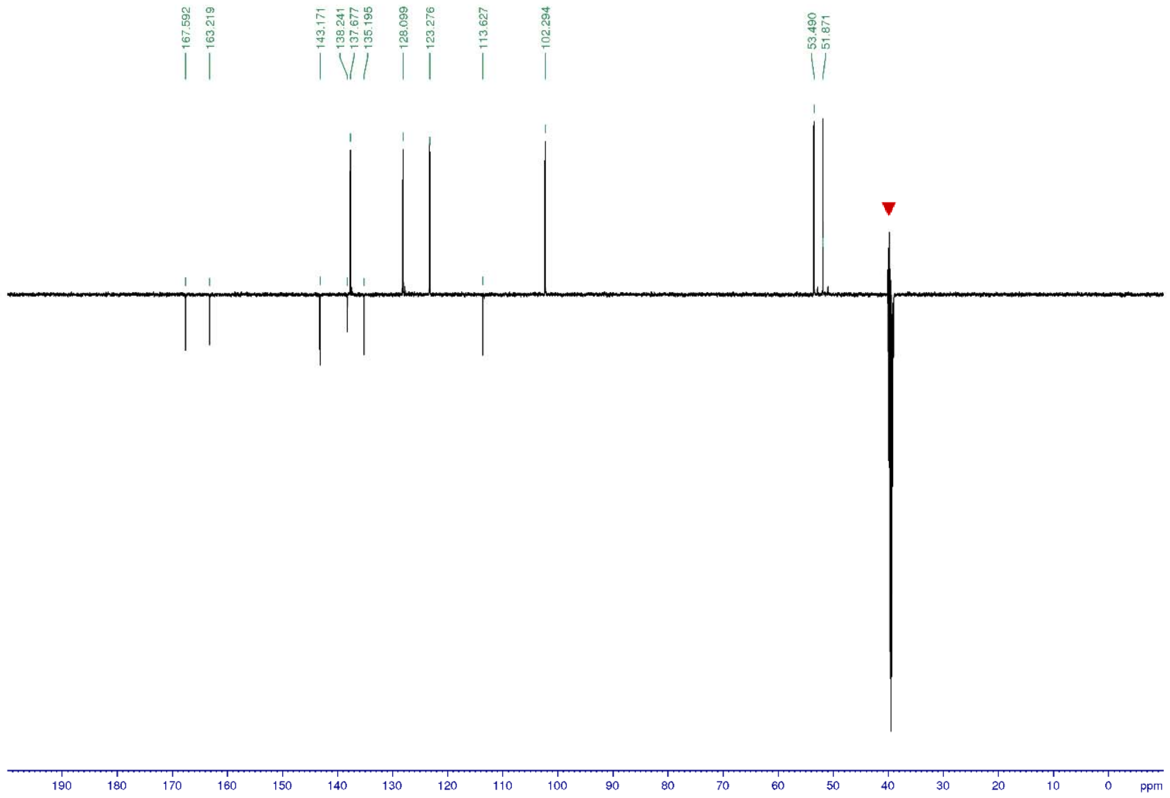
167.592
163.219
143.171
138.241
137.677
135.195
128.099
123.276
113.627
102.294
53.490
51.871
190 180 170 160 150 140 130 120 110 100 90 80 70 60 50 40 30 20 10 0 ppm

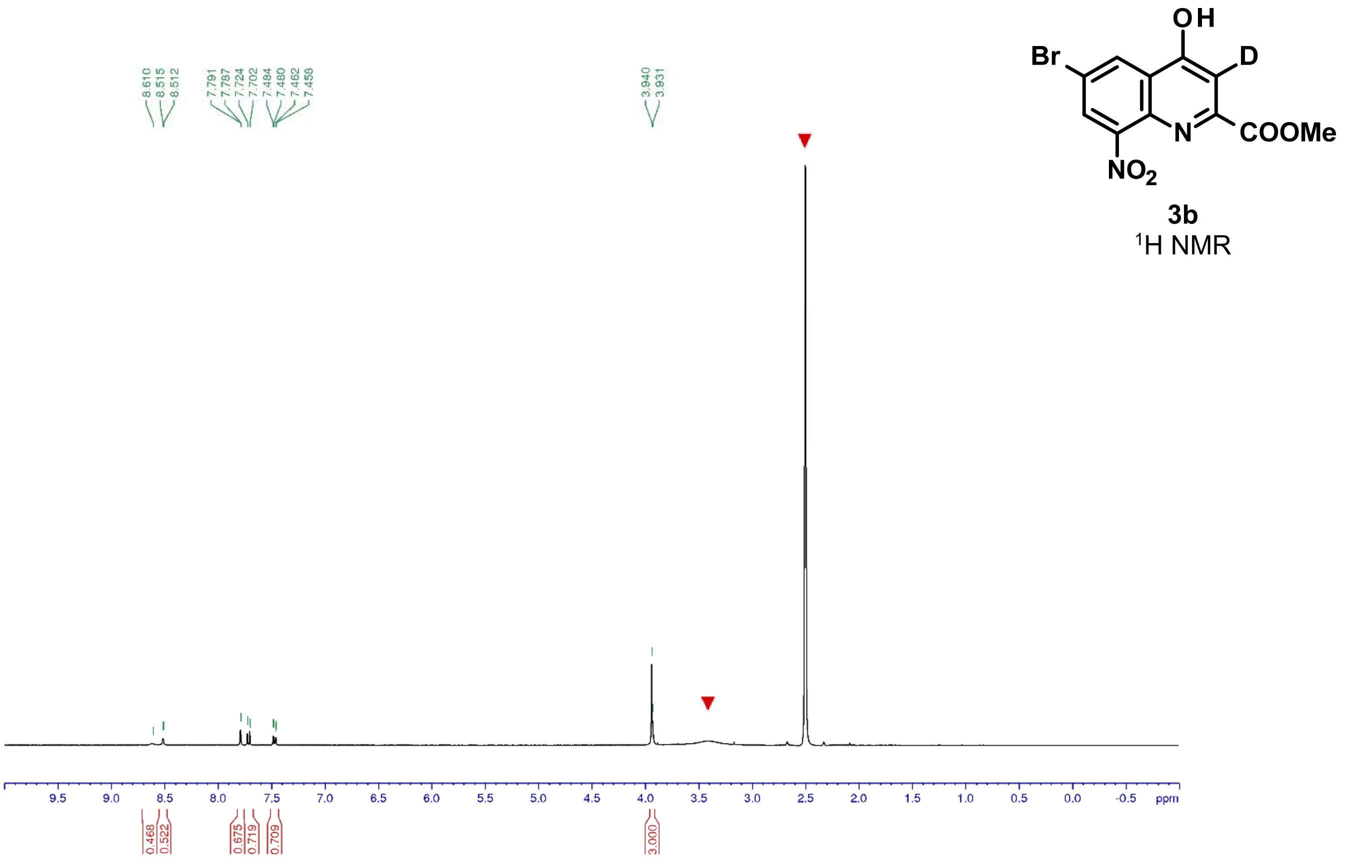

3b
^{1}H NMR

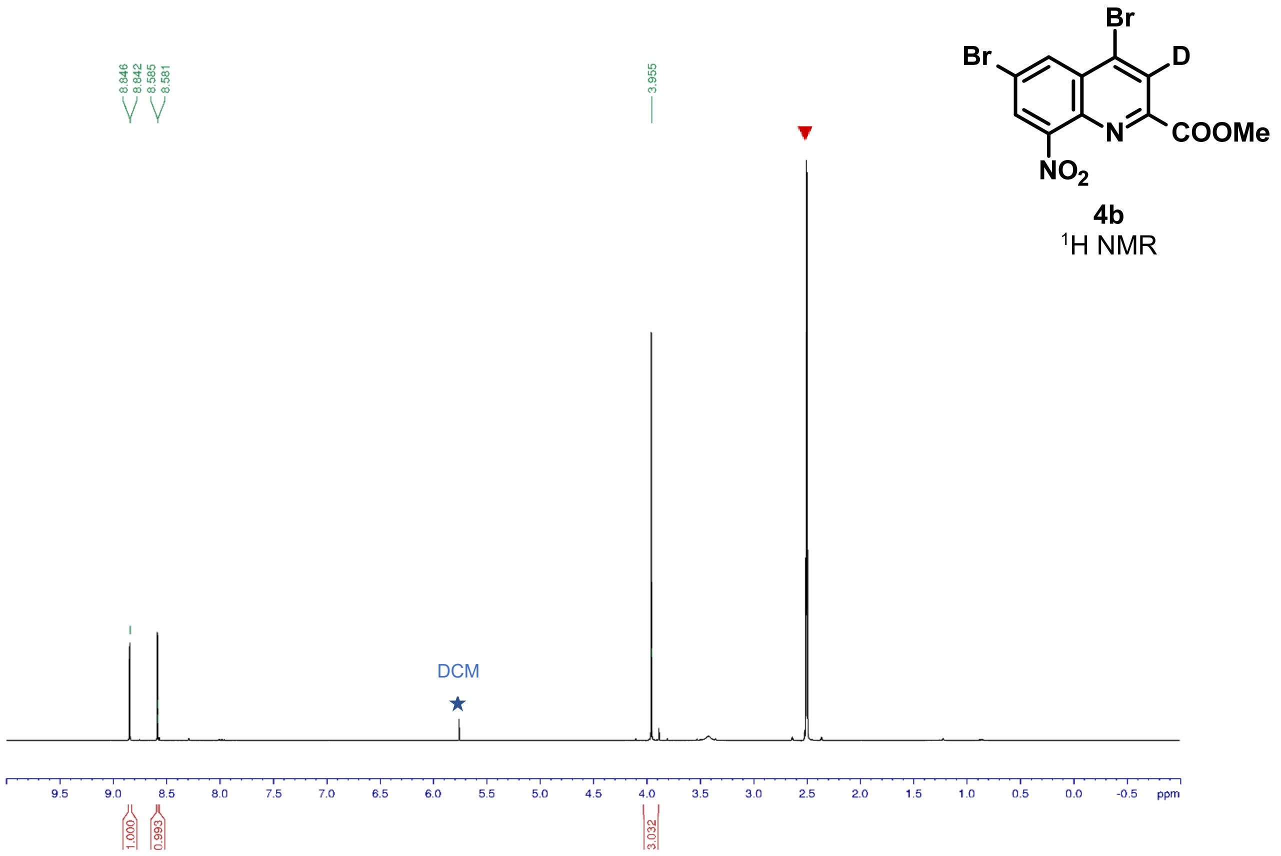

4b
^{1}H NMR
DCM

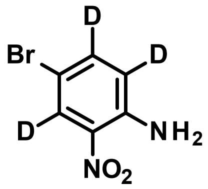
Br
D
D
D
NH2
NO2

5

^{1}H, ^{13}C NMR

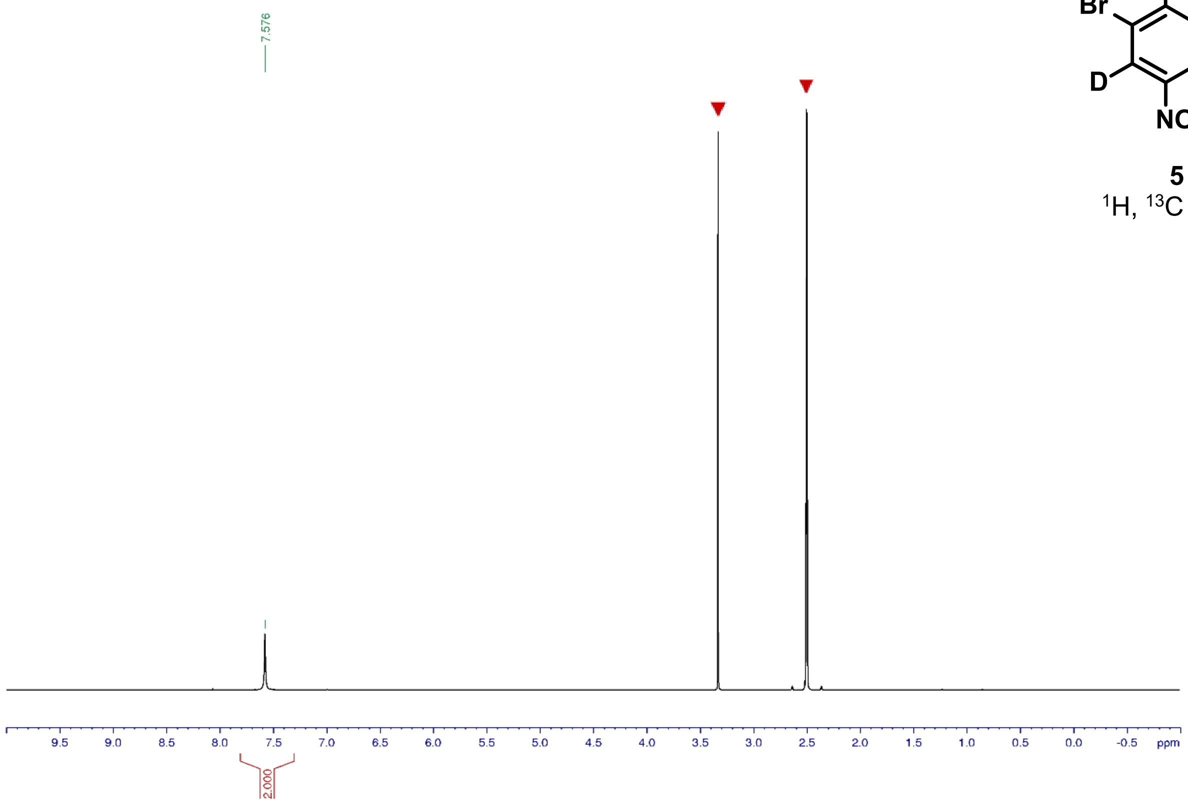
7.576
9.5 9.0 8.5 8.0 7.5 7.0 6.5 6.0 5.5 5.0 4.5 4.0 3.5 3.0 2.5 2.0 1.5 1.0 0.5 0.0 -0.5 ppm
2.000

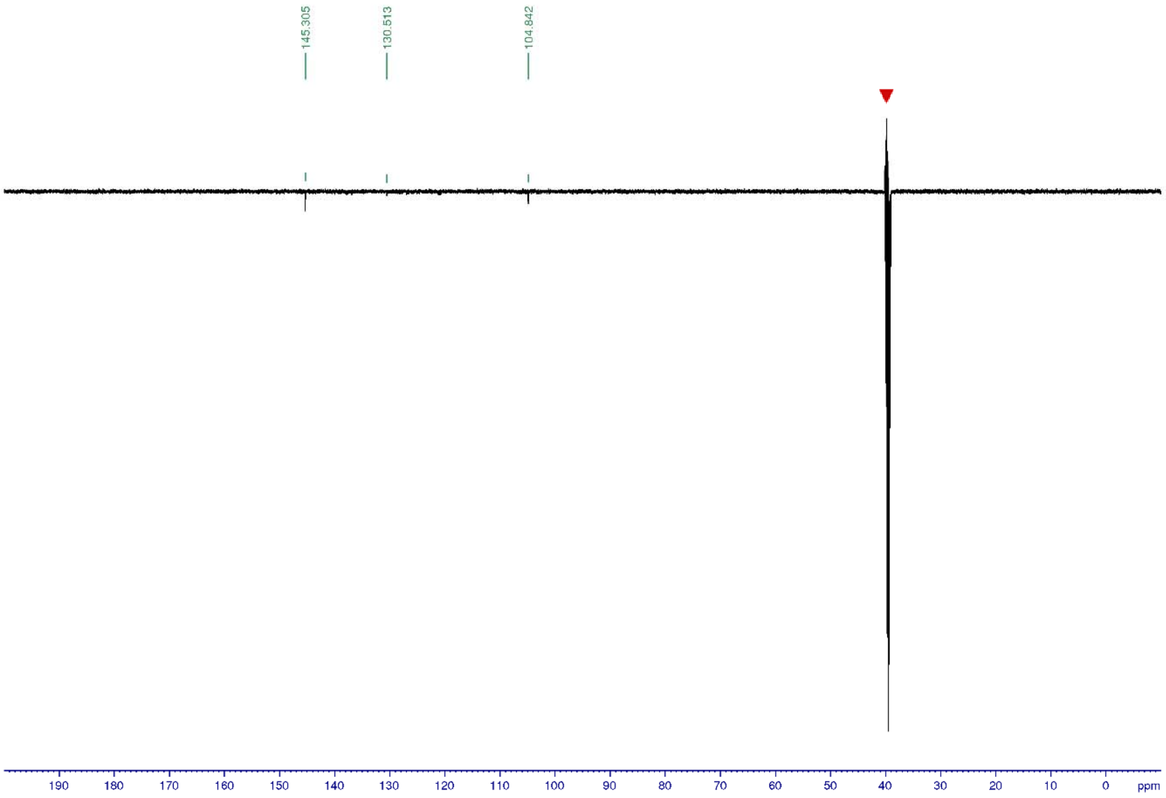
145.305
130.513
104.842
190 180 170 160 150 140 130 120 110 100 90 80 70 60 50 40 30 20 10 0 ppm

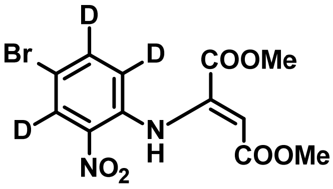
Br
D
D
COOMe
D
N
H
NO2
COOMe

6a

^1H, ^{13}C NMR

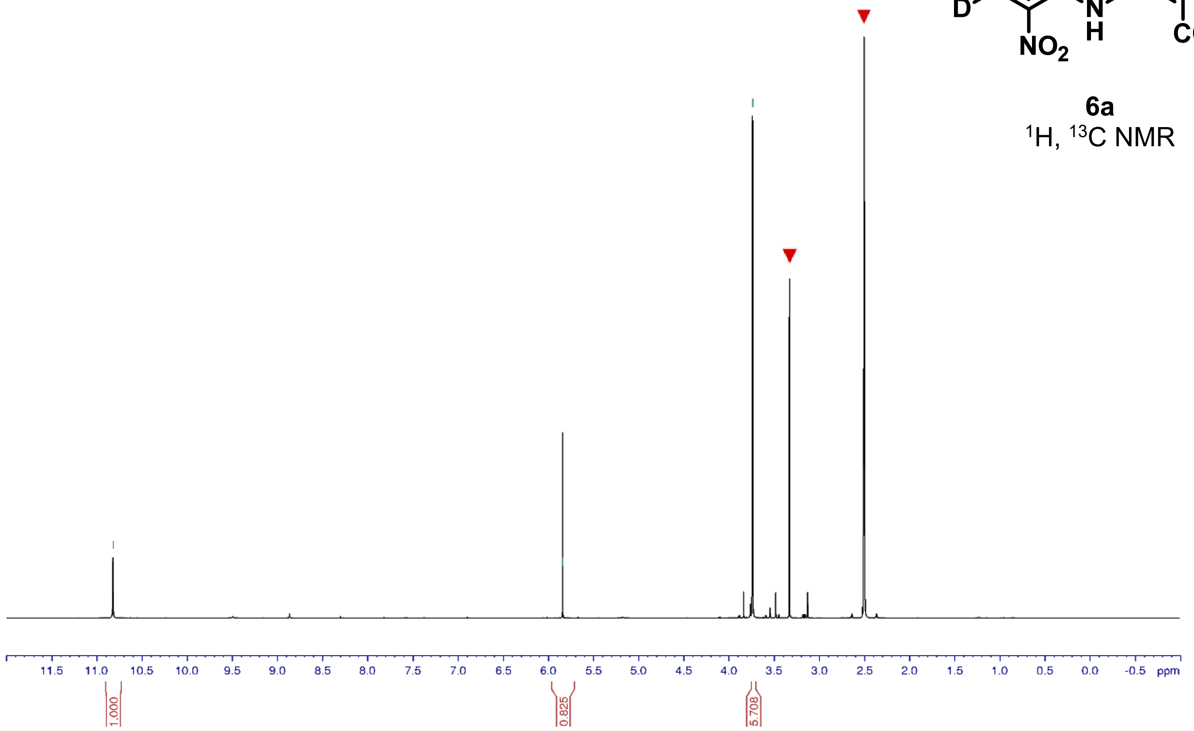
10.820
5.839
3.739
3.734
11.5 11.0 10.5 10.0 9.5 9.0 8.5 8.0 7.5 7.0 6.5 6.0 5.5 5.0 4.5 4.0 3.5 3.0 2.5 2.0 1.5 1.0 0.5 0.0 -0.5 ppm
1.000
0.825
5.708

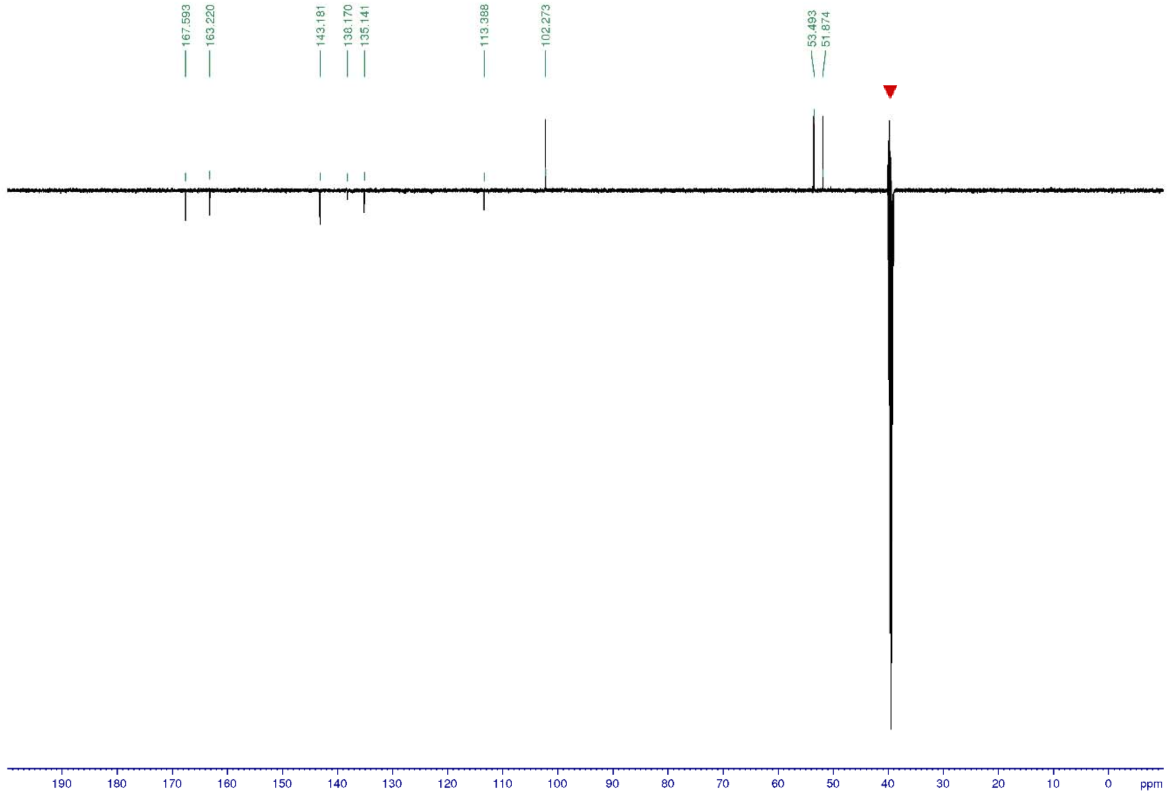
167.593
163.220
143.181
138.170
135.141
113.388
102.273
53.493
51.874
190 180 170 160 150 140 130 120 110 100 90 80 70 60 50 40 30 20 10 0 ppm

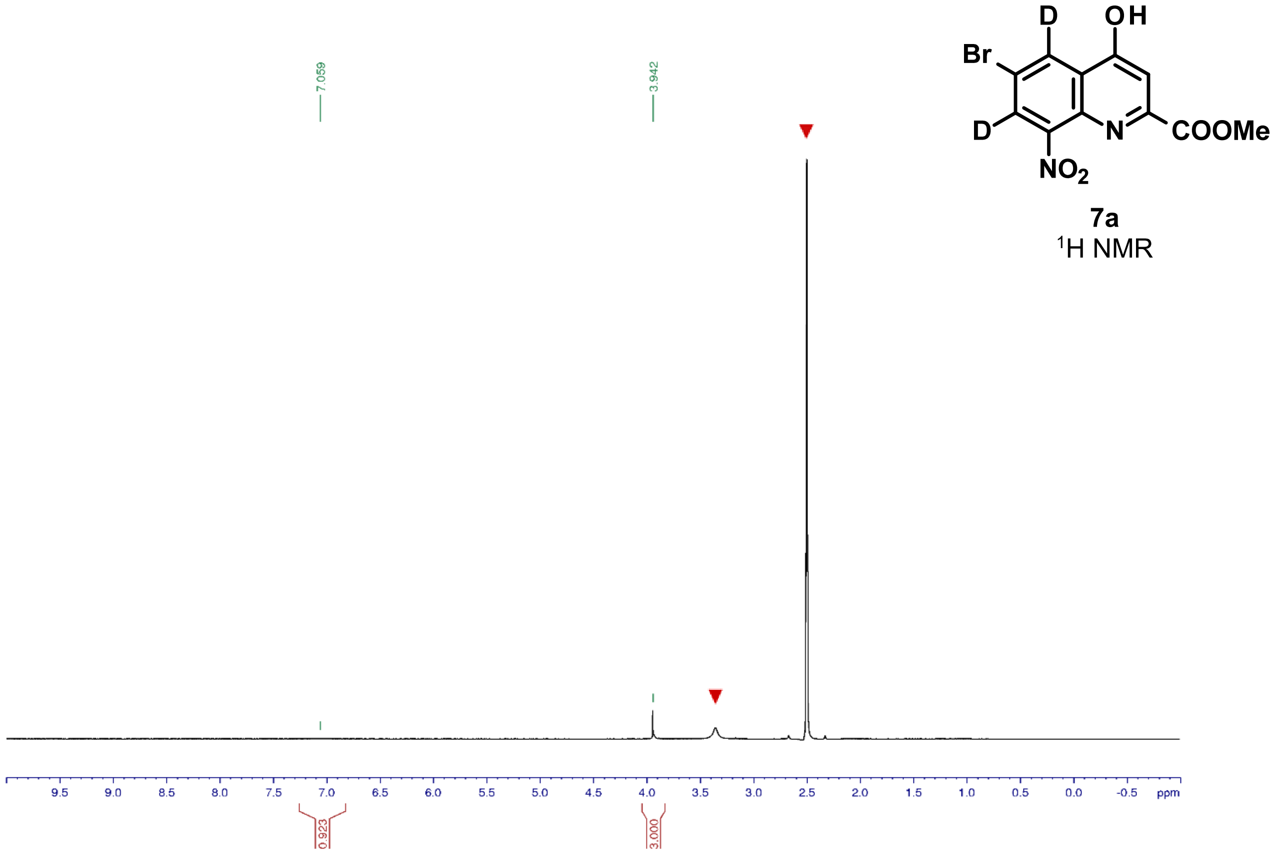

7a
^{1}H NMR

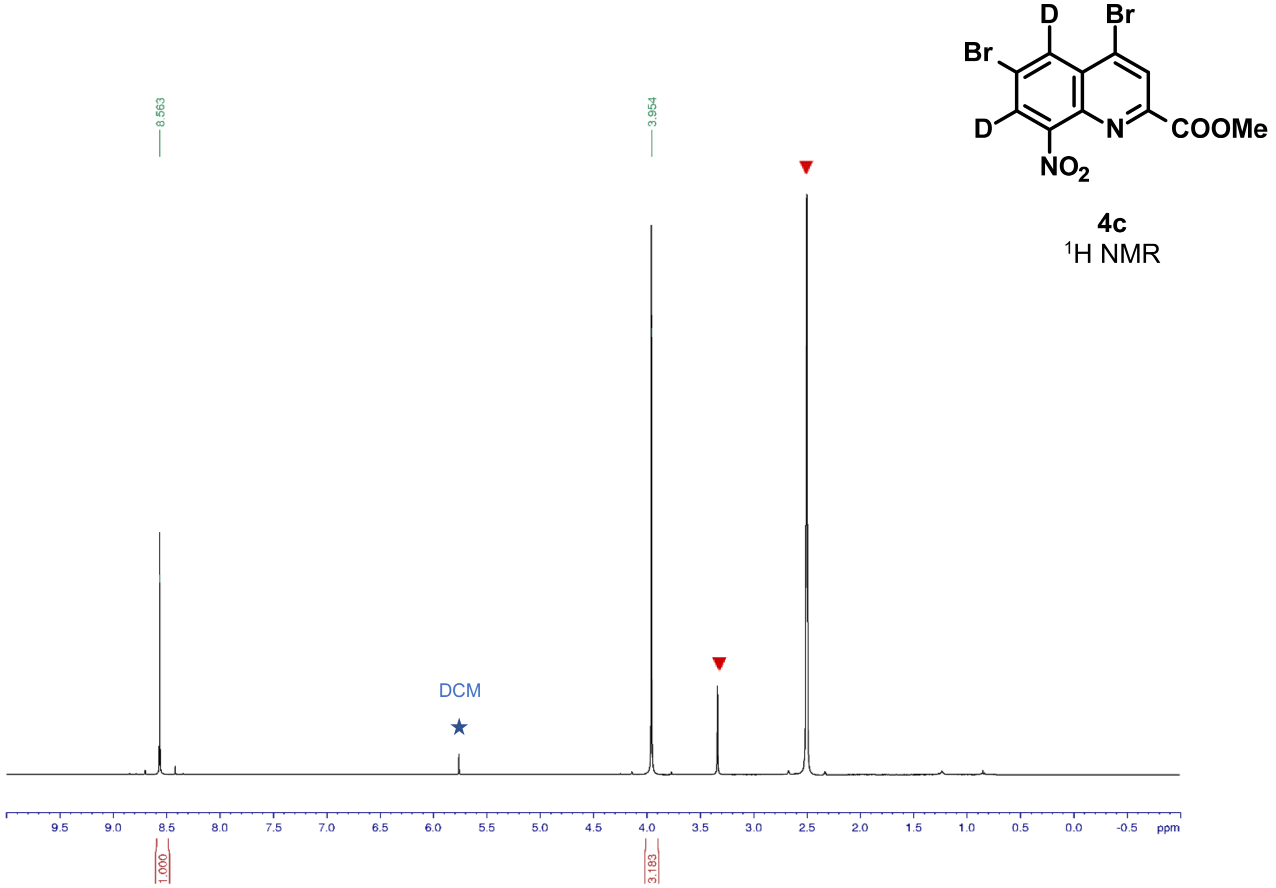

4c
^{1}H NMR

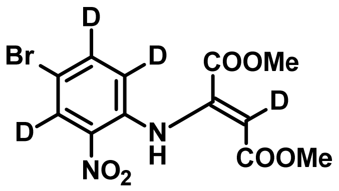

6b

^1H, ^{13}C NMR

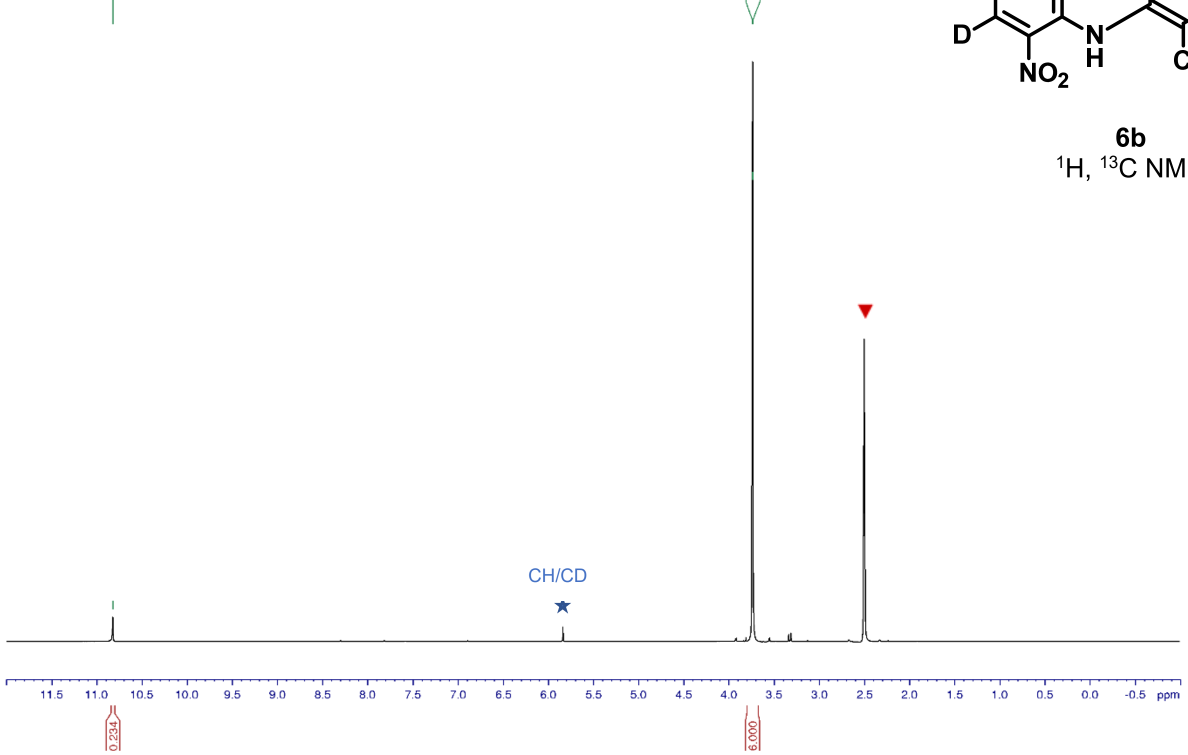
10.822
3.740
3.733
CH/CD
0.234
6.000
11.5 11.0 10.5 10.0 9.5 9.0 8.5 8.0 7.5 7.0 6.5 6.0 5.5 5.0 4.5 4.0 3.5 3.0 2.5 2.0 1.5 1.0 0.5 0.0 -0.5 ppm

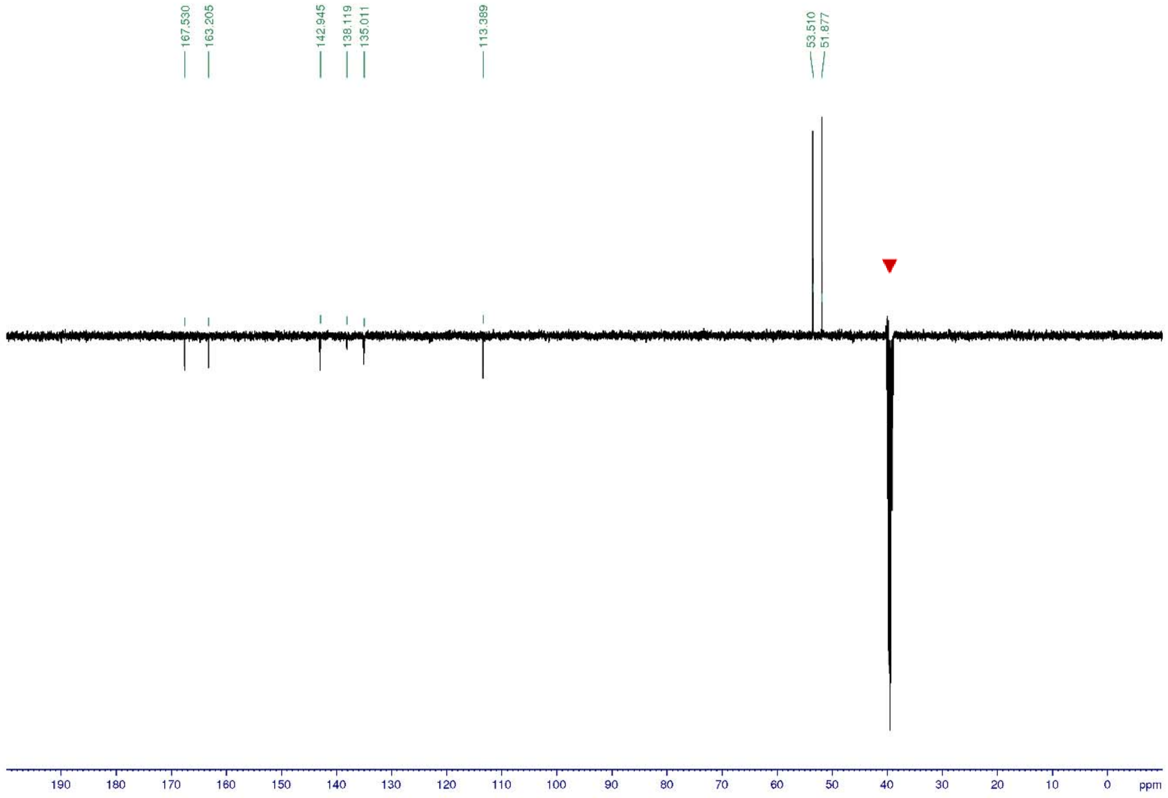
167.530
163.205
142.945
138.119
135.011
113.389
53.510
51.877
190 180 170 160 150 140 130 120 110 100 90 80 70 60 50 40 30 20 10 0 ppm

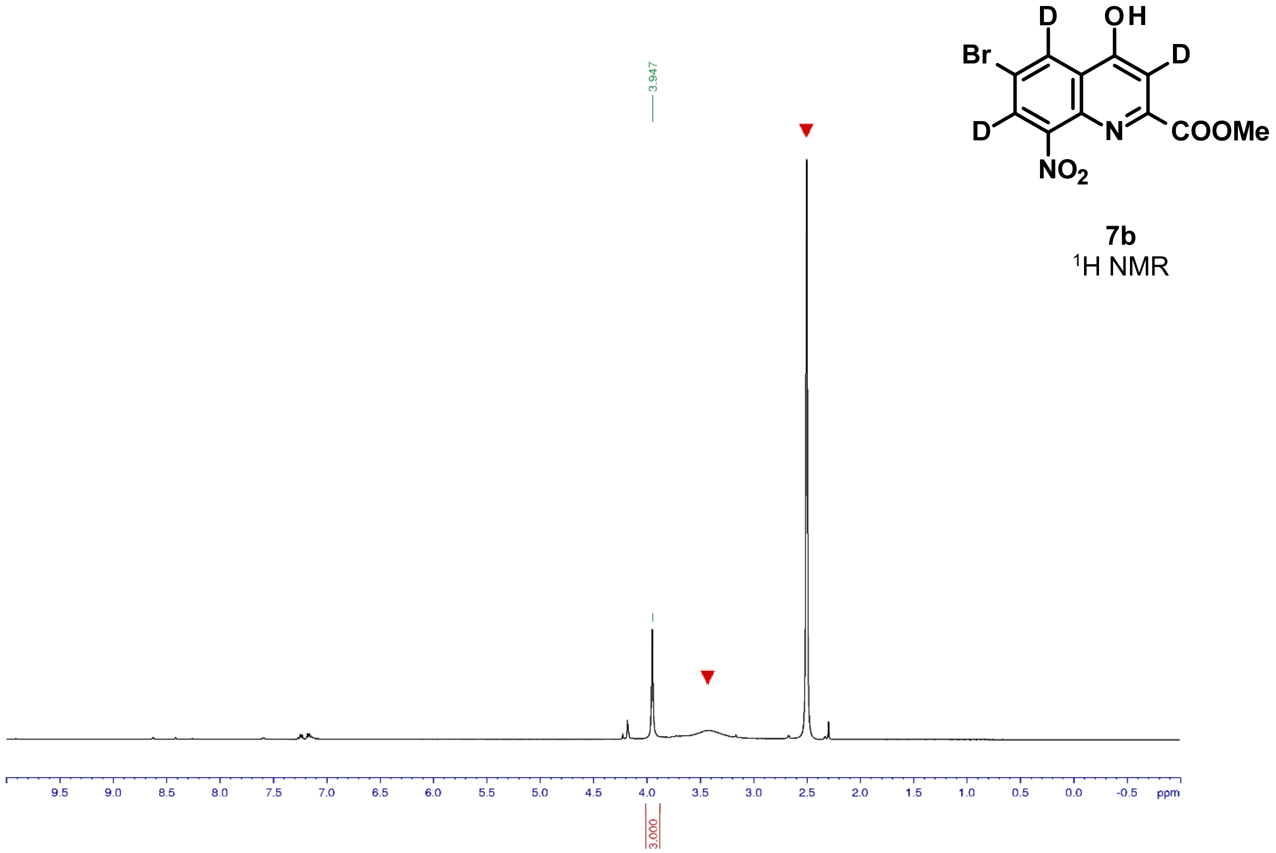

7b
^{1}H NMR

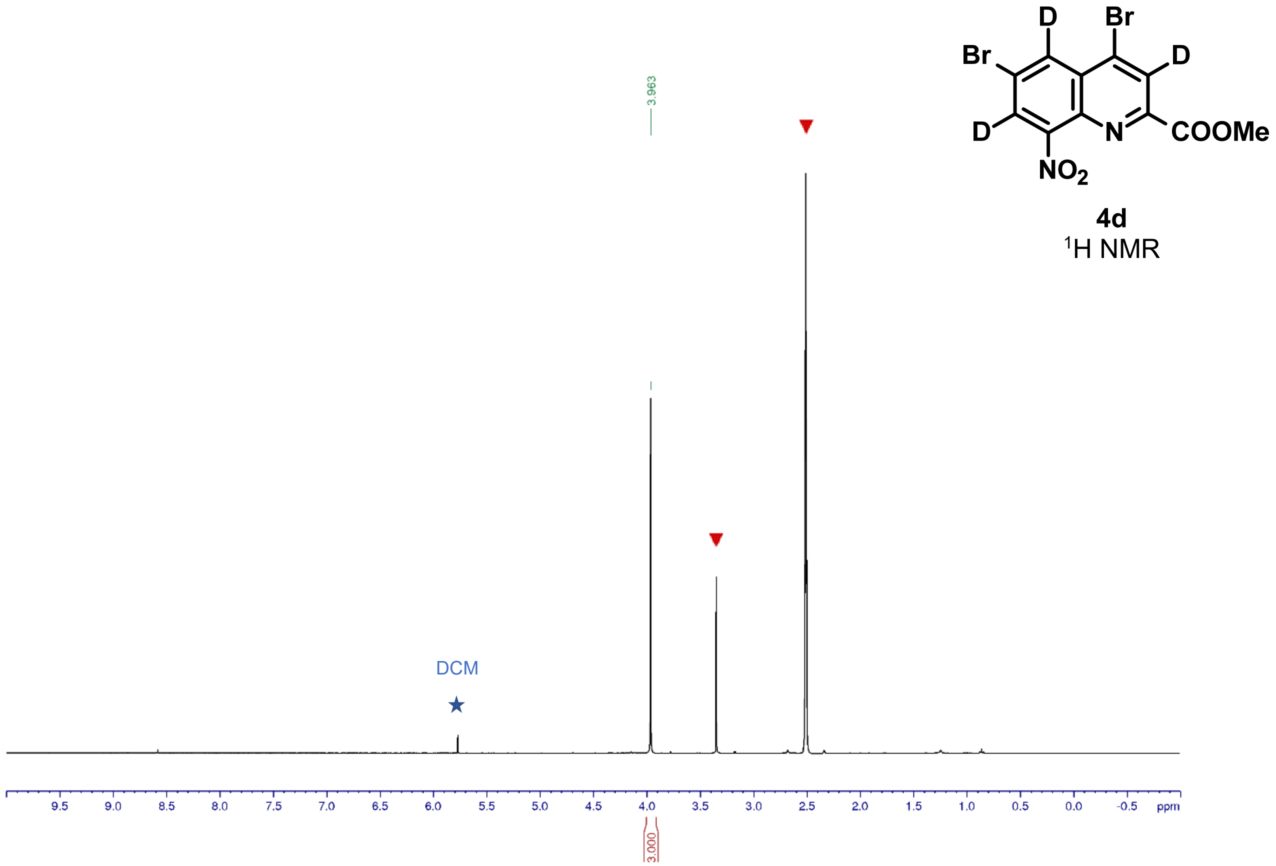

4d
^{1}H NMR

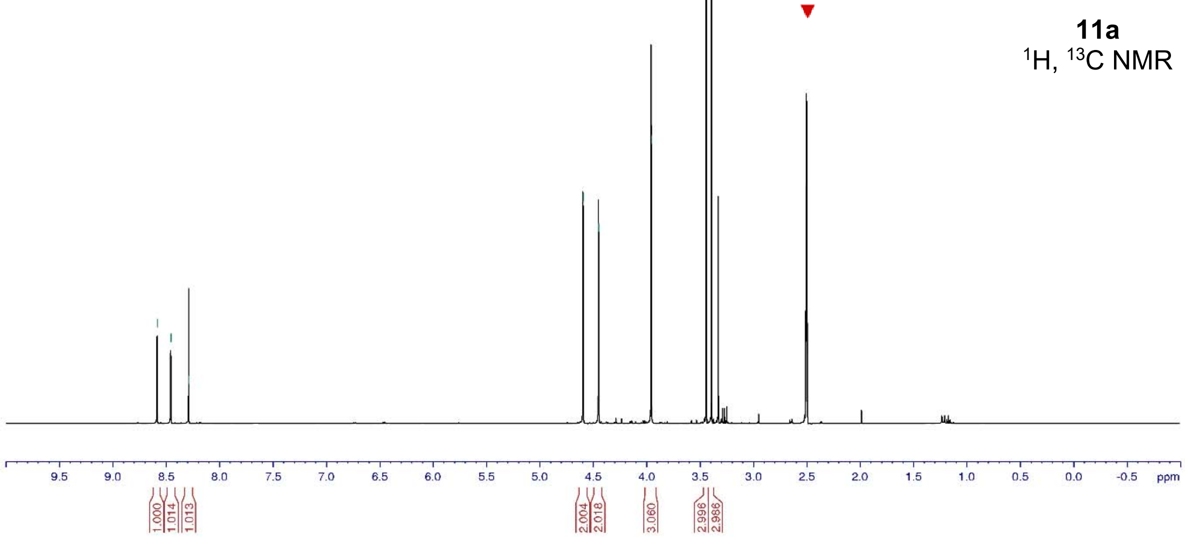

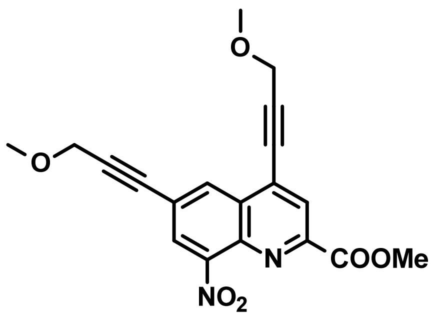

11a

^1H, ^{13}C NMR

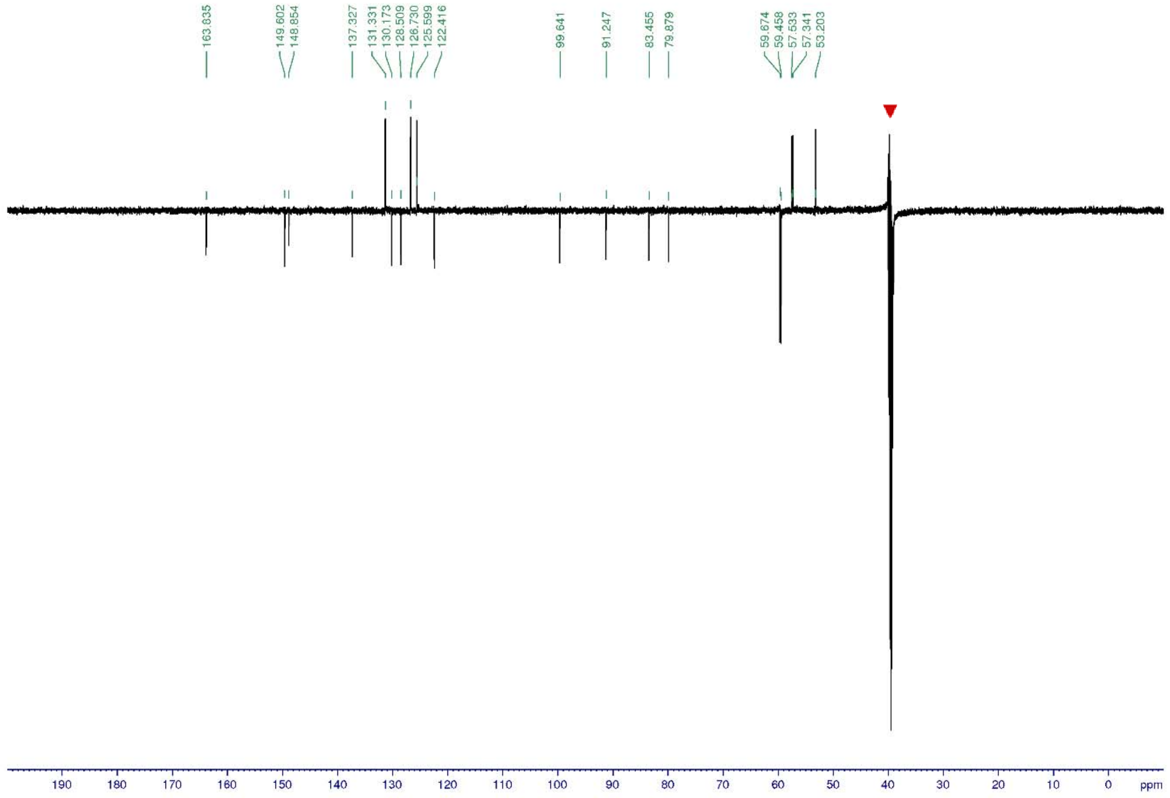

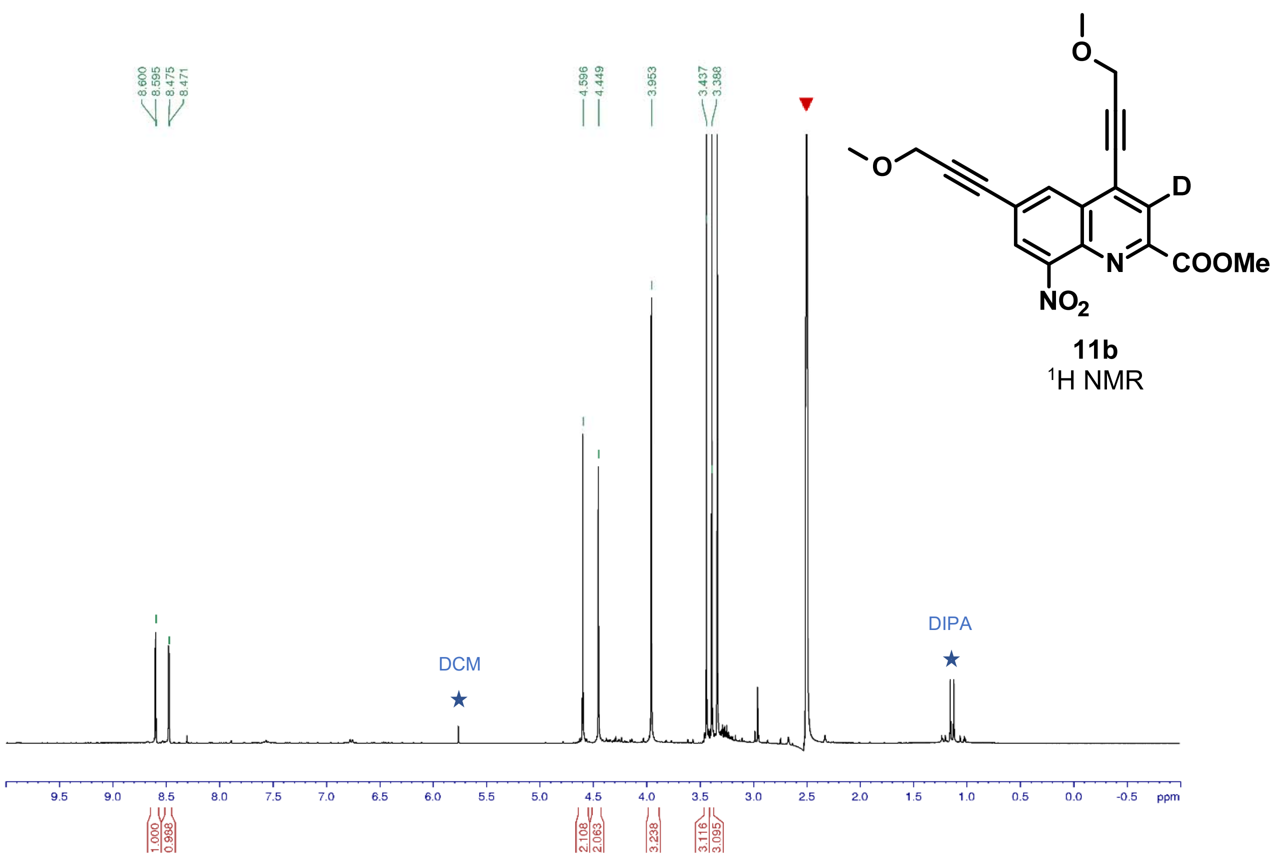

11b
^{1}H NMR

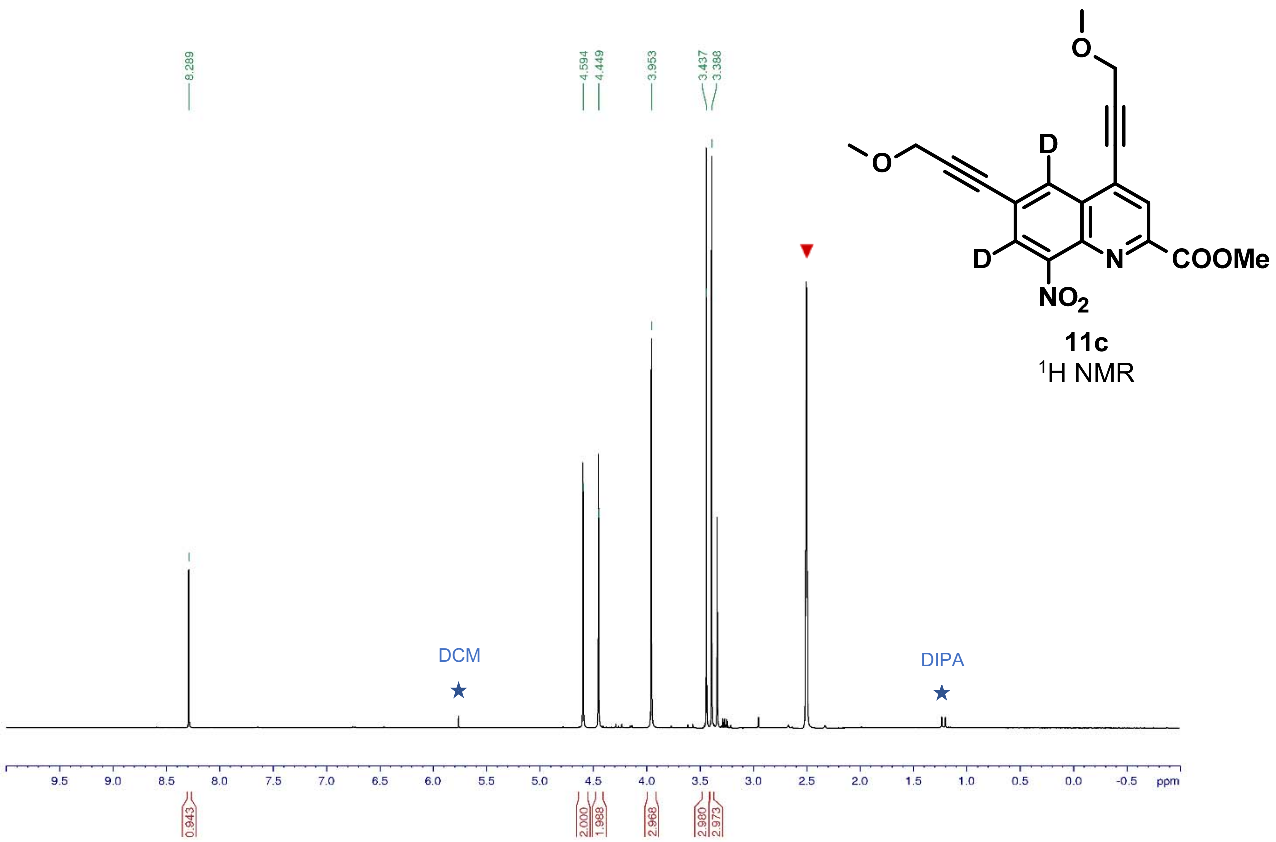

11c
^{1}H NMR

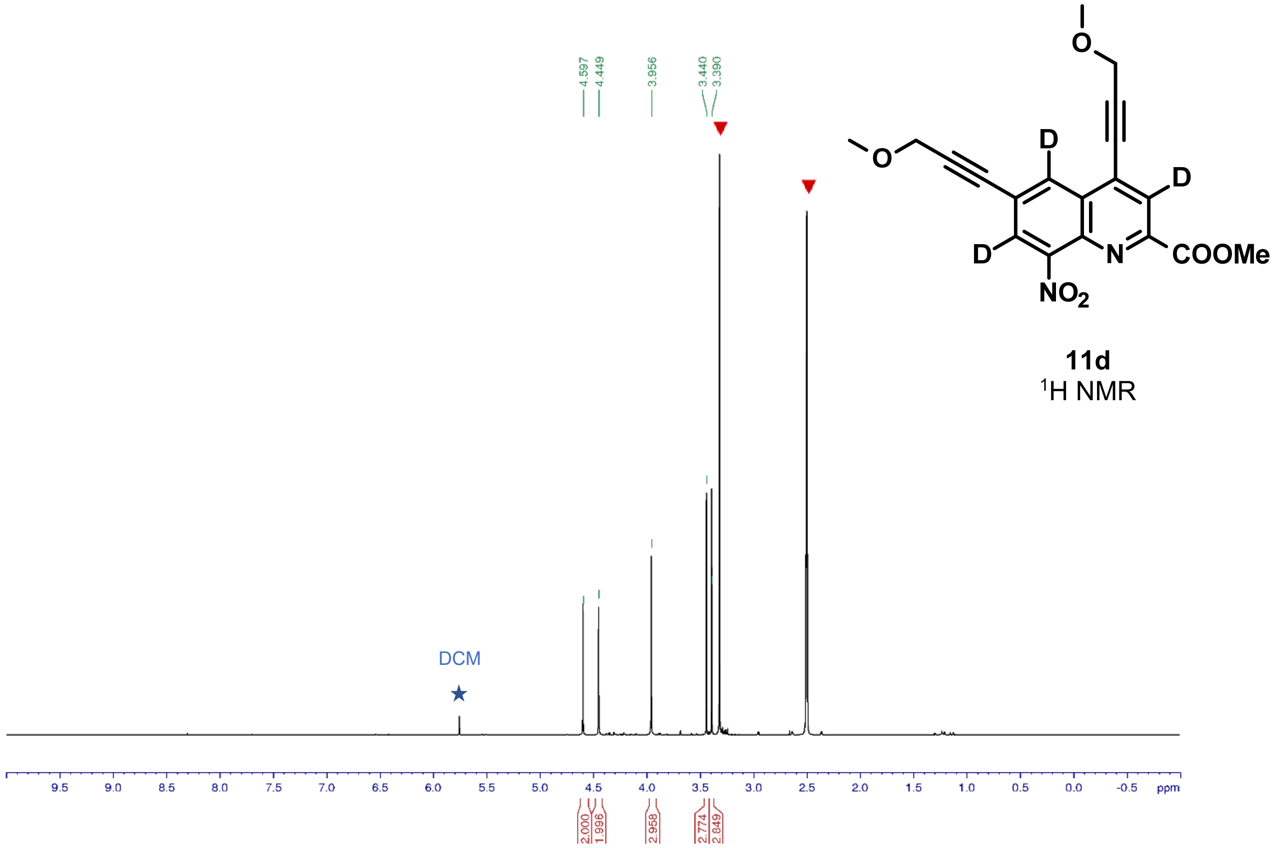

11d
^{1}H NMR

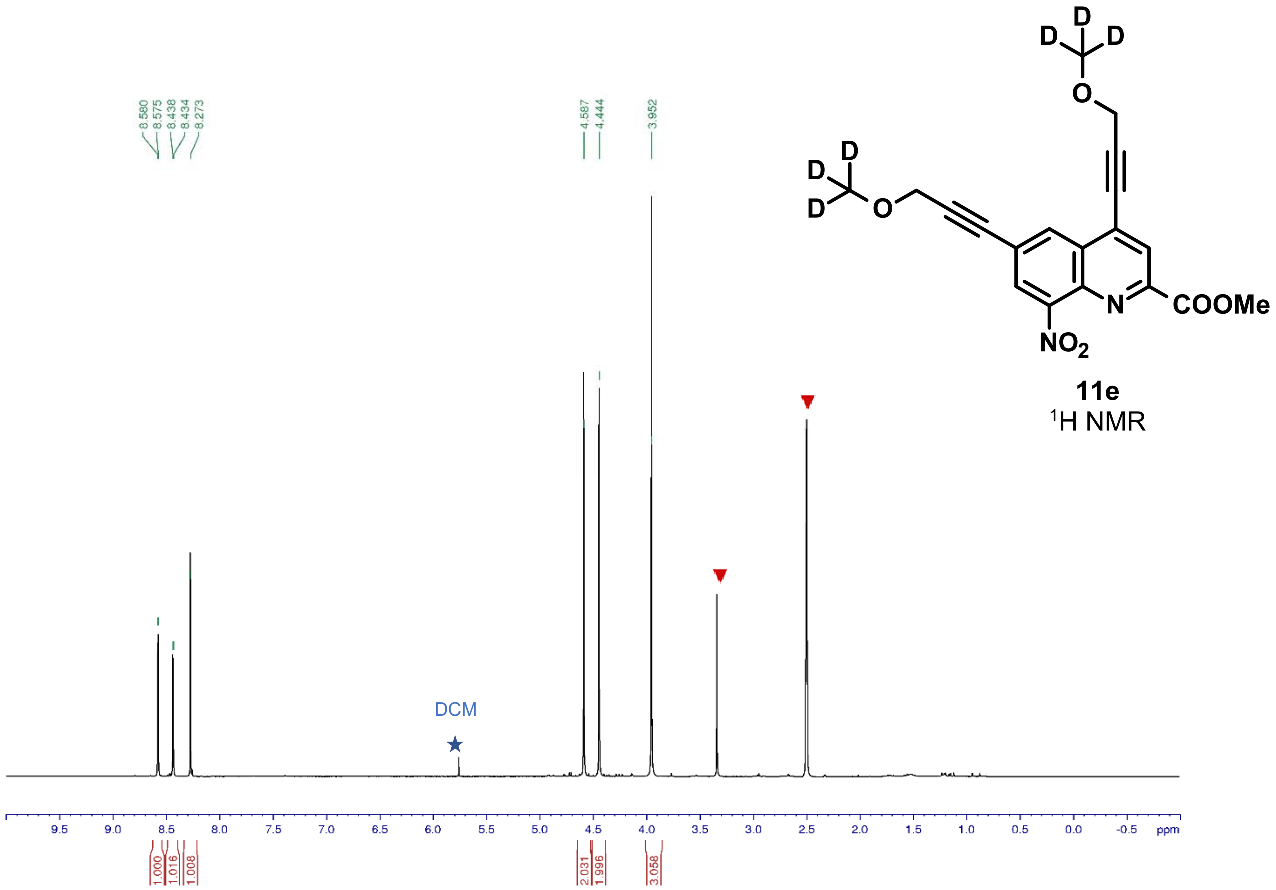

11e
^{1}H NMR

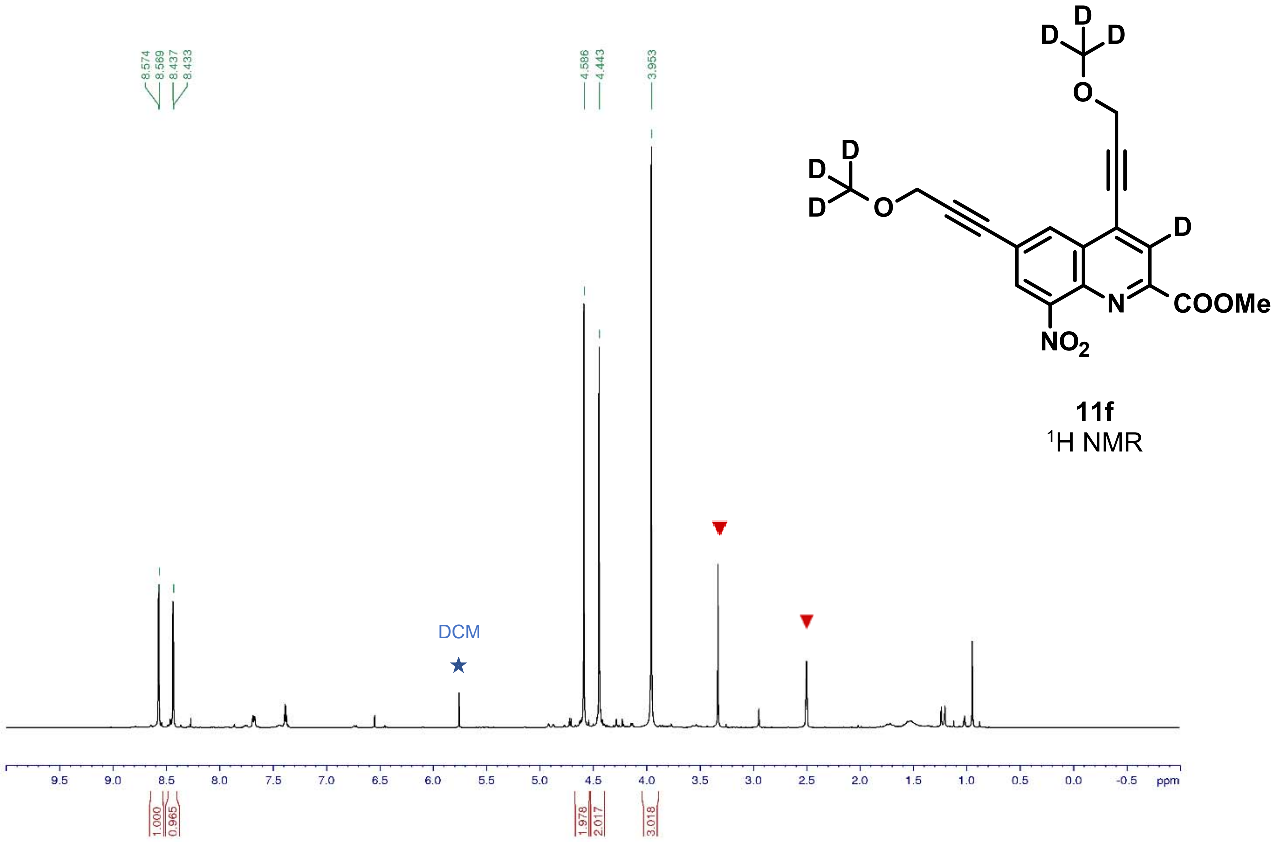

8.574
8.569
8.437
8.433
4.586
4.443
3.953
DCM
11f
1H NMR
COOMe
NO2
ppm
1.000
0.965
1.978
2.017
3.018

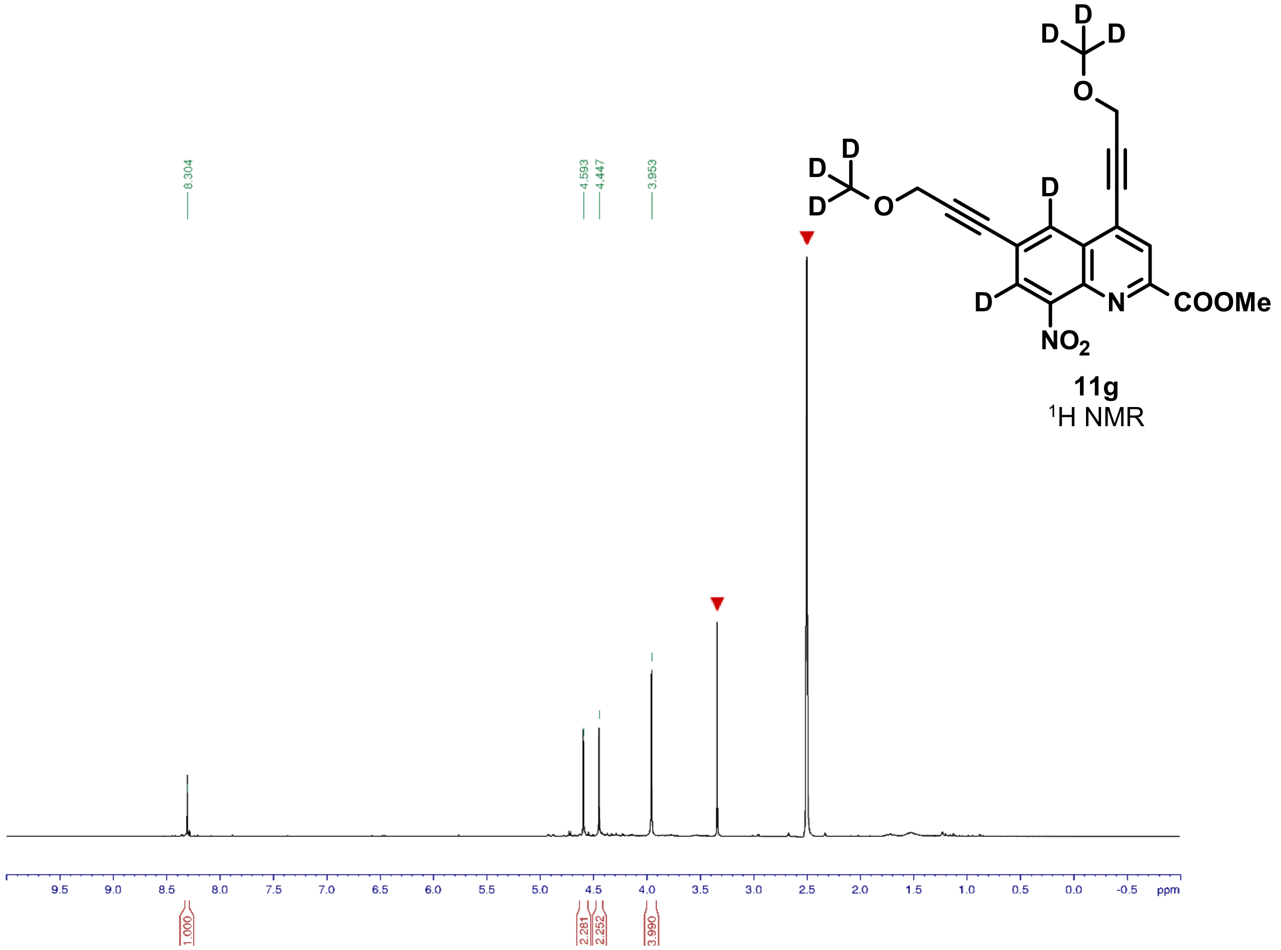

8.304
4.593
4.447
3.953
11g
1H NMR
COOMe
NO2
ppm
1.000
2.281
2.252
3.990

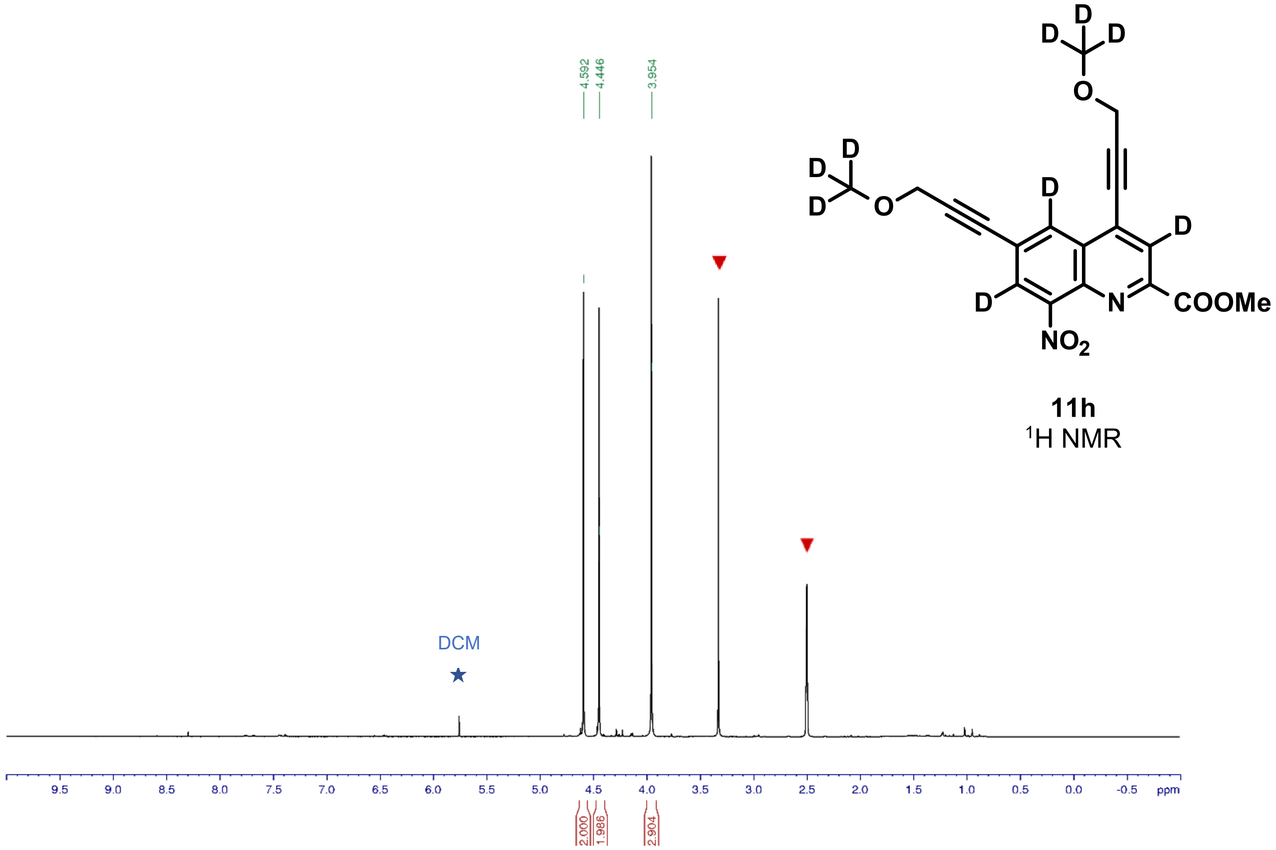

11h
^{1}H NMR

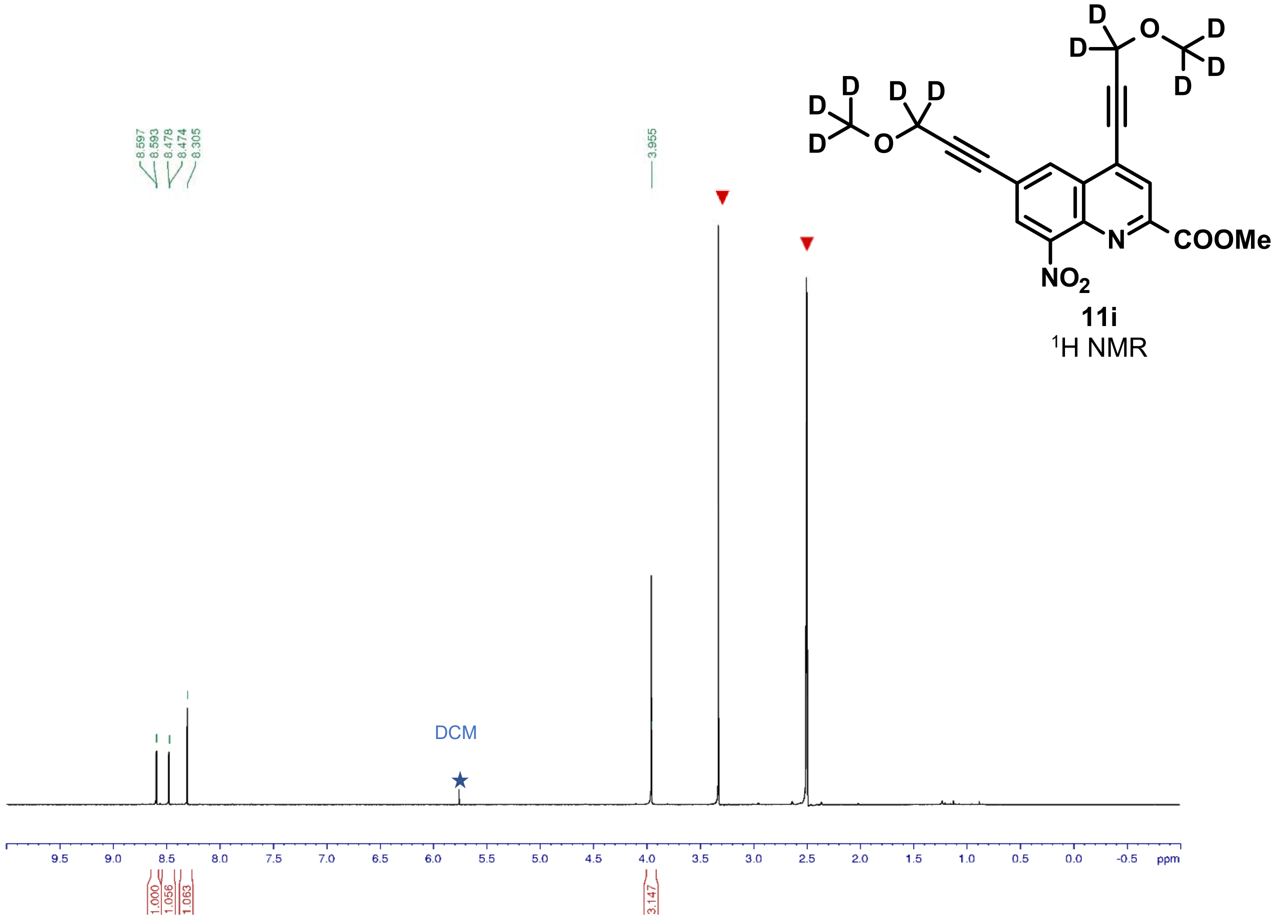

11i
^{1}H NMR

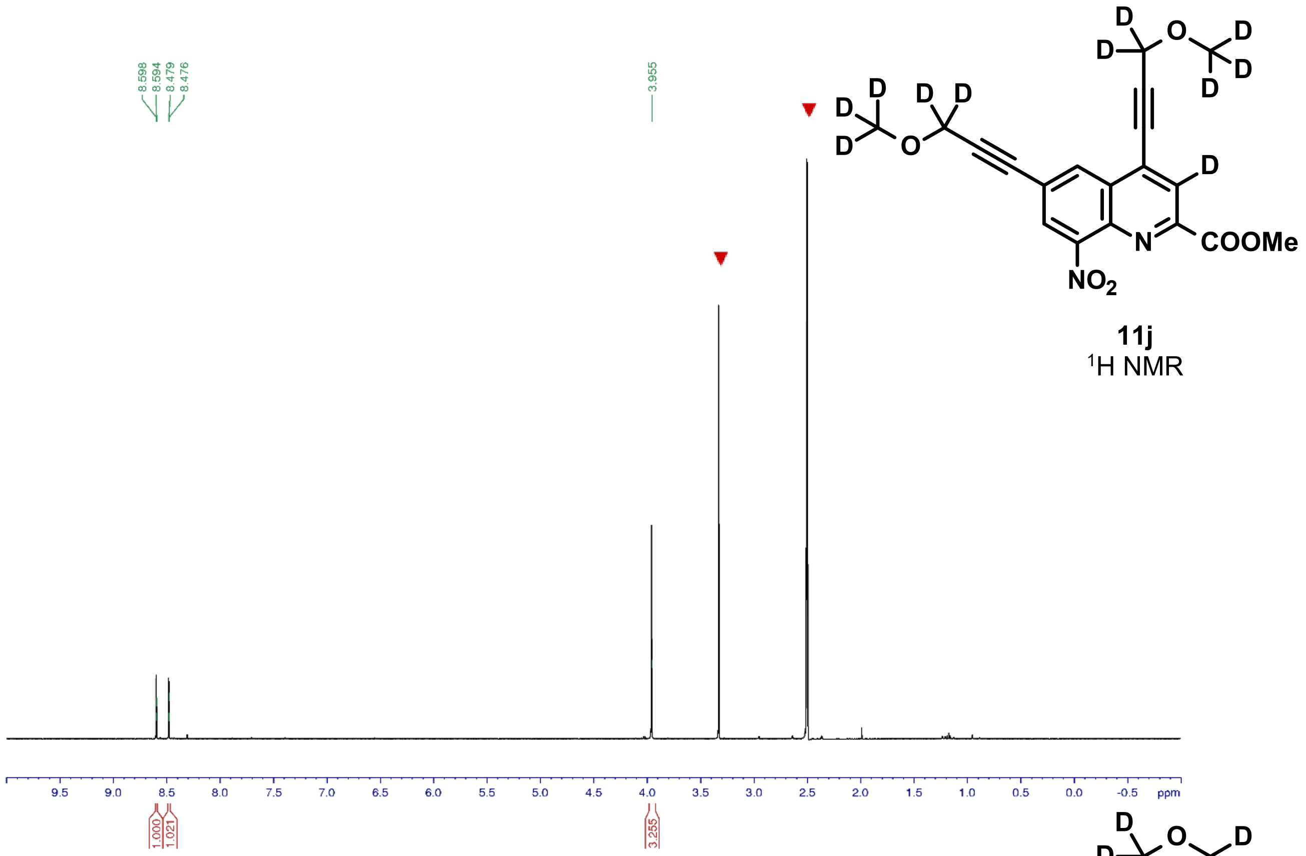

11j
^{1}H NMR

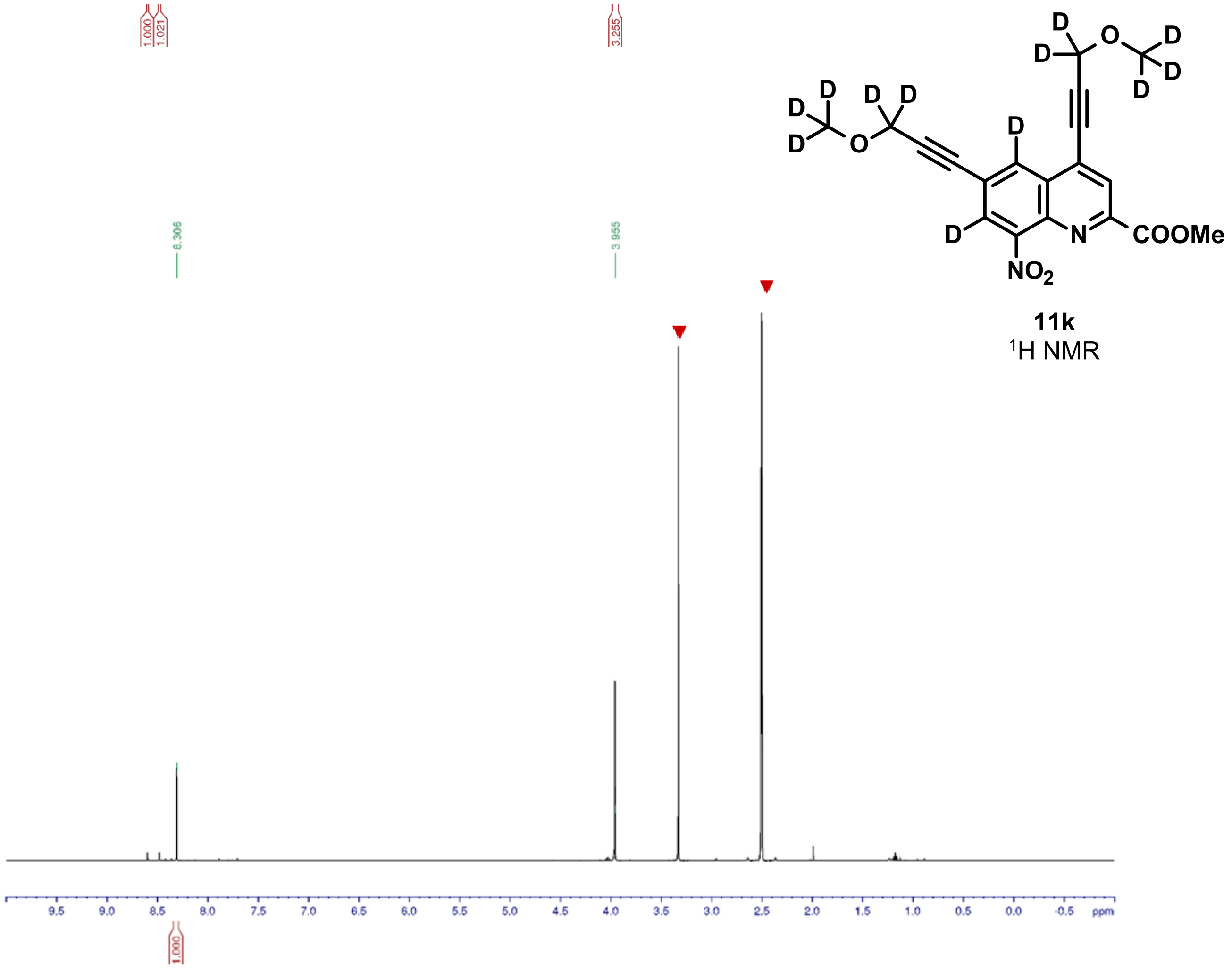

11k
^{1}H NMR

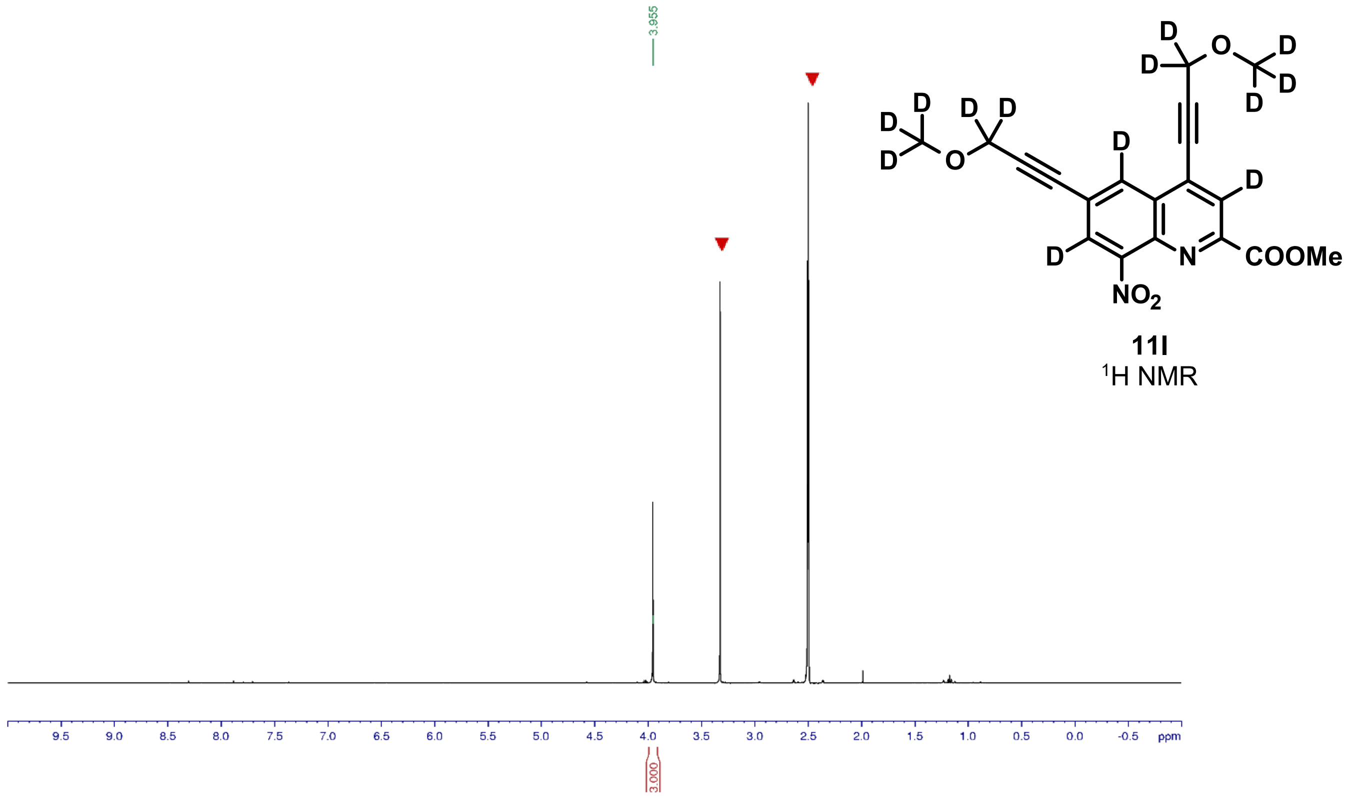
3.955
9.5
9.0
8.5
8.0
7.5
7.0
6.5
6.0
5.5
5.0
4.5
4.0
3.5
3.0
2.5
2.0
1.5
1.0
0.5
0.0
-0.5
ppm
3.000
D
O
NO2
N
COOMe

11l
^{1}H NMR

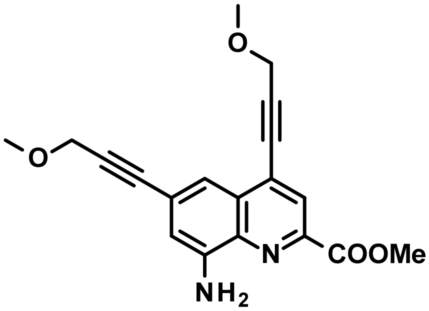

O
O
N
COOMe
NH2

21
^{1}H, ^{13}C NMR

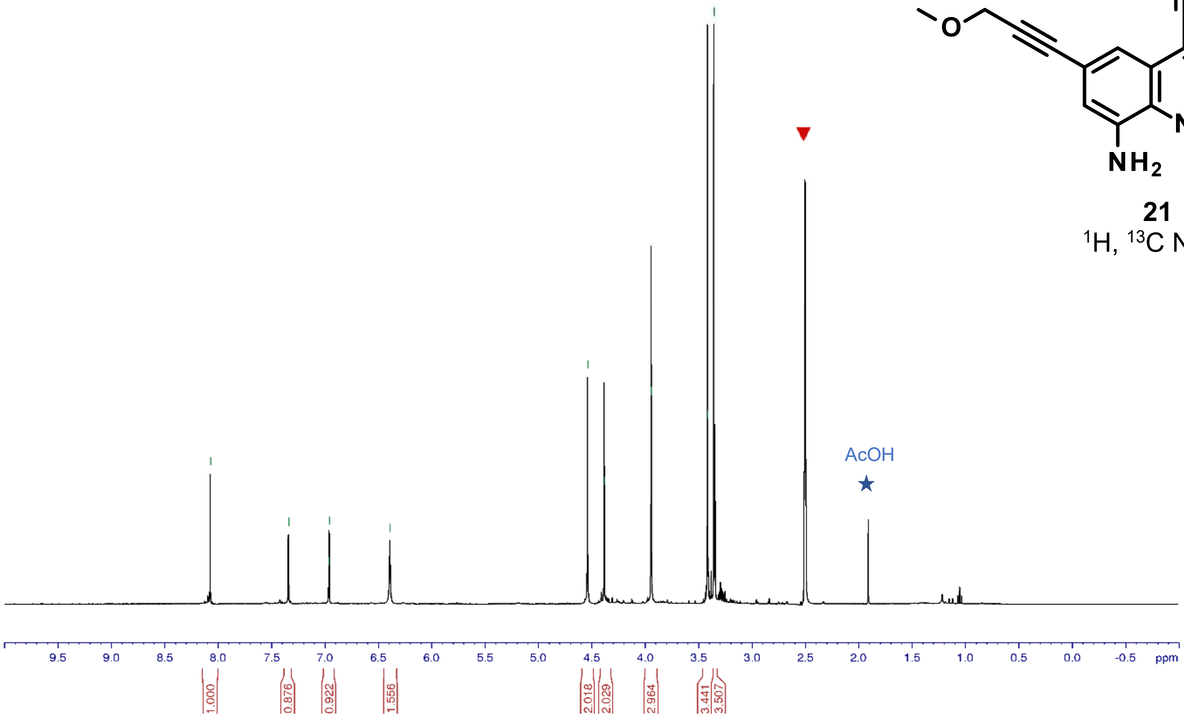

8.072
7.342
7.337
6.961
6.957
6.389
4.539
4.381
3.941
3.414
3.355
AcOH
9.5 9.0 8.5 8.0 7.5 7.0 6.5 6.0 5.5 5.0 4.5 4.0 3.5 3.0 2.5 2.0 1.5 1.0 0.5 0.0 -0.5 ppm
1.000
0.876
0.922
1.556
2.018
2.029
2.964
3.441
3.507

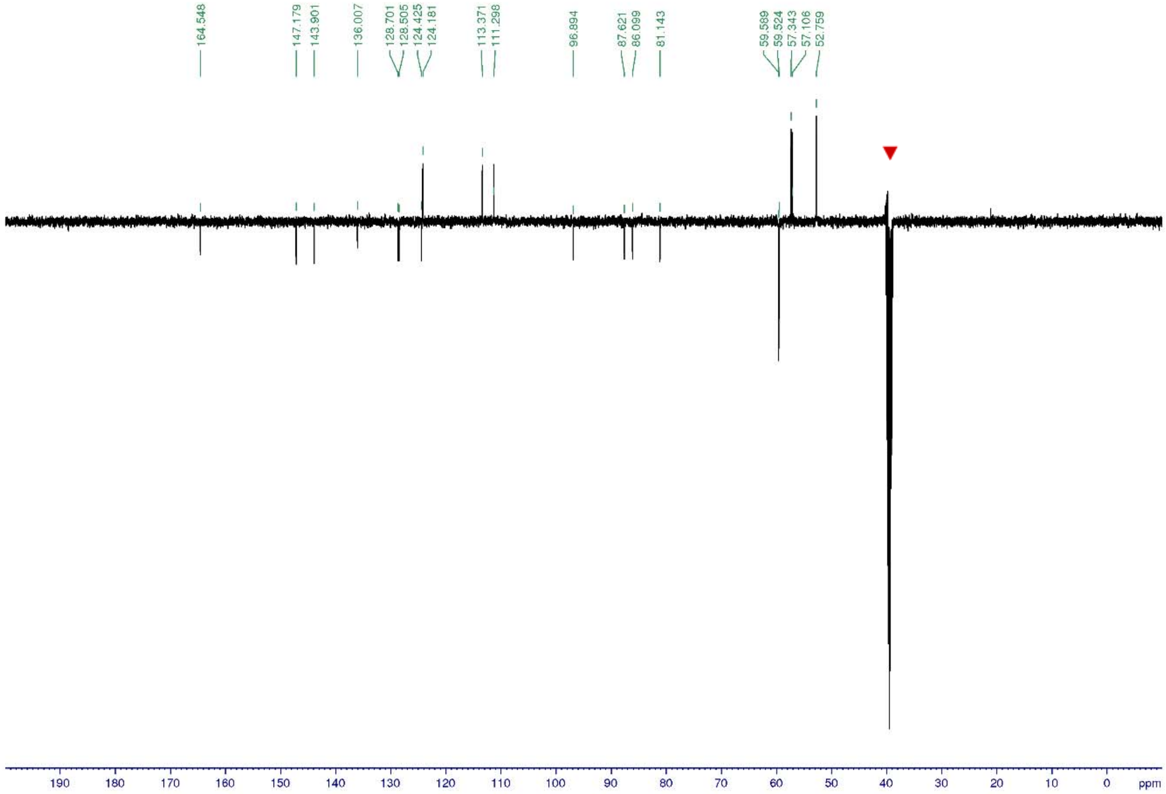

164.548
147.179
143.901
136.007
128.701
128.505
124.425
124.181
113.371
111.298
96.894
87.621
86.099
81.143
59.589
59.524
57.343
57.106
52.759
190 180 170 160 150 140 130 120 110 100 90 80 70 60 50 40 30 20 10 0 ppm

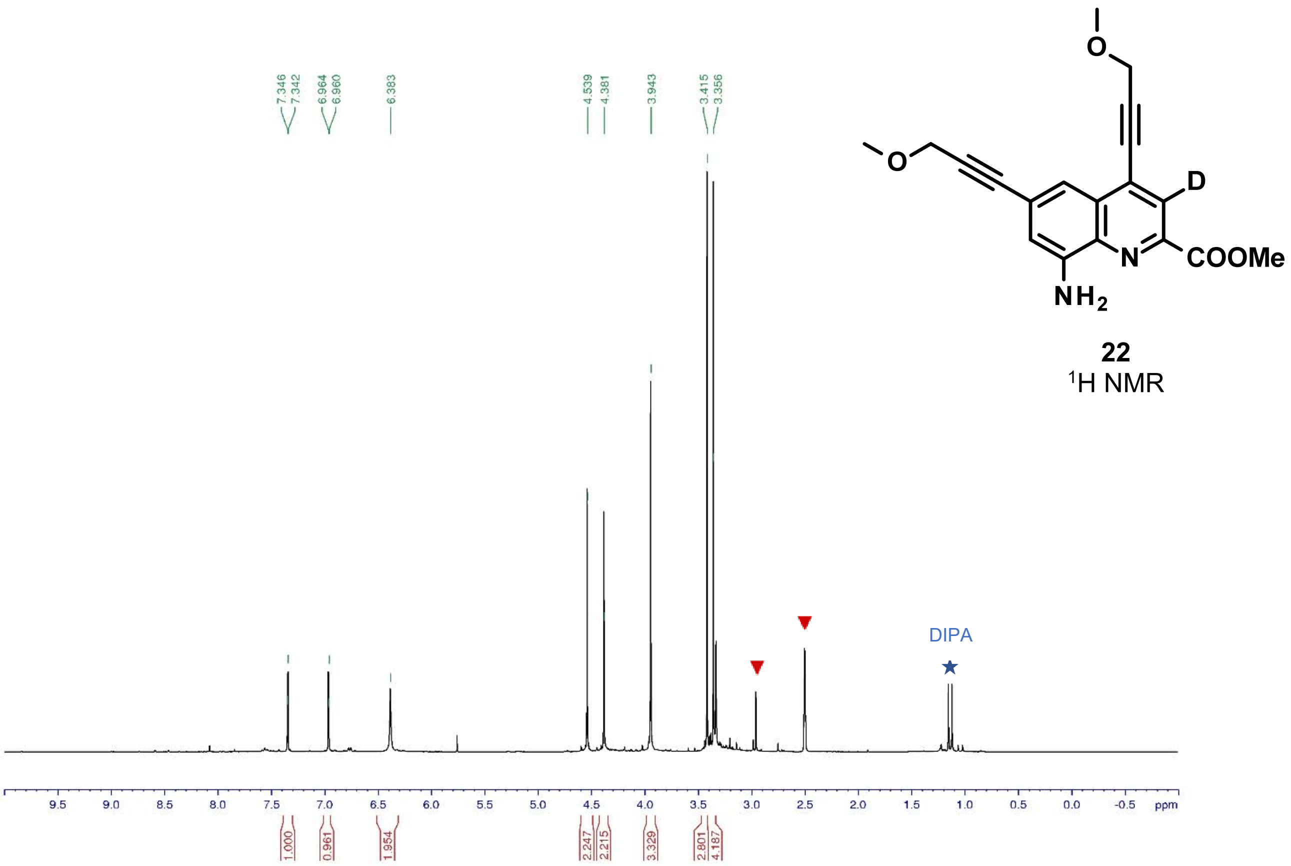

22
^{1}H NMR

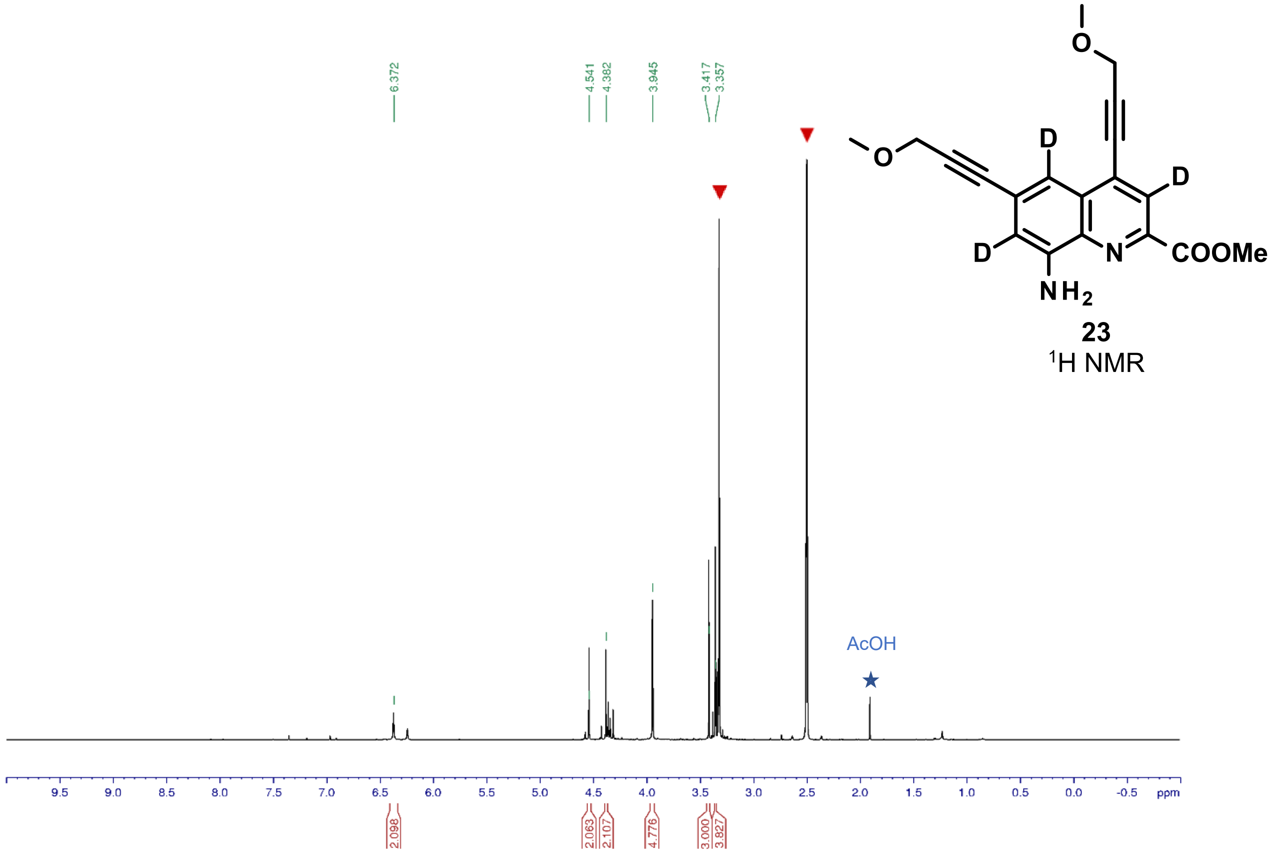

23
^{1}H NMR

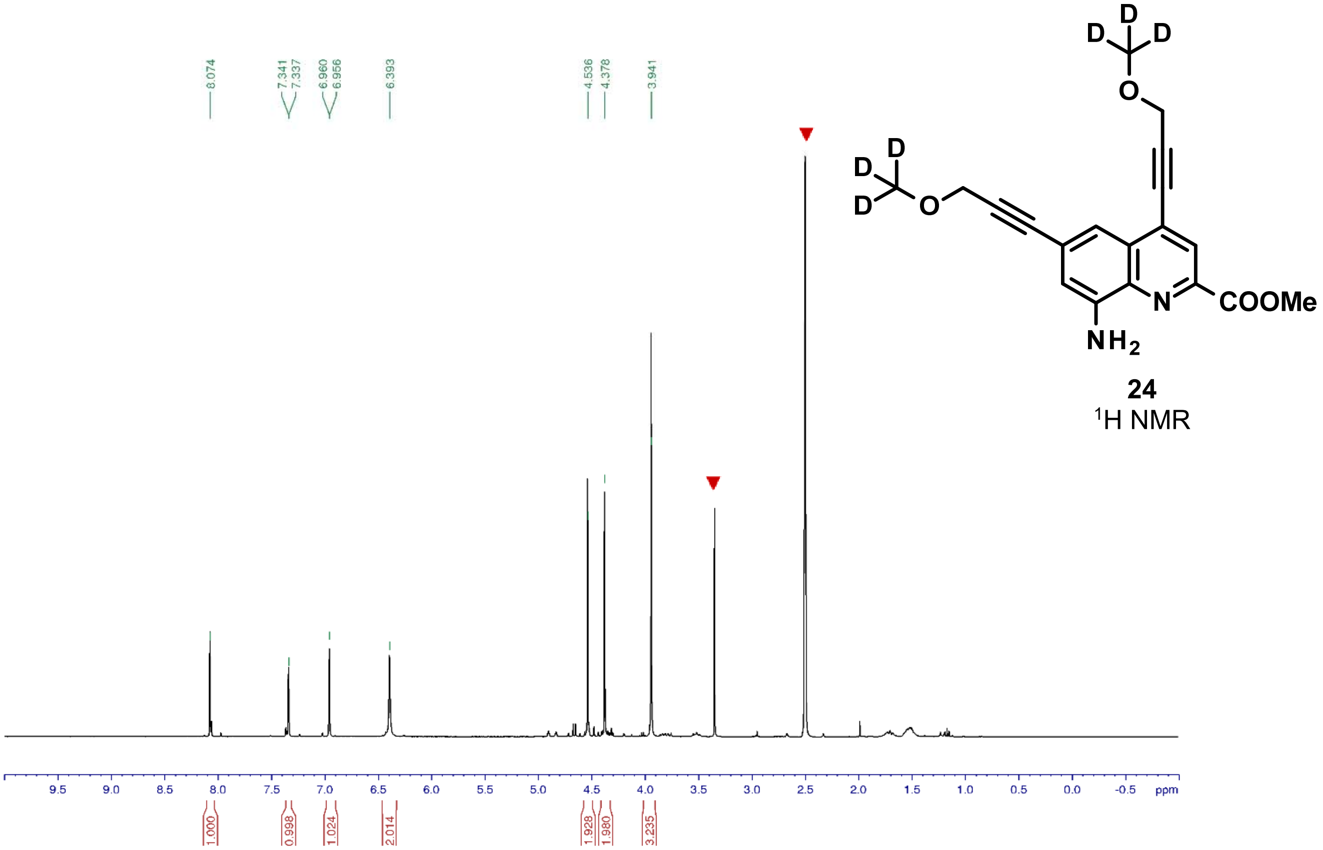

24
^{1}H NMR

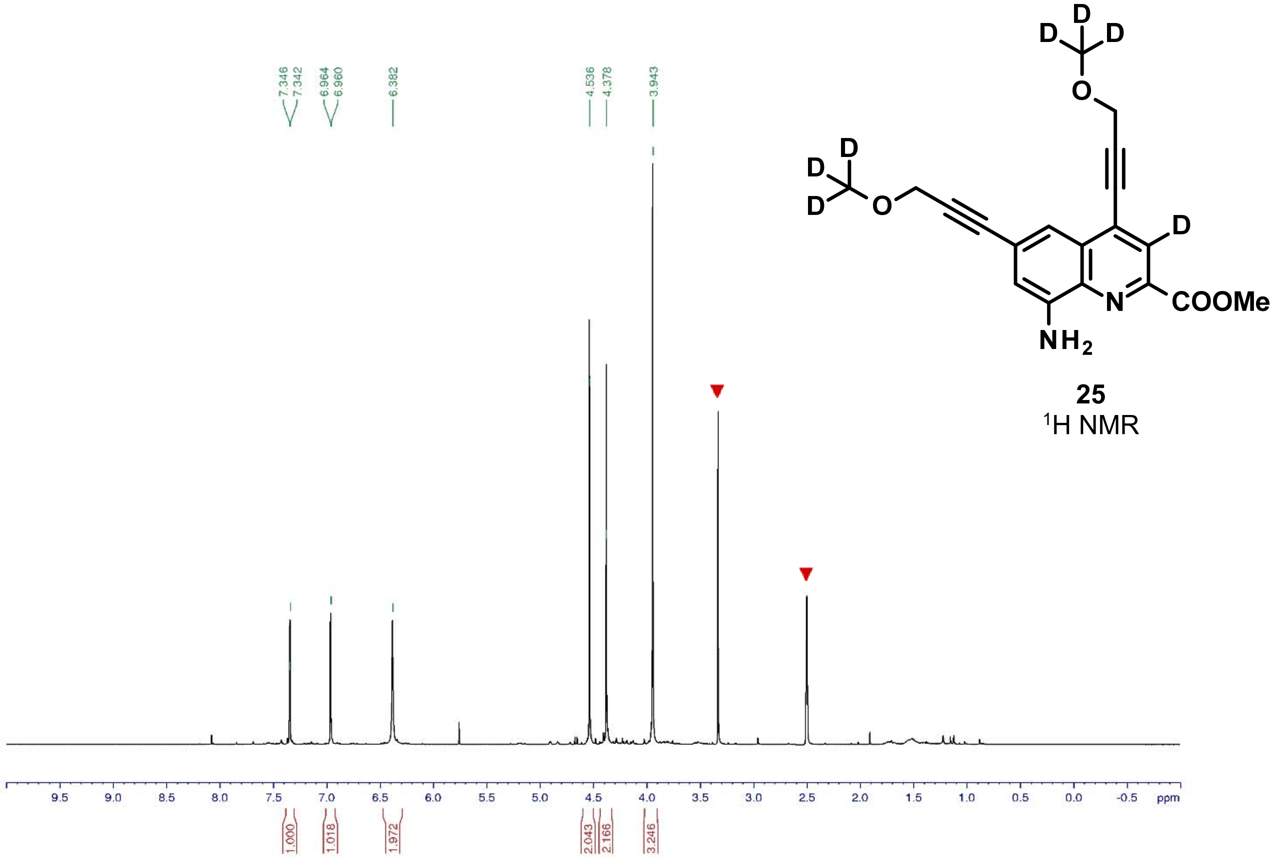

25
^{1}H NMR

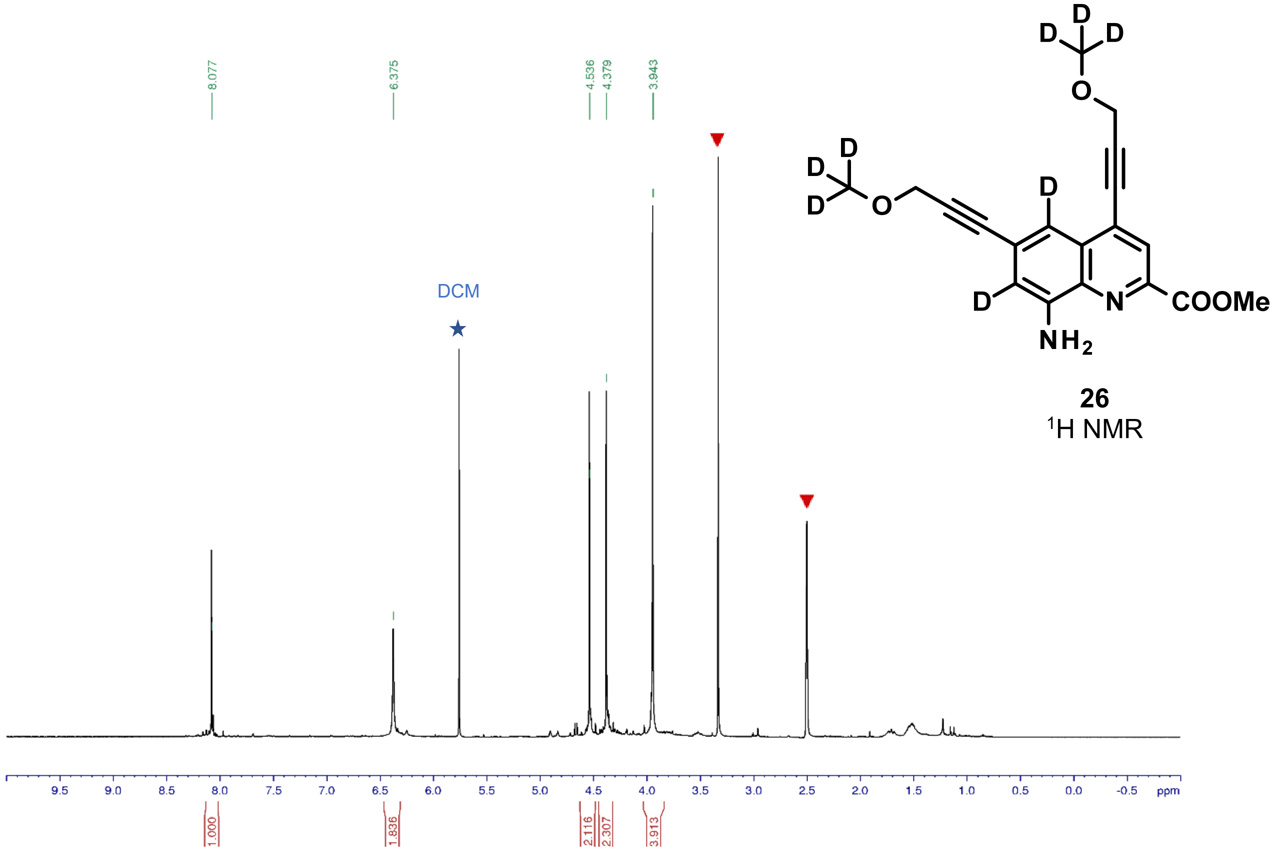
8.077
6.375
4.536
4.379
3.943
DCM
COOMe
NH2
ppm
1.000
1.836
2.116
2.307
3.913

26
^{1}H NMR

12a
^{1}H, ^{13}C NMR

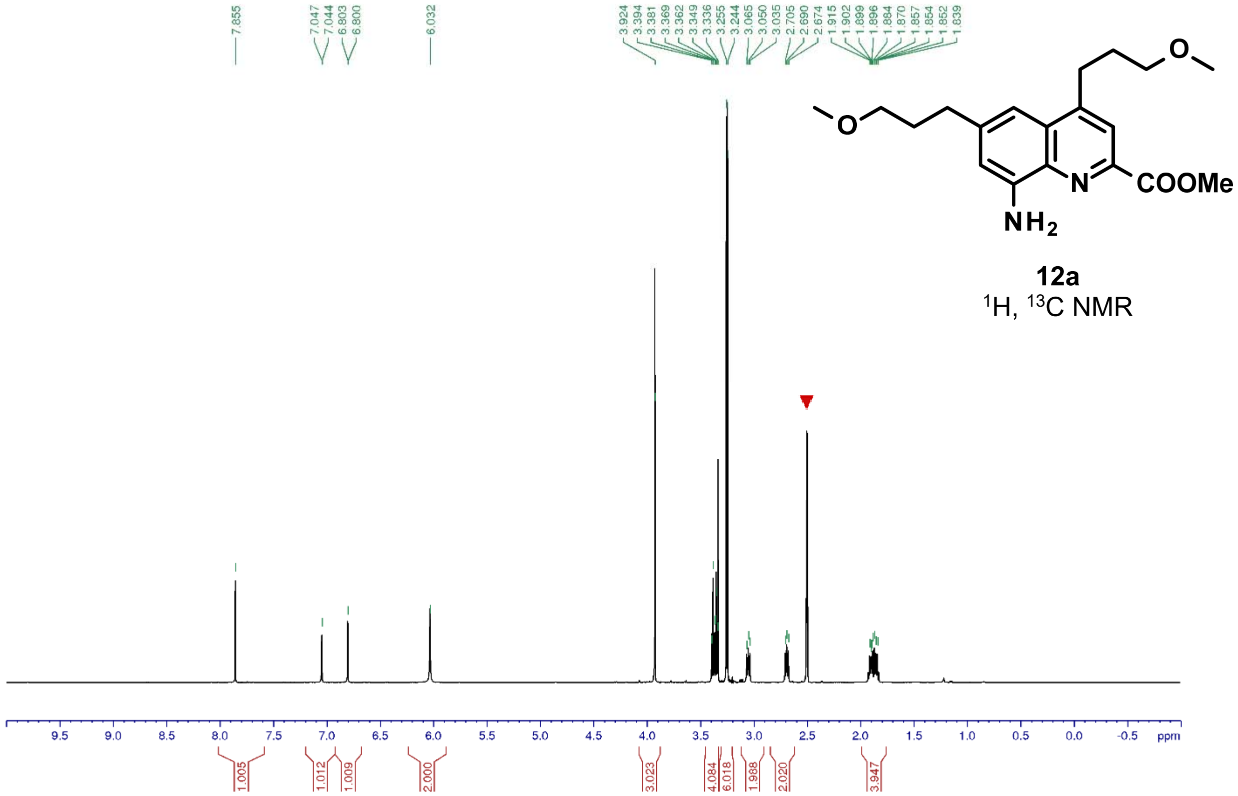

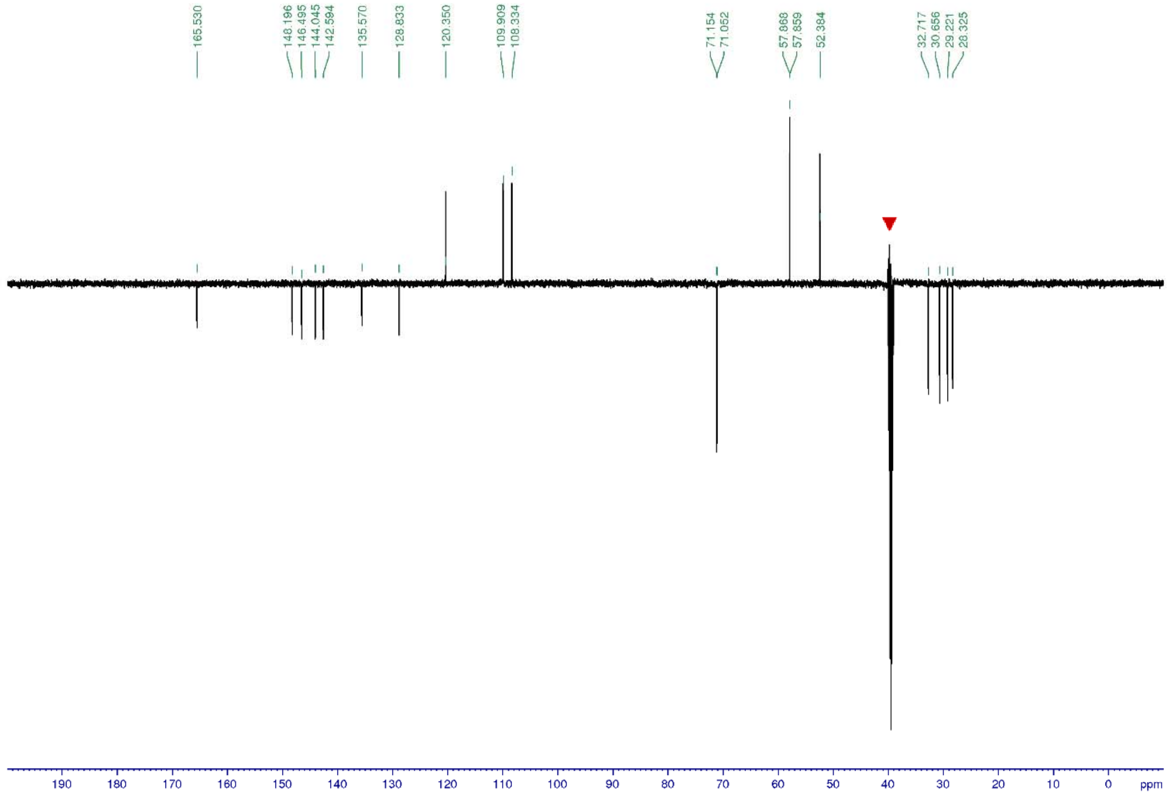

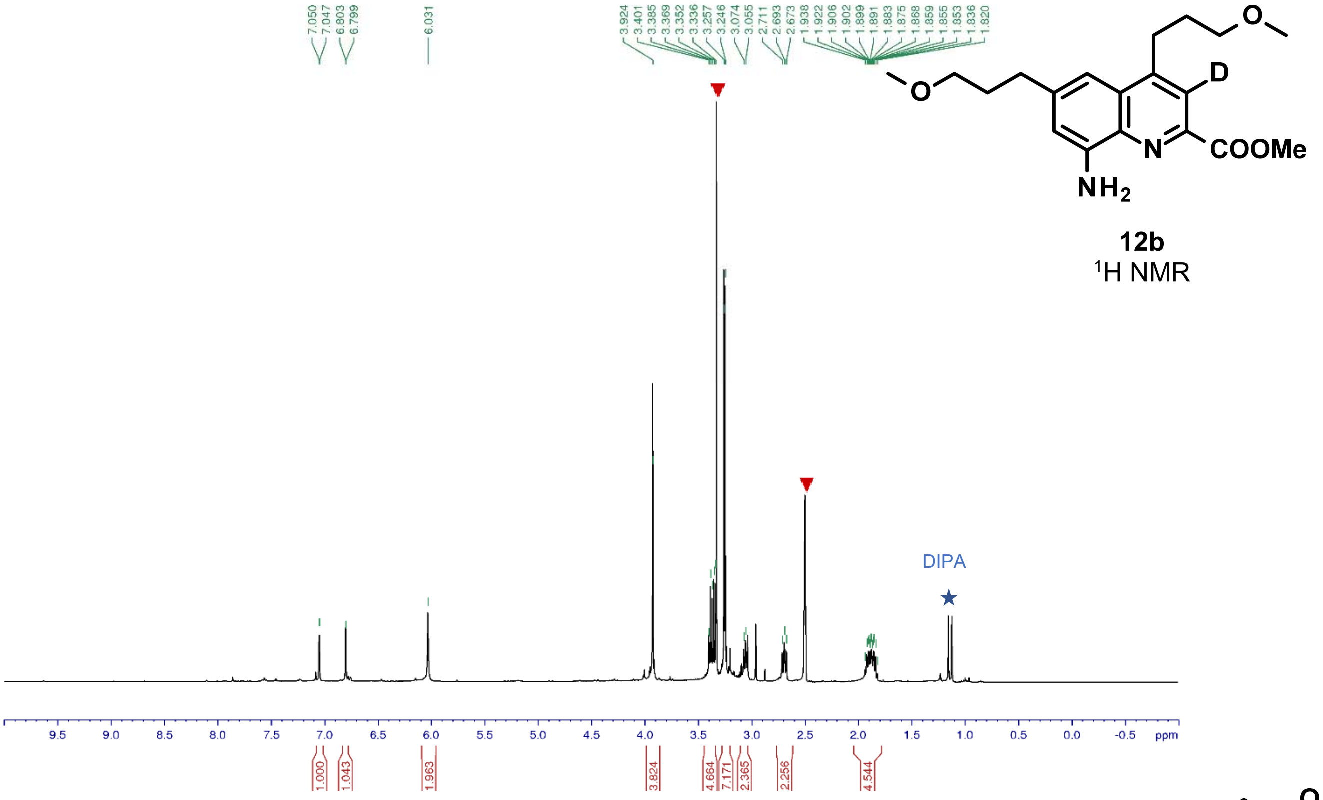

12b
^{1}H NMR

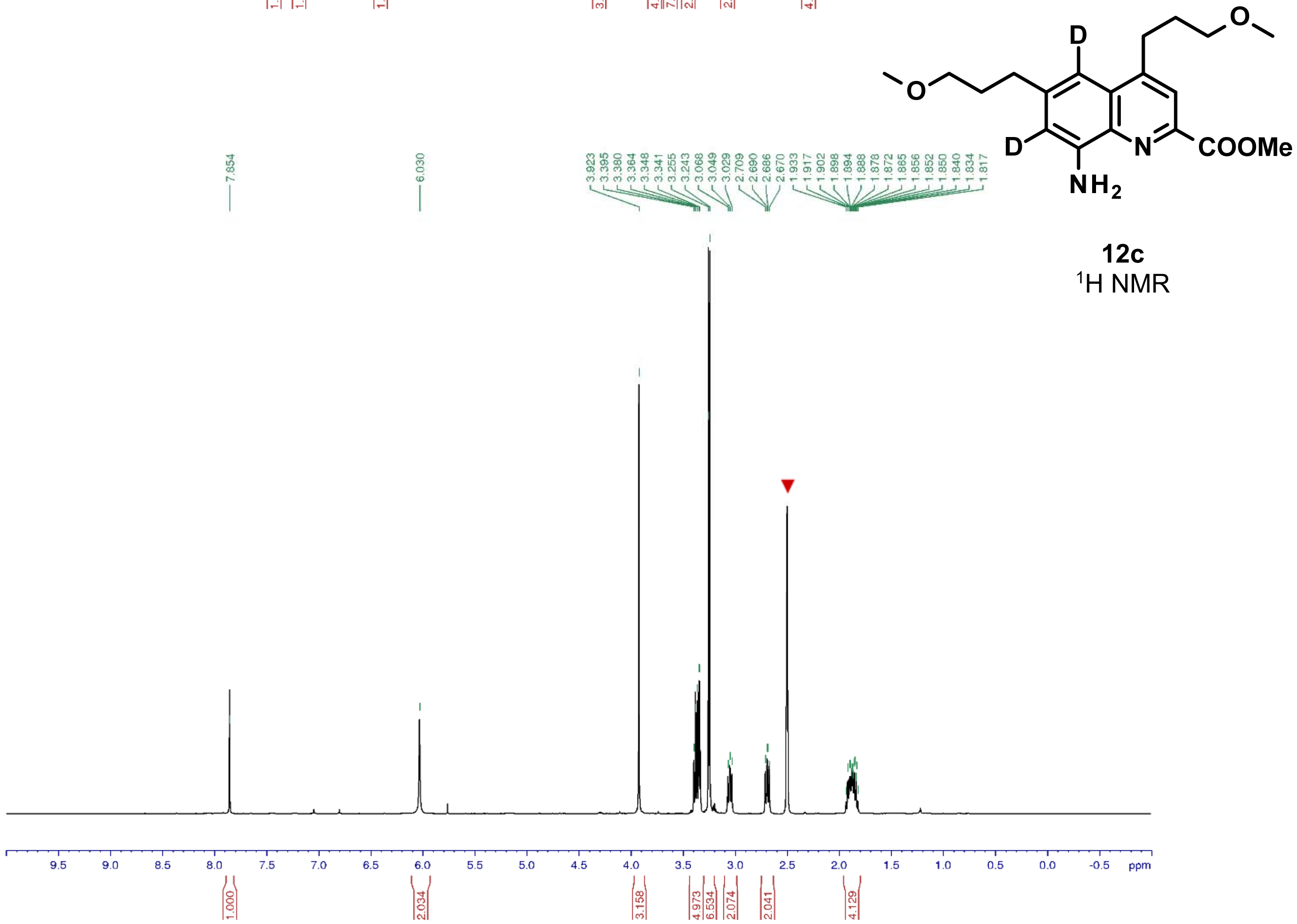

12c
^{1}H NMR

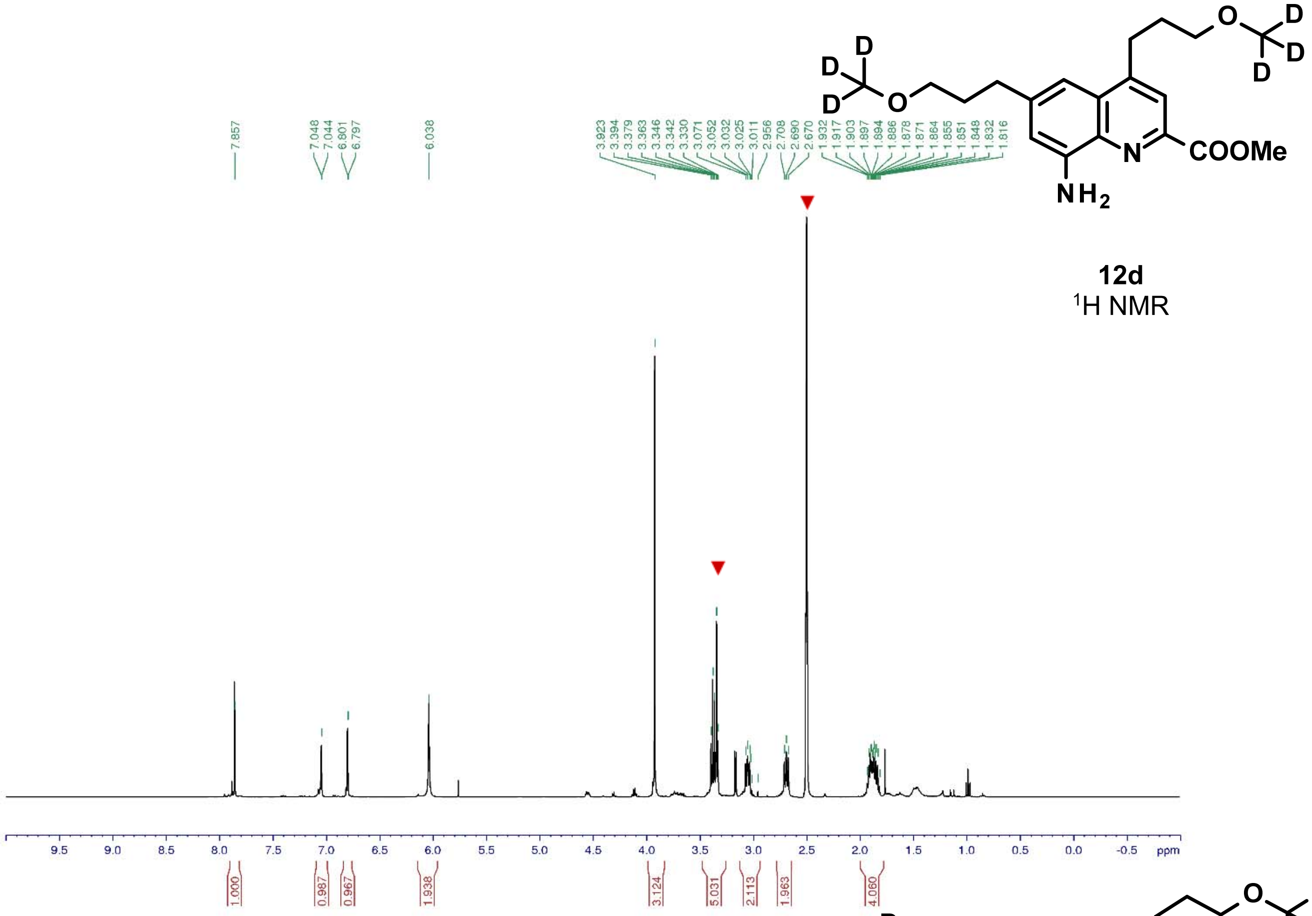

12d
^{1}H NMR

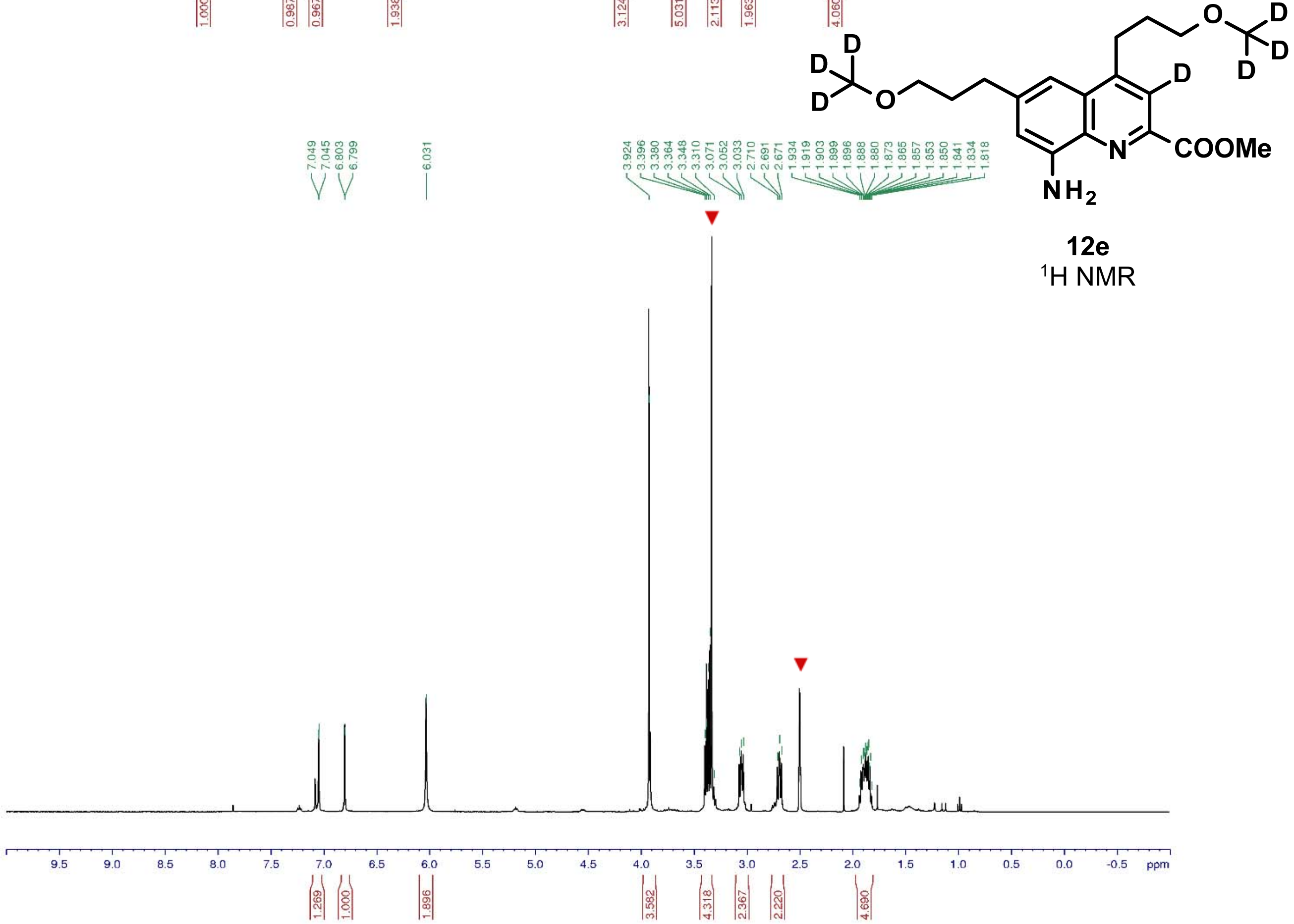

12e
^{1}H NMR

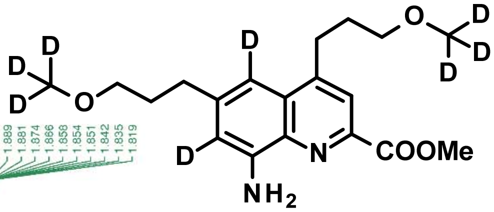

12f
^{1}H NMR

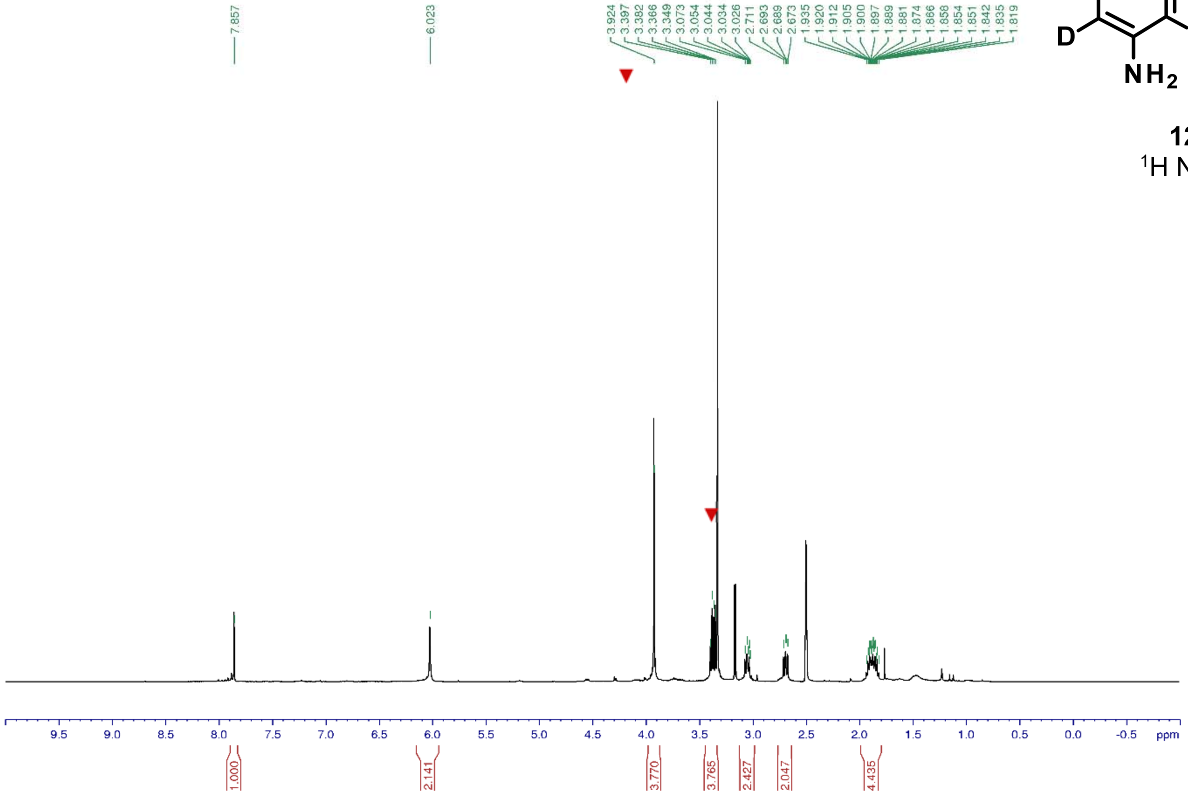

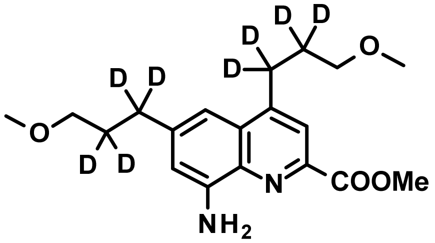

27
^{1}H NMR

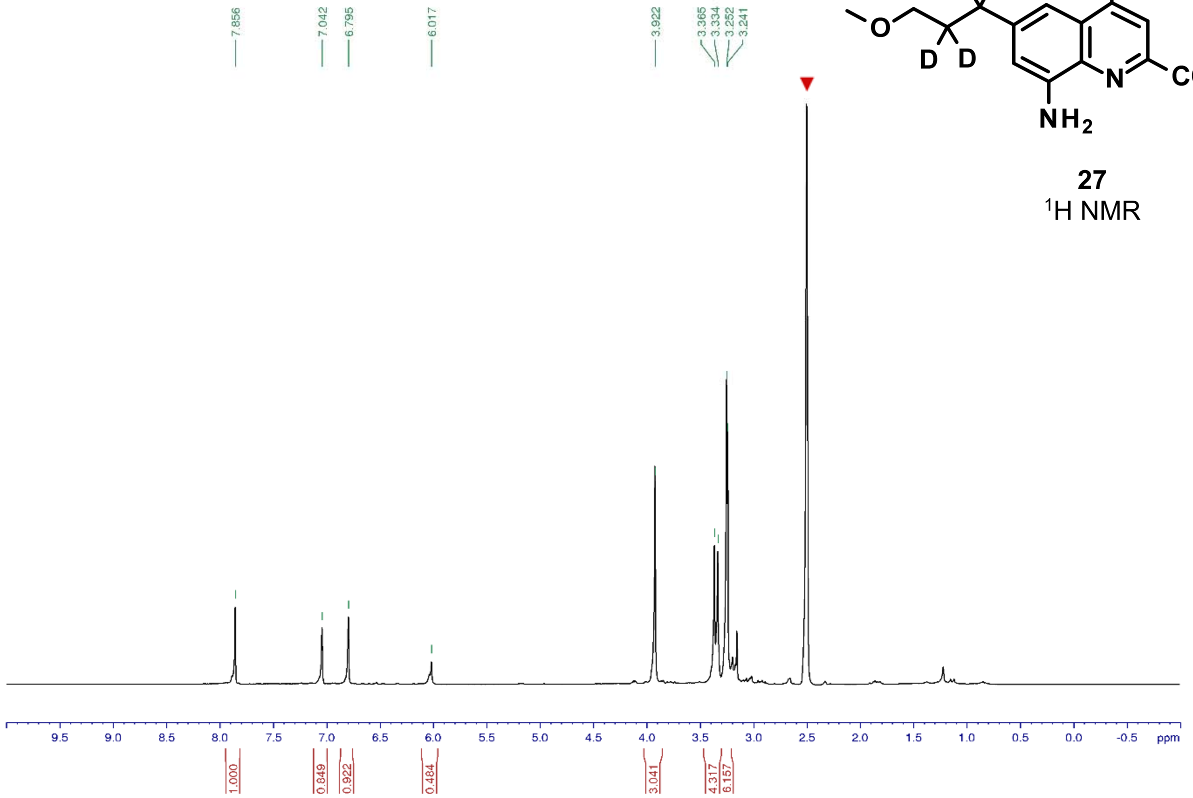

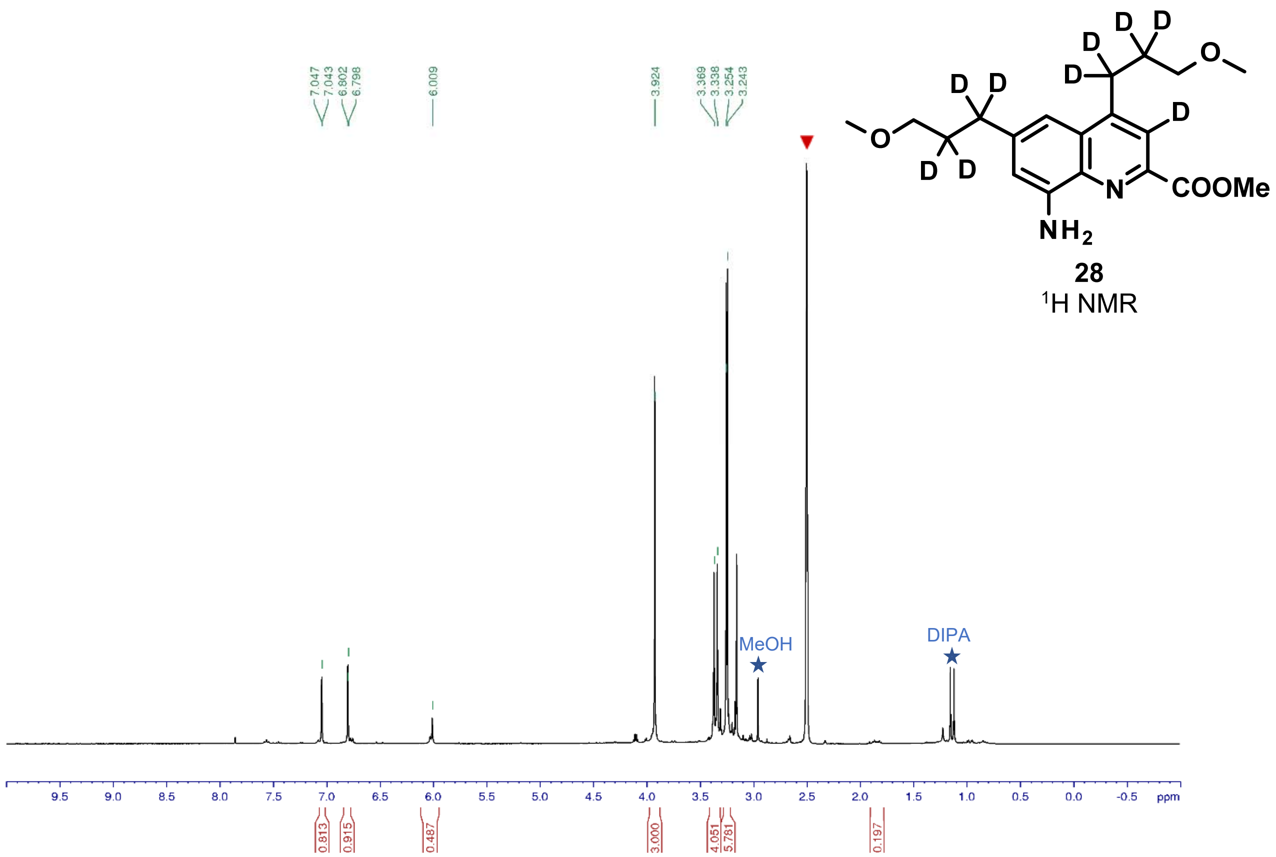
7.047
7.043
6.802
6.798
6.009
3.924
3.369
3.338
3.254
3.243
MeOH
DIPA
ppm
0.813
0.915
0.487
3.000
4.051
5.781
0.197
COOMe
28
^{1}H NMR

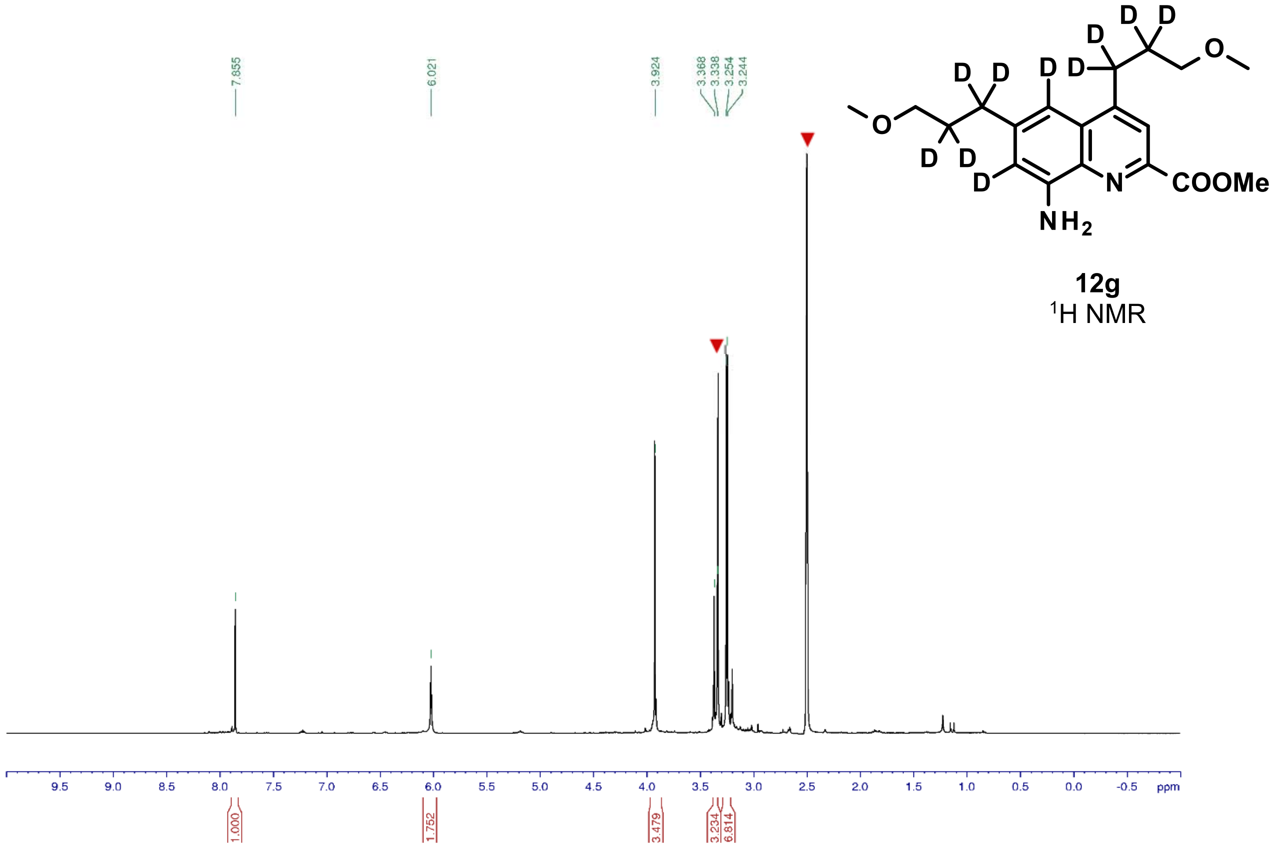
7.855
6.021
3.924
3.368
3.338
3.254
3.244
ppm
1.000
1.752
3.479
3.234
6.814
COOMe
12g
^{1}H NMR

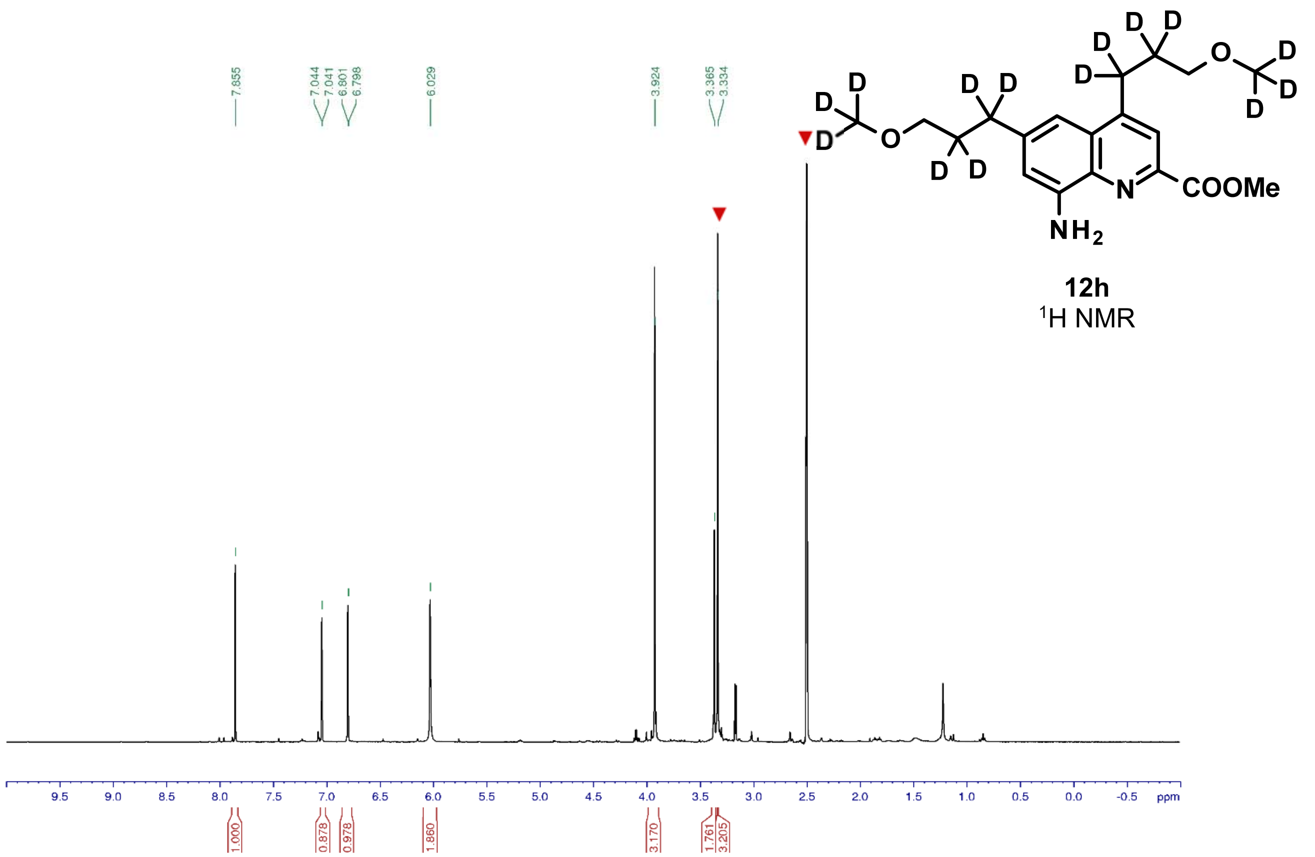
12h
^{1}H NMR
7.855
7.044
7.041
6.801
6.798
6.029
3.924
3.365
3.334
COOMe
NH_2
9.5 9.0 8.5 8.0 7.5 7.0 6.5 6.0 5.5 5.0 4.5 4.0 3.5 3.0 2.5 2.0 1.5 1.0 0.5 0.0 -0.5 ppm
1.000
0.878
0.978
1.860
3.170
1.761
3.205

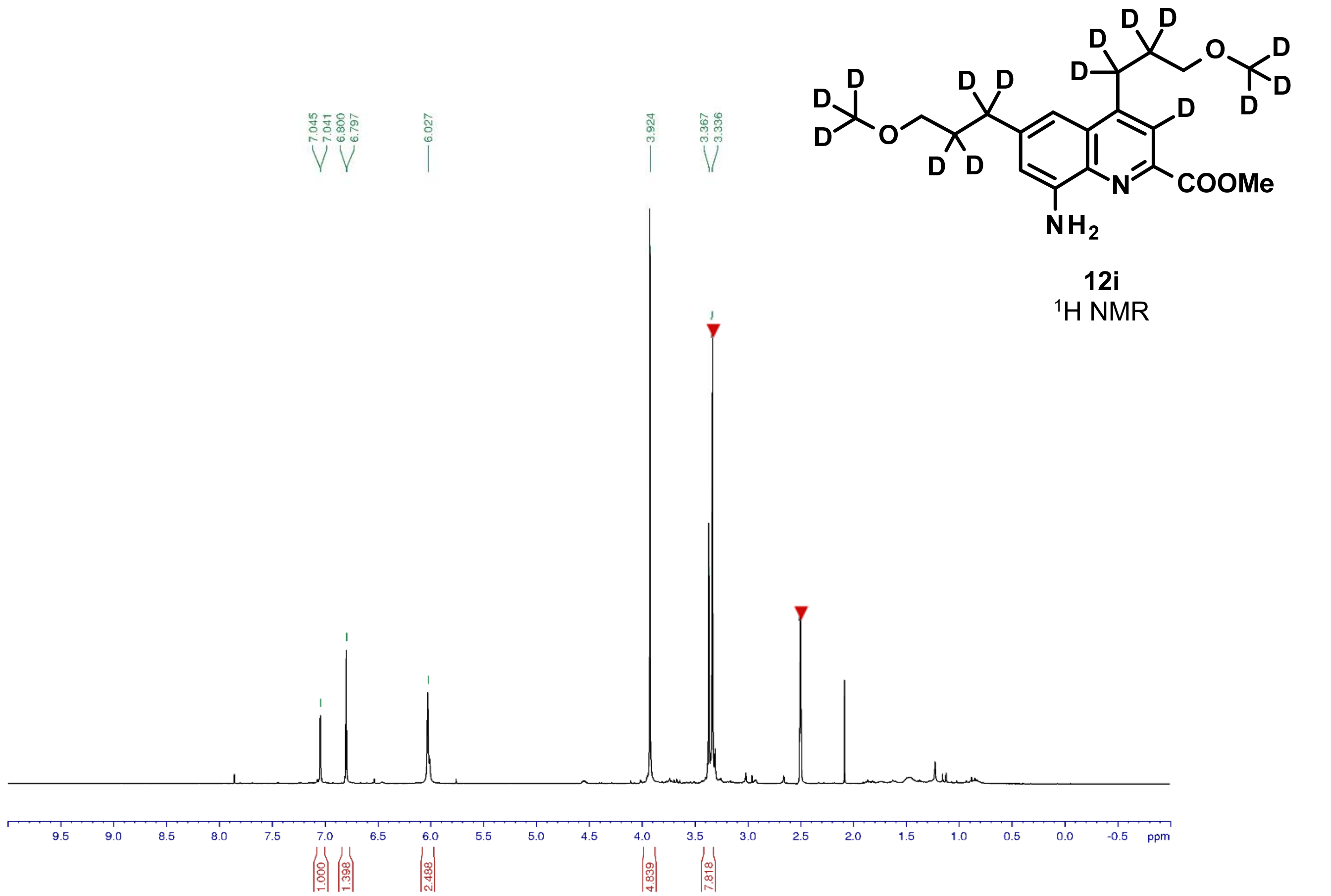
12i
^{1}H NMR
7.045
7.041
6.800
6.797
6.027
3.924
3.367
3.336
COOMe
NH_2
9.5 9.0 8.5 8.0 7.5 7.0 6.5 6.0 5.5 5.0 4.5 4.0 3.5 3.0 2.5 2.0 1.5 1.0 0.5 0.0 -0.5 ppm
1.000
1.398
2.488
4.839
7.818

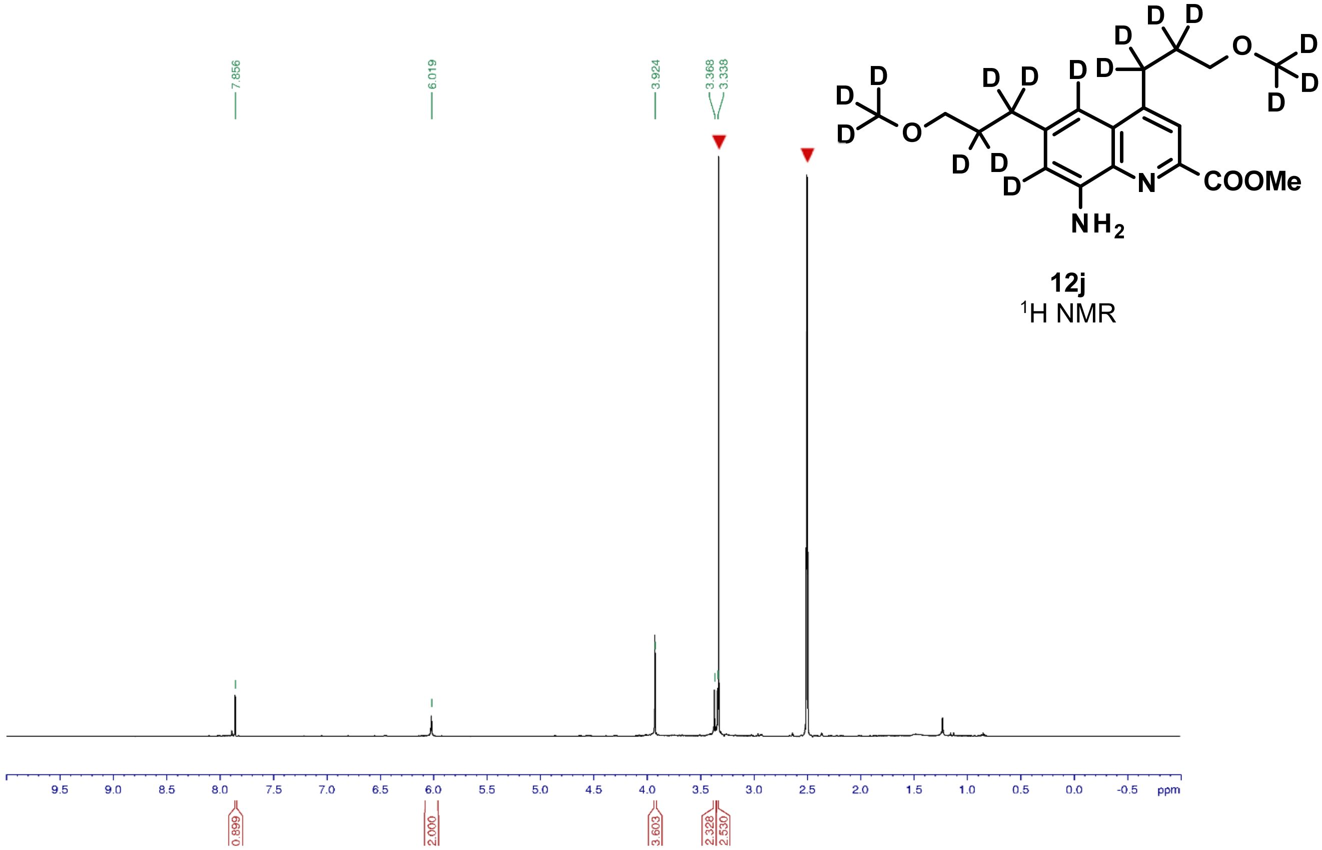

12j
^{1}H NMR

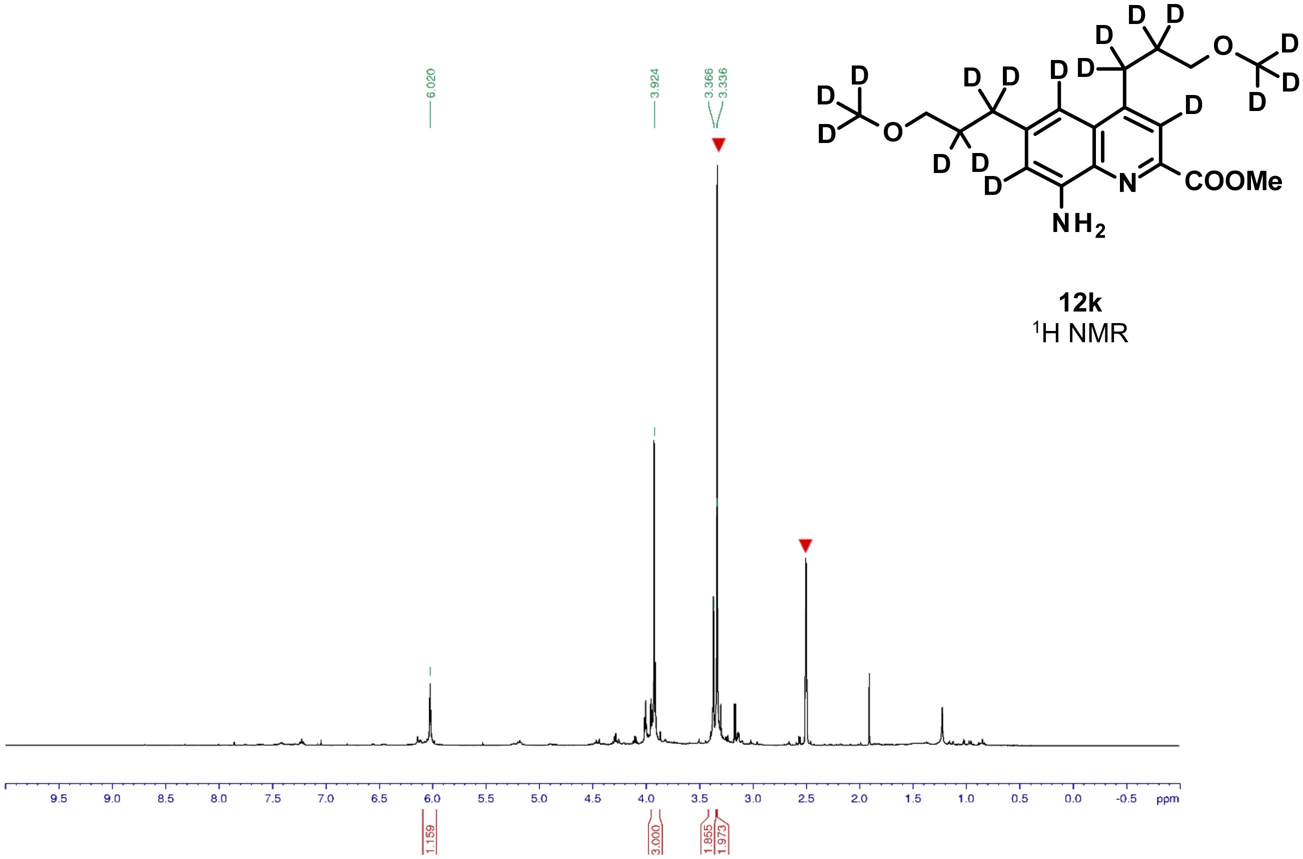

12k
^{1}H NMR

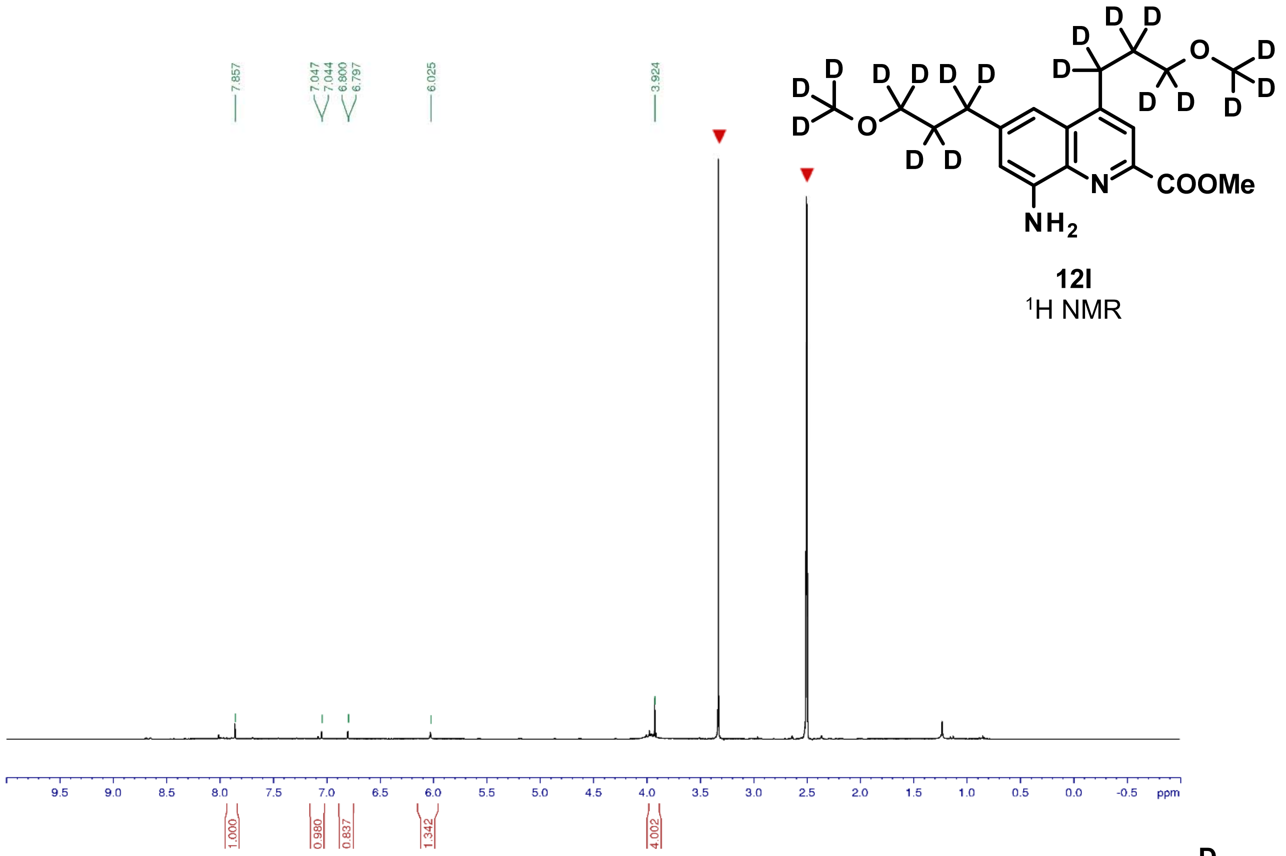

12l

^{1}H NMR

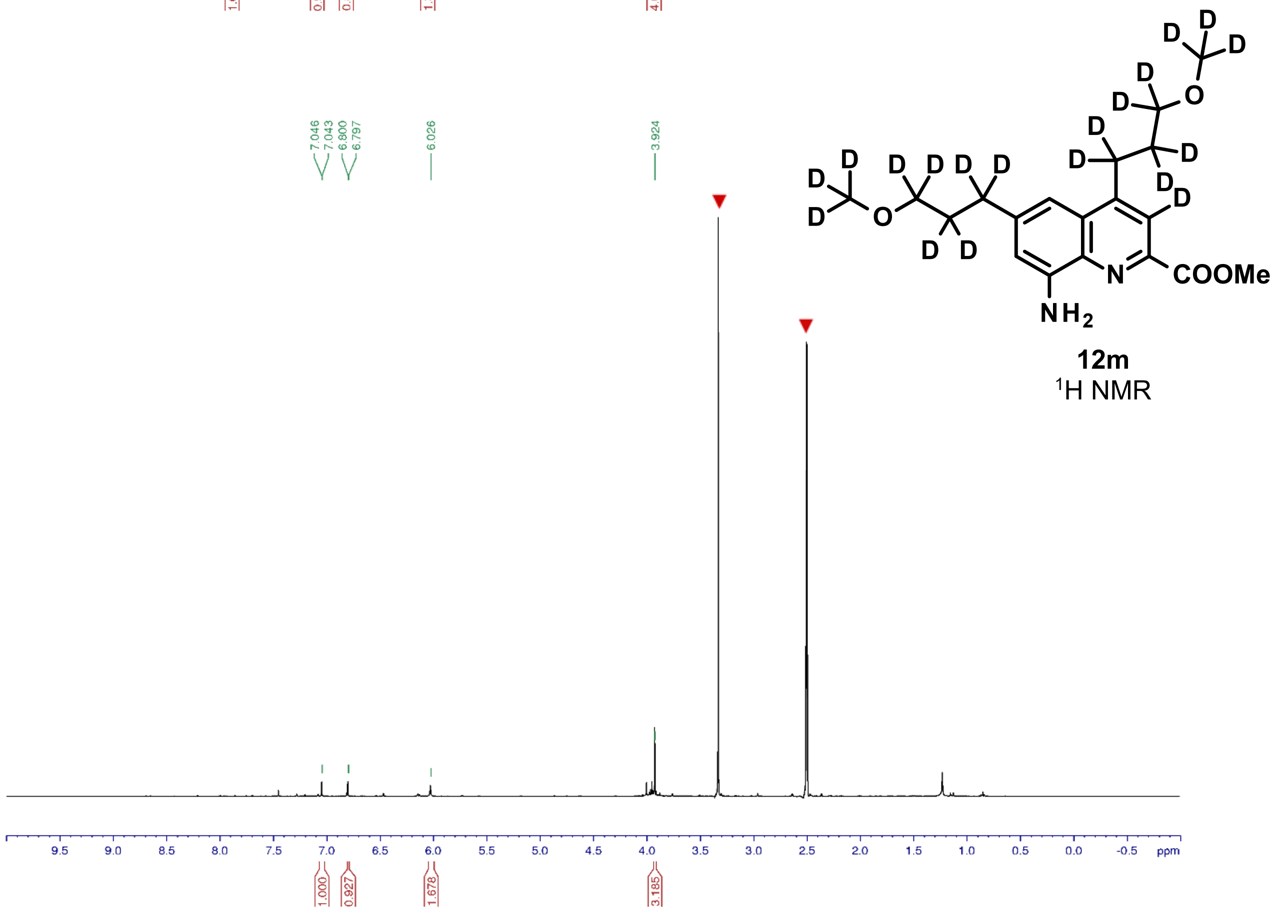

12m

^{1}H NMR

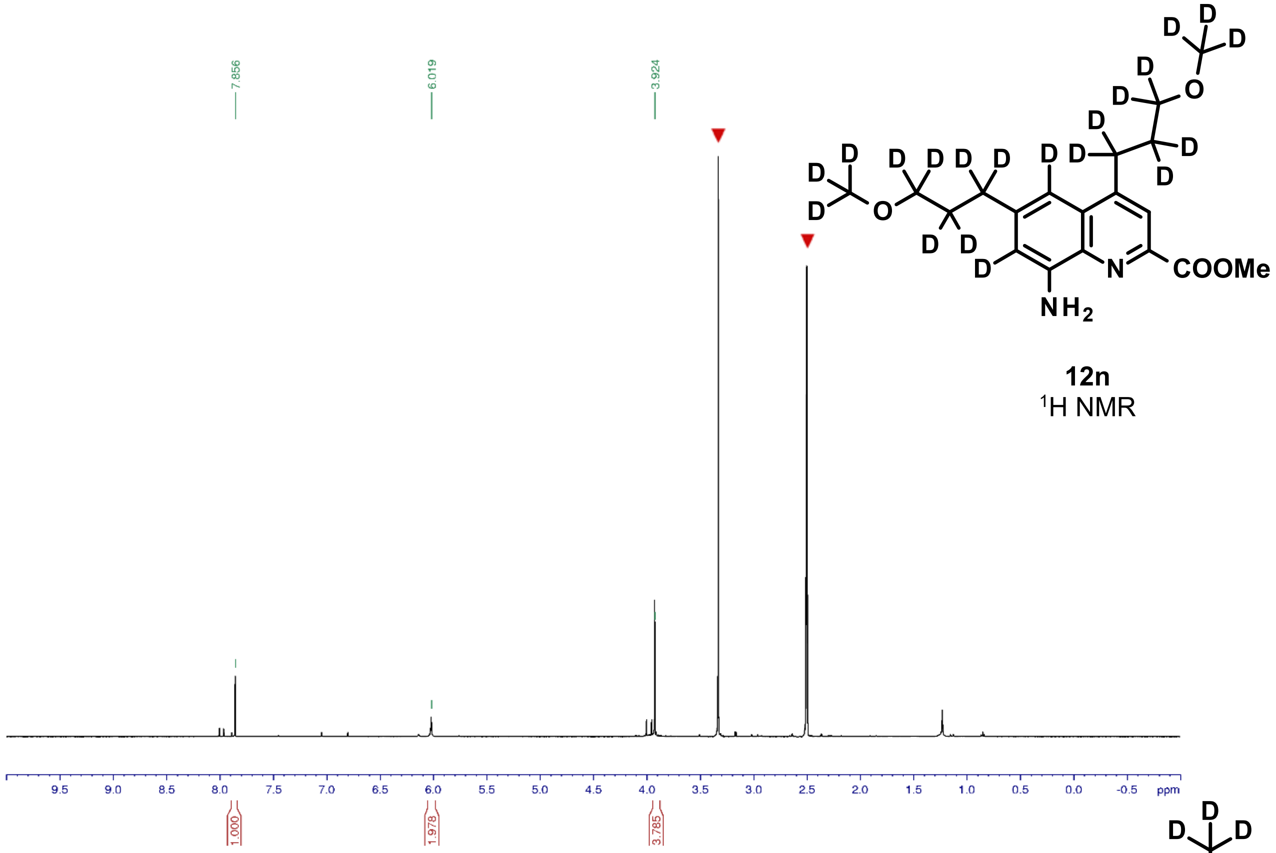

12n
^{1}H NMR

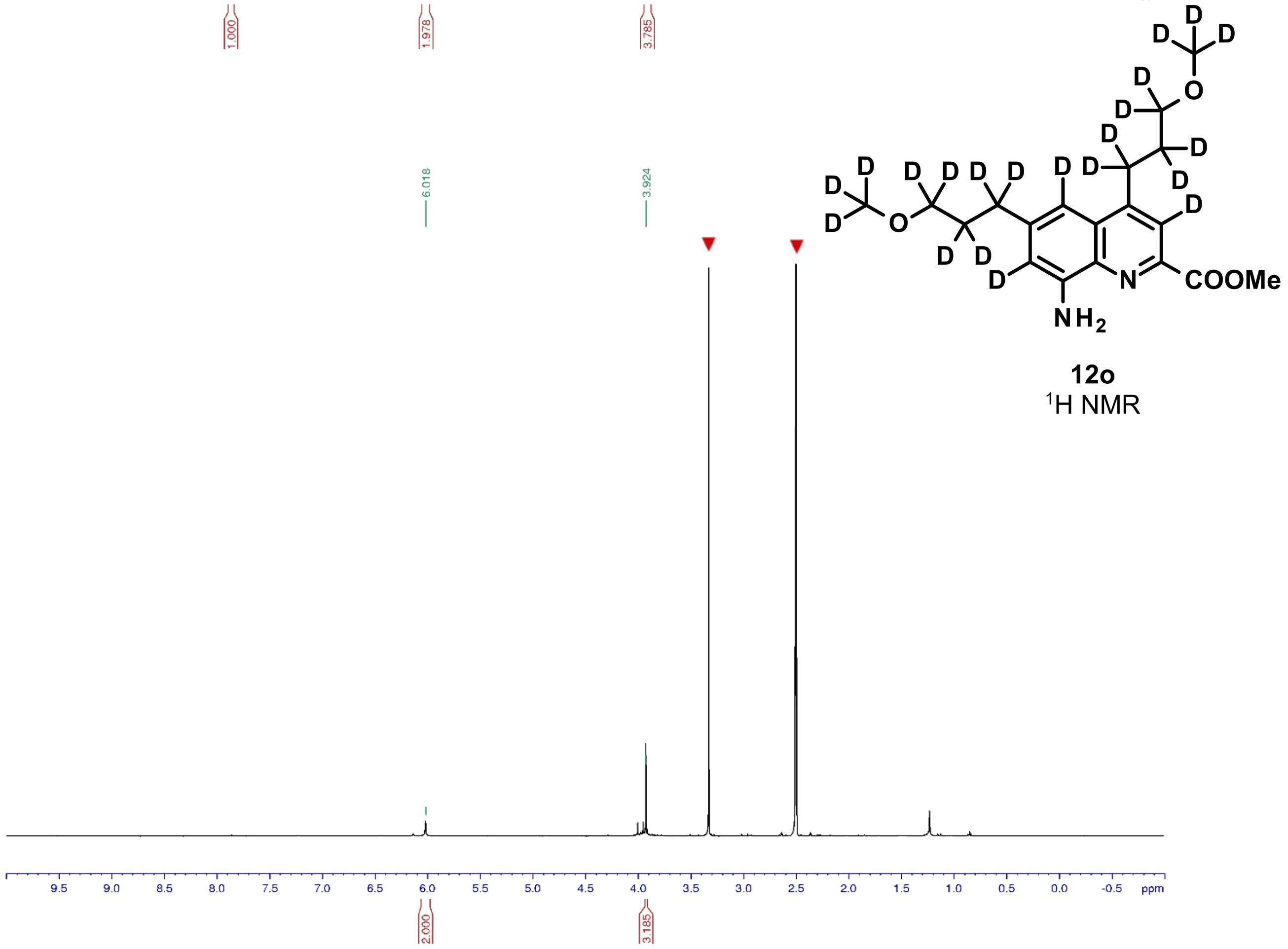

12o
^{1}H NMR

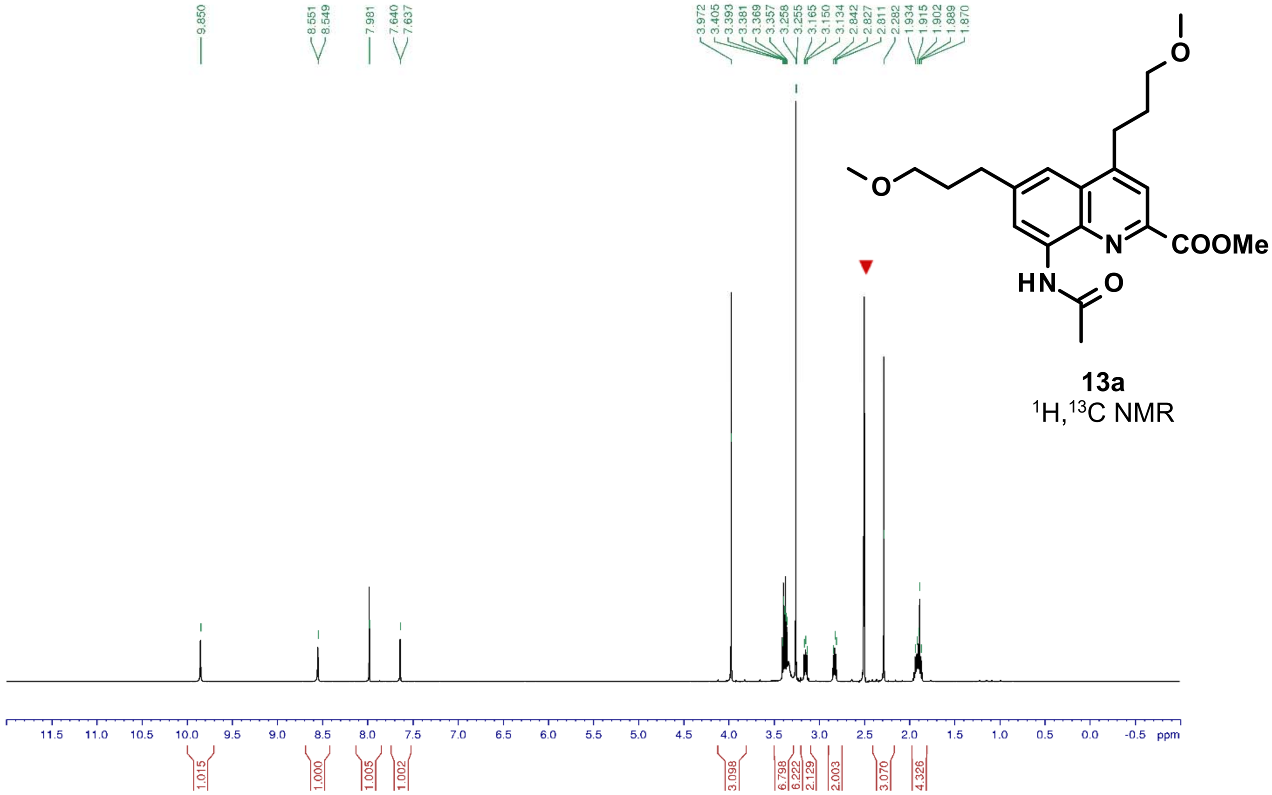

13a

^{1}H,^{13}C NMR

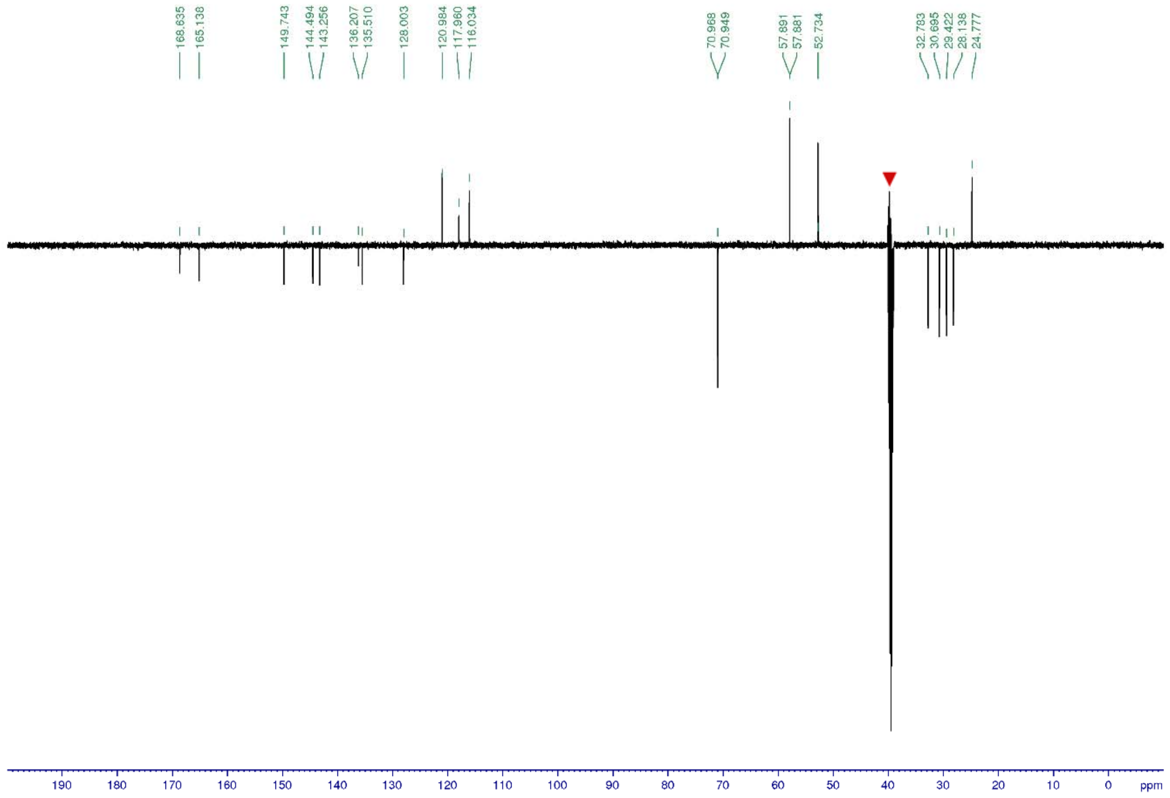

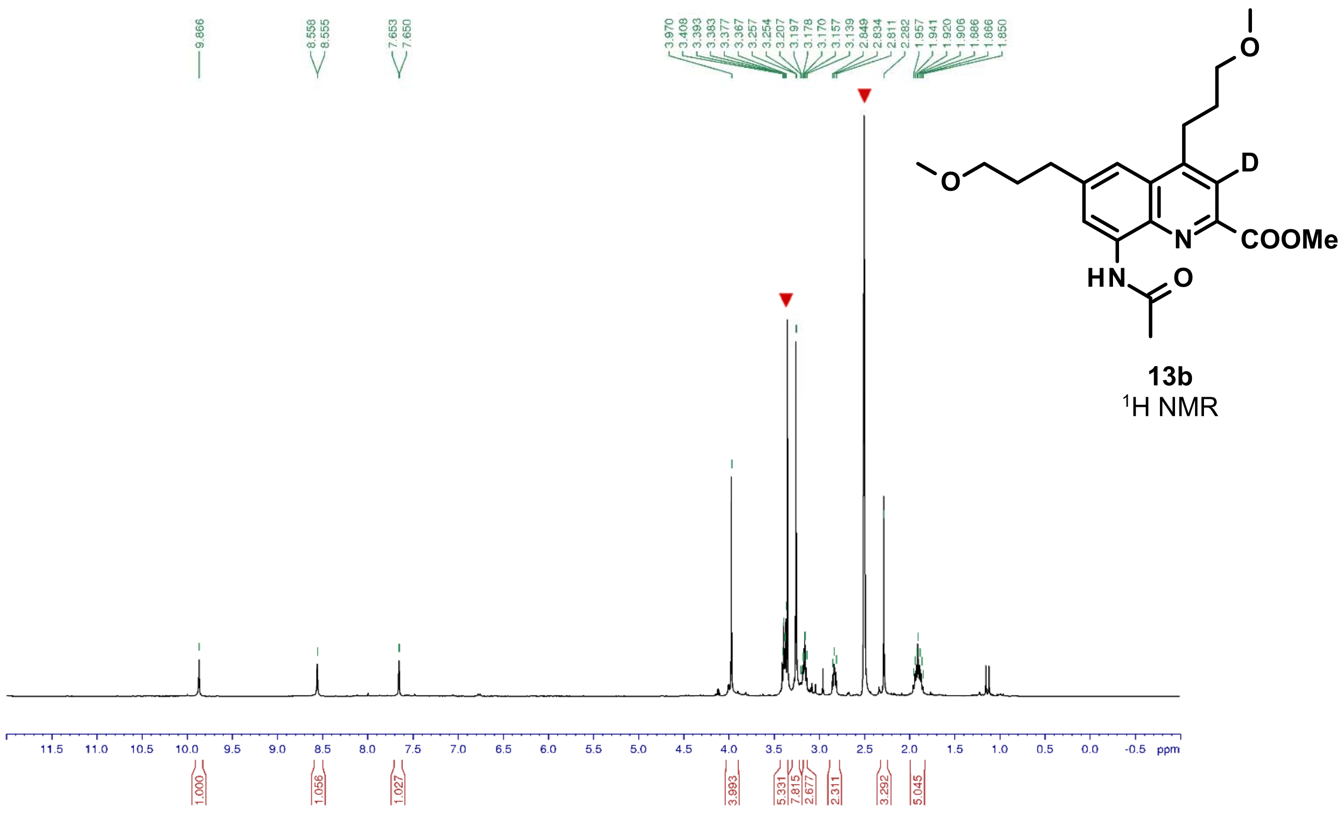

13b
^{1}H NMR

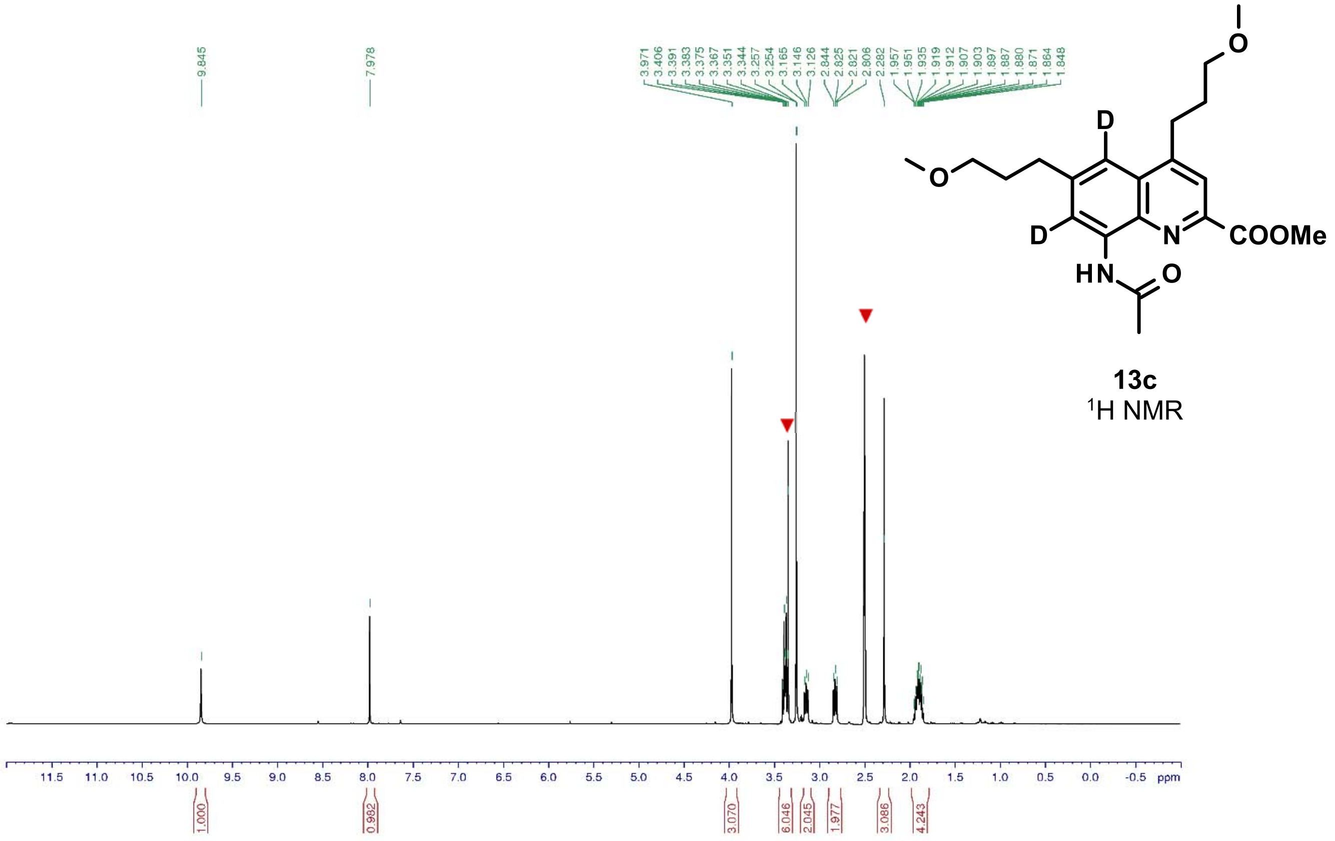

13c
^{1}H NMR

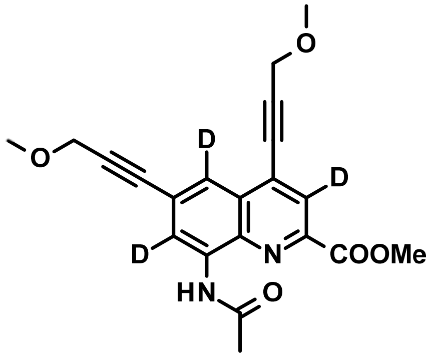

29
^{1}H, ^{13}C NMR

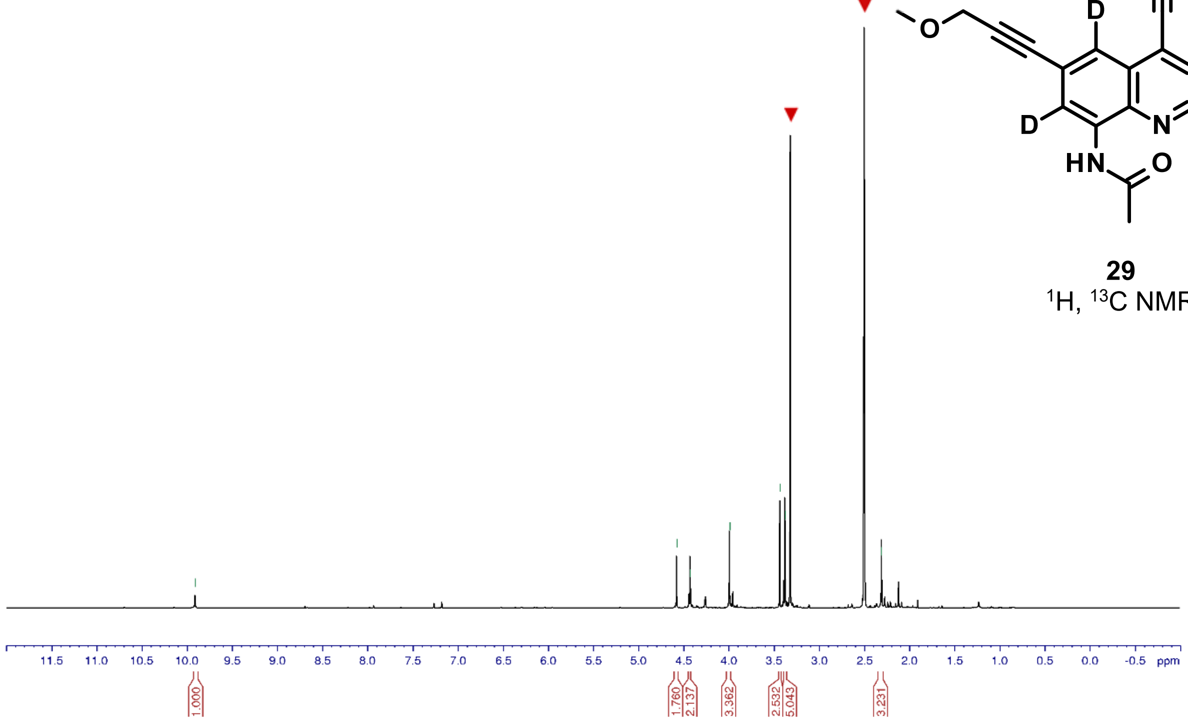
9.910
4.577
4.428
3.992
3.434
3.377
2.310
1.000
1.760
2.137
3.362
2.532
5.043
3.231
ppm

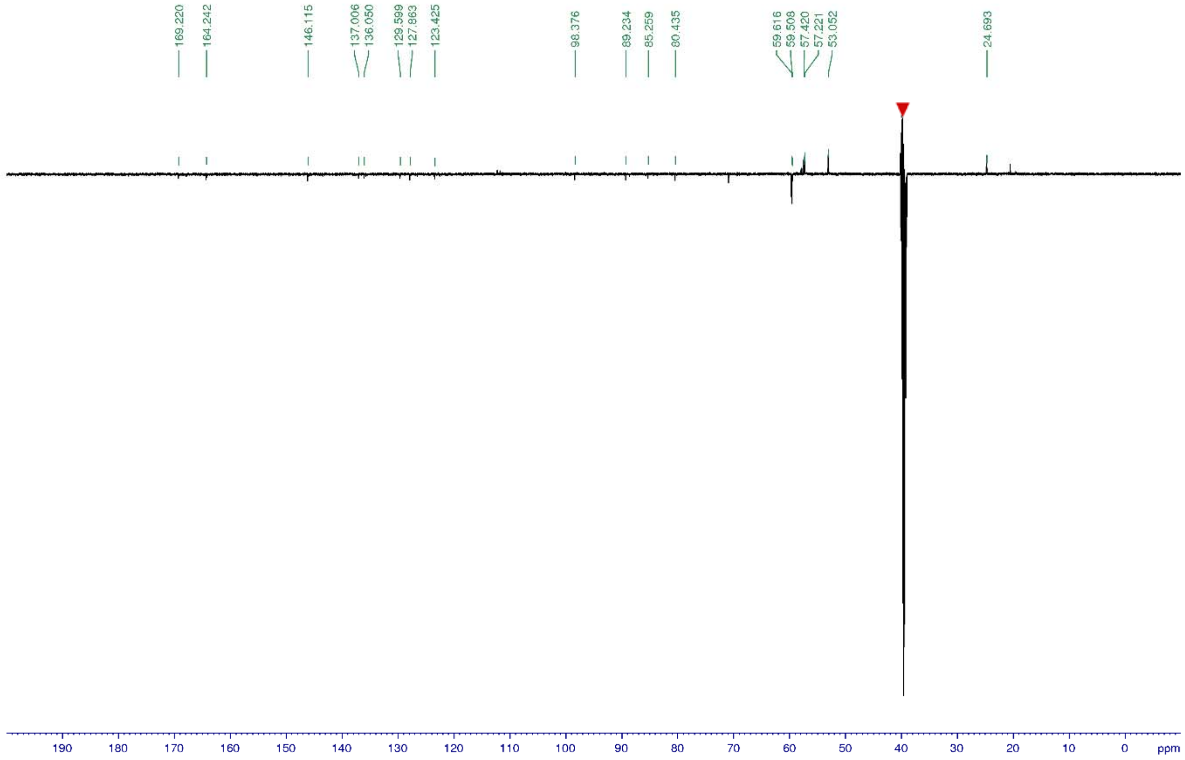
169.220
164.242
146.115
137.006
136.050
129.599
127.863
123.425
98.376
89.234
85.259
80.435
59.616
59.508
57.420
57.221
53.052
24.693
ppm

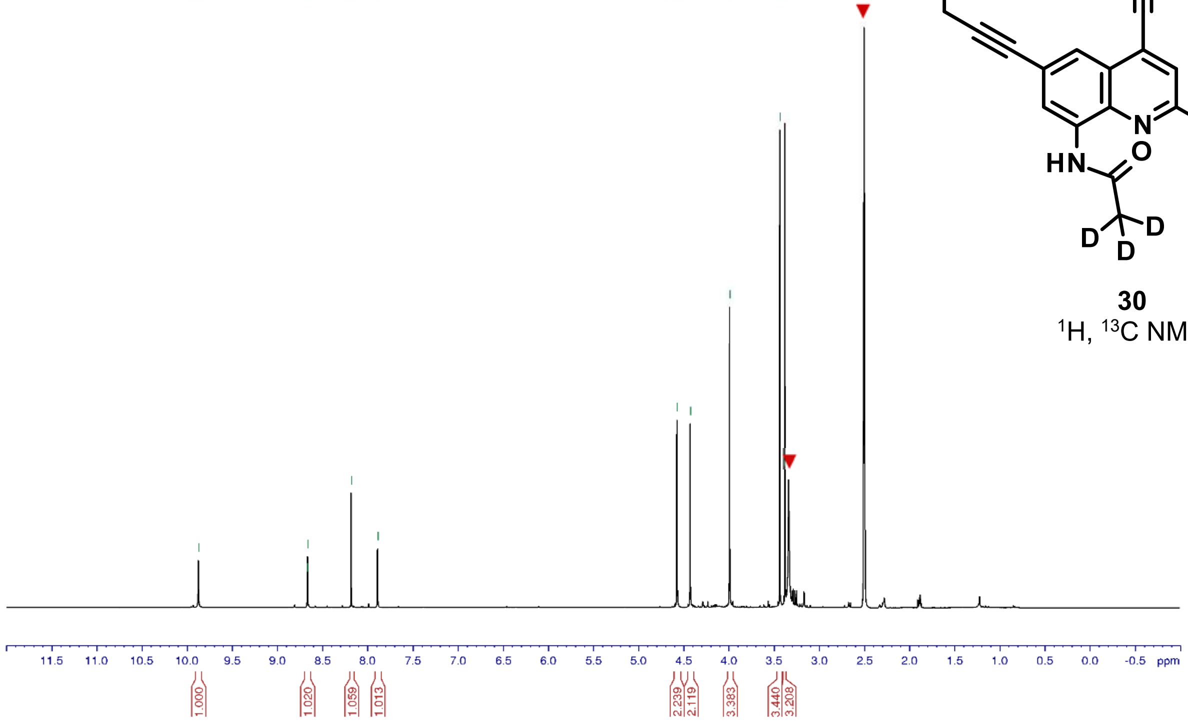
9.874
8.667
8.663
8.182
7.892
7.887
4.574
4.427
3.990
3.434
3.377
11.5 11.0 10.5 10.0 9.5 9.0 8.5 8.0 7.5 7.0 6.5 6.0 5.5 5.0 4.5 4.0 3.5 3.0 2.5 2.0 1.5 1.0 0.5 0.0 -0.5 ppm
1.000
1.020
1.059
1.013
2.239
2.119
3.383
3.440
3.208

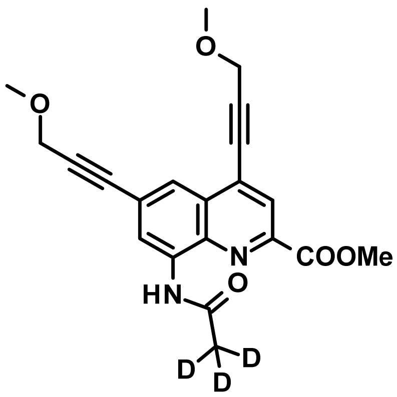
O
O
N
COOMe
HN
O
D
D
D

30
^{1}H, ^{13}C NMR

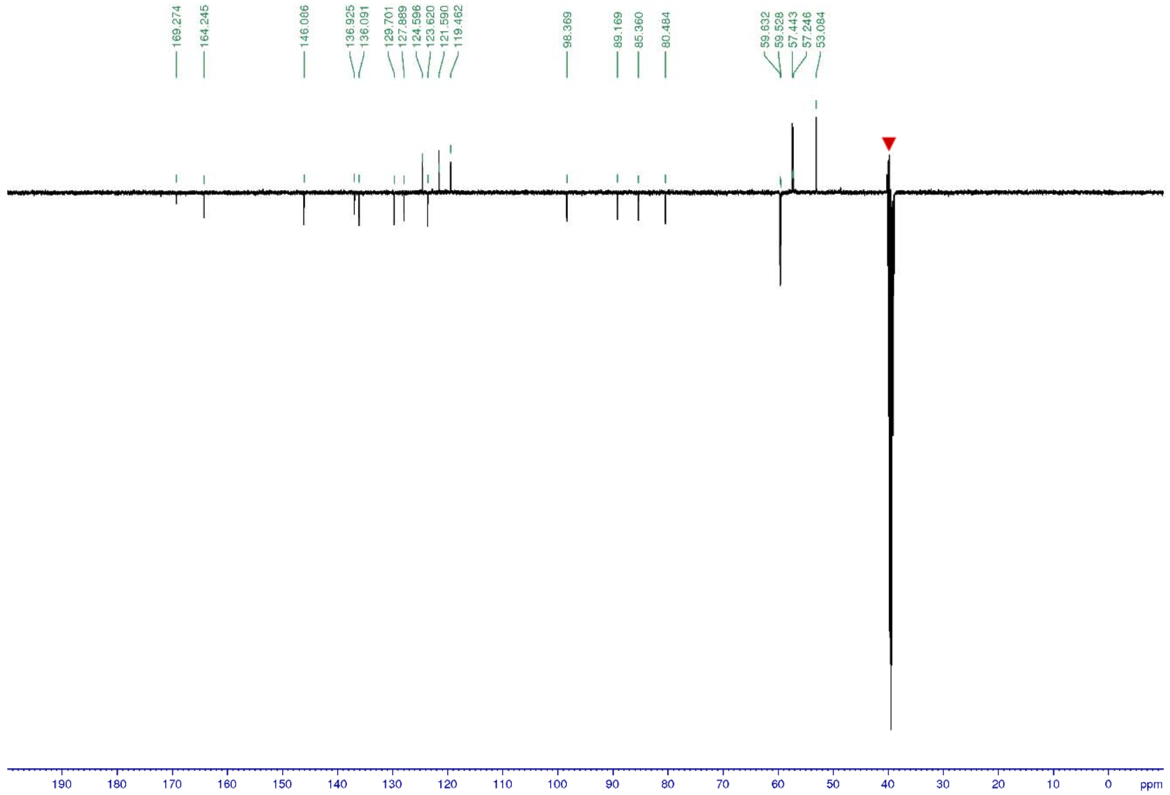
169.274
164.245
146.086
136.925
136.091
129.701
127.889
124.596
123.620
121.590
119.462
98.369
89.169
85.360
80.484
59.632
59.528
57.443
57.246
53.084
190 180 170 160 150 140 130 120 110 100 90 80 70 60 50 40 30 20 10 0 ppm

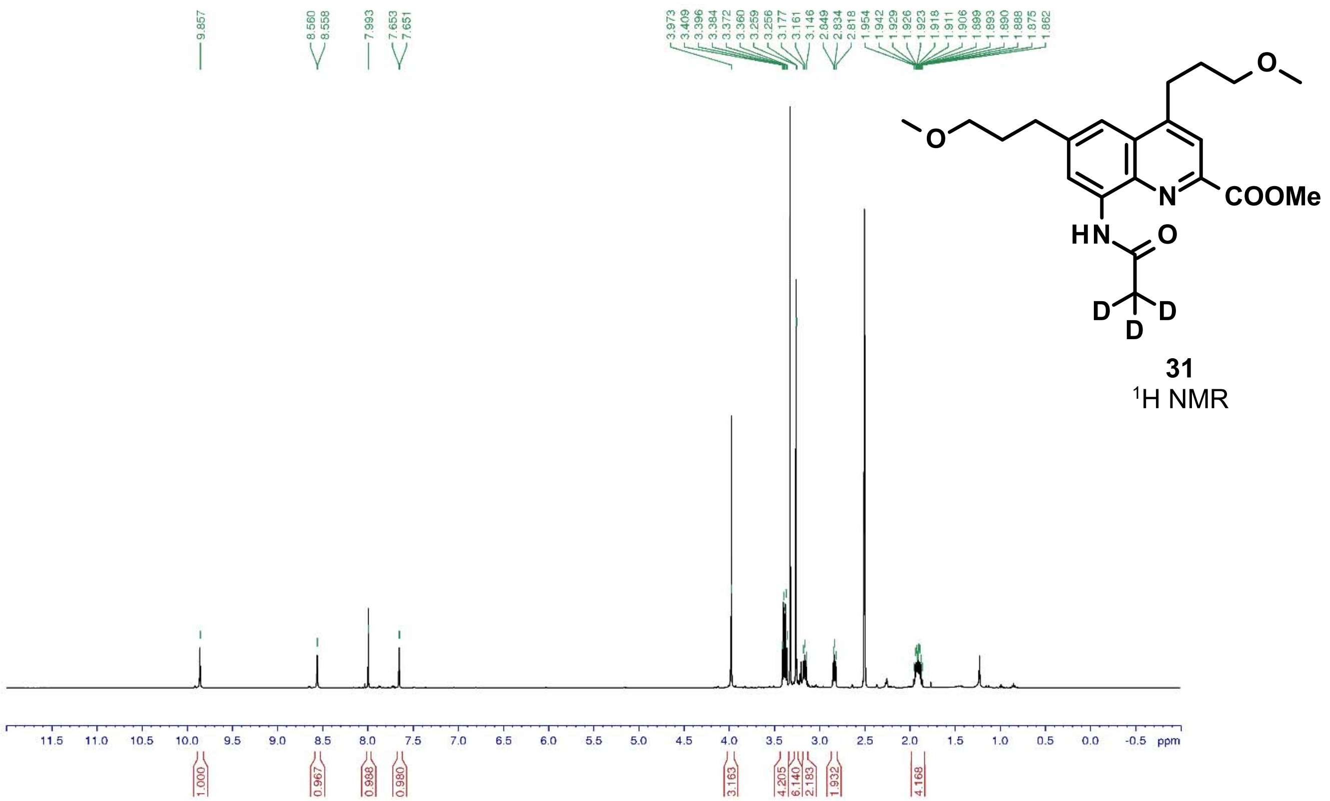

31
^{1}H NMR

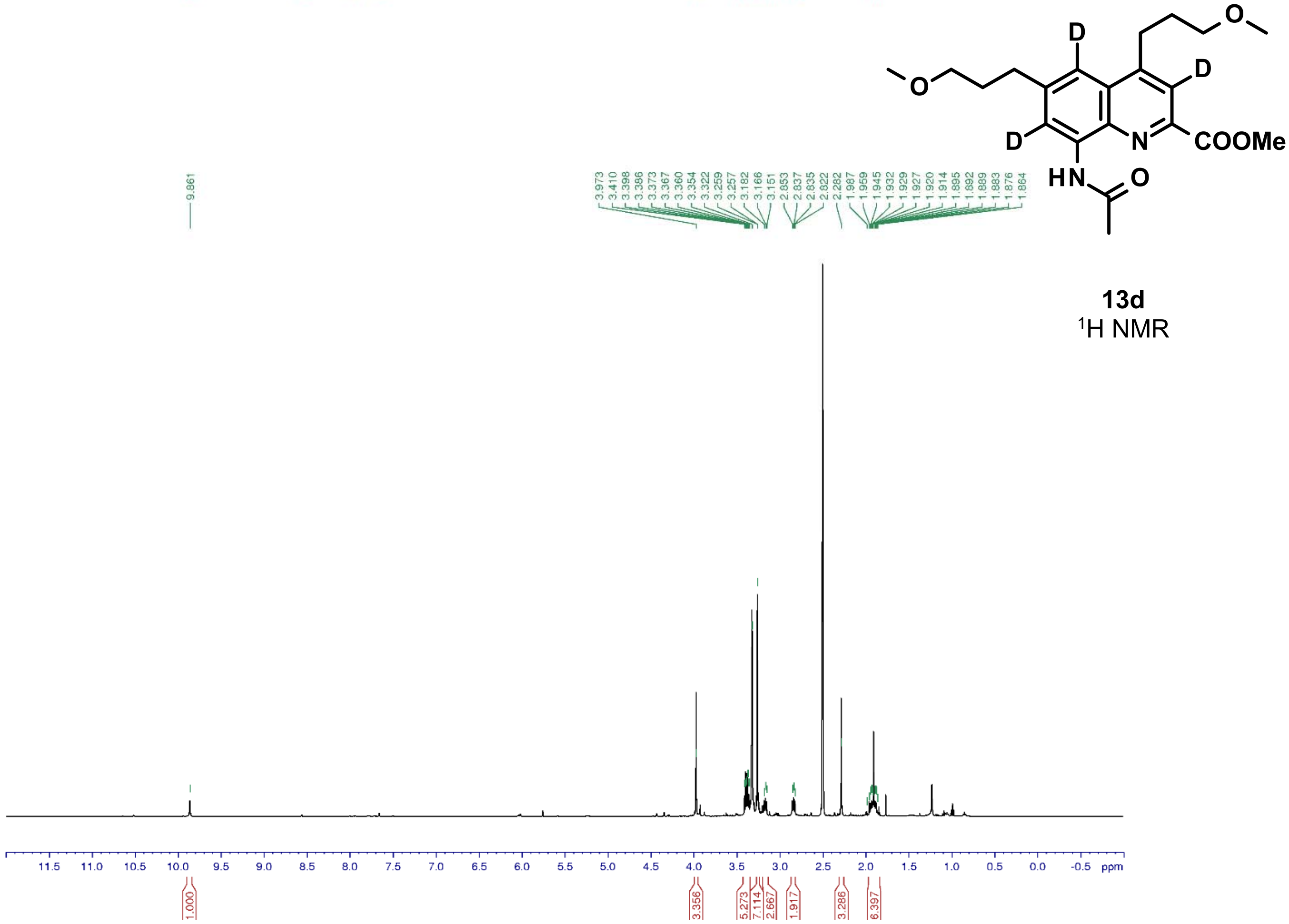

13d
^{1}H NMR

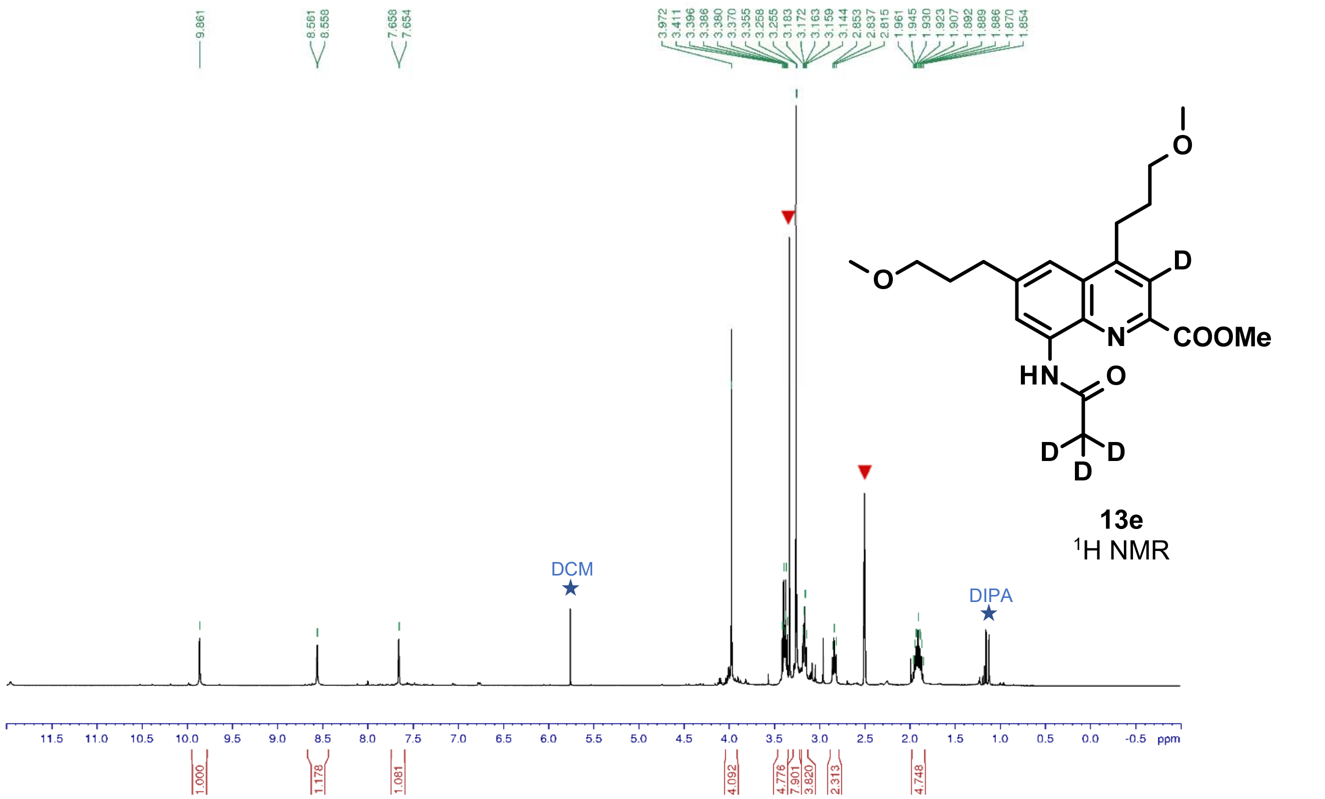
DCM
DIPA
13e
1H NMR

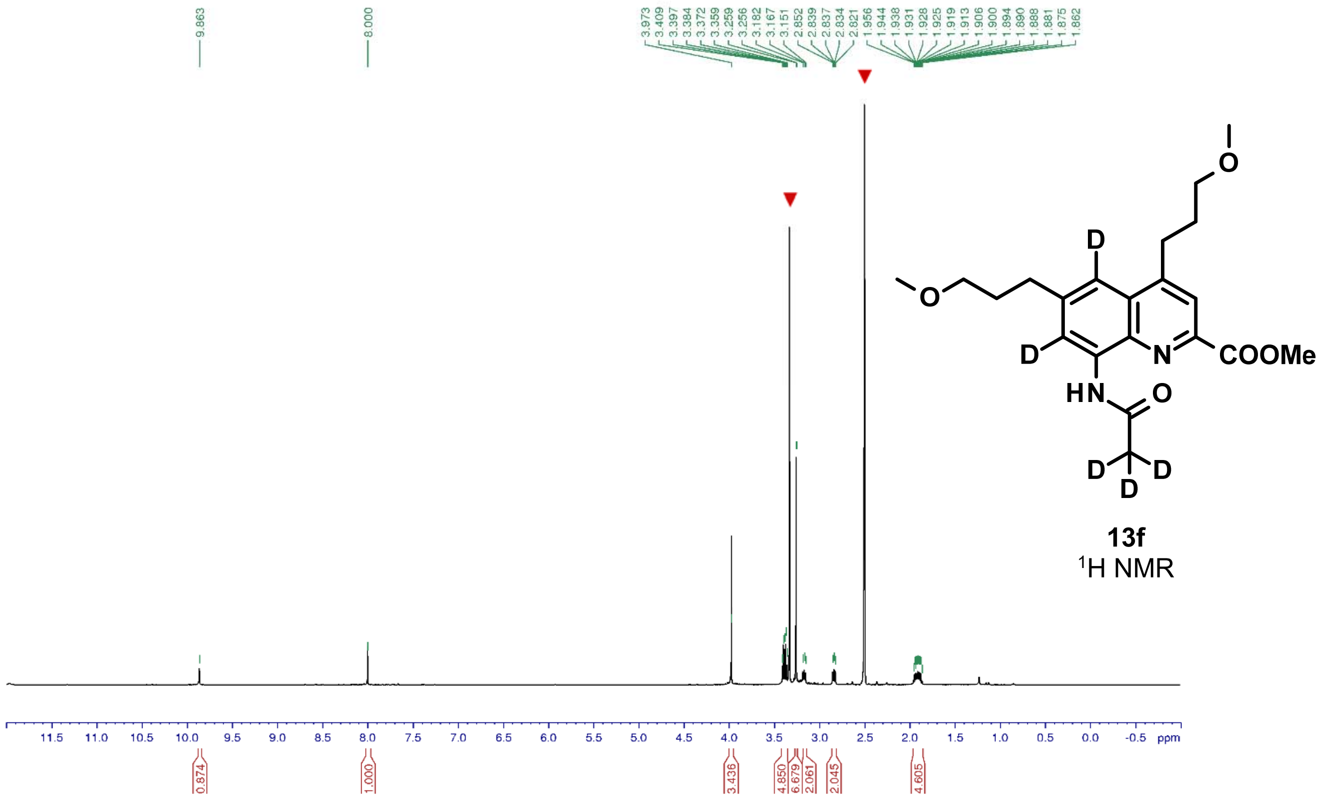
13f
1H NMR

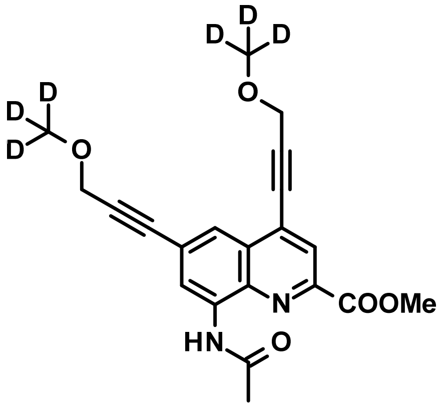

D
D
D
D
D
D
O
O
N
COOMe
HN
O

32

^{1}H, ^{13}C NMR

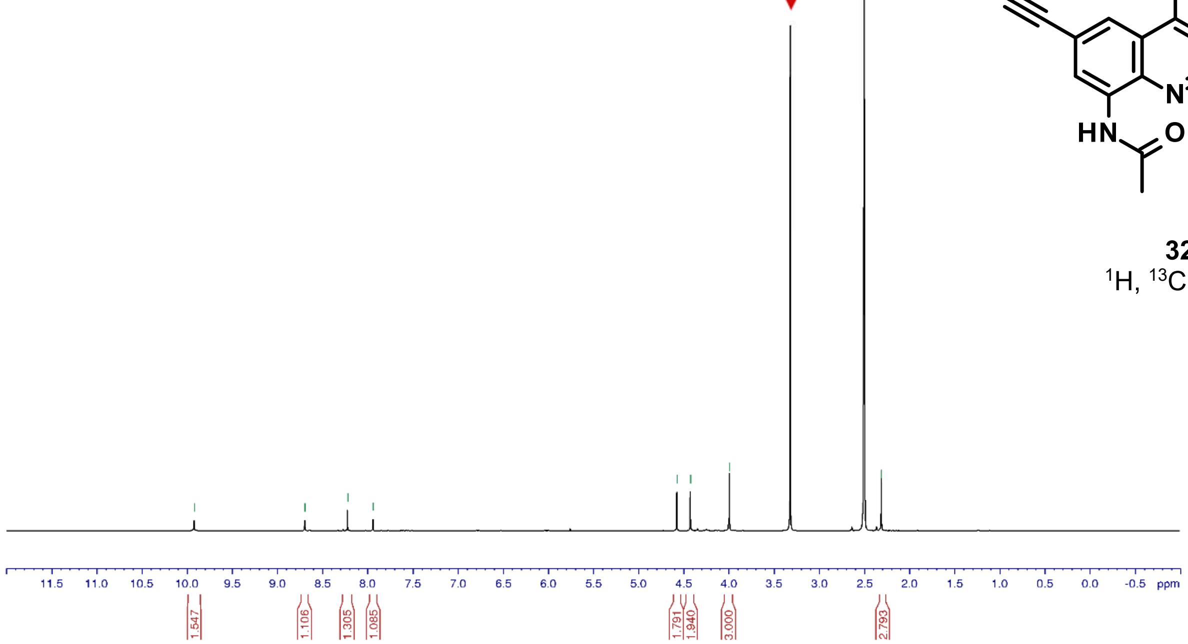

9.920
8.697
8.694
8.222
7.942
7.939
4.575
4.425
3.992
2.310
11.5 11.0 10.5 10.0 9.5 9.0 8.5 8.0 7.5 7.0 6.5 6.0 5.5 5.0 4.5 4.0 3.5 3.0 2.5 2.0 1.5 1.0 0.5 0.0 -0.5 ppm
1.547
1.106
1.305
1.085
1.791
1.940
3.000
2.793

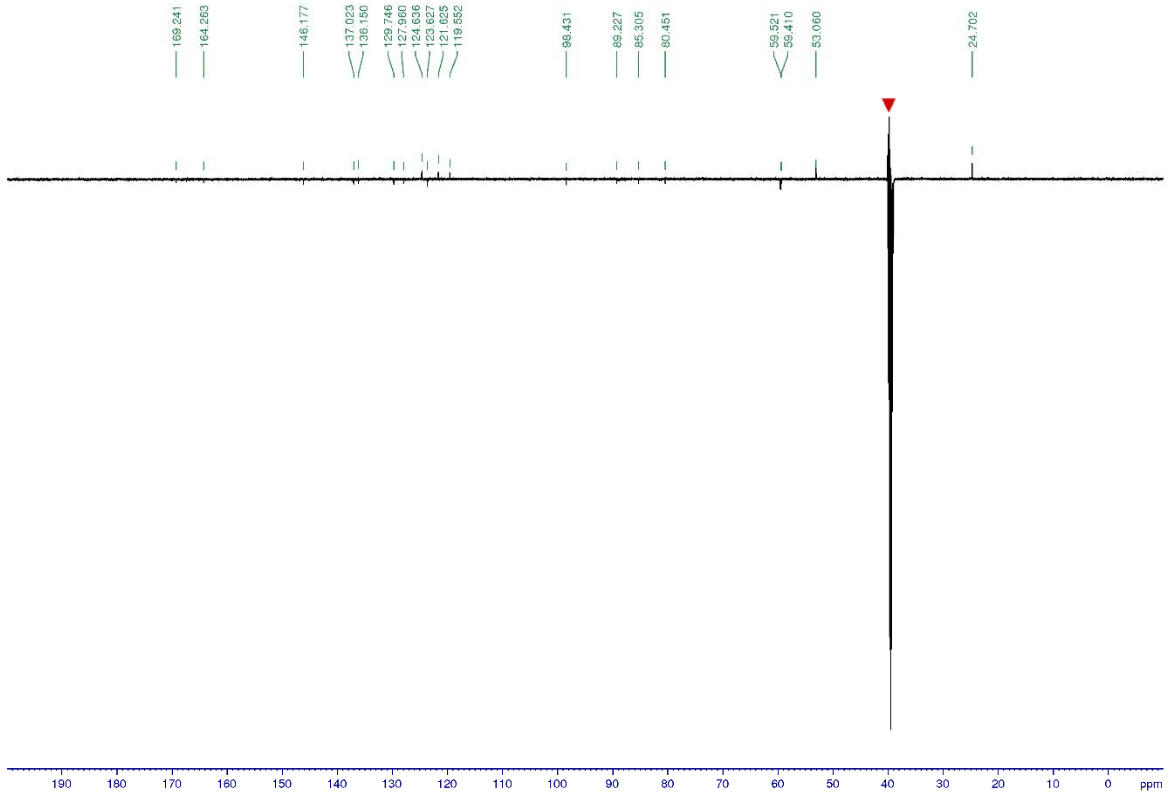

169.241
164.263
146.177
137.023
136.150
129.746
127.960
124.636
123.627
121.825
119.552
98.431
89.227
85.305
80.451
59.521
59.410
53.060
24.702
190 180 170 160 150 140 130 120 110 100 90 80 70 60 50 40 30 20 10 0 ppm

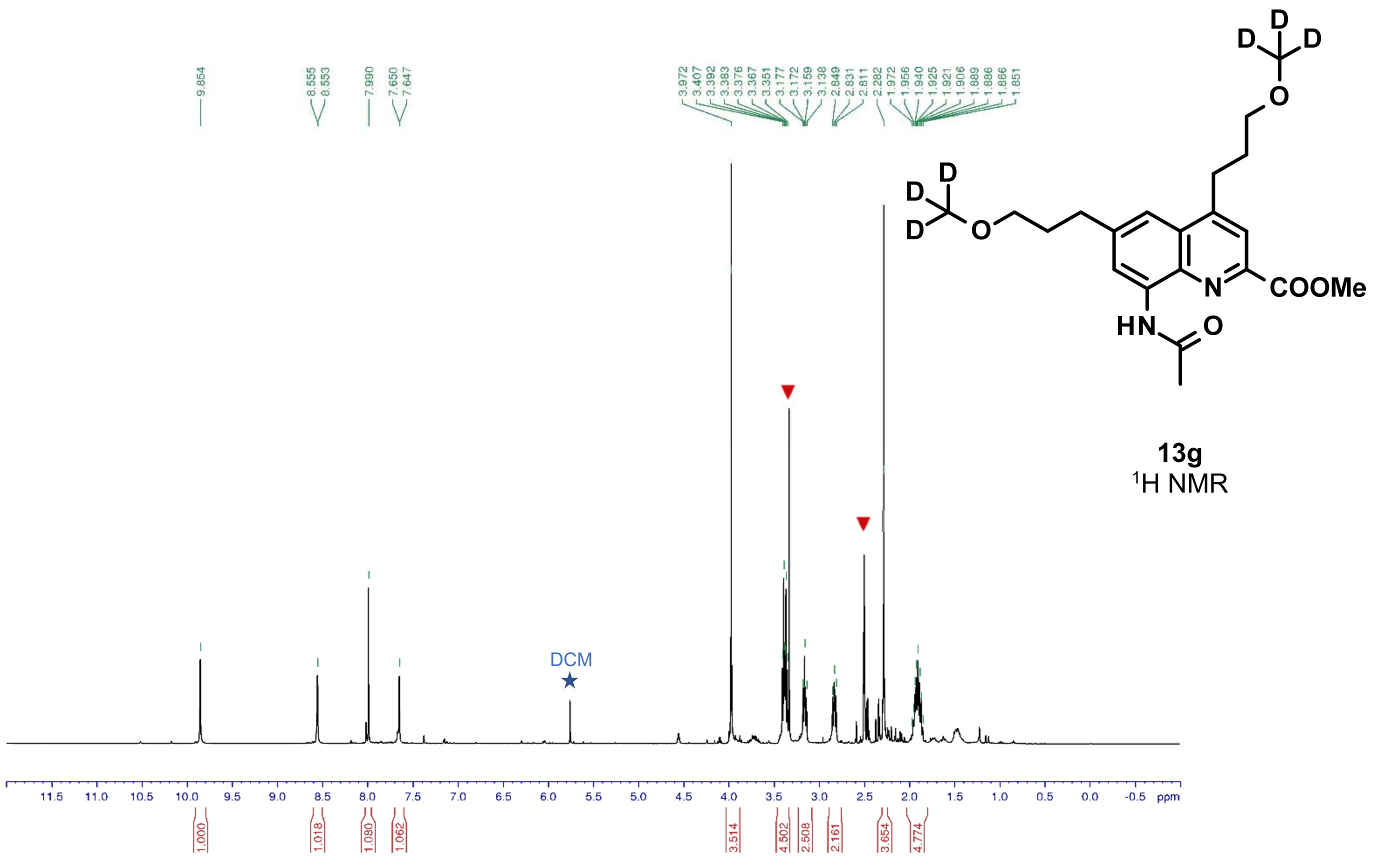

DCM
13g
1H NMR

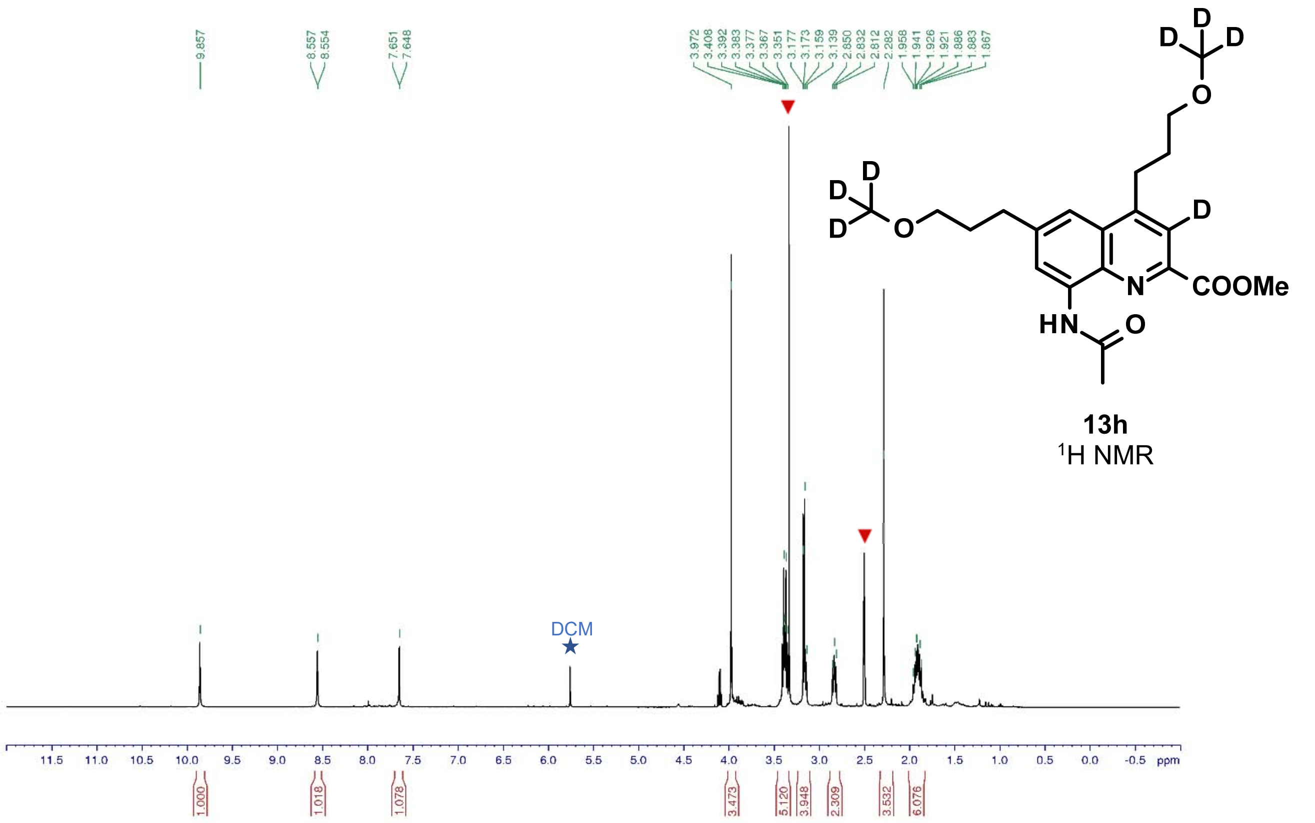

DCM
13h
1H NMR

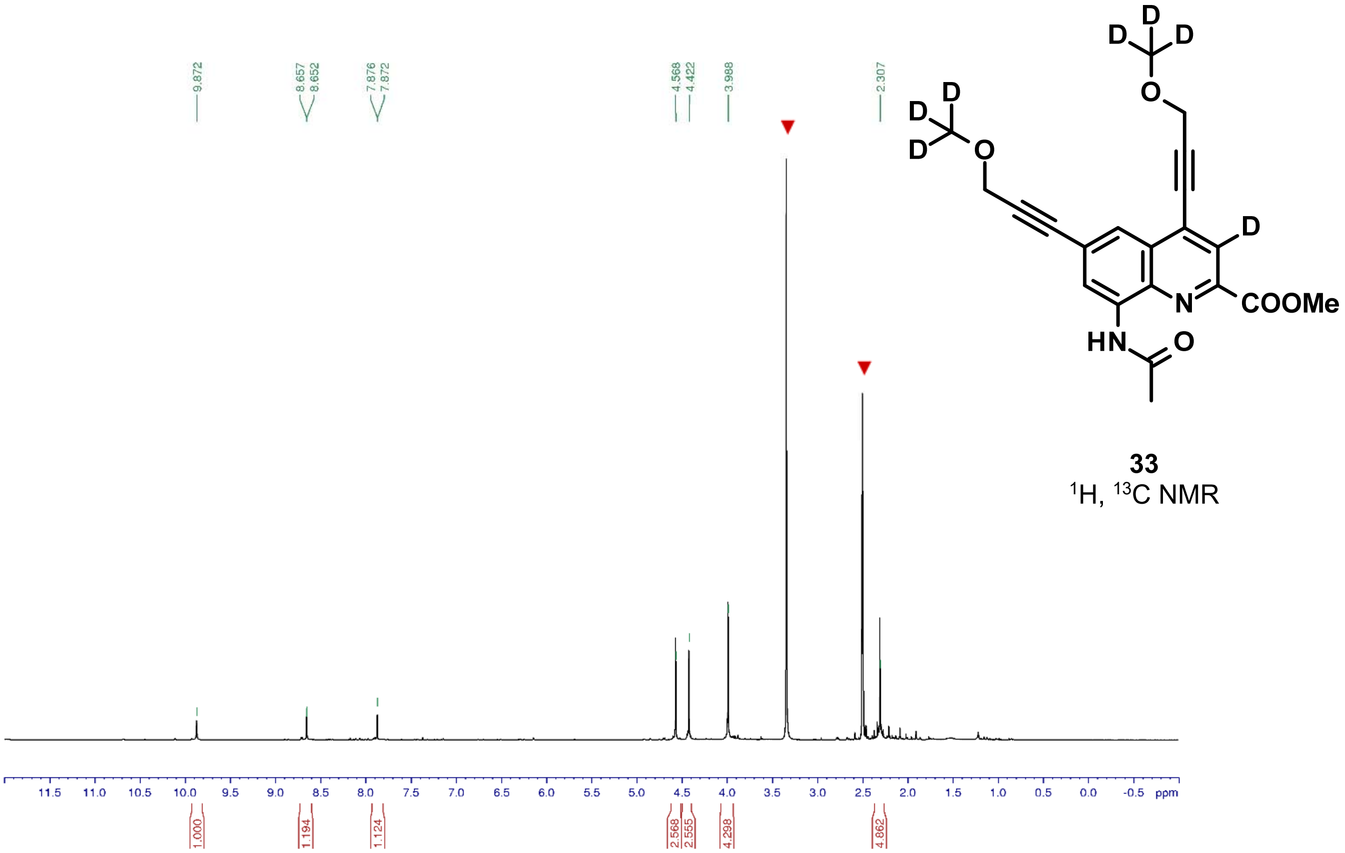
9.872
8.657
8.652
7.876
7.872
4.568
4.422
3.988
2.307
11.5 11.0 10.5 10.0 9.5 9.0 8.5 8.0 7.5 7.0 6.5 6.0 5.5 5.0 4.5 4.0 3.5 3.0 2.5 2.0 1.5 1.0 0.5 0.0 -0.5 ppm
1.000
1.194
1.124
2.568
2.555
4.298
4.862
D
D
D
O
D
D
D
O
D
N
COOMe
HN
O

33

^{1}H, ^{13}C NMR

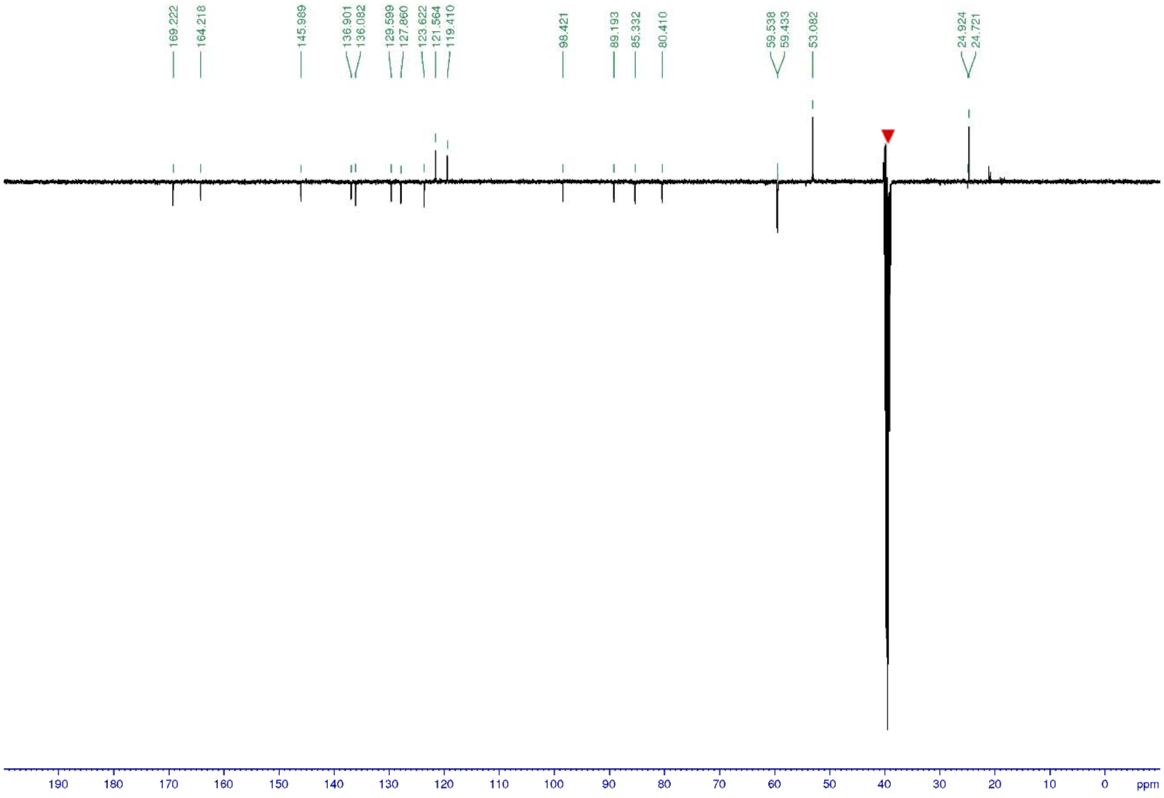
169.222
164.218
145.989
136.901
136.082
129.599
127.860
123.622
121.564
119.410
98.421
89.193
85.332
80.410
59.538
59.433
53.082
24.924
24.721
190 180 170 160 150 140 130 120 110 100 90 80 70 60 50 40 30 20 10 0 ppm

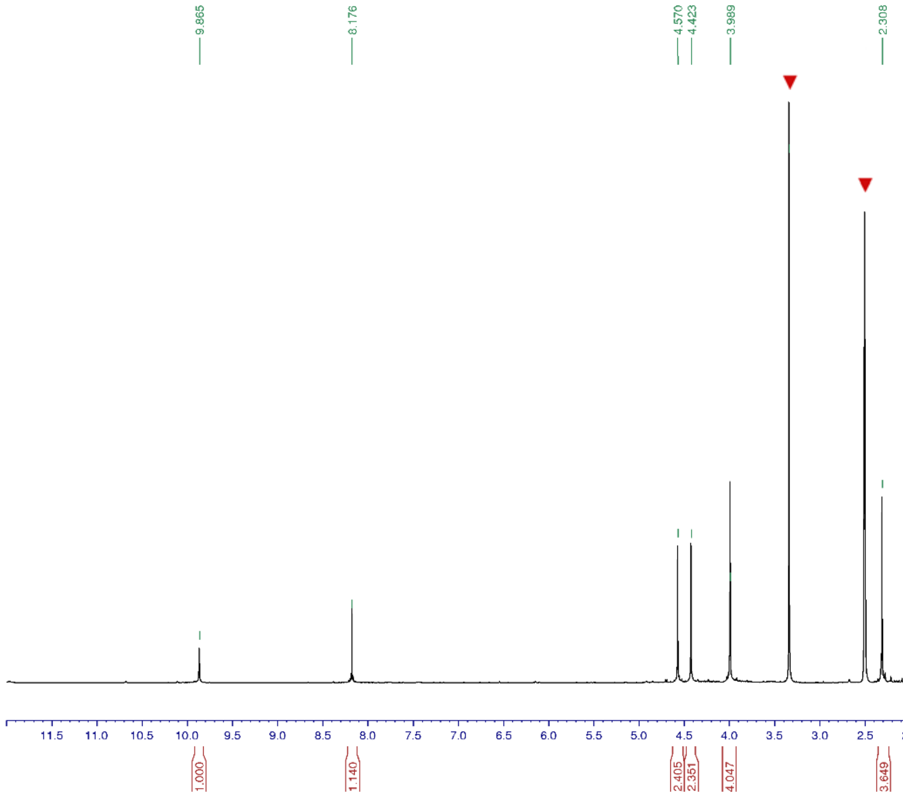
9.865
8.176
4.570
4.423
3.989
2.308
1.000
1.140
2.405
2.351
4.047
3.649
ppm

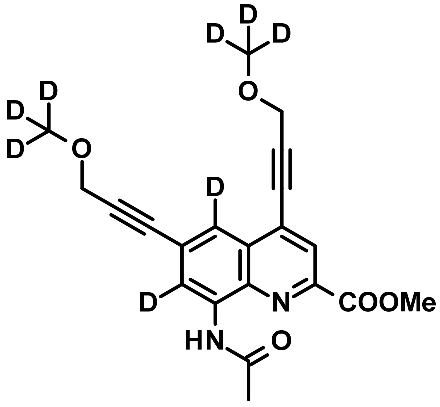
D
D
D
O
D
D
D
O
D
D
N
COOMe
HN
O

34
^1H,^{13}C NMR

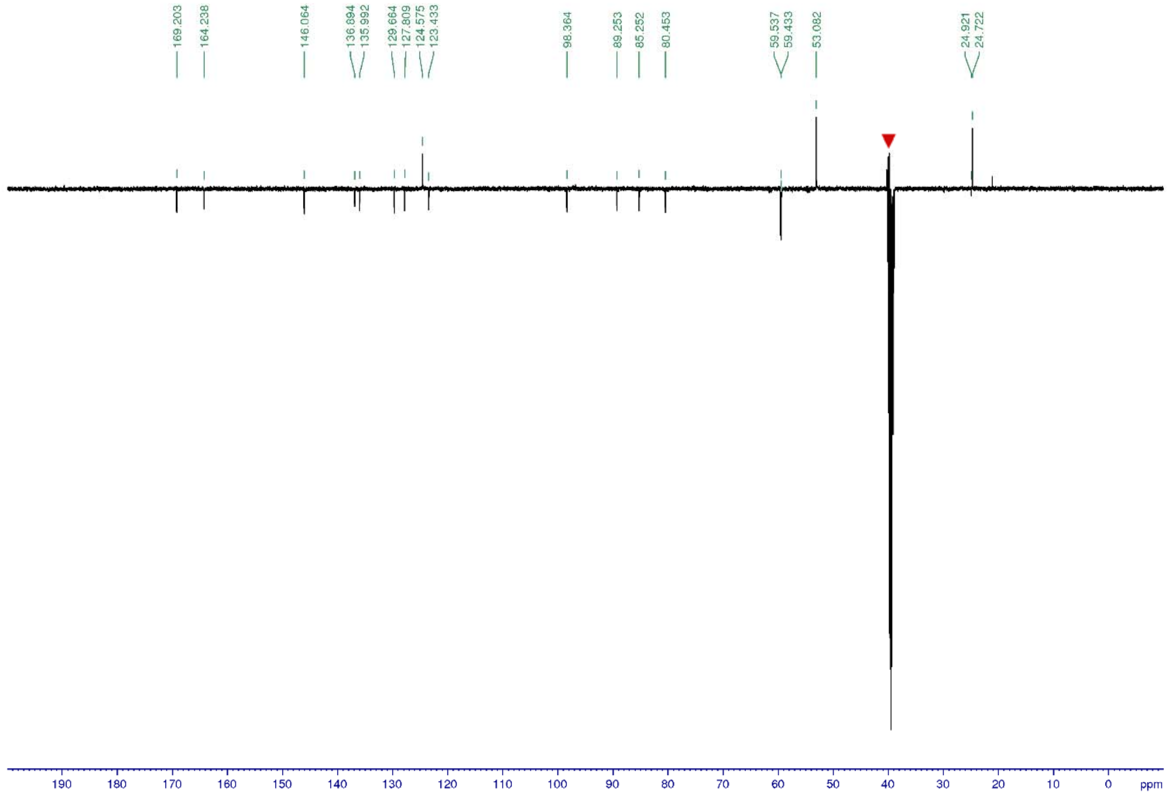
169.203
164.238
146.064
136.894
135.992
129.664
127.809
124.575
123.433
98.364
89.253
85.252
80.453
59.537
59.433
53.082
24.921
24.722
ppm

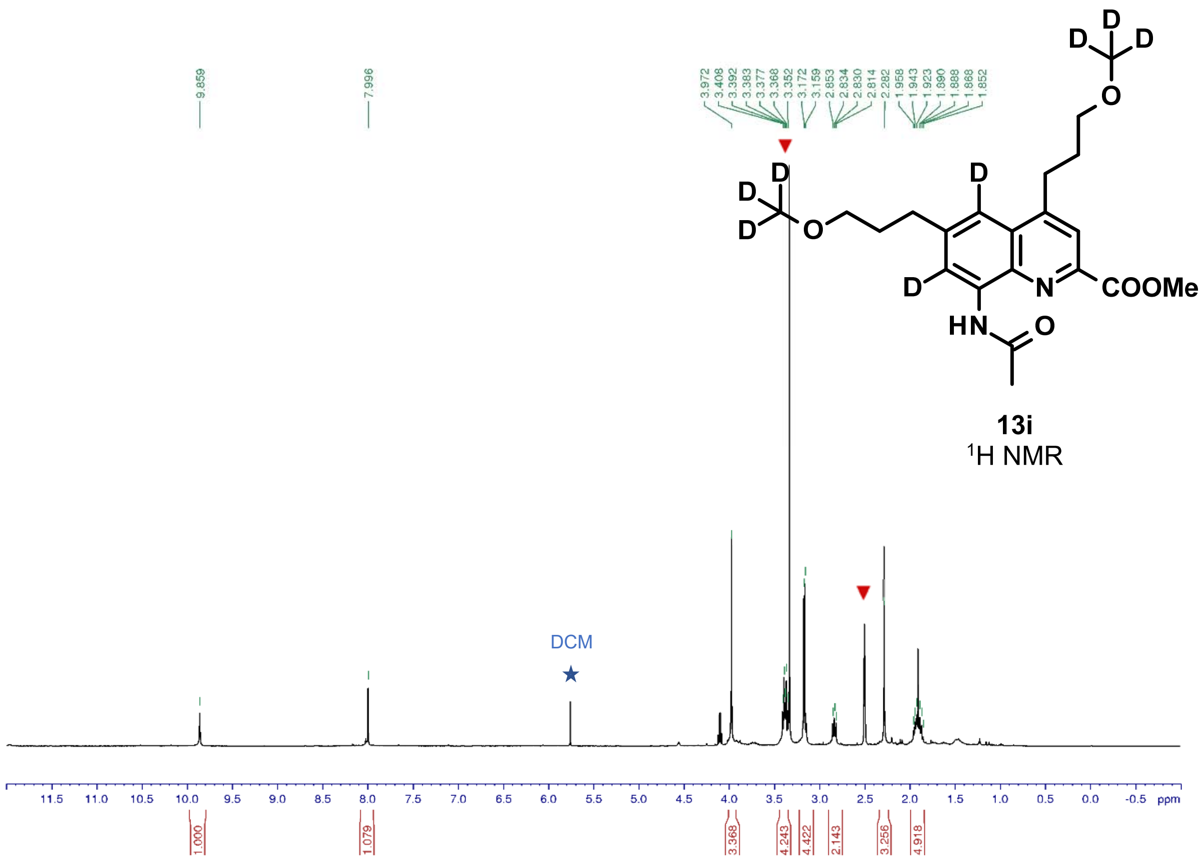

13i
^{1}H NMR

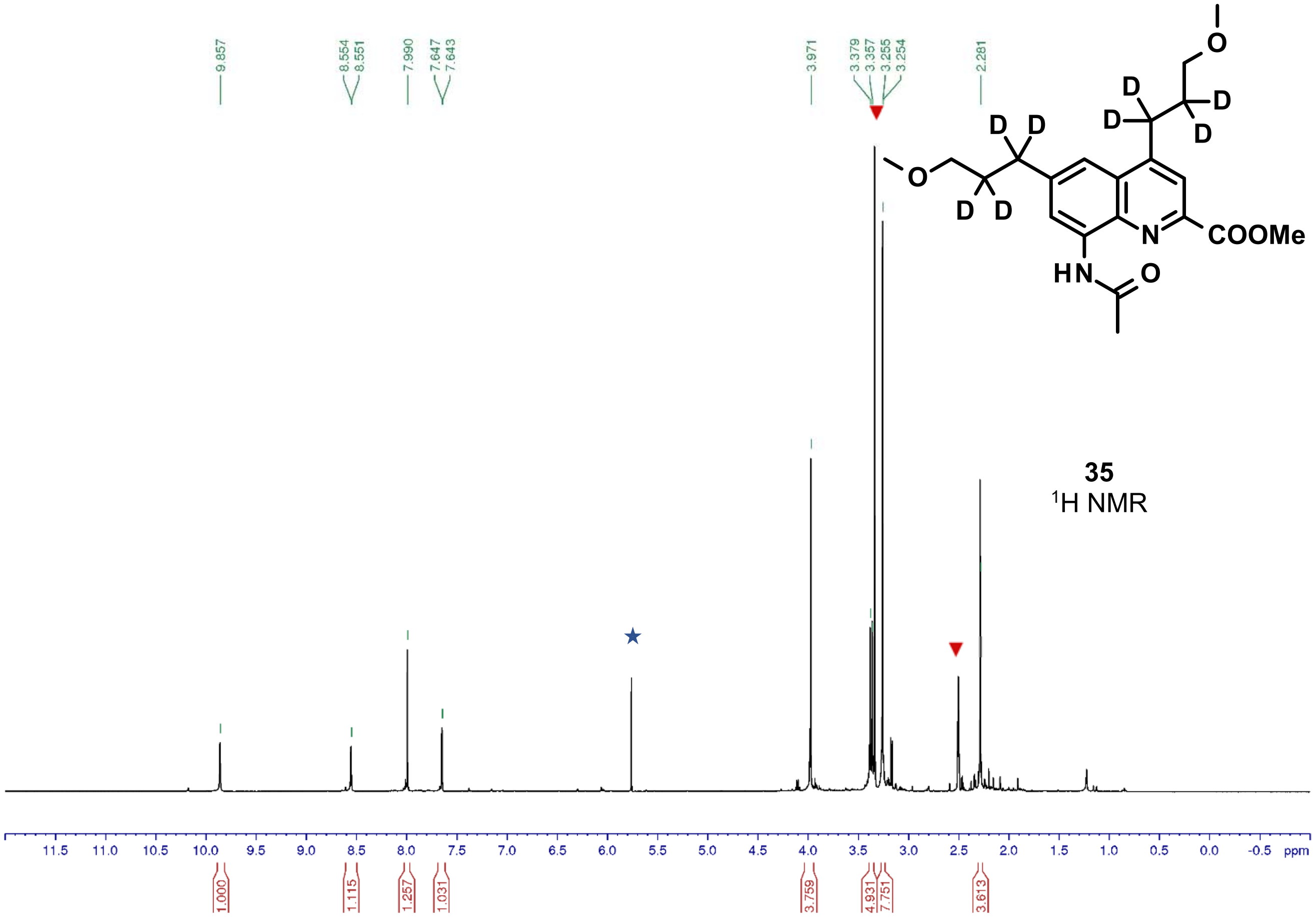

35
^{1}H NMR

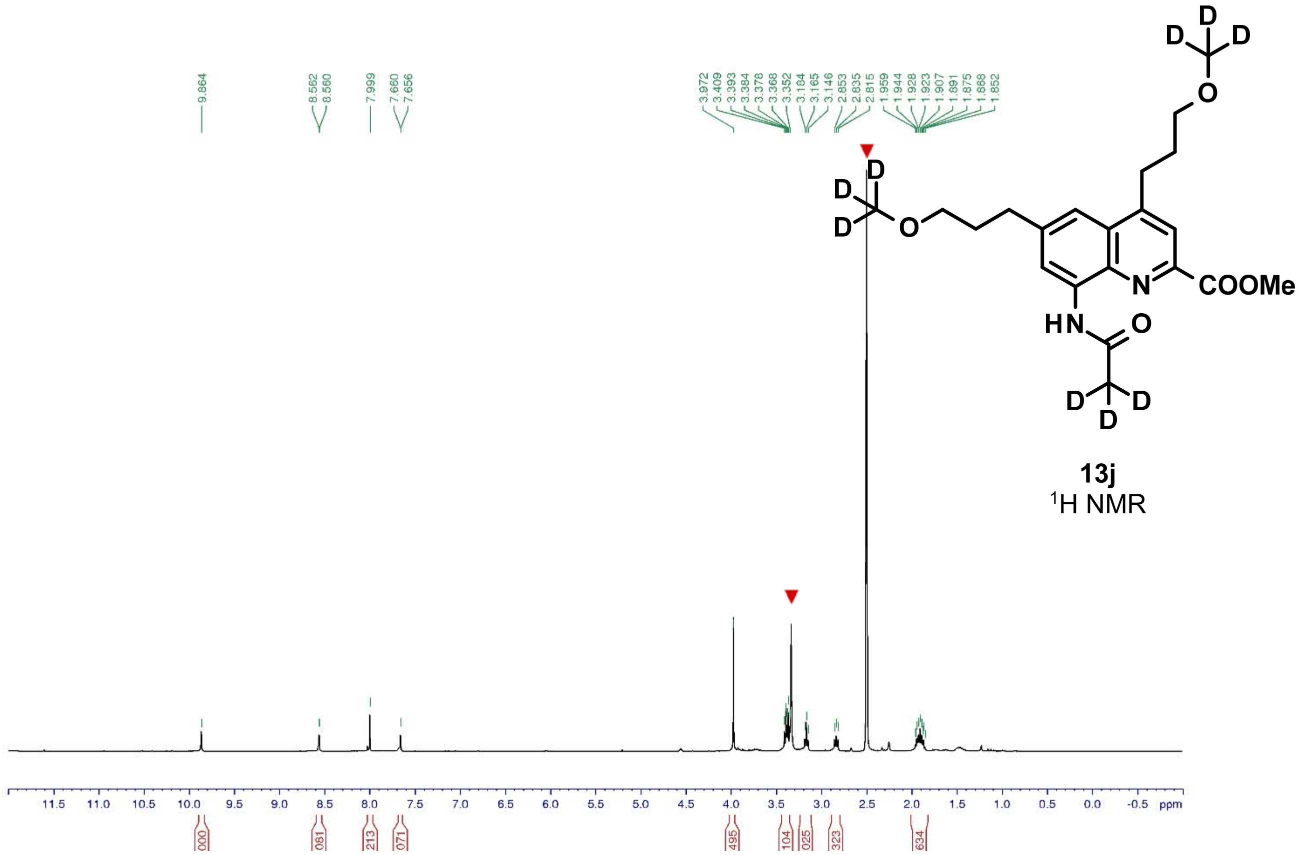

13j
^{1}H NMR

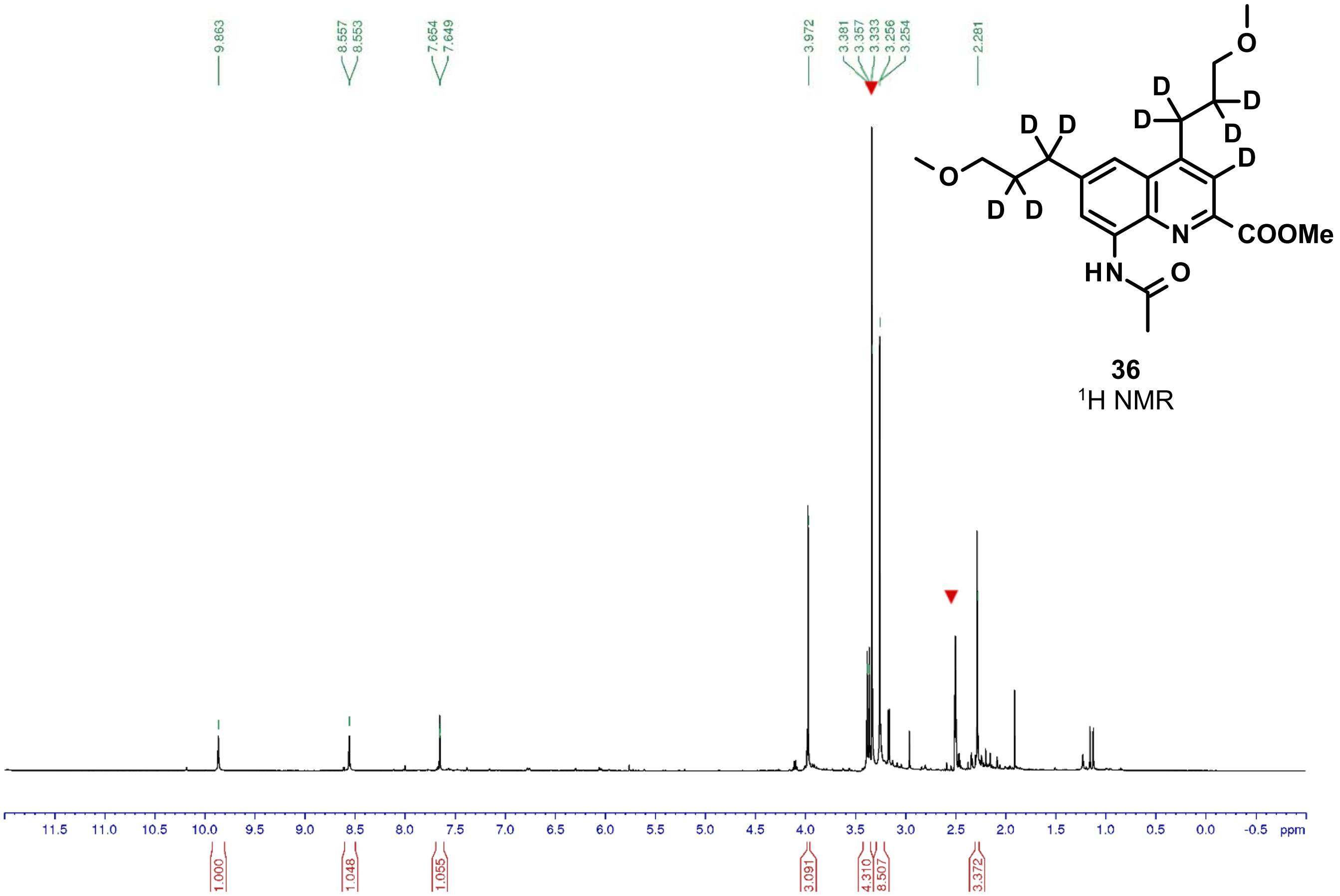

36
^{1}H NMR

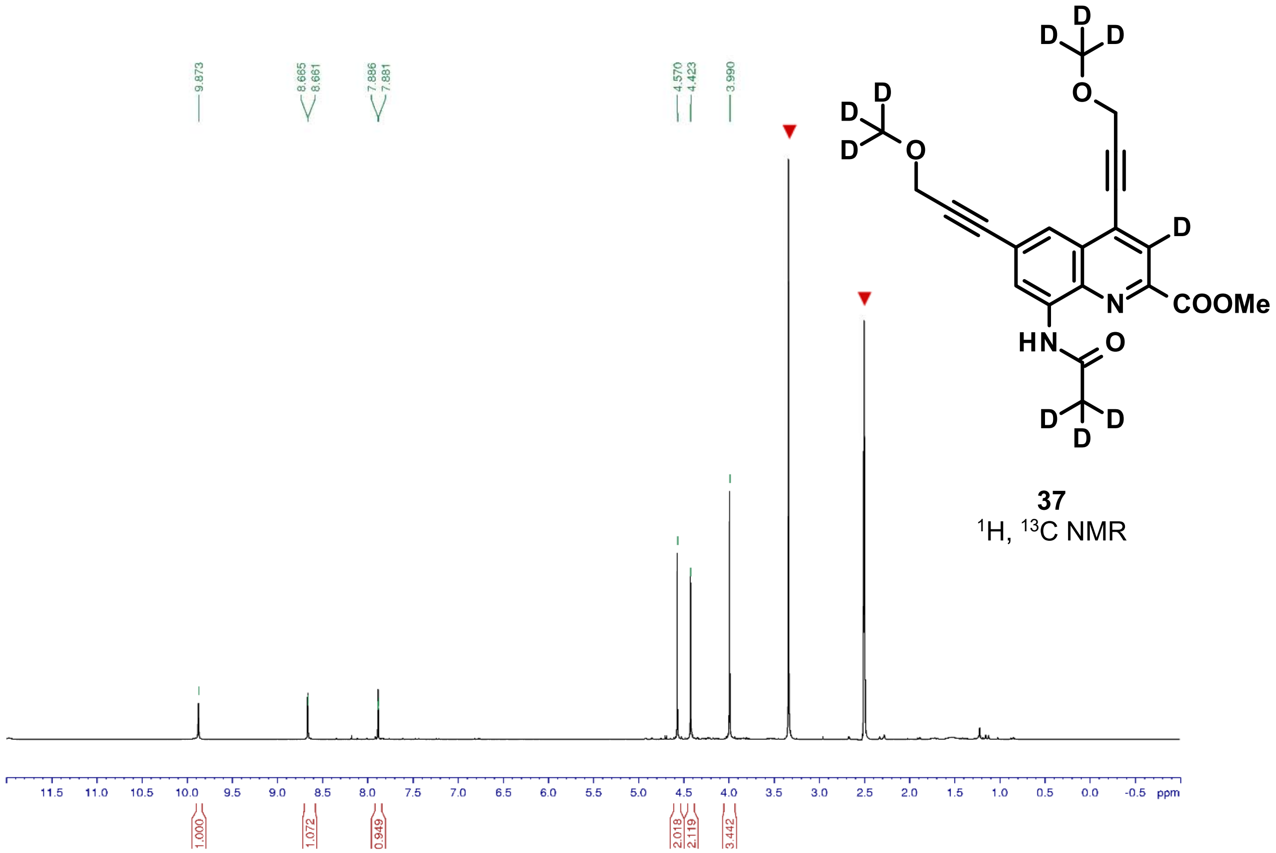
9.873
8.665
8.661
7.886
7.881
4.570
4.423
3.990
11.5 11.0 10.5 10.0 9.5 9.0 8.5 8.0 7.5 7.0 6.5 6.0 5.5 5.0 4.5 4.0 3.5 3.0 2.5 2.0 1.5 1.0 0.5 0.0 -0.5 ppm
1.000
1.072
0.949
2.018
2.119
3.442
D
D
D
O
D
D
D
O
D
N
COOMe
HN
O
D
D
D

37

^{1}H, ^{13}C NMR

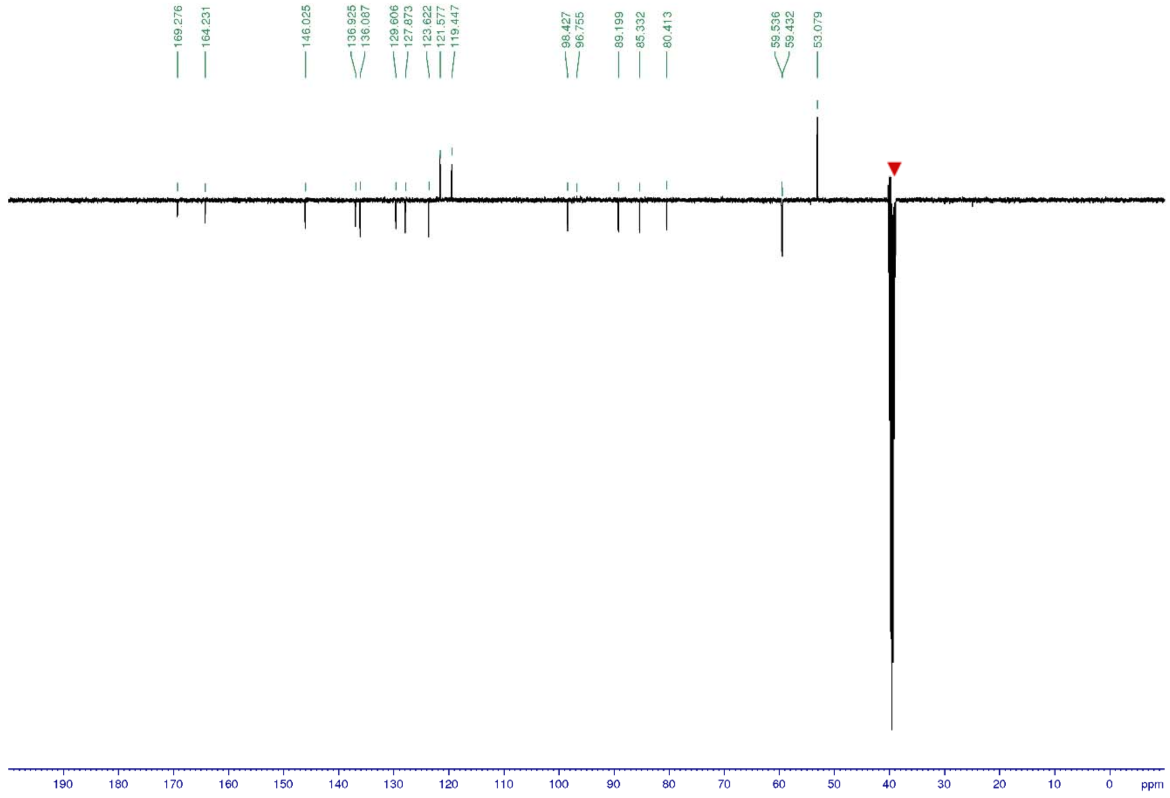
169.276
164.231
146.025
136.925
136.087
129.606
127.873
123.622
121.577
119.447
98.427
96.755
89.199
85.332
80.413
59.536
59.432
53.079
190 180 170 160 150 140 130 120 110 100 90 80 70 60 50 40 30 20 10 0 ppm

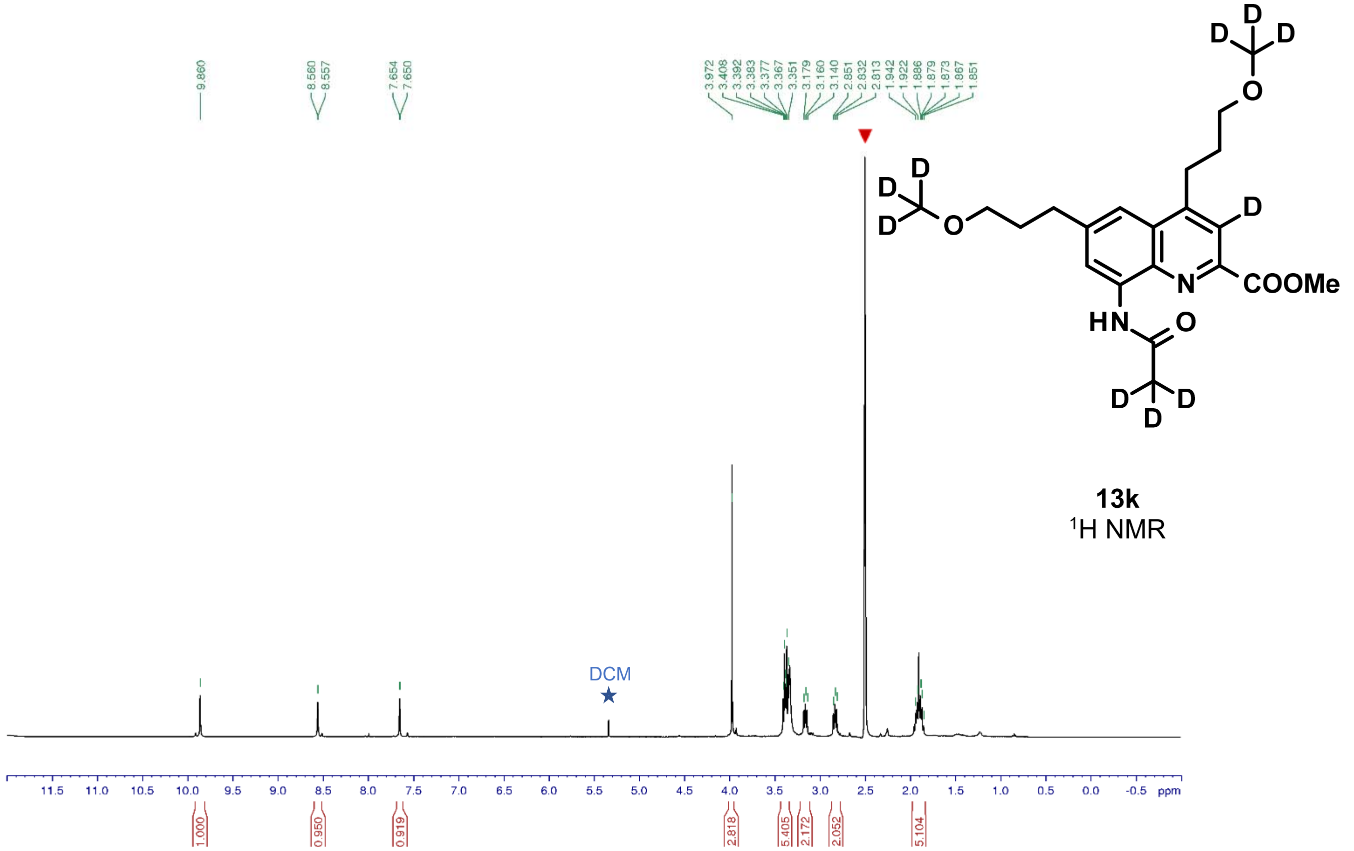

13k
^{1}H NMR

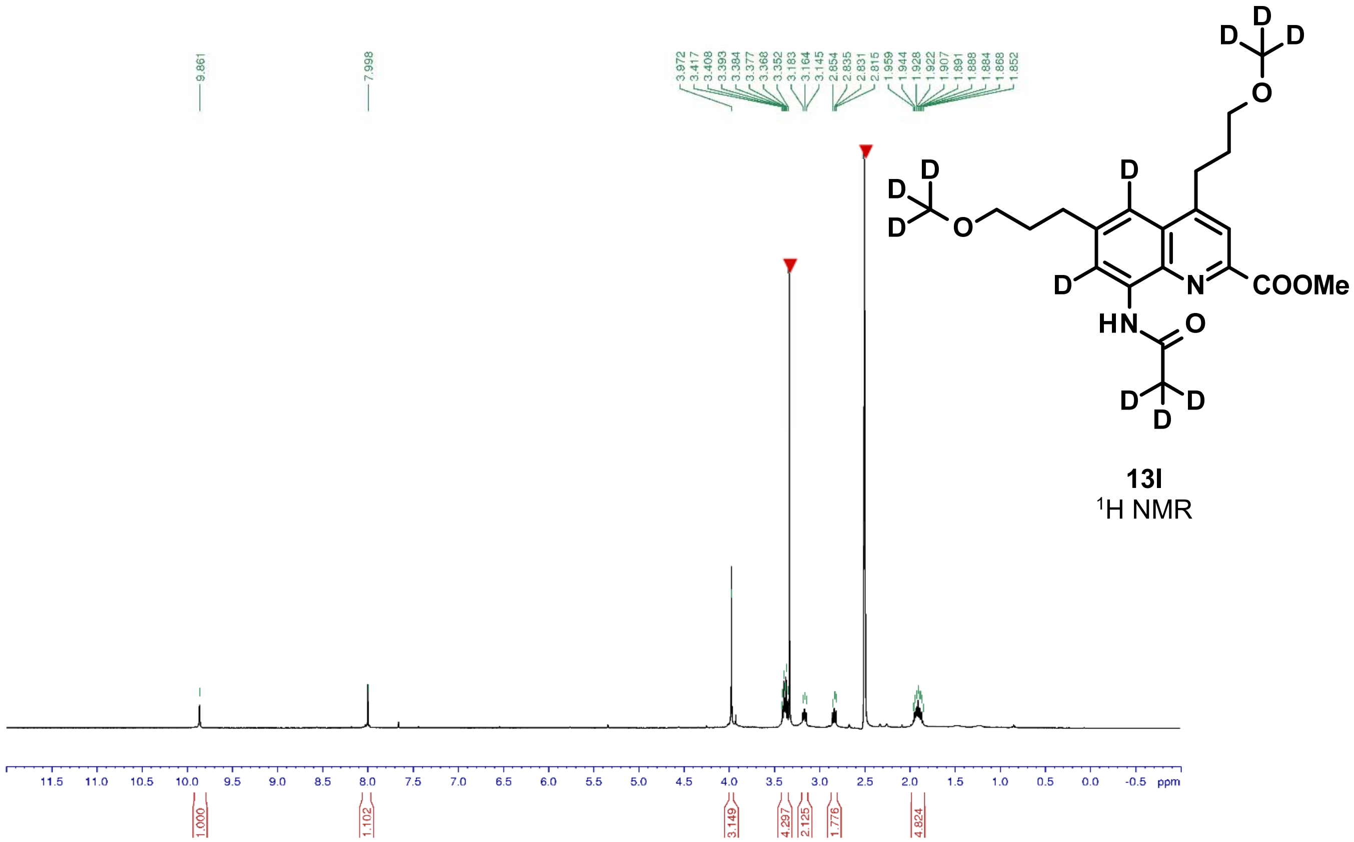

13l
^{1}H NMR

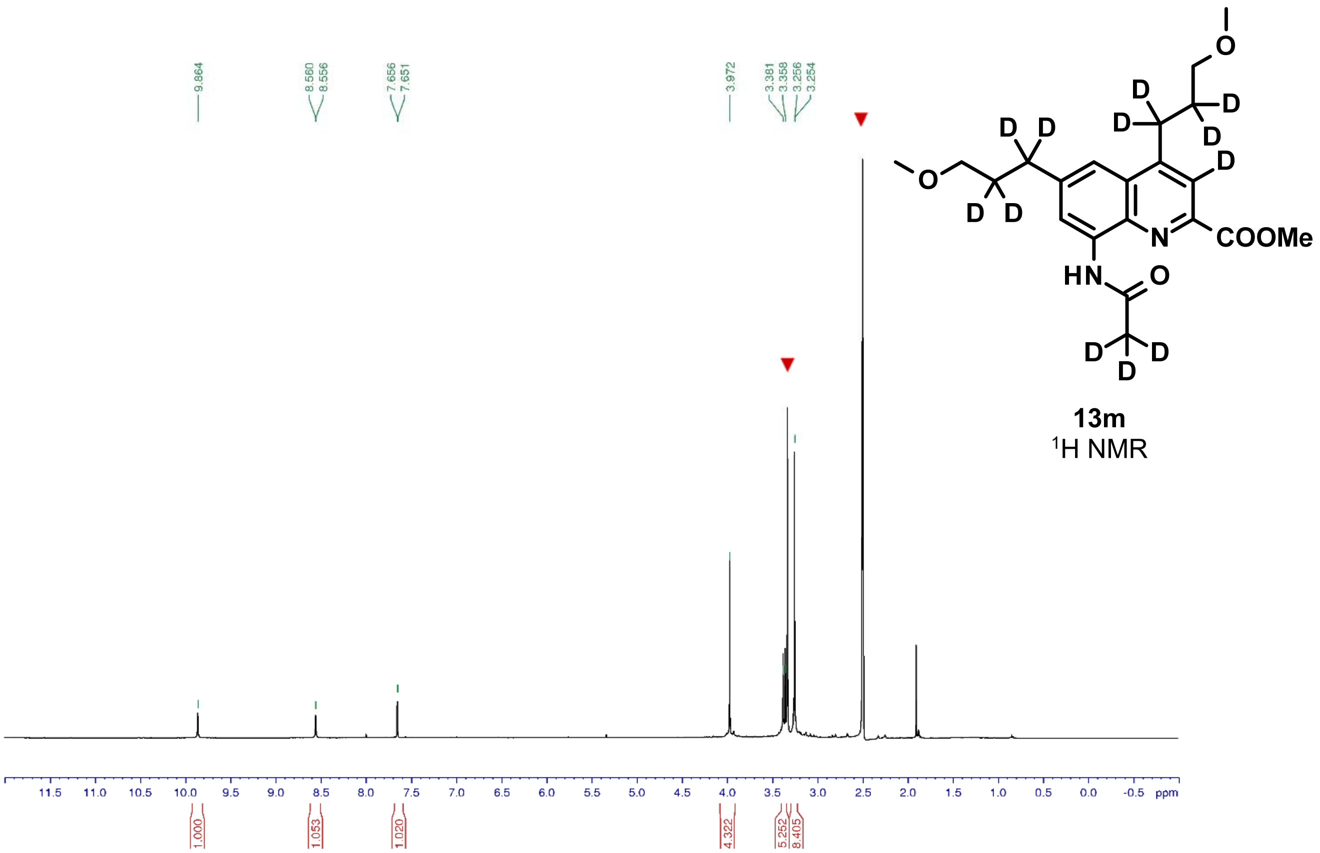

13m
^{1}H NMR

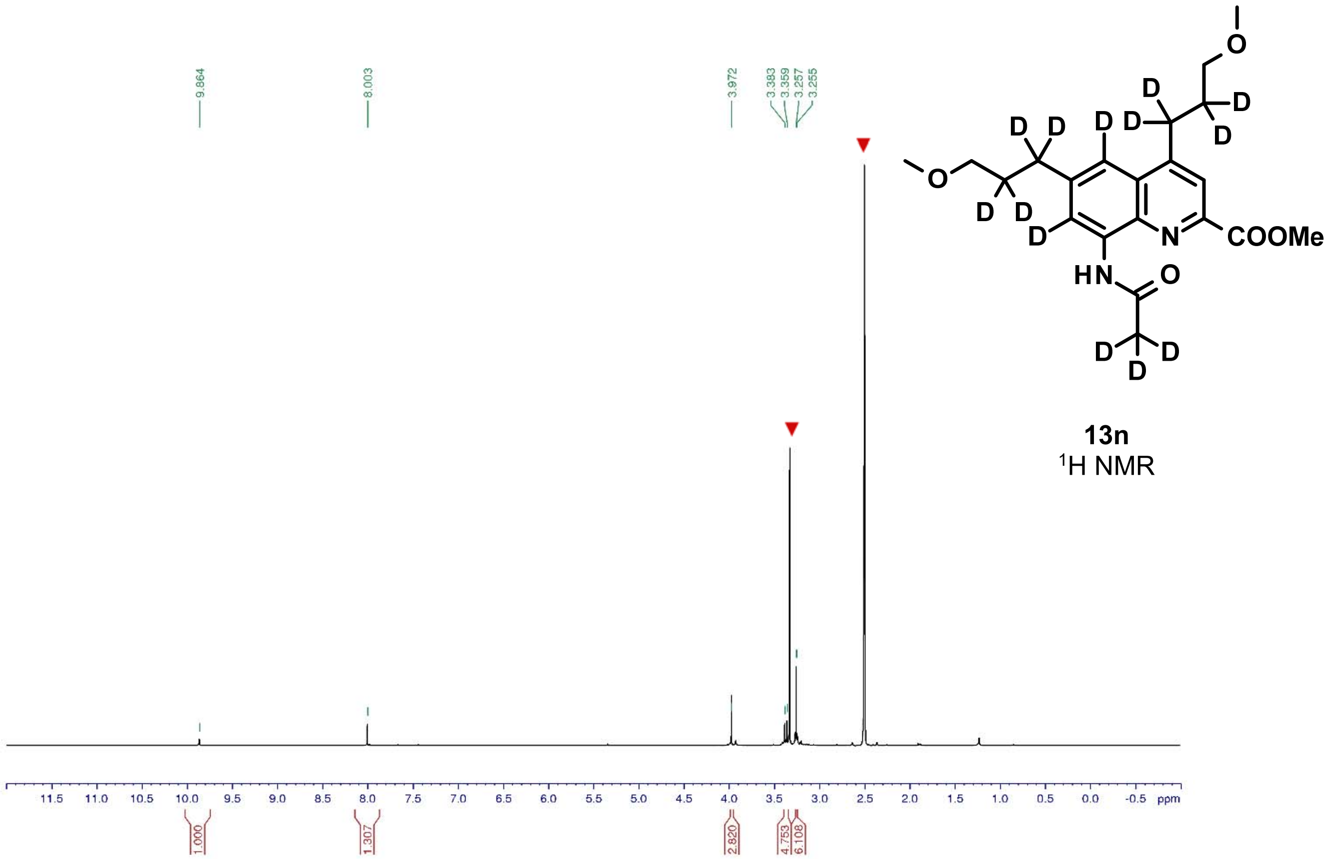

13n
^{1}H NMR

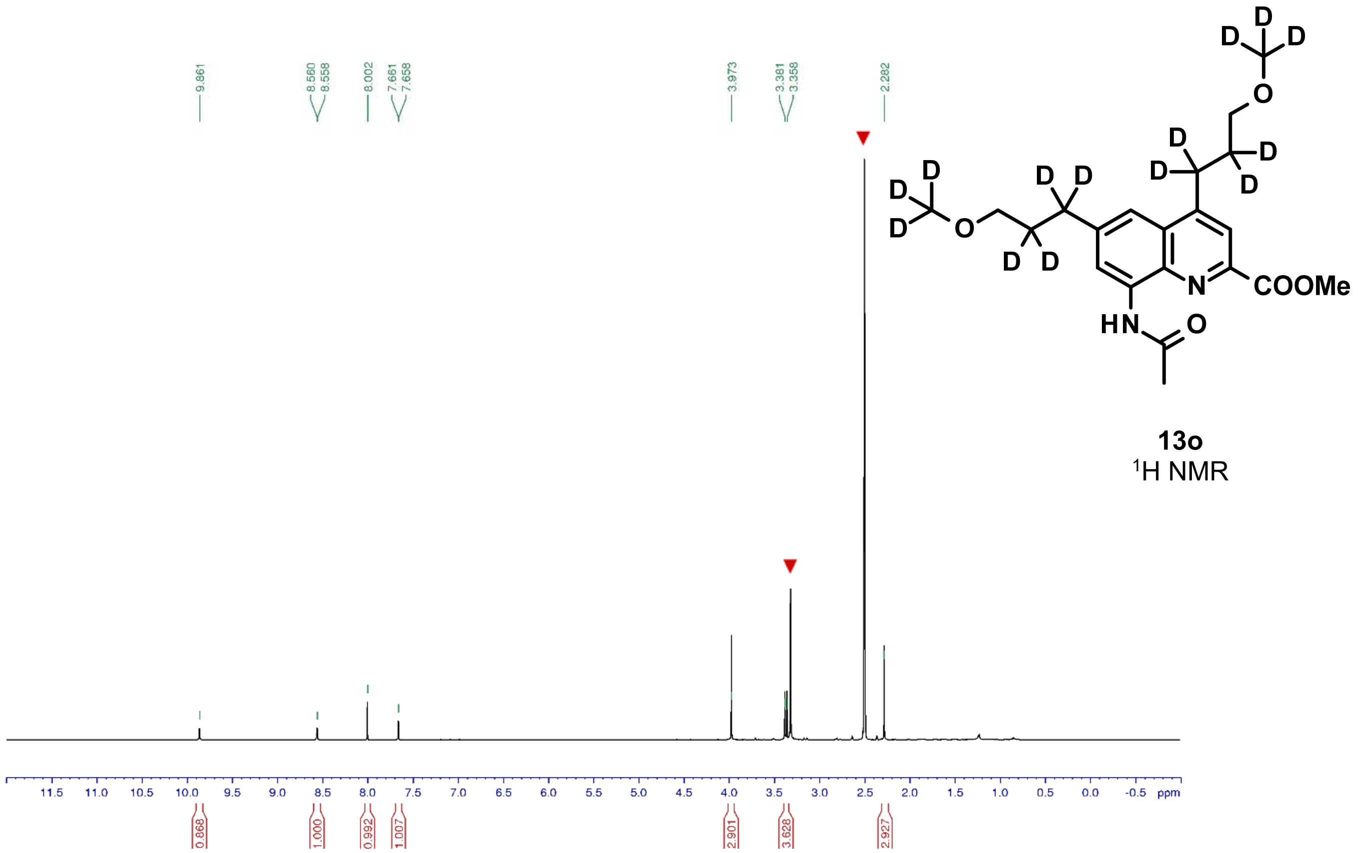

13o
^{1}H NMR

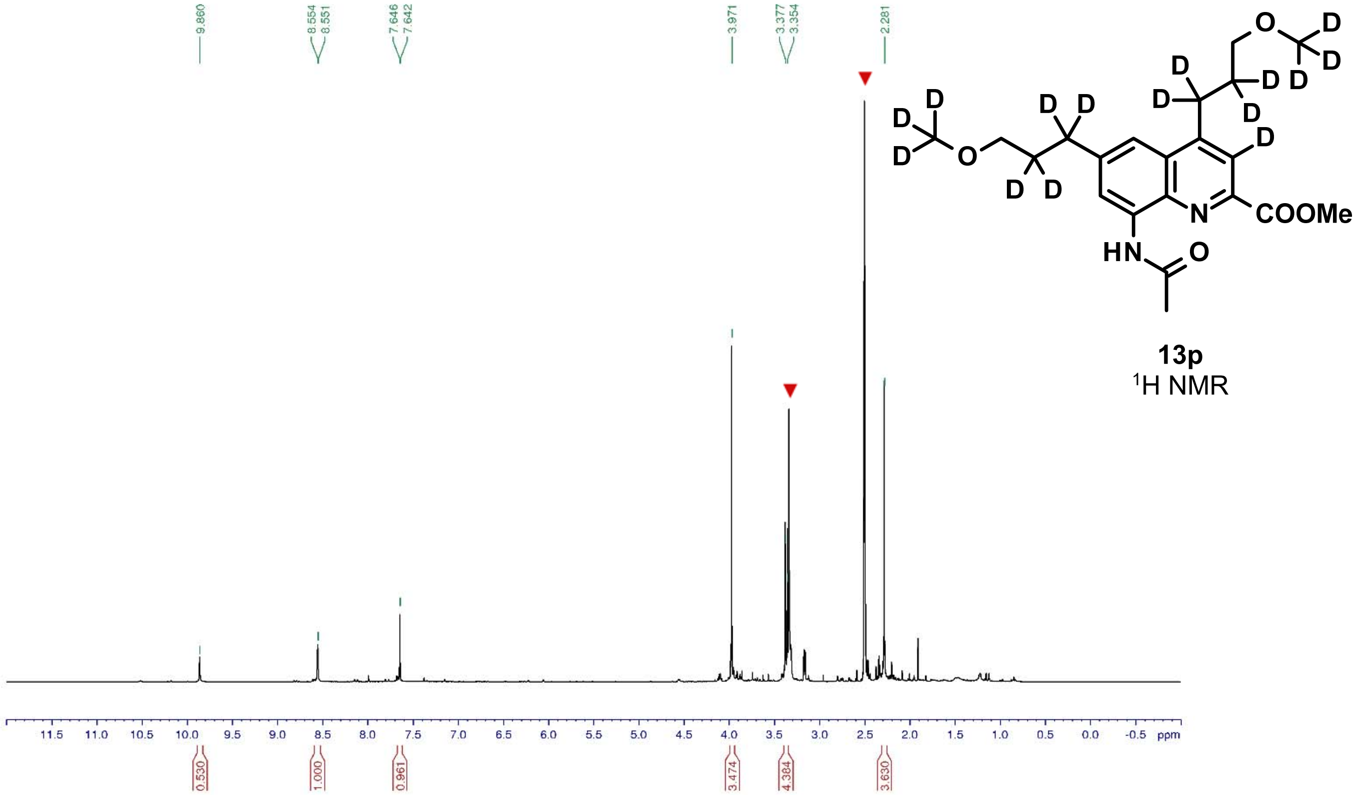

13p
^{1}H NMR

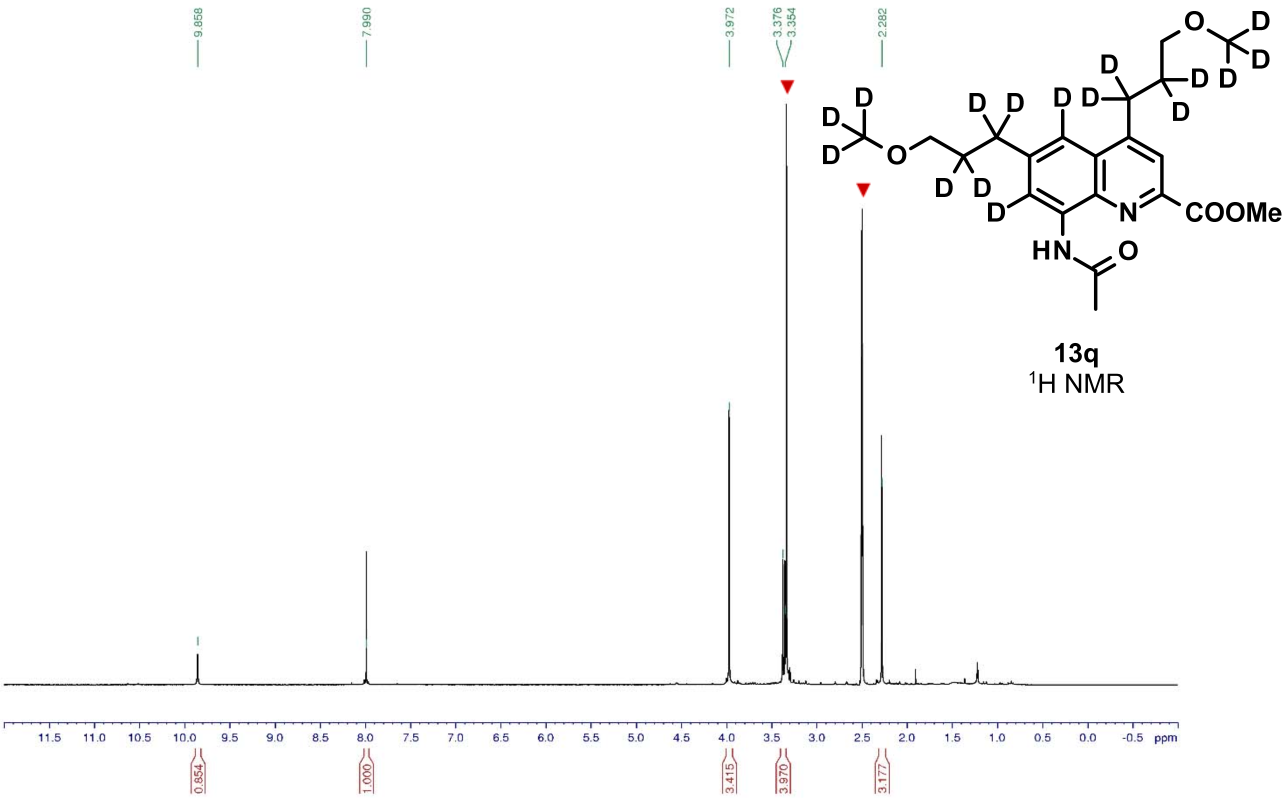

13q
^{1}H NMR

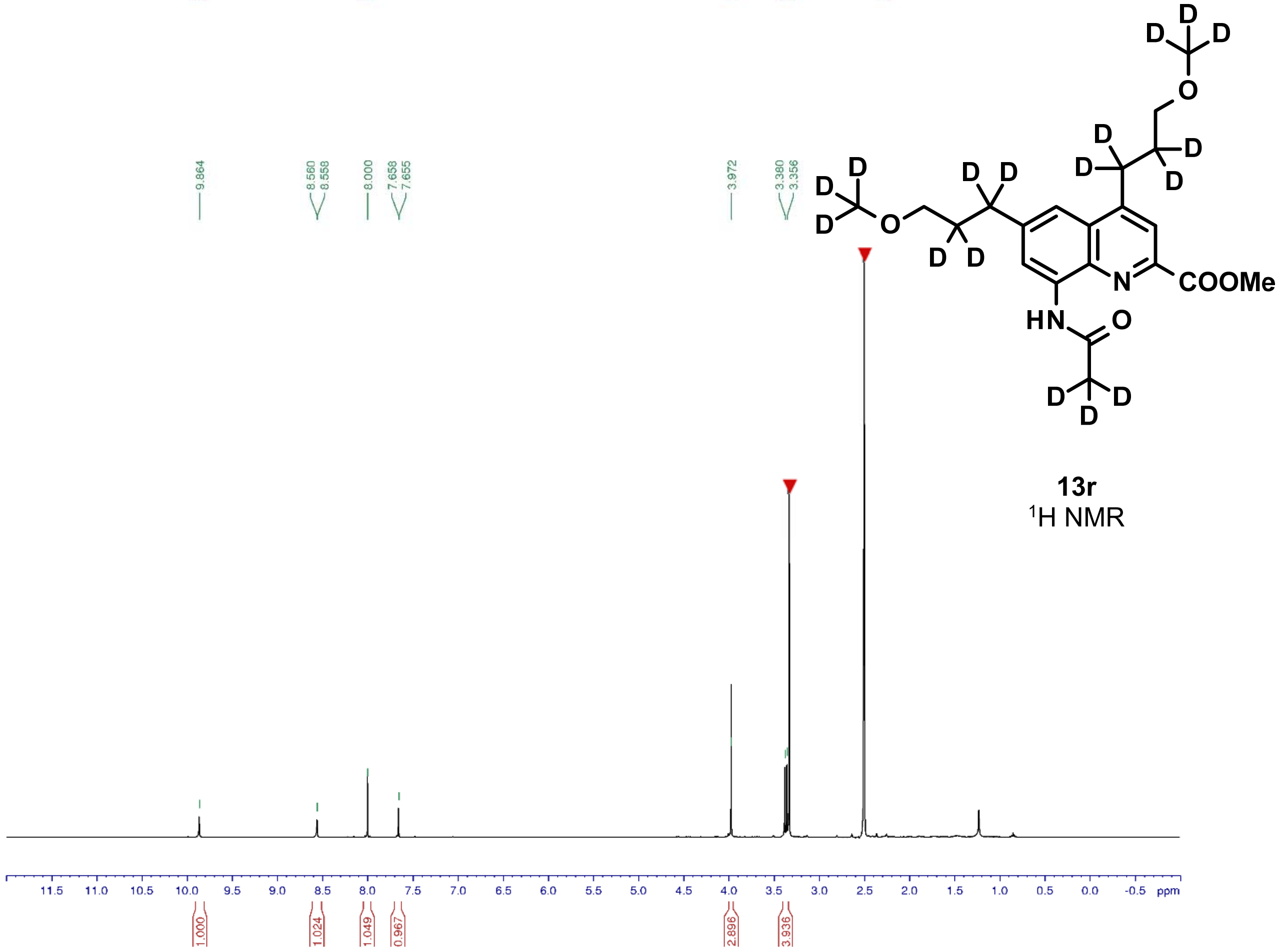

13r
^{1}H NMR

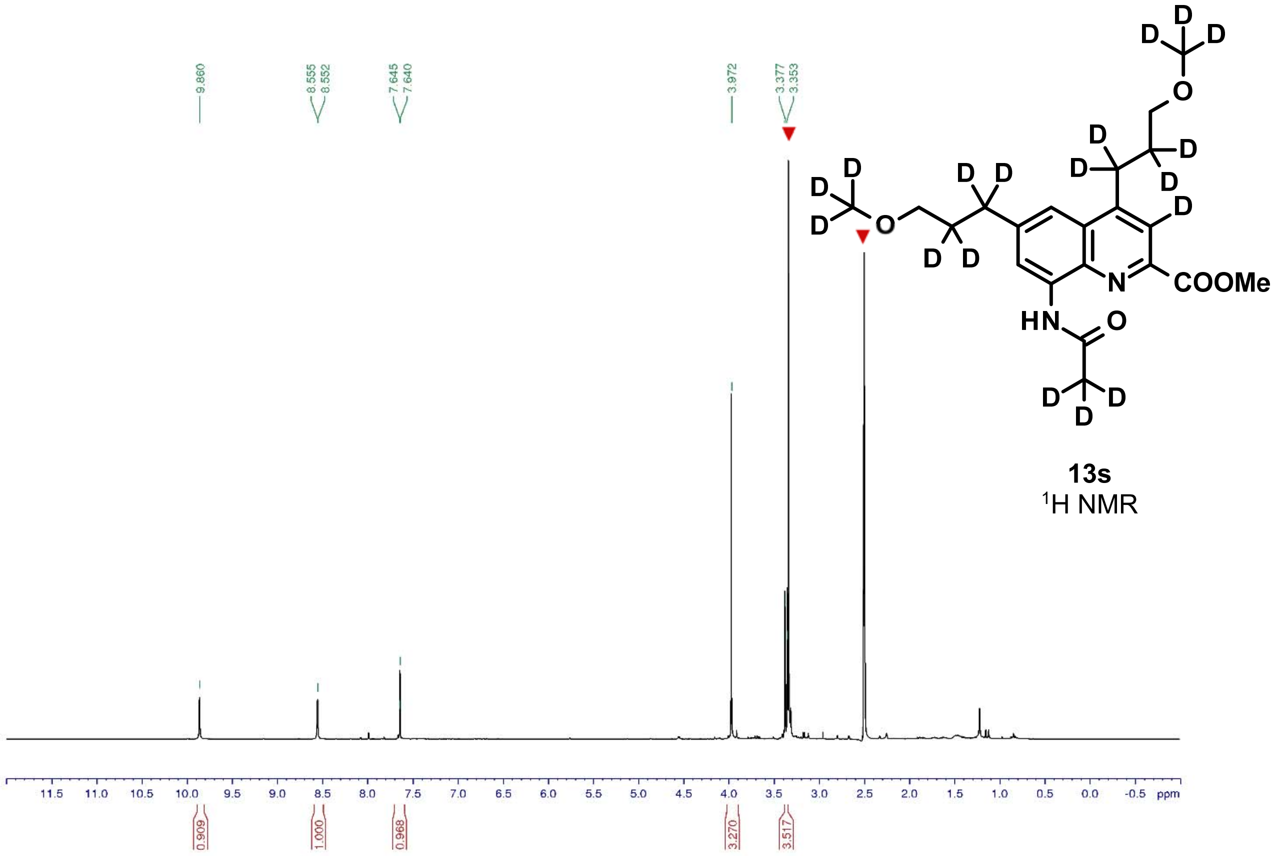

13s
^{1}H NMR

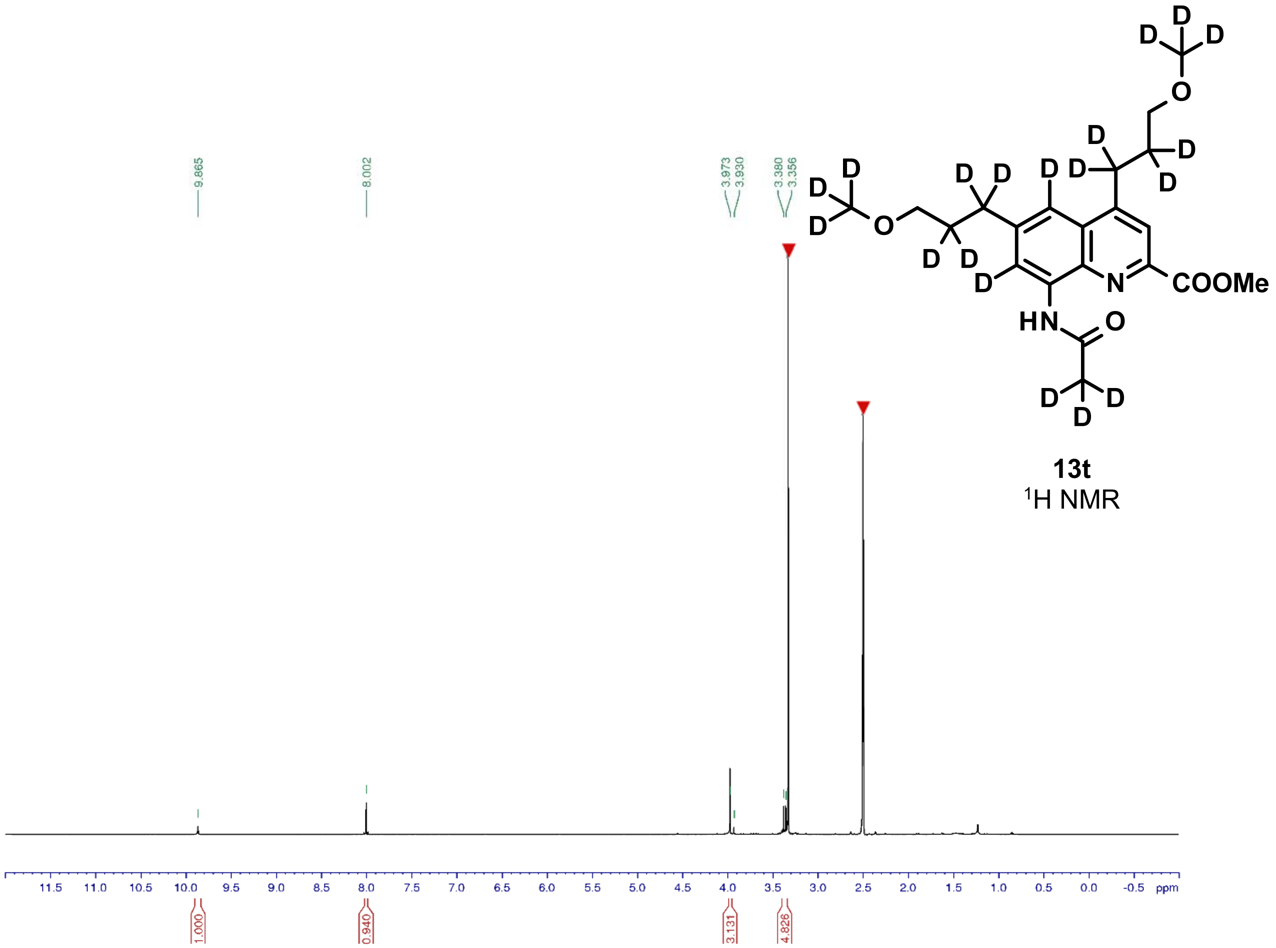

13t
^{1}H NMR

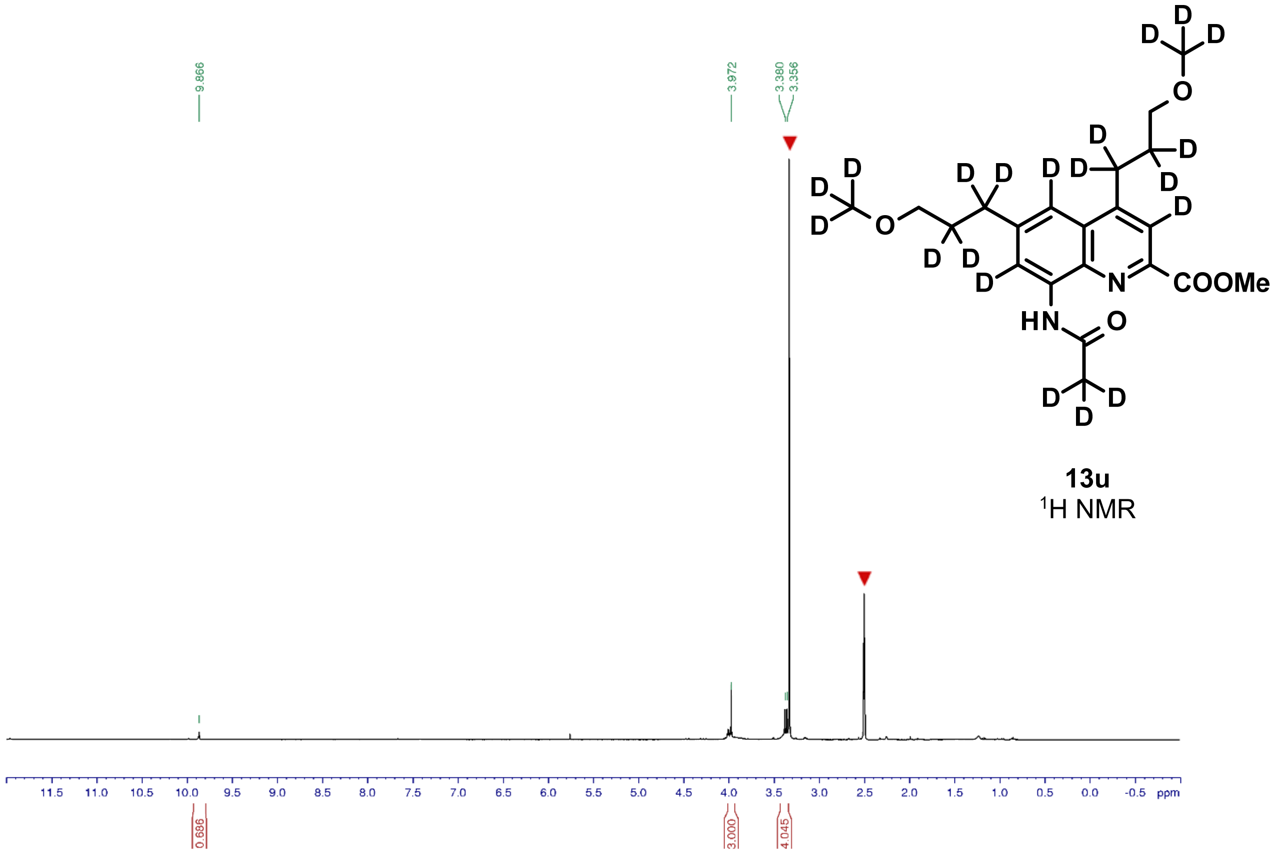

13u
^{1}H NMR

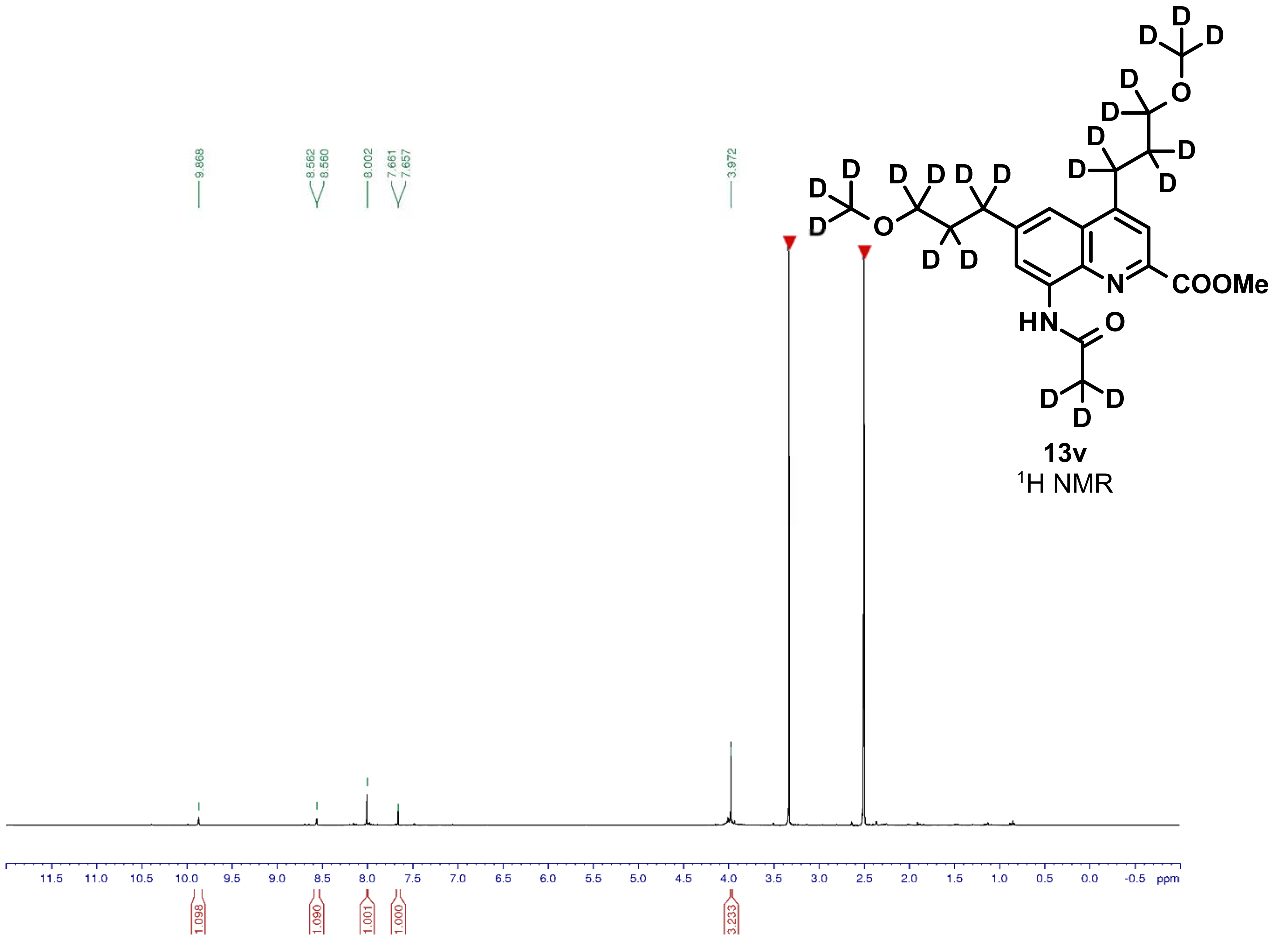

13v
^{1}H NMR

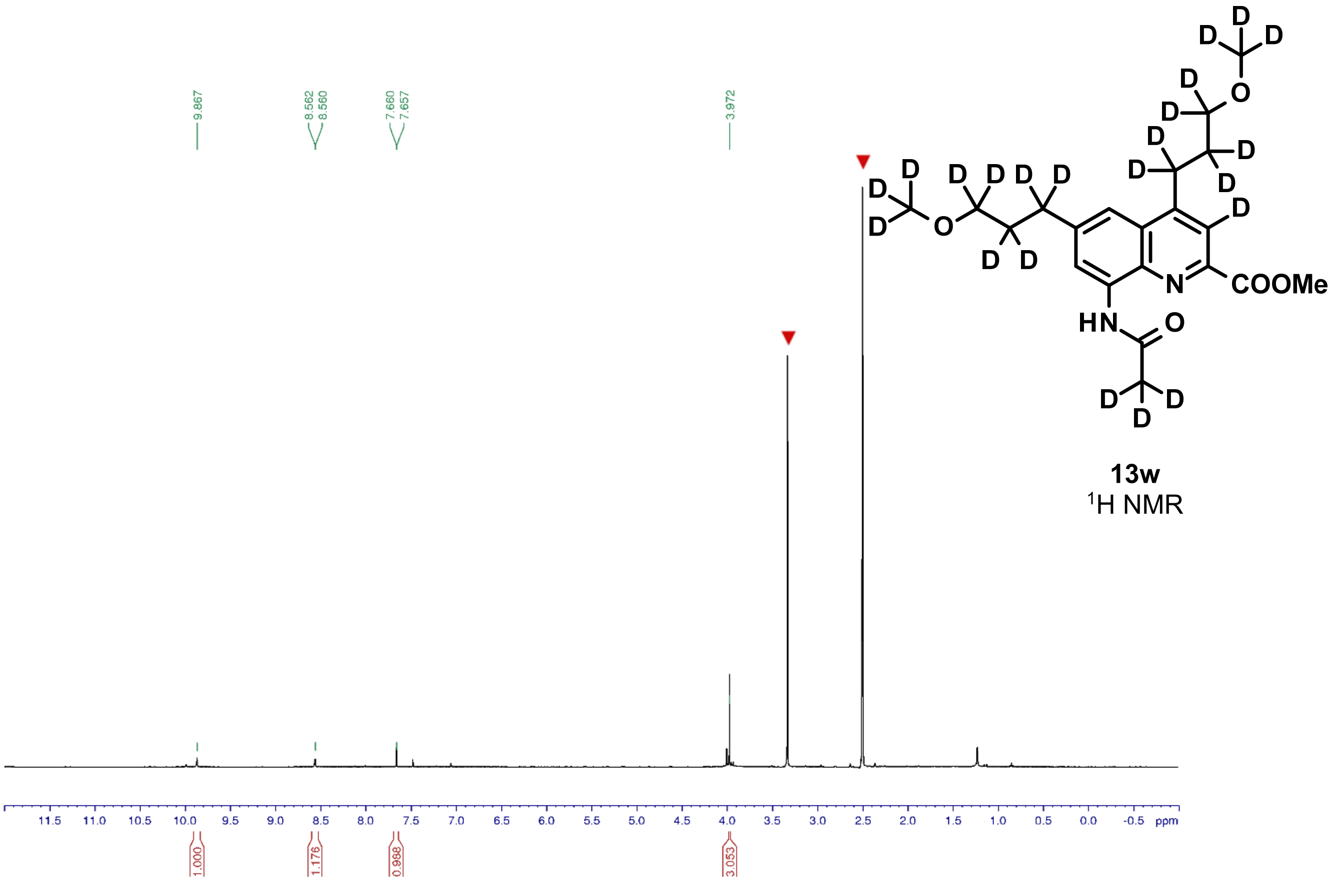

13w
^{1}H NMR

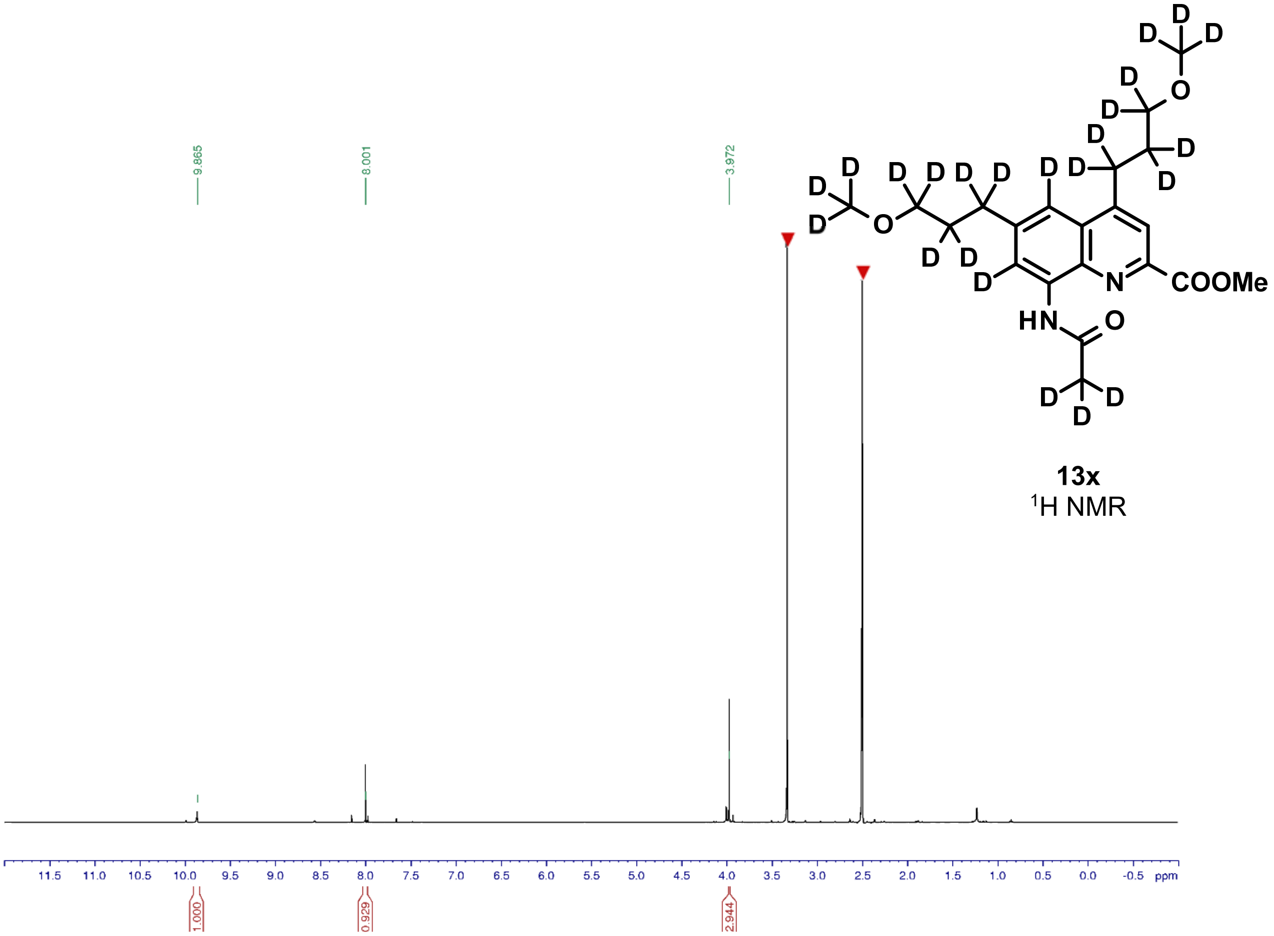

13x
^{1}H NMR

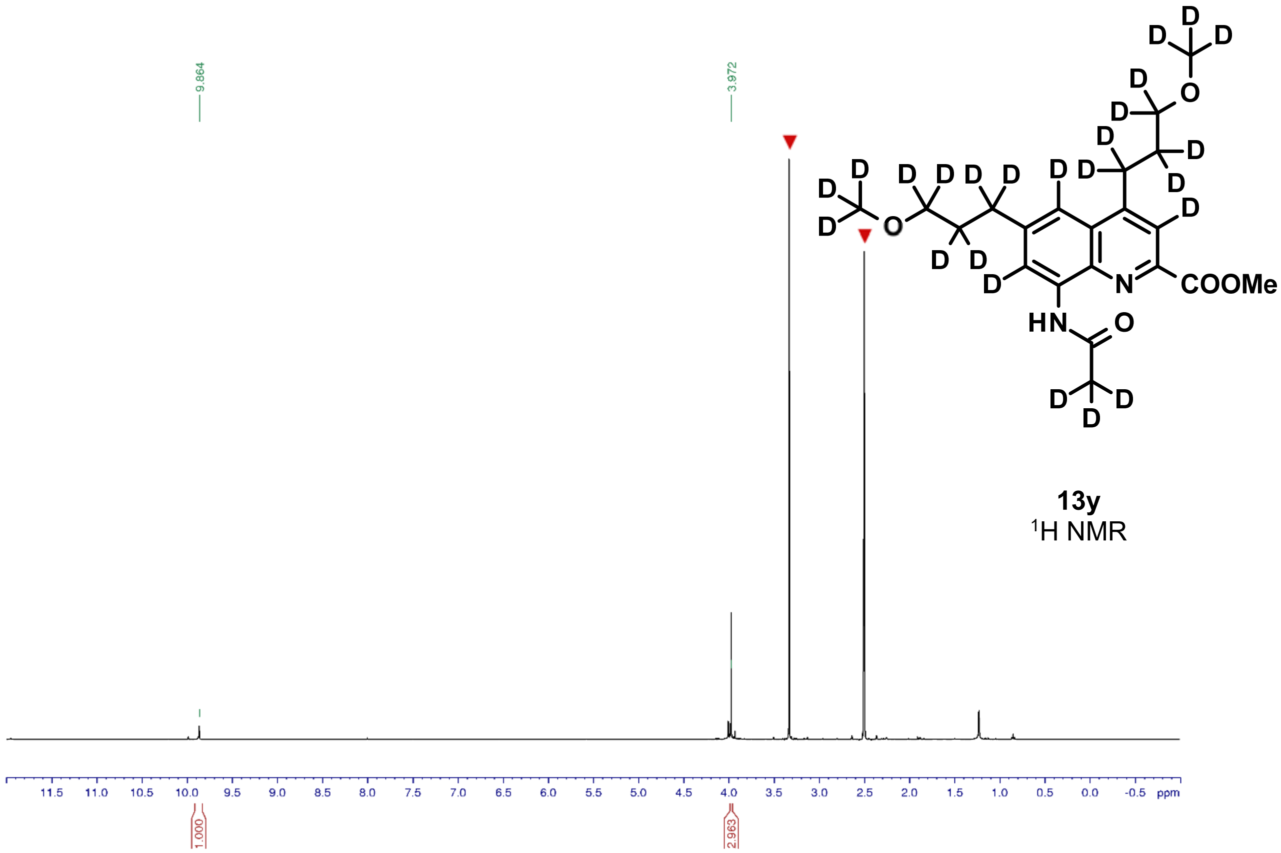

9.864
3.972
11.5 11.0 10.5 10.0 9.5 9.0 8.5 8.0 7.5 7.0 6.5 6.0 5.5 5.0 4.5 4.0 3.5 3.0 2.5 2.0 1.5 1.0 0.5 0.0 -0.5 ppm
1.000
2.963
COOMe
HN

13y
^{1}H NMR

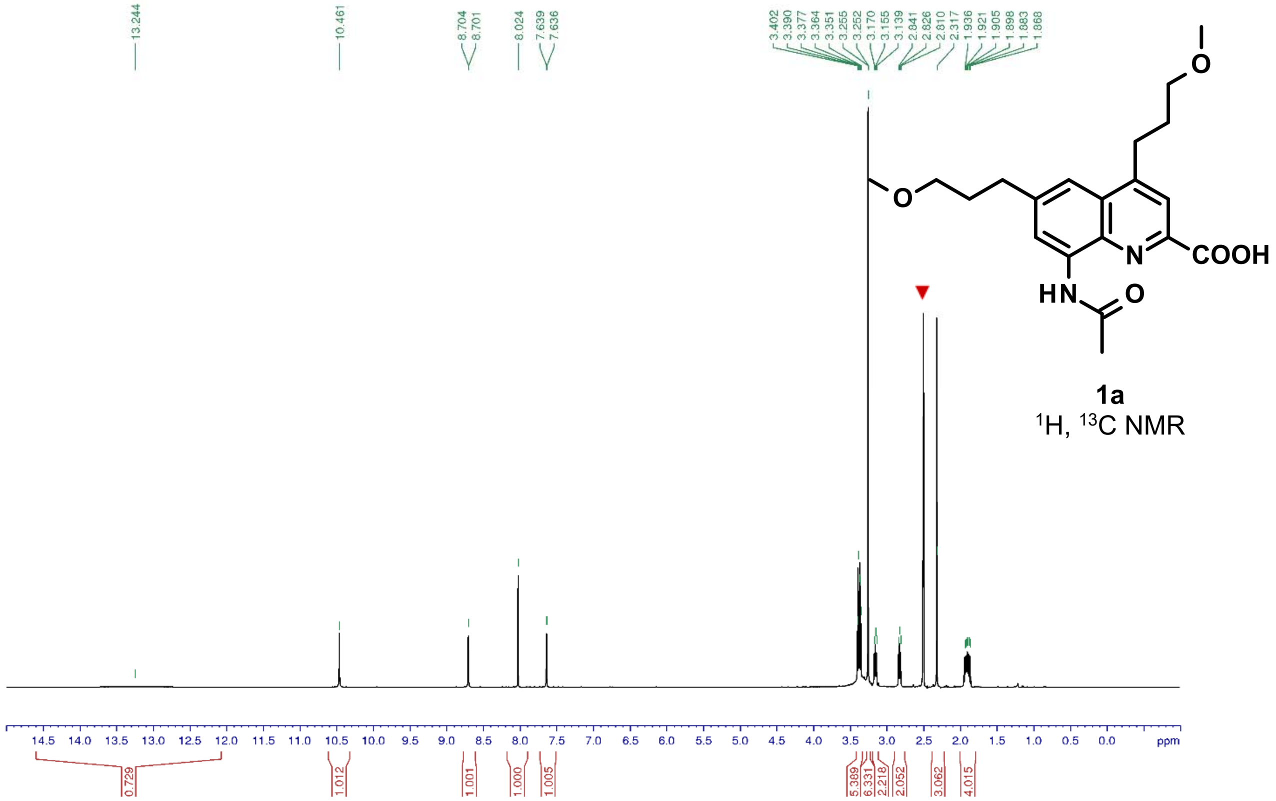

1a

^{1}H, ^{13}C NMR

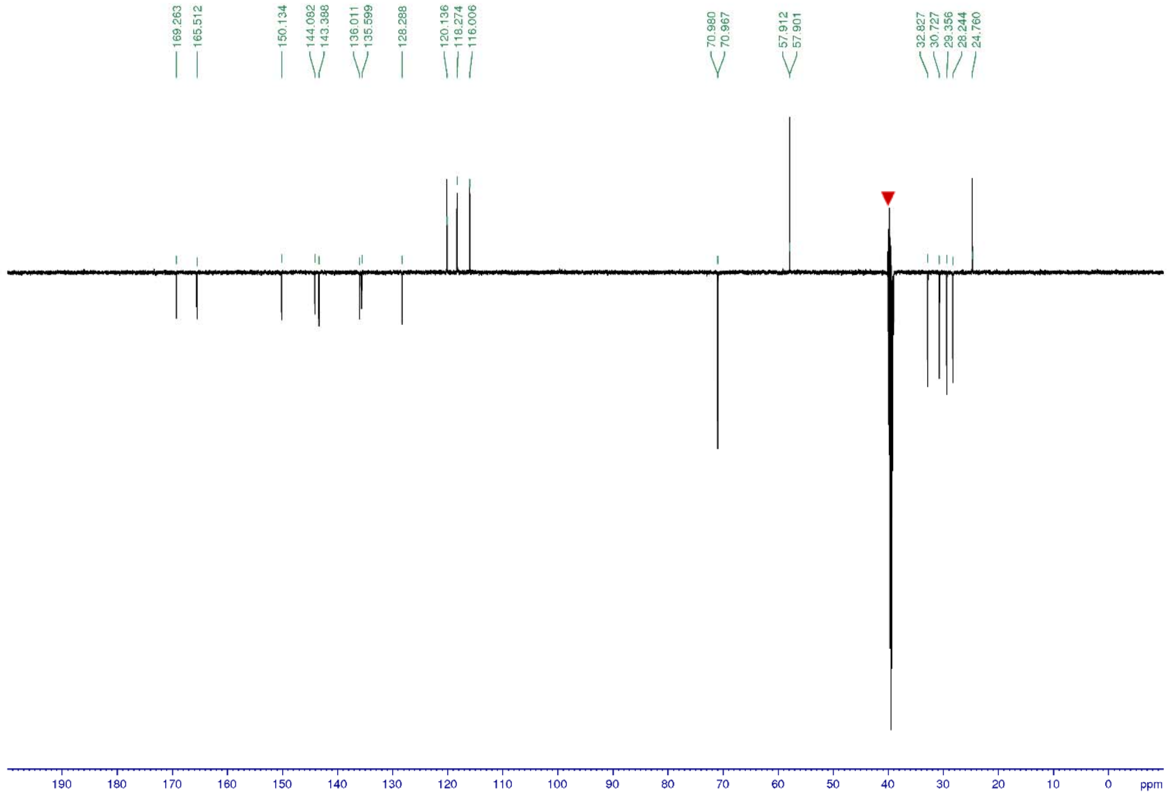

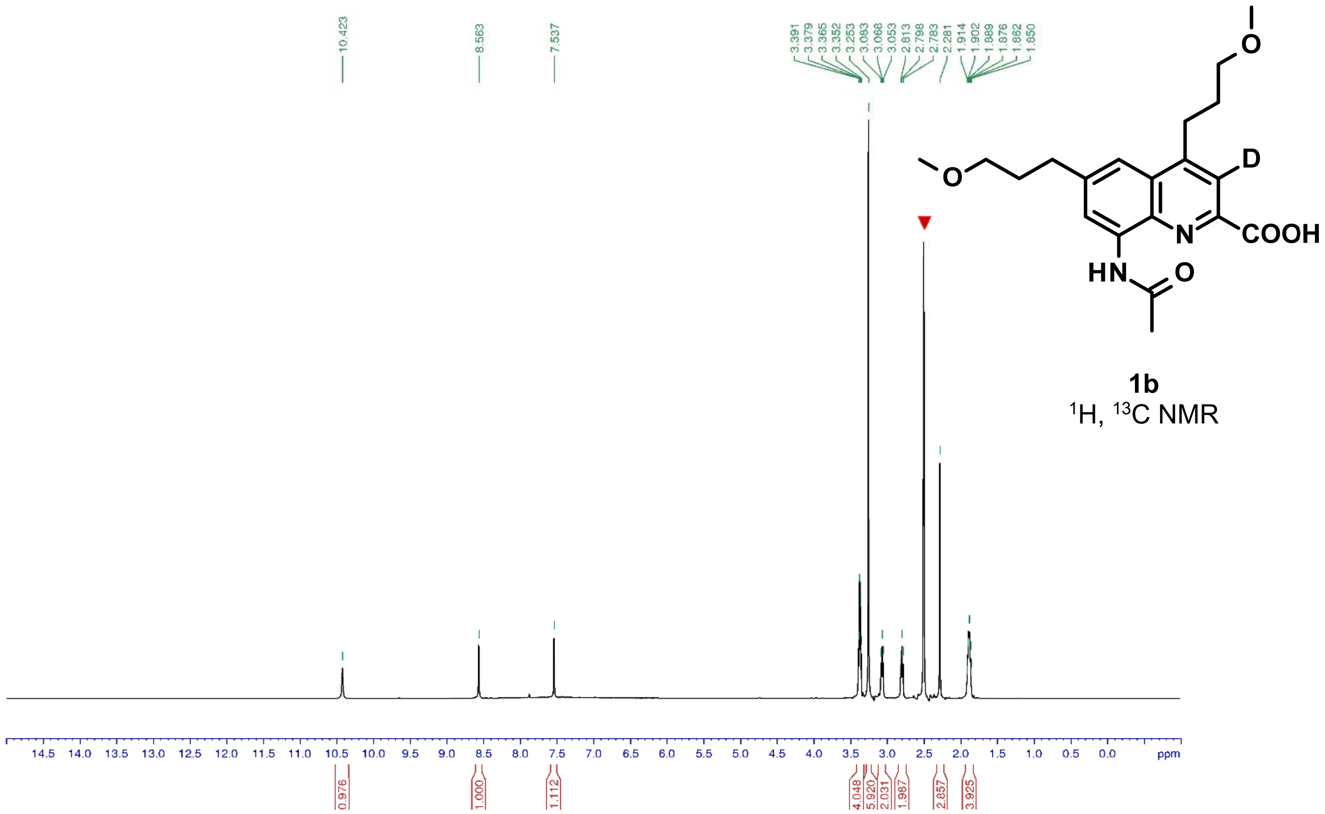

D
N
COOH
HN
O
O
O
10.423
8.563
7.537
3.391 3.379 3.365 3.352 3.253 3.083 3.068 3.053 2.813 2.798 2.783 2.281 1.914 1.902 1.889 1.876 1.862 1.850
14.5 14.0 13.5 13.0 12.5 12.0 11.5 11.0 10.5 10.0 9.5 9.0 8.5 8.0 7.5 7.0 6.5 6.0 5.5 5.0 4.5 4.0 3.5 3.0 2.5 2.0 1.5 1.0 0.5 0.0 ppm
0.976
1.000
1.112
4.048 5.920 2.031 1.987 2.857 3.925

1b
^{1}H, ^{13}C NMR

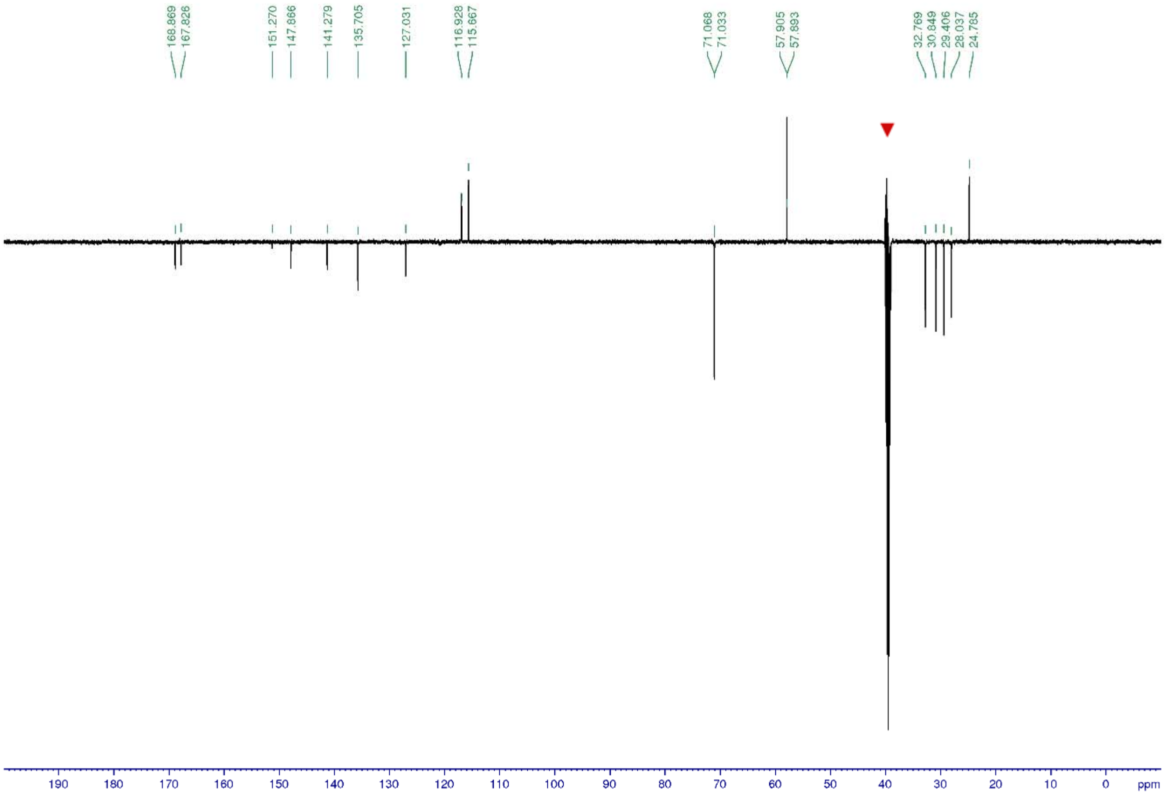

168.869 167.826
151.270 147.866 141.279 135.705 127.031 116.928 115.667
71.068 71.033
57.905 57.893
32.769 30.849 29.406 28.037 24.765
190 180 170 160 150 140 130 120 110 100 90 80 70 60 50 40 30 20 10 0 ppm

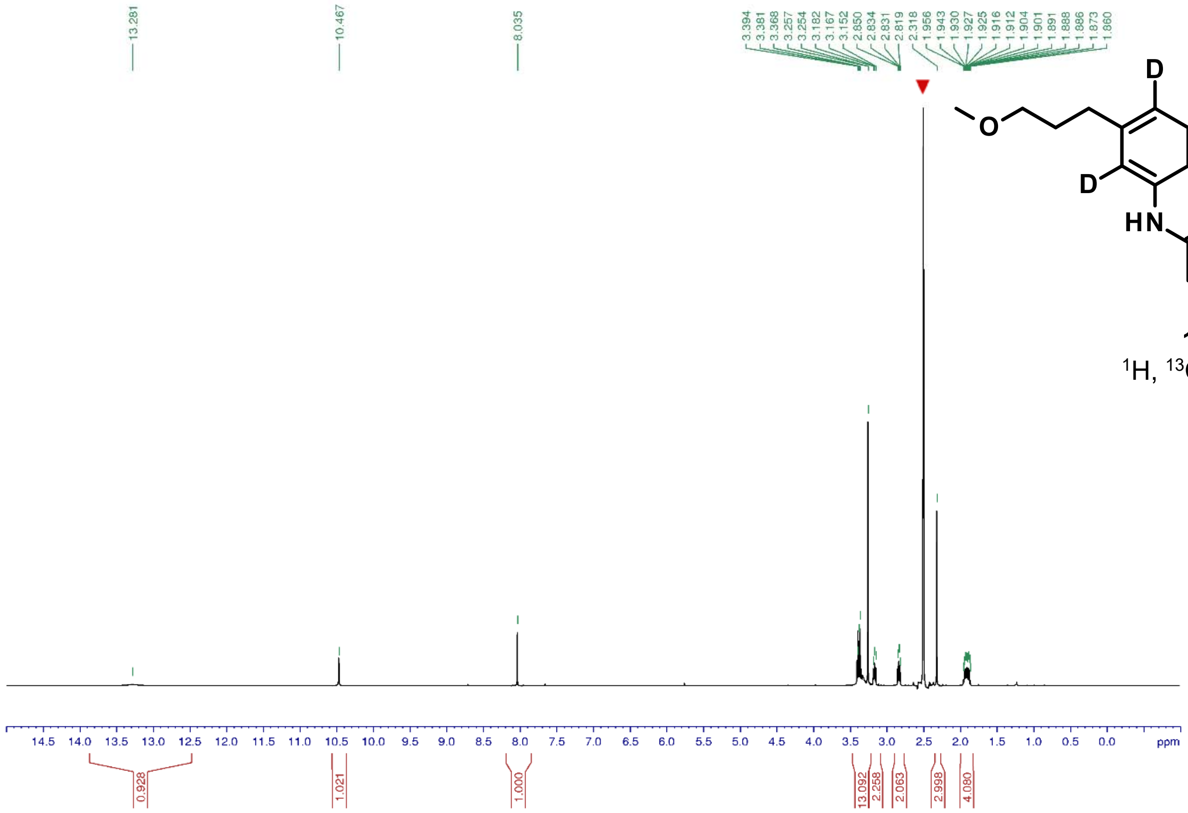
13.281
10.467
8.035
3.394 3.381 3.368 3.257 3.254 3.182 3.167 3.152 2.850 2.834 2.831 2.819 2.318 1.956 1.943 1.930 1.927 1.925 1.916 1.912 1.904 1.901 1.891 1.888 1.886 1.873 1.860
0.928
1.021
1.000
13.092 2.258 2.063 2.998 4.080
14.5 14.0 13.5 13.0 12.5 12.0 11.5 11.0 10.5 10.0 9.5 9.0 8.5 8.0 7.5 7.0 6.5 6.0 5.5 5.0 4.5 4.0 3.5 3.0 2.5 2.0 1.5 1.0 0.5 0.0 ppm

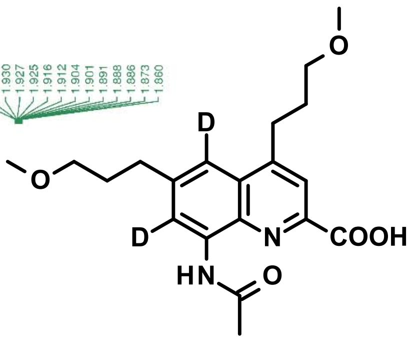
D
O
O
D
N
COOH
HN
O

1c

^{1}H, ^{13}C NMR

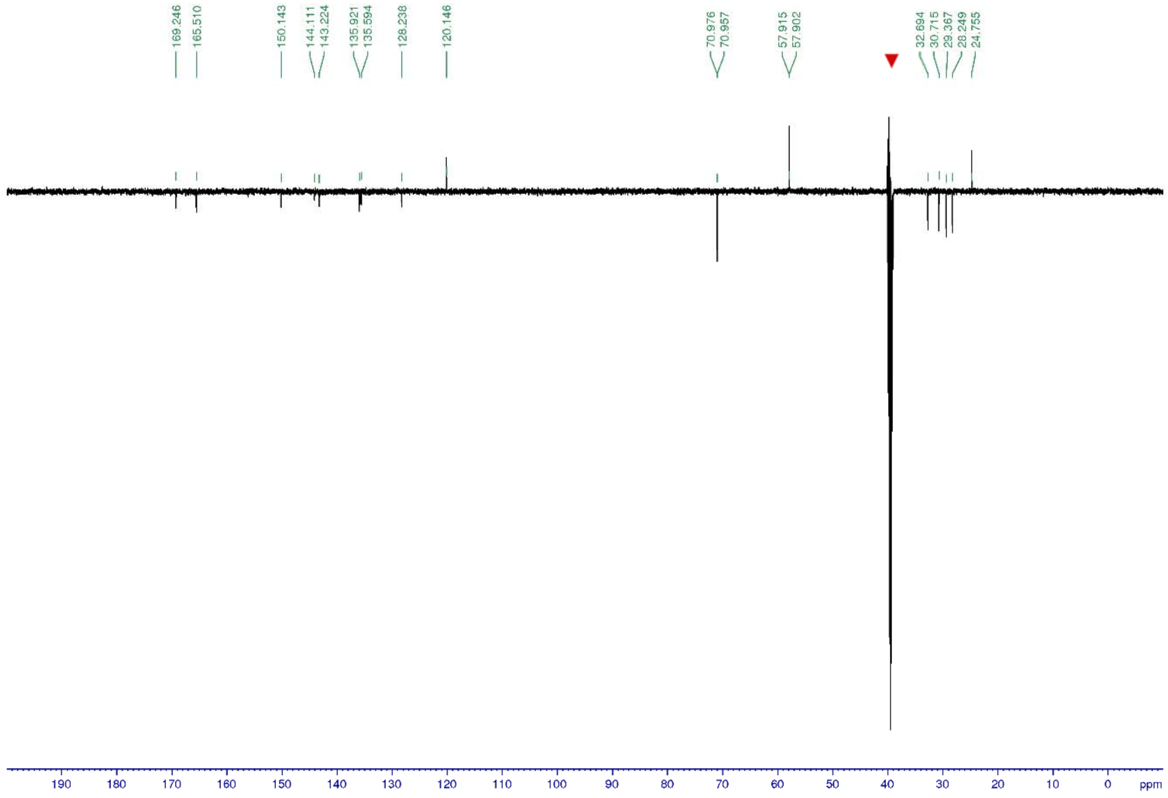
169.246
165.510
150.143
144.111
143.224
135.921
135.594
128.238
120.146
70.976
70.957
57.915
57.902
32.694
30.715
29.367
28.249
24.755
190 180 170 160 150 140 130 120 110 100 90 80 70 60 50 40 30 20 10 0 ppm

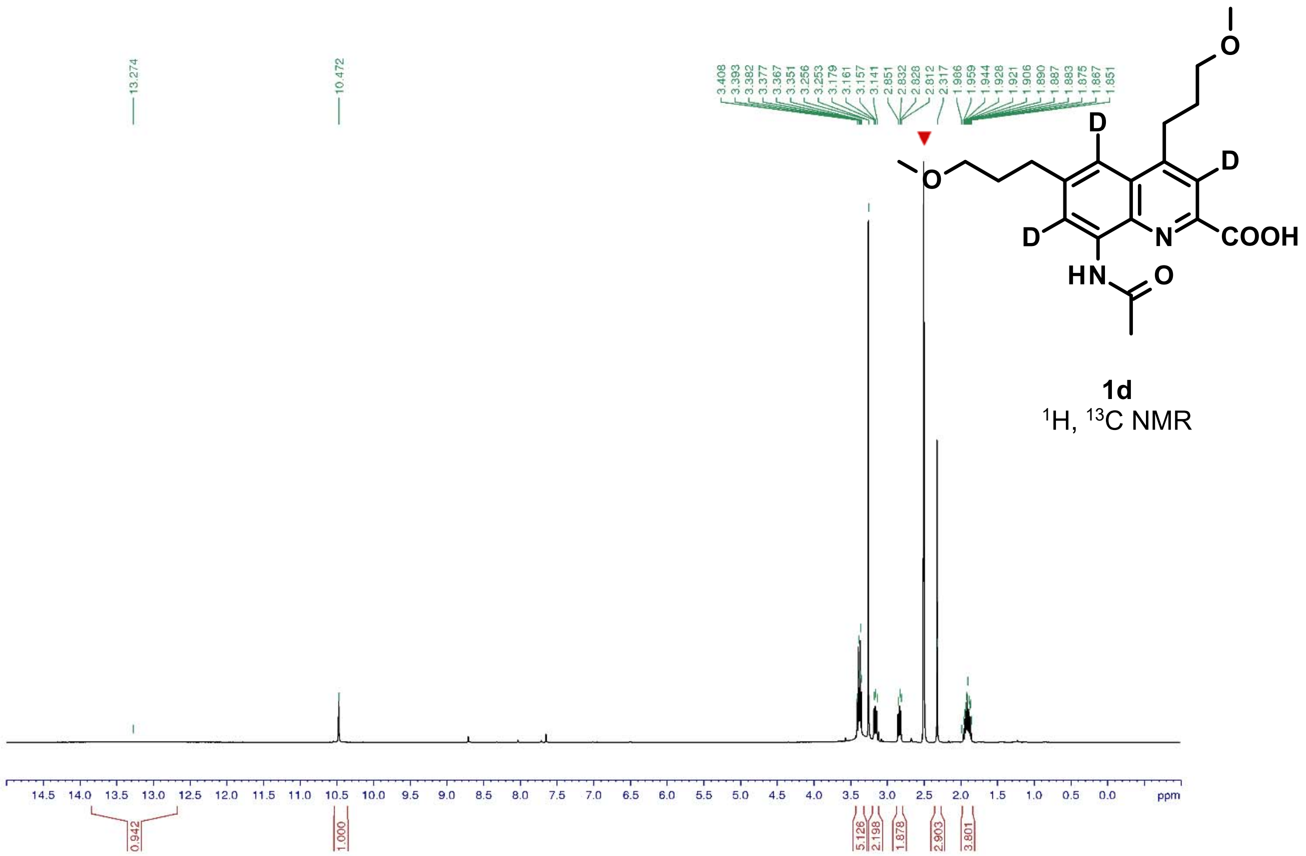

1d

^{1}H, ^{13}C NMR

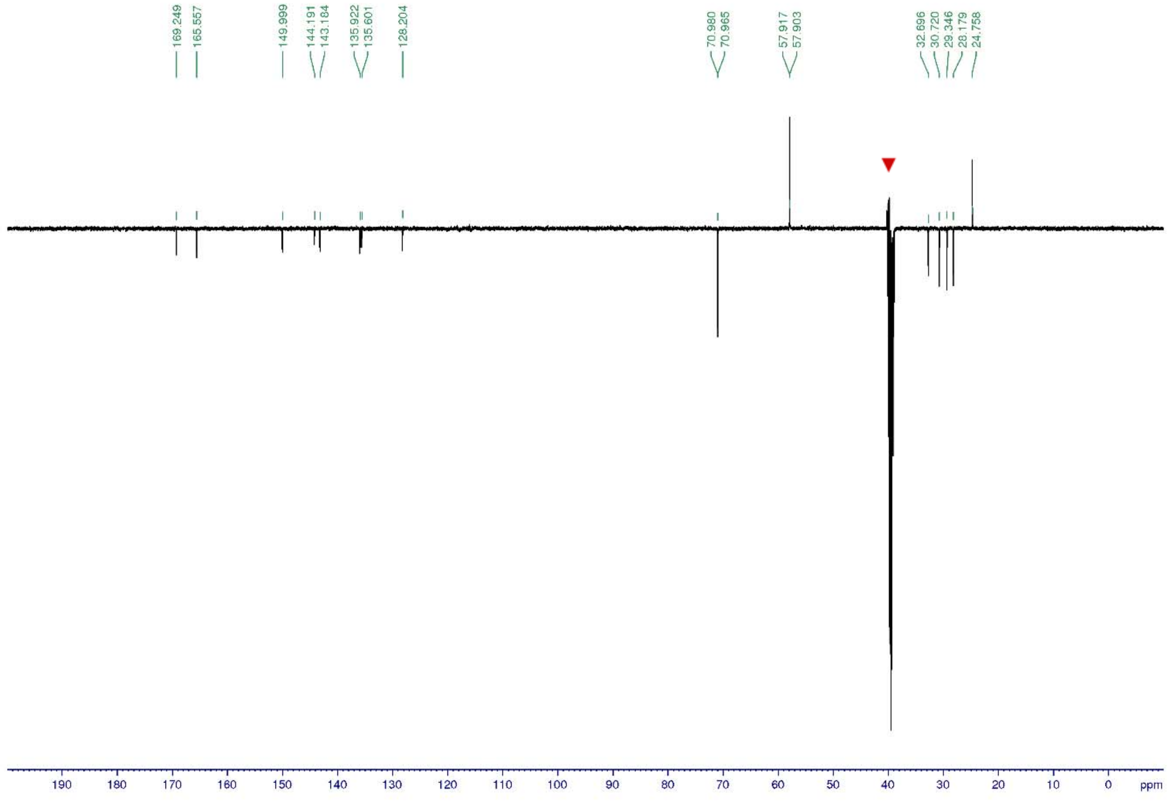

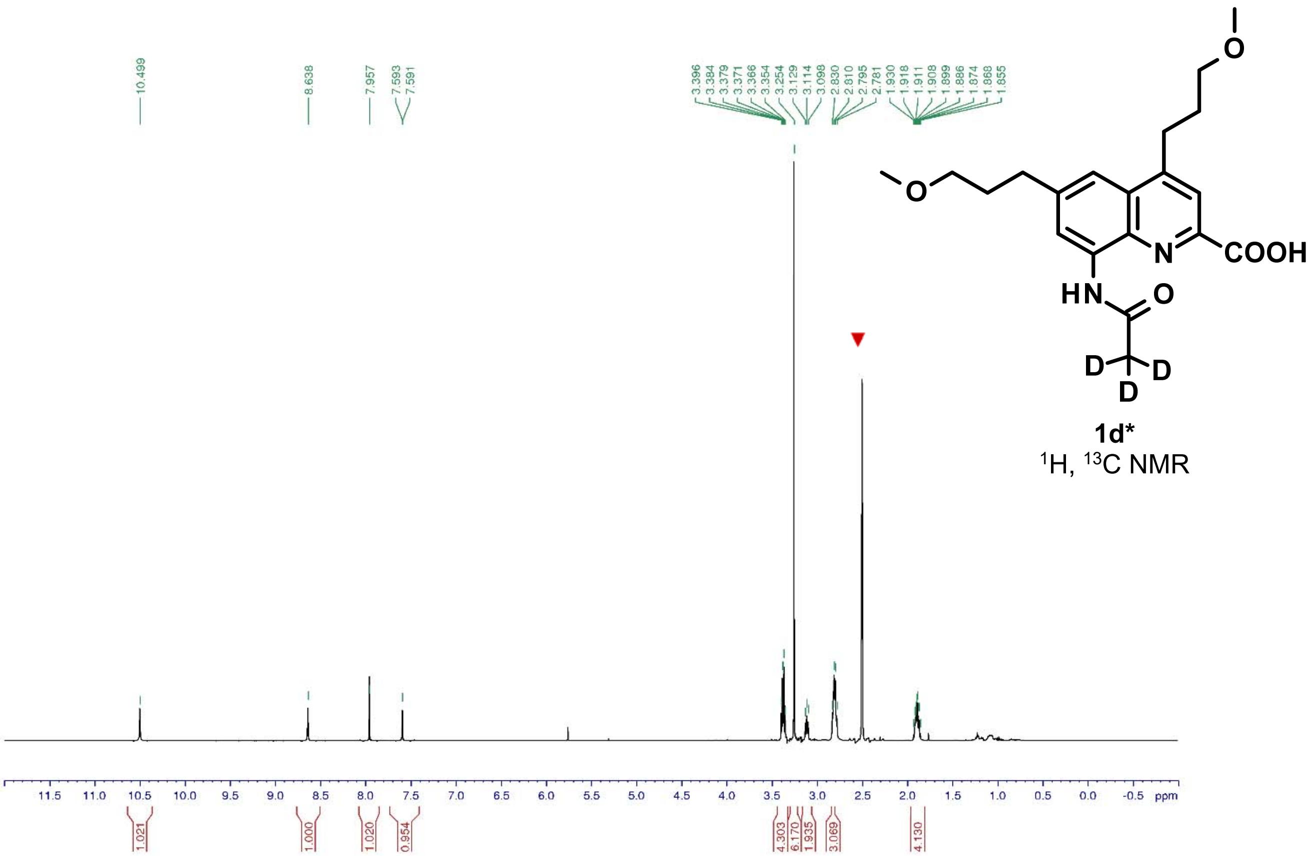

10.499
8.638
7.957
7.593
7.591
3.396 3.384 3.379 3.371 3.366 3.354 3.254 3.129 3.114 3.098 2.830 2.810 2.795 2.781 1.930 1.918 1.911 1.908 1.899 1.886 1.874 1.868 1.855
11.5 11.0 10.5 10.0 9.5 9.0 8.5 8.0 7.5 7.0 6.5 6.0 5.5 5.0 4.5 4.0 3.5 3.0 2.5 2.0 1.5 1.0 0.5 0.0 -0.5 ppm
1.021
1.000
1.020
0.954
4.303
6.170
1.935
3.069
4.130
O
O
N
COOH
HN
O
D
D
D

1d*
^{1}H, ^{13}C NMR

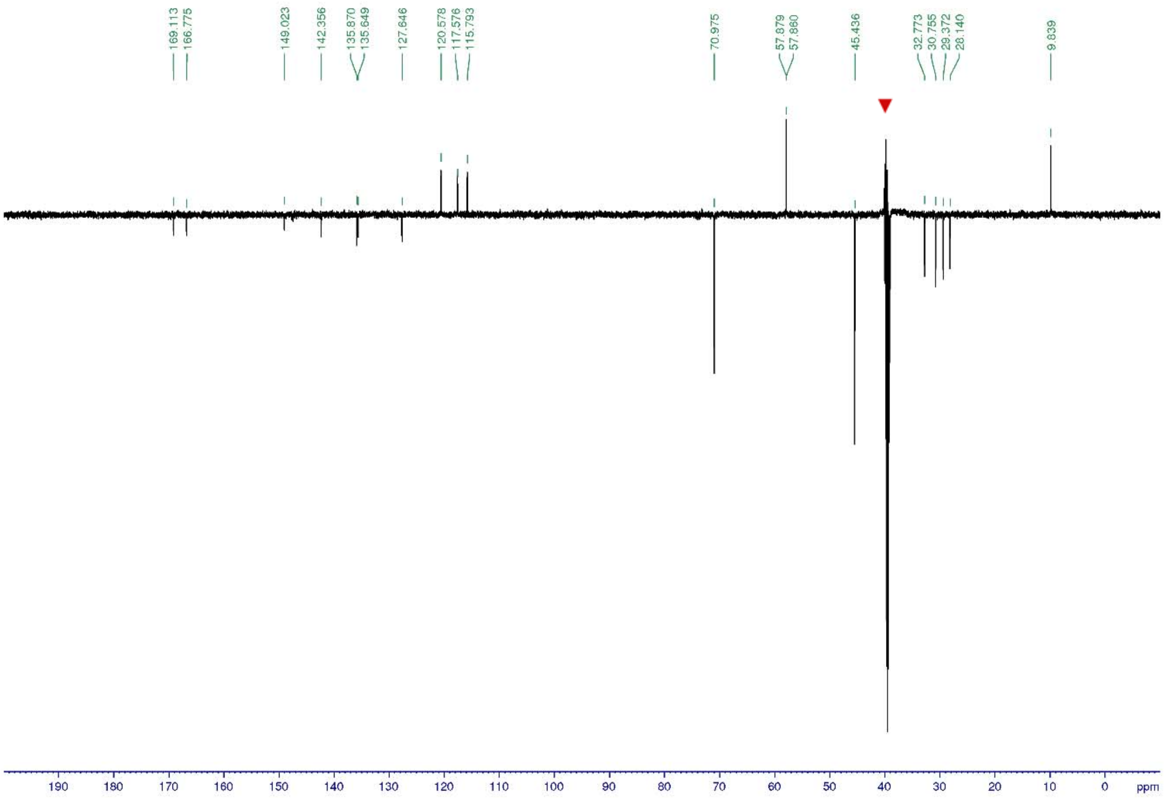

169.113
166.775
149.023
142.356
135.870
135.649
127.646
120.578
117.576
115.793
70.975
57.879
57.860
45.436
32.773
30.755
29.372
28.140
9.839
190 180 170 160 150 140 130 120 110 100 90 80 70 60 50 40 30 20 10 0 ppm

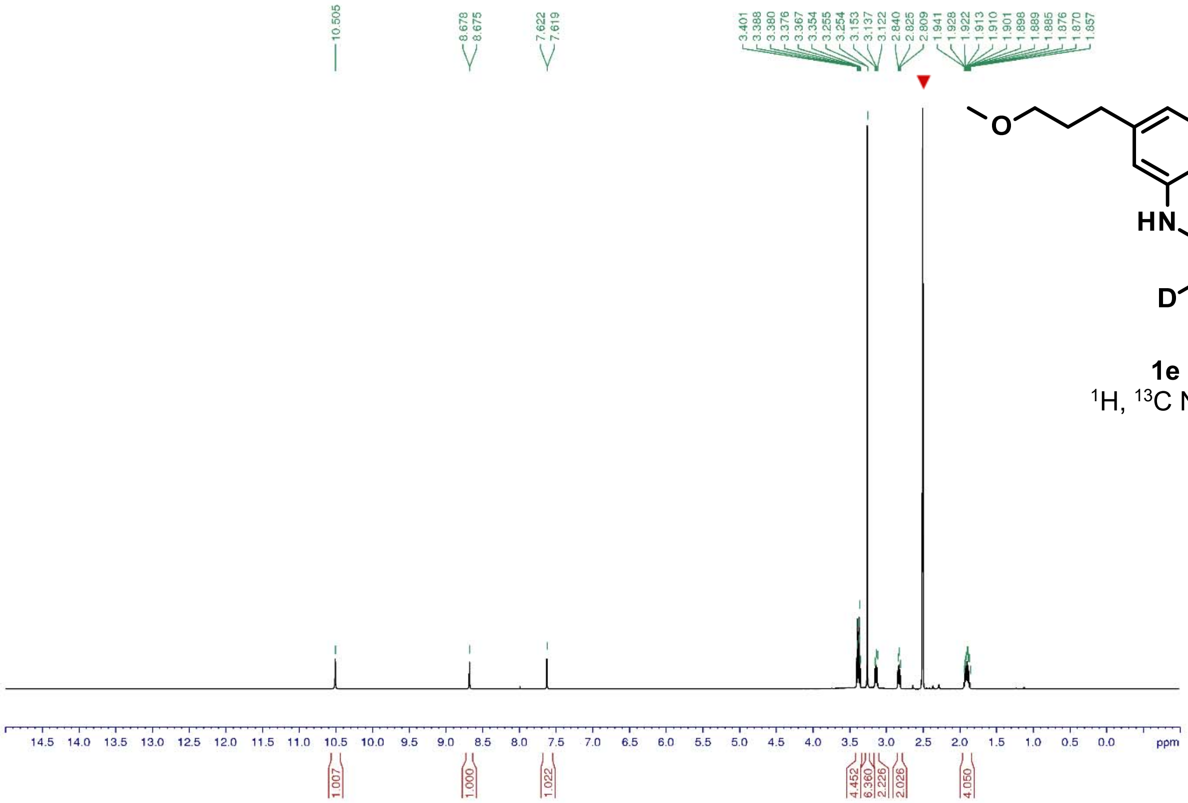

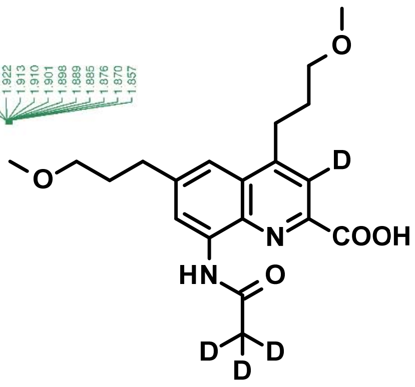

1e
^{1}H, ^{13}C NMR

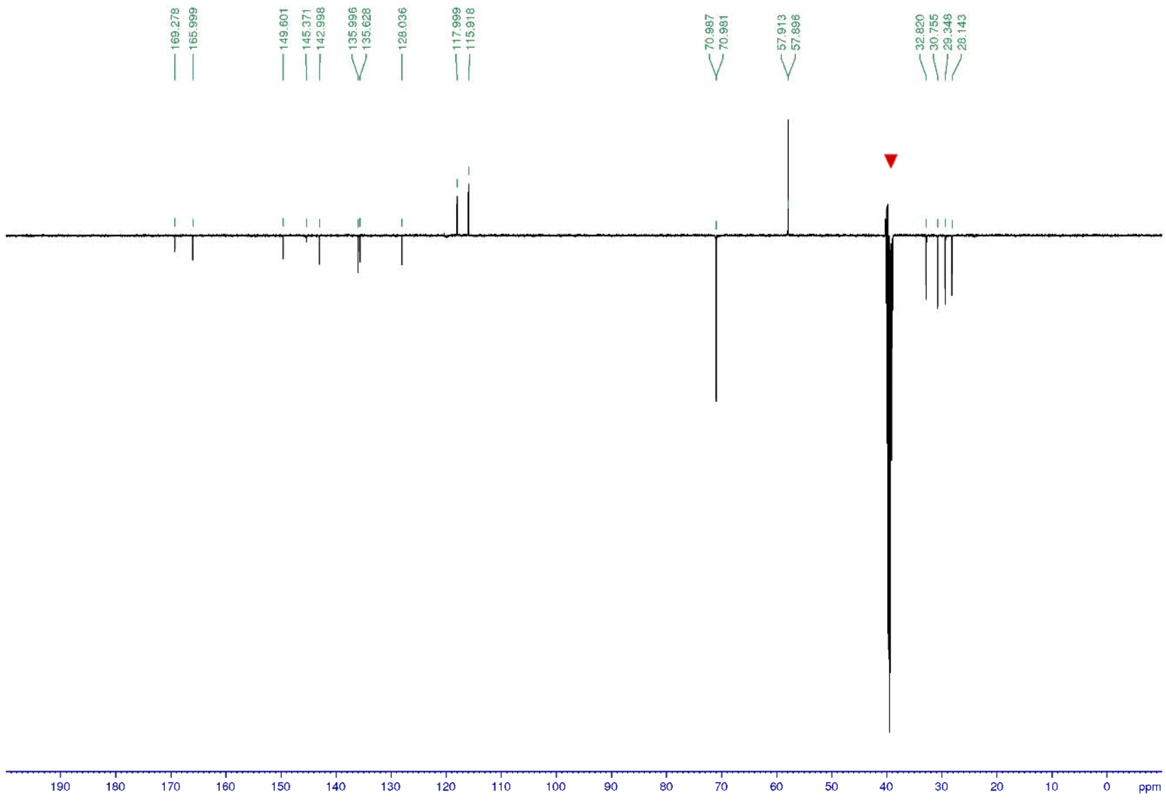

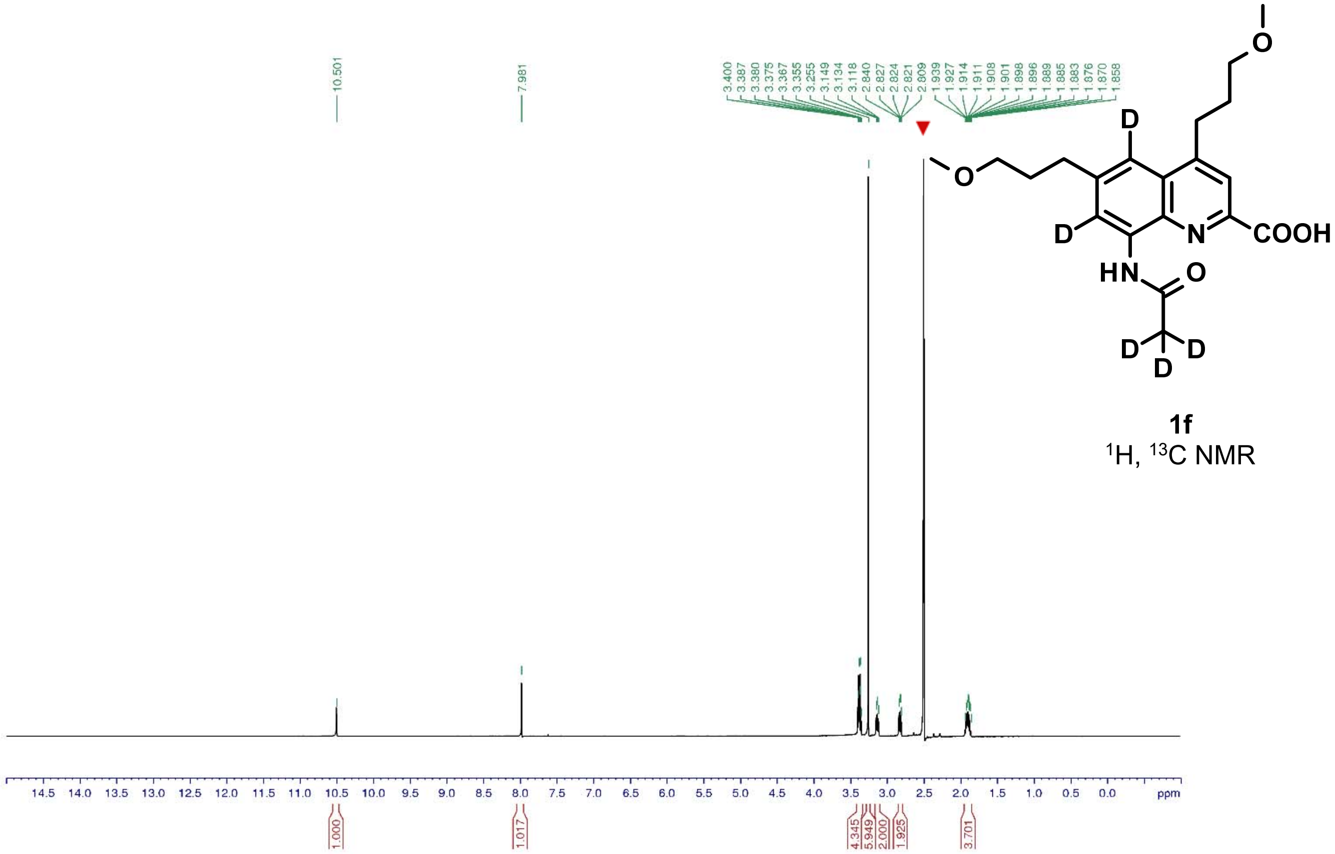

1f

^1H, ^{13}C NMR

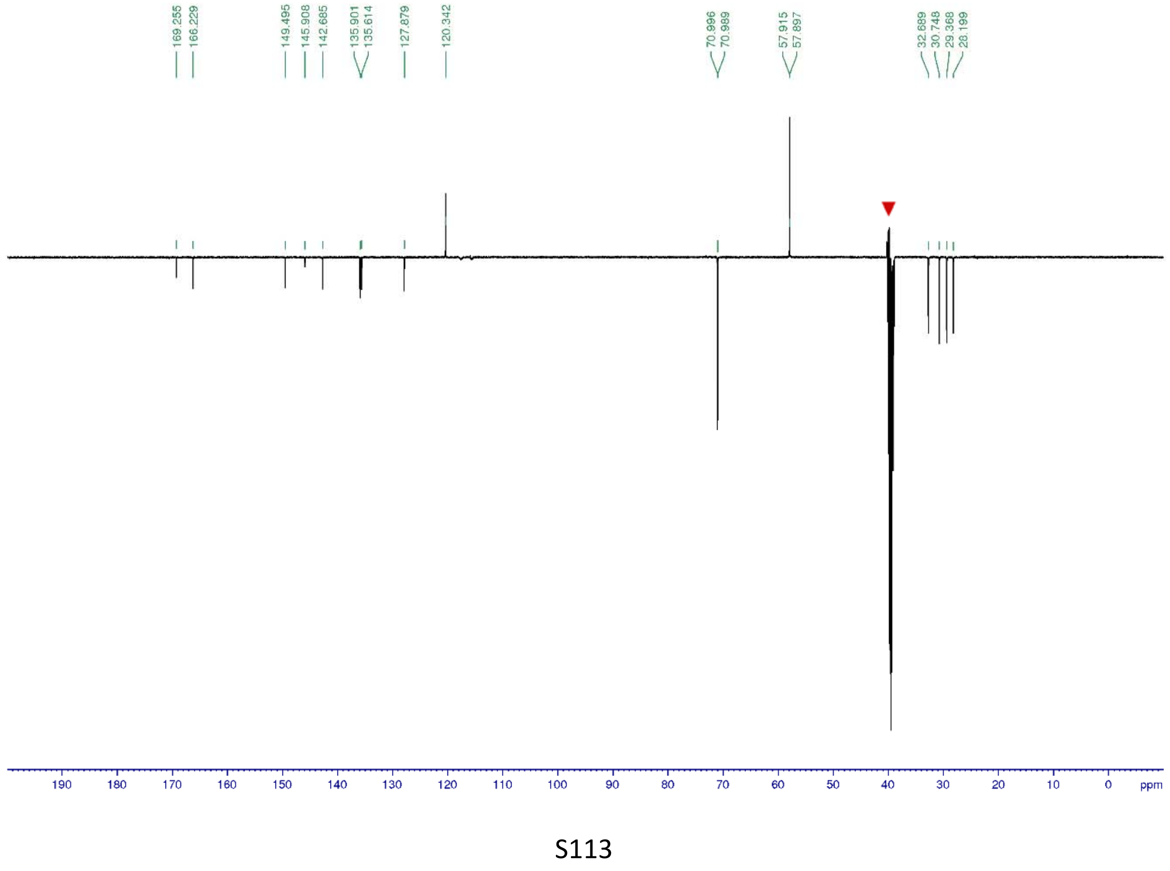

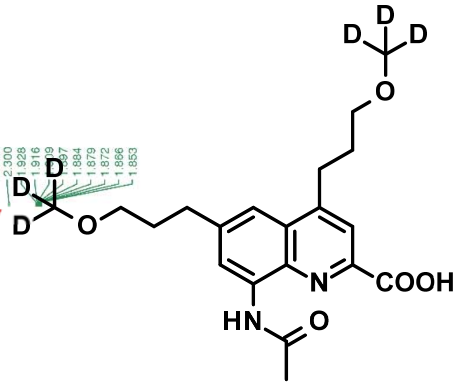
D
D
D
O
D
D
D
O
N
COOH
HN
O

1g
^{1}H, ^{13}C NMR

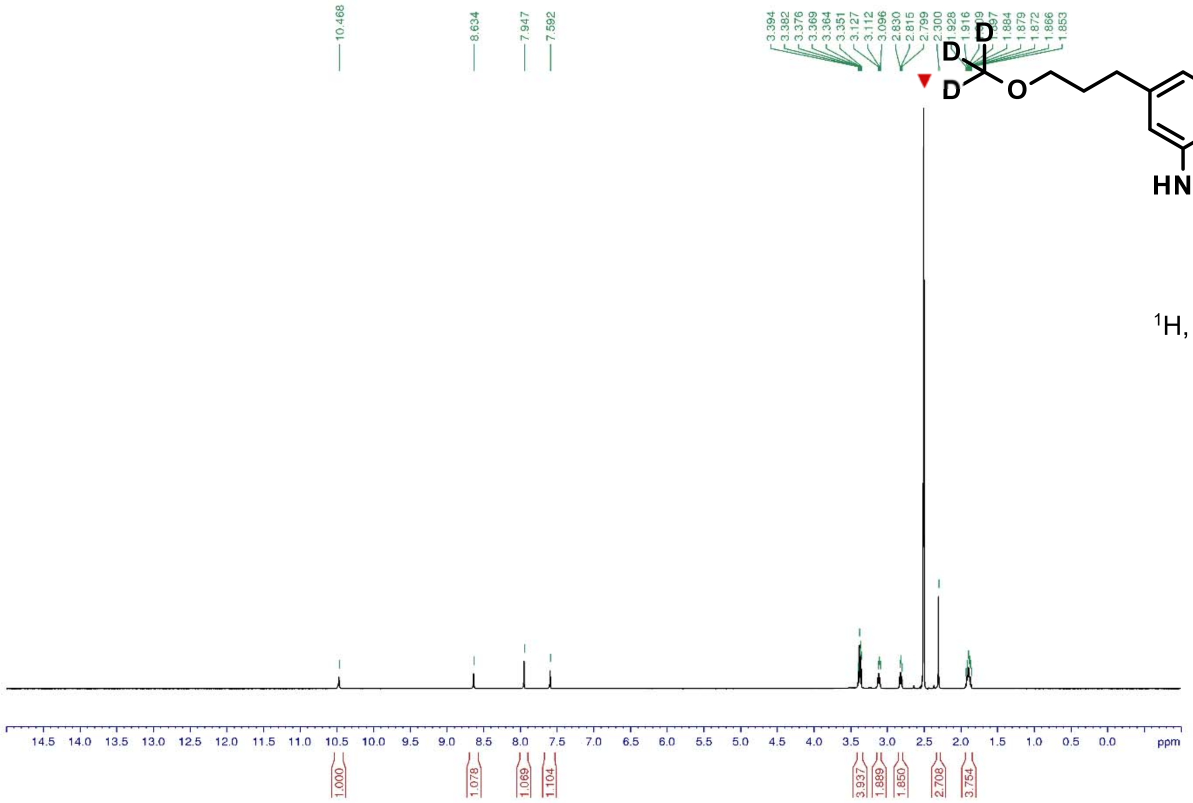
10.468
8.634
7.947
7.592
3.394
3.382
3.376
3.369
3.364
3.351
3.127
3.112
3.096
2.830
2.815
2.799
2.300
1.928
1.916
1.884
1.879
1.872
1.866
1.853
14.5 14.0 13.5 13.0 12.5 12.0 11.5 11.0 10.5 10.0 9.5 9.0 8.5 8.0 7.5 7.0 6.5 6.0 5.5 5.0 4.5 4.0 3.5 3.0 2.5 2.0 1.5 1.0 0.5 0.0 ppm
1.000
1.078
1.069
1.104
3.937
1.889
1.850
2.708
3.754

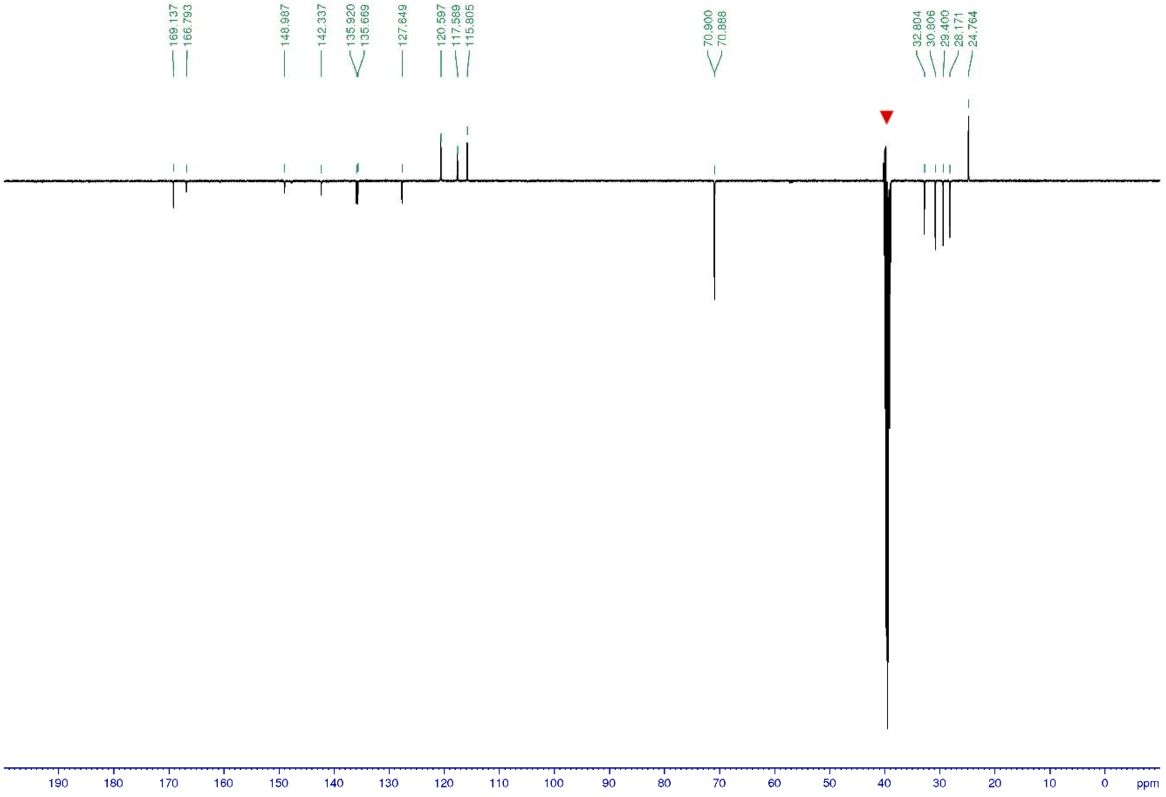
169.137
166.793
148.987
142.337
135.920
135.669
127.649
120.597
117.569
115.805
70.900
70.888
32.804
30.806
29.400
28.171
24.764
190 180 170 160 150 140 130 120 110 100 90 80 70 60 50 40 30 20 10 0 ppm

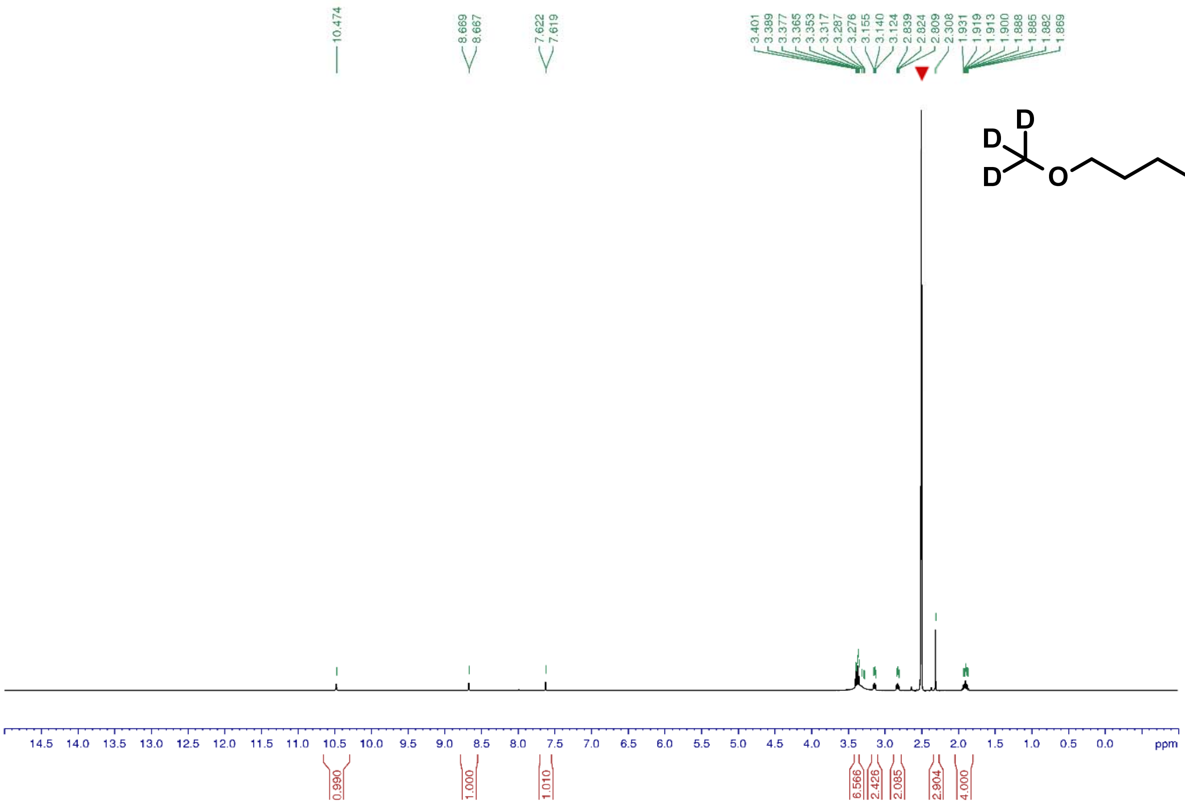
10.474
8.669
8.667
7.622
7.619
3.401
3.389
3.377
3.365
3.353
3.317
3.287
3.276
3.155
3.140
3.124
2.839
2.824
2.809
2.308
1.931
1.919
1.913
1.900
1.888
1.885
1.882
1.869
0.990
1.000
1.010
6.566
2.426
2.085
2.904
4.000
ppm

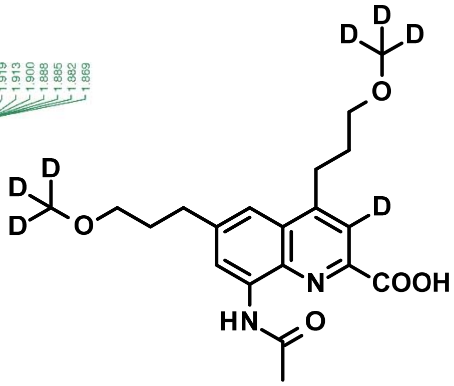
D
D
D
O
D
D
D
O
D
N
COOH
HN
O

1h

^{1}H, ^{13}C NMR

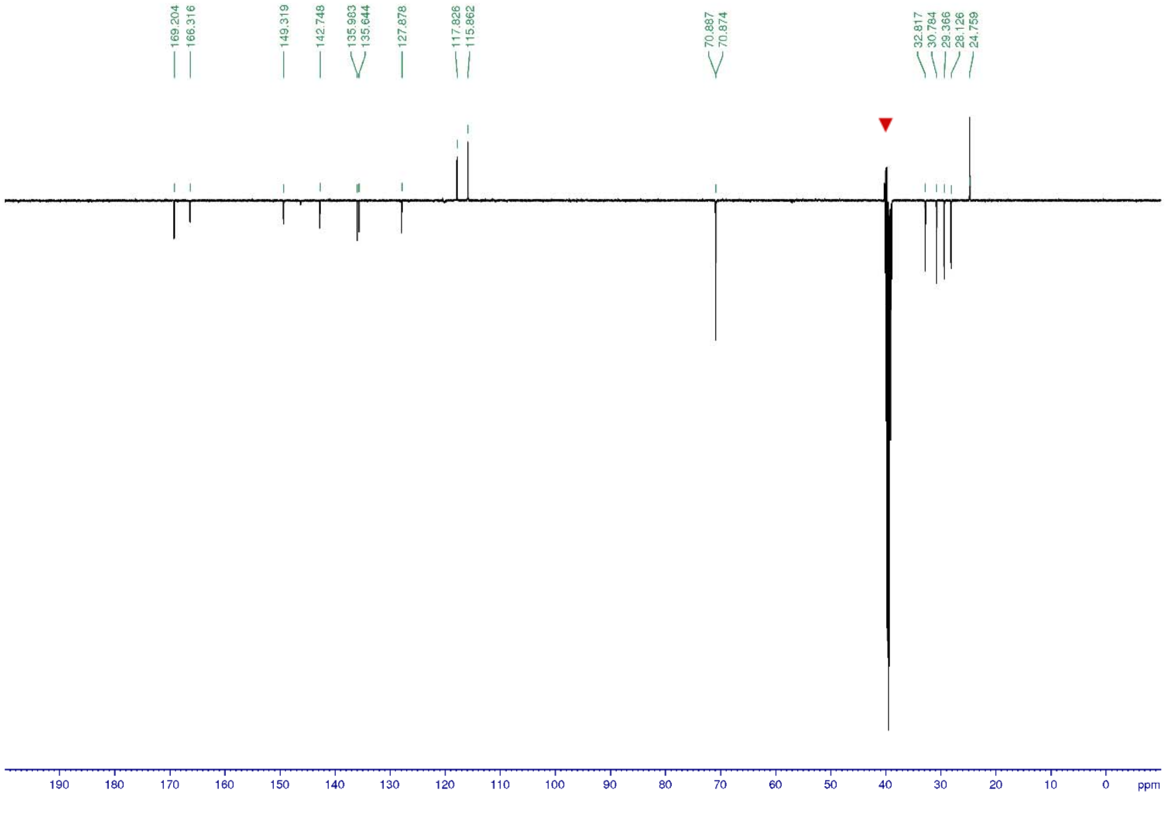
169.204
166.316
149.319
142.748
135.983
135.644
127.878
117.826
115.862
70.887
70.874
32.817
30.784
29.366
28.126
24.759
ppm

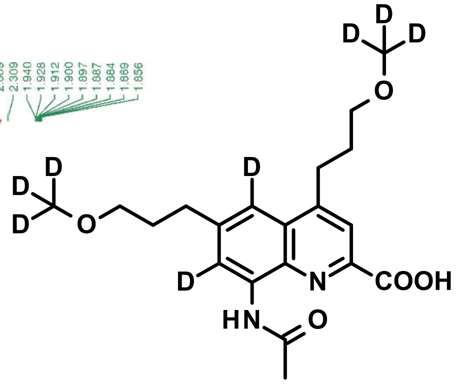

1i
^1H, ^{13}C NMR

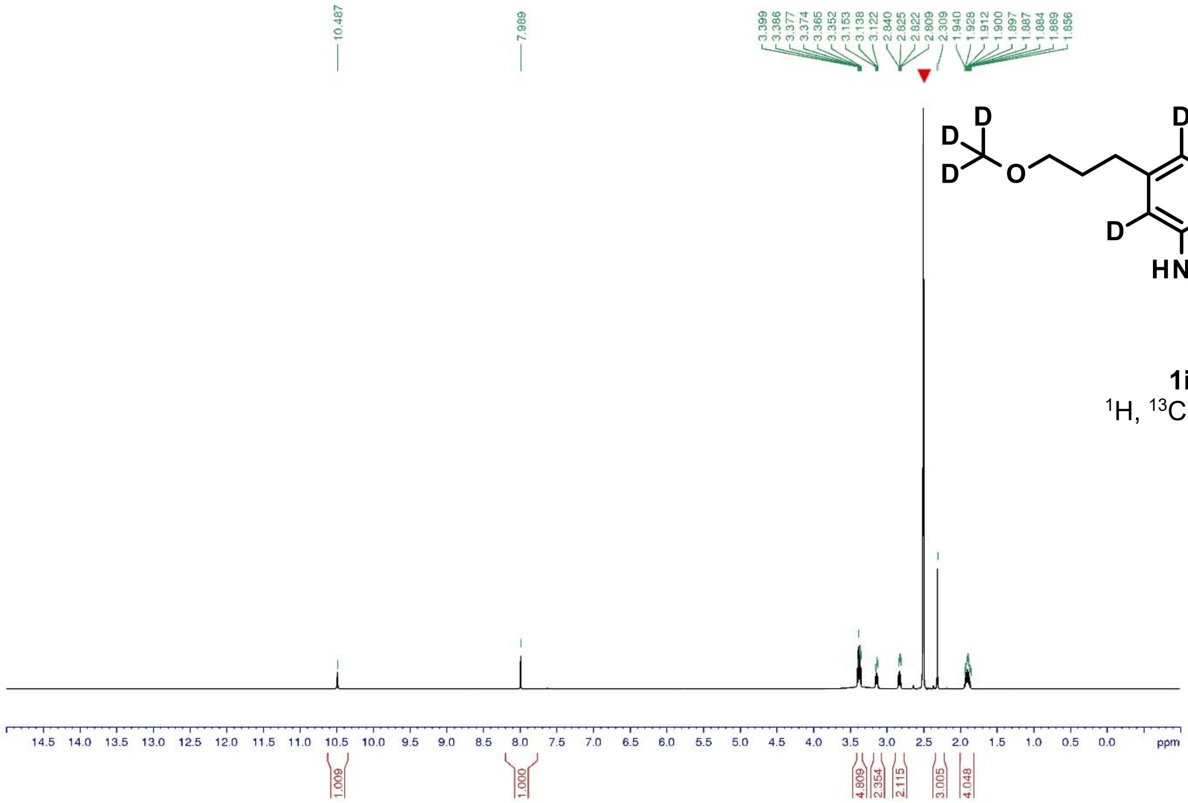

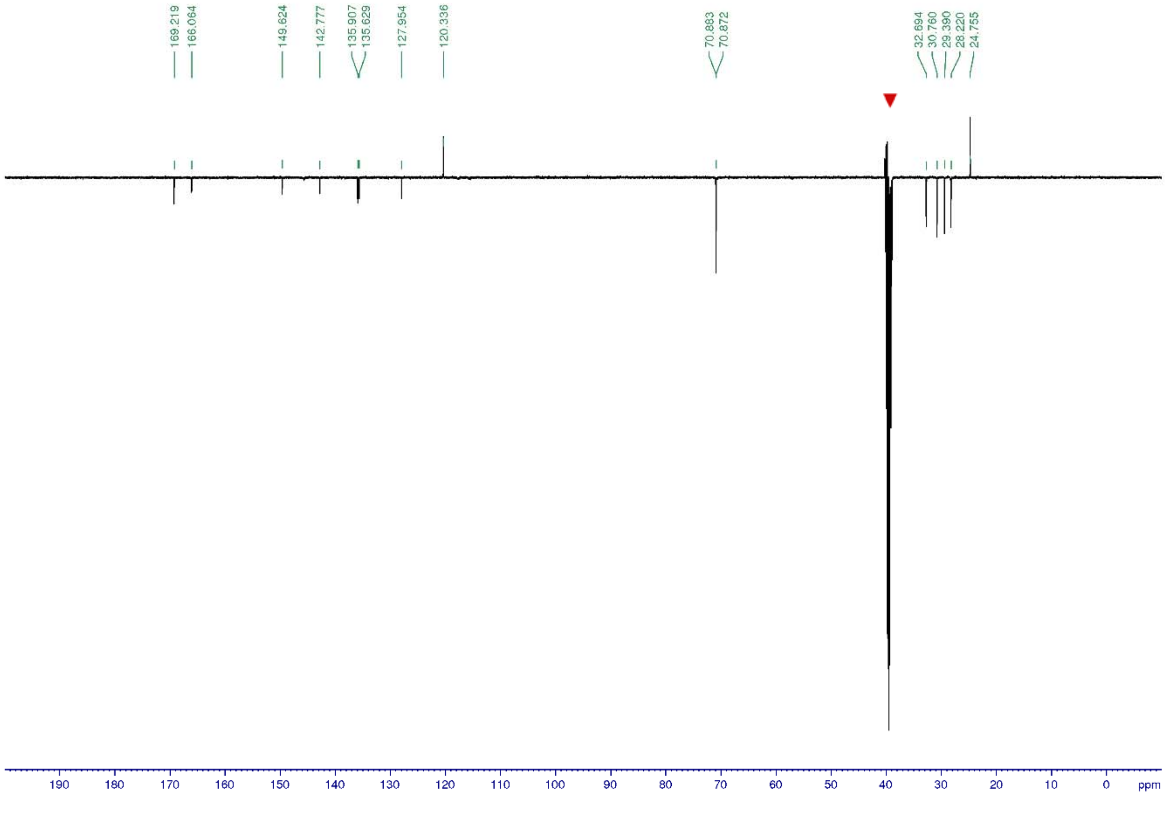

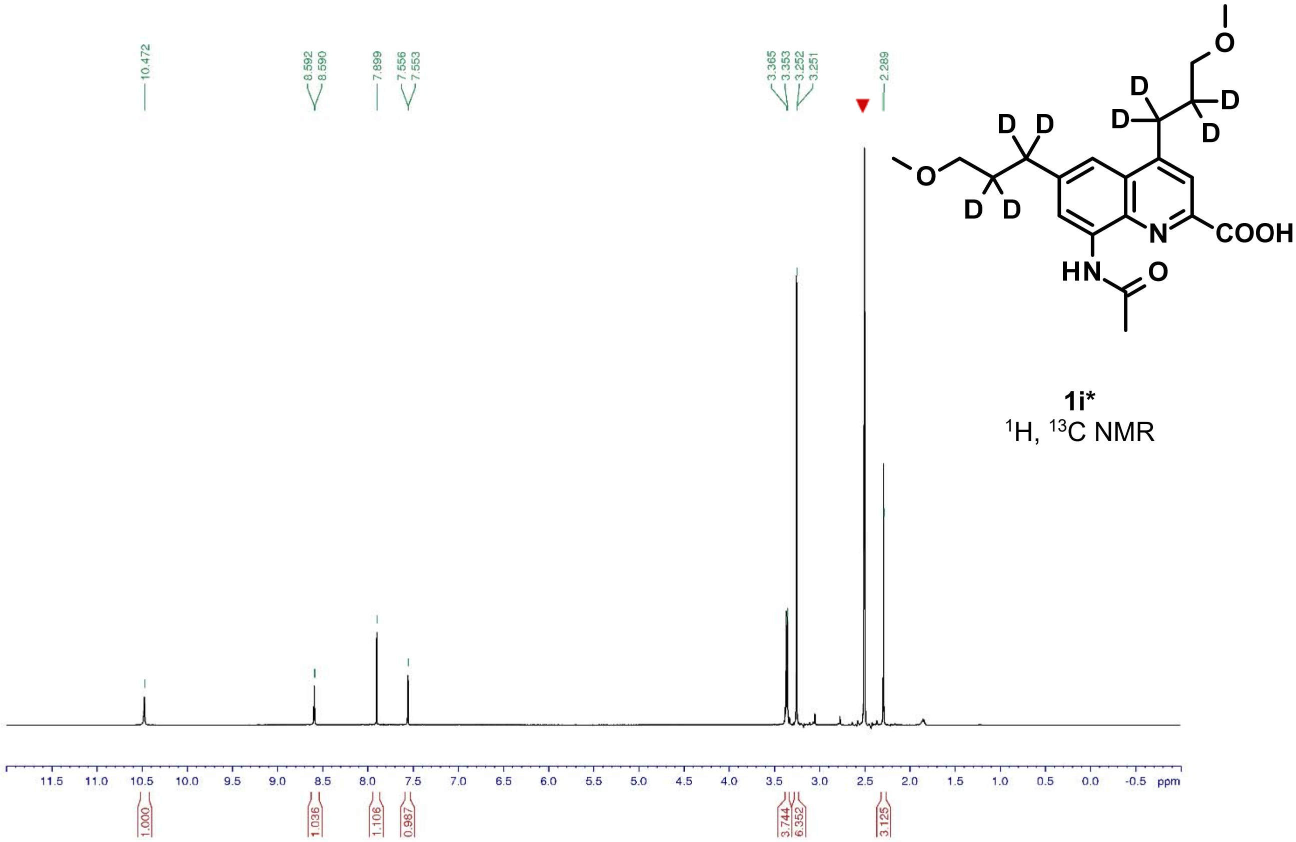
10.472
8.592
8.590
7.899
7.556
7.553
3.365
3.353
3.252
3.251
2.289
11.5 11.0 10.5 10.0 9.5 9.0 8.5 8.0 7.5 7.0 6.5 6.0 5.5 5.0 4.5 4.0 3.5 3.0 2.5 2.0 1.5 1.0 0.5 0.0 -0.5 ppm
1.000
1.036
1.106
0.987
3.744
6.352
3.125
D
D
D
D
D
D
D
D
O
O
N
COOH
HN
O

1i*

^{1}H, ^{13}C NMR

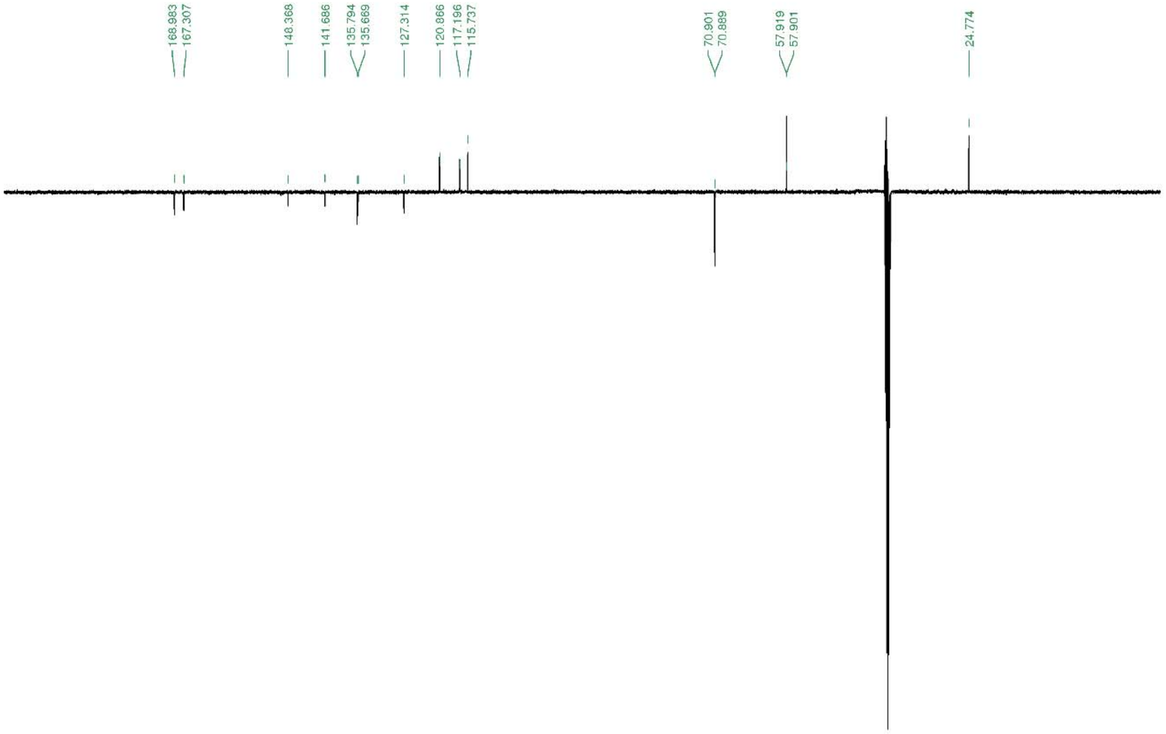
168.983
167.307
148.368
141.686
135.794
135.669
127.314
120.866
117.196
115.737
70.901
70.889
57.919
57.901
24.774
190 180 170 160 150 140 130 120 110 100 90 80 70 60 50 40 30 20 10 0 ppm

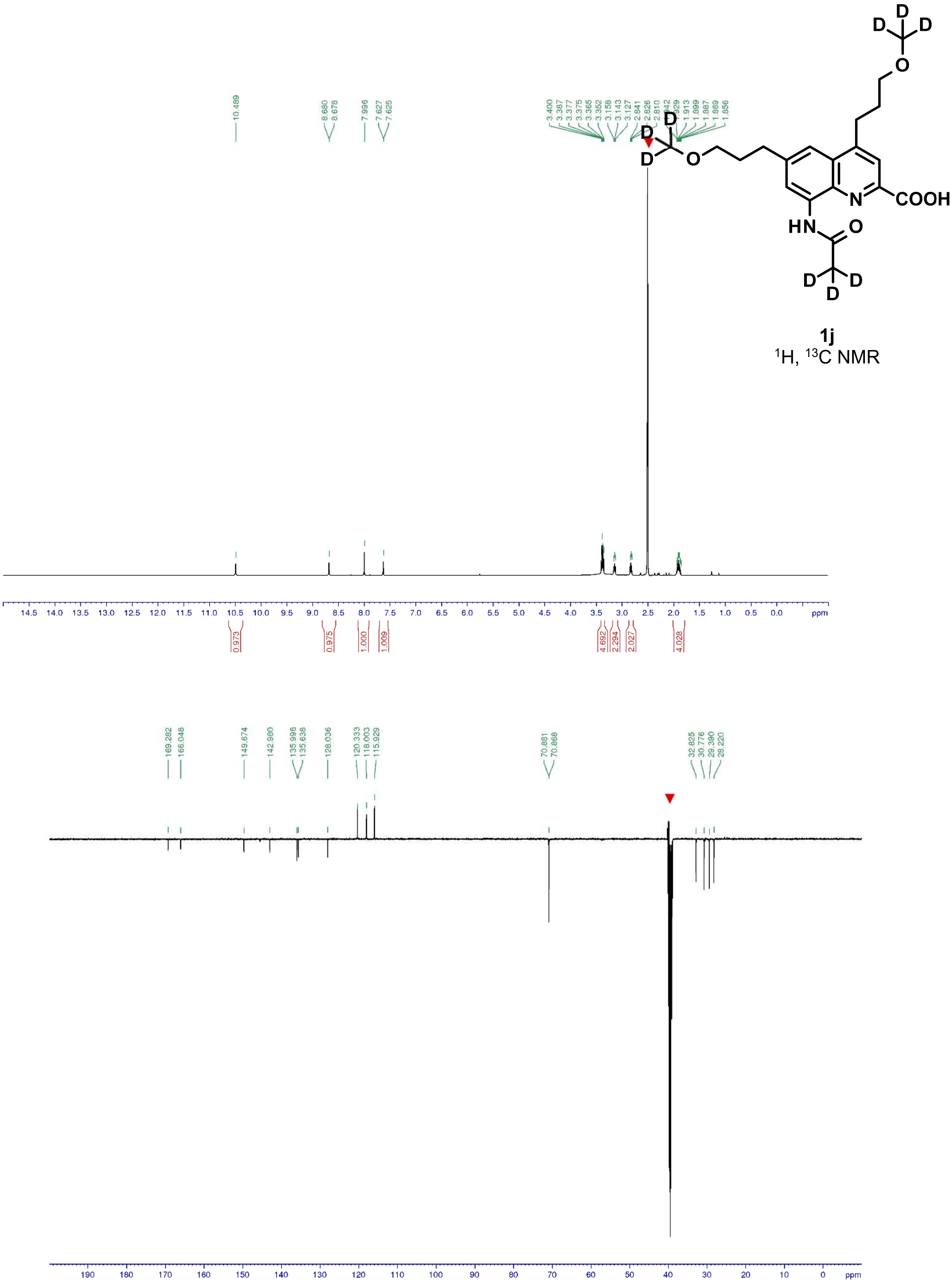
10.489
8.680
8.678
7.996
7.627
7.625
3.400
3.387
3.377
3.375
3.365
3.352
3.158
3.143
3.127
2.841
2.826
2.810
1.913
1.899
1.887
1.869
1.856
0.973
0.975
1.000
1.009
4.692
2.294
2.027
4.028
ppm
D
O
N
COOH
HN
169.282
166.048
149.674
142.980
135.996
135.638
128.036
120.333
118.003
115.929
70.881
70.868
32.825
30.776
29.390
28.220
ppm

1j

^{1}H, ^{13}C NMR

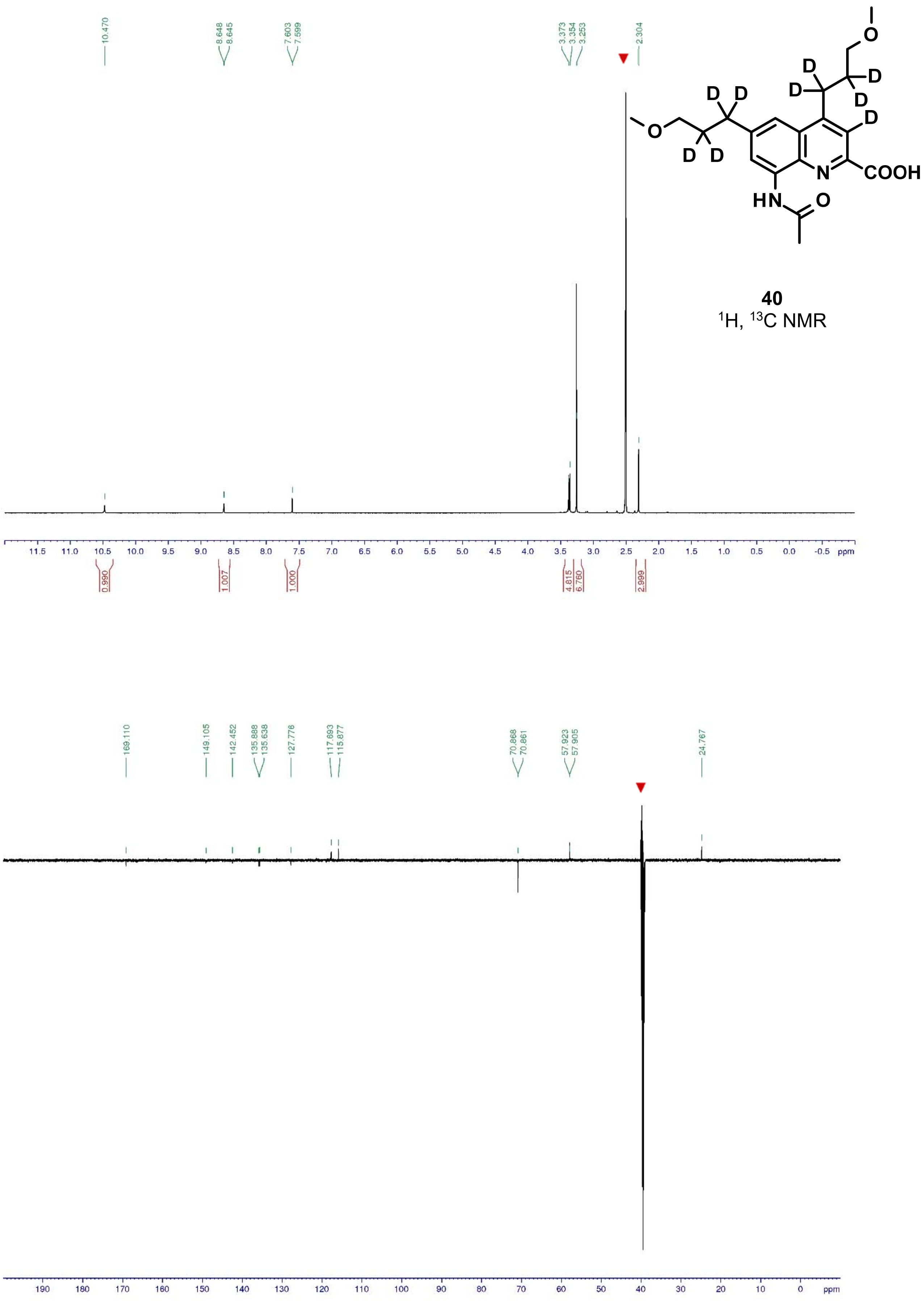

40
^{1}H, ^{13}C NMR

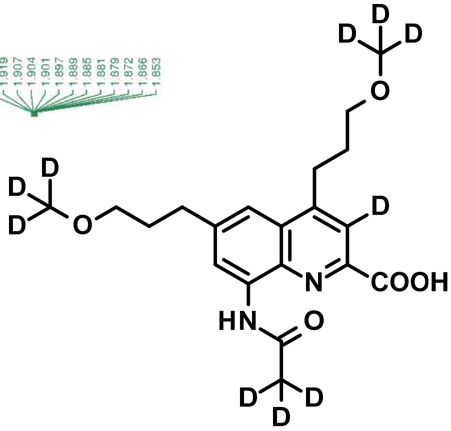

1k
^{1}H, ^{13}C NMR

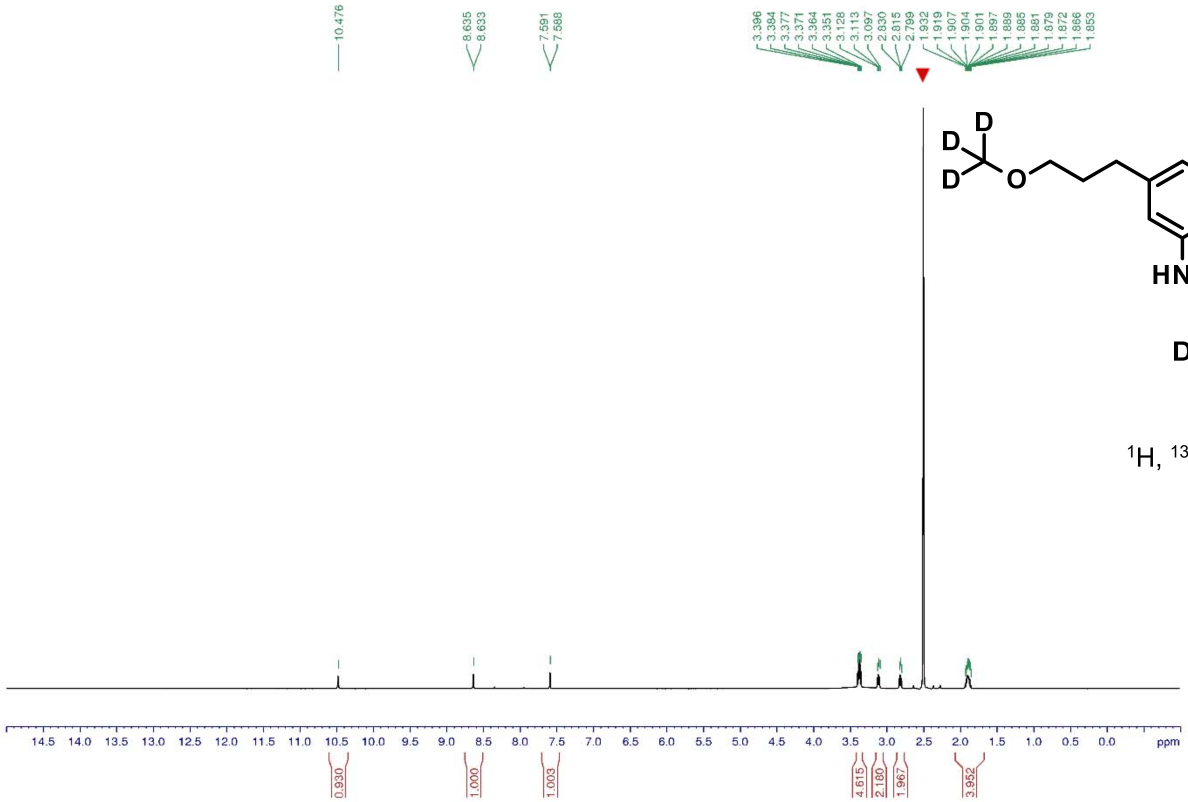
10.476
8.635
8.633
7.591
7.588
3.396
3.384
3.377
3.371
3.364
3.351
3.128
3.113
3.097
2.830
2.815
2.799
1.932
1.919
1.907
1.904
1.901
1.897
1.889
1.885
1.881
1.879
1.872
1.866
1.853
14.5 14.0 13.5 13.0 12.5 12.0 11.5 11.0 10.5 10.0 9.5 9.0 8.5 8.0 7.5 7.0 6.5 6.0 5.5 5.0 4.5 4.0 3.5 3.0 2.5 2.0 1.5 1.0 0.5 0.0 ppm
0.930
1.000
1.003
4.615
2.180
1.967
3.952

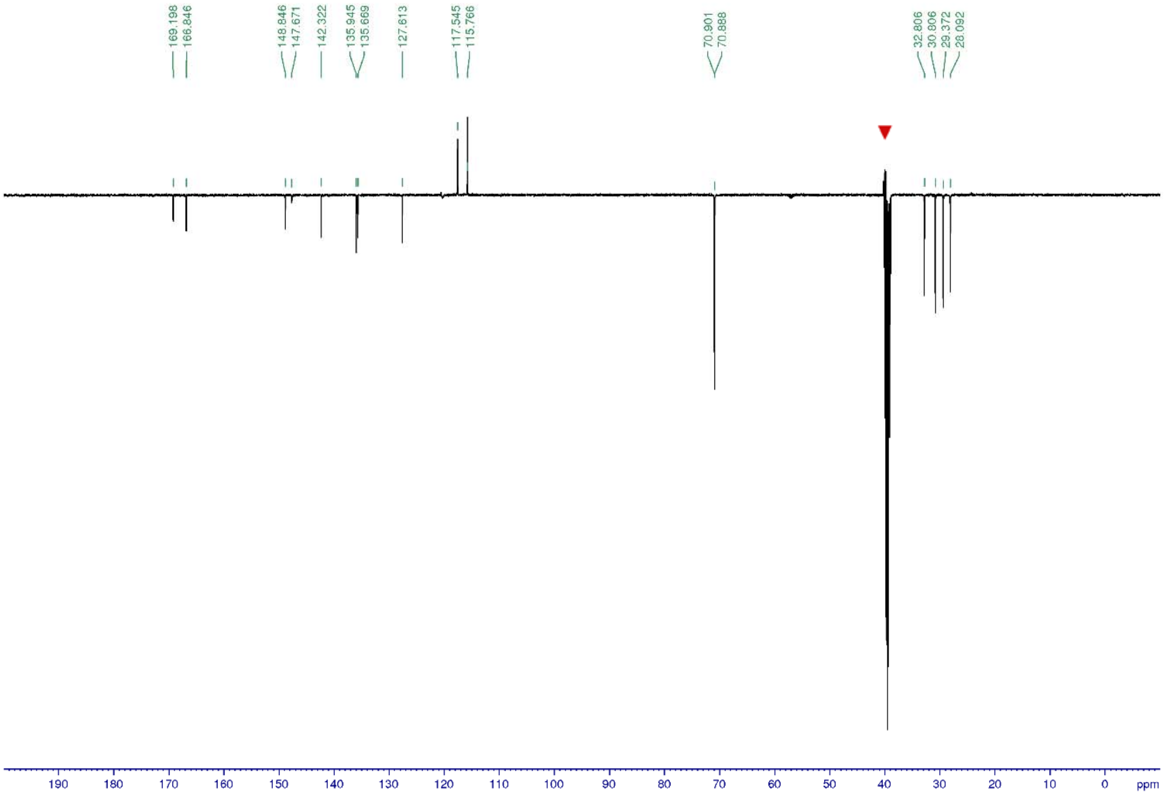
169.198
166.846
148.846
147.671
142.322
135.945
135.669
127.613
117.545
115.766
70.901
70.888
32.806
30.806
29.372
28.092
190 180 170 160 150 140 130 120 110 100 90 80 70 60 50 40 30 20 10 0 ppm

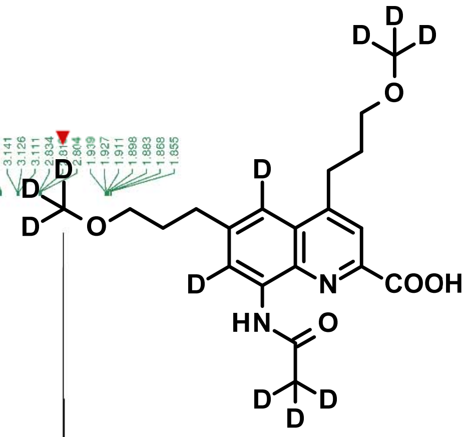
D
D
D
O
D
D
D
D
O
D
N
COOH
HN
O
D
D
D

1l
^{1}H, ^{13}C NMR

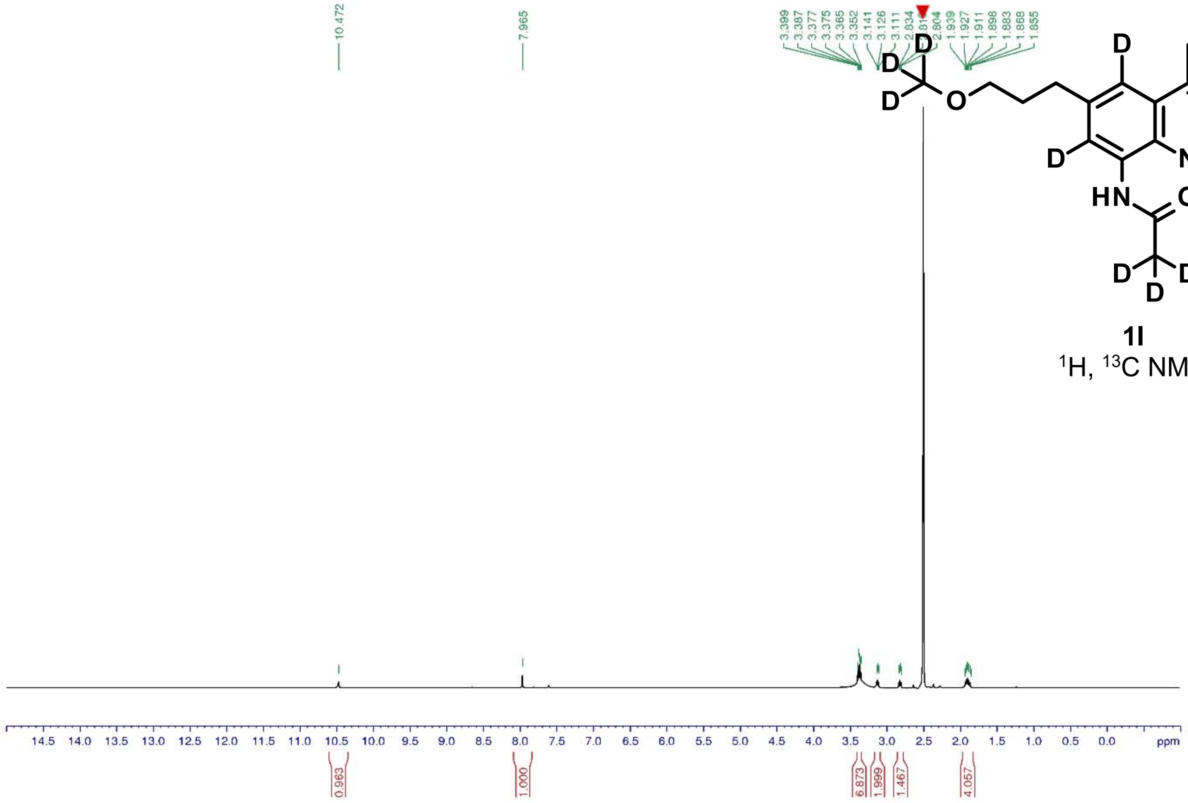
10.472
7.965
3.399
3.387
3.377
3.375
3.365
3.352
3.141
3.126
3.111
2.834
2.804
1.939
1.927
1.911
1.898
1.883
1.868
1.855
14.5 14.0 13.5 13.0 12.5 12.0 11.5 11.0 10.5 10.0 9.5 9.0 8.5 8.0 7.5 7.0 6.5 6.0 5.5 5.0 4.5 4.0 3.5 3.0 2.5 2.0 1.5 1.0 0.5 0.0 ppm
0.963
1.000
6.873
1.999
1.467
4.057

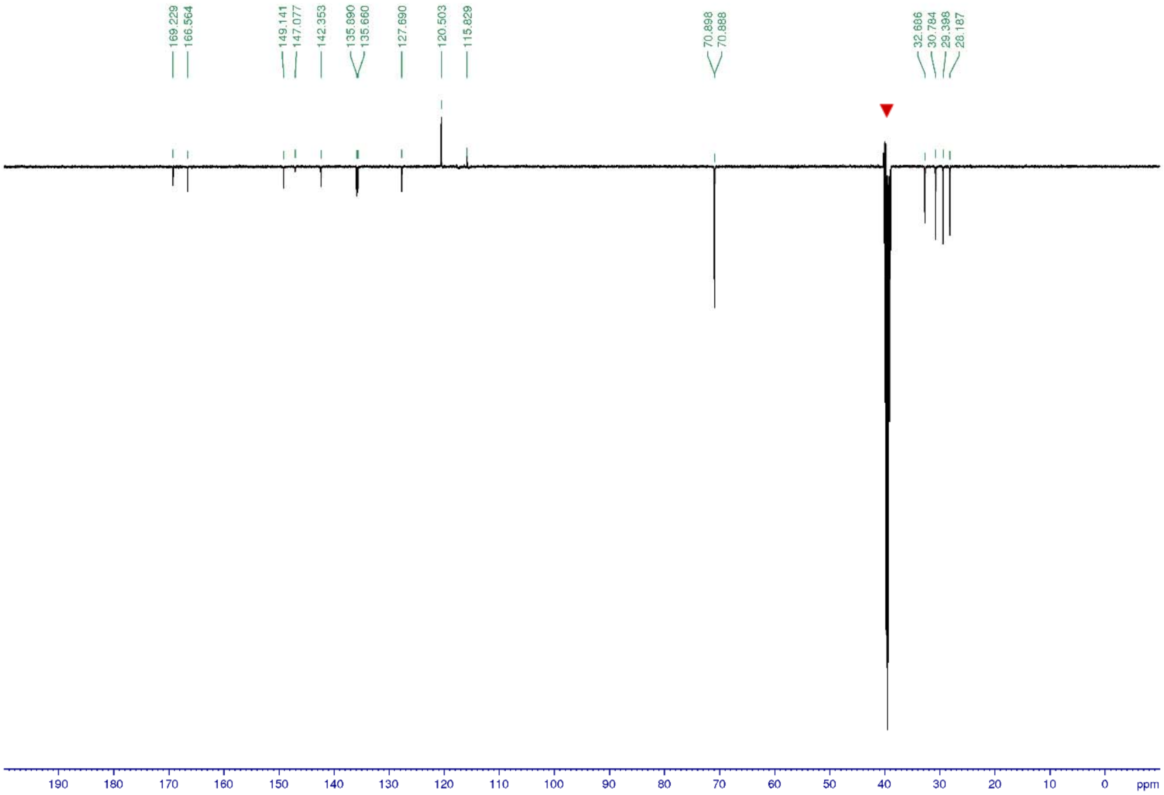
169.229
166.564
149.141
147.077
142.353
135.690
135.660
127.690
120.503
115.829
70.898
70.888
32.666
30.784
29.398
28.187
190 180 170 160 150 140 130 120 110 100 90 80 70 60 50 40 30 20 10 0 ppm

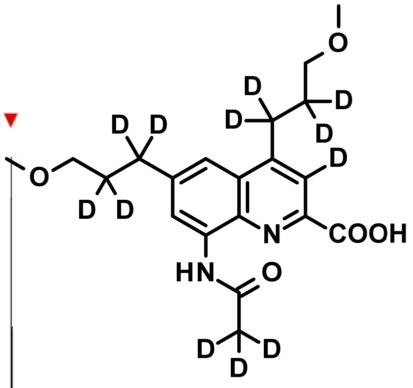
D
D
D
D
D
D
D
D
D
D
D
D
O
O
N
COOH
HN
O

1m

^{1}H, ^{13}C NMR

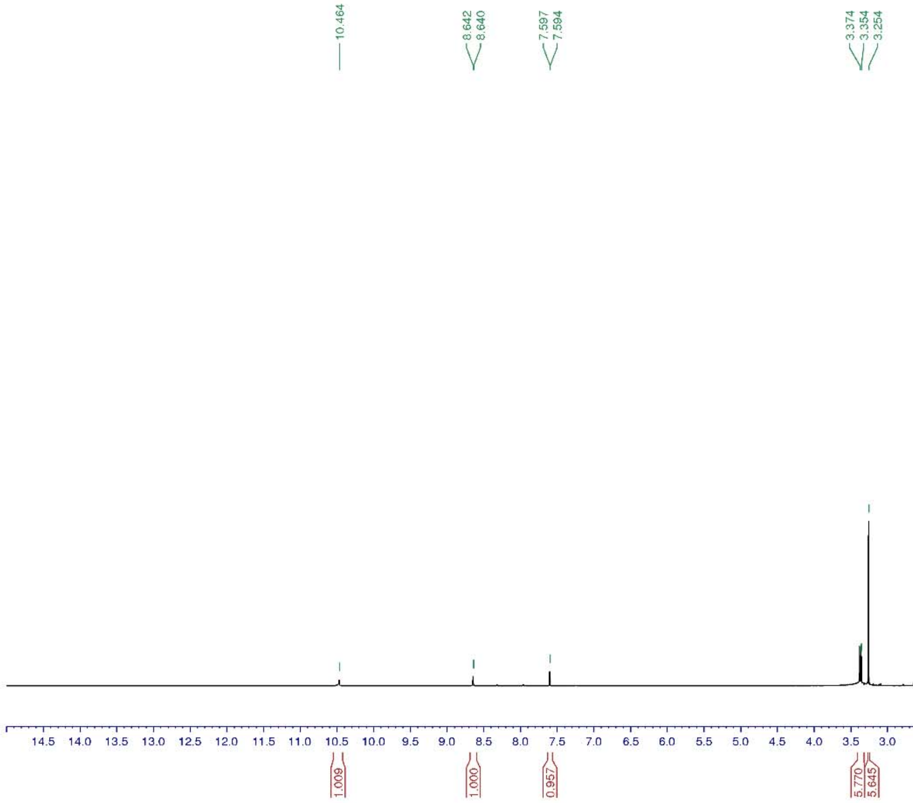
10.464
8.642
8.640
7.597
7.594
3.374
3.354
3.254
14.5 14.0 13.5 13.0 12.5 12.0 11.5 11.0 10.5 10.0 9.5 9.0 8.5 8.0 7.5 7.0 6.5 6.0 5.5 5.0 4.5 4.0 3.5 3.0 2.5 2.0 1.5 1.0 0.5 0.0 ppm
1.009
1.000
0.957
5.770
5.645

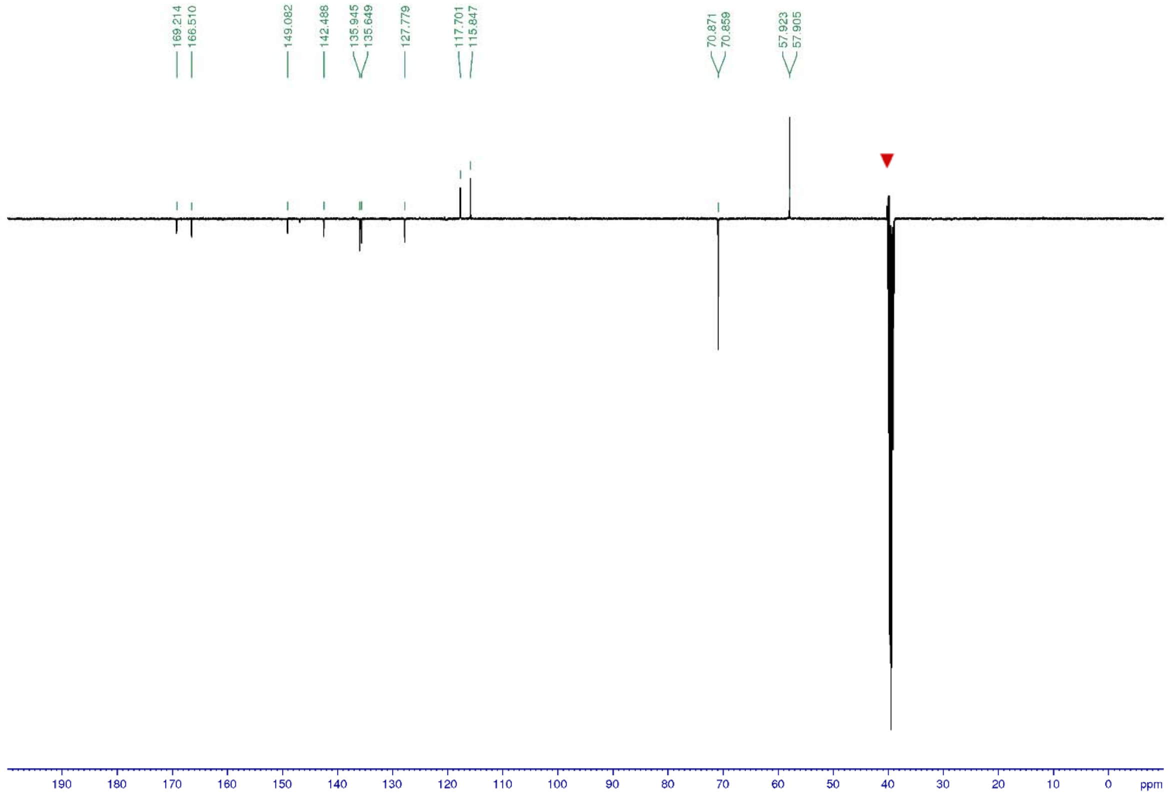
169.214
166.510
149.082
142.488
135.945
135.649
127.779
117.701
115.847
70.871
70.859
57.923
57.905
190 180 170 160 150 140 130 120 110 100 90 80 70 60 50 40 30 20 10 0 ppm

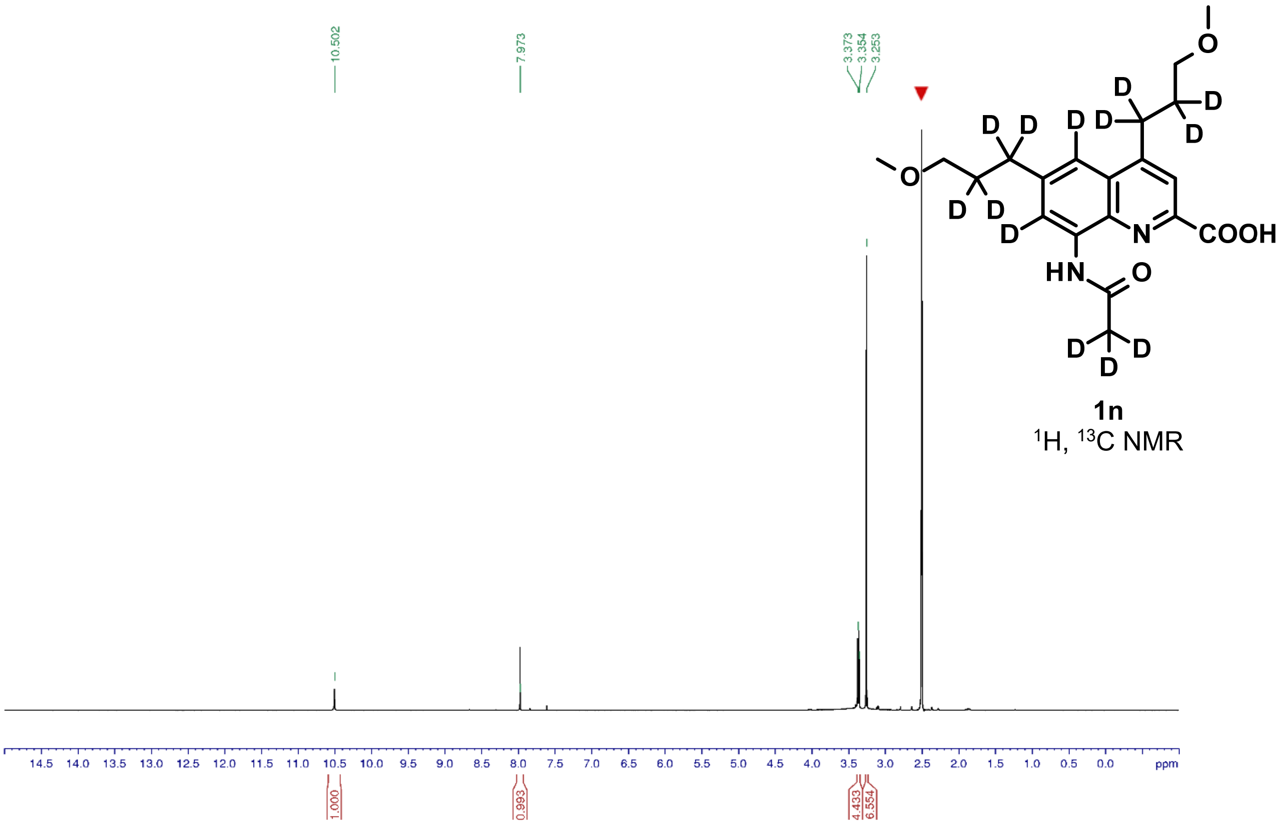

10.502
7.973
3.373
3.354
3.253
D
D
D
D
D
D
D
D
D
O
O
N
COOH
HN
O
D
D
D
14.5 14.0 13.5 13.0 12.5 12.0 11.5 11.0 10.5 10.0 9.5 9.0 8.5 8.0 7.5 7.0 6.5 6.0 5.5 5.0 4.5 4.0 3.5 3.0 2.5 2.0 1.5 1.0 0.5 0.0 ppm
1.000
0.993
4.433
6.554

1n

^{1}H, ^{13}C NMR

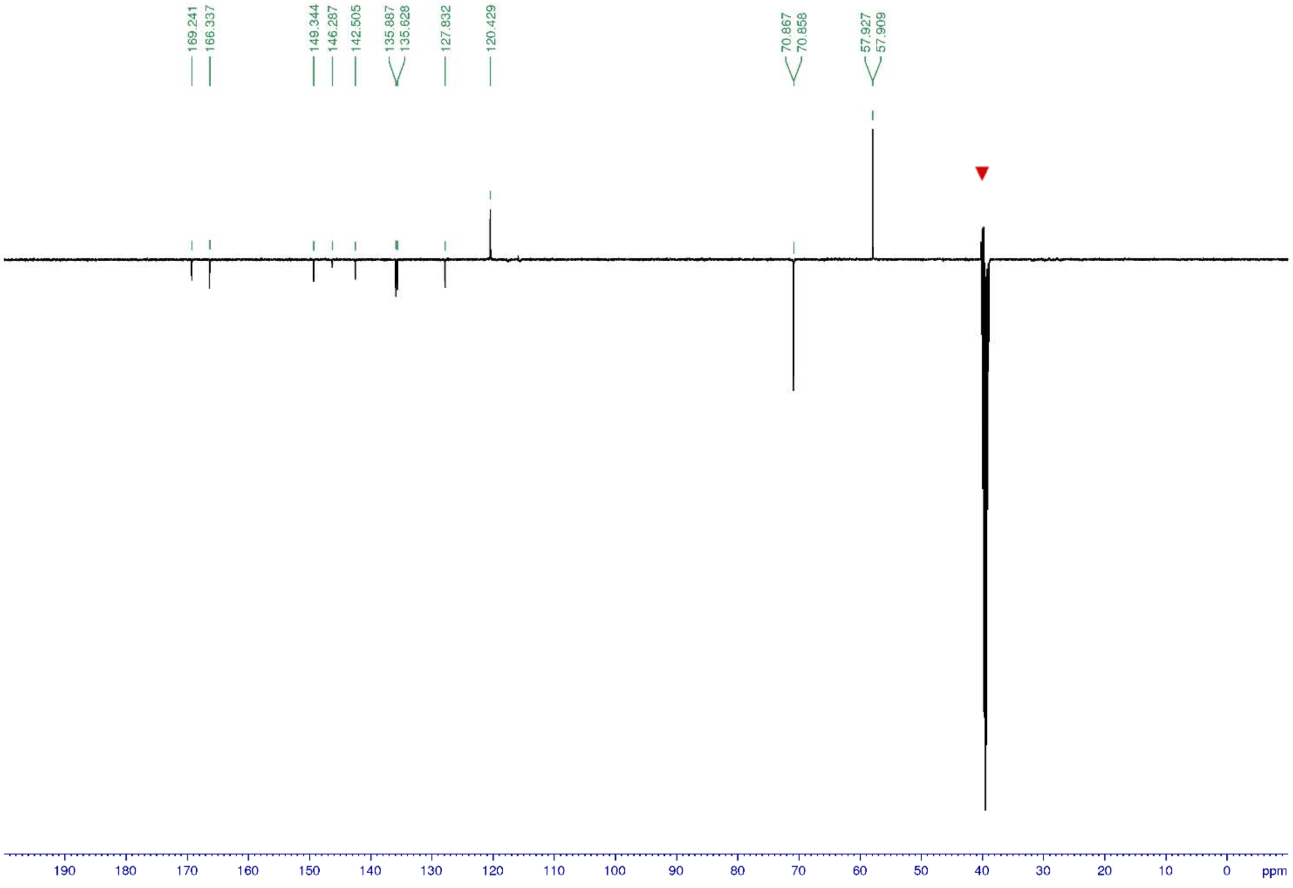

169.241
166.337
149.344
146.287
142.505
135.887
135.628
127.832
120.429
70.867
70.858
57.927
57.909
190 180 170 160 150 140 130 120 110 100 90 80 70 60 50 40 30 20 10 0 ppm

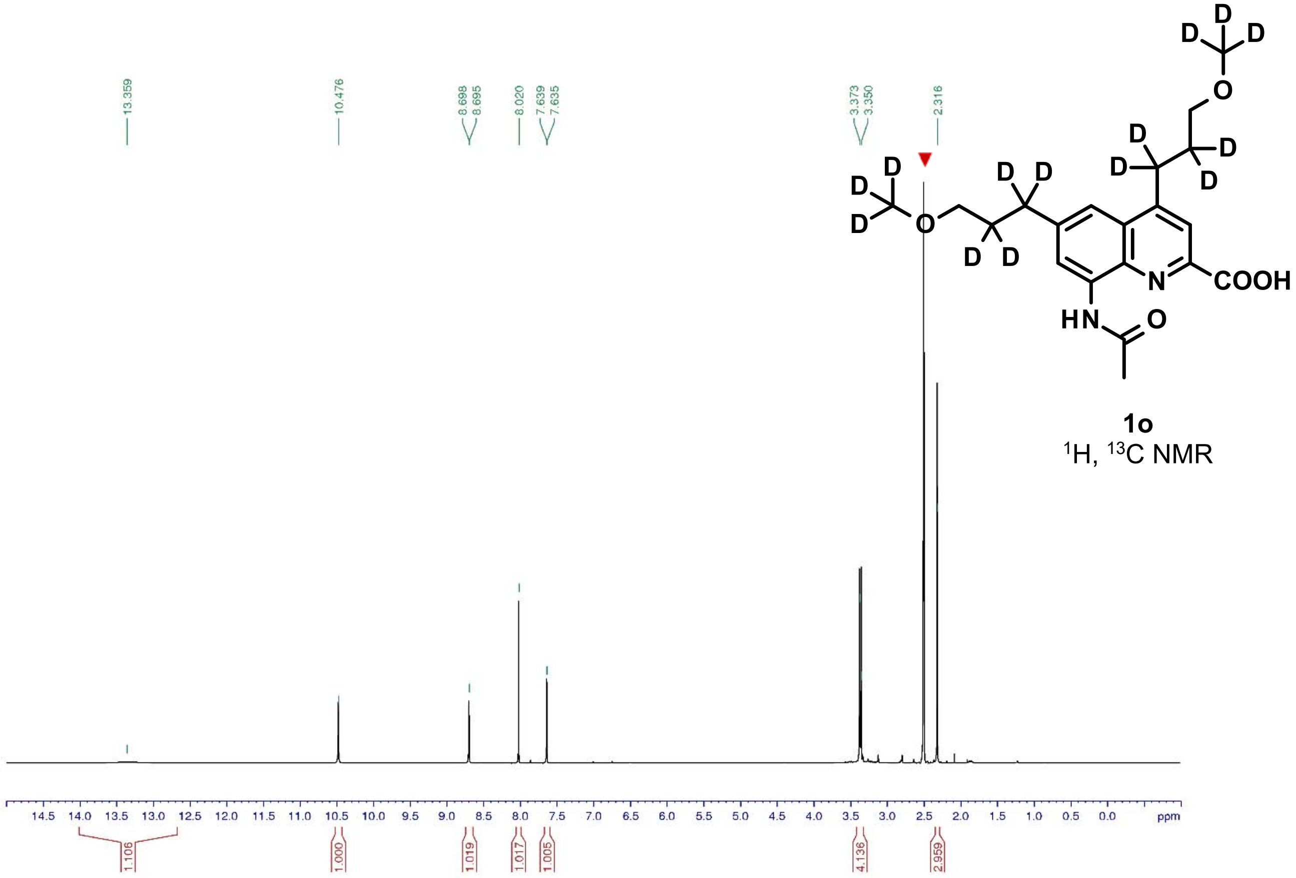
13.359
10.476
8.698
8.695
8.020
7.639
7.635
3.373
3.350
2.316
1.106
1.000
1.019
1.017
1.005
4.136
2.959
ppm
D
D
D
O
D
D
D
D
D
D
D
D
O
D
D
D
D
D
N
COOH
HN
O

1o

^{1}H, ^{13}C NMR

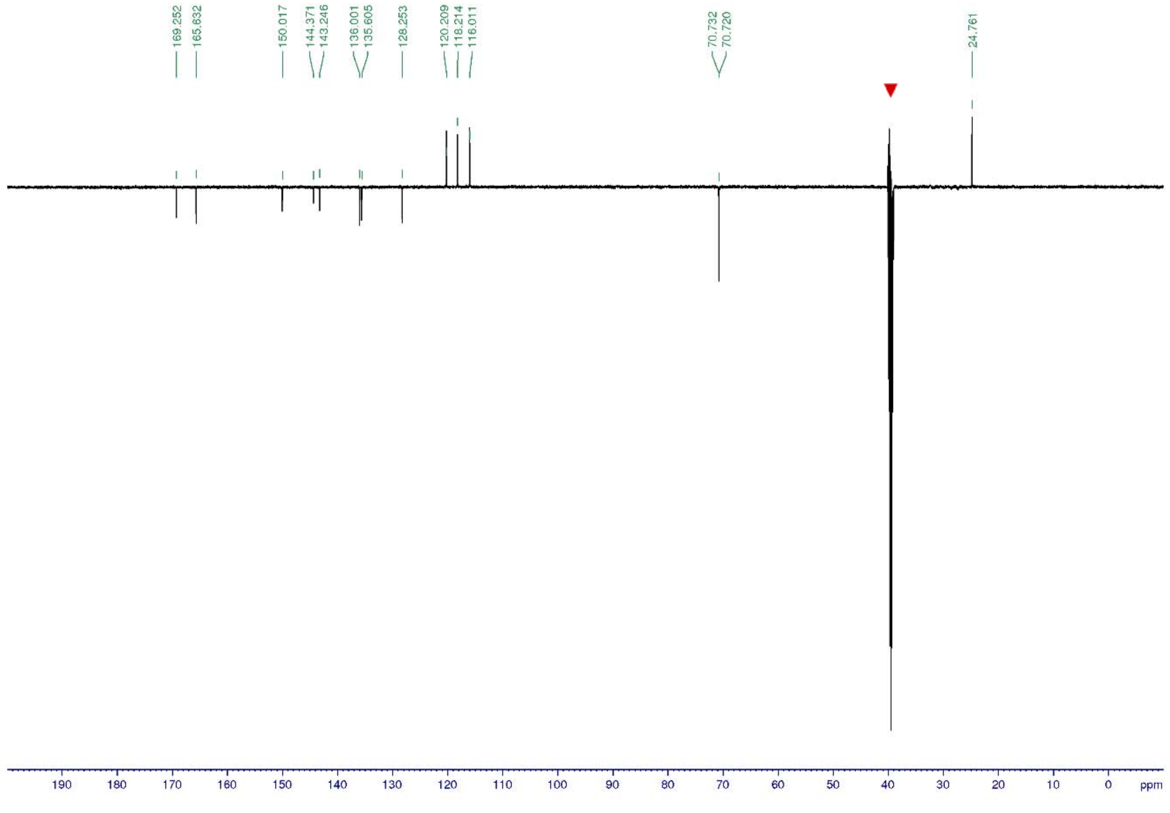
169.252
165.632
150.017
144.371
143.246
136.001
135.605
128.253
120.209
118.214
116.011
70.732
70.720
24.761
ppm

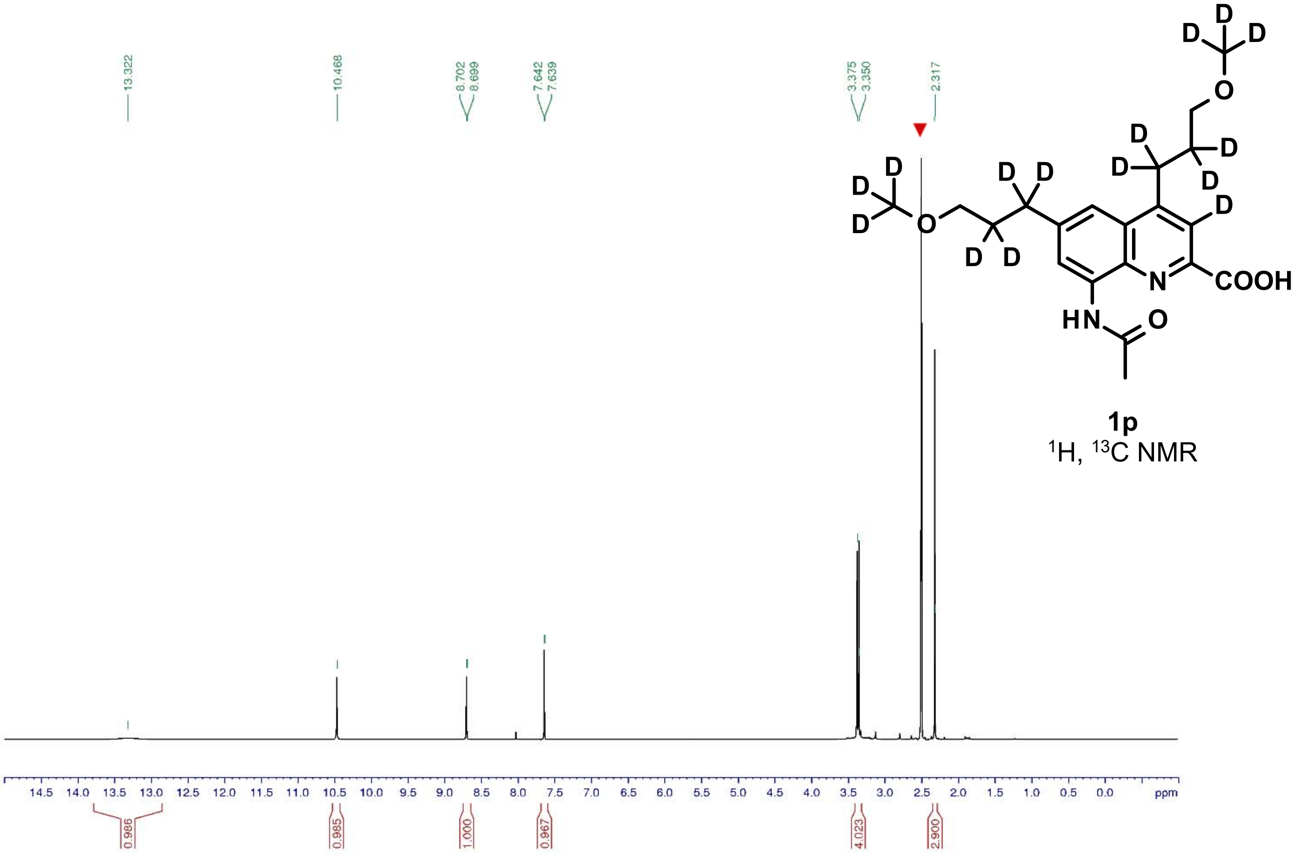

13.322
10.468
8.702
8.699
7.642
7.639
3.375
3.350
2.317
0.986
0.985
1.000
0.967
4.023
2.900
ppm
D
O
COOH
N
HN
O

1p

^{1}H, ^{13}C NMR

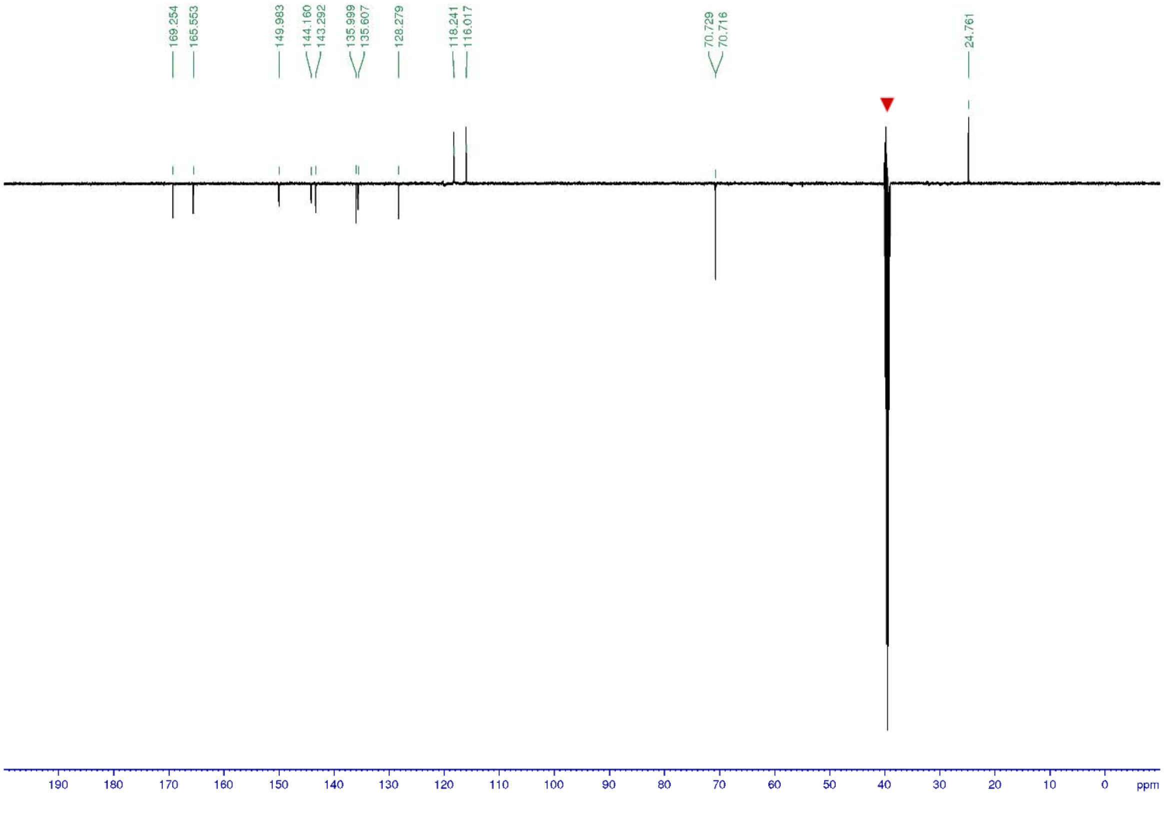

169.254
165.553
149.983
144.160
143.292
135.999
135.607
128.279
118.241
116.017
70.729
70.716
24.761
ppm

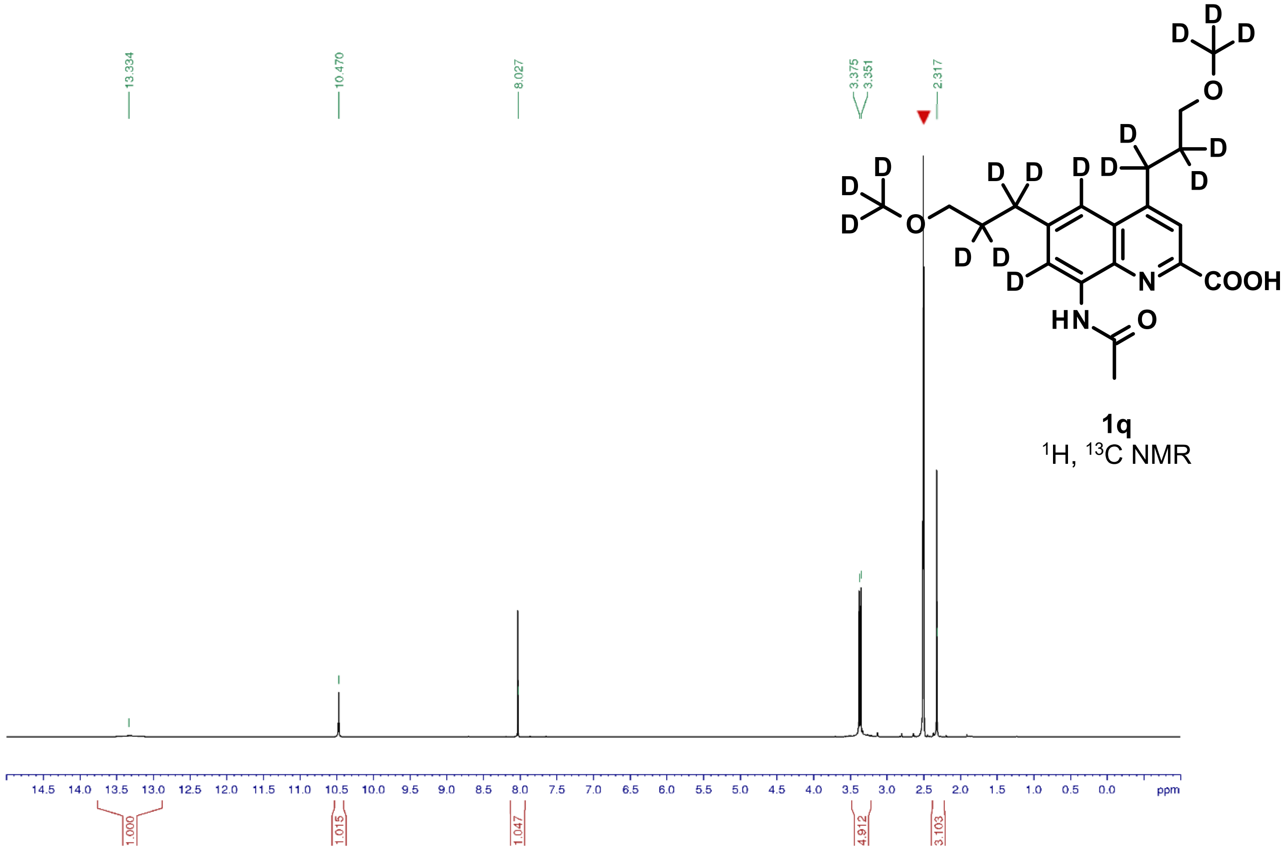
13.334
10.470
8.027
3.375
3.351
2.317
1.000
1.015
1.047
4.912
3.103
ppm
D
O
COOH
N
HN
O

1q
^{1}H, ^{13}C NMR

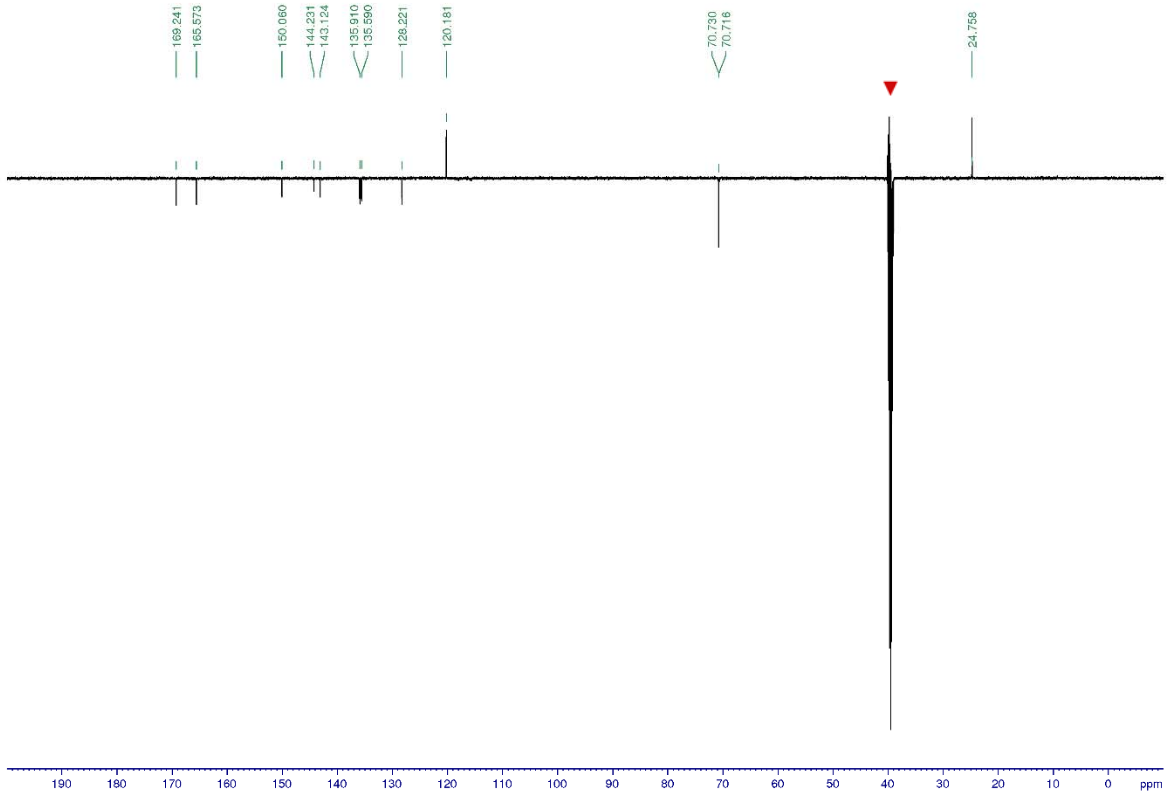
169.241
165.573
150.060
144.231
143.124
135.910
135.590
128.221
120.181
70.730
70.716
24.758
ppm

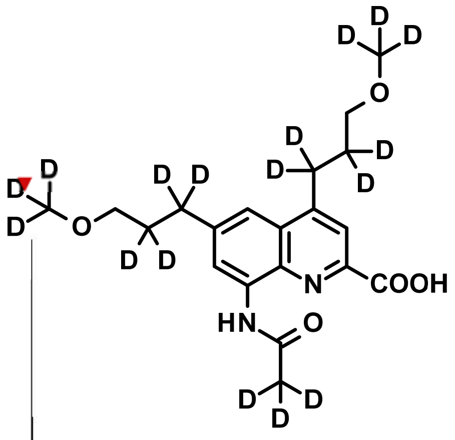

1r

^{1}H, ^{13}C NMR

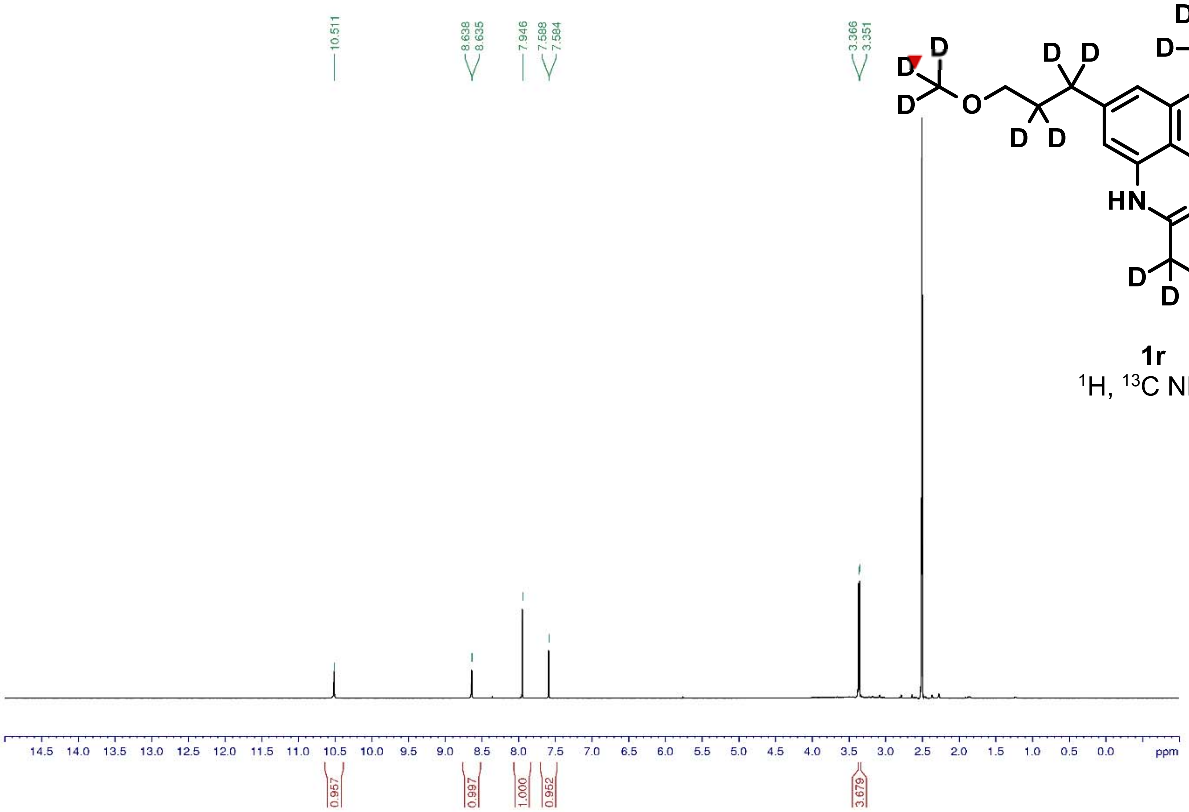

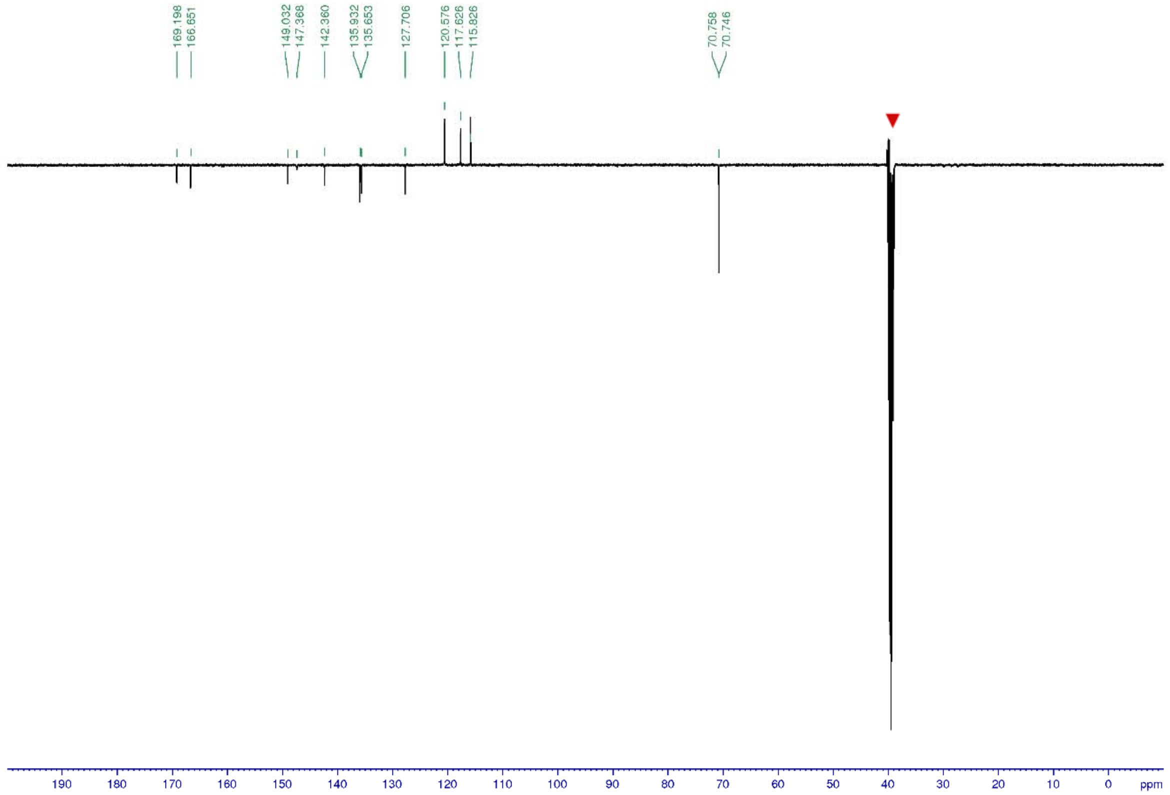

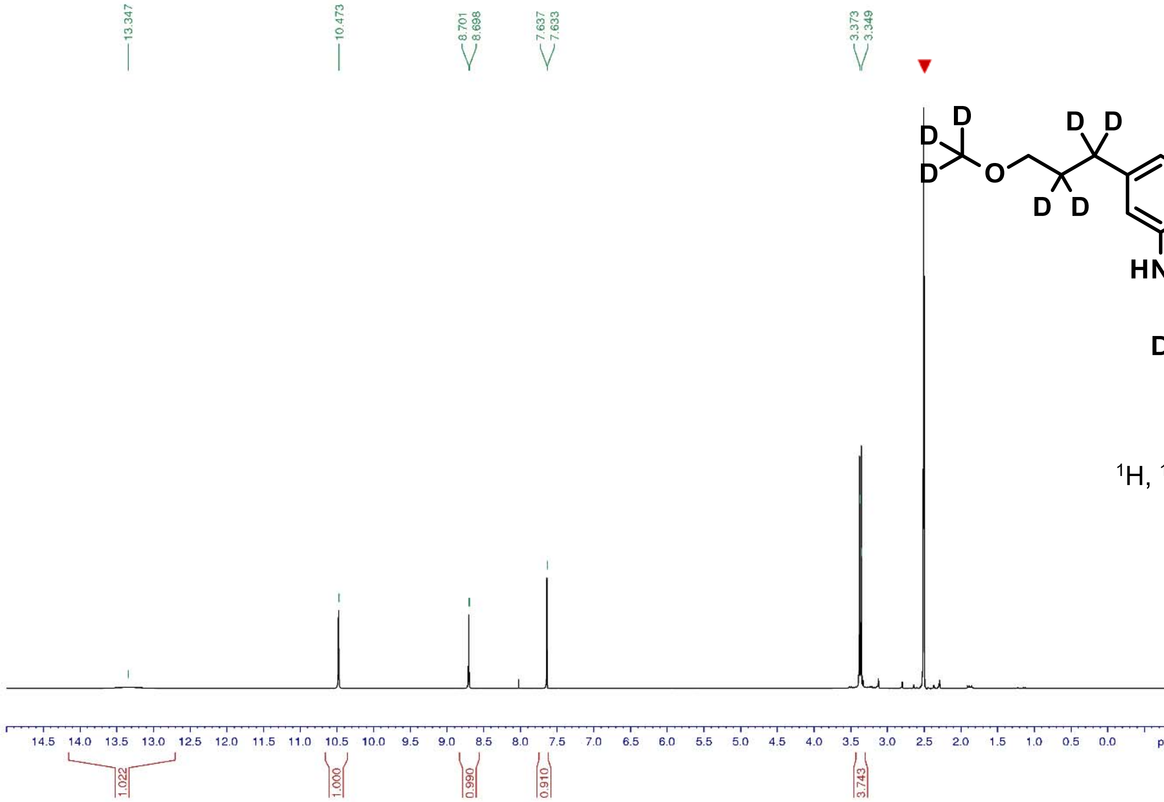

13.347
10.473
8.701
8.698
7.637
7.633
3.373
3.349
1.022
1.000
0.990
0.910
3.743
ppm

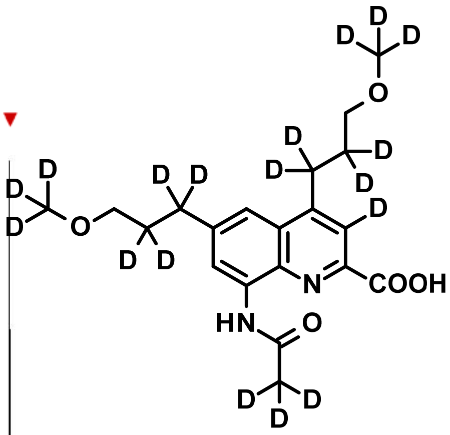

D
O
N
COOH
HN
O

1s

^{1}H, ^{13}C NMR

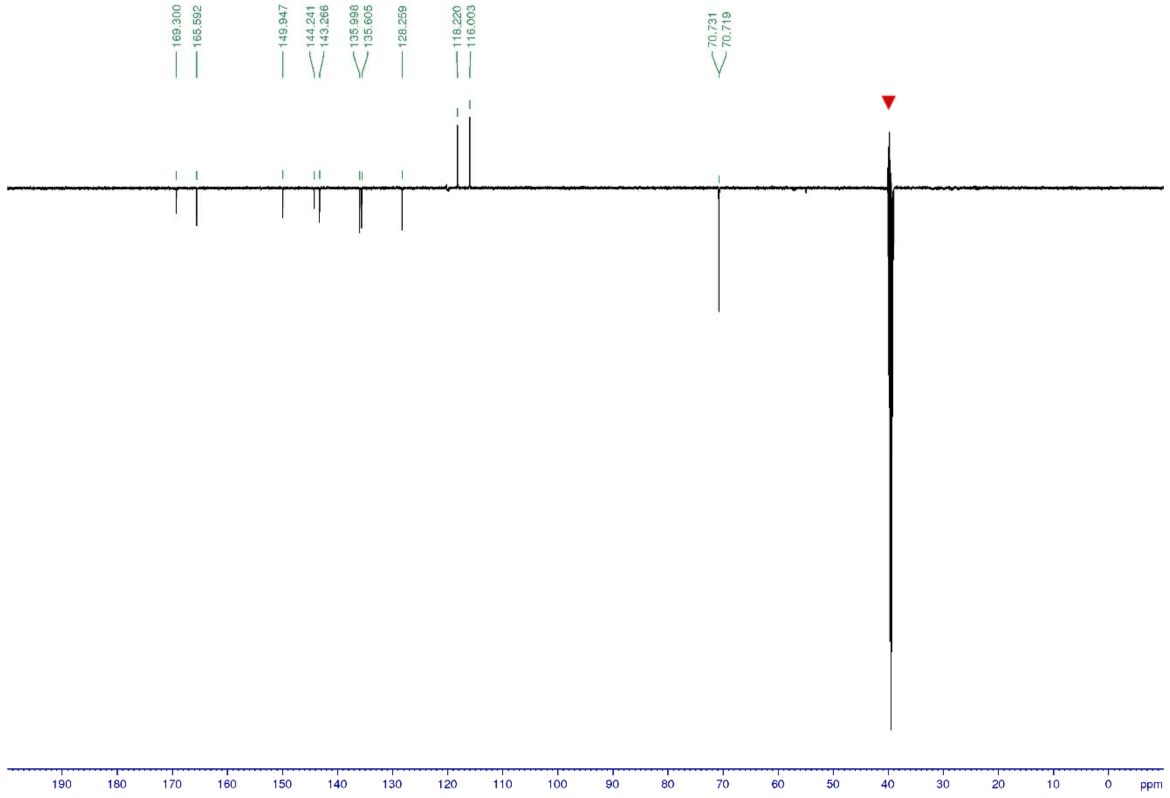

169.300
165.592
149.947
144.241
143.266
135.998
135.605
128.259
118.220
116.003
70.731
70.719
ppm

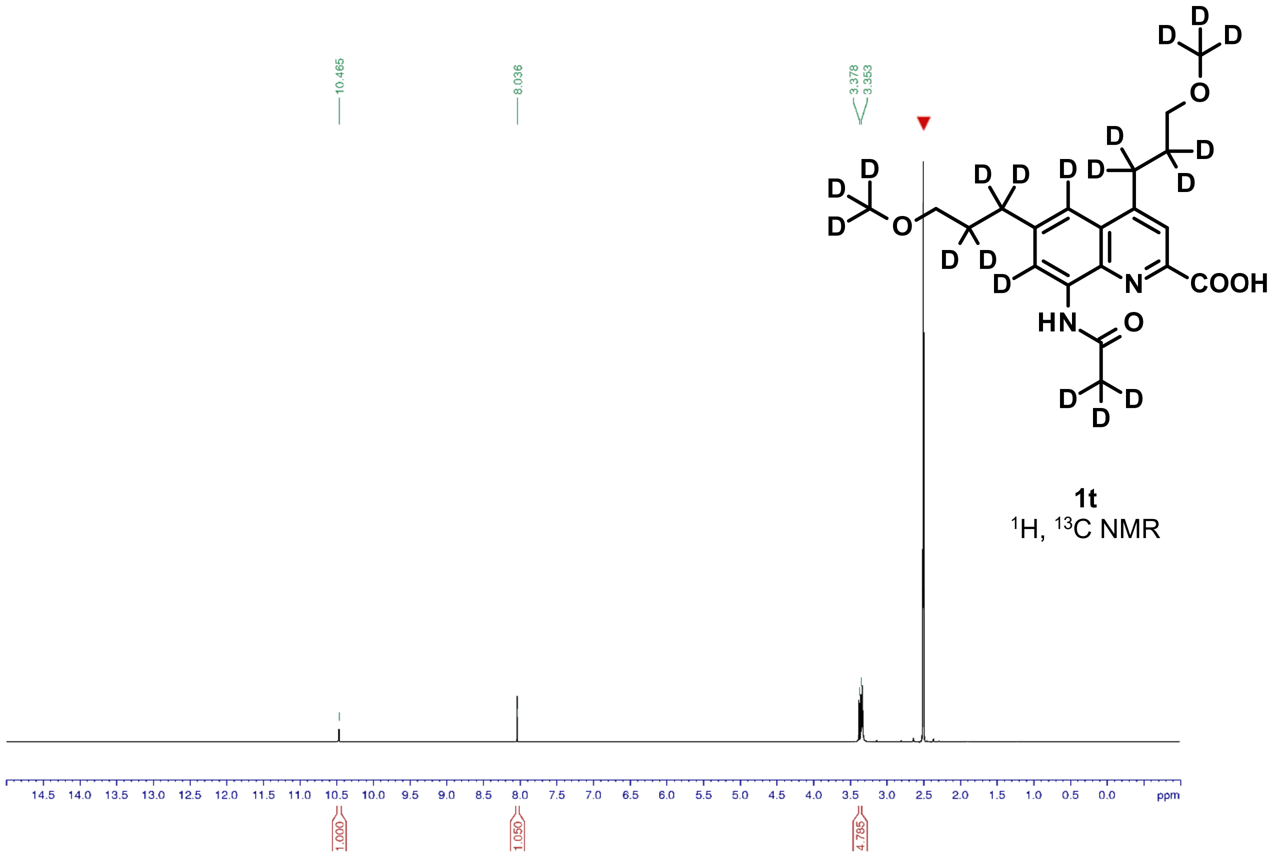

10.465
8.036
3.378
3.353
14.5 14.0 13.5 13.0 12.5 12.0 11.5 11.0 10.5 10.0 9.5 9.0 8.5 8.0 7.5 7.0 6.5 6.0 5.5 5.0 4.5 4.0 3.5 3.0 2.5 2.0 1.5 1.0 0.5 0.0 ppm
1.000
1.050
4.785
COOH
HN
O
N

1t
^1H, ^{13}C NMR

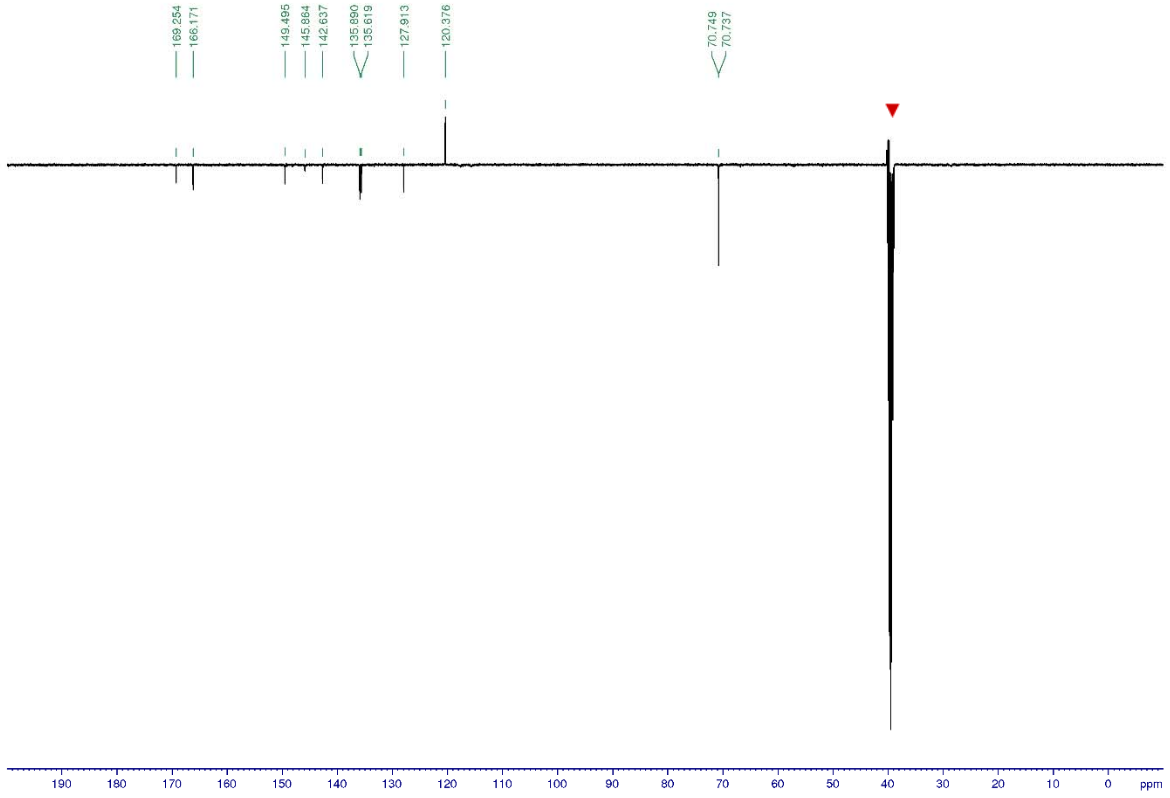

169.254
166.171
149.495
145.864
142.637
135.890
135.619
127.913
120.376
70.749
70.737
190 180 170 160 150 140 130 120 110 100 90 80 70 60 50 40 30 20 10 0 ppm

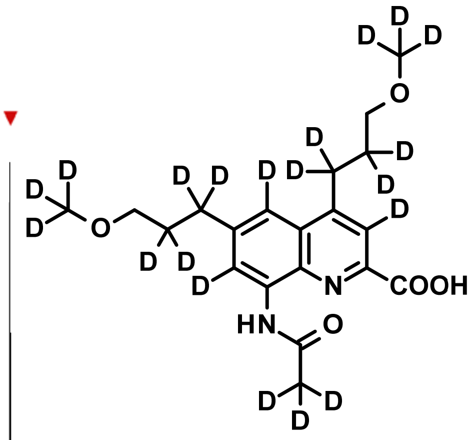

1u

^{1}H, ^{13}C NMR

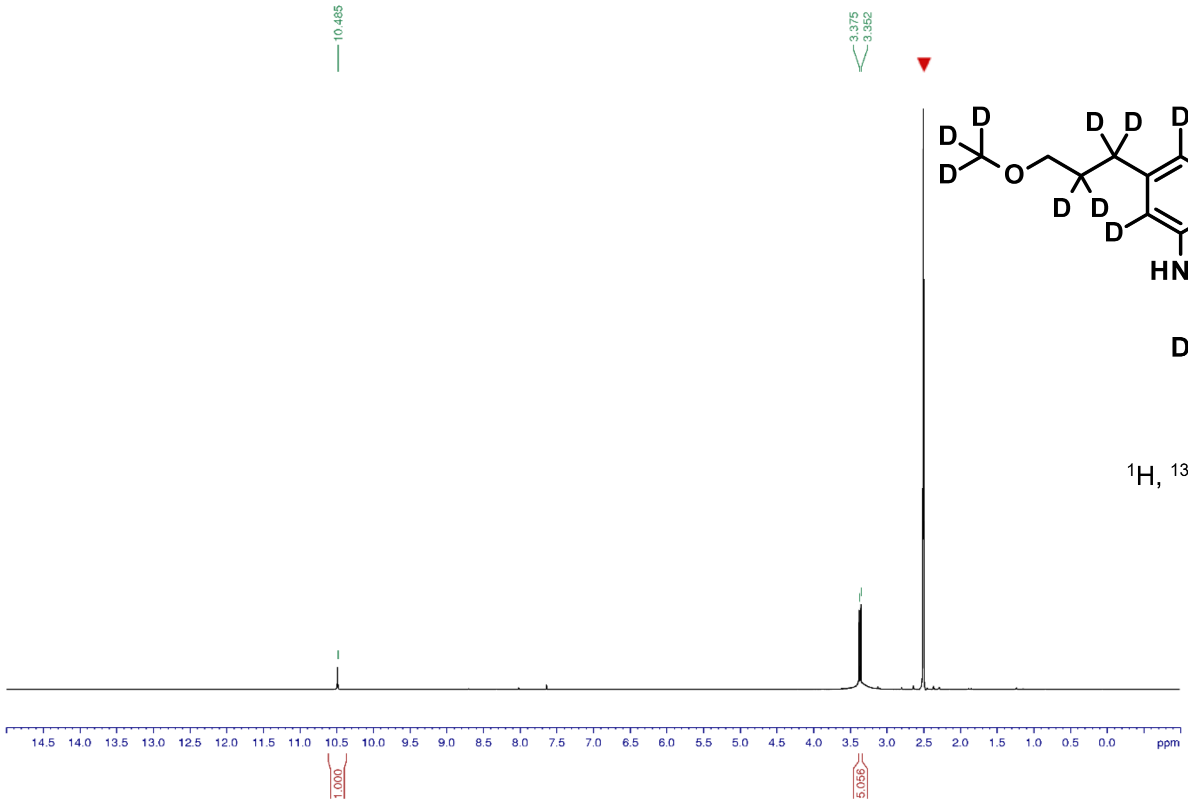

10.485
3.375
3.352
14.5 14.0 13.5 13.0 12.5 12.0 11.5 11.0 10.5 10.0 9.5 9.0 8.5 8.0 7.5 7.0 6.5 6.0 5.5 5.0 4.5 4.0 3.5 3.0 2.5 2.0 1.5 1.0 0.5 0.0 ppm
1.000
5.056

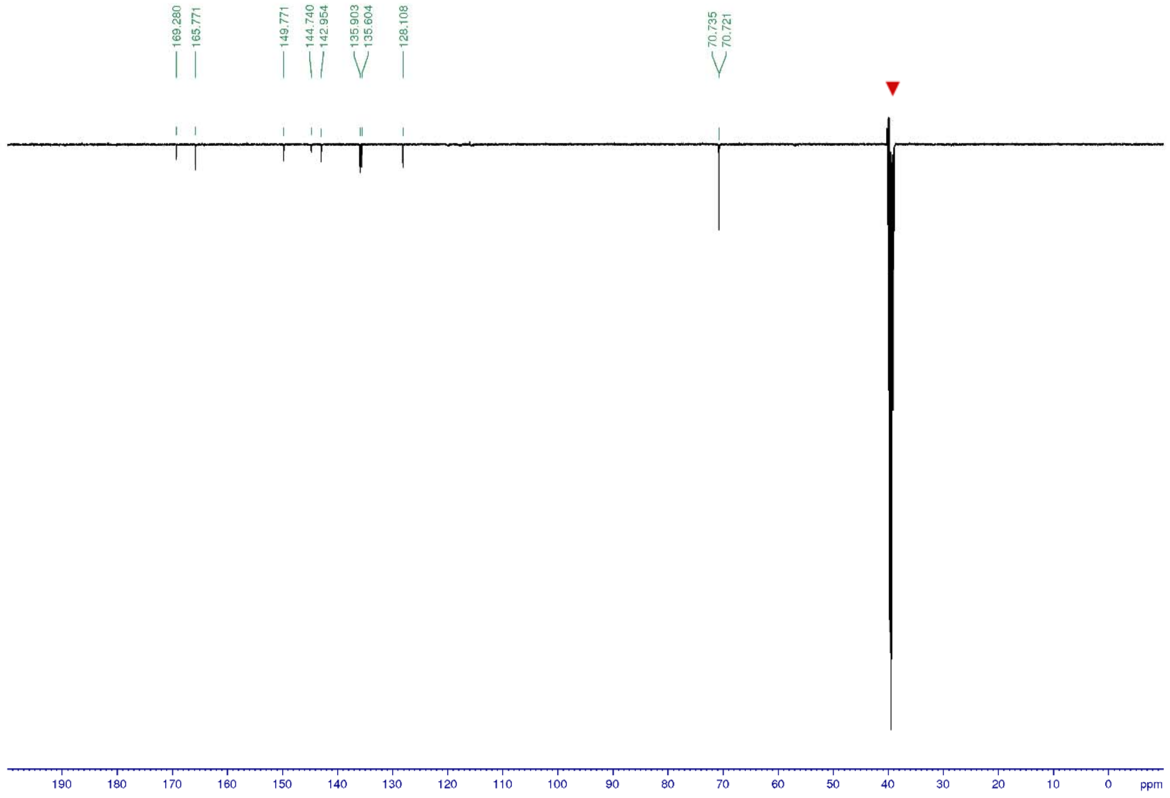

169.280
165.771
149.771
144.740
142.954
135.903
135.604
128.108
70.735
70.721
190 180 170 160 150 140 130 120 110 100 90 80 70 60 50 40 30 20 10 0 ppm

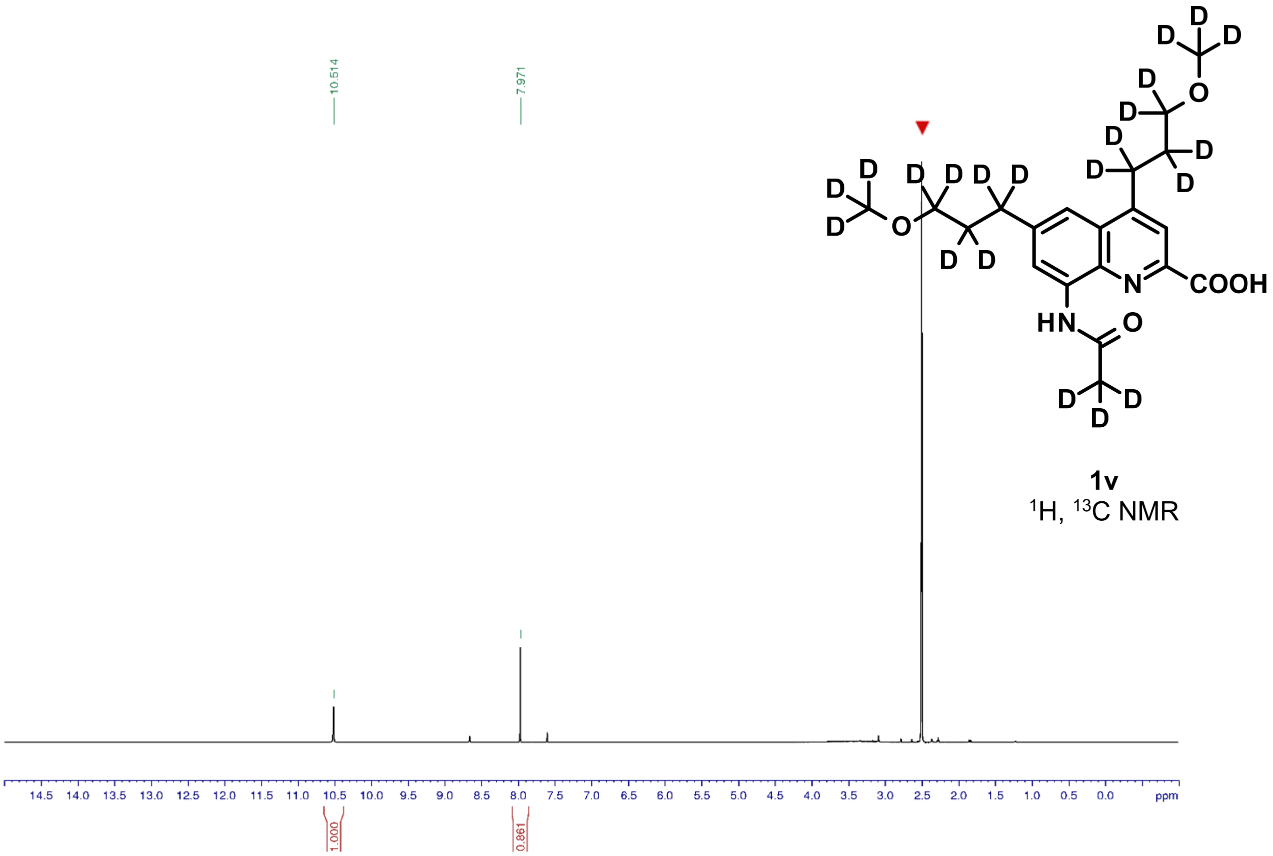
10.514
7.971
D
D
D
O
D
D
D
D
D
D
D
D
D
D
D
O
D
D
D
COOH
N
HN
O
D
D
D
14.5 14.0 13.5 13.0 12.5 12.0 11.5 11.0 10.5 10.0 9.5 9.0 8.5 8.0 7.5 7.0 6.5 6.0 5.5 5.0 4.5 4.0 3.5 3.0 2.5 2.0 1.5 1.0 0.5 0.0 ppm
1.000
0.861

1v
^{1}H, ^{13}C NMR

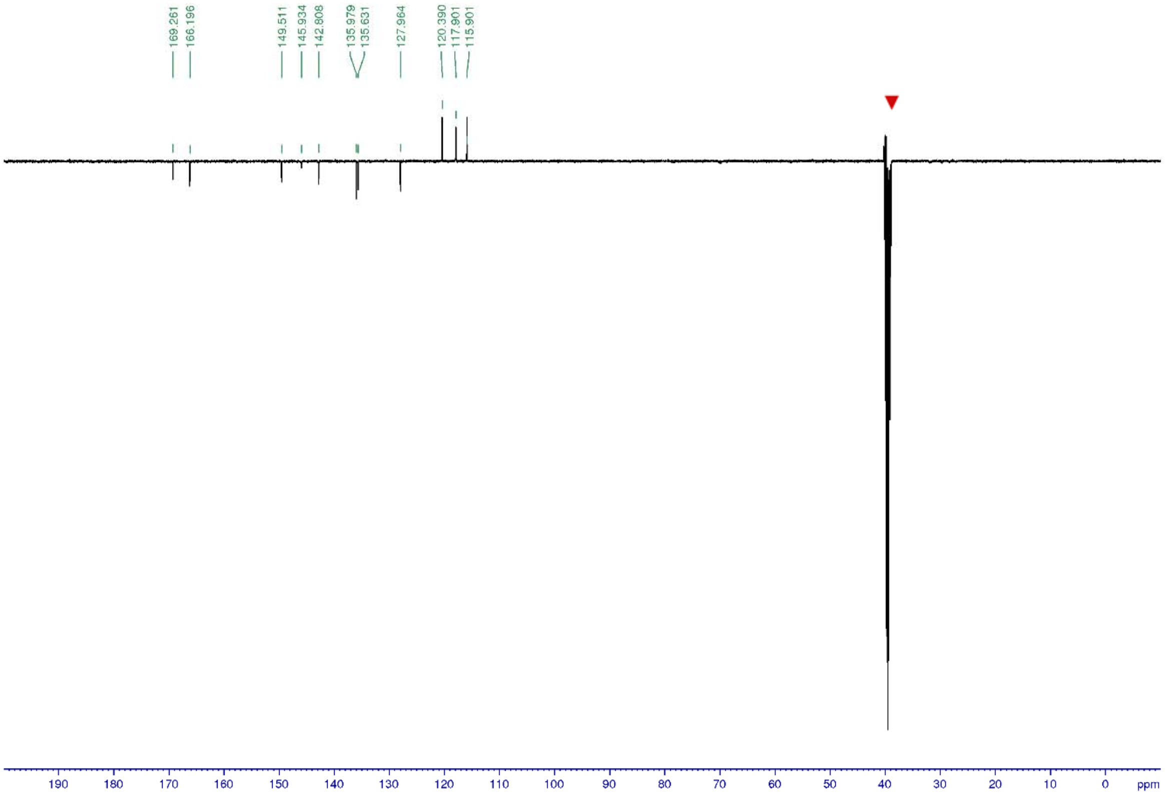
169.261
166.196
149.511
145.934
142.808
135.979
135.631
127.964
120.390
117.901
115.901
190 180 170 160 150 140 130 120 110 100 90 80 70 60 50 40 30 20 10 0 ppm

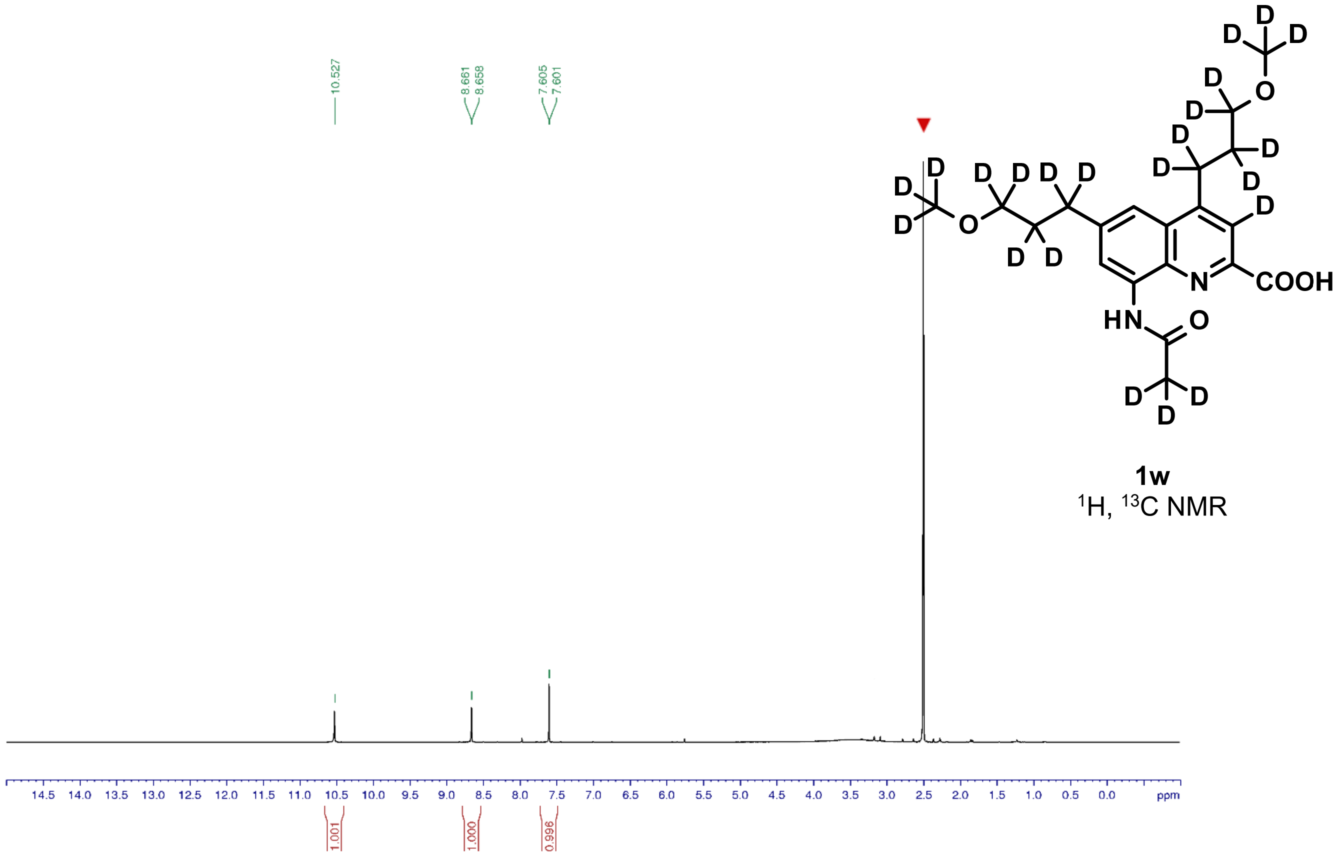

1w

^{1}H, ^{13}C NMR

10.527
8.661
8.658
7.605
7.601
14.5 14.0 13.5 13.0 12.5 12.0 11.5 11.0 10.5 10.0 9.5 9.0 8.5 8.0 7.5 7.0 6.5 6.0 5.5 5.0 4.5 4.0 3.5 3.0 2.5 2.0 1.5 1.0 0.5 0.0 ppm
1.001
1.000
0.996

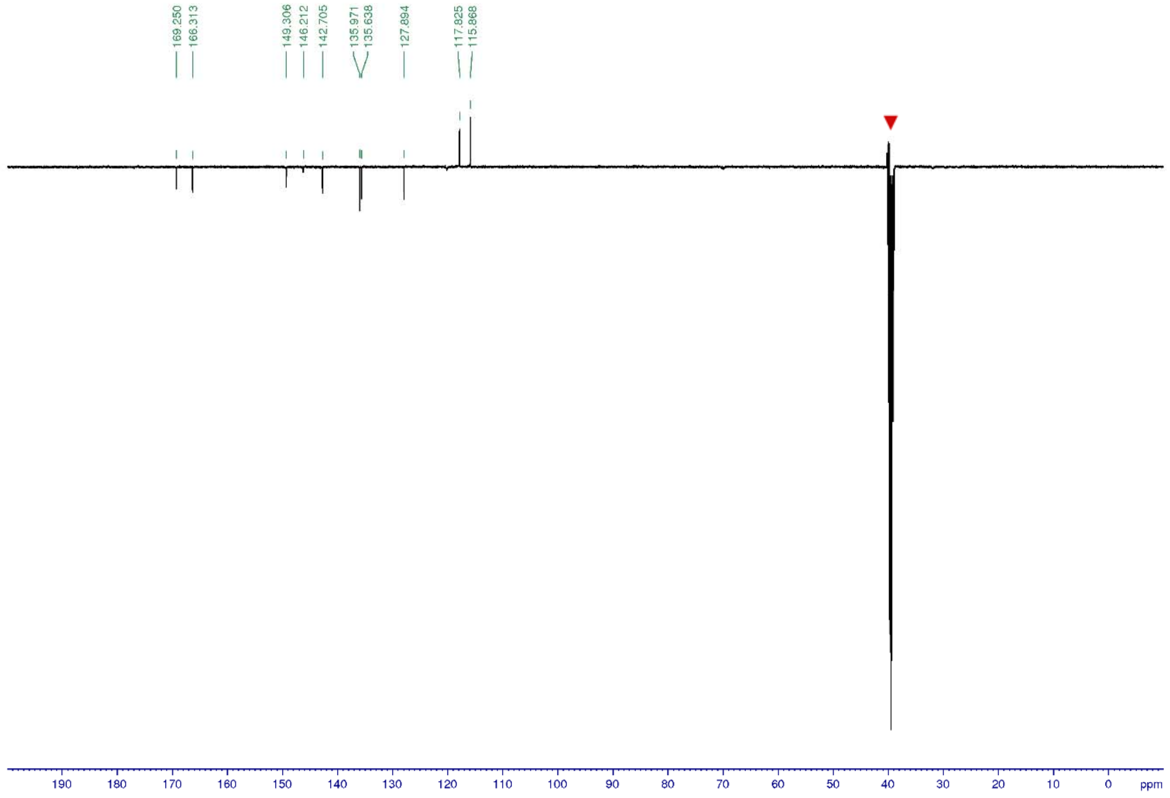

169.250
166.313
149.306
146.212
142.705
135.971
135.638
127.894
117.825
115.868
190 180 170 160 150 140 130 120 110 100 90 80 70 60 50 40 30 20 10 0 ppm

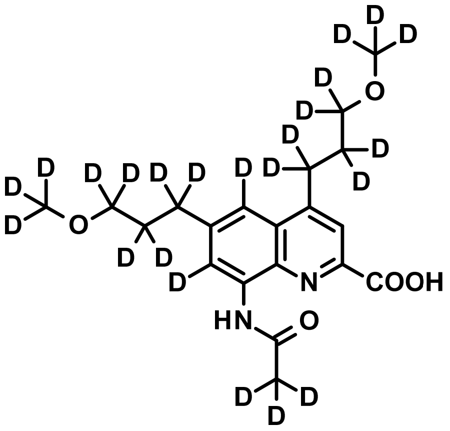

COOH
HN
O
N

1x
^{1}H, ^{13}C NMR

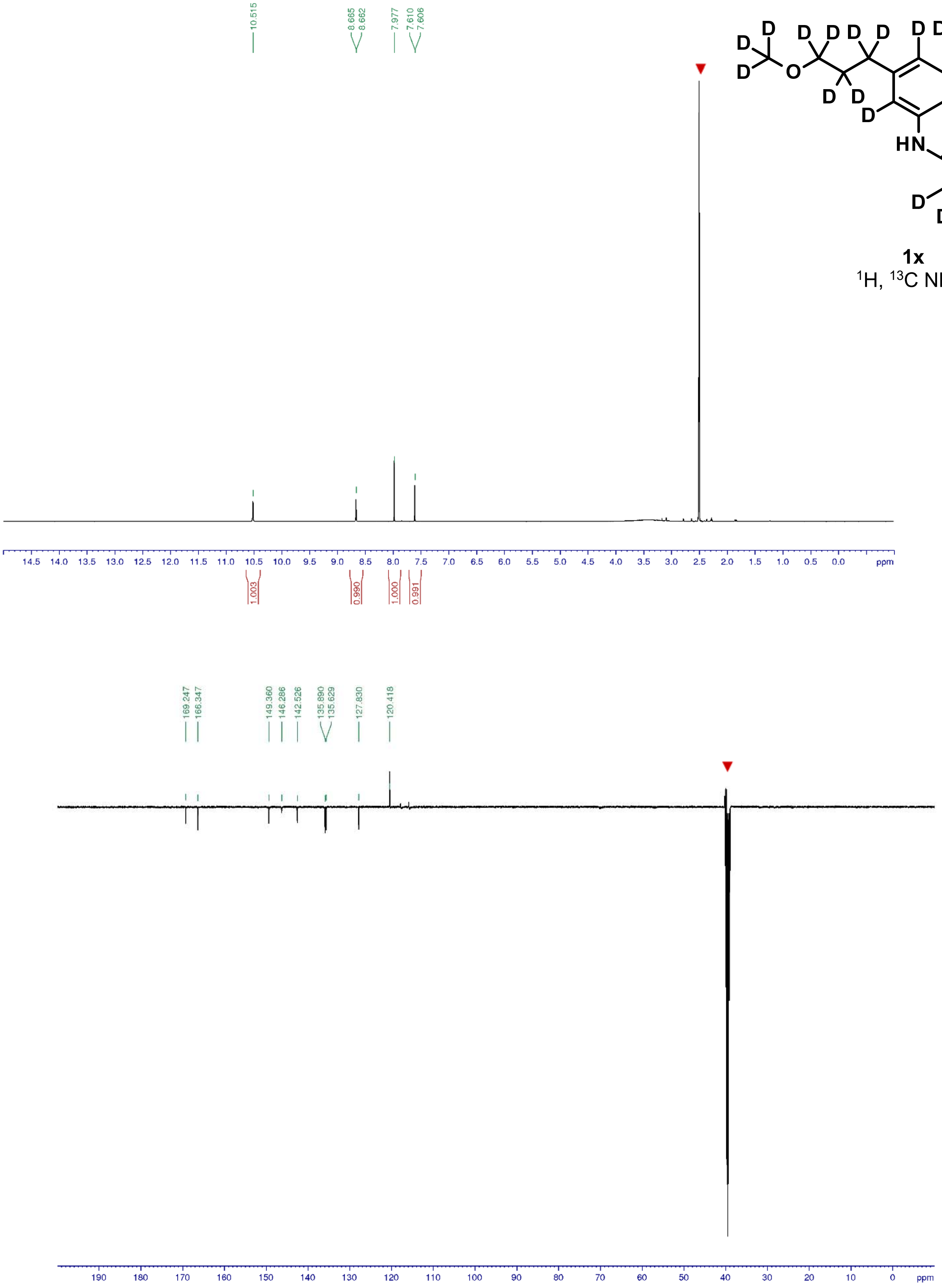

10.515
8.665
8.662
7.977
7.610
7.606
ppm
1.003
0.990
1.000
0.991
169.247
166.347
149.360
146.286
142.526
135.890
135.629
127.830
120.418
ppm

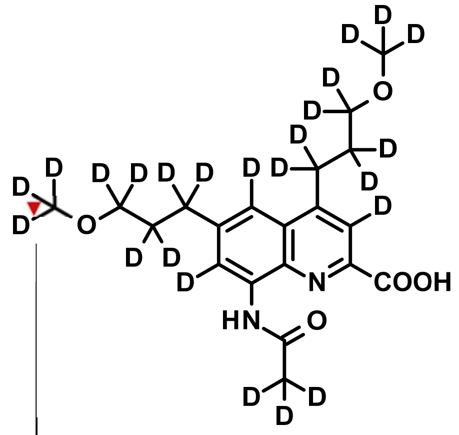

1y
^{1}H, ^{13}C NMR

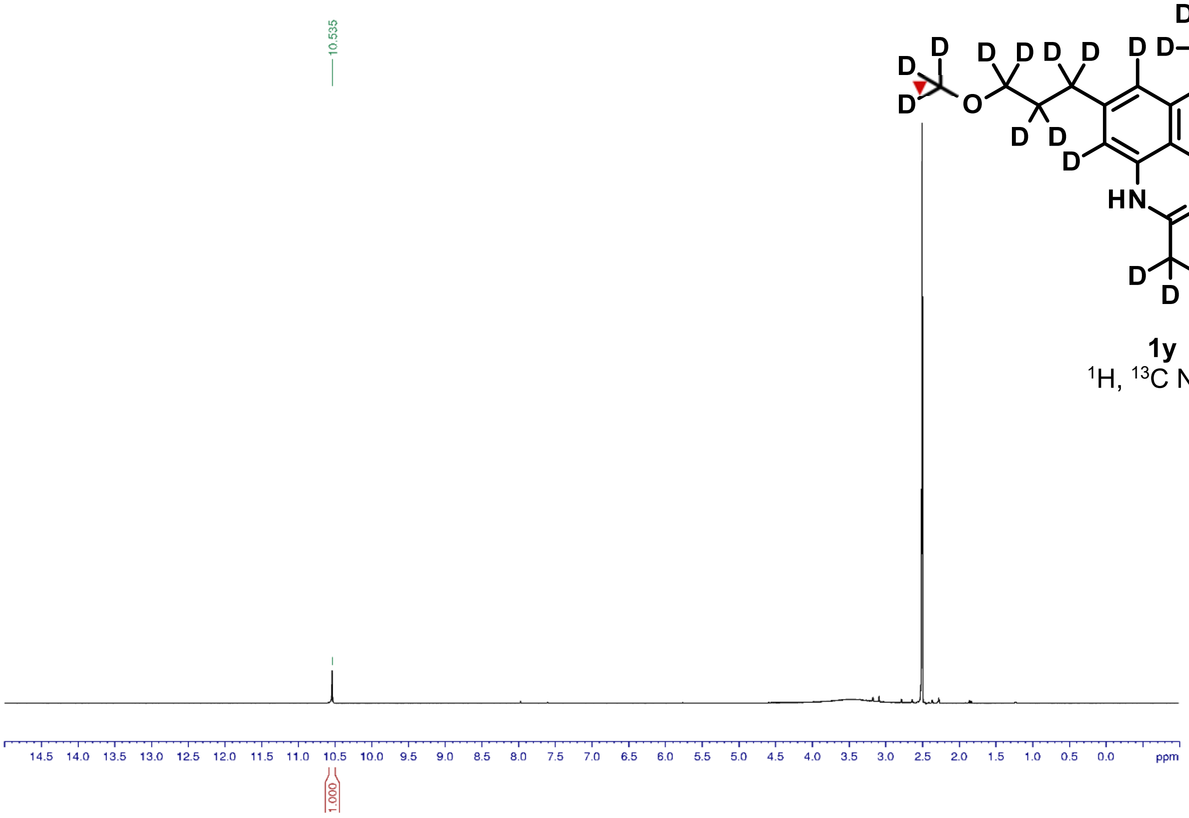
10.535
14.5 14.0 13.5 13.0 12.5 12.0 11.5 11.0 10.5 10.0 9.5 9.0 8.5 8.0 7.5 7.0 6.5 6.0 5.5 5.0 4.5 4.0 3.5 3.0 2.5 2.0 1.5 1.0 0.5 0.0 ppm
1.000

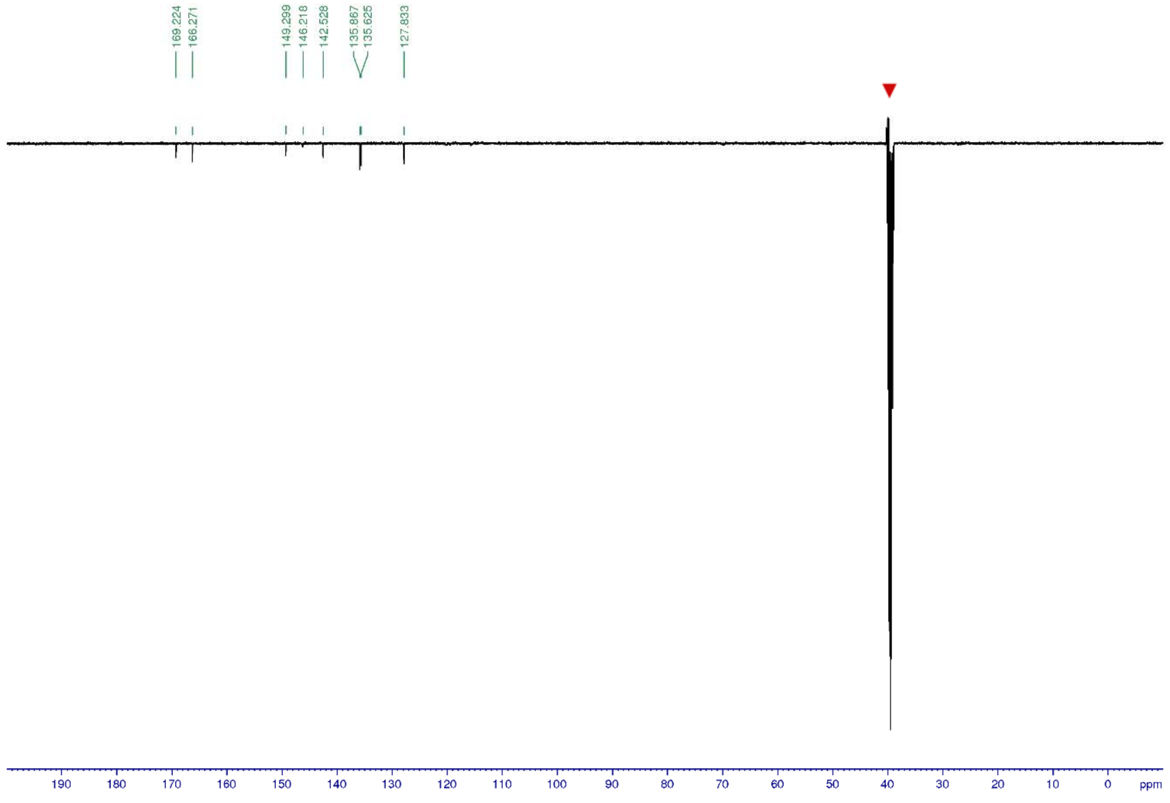
169.224
166.271
149.299
146.218
142.528
135.867
135.625
127.833
190 180 170 160 150 140 130 120 110 100 90 80 70 60 50 40 30 20 10 0 ppm

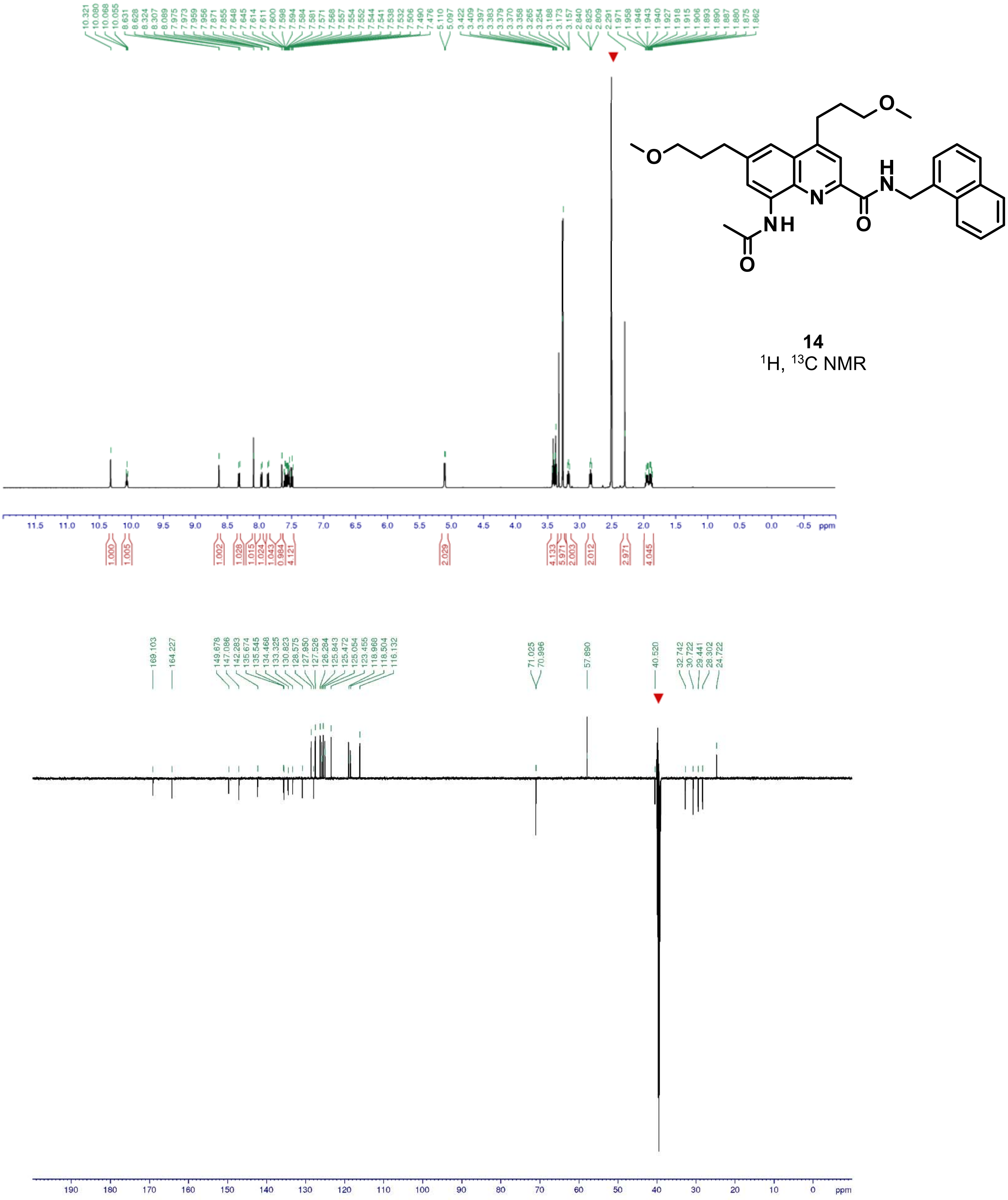

14
^{1}H, ^{13}C NMR

4.4 HRMS measurements and evaluation of the 1a-1y series

4.4.1 Conditions for HRMS measurements

To establish the optimal conditions for code reading by MS we prepared a dilution series of **1a** in acetonitrile, determined their signal range, and based on the results we settled on injecting 2 μL of 0.001 mg/mL solutions for measuring MS fingerprints. Approximatively 0.01 mg/mL stock solutions of **1a-y** were prepared in propionitrile with an accuracy of 1%. Mixtures were prepared by measuring the individual components (**1a-y**) into a vial using Sartorius Tacta mechanical 1-channel pipettes and the volume was adjusted to 1 mL using acetonitrile. To measure the MS fingerprints of the mixtures 2 μL aliquots were injected onto an Agilent 1290 Infinity II- Agilent 6545 LC-QTOF instrument having an InfinityLab Poroshell 120 SB-C18, 2.1 mm, 1.9 μm column. In case of the HRMS measurements of the **1a-y** components and all the mixtures prepared from them, 3 parallel measurements were carried out and the given peak areas and compositions are based on their average value and the presented HRMS spectra is picked from these three.

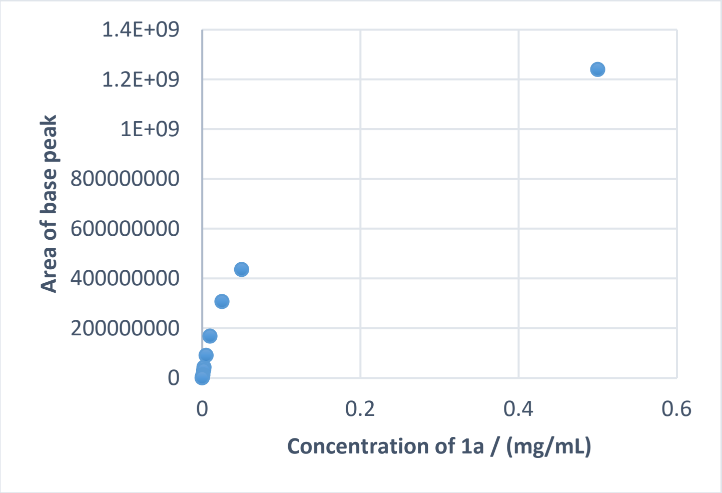

Scheme S1. Base peak areas of different concentrations of **1a** solutions.

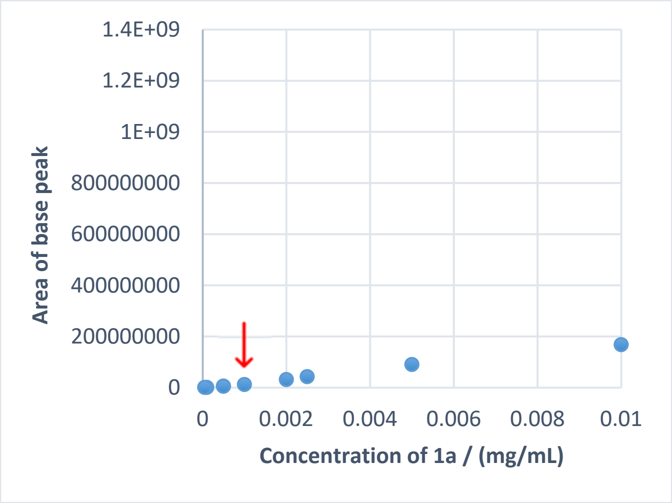

Scheme S2. Linearity range and the chosen concentration of **1a** based on the base peak areas of different concentrations.

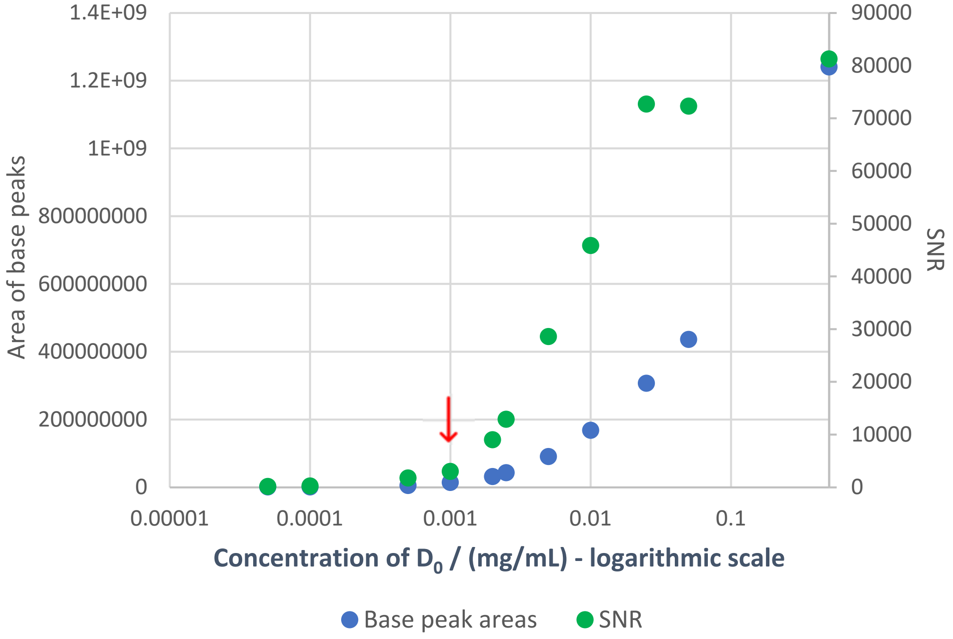

Scheme S3. Using the default **SNR calculation** algorithms of Agilent MassHunter Qualitative Analysis Navigator B.08.00 software, S/N ratios for the peaks at m/z=375 were calculated and plotted for all test concentrations, **S/N = 2978** for the chosen concentration.

The **mass spectrometric resolution** for component **1a** was determined by Agilent MassHunter Qualitative Analysis Navigator B.08.00 software as following: R=FWHM(m)/m=375.1919/0.01089=34442, where FWHM is the full width at half-maximum of the peak at m/z=375.

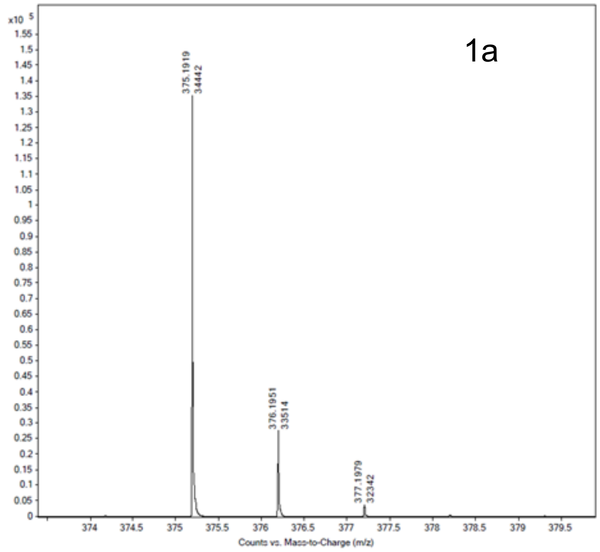

Scheme S4. The mass spectrum and mass spectrometric resolution of component **1a**.

4.4.2 HRMS measurements of **1a-1y**

Table S4. Peak areas of [M+H]$^+$ masses of 1a-1n in HRMS spectra.

	**375**	**376**	**377**	**378**	**379**	**380**	**381**	**382**	**383**	**384**	**385**	**386**	**387**	**387**	**388**	**389**	**390**	**391**	**392**	**393**	**394**	**395**	**396**	**397**	**398**	**399**	**400**	**401**	**402**	**403**
**1a**	12183502	2588672	379695	54738	8815	0	0	0	0	0	0	0	0	0	0	0	0	0	0	0	0	0	0	0	0	0	0	0	0	0
**1b**	308270	7715764	1525722	230616	28477	11426	2425	0	0	0	0	0	0	0	0	0	0	0	0	0	0	0	0	0	0	0	0	0	0	0
**1c**	34430	316839	6001977	1521312	351269	35050	7577	0	0	0	0	0	0	0	0	0	0	0	0	0	0	0	0	0	0	0	0	0	0	0
**1d**	33481	105411	1194206	6544837	1446278	229395	34733	7281	0	0	0	0	0	0	0	0	0	0	0	0	0	0	0	0	0	0	0	0	0	0
**1d***	44561	482690	3117322	7282973	1453339	225659	35433	0	0	0	0	0	0	0	0	0	0	0	0	0	0	0	0	0	0	0	0	0	0	0
**1e**	18981	31548	153234	1442444	6355013	1279306	195240	30965	13717	16985	2913	0	0	0	0	0	0	0	0	0	0	0	0	0	0	0	0	0	0	0
**1f**	5755	2498	18678	131811	1254782	6388419	1560822	258997	39284	6729	0	0	0	0	0	0	0	0	0	0	0	0	0	0	0	0	0	0	0	0
**1g**	0	0	0	0	8791	57806	7063192	1414392	214811	25394	4146	3329	0	0	0	0	0	0	0	0	0	0	0	0	0	0	0	0	0	0
**1h**	12052	50134	21178	11312	12375	16253	370214	8715353	1734241	255292	40383	10715	3531	0	0	0	0	0	0	0	0	0	0	0	0	0	0	0	0	0
**1i**	0	0	14150	4337	1676	11009	22017	256851	7875917	1951934	307488	46776	11604	0	0	0	0	0	0	0	0	0	0	0	0	0	0	0	0	0
**1i***	0	0	0	73268	128579	346478	3234491	9898324	30335310	10380938	2242771	413956	60728	28485	0	0	0	0	0	0	0	0	0	0	0	0	0	0	0	0
**1j**	6352	12613	0	8949	13045	10069	26722	232121	1935038	7572049	1506356	234308	36114	10350	0	0	0	0	0	0	0	0	0	0	0	0	0	0	0	0
**1k**	0	0	0	0	0	8736	7040	48565	442214	2719791	7833517	1523571	232052	33304	15247	9077	0	0	0	0	0	0	0	0	0	0	0	0	0	0
**1l**	0	0	0	0	0	0	6727	16995	90247	580927	3169468	7715336	1816218	305261	46102	9895	1991	0	0	0	0	0	0	0	0	0	0	0	0	0
**1m**	0	0	0	0	0	9109	10873	21644	72918	311911	1309867	3999031	6360185	1792287	386758	75725	22801	9467	7499	0	0	0	0	0	0	0	0	0	0	0
**1n**	10007	11012	7733	21430	14859	11620	4328	14864	31741	48226	265272	1242951	3816624	6004982	1577038	315666	54758	12006	4199	0	0	0	0	0	0	0	0	0	0	0

Table S5. Peak areas of [M+H]$^+$ masses of 1o-1y in HRMS spectra.

	375	376	377	378	379	380	381	382	383	384	385	386	387	387	388	389	390	391	392	393	394	395	396	397	398	399	400	401	402	403
**1o**	0	0	0	0	0	0	0	1928	11631	17257	36559	189677	388961	951709	4080967	1203976	267413	51971	14109	0	0	0	0	0	0	0	0	0	0	0
**1p**	0	0	0	0	0	0	0	1795	3918	4081	5020	16456	60685	331856	1268097	3951129	1363593	383484	90638	27959	9308	0	0	0	0	0	0	0	0	0
**1q**	0	0	0	0	0	0	0	0	0	2330	1022	8228	13046	17259	111980	827362	3988013	1198235	288722	56517	10380	0	0	0	0	0	0	0	0	0
**1r**	0	0	0	0	0	0	1349	6390	1583	10982	12565	16995	22111	46865	136569	700774	2678874	6020256	1656637	347330	68322	21654	5059	0	0	0	0	0	0	0
**1s**	0	0	0	0	0	0	0	0	0	0	585	5051	3019	4834	21725	88018	404140	1332696	2952178	1016348	275663	65218	18968	9553	0	0	0	0	0	0
**1t**	0	0	0	0	0	0	0	10473	9636	17343	3195	11980	5207	11713	16688	33243	106463	586927	2902354	9184089	2436117	505988	84207	23612	9457	0	0	0	0	0
**1u**	0	0	0	0	0	0	3663	7769	16981	13666	4918	12332	7436	0	6263	28898	61053	158591	776037	3203584	7168041	1750129	327384	60534	18510	15082	5820	0	0	0
**1v**	0	0	0	0	0	0	0	0	0	0	0	0	0	0	15893	24984	72936	154894	286059	1212018	4153256	7408865	1663991	283901	45870	18282	11776	5431	0	0
**1w**	0	0	0	0	0	0	0	0	0	0	0	0	9224	0	0	6225	6107	15024	30960	152165	853176	3853787	9264283	2054228	339112	55594	14794	4833	0	0
**1x**	0	0	0	0	0	0	0	0	0	0	0	0	2560	0	1667	2924	7724	11452	16576	45204	215692	1153470	4355993	7830804	1575881	252986	37684	9034	5985	0
**1y**	0	0	0	0	0	0	0	0	0	0	0	0	0	0	0	0	0	0	0	7317	34476	156368	816646	3264754	6747591	1336144	205007	32433	12609	3246

Figure S2. HRMS of compounds **1b-z**

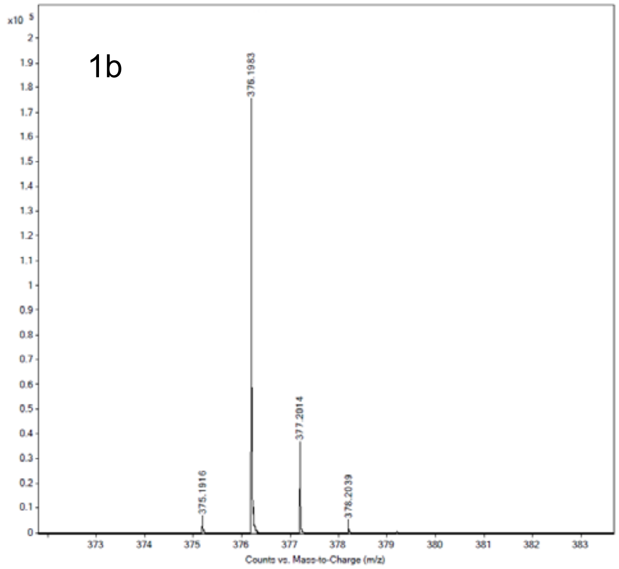

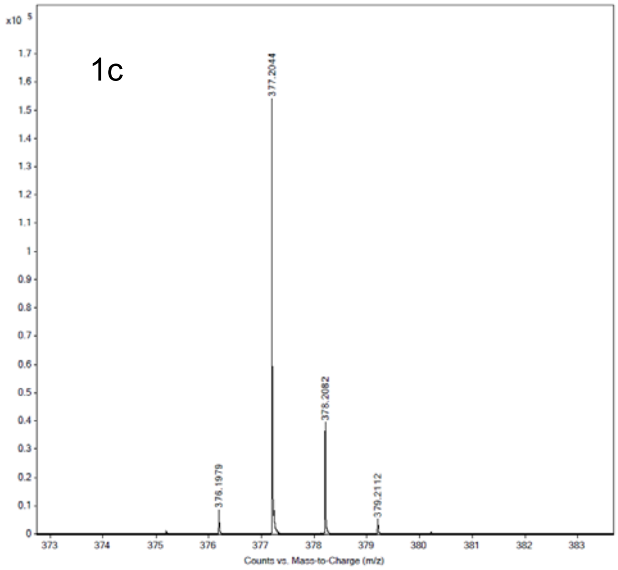

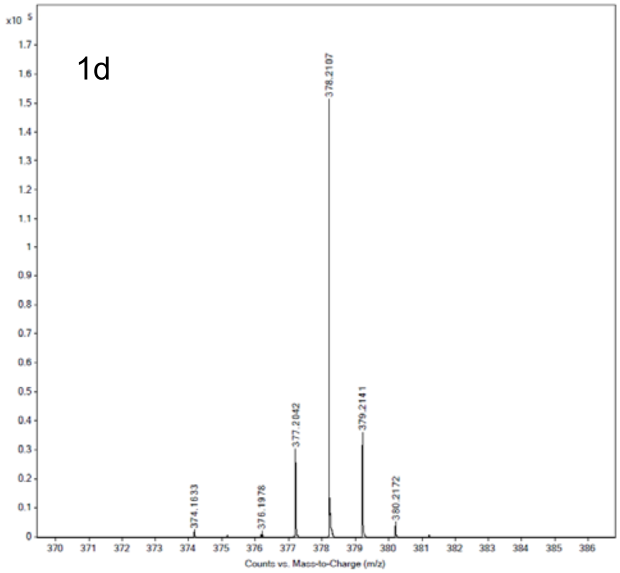

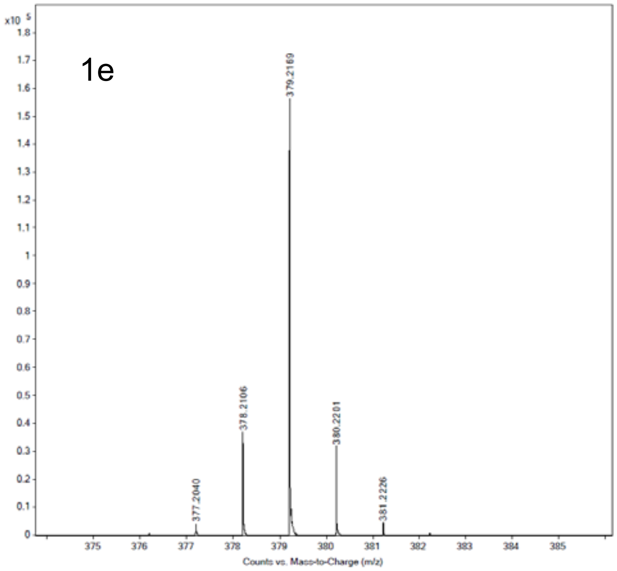

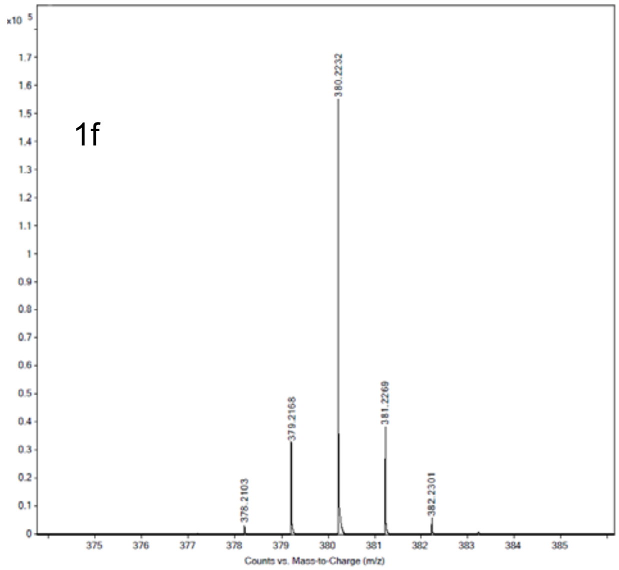

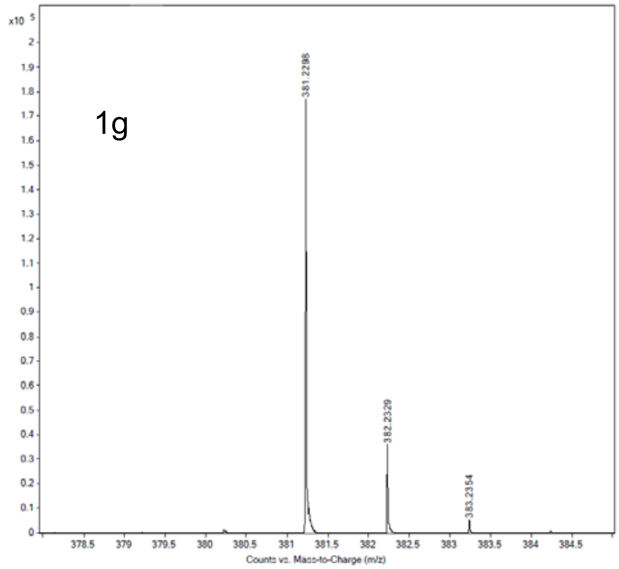

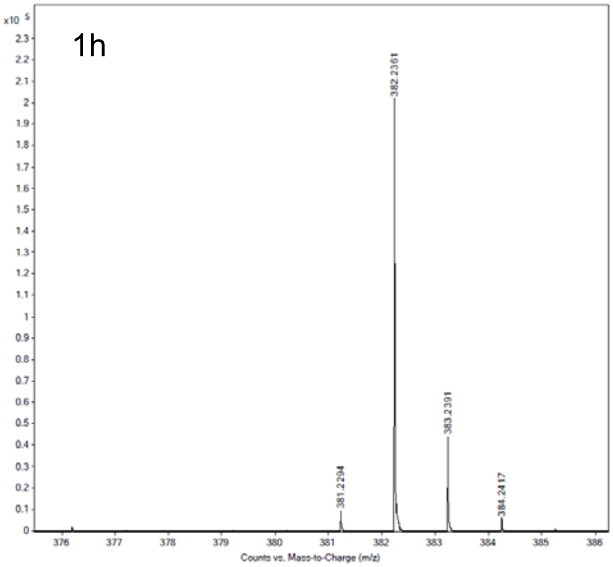
1h
x10 5
382.2361
383.2391
381.2294
384.2417
Counts vs. Mass-to-Charge (m/z)

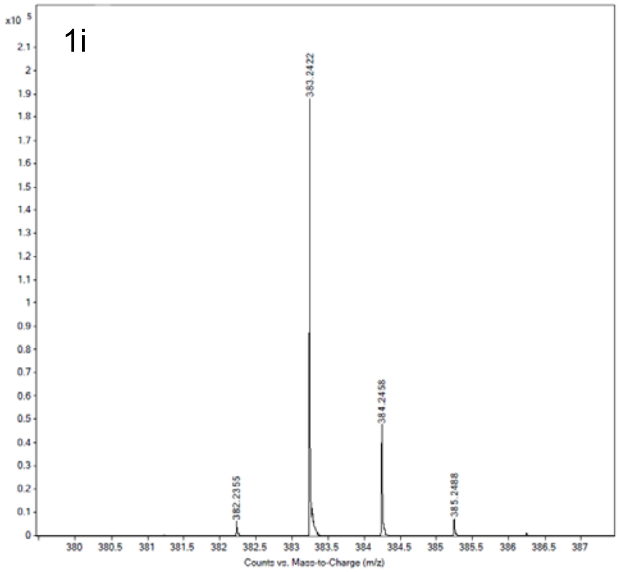
1i
x10 5
383.2422
384.2458
382.2355
385.2488
Counts vs. Mass-to-Charge (m/z)

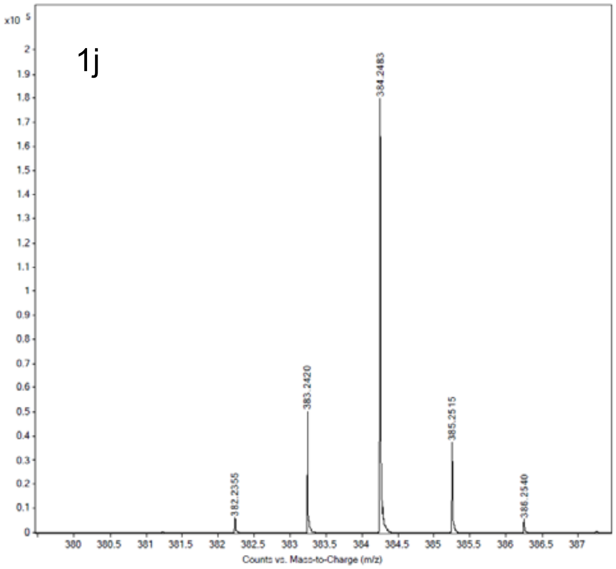
1j
x10 5
384.2483
383.2420
385.2515
382.2355
386.2540
Counts vs. Mass-to-Charge (m/z)

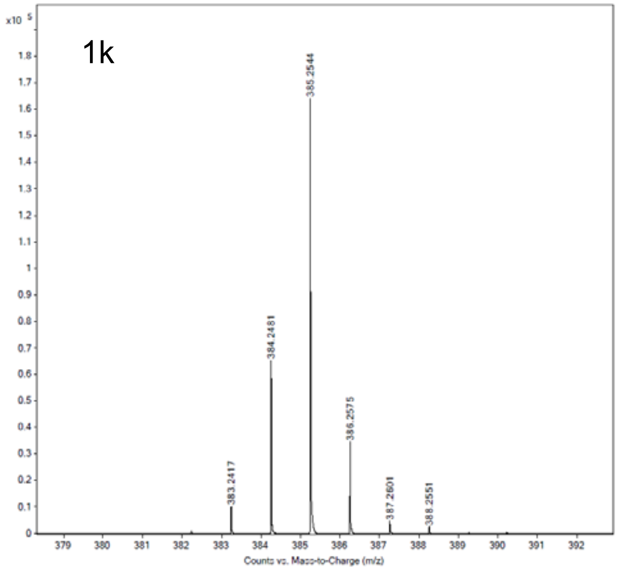
1k
x10 5
385.2544
384.2481
386.2575
383.2417
387.2601
388.2551
Counts vs. Mass-to-Charge (m/z)

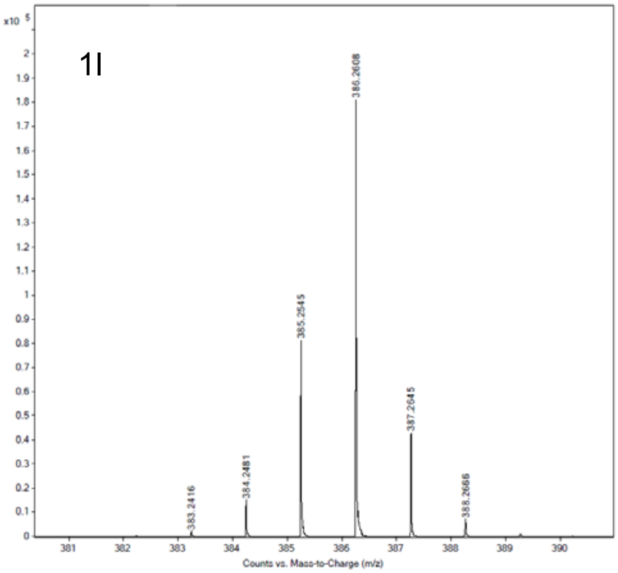
1l
x10 5
386.2608
385.2545
387.2645
384.2481
383.2416
388.2666
Counts vs. Mass-to-Charge (m/z)

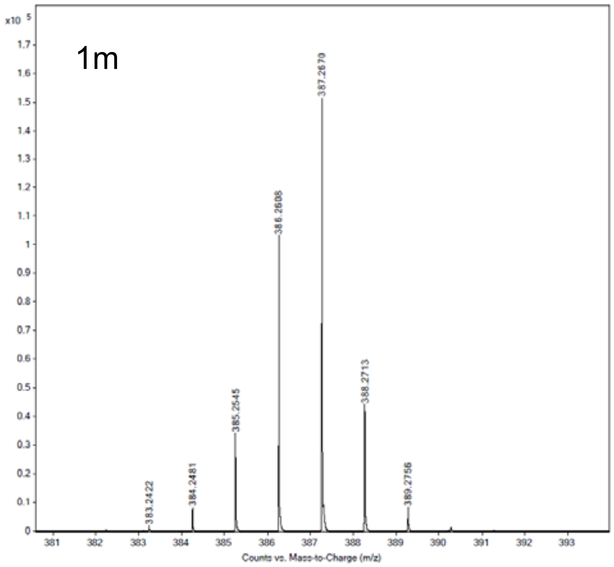
1m
x10 5
387.2670
386.2608
388.2713
385.2545
384.2481
383.2422
389.2756
Counts vs. Mass-to-Charge (m/z)

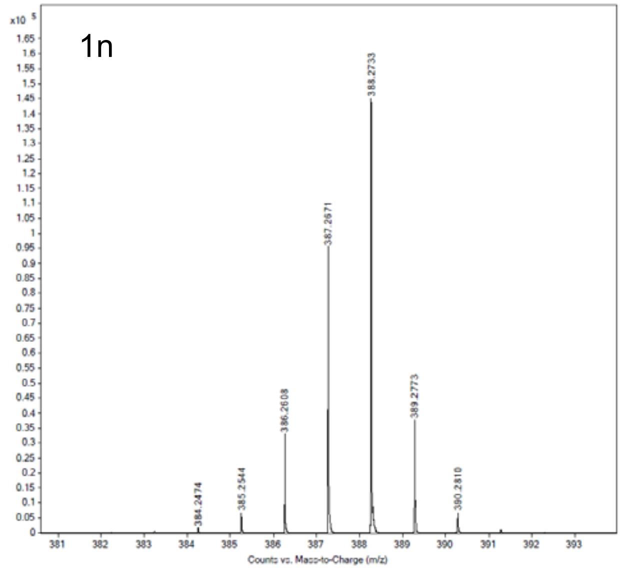
1n
x10 5
384.2474
385.2544
386.2608
387.2671
388.2733
389.2773
390.2810
Counts vs. Mass-to-Charge (m/z)

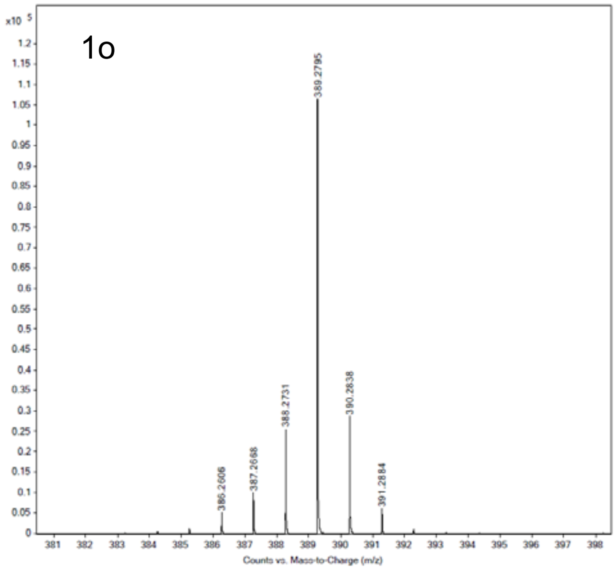
1o
x10 5
386.2606
387.2668
388.2731
389.2795
390.2838
391.2884
Counts vs. Mass-to-Charge (m/z)

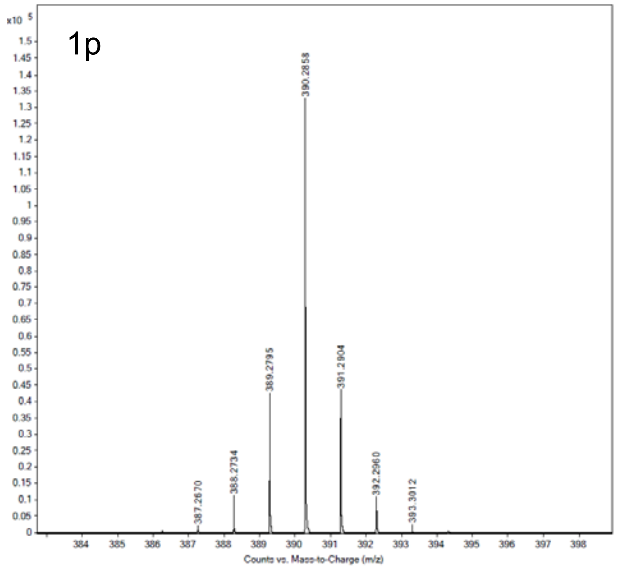
1p
x10 5
387.2670
388.2734
389.2795
390.2858
391.2904
392.2960
393.3012
Counts vs. Mass-to-Charge (m/z)

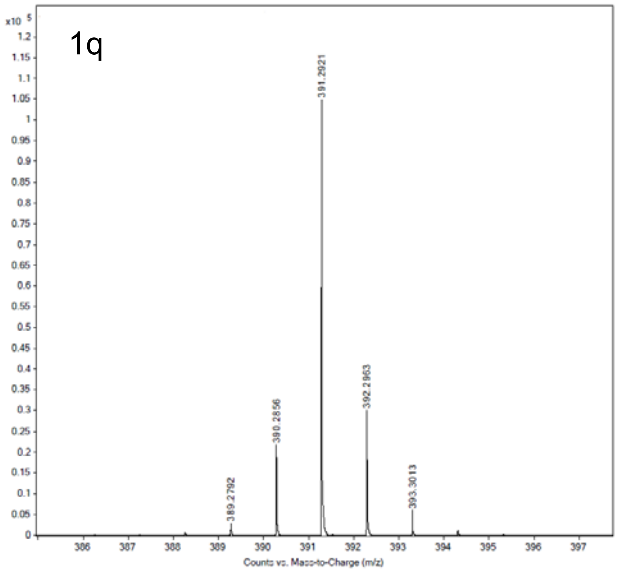
1q
x10 5
389.2792
390.2856
391.2921
392.2963
393.3013
Counts vs. Mass-to-Charge (m/z)

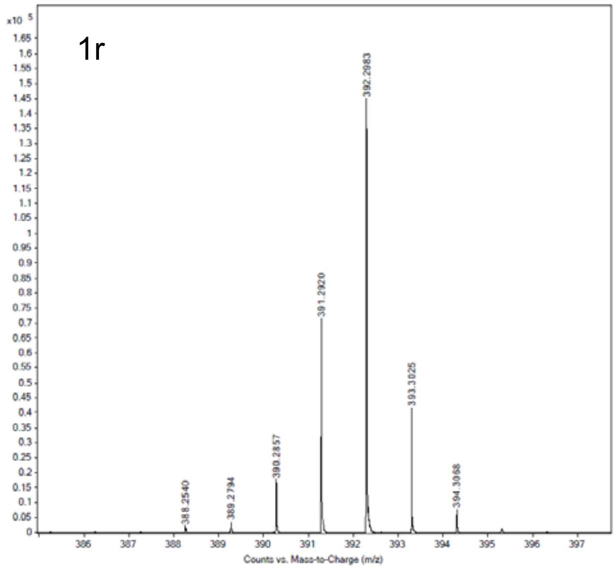
1r
x10 5
388.2540
389.2794
390.2857
391.2920
392.2983
393.3025
394.3068
Counts vs. Mass-to-Charge (m/z)

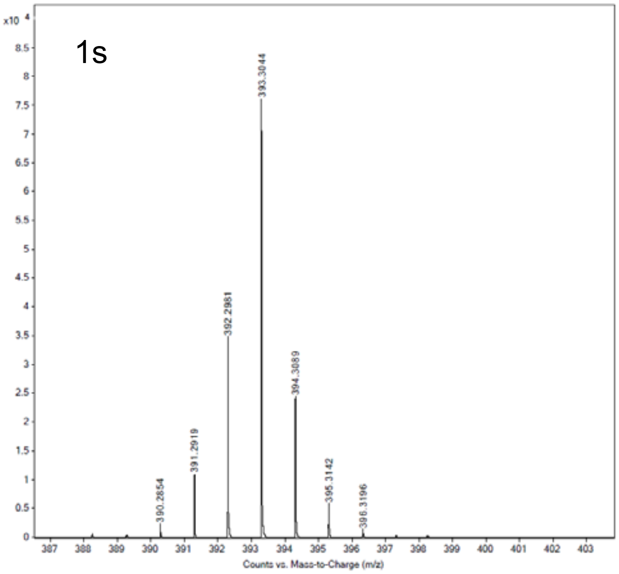
1s
x10 4
390.2854
391.2919
392.2981
393.3044
394.3089
395.3142
396.3196
Counts vs. Mass-to-Charge (m/z)

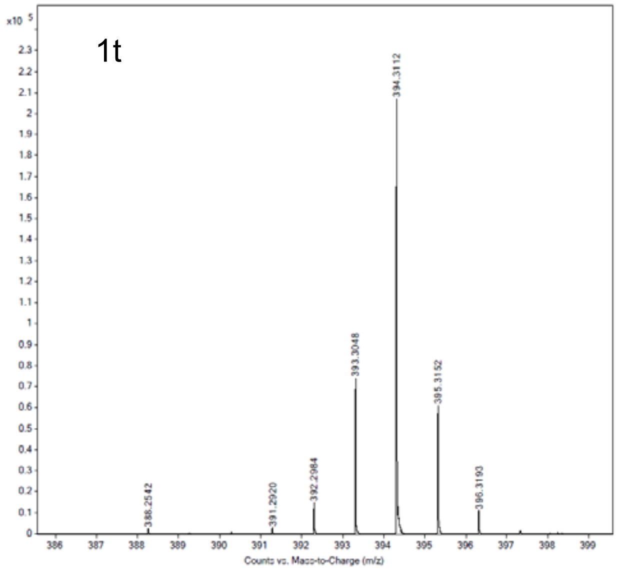
x10 5
1t
394.3112
393.3048
395.3152
396.3193
392.2984
391.2920
388.2542
Counts vs. Mass-to-Charge (m/z)

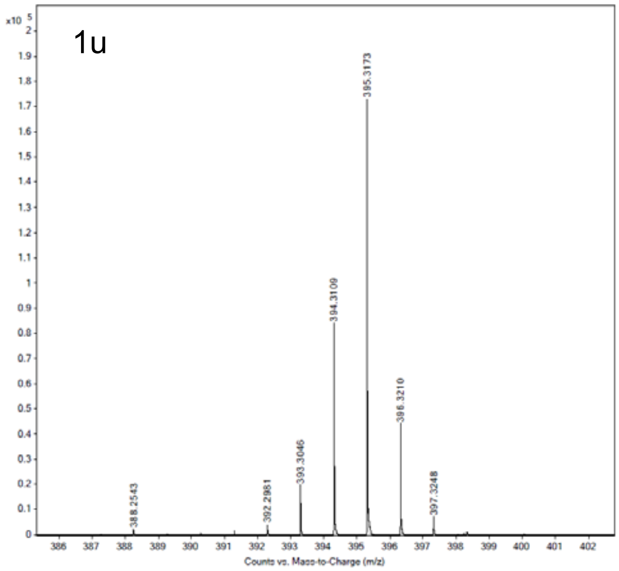
x10 5
1u
395.3173
394.3109
396.3210
393.3046
397.3248
392.2981
388.2543
Counts vs. Mass-to-Charge (m/z)

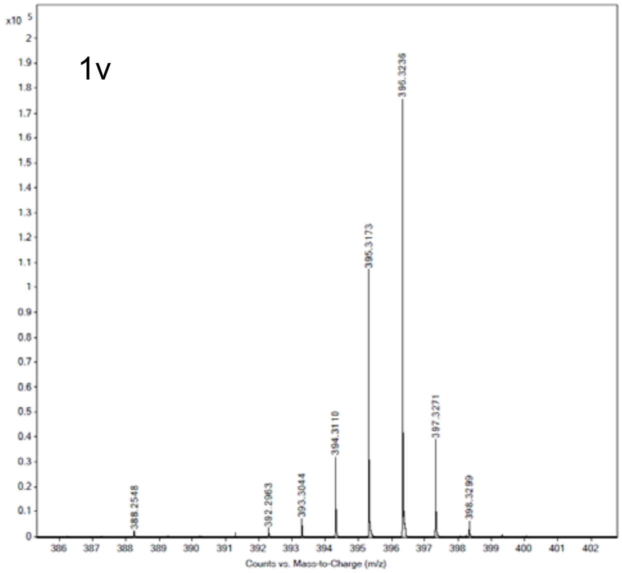
x10 5
1v
396.3236
395.3173
397.3271
394.3110
393.3044
392.2963
398.3299
388.2548
Counts vs. Mass-to-Charge (m/z)

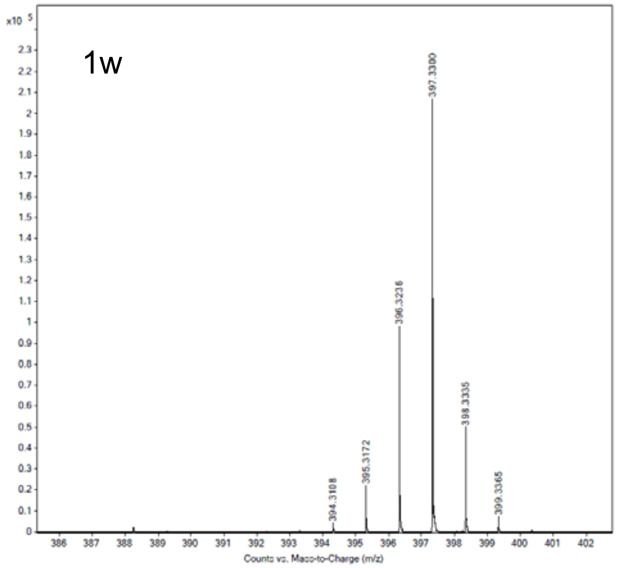
x10 5
1w
397.3300
396.3236
398.3335
395.3172
399.3365
394.3108
Counts vs. Mass-to-Charge (m/z)

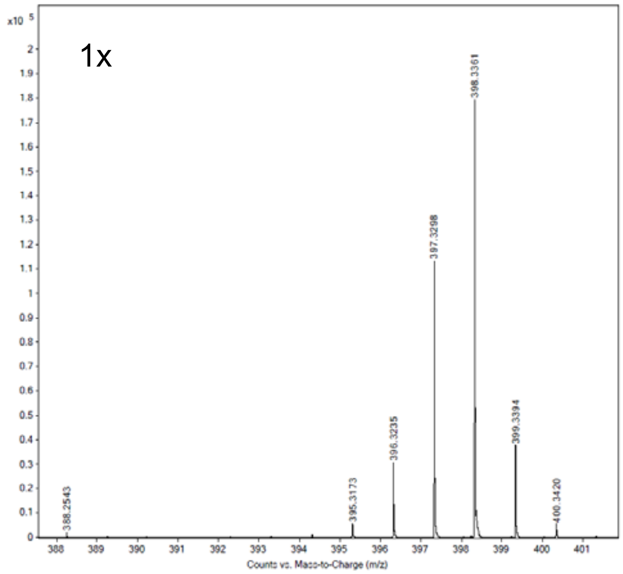
x10 5
1x
398.3361
397.3298
399.3394
396.3235
400.3420
395.3173
388.2543
Counts vs. Mass-to-Charge (m/z)

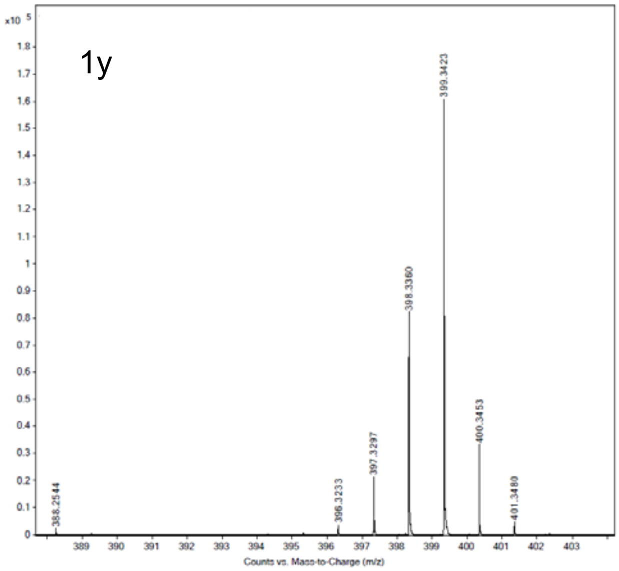
x10 5
1y
399.3423
398.3360
400.3453
397.3297
401.3489
396.3233
388.2544
Counts vs. Mass-to-Charge (m/z)

4.5 HRMS measurements of prepared mixtures

Table S6. Peak areas of [M+H]$^+$ masses of the most similar mixtures (NDP>0.9980) in HRMS spectra.

	375	376	377	378	379	380	381	382	383	384	385	386	387	388	389	390	391	392	393	394	395	396	397	398	399	400	401	402
**M1**	2614	8066	0	0	1460	0	13655	150605	4564957	1138053	201739	99025	210008	308058	90636	20886	6059	4268	0	0	0	0	0	0	0	0	0	0
**M2**	9662	1535	0	0	0	0	18979	161499	4719606	1215590	252921	228267	306045	93553	24995	7282	0	0	0	0	0	0	0	0	0	0	0	0
**M3**	5264792	1094150	172700	29221	2315	1653	1096	5342	8618	10626	16725	61696	176783	267295	77073	20366	0	0	0	0	0	0	0	0	0	0	0	0
**M4**	4679549	997209	153026	25284	3415	1076	0	3892	14964	14896	57048	165325	251669	83312	21539	4212	0	0	0	0	0	0	0	0	0	0	0	0
**M5**	0	0	0	0	0	12949	903288	296270	57570	10049	7640	15887	40157	56626	17233	6683	0	0	0	0	0	0	0	0	0	0	0	0
**M6**	0	0	0	0	0	47308	4360468	1378959	237916	46269	64913	177236	258484	82296	22352	2421	0	0	0	0	0	0	0	0	0	0	0	0
**M7**	4703738	1386545	242878	32711	5832	0	0	8216	11287	12123	19128	62291	174448	262346	77285	17344	0	0	0	0	0	0	0	0	0	0	0	0
**M8**	4783050	1446059	242889	40613	6585	0	2838	8057	13525	22848	68132	194740	297726	89633	29338	12341	0	0	0	0	0	0	0	0	0	0	0	0
**M15**	0	0	0	0	0	0	0	3565	37558	228072	1278448	3089850	921215	375301	86794	19250	2389	0	0	0	0	0	0	0	0	0	0	0
**M16**	0	0	0	0	0	0	0	9055	37273	256886	1393682	3380166	1057756	206448	35633	10975	0	0	0	0	0	0	0	0	0	0	0	0
**M17**	0	0	0	0	5160	47503	4663849	948835	144113	25534	22068	60410	160647	240007	69528	15089	4707	0	0	0	0	0	0	0	0	0	0	0
**M18**	0	0	0	0	6015	45438	4501339	928886	146049	30369	66847	171253	259187	78102	19043	4870	0	0	0	0	0	0	0	0	0	0	0	0
**M19**	0	0	0	0	0	47052	4684135	950589	151369	46127	140629	310650	85025	20164	0	0	0	0	0	0	0	0	0	0	0	0	0	0
**M20**	0	0	0	11055	92803	471557	4414499	912694	137355	24948	19509	61426	173773	263396	79480	15511	0	0	0	0	0	0	0	0	0	0	0	0
**M21**	0	0	0	13134	89210	442349	4310275	887976	133882	36368	59427	165893	241845	75233	26455	6599	0	0	0	0	0	0	0	0	0	0	0	0
**M22**	5333470	1106010	167196	24327	4920	0	1455	9246	13465	32945	162064	361437	100846	21386	2570	0	0	0	0	0	0	0	0	0	0	0	0	0
**M23**	155512	3619425	757272	118579	16271	0	2383	11712	12981	7223	14598	59331	163622	243255	70890	16175	0	0	0	0	0	0	0	0	0	0	0	0
**M24**	169405	3883098	815736	128659	16679	0	1473	13884	13240	19978	61512	179873	288213	83680	24197	5883	0	0	0	0	0	0	0	0	0	0	0	0
**M25**	37451	298310	5587049	1431126	234429	34589	13941	15332	21072	13243	20233	77595	220492	336446	98837	22991	5557	2127	0	0	0	0	0	0	0	0	0	0
**M26**	35784	295755	5542123	1390963	230094	35051	11814	21089	25308	25577	68983	209624	305125	100164	26662	9491	0	0	0	0	0	0	0	0	0	0	0	0
**M27**	4163	4536	0	4584	7482	5423	231841	5204724	1053976	155923	40942	76539	206216	308864	92887	22920	6966	2692	0	0	0	0	0	0	0	0	0	0
**M28**	7994	3569	6492	1569	1772	10384	221325	5076650	1031399	169956	91769	204284	307044	93548	24043	9805	1334	0	0	0	0	0	0	0	0	0	0	0

Table S7. Peak areas of [M+H]$^+$ masses of the prepared ternary, quaternary mixtures (NDP>0.9970), a 10-membered mixture and M36A-C quaternary mixtures in HRMS spectra.

	375	376	377	378	379	380	381	382	383	384	385	386	387	388	389	390	391	392	393	394	395	396	397	398	399	400	401	402
**M9**	20315	32935	220402	1077434	263449	47661	16138	20468	107752	536665	2732394	6832446	4297863	1152964	246177	53562	27755	3450	0	0	0	0	0	0	0	0	0	0
**M10**	43813	34540	209497	1027329	259837	58812	23148	24919	93161	525303	2658340	6592066	4118049	1660568	407549	89924	22609	7818	4622	0	0	0	0	0	0	0	0	0
**M11**	1140014	252167	46276	0	0	106686	8792539	1915861	335390	115721	402716	939998	643102	673447	193572	39342	12598	0	0	0	0	0	0	0	0	0	0	0
**M12**	1108048	263370	44028	0	0	99827	8489559	1918922	320583	148576	526014	1256268	886604	266207	63083	20687	10620	0	0	0	0	0	0	0	0	0	0	0
**M13**	8066877	3577747	679227	105758	19046	6659	9528	24651	9426	11206	46475	156081	456233	699754	208887	50879	37738	20294	103717	383089	810786	235720	190863	70273	24168	9102	4594	0
**M14**	8075019	3784415	695954	111564	23147	2982	38797	21690	15559	45304	166570	462072	705732	232218	60156	23934	34661	34797	99726	382452	814349	239861	190996	89019	30018	7478	0	0
**M29**	0	0	0	0	3981	3747	362013	7620366	1683557	262352	63424	156529	431056	673611	191225	45895	19805	18473	98292	362031	761567	336143	478682	757510	184477	34892	17849	0
**M30**	0	0	0	0	0	8388	349079	7446736	1624249	281099	177999	426912	638754	210392	48364	23126	10755	27615	95689	358244	739716	322249	454612	750362	176853	34200	0	0
**M31**	681948	182046	35477	0	0	0	14732	143526	4010877	1332015	795555	380654	403181	128626	26774	9115	9546	729	0	0	0	0	0	0	0	0	0	0
**M32**	691532	191817	37603	4149	0	0	14313	146743	4014930	1308394	714751	228059	270036	370192	110886	23583	10136	0	0	0	0	0	0	0	0	0	0	0
**M33**	166325	3532126	822250	126282	20651	0	0	0	6839	21900	83609	239507	356244	111607	27210	2382	14755	9102	21954	80212	248854	435578	179654	227667	499296	117048	19597	0
**M34**	168245	3581997	807414	139629	21912	0	0	0	0	0	24809	83175	240215	354911	105655	28015	15086	11916	31735	85644	254159	442280	177881	235525	481375	105607	24220	3150
**M35**	3021378	2777505	638218	574894	1819332	449538	115691	120494	2466816	907844	1159488	2499056	1635250	1745836	570638	404586	1095486	2555795	2419330	1142540	1318229	1950460	851614	1197607	2053454	489484	125088	57849
**M36A**	0	4682	132272	658965	157790	28131	7447	26488	653864	182985	62129	107334	293495	435033	124157	27701	17388	47140	206264	571801	174335	42909	1447	0	0	0	0	0
**M36B**	3715	39553	216876	476527	106835	21558	2501	28437	596577	172664	55538	95531	267909	411728	119293	33699	17503	40648	183244	517841	155268	41110	5485	0	0	0	0	0
**M36C**	0	11723	120649	606225	152545	32478	35892	140508	408905	160222	56961	98527	278684	429068	120757	25215	14560	39929	189723	536244	165230	37198	4855	0	0	0	0	0
**M36D**	12648	38393	220356	474928	112391	23228	38896	141797	410586	158531	54694	98499	268068	386800	117422	23946	15844	39677	197529	520297	153842	35751	4371	0	0	0	0	0

Figure S3. HRMS of mixtures **M1-M36D**

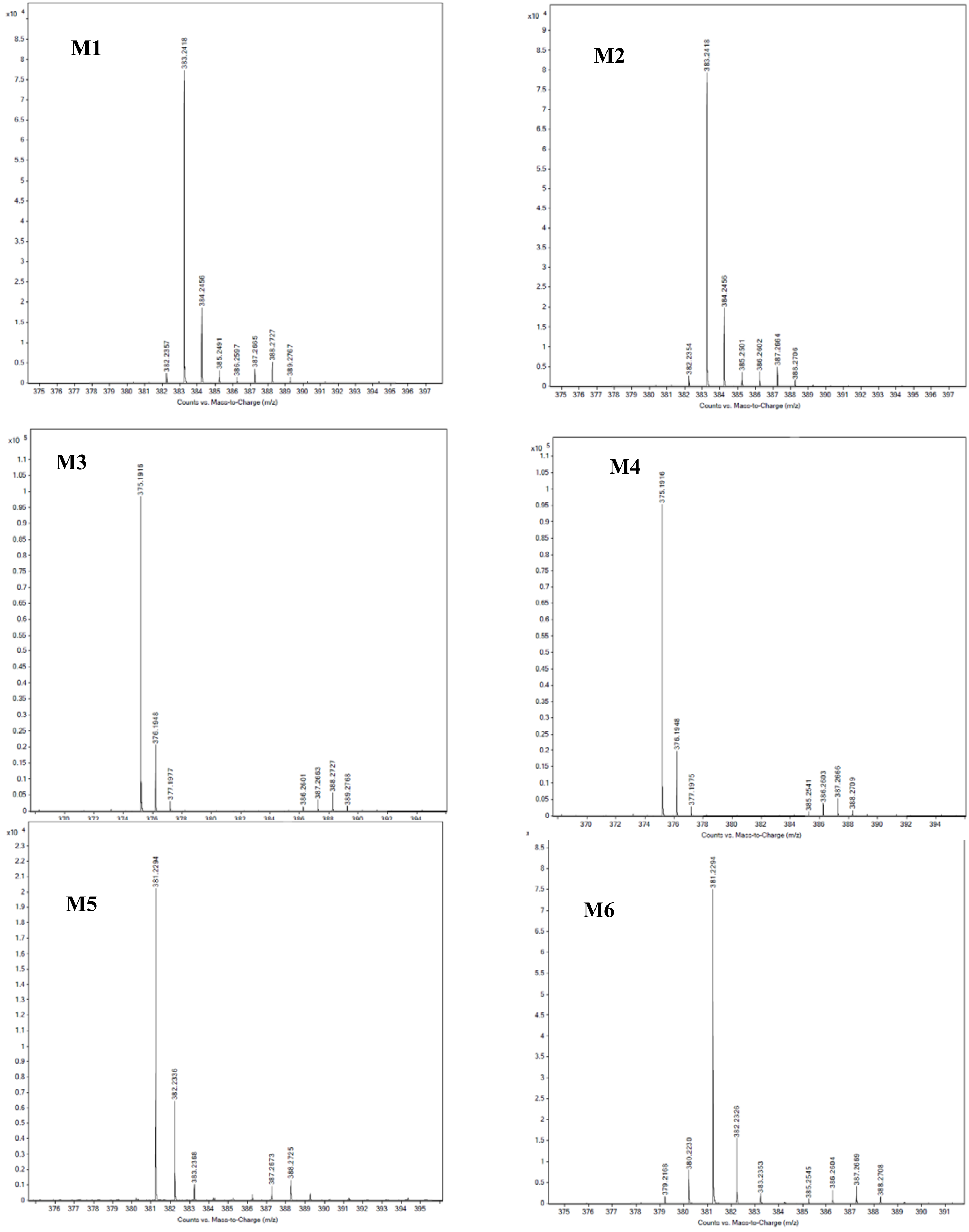

M7

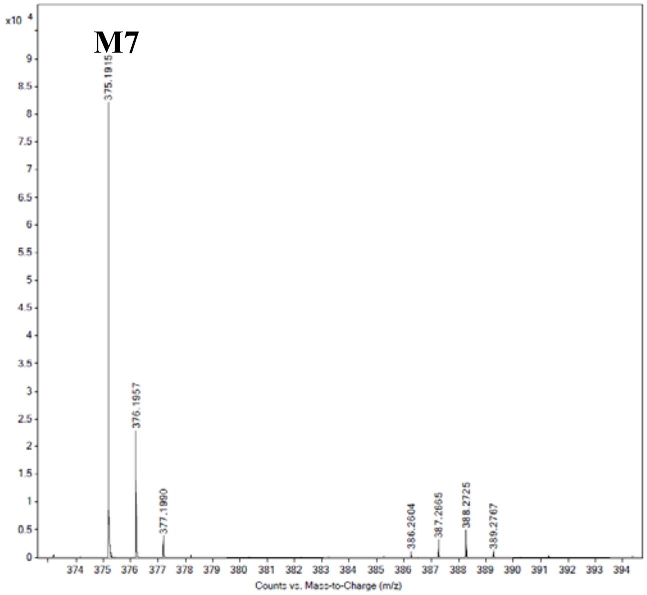

M8

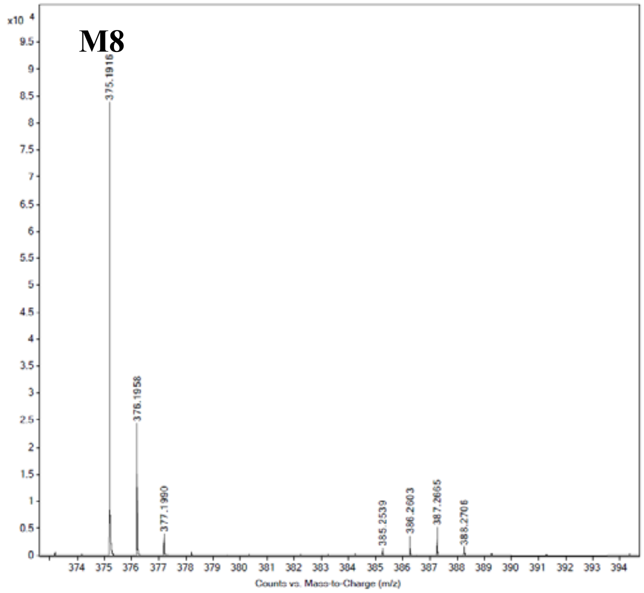

M9

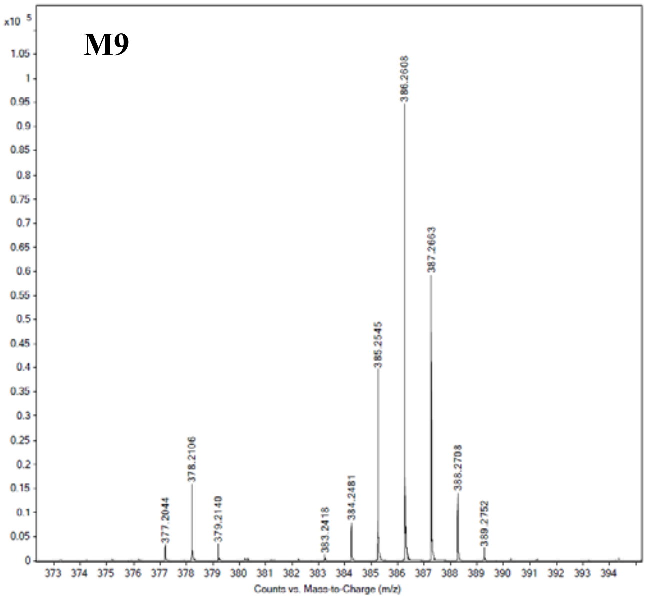

M10

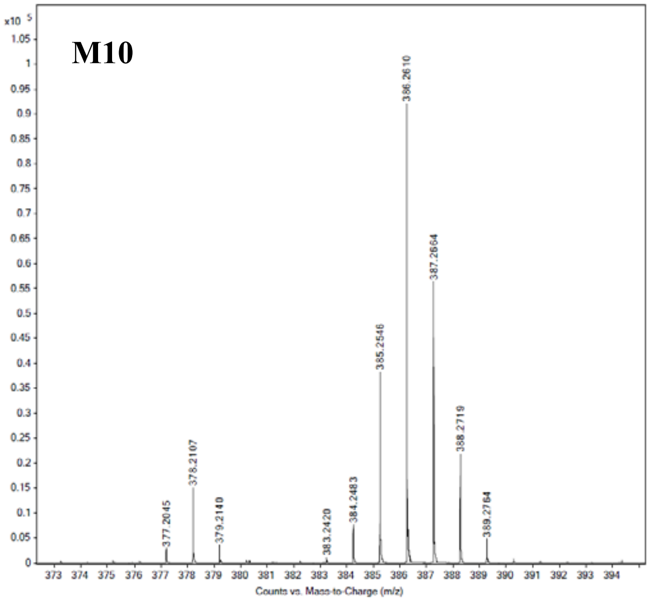

M11

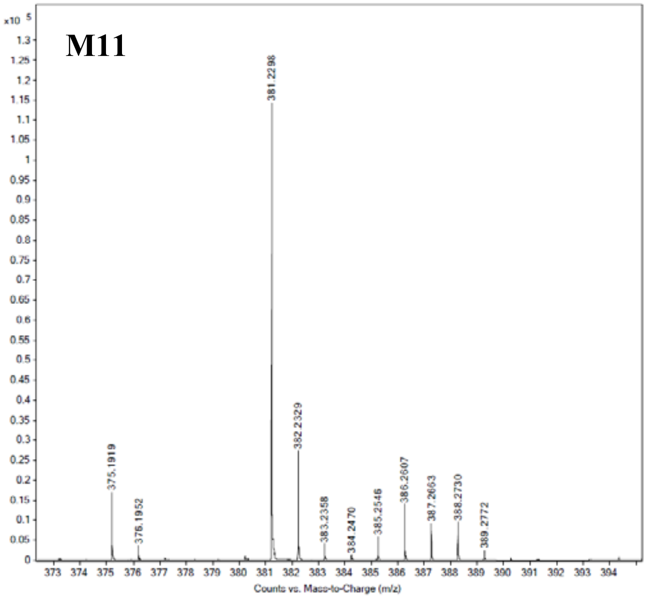

M12

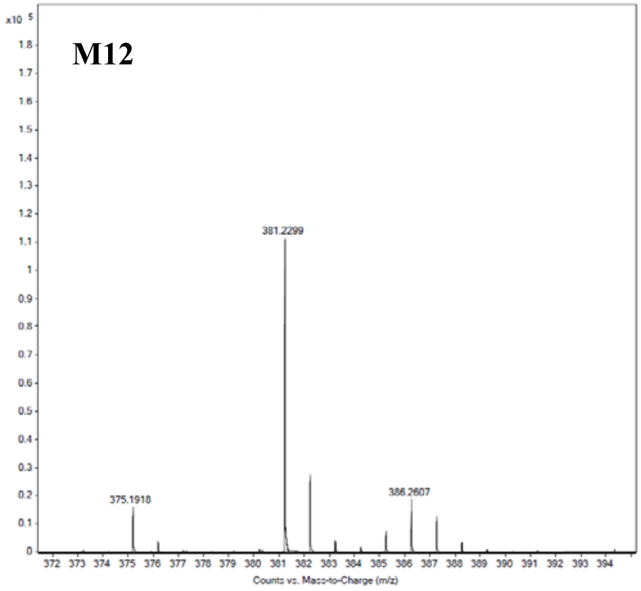

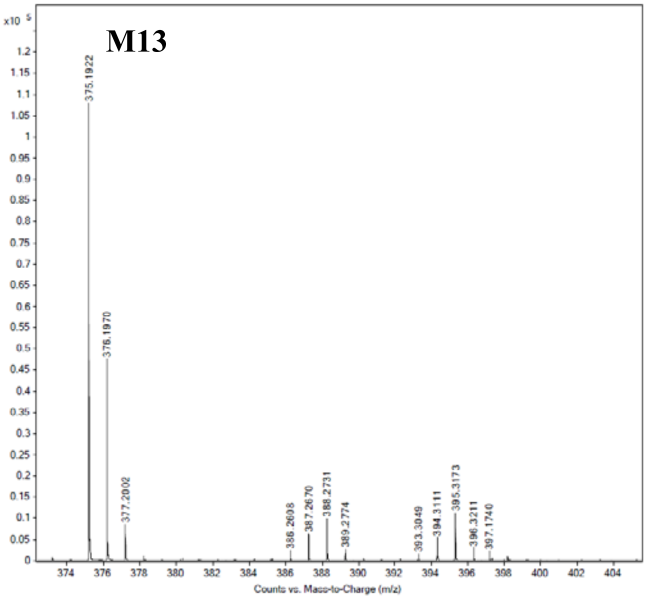
M13
375.1922
376.1970
377.2002
386.2608
387.2670
388.2731
389.2774
393.3049
394.3111
395.3173
396.3211
397.1740
Counts vs. Mass-to-Charge (m/z)

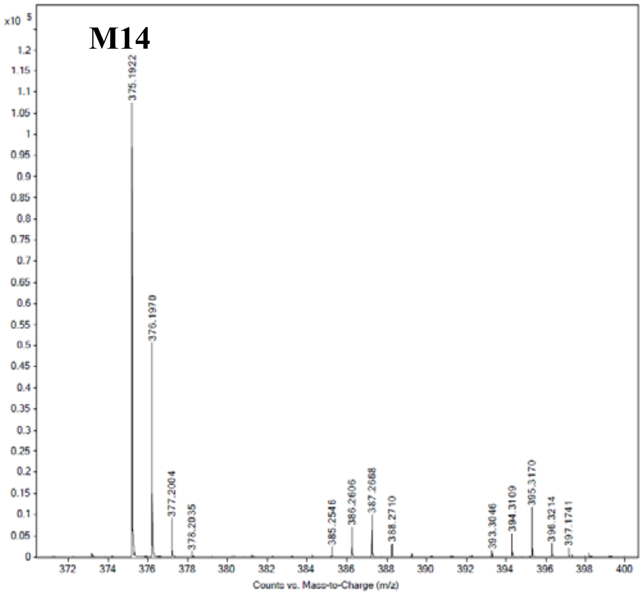
M14
375.1922
376.1970
377.2004
378.2035
385.2546
386.2606
387.2668
388.2710
393.3046
394.3109
395.3170
396.3214
397.1741
Counts vs. Mass-to-Charge (m/z)

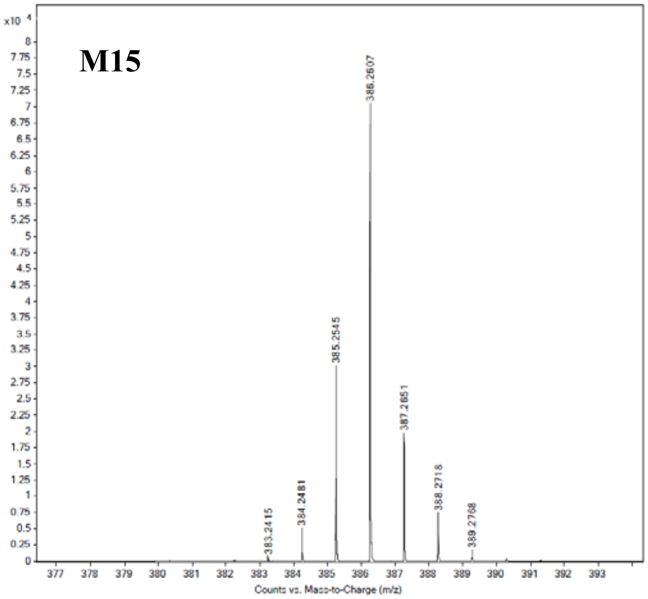
M15
383.2415
384.2481
385.2545
386.2607
387.2651
388.2718
389.2768
Counts vs. Mass-to-Charge (m/z)

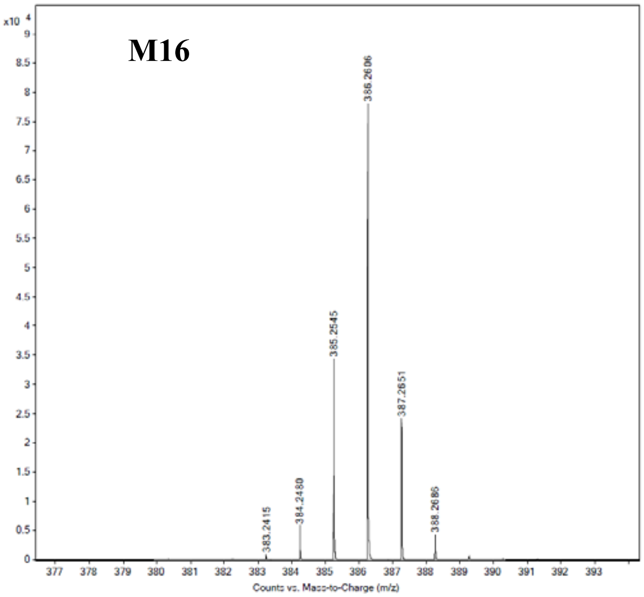
M16
383.2415
384.2480
385.2545
386.2606
387.2651
388.2686
Counts vs. Mass-to-Charge (m/z)

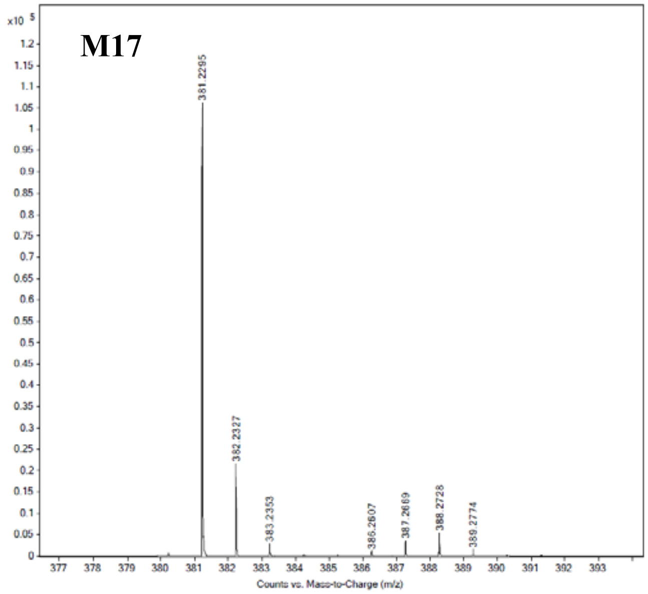
M17
381.2295
382.2327
383.2353
386.2607
387.2669
388.2728
389.2774
Counts vs. Mass-to-Charge (m/z)

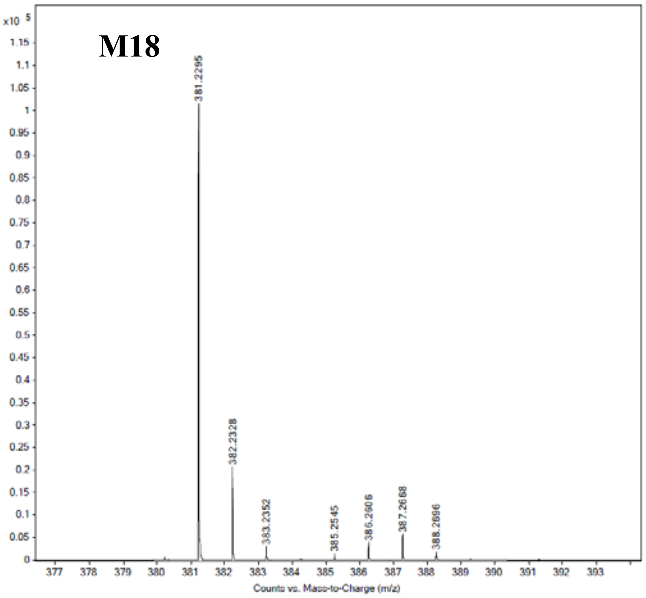
M18
381.2295
382.2328
383.2352
385.2545
386.2606
387.2668
388.2696
Counts vs. Mass-to-Charge (m/z)

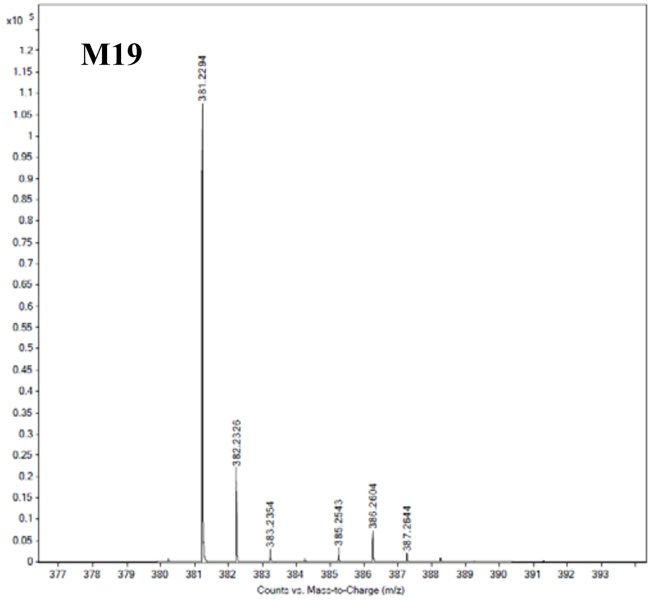
M19
x10 5
381.2294
382.2326
383.2354
385.2543
386.2604
387.2644
Counts vs. Mass-to-Charge (m/z)

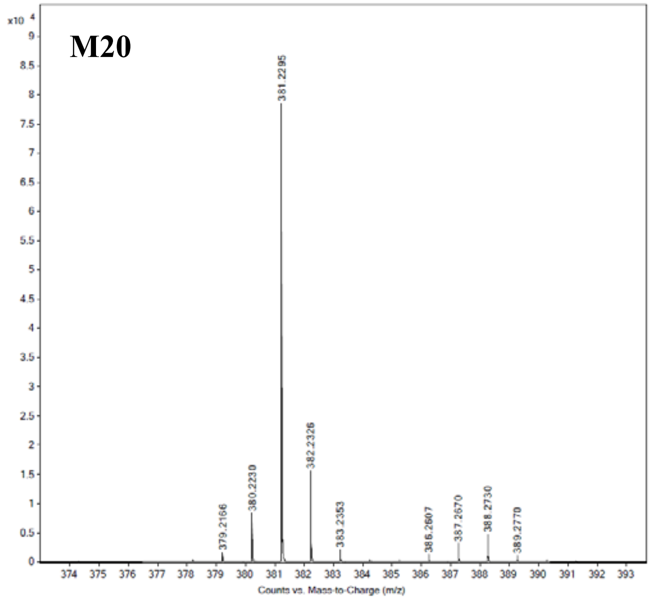
M20
x10 4
381.2295
379.2166
380.2230
382.2326
383.2353
386.2607
387.2670
388.2730
389.2770
Counts vs. Mass-to-Charge (m/z)

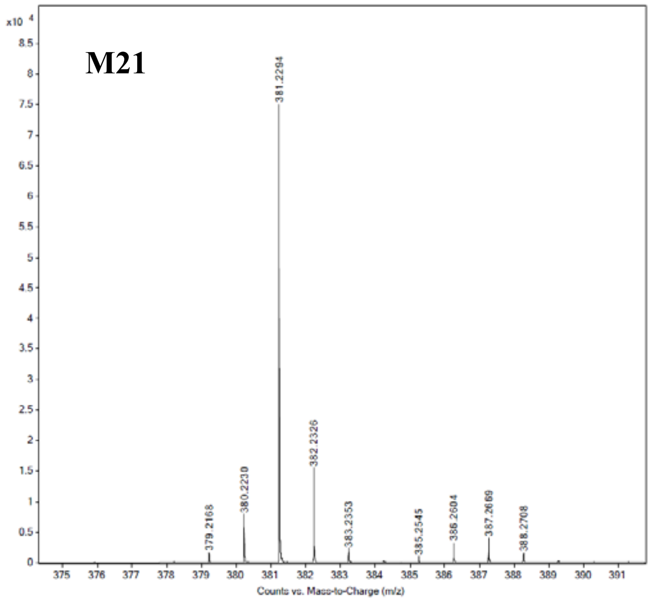
M21
x10 4
381.2294
379.2168
380.2230
382.2326
383.2353
385.2545
386.2604
387.2669
388.2708
Counts vs. Mass-to-Charge (m/z)

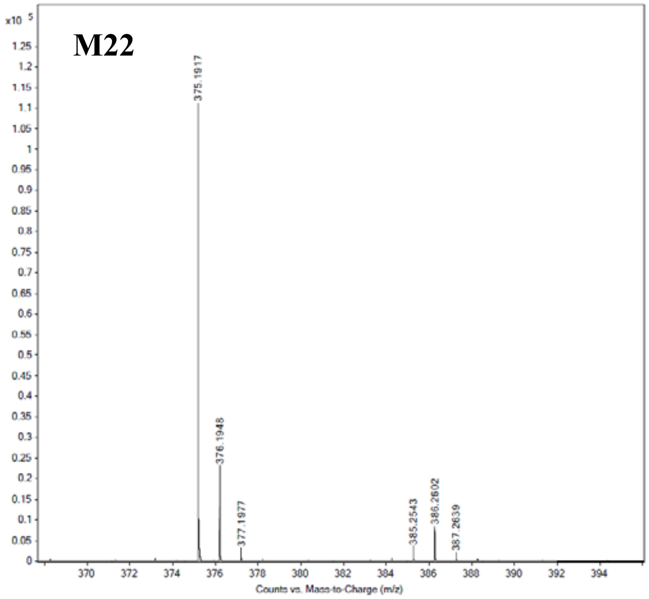
M22
x10 5
375.1917
376.1948
377.1977
385.2543
386.2602
387.2639
Counts vs. Mass-to-Charge (m/z)

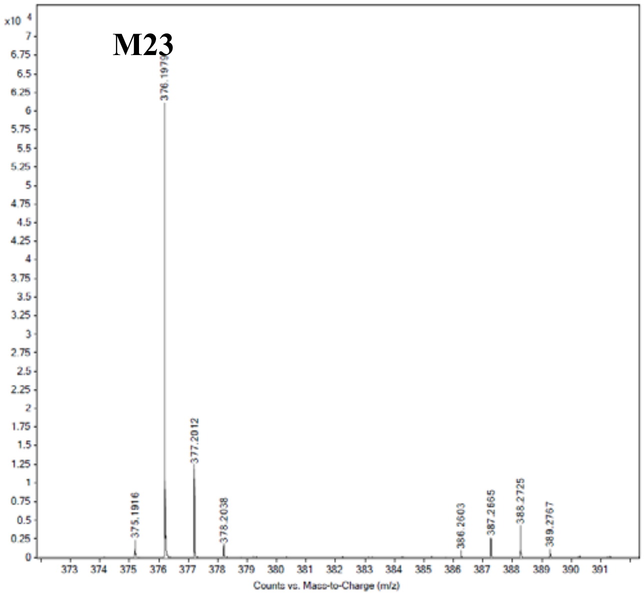
M23
x10 4
375.1916
377.2012
378.2038
386.2603
387.2665
388.2725
389.2767
Counts vs. Mass-to-Charge (m/z)

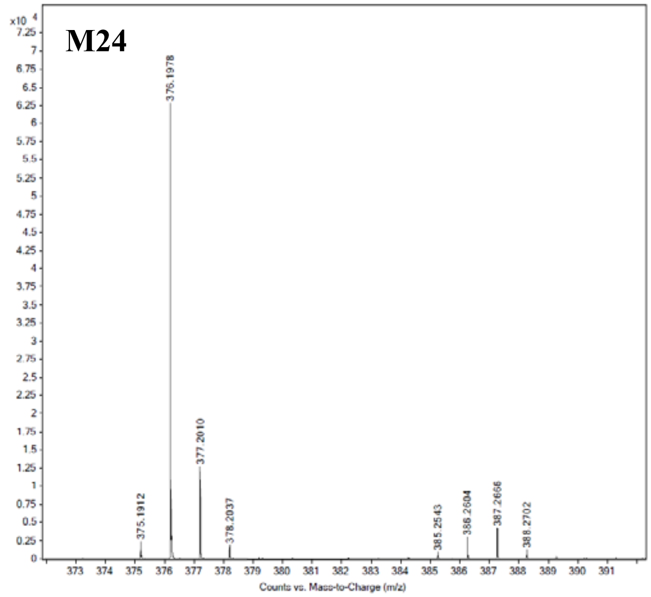
M24
x10 4
376.1978
375.1912
377.2010
378.2037
385.2543
386.2604
387.2666
388.2702
Counts vs. Mass-to-Charge (m/z)

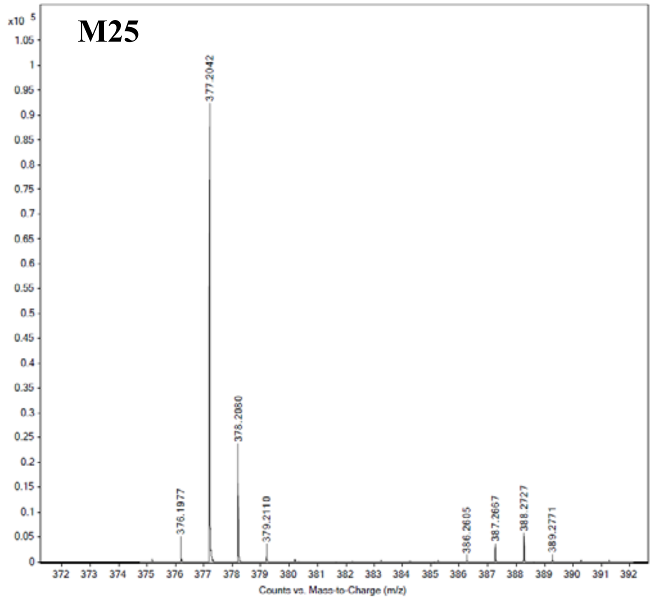
M25
x10 5
377.2042
378.2080
376.1977
379.2110
386.2605
387.2667
388.2727
389.2771
Counts vs. Mass-to-Charge (m/z)

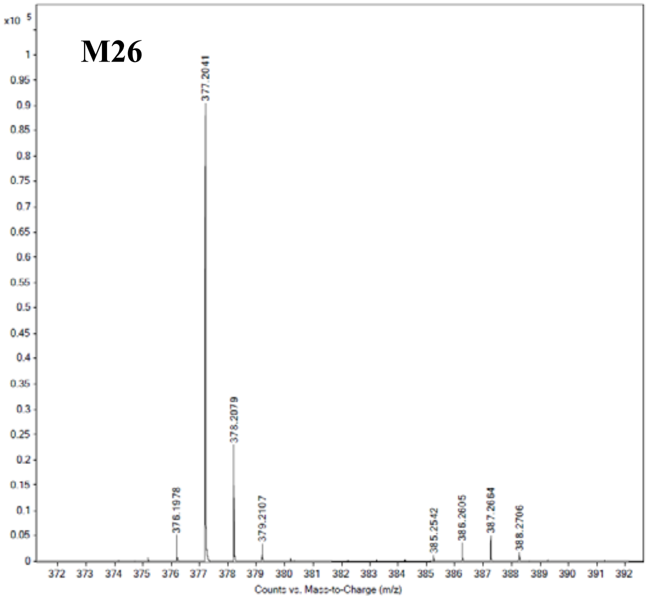
M26
x10 5
377.2041
378.2079
376.1978
379.2107
385.2542
386.2605
387.2664
388.2706
Counts vs. Mass-to-Charge (m/z)

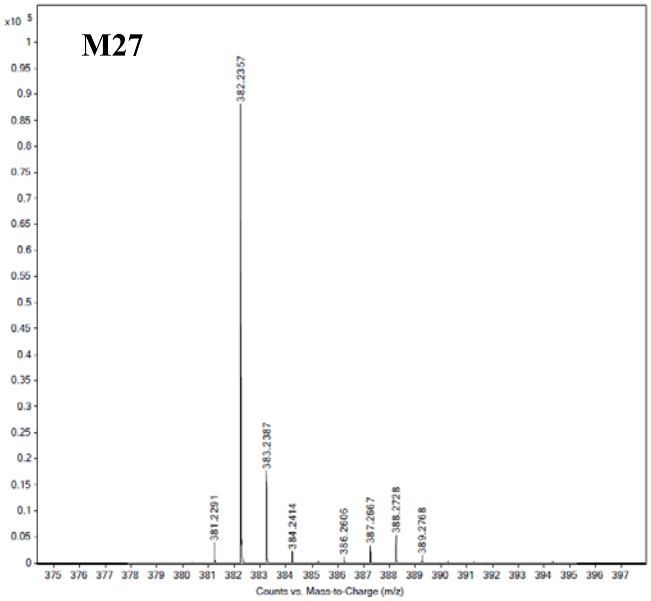
M27
x10 5
382.2357
383.2387
381.2291
384.2414
386.2606
387.2667
388.2728
389.2768
Counts vs. Mass-to-Charge (m/z)

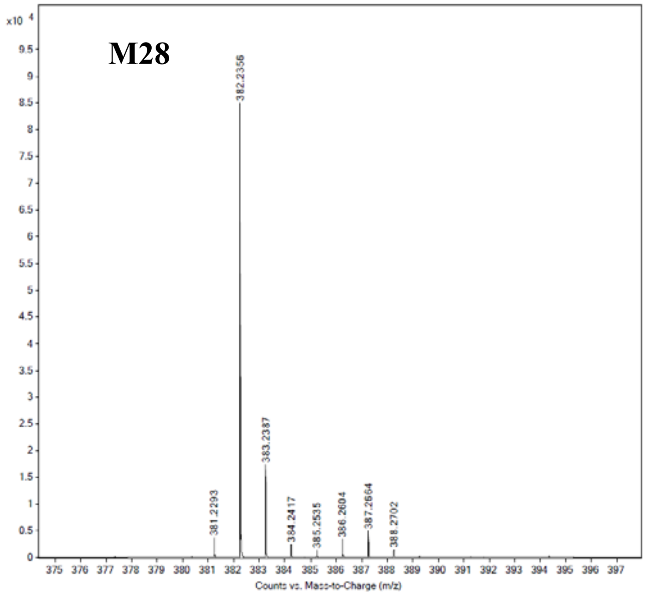
M28
x10 4
382.2356
383.2387
381.2293
384.2417
385.2535
386.2604
387.2664
388.2702
Counts vs. Mass-to-Charge (m/z)

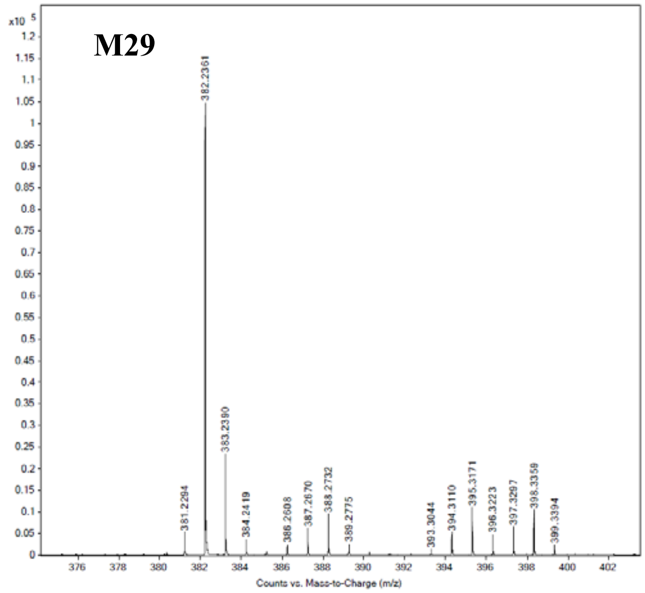
M29
x10 5
382.2361
383.2390
381.2284
384.2419
386.2608
387.2670
388.2732
389.2775
393.3044
394.3110
395.3171
396.3223
397.3297
398.3358
399.3394
Counts vs. Mass-to-Charge (m/z)

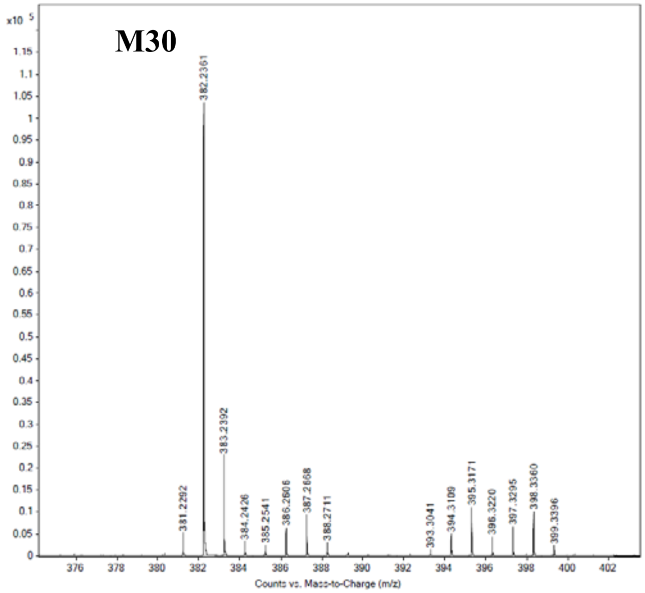
M30
x10 5
382.2361
383.2392
381.2292
384.2426
385.2541
386.2606
387.2668
388.2711
393.3041
394.3109
395.3171
396.3220
397.3295
398.3360
399.3396
Counts vs. Mass-to-Charge (m/z)

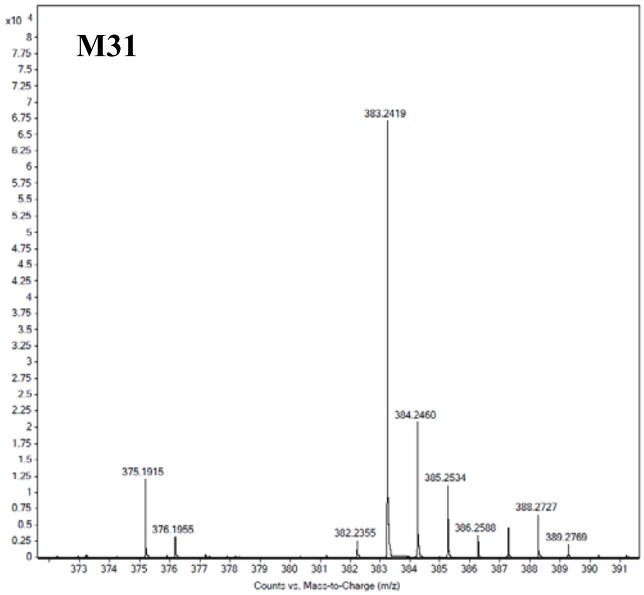
M31
383.2419
384.2460
375.1915
385.2534
376.1955
382.2355
386.2588
388.2727
389.2769
Counts vs. Mass-to-Charge (m/z)

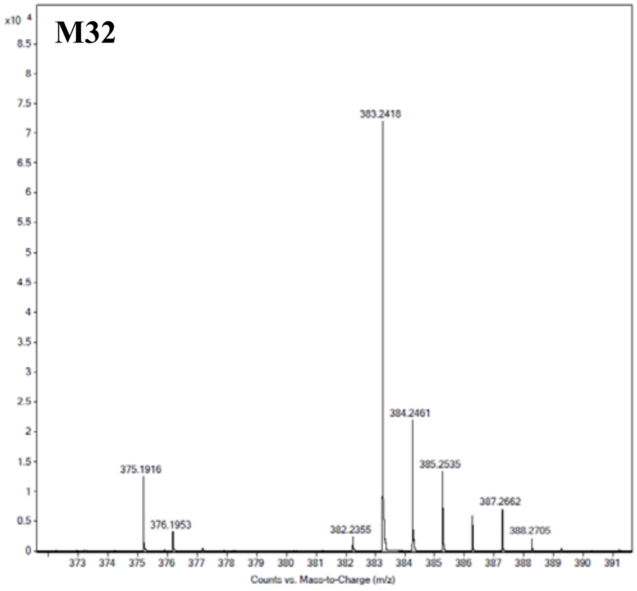
M32
383.2418
384.2461
375.1916
385.2535
387.2662
376.1953
382.2355
388.2705
Counts vs. Mass-to-Charge (m/z)

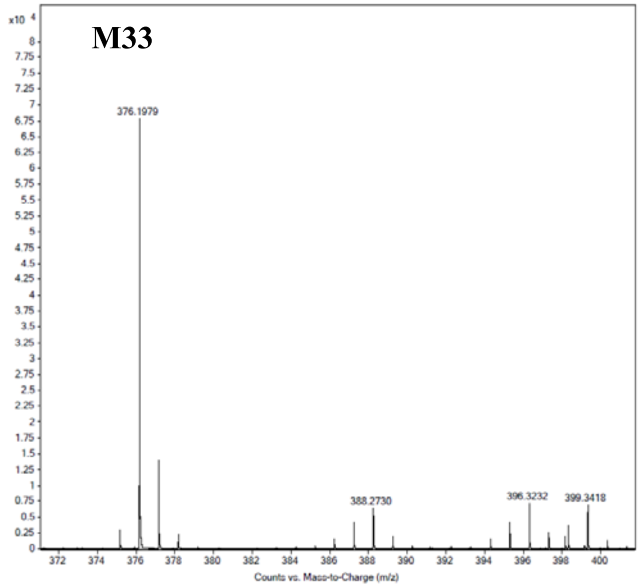
M33
376.1979
388.2730
396.3232
399.3418
Counts vs. Mass-to-Charge (m/z)

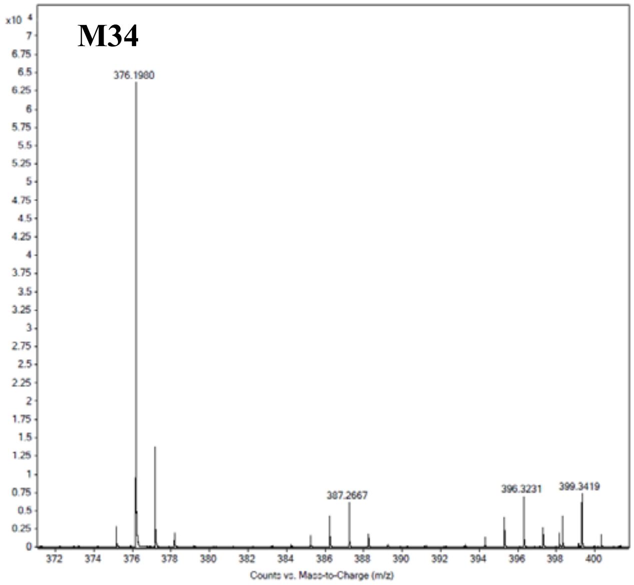
M34
376.1980
387.2667
396.3231
399.3419
Counts vs. Mass-to-Charge (m/z)

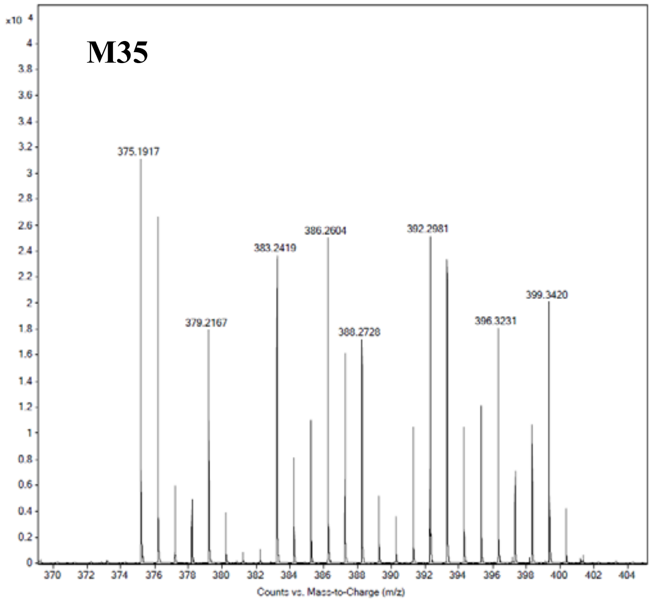
M35
375.1917
383.2419
386.2604
392.2981
379.2167
388.2728
399.3420
396.3231
Counts vs. Mass-to-Charge (m/z)

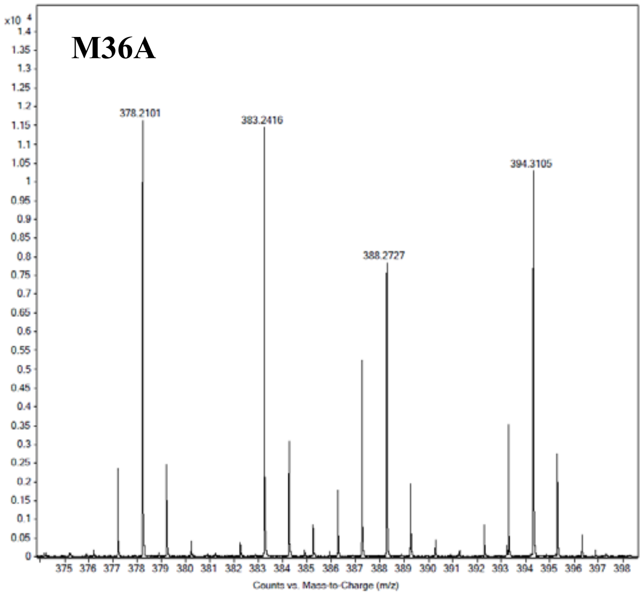
M36A
x10 4
378.2101
383.2416
388.2727
394.3105
Counts vs. Mass-to-Charge (m/z)

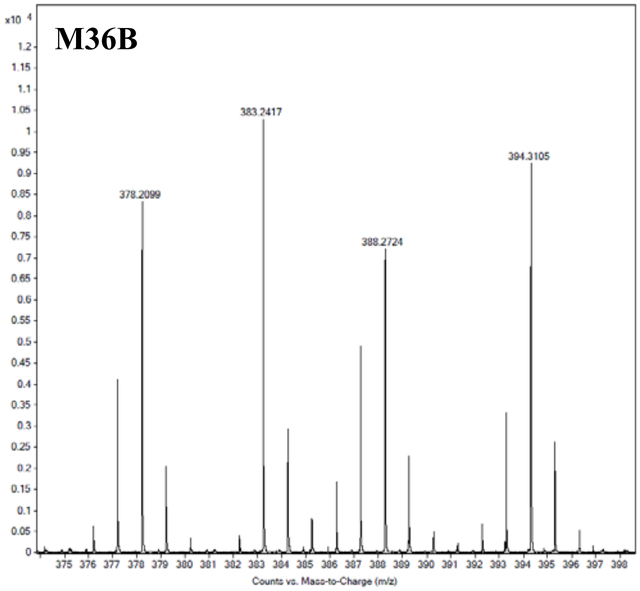
M36B
x10 4
378.2099
383.2417
388.2724
394.3105
Counts vs. Mass-to-Charge (m/z)

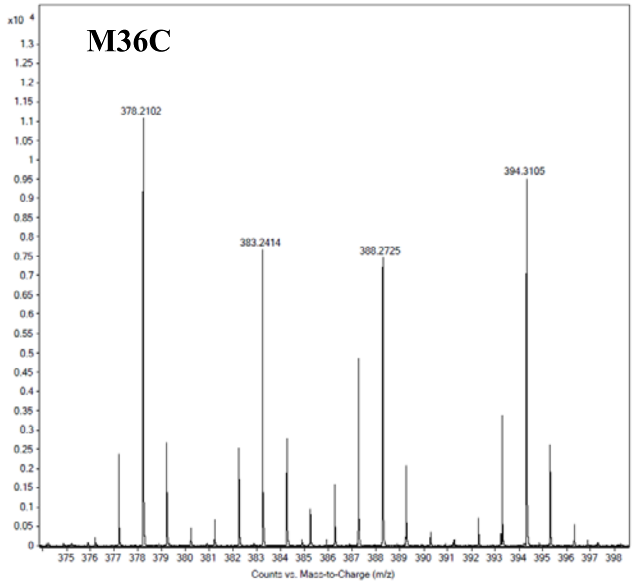
M36C
x10 4
378.2102
383.2414
388.2725
394.3105
Counts vs. Mass-to-Charge (m/z)

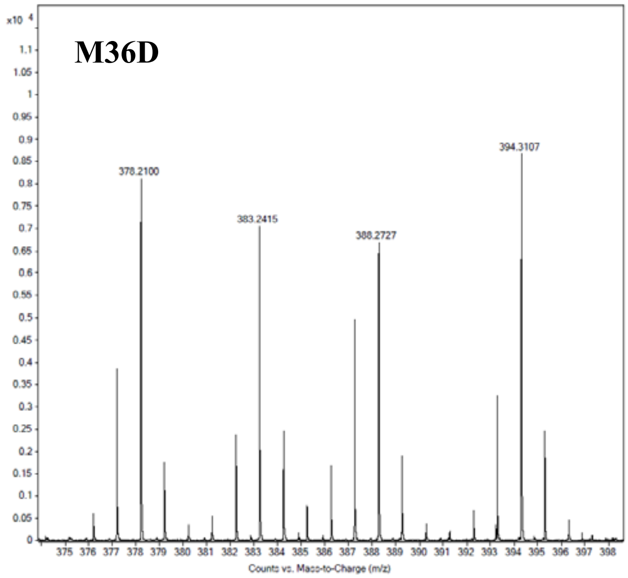
M36D
x10 4
378.2100
383.2415
388.2727
394.3107
Counts vs. Mass-to-Charge (m/z)

4.6 Amide coupling: procedure, HRMS measurements

Synthesis of deuterated 8-acetamido-*N*-benzyl-4,6-bis(3-methoxypropyl)quinoline-2-carboxamide mixture for HRMS analysis

A 20 mL, screw-cap vial equipped with a magnetic stirring bar was filled with a mixture of 11.60 mg **8-acetamido-4,6-bis(3-methoxypropyl)quinoline-2-carboxylic acid** (0.28 equiv., 0.0310 mmol), 9.92 mg **8-acetamido-4,6-bis[3-(trideuteriomethoxy)propyl]quinoline-2-carboxylic acid** (0.24 equiv., 0.0261 mmol), 10.16 mg **3-deuterio-8-[(2,2,2-trideuterioacetyl)amino]-4,6-bis[3-(trideuteriomethoxy)propyl]quinoline-2-carboxylic acid** (0.24 equiv., 0.0264 mmol), 10.12 mg **3-deuterio-4,6-bis[1,1,2,2-tetradeuterio-3-(trideuteriomethoxy)propyl]-8-[(2,2,2-trideuterioacetyl)amino]quinoline-2-carboxylic acid** (0.24 equiv., 0.0258 mmol) (**M37**), 62.8 mg benzotriazol-1-yloxy-tris(dimethylamino)phosphonium, hexafluorophosphate (1:1) (1.3 equiv., 0.142 mmol), and 38 µL DIPEA (2.0 equiv., 0.2187 mmol) and 3.29 mL dry, degassed DCM. The mixture was stirred at RT for 30 min, then 21.5 mg **1-naphthylmethanamine** (2.0 equiv., 0.2187 mmol) was added and the reaction mixture was stirred for further 30 min, until full conversion was observed. The mixture was concentrated *in vacuo*. Then the crude product was purified *via* RP-HPLC using 1% aq. HCOOH solution and MeCN as eluents to yield 28 mg differently deuterated mixture of **8-acetamido-*N*-benzyl-4,6-bis(3-methoxypropyl)quinoline-2-carboxamide**.(**M38**)

Table S8. Peak areas of $[M+H]^+$ masses of the measured carboxamide mixture (M37) in HRMS spectra

	375	376	377	378	379	380	381	382	383	384	385	386	387	388	389	390	391	392	393	394	395	396	397
integrate area	18129928	4041777	644526	114025	57289	165453	13711766	3165833	1218057	4138079	11032209	2491994	434549	100996	95435	313792	1337414	4212976	9006071	3243952	969887	260191	0
integrate ratio	0.2298	0.0512	0.0082	0.0014	0.0007	0.0021	0.1738	0.0401	0.0154	0.0525	0.1398	0.0316	0.0055	0.0013	0.0012	0.0040	0.0170	0.0534	0.1142	0.0411	0.0123	0.0033	0

Table S9. Peak integrate ratios of $[M+H]^+$ masses of the calculated and measured amine in HRMS spectra

	158	159	160	161
Calculated integrate ratio	0.8834	0.1101	0.0063	0.0002
Measured integrate ratio	0.8821	0.1089	0.0082	0.0007

Table S10. Peak areas and integral ratios of [M+H]$^+$ masses of the calculated and measured adduct mixture (M38) in HRMS spectra

	514	515	516	517	518	519	520	521	522	523	524	525	526	527	528	529	530	531	532	533	534	535	536
calculated integrate ratio	0.1959	0.0679	0.0142	0.0027	0.0009	0.0019	0.1484	0.0525	0.0188	0.0468	0.1249	0.0421	0.0092	0.0020	0.0012	0.0035	0.0149	0.0474	0.1032	0.0476	0.0158	0.0045	0
measured integrate area	28909460	11141681	2293640	444756	136901	256423	23058821	8660401	2998798	7426119	19972260	6918664	1512908	354659	297538	609663	2300787	7266433	15978770	7426798	2500198	733353	0
measured integrate ratio	0.1912	0.0737	0.0152	0.0029	0.0009	0.0017	0.1525	0.0573	0.0198	0.0491	0.1321	0.0458	0.0100	0.0023	0.0020	0.0040	0.0152	0.0481	0.1057	0.0491	0.0165	0.0049	0.0000

Calculated NDP for similarity between calculated and measured adduct mixture: 0.99934.

Figure S4. HRMS of compounds **M37-M38**

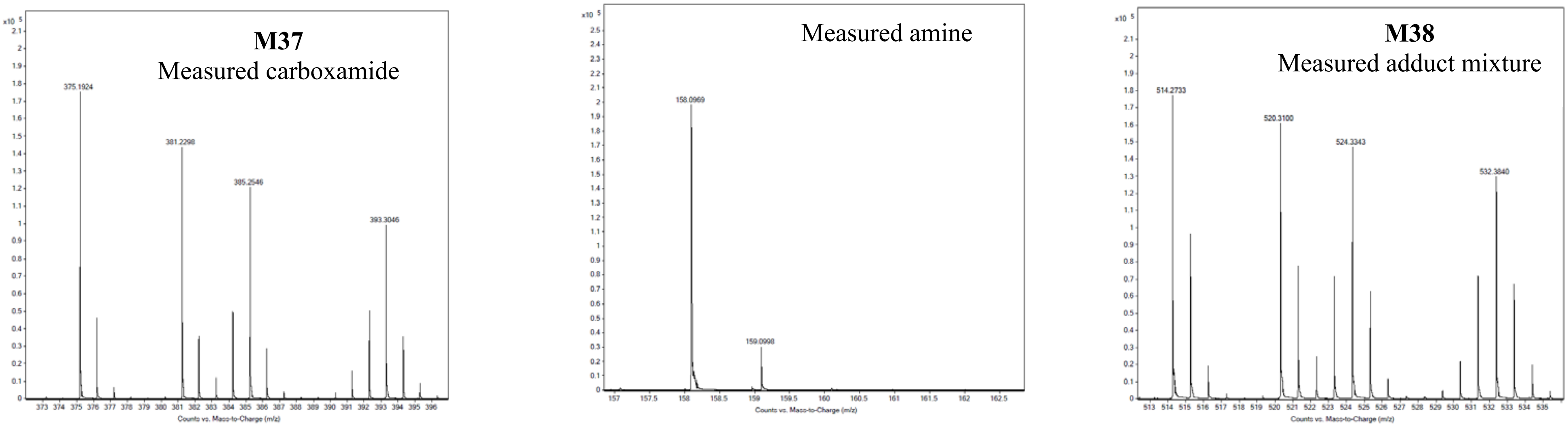

Supplement: SC-015-D4SC03519D-s001 [file SC-015-D4SC03519D-s001.pdf]
